# Comparing the Clique Percolation algorithm to other overlapping community detection algorithms in psychological networks: A Monte Carlo simulation study

**DOI:** 10.3758/s13428-024-02415-2

**Published:** 2024-05-01

**Authors:** Pedro Henrique Ribeiro Santiago, Gustavo Hermes Soares, Adrian Quintero, Lisa Jamieson

**Affiliations:** 1 Adelaide Dental School, The University of Adelaide, Level 4, 50 Rundle Mall, Rundle Mall Plaza, Adelaide, Australia; 2 ICFES - Colombian Institute for Educational Evaluation, Bogotá, Colombia

**Keywords:** Network psychometrics, Clique percolation, Community detection algorithms, Overlapping symptoms, Fuzzy Modularity

## Abstract

In psychological networks, one limitation of the most used community detection algorithms is that they can only assign each node (symptom) to a unique community, without being able to identify overlapping symptoms. The clique percolation (CP) is an algorithm that identifies overlapping symptoms but its performance has not been evaluated in psychological networks. In this study, we compare the CP with model parameters chosen based on fuzzy modularity (CPMod) with two other alternatives, the ratio of the two largest communities (CPRat), and entropy (CPEnt). We evaluate their performance to: (1) identify the correct number of latent factors (i.e., communities); and (2) identify the observed variables with substantive (and equally sized) cross-loadings (i.e., overlapping symptoms). We carried out simulations under 972 conditions (3x2x2x3x3x3x3): (1) data categories (continuous, polytomous and dichotomous); (2) number of factors (two and four); (3) number of observed variables per factor (four and eight); (4) factor correlations (0.0, 0.5, and 0.7); (5) size of primary factor loadings (0.40, 0.55, and 0.70); (6) proportion of observed variables with substantive cross-loadings (0.0%, 12.5%, and 25.0%); and (7) sample size (300, 500, and 1000). Performance was evaluated through the Omega index, Mean Bias Error (MBE), Mean Absolute Error (MAE), sensitivity, specificity, and mean number of isolated nodes. We also evaluated two other methods, Exploratory Factor Analysis and the Walktrap algorithm modified to consider overlap (EFA-Ov and Walk-Ov, respectively). The Walk-Ov displayed the best performance across most conditions and is the recommended option to identify communities with overlapping symptoms in psychological networks.

**Supplementary Information:**

The online version contains supplementary material available at 10.3758/s13428-024-02415-2.

## Introduction 

In the last decade, a new theoretical framework, named the *network theory of psychological processes* (or network psychometrics) (Robinaugh et al., 2020) has been increasingly used to conceptualize, model, and explain psychological data (Borsboom et al., 2021). Contrasting to the *latent trait theory* (which postulates that unobservable psychological processes are the “common cause” of observable psychological processes), the *network theory of psychological processes* postulates that observable psychological processes such as behaviors, cognitions, and emotions mutually causally reinforce each other. For example, difficulty sleeping causes fatigue, fatigue causes loss of interest in pleasurable activities, loss of interest in pleasure activities causes sad mood, sad mood causes difficulty sleeping, and so forth. This network of mutually causally reinforcing behaviors, cognitions, and emotions can then be referred to by the *verbal label* of “depression” (Schmittmann et al., 2013). Due to a paradigm shift in interpreting psychological data, the field of network psychometrics brought new insights into several psychological processes (including psychopathologies, personality, and attitudes) such as depression (Beard et al., 2016; Wichers et al., 2021), post-traumatic stress disorder (McNally et al., 2017) and substance abuse (Rhemtulla et al., 2016), among many others (Robinaugh et al., 2020).

Based on the network theory of psychological processes, researchers started investigating statistical models to estimate psychological networks from the data. The most commonly used models are *graphical models*, such as the Ising model (Van Borkulo et al., 2014) for binary data and the Gaussian Graphical Model (Epskamp & Fried, 2018) for continuous data, which describe as a network the structure of *conditional dependence* between variables. In these models, variables are represented by nodes and the conditional associations between variables are represented by edges. The choice of the measure of conditional association depends on the nature of the data. For instance, in multivariate normal data, partial correlations are employed (Borsboom et al., 2021). Psychological networks are *weighted* (the *edge weight* is the size of the partial correlation) and *signed* (since partial correlations can be positive or negative). Furthermore, statistical measures and procedures originally developed for network sciences, such as centrality measures (Freeman, 1977) and community detection algorithms (Golino & Epskamp, 2017), were also adopted to describe the features of psychological networks.

Among these, the detection of network communities (Golino & Epskamp, 2017) became a central feature in network psychometrics (Golino et al., 2020). In psychological networks, the specific interpretation of a network community is that it indicates behaviors, cognitions, and emotions that establish strong mutually reinforcing causal effects among themselves compared to other behaviors, cognitions, and emotions in the psychological network (Christensen et al., 2020; Jones et al., 2021). Network communities in psychological networks can represent distinct psychological processes, such as psychopathologies (e.g., depression, anxiety) (Borsboom & Cramer, 2013), and personality traits (e.g., agreeableness, conscientiousness) (Cramer et al., 2012), among others. This theoretical interpretation of communities in psychological networks was coupled with methodological advances demonstrating that network models are statistically consistent^1^ with factor models when the data are generated from a factor model (but factor models are not statistically consistent with network models when the data are generated from a network model) (Christensen & Golino, 2021; Kruis & Maris, 2016; van Bork et al., 2021). Notably, the *latent* *factor* (from a factor model) and the *network community* (from a network model) are consistent (see Golino & Epskamp (2017) for a mathematical demonstration of their consistency). Due to this statistical consistency, both the number of network communities (in network models) and latent factors (in factor models) indicate the “dimensionality” of psychological data (Jimenez et al., 2022). Consequently, community detection algorithms (originally developed for network models) can also be used to identify dimensionality in data structures generated from a factor model (in addition to traditional eigenvalue-based procedures such as Kaiser–Guttman eigenvalue greater than 1 rule, Scree test, Parallel Analysis, among others) (Christensen et al., 2020).

### The Walktrap algorithm

In network science, several network community detection algorithms have been developed, such as the Walktrap algorithm (Pons & Latapy, 2005), the Spinglass algorithm (Reichardt & Bornholdt, 2006), and the Louvain algorithm (Blondel et al., 2008), among many others. In psychological networks, Christensen and Golino (2020) performed a simulation study to evaluate the performance of several community detection algorithms to identify dimensionality in data structures generated from factor models, indicating that the Walktrap and Louvain algorithms achieved the best performance.

The Walktrap algorithm has been the most commonly applied community detection algorithm in network psychometric research. A detailed explanation of the Walktrap algorithm is provided in Appendix A (Supplementary Material). In summary, the Walktrap algorithm uses random walks to identify clusters of nodes (i.e., communities) in a network and then employs Ward’s agglomerative clustering approach, initially considering each node as its own community and progressively combining nodes with small distances into larger clusters, ending up in a single community containing all nodes. However, this agglomerative clustering approach cannot indicate the number of communities (between one community per node and one community containing all nodes) that is the most suitable to describe the network. To determine the number of communities, *metrics* were developed to evaluate the “goodness” of a community structure, i.e., the quality of the partitioning of nodes into a community structure (Chakraborty et al., 2017, p. 54). The most prominent metric for determining the goodness of a community structure is *modularity* (Fan et al., 2007; Newman & Girvan, 2004). Higher values of modularity indicate that nodes belonging to a network community have more connections within the community and fewer connections outside of the community (Jamison et al., 2021).

Formally, modularity for weighted networks is defined as:

1 $$Q=\frac{1}{2w}\sum\limits_{g\in G}\sum\limits_{i,j\in V}({w}_{ij}-\frac{{w}_{i}{w}_{j}}{2w})({\delta }_{gi}{\delta }_{gj})$$

where V is the set of vertices (i.e., nodes), G is the set of communities corresponding to a partition, ${w}_{ij}$ is the edge weight between observed variables *i* and* j*, ${w}_{i}$ is the sum of all item *i* edge weights, and $w$ is the sum of all edge weights in the network. Each node belongs only to a single community so ${\delta }_{gi}$ has a value of 1 if node *i* belongs to community *g* and 0 otherwise, and $({\delta }_{gi}*{\delta }_{gj})$ is the Kronecker delta function which has a value of 1 when nodes *i* and *j* belong to the same community *g* and 0 otherwise. The Kronecker delta function $({\delta }_{gi}*{\delta }_{gj})$ ensures that edge weights are only considered in the calculation of modularity when nodes belong to the same community. Certain community detection algorithms (such as the Louvain and fast greedy algorithms) will use heuristics seeking to directly maximize modularity *Q*, referred to as “modularity-optimization” (although many community detection algorithms cannot guarantee that modularity is maximized; Brusco et al., 2022b). In contrast, in the Walktrap algorithm, the standard practice is to select, among the community configurations identified during the agglomerative clustering approach, the community structure that achieved the highest modularity *Q* (Brusco et al., 2022a; Christensen & Golino, 2020). This is one potential advantage of the Walktrap algorithm compared to other community detection algorithms (such as the Louvain algorithm) since the Walktrap does not seek to maximize modularity directly in the algorithmic process and only uses modularity post hoc to determine the number of communities (this can be beneficial under certain circumstances such as unequally sized communities in which modularity is a potentially "less viable" measure; Brusco et al., 2022b, p. 3562).

The use of the Walktrap algorithm (or other high-performing algorithms) to identify communities in psychological networks is usually referred to by the term Exploratory Graph Analysis (EGA). Despite the excellent performance of the Walktrap algorithm and its widespread use in psychological research (Christensen & Golino, 2020), one limitation of the Walktrap algorithm (and other algorithms evaluated in simulation studies; Christensen et al., 2023) is that it can only assign each node to a single community (instead of assigning a node to two or more communities). Community detection algorithms that can only assign each node to a single community are named *non-overlapping* *community detection* *algorithms* since they cannot identify overlapping nodes (i.e., nodes belonging to more than one community). The availability of community detection algorithms that can assign a node to multiple communities is especially relevant in psychological networks due to *overlapping* *symptoms* (Blanken et al., 2018).

### Overlapping symptoms

In network science, overlapping nodes refer to nodes that belong to more than one community (Lancichinetti & Fortunato, 2009). In psychological networks, overlapping symptoms refer to cognitions, behaviors, and emotions that belong to more than one community in a psychological network (Blanken et al., 2018). For example, posttraumatic stress disorder symptoms overlap considerably with symptoms of depression, so certain symptoms are part of both the posttraumatic stress disorder community and the depression community (Jones et al., 2021). Overlapping symptoms are fundamental to explaining the comorbidity observed between mental disorders, such as the comorbidity between major depressive disorder and generalized anxiety disorder since both disorders contain symptoms that “are connected with one (mental disorder or) another, and comorbidity arises only through connections between overlapping symptoms” (Cramer et al., 2010, p. 140).

In the emerging field of network psychometrics, while the terms *bridging* and *overlapping* symptoms were initially used interchangeably (Afzali et al., 2017), Jones et al. (2021) established a theoretical distinction between *bridging symptoms* and *overlapping symptoms*. Bridging symptoms refers to symptoms (nodes) that belong to only one community but are conditionally associated with (nodes from) another community (i.e., another psychological process) and constitute a “bridge” between the communities. Overlapping symptoms refers to symptoms (nodes) that belong to more than one community and are strongly conditionally associated with (nodes from) both communities (i.e., both psychological processes).

**Fig. 1:**
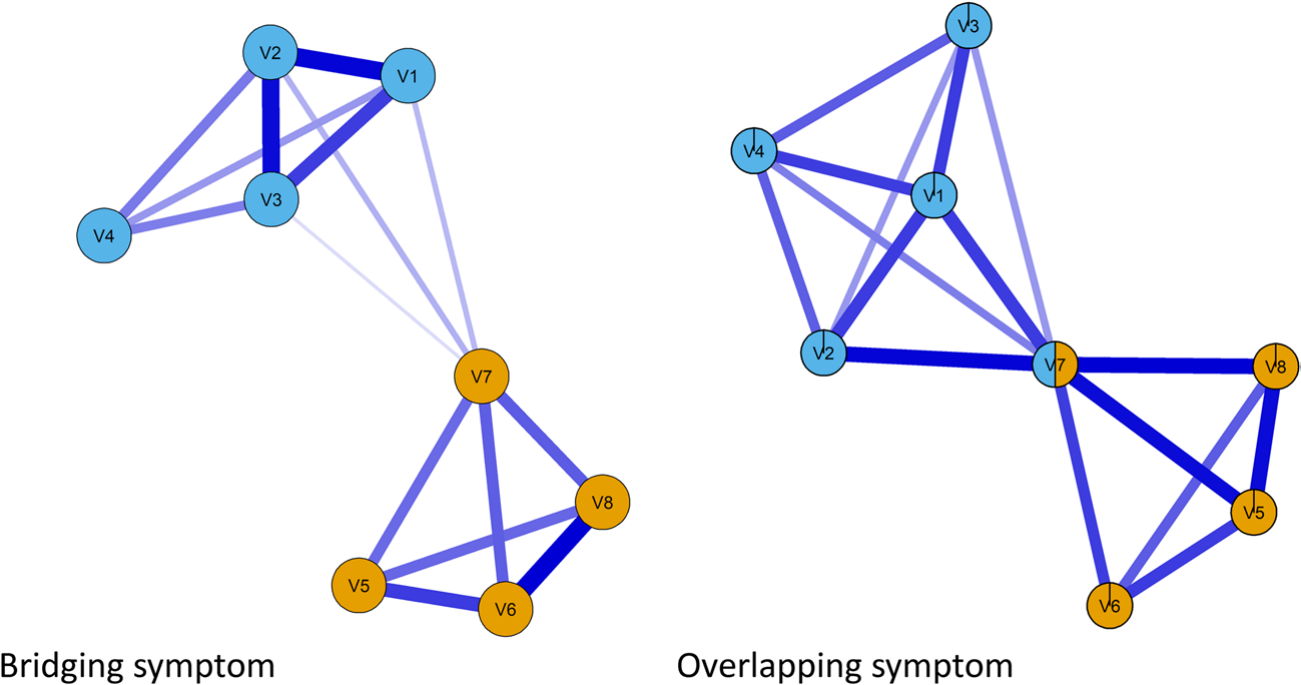
Bridging and overlapping symptoms. In the *left panel*, Item 7 constitutes a bridging symptom. In the *right panel*, Item 7 constitutes an overlapping symptom

In both cases, the bridging or overlapping symptoms establish conditional associations with several nodes from another community, so both bridging and overlapping symptoms display *network cross-loadings* (i.e., *network loadings* to more than one community). Network loadings are defined as the standardized sum of each node’s connection with nodes from a particular community in the network (Christensen & Golino, 2021). Network cross-loadings are also statistically consistent with factor cross-loadings when the data-generating mechanism is a factor model (Christensen & Golino, 2021). One proposition to empirically distinguish bridging and overlapping symptoms is according to the *strength of the network cross-loadings*. As the symptom gets more strongly connected with another community (i.e., stronger network cross-loadings), it goes from belonging to one community and bridging the symptoms to another community (Fig. 1, left panel) to belonging to both communities (Fig. 1, right panel). A visual representation showing this proposed empirical distinction between bridging and overlapping symptoms is displayed in Fig. 1.

Despite recent methodological advances such as the development of *bridge centrality measures* to identify bridge symptoms in psychological networks (Jones et al., 2021), there have been no methods validated by simulation studies as capable of identifying overlapping symptoms in psychological networks. In the network science literature, several *overlapping* *community detection* *algorithms* have been proposed such as the InfoMap (Xie et al., 2013) and ModuLand (Kovács et al., 2010) algorithms. One community detection algorithm that can assign a node to multiple communities and has been argued as potentially useful for the analysis of psychological networks (Lange, 2021b) is the Clique Percolation (CP) algorithm (Palla et al., 2005).

**Fig. 2:**
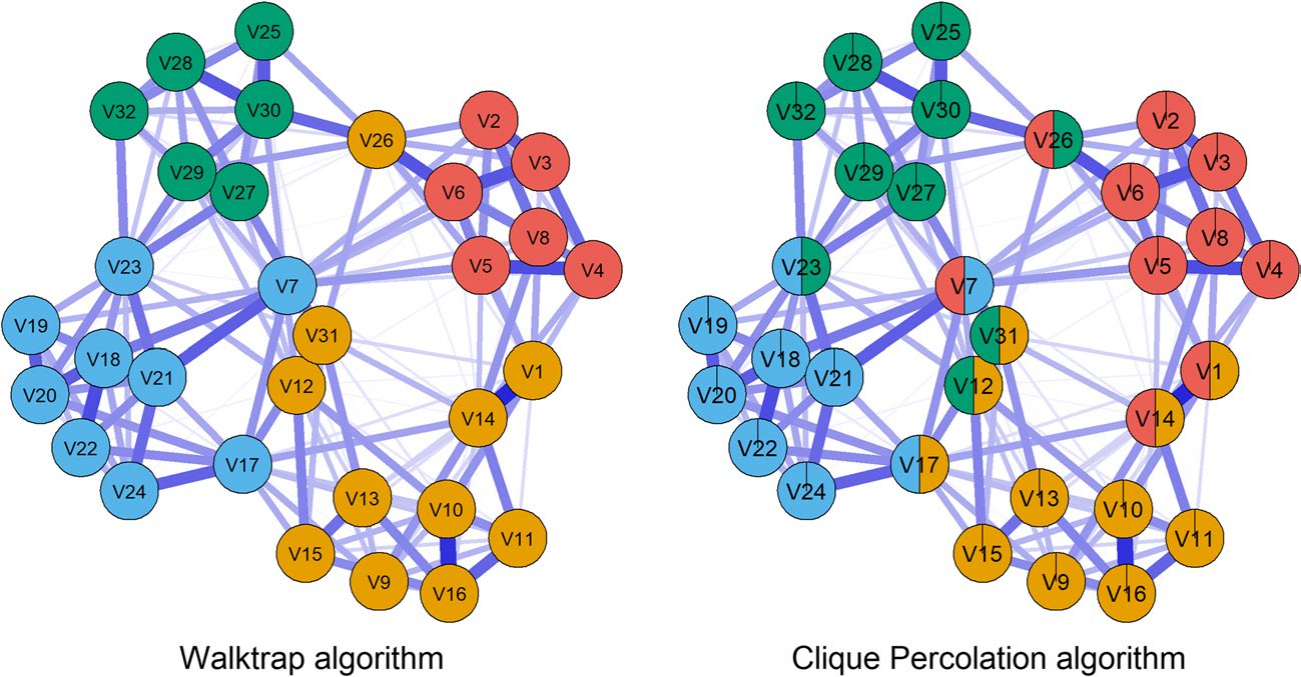
Node assignment comparison between overlapping and non-overlapping community detection algorithms in psychological networks with overlapping symptoms. The *left panel* indicates the psychological network with node assignment according to the Walktrap algorithm. The *right panel* indicates the psychological network with node assignment according to the CP algorithm. The overlapping symptoms were the eight variables: V1, V7, V12, V14, V17, V23, V26, and V31

To illustrate the distinction between non-overlapping and overlapping community detection algorithms, Fig. 2 provides an example of a simulated psychological network (for more details on simulating psychological network with overlap, please refer to the Methods section of this paper) and compares the Walktrap algorithm (a non-overlapping community detection algorithm) with the CP algorithm (an overlapping community detection algorithm). In this example, eight variables (V1, V7, V12, V14, V17, V23, V26, and V31) were simulated to be overlapping and they were correctly identified as overlapping by the CP algorithm (described in the next section).

### Clique Percolation algorithm

In a network, a *k-clique* is defined as the complete (fully connected) subgraphs of *k* nodes (e.g., a *k-clique *with three nodes is equivalent to a triangle). A *k-clique adjacency* occurs when two *k-cliques* share *k*–1 nodes, differing only by a single node. Within the CP framework, the network community is defined as the maximum set of *k*–1 adjacent cliques. Considering that one node can belong to more than one *k-clique*, the identification of network communities by the CP algorithm naturally allows nodes to be assigned to more than one community. For weighted networks (such as partial correlation networks), a *weighted* *k-clique* ${C}_{k}$ is a complete subgraph of *k* nodes when the geometric mean of the edge weights associated with the clique ${C}_{k}$:

2 $$I\left({C}_{k}\right)={(\prod\nolimits_{\left\{i,j\right\}\in {C}_{k}}{w}_{ij})}^{2/(k(k-1))}$$

is greater than an established intensity threshold *I* (Farkas et al., 2007). Since in the CP algorithm the absolute value of the edge weight is considered and negative edges are treated as positive edges, the intensity threshold *I* can only be positive (Lange, 2021b). For each value of *k*, while large values of the intensity threshold *I* lead to the exclusion of most *k-*cliques (so all nodes are considered isolated and no community is identified), smaller values of the intensity threshold *I* will increasingly include most to all *k-*cliques. In this case, the increasing addition of *k-*cliques by lowering the intensity threshold *I* below a critical point will eventually lead to a “giant” community being identified and the emergence of this “giant” community is analogous to a percolation transition (Farkas et al., 2007, p. 7). Furthermore, in one of the early software programs developed to run the CP algorithm (named CFinder), an additional new rule was implemented in the CP algorithm (Adamcsek et al., 2006). In this new rule, besides the geometric mean of the edge weights associated with the clique ${C}_{k}$ being required to be greater than the intensity threshold *I*, the edge weight of the shared edges between two adjacent *k-*cliques also needed to be greater than the intensity threshold *I* so the *k-*cliques were considered adjacent (Lange, 2021a). In summary, the number of communities identified by the CP algorithm is based on two parameters, *k* (the number of nodes in a clique) and *I* (the intensity threshold), and determining the number of communities becomes an issue of *model selection*.

For each *k*, Farkas et al. (2007) recommended that the optimal value of *I* is just above the critical point, allowing most of the *k*-cliques to participate in communities while prohibiting the emergence of a giant community. At the critical point, the size of the communities follows a power-law distribution, so the optimal value of *I* can be found by starting with the “highest meaningful value of *I*” and lowering it until the ratio of the two largest communities equals 2 (Farkas et al., 2007, p. 8). Besides the ratio of the two largest communities, another metric proposed to determine the clique size *k* and intensity threshold *I* is *entropy* (Lange, 2021b). The *entropy* is defined as:

3 $$Entropy= -\sum\limits_{g=1}^{N}{p}_{g}{{\text{log}}}_{2}{p}_{g}$$

where *N* is the number of communities and ${p}_{g}$ is the probability of being in community *g*. Lange (2021b, p. 3) recommended that the entropy of the community assignment should be maximized (to choose the optimal values of *k* and *I*) and discussed how the “entropy is maximal when the resulting communities are equally sized with a small number of isolated nodes”. Finally, a third metric proposed to determine the parameters *k* and *I* (and, consequently, the community assignment) is the variance of the community sizes after excluding the largest community, denoted as χ (Farkas et al., 2007).

### The CP algorithm and metrics used for model selection in psychological networks

In recent years, due to the CP algorithm being able to detect overlapping symptoms that belong to multiple communities, the CP algorithm has been used to investigate psychological networks of depression and anxiety (Blanken et al., 2018; Kaiser et al., 2021), semantic memory (Cosgrove et al., 2021), emotions (Lange & Zickfeld, 2021), cognitive ability (Ismatullina et al., 2022), aphasia (Ashaie & Castro, 2021), among others (Martarelli et al., 2021). However, the performance of the CP algorithm to identify nodes belonging to multiple communities in psychological data has not been evaluated by simulation studies. This evaluation is crucial since there are several concerns regarding the use of the CP algorithm as an overlapping community detection algorithm in psychological networks (Brusco et al., 2023). One of the most serious limitations is that the CP “does not look for actual communities, consistent with the shared notion of dense subgraphs, but for subgraphs ‘containing’ many cliques, which may be quite different objects than communities (for instance, they could be ‘chains’ of cliques with low internal edge density)” (Fortunato, 2010, p. 131). Moreover, other limitations of the CP algorithm include the community partitioning leaving many isolated nodes (often failing to assign a node to at least one community) and the *ad hoc* selection of the parameters clique size *k* and intensity threshold *I* based on a specific metric (Brusco et al., 2023). There are also concerns regarding the metrics that are currently the most used to determine the CP parameters clique size *k* and intensity threshold *I* in psychological networks, notably the ratio of the two largest communities (Farkas et al., 2007) and entropy (Lange, 2021b).

**Fig. 3:**
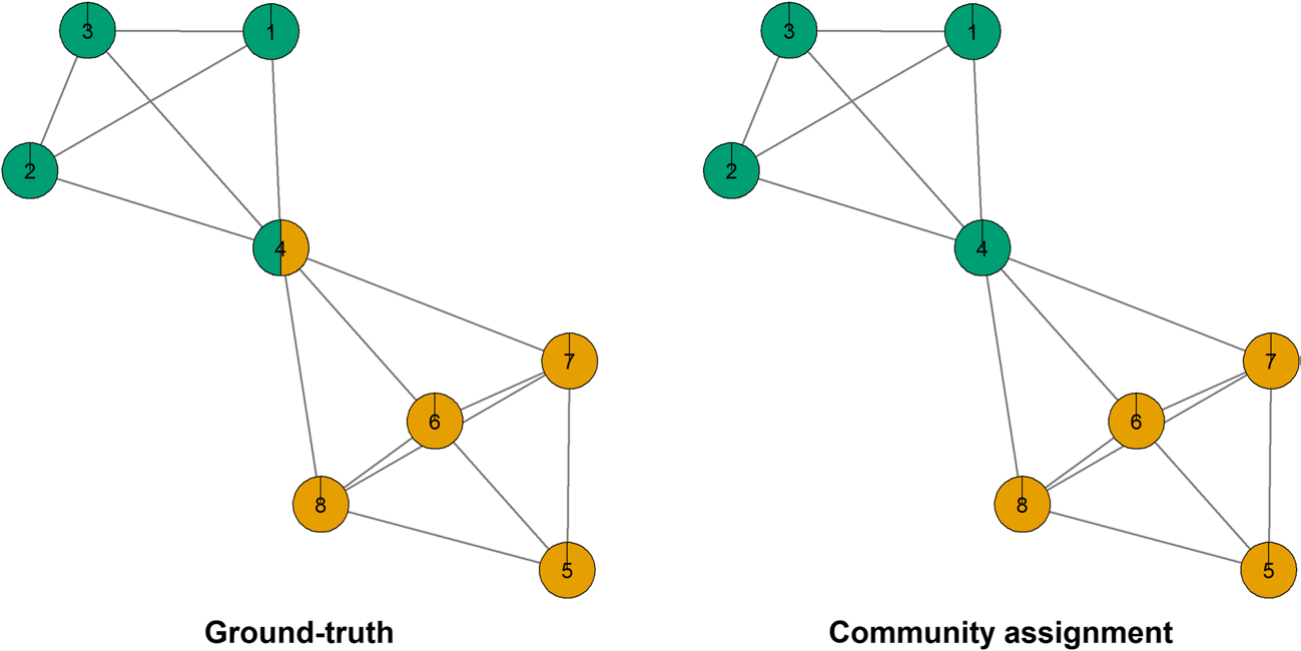
Entropy comparison between ground-truth network and an incorrect community assignment. The *left panel* indicates the “ground-truth” network, the correct node assignment to communities. The *right panel* indicates an example of incorrect node assignment to communities

Regarding the ratio of the two largest communities, Lange (2021a) discussed how the CP algorithm was originally developed to identify communities in large networks (sometimes containing hundreds or thousands of nodes), while psychological networks are very small networks, usually containing only up to 25 nodes. In the case of very small networks, the ratio between the largest communities is uninformative (Lange, 2021a). While entropy was proposed as an alternative (Lange, 2021a), one main concern is that using entropy as a metric can provide the wrong solution under specific circumstances. For instance, consider the example displayed in Fig. 3.

Figure 3 (left panel) indicates the “ground-truth” network (i.e., the correct community assignment indicating which nodes are overlapping and which nodes are not), which includes one community with three nodes, another community with four nodes and one node belonging to two communities (i.e., node 4). Figure 3 (right panel) indicates an incorrect community assignment, which includes two communities with four nodes and no overlapping node. Using Eq. (3)) to calculate the entropy, the entropy for the ground-truth network is 0.989 and the entropy for the incorrect community assignment is 1.000. Hence, if the maximal entropy is used as a criterion to evaluate community partitioning, the entropy would favor the incorrect community assignment (Fig. 3 right panel) instead of the ground-truth (Fig. 3 left panel). The reason is that, as Lange (2021b) discussed, entropy is maximal when the communities are *equally sized* with a small number of isolated nodes. In this case, the incorrect community assignment favored equally sized communities (four nodes in each community) instead of the ground-truth of unequally sized communities (one community was larger due to an overlapping node).

Furthermore, Lange (2021b) discussed how the χ is also unreliable for a small number of communities (as observed in psychological networks) and can only be computed when there are three or more communities. Since many scenarios of this simulation study included only two communities (also commonly observed in psychological networks; Santiago et al., 2022), the χ could not be computed and, for these reasons, was not considered in this simulation study.

Given these limitations, the performance of other metrics (besides the ratio of the two largest communities and entropy) must also be evaluated to inform model selection by the CP algorithm in psychological networks. One potential alternative discussed by Tóth et al. (2013) as a metric for the CP algorithm is the *fuzzy modularity* *for weighted networks*. The fuzzy modularity for weighted networks is a generalization of the modularity for weighted networks (Eq. 1). In the modularity for weighted networks (Eq. 1), a node *i* can only belong to one community and the indicator ${\delta }_{gi}$ has a value of 1 if node *i* belongs to community *g* and 0 otherwise. In the fuzzy modularity for weighted networks (Eq. 4), a node *i* can belong to more than one community and the term ${\delta }_{gi}$ is substituted by the “belonging coefficient” ${\alpha }_{gi}$ (Chen et al., 2010):

4 $${\alpha }_{gi}=\frac{\sum\nolimits_{k\ \in\ g}{w}_{ik}}{{\sum\nolimits }_{{G}^{^\prime}}{\sum\nolimits }_{k\ \in\ g}{w}_{ik}}$$

where $g$ is a network community and ${G}{\prime}$ is the set of network communities that node *i* is assigned to. The “belonging coefficient” ${\alpha }_{gi}$ has a value ranging from 0 to 1 that indicates the contribution of the node to each of the communities it belongs to. Similar to ${\delta }_{gi}$, the belonging coefficient” ${\alpha }_{gi}$ has a value of 0 for the communities the node has not been assigned to. More formally, the *fuzzy modularity for weighted networks* is defined as follows (Chen et al., 2010):

5 $${Q}_{ov}=\frac{1}{2w}\sum\limits_{g\ \in\ G}\sum\limits_{i,j\in\ V}({w}_{ij}-\frac{{w}_{i}{w}_{j}}{2w})(\frac{{\sum\nolimits }_{k\ \in\ g}{w}_{ik}}{{\sum\nolimits }_{{G}^{^\prime}}{\sum }_{k\ \in\ g}{w}_{ik}}\frac{{\sum\nolimits }_{k\ \in\ g}{w}_{jk}}{{\sum\nolimits }_{{G}^{^\prime}}{\sum\nolimits }_{k\ \in\ g}{w}_{jk}})$$

Considering that in the application of the CP algorithm to large networks, the optimal value of the intensity threshold *I* is just above the critical point (in which a giant community emerges) (Farkas et al., 2007), Tóth et al. (2013) evaluated whether the *fuzzy modularity for weighted networks* would also identify the correct community assignment by investigating whether the fuzzy modularity was maximized just above the critical point. Their simulation study provided evidence that “the overlapping modularities show a maximum close to the critical point” and that fuzzy modularity could also be used to inform the selection of the intensity threshold *I* and, consequently, identify the correct community assignment in large networks (Tóth et al., 2013, p. 1). While Tóth et al. (2013) evaluated the performance of the CP with fuzzy modularity in large networks (in which both the ratio of the two largest communities and fuzzy modularity can be used to identify the correct community structure), fuzzy modularity is a promising metric for psychological networks (which are very small networks) for two reasons: (1) it provides an alternative to the ratio of the two largest communities (Farkas et al., 2007) since this ratio is uninformative for very small networks (Lange, 2021a); and (2) modularity is a metric already used successfully by non-overlapping community detection algorithms to identify communities in psychological networks such as the Walktrap algorithm (Golino et al., 2020).

Furthermore, in psychological networks (such as partial correlation networks), one consideration for the calculation of modularity is that network edge weights are *signed* (there are positive and negative partial correlations). The formulation of the modularity for weighted networks (Fan et al., 2007) considers that the term $\frac{{w}_{i}}{2w}$ (see Eq. 1) can be interpreted as the probability that node *i* make connections to other nodes in a random network (analogous to the node degree used in the original formulation of modularity for unweighted networks; Newman & Girvan, 2004). However, it can easily be seen that this formulation loses its probabilistic interpretation when positive and negative edge weights are considered (since positive and negative edge weights cancel each other out) (Gómez et al., 2009). As such, Gómez et al. (2009) proposed *modularity for signed weighted networks*, generalized as *fuzzy modularity for signed weighted networks:*

6 $${Q}_{ovs}=\frac{2{w}^{+}}{2{w}^{+}+2{w}^{-}}(\frac{1}{2{w}^{+}}\sum\limits_{g\ \in\ G}\sum\limits_{i,j\ \in\ V}({w}_{ij}^{+}-\frac{{w}_{i}^{+}{w}_{j}^{+}}{2{w}^{+}})(\frac{{\sum\nolimits }_{k\ \in\ g}{w}_{ik}^{+}}{{\sum\nolimits }_{{G}^{^\prime}}{\sum\nolimits }_{k\ \in\ g}{w}_{ik}^{+}}\frac{{\sum\nolimits }_{k\ \in\ g}{w}_{jk}^{+}}{{\sum\nolimits }_{{G}^{^\prime}}{\sum\nolimits }_{k\ \in\ g}{w}_{jk}^{+}}))-\frac{2{w}^{-}}{2{w}^{+}+2{w}^{-}}(\frac{1}{2{w}^{-}}\sum\nolimits_{g\ \in\ G}\sum\nolimits_{i,j\ \in\ V}({w}_{ij}^{-}-\frac{{w}_{i}^{-}{w}_{j}^{-}}{2{w}^{-}})(\frac{{\sum\nolimits }_{k\ \in\ g}{w}_{ik}^{-}}{{\sum\nolimits }_{{G}^{^\prime}}{\sum\nolimits }_{k\ \in\ g}{w}_{ik}^{-}}\frac{{\sum\nolimits }_{k\ \in\ g}{w}_{jk}^{-}}{{\sum\nolimits }_{{G}^{^\prime}}{\sum\nolimits }_{k\ \in\ g}{w}_{jk}^{-}}))$$

where ${w}_{ik}^{+}$ is the positive edge weight between observed variables *i* and *j,* ${w}_{i}^{+}$ is the sum of all item *i* positive edge weights,${w}^{+}$ is the sum of all positive edge weights in the network, ${w}_{ik}^{-}$ is the negative edge weight between observed variables *i* and *j,* ${w}_{i}^{-}$ is the sum of all item *i* negative edge weights,${w}^{-}$ is the sum of all negative edge weights in the network. In cases of weighted networks that have only positive edge weights, the *fuzzy modularity for signed weighted networks* reduces to the *fuzzy modularity for weighted networks*. In cases of assignment of only one node per community (no overlap), the *fuzzy modularity for weighted networks* reduces to the *modularity for weighted networks*. Despite the CP algorithm considering only the absolute values of the edge weights, the *fuzzy modularity for signed weighted networks* is calculated based on the adjacency matrix and does consider the sign of the edge weights, so it can be used as a metric to inform (a posteriori) the quality of the community partitioning proposed by the CP algorithm (according to the choice of clique size *k* and intensity threshold *I*). As such, given that edges in psychological networks are signed, it is also important to investigate whether the *fuzzy modularity for signed weighted networks* is a better metric compared to *fuzzy modularity for weighted networks*.

Overall, for empirical researchers to know whether the CP algorithm can accurately identify communities and overlapping symptoms in *psychological networks*, it is fundamental that the performance of the CP algorithm (and the proposed model selection metrics) is evaluated in a simulation study. For this reason, the current study aims to evaluate the performance of the CP algorithm with *fuzzy modularity for weighted networks* (and *fuzzy modularity for signed weighted networks*) given previously discussed limitations of the ratio between the two largest communities (Farkas et al., 2007) and the entropy (Lange, 2021b) in psychological networks.

Another research gap that the current study aims to address is that there are, to the best of our knowledge, no benchmarks in terms of simulation strategies to evaluate the performance of *overlapping* community detection algorithms in psychological networks. There are clear benchmarks for simulation studies investigating the performance of *non-overlapping* community detection algorithms in psychological networks, originally developed by Golino and Epskamp (2017) and later employed, for example, by Christensen et al. (2023) to evaluate a multiplicity of non-overlapping community detection algorithms. However, these benchmarks are not yet available for overlapping community detection in psychological networks. Benchmarks have been developed for overlapping community detection in network science (Lancichinetti & Fortunato, 2009). However, the benchmarks developed by Lancichinetti and Fortunato (2009) were not developed considering the specificities of psychological networks. Firstly, they generate networks with node degree distribution based on power law, while in small (but also sparse) networks (such as psychological networks) the node degree distribution does not follow a power law (Dablander & Hinne, 2019). Secondly, psychological networks are partial correlation networks of multivariate data that can already be successfully simulated by generating data from factor models (Golino & Epskamp, 2017). The current study aims to propose the first benchmark for simulating psychological networks with overlapping symptoms.

### The current study

To simulate psychological networks with overlapping symptoms, it is necessary to establish first an *empirical criterion* for symptoms to be considered as overlapping symptoms in psychological networks. An empirical criterion of overlapping symptoms is required to define the “ground truth” network (i.e., the “true” network model indicating which symptoms are overlapping and which symptoms are not) and this “ground truth” network will then be compared with the community assignment from overlapping community detection algorithms to evaluate the algorithm accuracy. We followed Lancichinetti and Fortunato (2009, p. 2) recommendations that, when specifying for an overlapping node how edges (and edge weights for weighted networks) should be distributed among the communities the node belongs to, “one can follow several recipes; we chose the simple equipartition”. That is, the node has an equal number of edges across two communities in unweighted networks and an equal *number* and *weights* of edges across two communities in weighted networks. There are also theoretical reasons for considering nodes with equal edge weights across communities as overlapping nodes since it is “undecidable” which community the node should be assigned to (Reichardt & Bornholdt, 2006, p. 7). Consequently, we considered an overlapping symptom in a psychological network as a symptom that *has substantive and equally sized* *network loadings to more than one community*. As such, the overlapping node establishes equivalent conditional associations with two or more communities in psychological networks.

Given this definition, to investigate the performance of the CP algorithm in psychological networks, we will simulate psychological data from multiple latent factors (i.e., equivalent to network communities) and include observed variables with substantive and equally sized cross-loadings (i.e., equivalent to substantive and equally sized network cross-loadings). We will first evaluate the performance of the CP algorithm to: (1) identify the correct number of latent factors (i.e., correct number of communities); and (2) identify overlapping symptoms (i.e., nodes with substantive and equally sized cross-loadings across more than one community).

Based on the recommendations from the simulation study, we will investigate the performance of the CP algorithm in an empirical dataset. We will conduct a re-analysis of Santiago et al. (2021) to evaluate the dimensionality of the Strengths and Difficulties Questionnaire (SDQ) (Goodman, 1997), the world’s most widely used psychological instrument to evaluate child well-being, among Australian children aged 4 to 10 years. Finally, we will provide recommendations for empirical researchers for the use of the CP algorithm in psychological data.

## Methods

### Simulation design

To investigate the CP algorithm performance under different types of psychological networks, we conducted a large simulation study and manipulated seven variables using Monte Carlo methods: data categories; number of factors; number of variables per factor; factor correlations; size of primary factor loadings; proportion of observed variables with substantive (and equally sized) cross-loadings (i.e., overlapping symptoms); and sample size.

In the simulation, the data categories evaluated were continuous, polytomous (five-point Likert scale) and dichotomous. The data simulation strategy for continuous, polytomous, and dichotomous variables is described in Appendix B (Supplementary Material). The simulation study investigated two and four factors, with four or eight variables per factor. Factor correlations were 0.0 (orthogonal), 0.5 (medium), and 0.7 (large) correlations. The primary factor loadings were low (0.40), medium (0.55), and high (0.70). There is evidence that the size of factor loadings has the largest and most substantial effect on community detection in psychological networks (Golino et al., 2020). Furthermore, considering that, in weighted networks, the CP algorithm establishes a *k*-clique according to the clique intensity (the geometric mean of the *edge weights*) (Brusco et al., 2023), the size of the factor loadings (which will influence the size of the edge weights with nodes from the same community) is likely to affect community detection by the CP algorithm. The proportions of observed variables with substantive cross-loadings were 0.0%, 12.5%, and 25.0%. The proportion of observed variables with substantive cross-loadings between 0.0% and 25.0% was chosen based on previous simulation studies that investigated the effect of cross-loadings on factor analytical dimensionality procedures (e.g., Parallel Analysis, empirical Kaiser criterion) (Li et al., 2020). For example, in a factor model with four factors and eight variables per factor with a proportion of 12.5% of observed variables with substantive cross-loadings, the number of observed variables with substantive cross-loadings was four.

One cautionary note is that in cases where there were three observed variables with substantive cross-loadings between two factors, these three observed variables become strongly conditionally associated among themselves and these conditional associations are stronger than the conditional associations they established with the two factors. This is conceptually problematic since the definition of a network community is nodes that are more strongly connected among themselves compared to other nodes in the network (Radicchi et al., 2004). Based on this definition, these three observed variables would constitute a separate community rather than overlapping symptoms between two factors. As such, the simulation restricted a maximum of two overlapping symptoms between two factors (i.e., sparse overlap between communities; Yang & Leskovec, 2013). For this reason, in the specific simulation scenario with two factors, eight variables and 25% of the observed variables with substantive cross-loadings, the number of variables with cross-loadings was reduced to two (instead of four) and simulation findings are equal to those with 12.5% of the observed variables with substantive cross-loadings.

Furthermore, the size of the substantive cross-loadings across two factors was bounded according to $\sqrt{\frac{0.9}{2+(2*\phi )}}$ and rounded down so the observed variables' communalities did not exceed 0.90 (e.g., for highly correlated factors ($\phi$ =0.7), the maximum substantive and equally sized cross-loading so communalities did not exceed 0.90 was 0.50)^2^. We also established that the sizes of substantive (and equally sized) cross-loadings were smaller than the primary factor loadings. As such, when the primary factor loadings were 0.70, 0.55, and 0.40, the substantive cross-loadings were 0.50, 0.40, and 0.30, respectively^3^. In addition to overlapping symptoms, considering that in psychological networks observed variables usually have small non-substantive cross-loadings, we included non-substantive cross-loadings (“noise”) randomly drawn from a normal distribution $\aleph (\mathrm{0.00,0.05})$ from an observed variable to all other factors (Golino et al., 2020). Finally, we also evaluated models with no substantive cross-loadings (0.0% proportion of substantive cross-loadings) so the performance of the CP algorithm could be investigated in conditions without overlapping symptoms.

In summary, the simulation strategy examined 972 conditions (3x2x2x3x3x3x3): (1) data categories (continuous, polytomous, and dichotomous); (2) number of factors (two and four); (3) number of observed variables per factor (four and eight); (4) factor correlations (0.0, 0.5, and 0.7); (5) size of primary factor loadings (0.40, 0.55, and 0.70); (6) proportion of observed variables with substantive cross-loadings (0.0%, 12.5%, and 25.0%); and (7) sample size (300, 500, and 1000). Our study investigated the 972 conditions across 100 simulated samples, resulting in a total of 97,200 datasets.

### Network estimation and community detection 

Considering that variables were continuous, the network model employed was the Gaussian graphical model (GGM) estimated with the Graphical Least Absolute Shrinkage and Selector Operator (GLASSO) based on the minimization of the extended Bayesian information criterion (EBIC) (Epskamp & Fried, 2018). Although other estimation methods are available (and may even be considered preferable) (Waldorp & Haslbeck, 2022; Williams & Rast, 2020), we employed the GLASSO since it became the most commonly used method by empirical researchers and it was the estimation method used in simulation studies evaluating the performance of non-overlapping community detection algorithms (e.g., Walktrap, Louvain) in psychological networks (Christensen et al., 2023; Golino & Epskamp, 2017; Golino et al., 2020). Notably, since distinct estimation procedures produce different weighted adjacency matrices (i.e., the presence and size of edges in a network) (Isvoranu & Epskamp, 2021), the community assignment by the CP algorithm (and other clustering algorithms) is contingent upon the estimation procedure (Brusco et al., 2022a) and might differ in case a different estimation procedure is used (non-regularized GGMs; Williams & Rast, 2020).

#### The CP algorithm

For community detection, the CP algorithm for weighted networks was implemented according to its use in the software CFinder (Adamcsek et al., 2006). For the CP algorithm, we followed recommendations from Farkas et al. (2007, p. 8) to establish the range of the intensity threshold *I* based on the “highest meaningful value of *I*”, so the intensity threshold *I* ranged from the maximum absolute value of the edge weights to 0.00. The size of the clique *k* ranged from 3 to 8. The selection of the CP algorithm parameters *k* and *I* were based on four criteria: (1) the maximization of the fuzzy modularity for signed weighted networks (CPSigMod); (2) the maximization of the fuzzy modularity for weighted networks (CPMod); (3) the minimization of the ratio between the two largest communities when the ratio is above or equal to 2 (CPRat) (Farkas et al., 2007); and (4) the maximization of the entropy (CPEnt) (Lange, 2021b). Together with the other metrics (CPRat and CPEnt), the CPMod and CPSigMod were recently implemented in the R package CliquePercolation (current version 0.4.0; Lange, 2021a). We hypothesize that the CP algorithm with parameter selection based on the maximization of the fuzzy modularity for weighted networks (CPMod) and maximization of the fuzzy modularity for signed weighted networks (CPSigMod) will have the best performance for networks with overlapping symptoms, given the limitations of the ratio between the two largest communities (CPRat) (Farkas et al., 2007) and the entropy (CPEnt) (Lange, 2021b) in very small networks (e.g., psychological networks).

#### Comparing the CP algorithm with established dimensionality assessment methods

Considering that the performance of the CP to identify the number of dimensions and overlapping symptoms is investigated in psychological networks (instead of other types of networks, such as social networks or gene networks), as a complementary research question, we also evaluate the CP performance compared to gold-standard methods used to investigate dimensionality in psychological data (Christensen et al., 2020). Firstly, following Golino et al. (2020) and Jimenez et al. (2022), we implemented: (5) Parallel Analysis (Horn, 1965) with Principal Component Analysis (PA_PCA_) using the generalized weighted least squares (GLS) factoring method to identify the number of dimensions. PA_PCA_ obtains eigenvalues using Principal Component Analysis (PCA) and compares the sample eigenvalues to the mean of eigenvalues from data simulated under a null model (i.e., uncorrelated normally distributed random variables). The number of sample eigenvalues that are larger than the reference eigenvalues obtained from the simulated data indicates the number of meaningful dimensions (Horn, 1965). Once the PA_PCA_ identified the number of dimensions, we implemented Exploratory Factor Analysis (EFA) with oblimin rotation to identify the factor loadings. Each variable was assigned to a factor according to the variable highest factor loading. According to conventional guidelines, absolute factor loadings higher than 0.4 are considered substantial (Comrey & Lee, 2013) so we considered as overlapping symptoms the observed variables that displayed substantive cross-loadings (i.e., absolute factor loadings higher or equal to 0.40) across more than one factor. We refer to this new procedure (of determining overlapping symptoms based on the items with substantive factor loadings across more than one factor) as EFA with overlapping symptoms (EFA-Ov).

Similarly, an important methodological question in network psychometrics is whether, instead of using overlapping community detection algorithms, it would be simpler to use a heuristic based on the size of network cross-loadings identified through non-overlapping community detection algorithms (Jimenez et al., 2022). Given that a network loading of 0.15 is equivalent to a factor loading of 0.40 (and, consequently, a network loading of 0.15 is considered substantial) (Christensen & Golino, 2021), we also implemented: (6) the Walktrap algorithm to identify the number of communities and then considered overlapping symptoms as those who displayed substantive cross-loadings (i.e., absolute network loadings higher or equal to 0.15) across more than one community. We refer to this new procedure (of determining overlapping symptoms based on the items with substantive network loadings across more than one community) as Walktrap with overlapping symptoms (Walk-Ov). In this simulation study, we used the default implementation of the Walktrap algorithm (i.e., function *EGA*) in the EGAnet R package version 1.2.3 so unidimensionality was evaluated with the Louvain algorithm.

### Statistical analysis

To evaluate the performance of the CP algorithm (and the EFA-Ov and Walk-Ov algorithms), we employed the Omega index (Collins & Dent, 1988) defined as:

7 $$\omega \left({A}_{1},{A}_{2}\right)= \frac{{\omega }_{u}\left({A}_{1},{A}_{2}\right)-{\omega }_{e}\left({A}_{1},{A}_{2}\right) }{1- {\omega }_{e}\left({A}_{1},{A}_{2}\right)}$$

where ${\omega }_{u}\left({A}_{1},{A}_{2}\right)$ indicates the *observed* agreement between community partitioning:

8 $${\omega }_{u}\left({A}_{1},{A}_{2}\right)= \frac{1}{M}\sum\limits_{j=0}^{{\text{max}}({K}_{1},{K}_{2})}\left|{t}_{j}({A}_{1})\bigcap {t}_{j}({A}_{2})\right|$$

and ${K}_{1}$ and ${K}_{2}$ are the number of communities in the community assignments ${A}_{1}$ and ${A}_{2}$, M indicates the number of node pairs and ${t}_{j}({A}_{1})$ is the number of times *j* that a set of node pairs appears together in community assignment ${A}_{1}$. For example, in a graph V= {A,B,C}, consider community assignment ${A}_{1}$ where community 1 is {A,B} and community 2 is {A,B,C}, so the node pair A-B appears 2 times together (in communities 1 and 2) and ${t}_{2}\left({A}_{1}\right)=1$ (for a step-by-step explanation of the Omega index calculation, please refer to Collins & Dent (1988)). Furthermore, ${\omega }_{e}\left({A}_{1},{A}_{2}\right)$ indicates the *expected* agreement between community partitioning under a null model:

9 $${\omega }_{u}\left({A}_{1},{A}_{2}\right)= \frac{1}{{M}^{2}}\sum\limits_{j=0}^{{\text{max}}({K}_{1},{K}_{2})}\left|{t}_{j}\left({A}_{1}\right)*{t}_{j}\left({A}_{2}\right)\right|$$

The Omega index can be used to quantify the agreement of the community assignment by the algorithm with the “ground-truth" (with values ranging from (– 1, 1] so negative values indicate worse agreement compared to what would be expected by chance, values near 0 indicate chance agreement and the value of 1 indicates perfect agreement) (Brusco et al., 2023; Canu et al., 2016). The Omega index is a generalization of the frequently employed Adjusted Rand Index (ARI) (Hubert & Arabie, 1985) since the Omega index can account for nodes being assigned to more than one community. In cases where nodes are only assigned to one community, the Omega index reduces to and becomes equal to the ARI (Xie et al., 2013). We employed the Omega index to evaluate community assignment, in addition to measures such as the Mean Absolute Error (MAE) and Mean Bias Error (MBE) calculated based on the identified *number* of factors, since the community detection algorithm might identify the correct *number* of factors but might still *incorrectly* assign nodes to communities (Christensen et al., 2023).

We also calculated the *sensitivity* and *specificity* of the algorithms (e.g., CPMod, Walk-Ov) to identify overlapping symptoms. The sensitivity is defined as the number of “true” overlapping symptoms *identified by the algorithm* over the number of “true” overlapping symptoms in the network. The specificity is defined as the number of “true” non-overlapping symptoms *identified by the algorithm* over the number of “true” non-overlapping symptoms in the network. Since one criticism against the CP algorithm is that it can leave “isolated” nodes (Brusco et al., 2023), we also calculated the mean number of isolated nodes. The sensitivity and specificity were only calculated in conditions with the presence of substantive cross-loadings.

Finally, we conducted analyses of variance (ANOVA) to investigate how the manipulated variables (data categories; number of factors; number of variables per factor; factor correlations; size of primary factor loadings; proportion of observed variables with cross-loadings; and sample size) affected the performance of the algorithms (e.g., CPMod, EFA-Ov) to correctly assign nodes into communities. In the ANOVA, the Omega index was considered the dependent variable and the manipulated variables were the independent variables (Christensen et al., 2023). We report the partial eta squared (${\eta }^{2}$) to inform effect sizes. Partial eta squares values of 0.01, 0.06, and 0.14 are deemed small, medium, and large effect sizes, respectively (Cohen, 1988). We report medium and large effect sizes. The simulation findings (and illustration of case-by-case examples) are displayed in an R-shiny app available at https://pedroribeirosantiago.shinyapps.io/cliquepercolation/.

## Results

All simulation findings are displayed in Supplementary Tables 1 and 2. The main findings of the simulation study are presented below.

### Correct node assignment (by sample size)

**Fig. 4:**
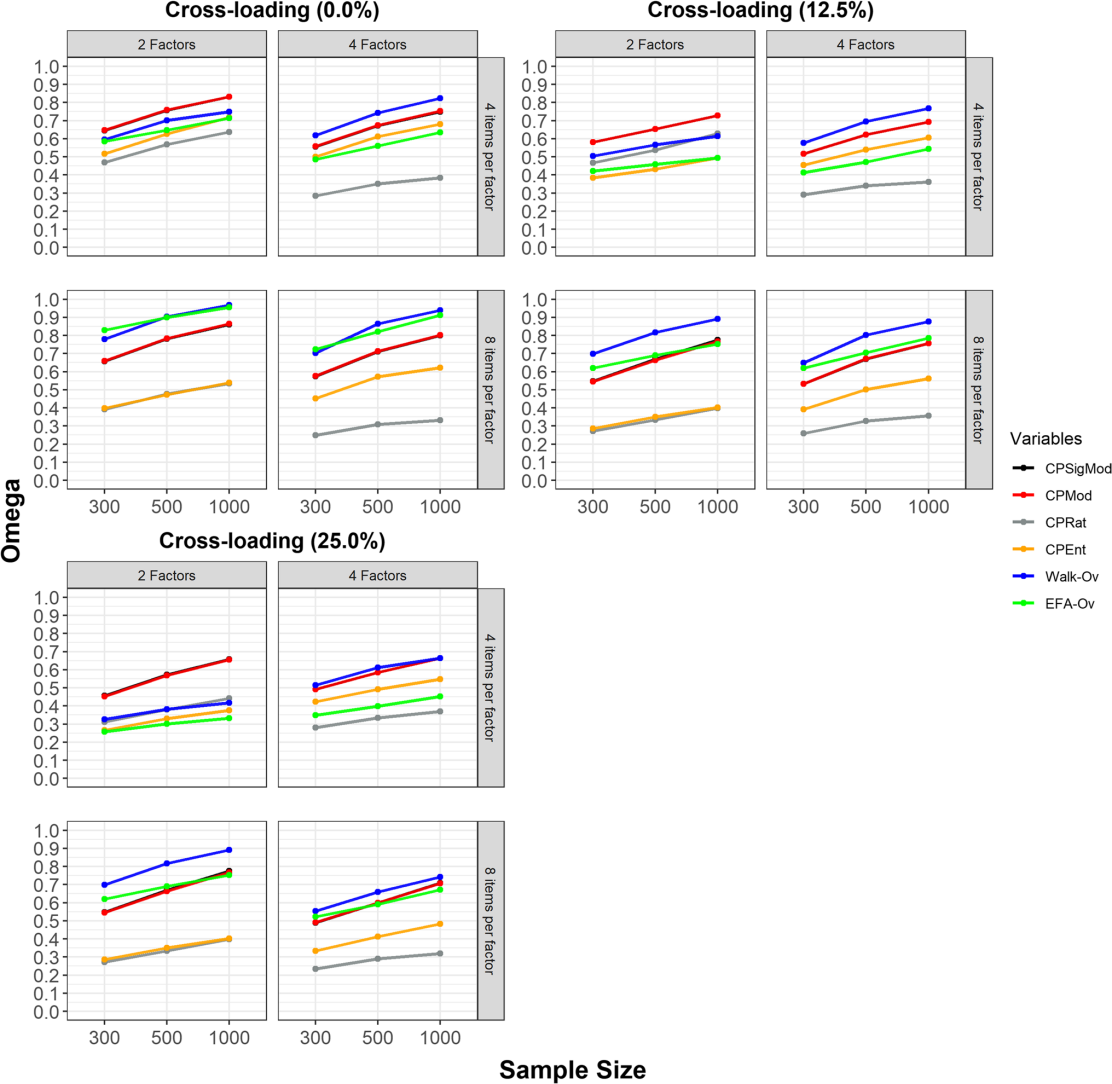
Correct node assignment according to sample size. CPSigMod = CP algorithm with maximization of fuzzy modularity for signed weighted networks; CPMod = CP algorithm with maximization of fuzzy modularity for weighted networks; CPRat = CP algorithm with minimization of the ratio between the two largest communities when the ratio is above or equal to 2; CPEnt = CP algorithm with maximization of entropy; Walk-Ov = Walktrap algorithm with overlapping nodes identified through network loadings > = |.15|; EFA-Ov = EFA-Ov = Exploratory Factor Analysis with overlapping nodes identified through factor loadings > = |.40|. The *x*-axis indicates sample size. The *y*-axis indicates the Omega index. Higher values of the Omega index indicate more accurate node assignment to communities

Considering all data categories (i.e., findings are not stratified according to data category), we present the correct node assignment as indicated by the Omega index according to sample size for conditions with the proportions of 0.0%, 12.5%, and 25.0% of observed variables with cross-loadings in Fig. 4.

Figure 4 indicates that the performances of the CPMod and CPSigMod were mostly indistinguishable and the CPSigMod did not provide additional accuracy in community assignment. For this reason, from now on, we will refer only to the CPMod (since this is a more parsimonious metric but the findings also apply to the CPSigMod). No method was superior across all conditions. When only the CP is considered, the CPMod displayed superior performance to the CPRat and CPEnt across all conditions. Furthermore, the CPMod also displayed a lower number of isolated nodes (a well-known limitation of the CP algorithm) compared to the CPRat and CPEnt across most conditions (Supplementary Figure 3). When all algorithms are considered, the Walk-Ov displayed superior or equivalent performance to the CPMod (and the other algorithms) across most conditions. The exceptions were conditions with two factors and four variables per factor, in which the CPMod displayed superior performance. Furthermore, the performance of all algorithms increased with larger samples.

The ANOVA results are displayed in Supplementary Table 3. The two large effect sizes identified in the ANOVA were the size of primary factor loadings (${\eta }_{p}^{2}=0.2616$) and the factor correlation (${\eta }_{p}^{2}=0.1477$). The two medium effect sizes identified in the ANOVA were the proportion of substantive cross-loadings (${\eta }_{p}^{2}=0.0697$) and the sample size (${\eta }_{p}^{2}=0.0559$). For this reason, in addition to the stratification by sample size (presented above), we also present, in the section below, the findings stratified according to the size of primary factor loadings and factor correlations (due to this study research question, all findings are already presented in plots stratified according to the proportion of substantive cross-loadings). Finally, despite the impact of data categories being small (${\eta }_{p}^{2}=0.0003$), we also present for completeness all findings stratified according to data categories in Supplementary Figures 16 to 12. Overall, the performance of all algorithms was better for continuous variables compared to ordinal variables, and better for ordinal variables compared to dichotomous variables (Supplementary Figure 16).

### Correct node assignment (by size of primary factor loadings)

**Fig. 5:**
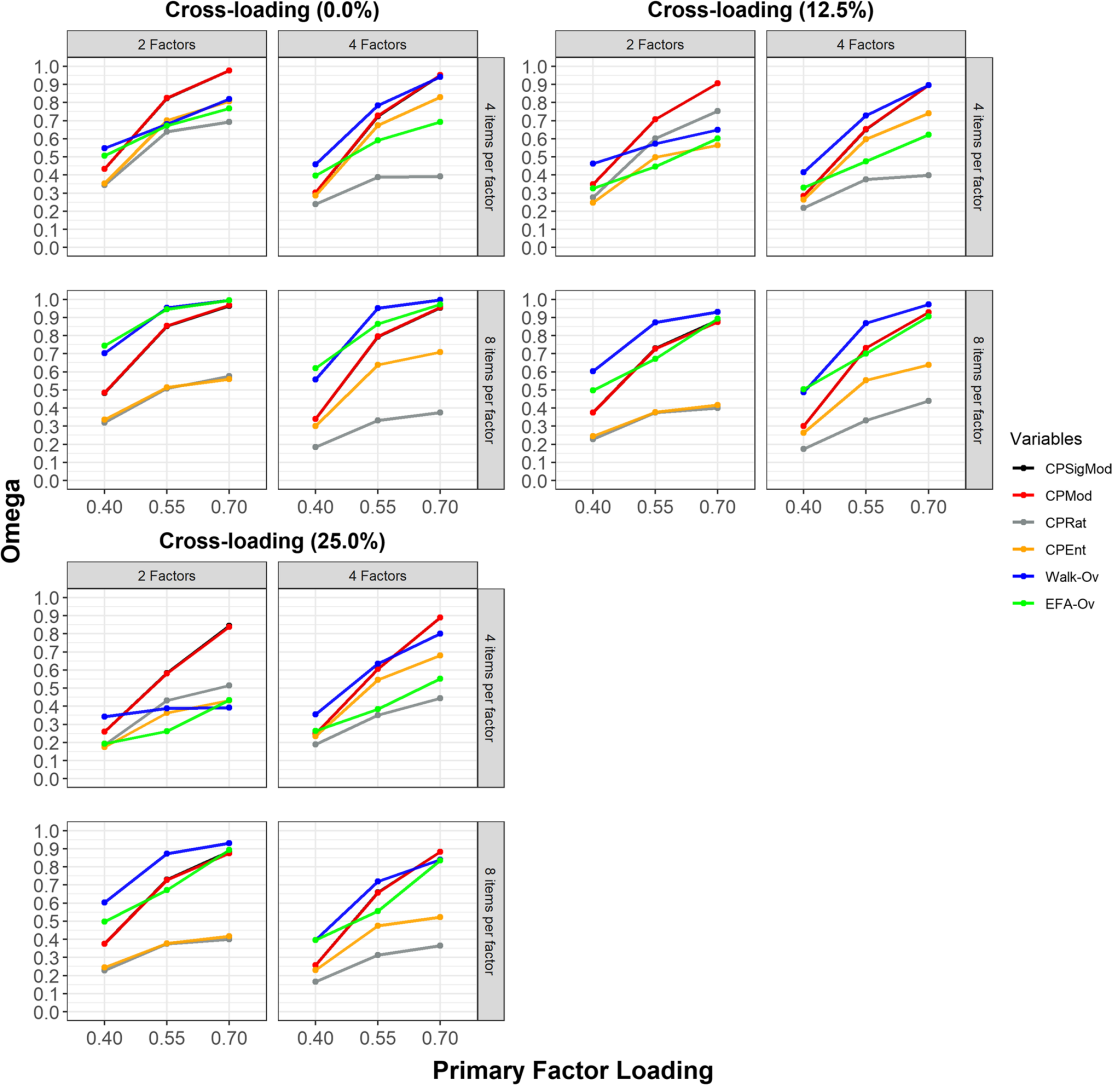
Correct node assignment according to size of primary factor loadings. CPSigMod = CP algorithm with maximization of fuzzy modularity for signed weighted networks; CPMod = CP algorithm with maximization of fuzzy modularity for weighted networks; CPRat = CP algorithm with minimization of the ratio between the two largest communities when the ratio is above or equal to 2; CPEnt = CP algorithm with maximization of entropy; Walk-Ov = Walktrap algorithm with overlapping nodes identified through network loadings > = |.15|; EFA-Ov = EFA-Ov = Exploratory Factor Analysis with overlapping nodes identified through factor loadings > = |.40|. The *x*-axis indicates the size of primary factor loadings. The *y*-axis indicates the Omega index. Higher values of the Omega index indicate more accurate node assignment to communities

The correct node assignment as indicated by the Omega index according to the size of primary factor loadings for conditions with the proportions of 0.0%, 12.5%, and 25.0% of observed variables with cross-loadings is displayed in Fig. 5.

Figure 5 indicates that the performance of all methods decreased with the size of the primary factor loadings. Notably, the performance of all methods *strongly* decreased when the size of primary factor loadings was 0.40. When the size of primary factor loadings was 0.40, all algorithms underestimated or overestimated the number of dimensions, as indicated by the MAE (Supplementary Figure 9) and MBE (Supplementary Figure 10). Furthermore, the sensitivity of EGA-Ov strongly decreased when the size of primary factor loadings were 0.40 and 0.50, being one of the main reasons behind the overall poorer performance of this method compared to Walk-Ov (Supplementary Figure 6). Overall, the Walk-Ov displayed superior or equivalent performance to the other methods across most conditions. The exceptions were conditions with two factors, four variables per factor and size of primary factor loadings of 0.55 or 0.70, in which the CPMod displayed superior performance.

### Correct node assignment (by factor correlation)

**Fig. 6:**
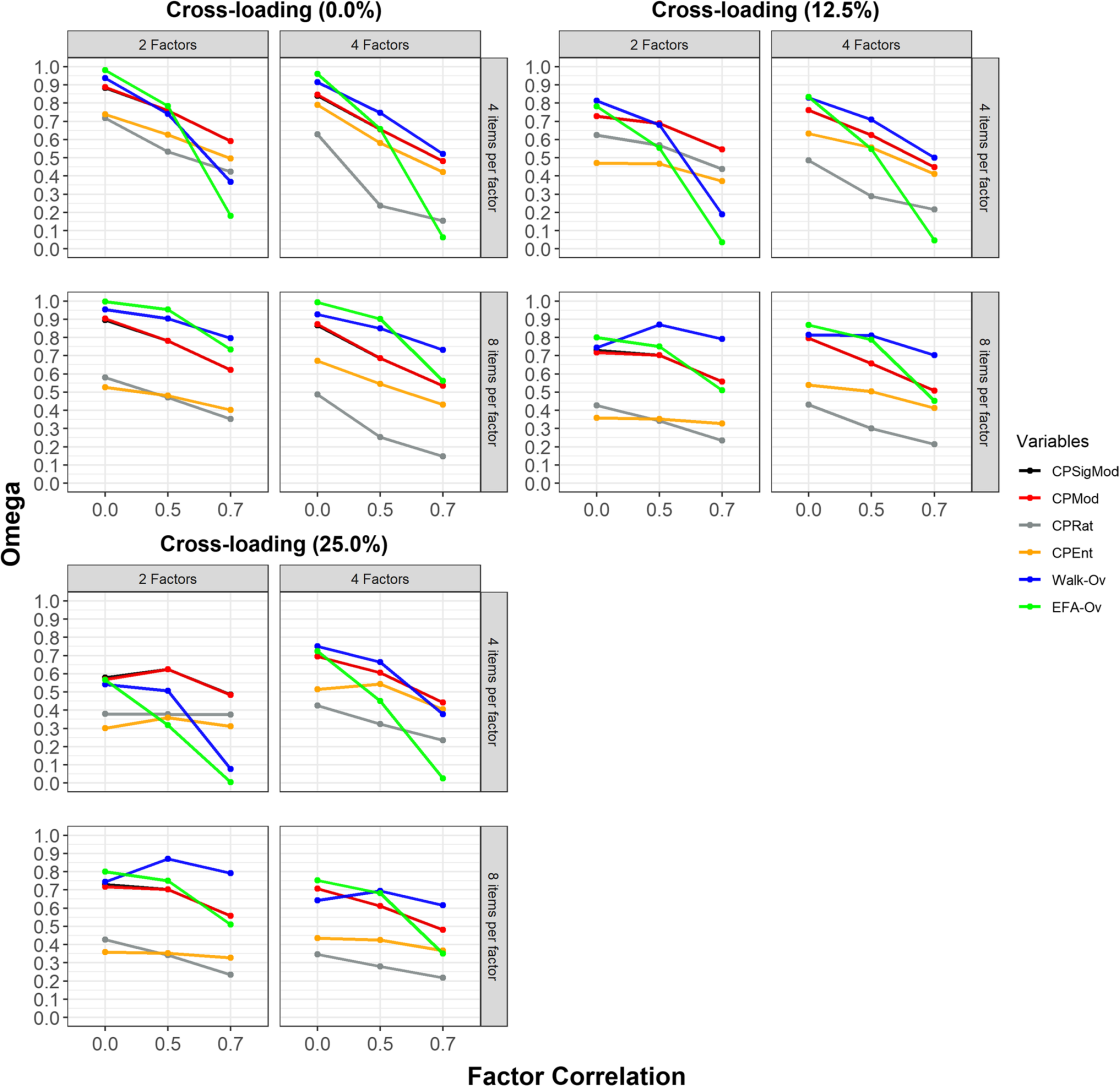
Correct node assignment according to factor correlation. CPSigMod = CP algorithm with maximization of fuzzy modularity for signed weighted networks; CPMod = CP algorithm with maximization of fuzzy modularity for weighted networks; CPRat = CP algorithm with minimization of the ratio between the two largest communities when the ratio is above or equal to 2; CPEnt = CP algorithm with maximization of entropy; Walk-Ov = Walktrap algorithm with overlapping nodes identified through network loadings > = |.15|; EFA-Ov = EFA-Ov = Exploratory Factor Analysis with overlapping nodes identified through factor loadings > = |.40|. The *x*-axis indicates the factor correlation. The *y*-axis indicates the Omega index. Higher values of the Omega index indicate more accurate node assignment to communities

The correct node assignment as indicated by the Omega index according to factor correlation for conditions with the proportions of 0.0%, 12.5%, and 25.0% of observed variables with cross-loadings is displayed in Fig. 6.

Figure 6 indicates that the performance of all methods decreased according to factor correlation. In many conditions, the performance of all methods strongly decreased when the factor correlation was 0.70. Furthermore, EGA-Ov systematically underestimated the number of dimensions in conditions when factor correlation was 0.70 as indicated by the MBE (Supplementary Figure 15), being another reason for the poorer performance of this method compared to Walk-Ov. Overall, the Walk-Ov displayed superior or equivalent performance to the other methods across most conditions. The exceptions were conditions with two factors, four variables per factor and factor correlations of 0.70, in which the CPMod displayed superior performance. The Walk-Ov performance strongly deteriorated in conditions with two factors, four variables per factor, 25% of observed variables with substantive cross-loadings and a factor correlation of 0.70. In these conditions, the Walk-Ov systematically underestimated the number of dimensions as indicated by the MBE (Supplementary Figure 15) and displayed poorer sensitivity (Supplementary Figure 11).

### Empirical example

Based on findings from the simulation study favoring first the Walk-Ov and second the CPMod over the other four methods (CPRat, CPEnt, and EFA-Ov), we chose the Walk-Ov and CPMod algorithms to demonstrate the performance of overlapping community detection algorithms compared to a non-overlapping community detection algorithm, the Walktrap algorithm, in an empirical dataset. To do so, we conducted a re-analysis of Santiago et al. (2021) to evaluate the dimensionality of the Strengths and Difficulties Questionnaire (SDQ) (Goodman, 1997). The SDQ is a 25-item brief behavioral instrument for children aged 4 to 17 years, comprising the “Hyperactivity”, “Emotional Symptoms”, “Conduct Problems”, “Peer Problems” and “Prosocial Behaviors” factors. As per the manual, ten items were reverse-scored before analysis, so higher scores indicated higher behavioral or emotional difficulties (Goodman, 1997). The SDQ is the world’s most widely used measure of child well-being and has been adopted worldwide both in research and clinical practice and translated into more than 60 languages (Stone et al., 2010).

Despite its widespread use, the dimensionality of the SDQ has been deemed an “ongoing controversy” (Goodman et al., 2010, p. 1179). The reason is that, instead of the SDQ theoretical five-factor structure (“Hyperactivity”, “Emotional Symptoms”, “Conduct Problems”, “Peer Problems” and “Prosocial Behaviors”), several studies have reported models with three factors (Gómez-Beneyto et al., 2013) and four factors (Liu et al., 2013), and other non-theoretical structures. Furthermore, systematic reviews have consistently indicated mixed (“good RMSEA and bad CFI” (Lai & Green, 2016, p. 221)) or poor model fit of the SDQ theoretical five-factor structure (Kersten et al., 2016). Garrido et al. (2018) and Santiago et al. (2021) hypothesized that the poor model fit of the five-factor structure was due to the presence of observed variables with strong cross-loadings. For instance, the original analysis by Santiago et al. (2021) evaluated the community structure with the Walktrap algorithm which does not account for overlapping symptoms.

**Fig. 7:**
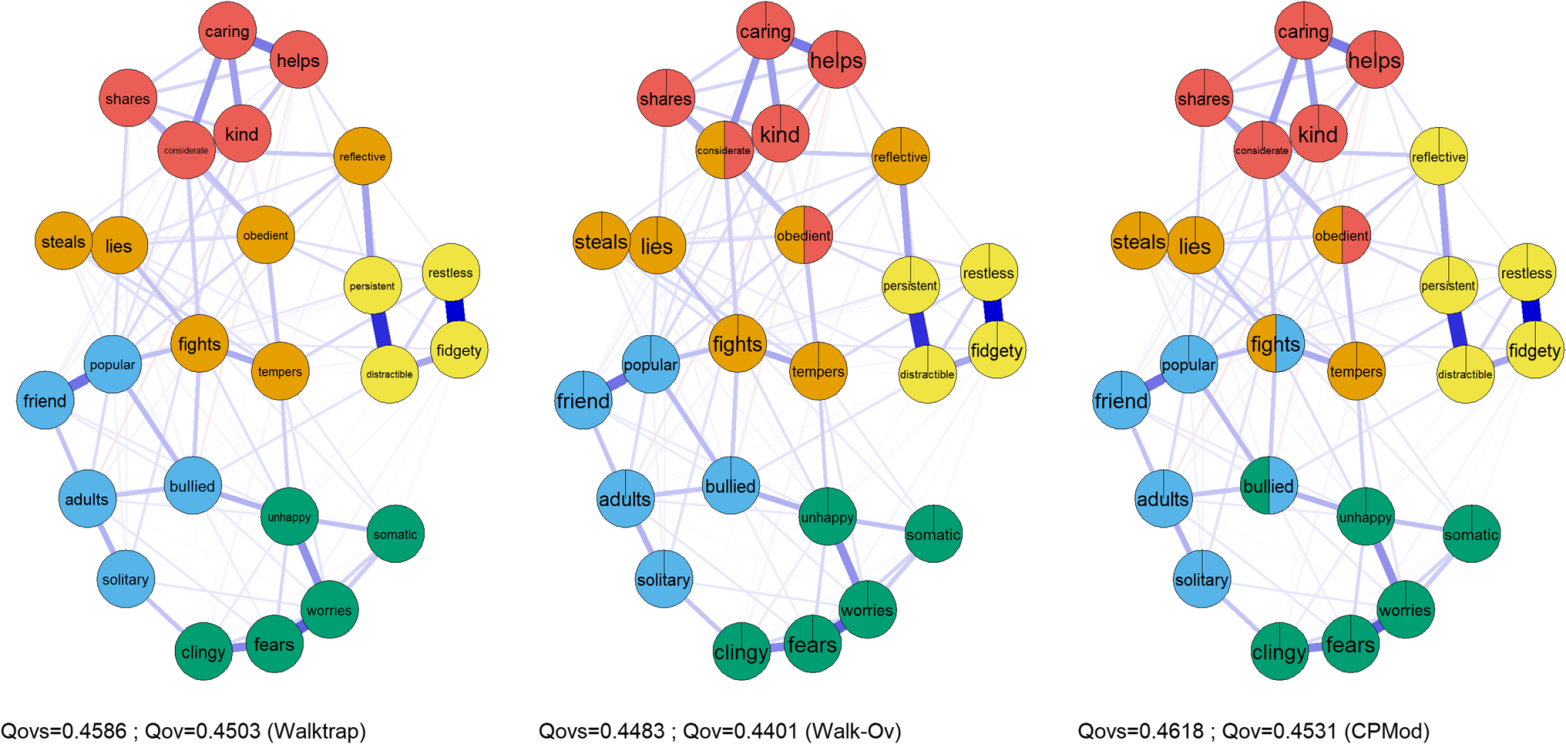
The SDQ community structure according to non-overlapping (Walktrap) and two overlapping (Walk-Ov and CPMod) community detection algorithms. Walk-Ov = Walktrap algorithm with overlapping nodes identified through network loadings > = |.15|; CPMod = CP algorithm with maximization of fuzzy modularity for weighted networks; Qovs = fuzzy modularity for signed weighted networks; Qov = fuzzy modularity for weighted networks. The *left panel* indicates the SDQ psychological networks with node assignment according to the Walktrap algorithm. The *middle panel* indicates the SDQ psychological networks with node assignment according to the Walk-Ov algorithm. The *right panel* indicates the SDQ psychological network with node assignment according to the CPMod algorithm

We conducted a re-analysis of Santiago et al. (2021) with the CPMod algorithm. The SDQ version for children aged 4–10 years was evaluated and data was from the Longitudinal Study of Australian Children (LSAC) Wave 5, B Cohort (*n* = 4,004). In-depth details about the LSAC, ethical approval and the original analysis can be found in Santiago et al. (2021). All item labels are reported in Supplementary Table 4. The polychoric correlation matrix can be found in Supplementary Table 5. The comparison between the community assignment from the Walktrap algorithm, the Walk-Ov and the CPMod is displayed in Fig. 7.

In cases where the “ground truth” regarding the network communities is not known (such as the network communities of the SDQ), the fuzzy modularity can potentially be used (instead of the Omega index) to inform the best community partitioning, a procedure which is common practice in network science (Chen et al., 2010; Tsung et al., 2020). The partitioning with five communities and three overlapping nodes established by the CPMod increased the fuzzy modularity (Qovs = 0.4618; Qov = 0.4531) compared to the partitioning with five communities and two overlapping nodes established by the Walk-Ov algorithm (Qovs = 0.4601; Qov = 0.4517) and the partitioning with five communities and no overlapping nodes established by the Walktrap algorithm which presented the lowest value (Qovs = 0.4586; Qov = 0.4503).

The re-analysis with CPMod (which provided the best community partitioning) indicates the following overlapping nodes: *bullied*,* fights*, and *obedient*. Two examples of overlapping nodes (*bullied* and *obedient)* are discussed. Firstly, the item *bullied* was an overlapping node across the “Peer Problems” and “Emotional Problems” dimensions. This is consistent with a robust body of research on peer victimization among children indicating that victims often experience emotional problems, including loneliness and anxiety (Reijntjes et al., 2010). Secondly, the item *obedient* was an overlapping node across the “Conduct Problems” and “Prosocial Behaviors” dimensions. The multidimensionality of the *obedient* item has been extensively reported by studies investigating the dimensionality of the caregiver-informant SDQ, including as early as in the initial studies conducted by Goodman (2001). In addition to loading on its theoretical dimension (“Conduct Problems”), previous studies have indicated that the item obedient displayed cross-loadings with the dimensions of “Prosocial Behaviors” (Dickey & Blumberg, 2004; Gómez-Beneyto et al., 2013; Liu et al., 2013; Matsuishi et al., 2008; Smedje et al., 1999) and “Hyperactivity” (Dickey & Blumberg, 2004; Gómez-Beneyto et al., 2013; Smedje et al., 1999).

The re-analysis of Santiago et al. (2021) made clear the presence of several overlapping symptoms in the SDQ psychological network, one of the potential reasons why the theoretical five-factor structure (which requires one item to be associated with a single factor) has consistently displayed poor fit across multiple studies and cannot be replicated as the correct dimensionality of the SDQ.

## Discussion

The current study conducted a large simulation study to evaluate the performance of the CP algorithm to identify overlapping symptoms in psychological networks, also comparing it with two distinct methods (Walk-Ov and EGA-Ov). Considering that the correspondence between factors and non-overlapping communities (Golino & Epskamp, 2017) seems to weaken in the presence of substantial cross-loadings, Brandenburg and Papenberg (2022, p. 22) recently discussed that “the correspondence between overlapping communities and factors remains an open question” in psychology. Our simulation study provided initial evidence towards answering this question by demonstrating that, when data is generated from latent factors including variables with substantive (and equally sized) cross-loadings, overlapping community detection algorithms (such as the Walk-Ov and the CPMod) can accurately identify the dimensionality (i.e., network communities) *and* the variables with substantive cross-loadings (i.e., overlapping symptoms) in several plausible scenarios (e.g., different number of items per factor, factor correlations, etc.). Overall, our findings indicated that the CPMod had a superior performance than the CPRat or CPEnt across all conditions, notably due to fuzzy modularity being the only metric that considers edge weights. When all algorithms were compared, the Walk-Ov displayed superior performance across most conditions. The implications for overlapping community detection in psychological networks are discussed below.

### Method performance

In the current study, we replicated findings that the performance of community detection algorithms (and also EFA based on Parallel Analysis) decreased with: (1) smaller factor loadings (Golino et al., 2020; Li et al., 2020); (2) larger factor correlations (Golino et al., 2020); (3) higher proportions of variables with cross-loadings (Brandenburg & Papenberg, 2022; Li et al., 2020) and (4) smaller sample sizes (Golino et al., 2020; Li et al., 2020). These findings have several implications. Firstly, while the performance of the CPMod was comparable to the Walk-Ov (best method) under *ideal* conditions (e.g., primary factor loadings of 0.70), the CPMod performance deteriorated in more realistic scenarios (e.g., primary factor loadings of 0.55). This is possibly due to the well-known limitation of the CP algorithm in detecting communities according to their standard definition (i.e., dense subgraph of the network), detecting instead chains of clique (which can have low internal density) (Brusco et al., 2023; Fortunato, 2010). For this reason, alternatives to the CP algorithm, such as a maximum-clique-based set-covering approach, have been recently developed (Brusco et al., 2023).

Secondly, while our study showed that the Walk-Ov demonstrated good accuracy in many scenarios commonly encountered by empirical researchers (e.g., primary factor loadings of 0.55, ordinal data, factor correlation of 0.50), the algorithm performance was uneven (e.g., in certain conditions it had around a 35% accuracy, while in others it had a 90% accuracy), and there is room for algorithmic improvement. Future studies should investigate other overlapping community detection algorithms (e.g., BigClam (Yang & Leskovec, 2013), Copra (Gregory, 2007), Infomap (Xie et al., 2013), ModuLand (Kovács et al., 2010)) and compare them with the Walk-Ov in future simulation studies.

Thirdly, our study also showed that overlapping community detection algorithms (e.g., Walk-Ov, CPMod) were accurate when no variables with substantive cross-loadings were present. These findings are consistent with studies from network science, indicating that overlapping community detection algorithms “perform relatively well when the number of memberships per node is set as one, i.e., when the community detection problem is solved as a partition problem” in simulated and empirical networks (e.g., social networks) (Vieira et al., 2020). That is, non-overlapping community detection algorithms are also accurate and can potentially substitute overlapping community detection algorithms when no overlapping nodes are present.

### Recommendations for practice

Based on the findings of the current study, in case researchers have to choose one algorithm to conduct overlapping community detection, the Walk-Ov is the preferred algorithm. Nonetheless, in practice, researchers usually have access to more than one community-detection algorithm. As such, we encourage empirical researchers to employ distinct overlapping and non-overlapping community-detection algorithms and identify the community partitioning (with or without overlap) that maximizes fuzzy modularity (until metrics are developed specifically for psychological networks). For instance, in the empirical example we provided the CPMod (instead of Walk-Ov), which suggested the best partitioning. Despite the well-known limitations regarding the use of modularity to evaluate community partitioning (e.g., resolution problem, penalization of unidimensional structures, worse performance when communities are unequally sized), modularity continues to be the most popular approach for determining the quality of a community assignment in networks since its proposal two decades ago (Fortunato & Newman, 2022).

Furthermore, besides the inclusion of (*fuzzy*)* modularity for signed weighted networks* in the CPSigMod not providing additional accuracy in community assignment compared to the CPMod, there are theoretical concerns regarding the use of the *modularity for signed weighted networks *proposed by Gómez et al. (2009)in psychological networks. The reason is that Gómez et al. (2009) conceptualized positively signed edges as contributing to the “attractiveness” of nodes to a community and negatively signed edges as contributing to the “repulsiveness” of nodes to a community. However, this interpretation is not valid for psychological networks since negatively signed edges can occur due to items that measure the construct with negatively valenced wording instead of due to items that measure features that are “repulsive” (i.e., opposite) to the construct. For example, among the five items of the SDQ subscale named “Conduct Problems”, the item *obedient* (“Generally well behaved, usually does what adults request”) is the only one with negatively valanced wording (so higher scores indicate fewer conduct problems). In case there is no reverse scoring, the item *obedient* will establish negatively signed edges (i.e., negative partial correlations) with the other four items of the “Conduct Problems” subscale. That is, the *obedient* item measures the construct of “Conduct Problems”^4^ and the negatively signed edges established with the other four items occur solely due to this item’s negatively valenced wording (instead of indicating that the *obedient* item measures something that is opposite, “repulsive” to “Conduct Problems”). For this reason, the use of (*fuzzy*) *modularity for signed weighted networks *is not recommended in psychological networks and (*fuzzy*) *modularity for weighted networks *based on absolute values of edge weights should be preferred.

Finally, we also recommend that future simulation and empirical studies should compare overlapping algorithms that directly seek to maximize fuzzy modularity (such as integer programming or algorithms using heuristics; Brusco et al., 2022b) with those that do not directly seek to maximize fuzzy modularity but in which fuzzy modularity is used as a post hoc metric for model selection (such as the CPMod).

### Limitations

Despite constituting a large simulation study, there are also several conditions that the current study did not evaluate. Firstly, there was an equal number of variables per factor and research has shown that modularity-based methods are less precise in conditions with unequal numbers of variables per factor (Brusco et al., 2022b). While this limitation with respect to unequally sized communities is potentially more relevant to the use of fuzzy modularity as a metric for model selection in the CP algorithm (or other algorithms) than to the performance of the CP algorithm (or the Walktrap algorithm) itself, future simulation studies should further investigate the impact of unequally sized communities on the performance of overlapping community detection algorithms. Secondly, the number of communities evaluated was small (up to four communities), while a larger number has been identified in empirical studies (Lange & Zickfeld, 2021). Thirdly, the simulation did not evaluate unidimensional structures and modularity is known to incorrectly penalize unidimensional structures (favoring two or more dimensions) (Christensen, 2022). Fourthly, the simulation only investigated conditions in which the substantive cross-loadings were smaller than the primary factor loadings, but conditions in which the substantive cross-loadings were larger than the primary factor loadings are also possible. Fourthly, all non-substantive cross-loadings were very weak (i.e., “noise” cross-loadings to emulate measurement error). It is important to evaluate whether the Walk-Ov retains good sensitivity and specificity when bridging symptoms, variables with stronger cross-loadings but still belonging to a unique community, are present in the network. Fifthly, the data categories (continuous, polytomous, and dichotomous) were simulated without skewness, while psychological data are usually skewed. Sixthly, our simulated strategy generated *sparse* overlapping regions between communities, so it’s also important to develop simulation benchmarks of *dense* overlapping regions between communities (Vieira et al., 2020; Yang & Leskovec, 2013). Psychological networks with *dense* overlap between communities have been observed in empirical research (Lange & Zickfeld, 2021). The performance of the algorithms investigated here can potentially deteriorate in these more complex scenarios.

### Contributions and avenues of future research

One main contribution of the current study was to propose an initial benchmark for simulating psychological networks with overlapping symptoms. Following previous benchmarks (Lancichinetti & Fortunato, 2009) and theoretical recommendations (Reichardt & Bornholdt, 2006) from network science, we proposed an empirical criterion for overlapping symptoms (equally sized cross-loadings across two factors). Based on this empirical criterion, psychological networks with overlapping symptoms were simulated from factor models. However, in contrast to traditional benchmarks from network science for overlapping communities (Lancichinetti & Fortunato, 2009), our benchmark takes into account the specificities of psychological networks (small partial correlation networks of multivariate variables) (Golino & Epskamp, 2017). Overall, the findings of our simulation study are contingent upon this empirical criterion for overlapping symptoms.

In terms of avenues of future research, empirical research on overlapping symptoms is emerging in psychology and cumulative research evidence should elucidate the type of overlap (e.g., dense x sparse) that is the most representative of psychological networks. One fundamental challenge associated with this elucidation is that methodological guidelines in traditional psychological research (from the perspective of the *latent trait theory*) recommended the removal of variables with substantive cross-loadings across two or more factors from psychological measures and, consequently, from psychological research (Comrey & Lee, 2013). From a network perspective on psychological measurement (Christensen et al., 2020), these variables with substantive cross-loadings are overlapping symptoms, so they should be considered as an important constituent of the network and can be retained in practice (without necessarily being included in the construction of summed scale scores). As such, the identification of psychological networks with overlapping symptoms will be facilitated by research and development of psychological measures conducted exclusively within the network perspective (rather than the latent trait perspective) (Christensen et al., 2020). Furthermore, the use of psychological measures developed from a network perspective will also help elucidate whether the *theoretical* distinction between *bridging* and *overlapping symptoms* (Jones et al., 2021) is substantive and clinically meaningful. Importantly, the evaluation of empirical networks will inform which type of symptom is more common, symptoms that are strongly conditionally associated with one community and more weakly associated with other communities (i.e., bridging symptoms) or symptoms that are equally strongly associated with more than one community (i.e., overlapping symptoms according to the definition of network science; Lancichinetti & Fortunato, 2009; Reichardt & Bornholdt, 2006, p. 7). The evaluation of overlapping community detection algorithms in psychological networks is a new and fundamental research question that just started to be explored.

## Conclusions

The identification of overlapping symptoms in psychological networks is a crucial feature of network psychometrics. To the best of our knowledge, our study was the first to propose a benchmark for simulating psychological networks with overlapping symptoms. The findings from our study indicated that, while the CPMod constituted an improvement over the CPRat and CPEnt, the Walk-Ov algorithm was the most accurate across most conditions. Future simulation studies should evaluate other overlapping community detection algorithms and compare their performance with the Walk-Ov algorithm in psychological networks.

### Supplementary Information

Below is the link to the electronic supplementary material.

Supplementary file1 (DOCX 2.57 MB)

## Data Availability

This simulation study has not been pre-registered. For reproducibility purposes, the data from this simulation study is available at the Open Science Framework repository (https://osf.io/6c9bw/). The LSAC data cannot be publicly shared because of its sensitive nature. Researchers interested in accessing data from the LSAC should apply for data access to the Australian Data Archive (https://dataverse.ada.edu.au/).

## Footnotes

1 The term “consistent” here does not refer to the statistical property of *consistency*, defined as the property of an estimator in which the estimate converges in probability to the true parameter value as the sample size increases (Lehmann & Casella, 2006). We use the term statistical consistency, instead, to indicate that different data generating mechanisms, such as those suggested by certain latent variable and network models, can create similar sets of statistical relationships, extending into the first (means) and second (variance-covariance matrix) moments (Christensen & Golino, 2021).

2 In the case of a factor model with two correlated factors (factors 1 and 2), the communality of a variable X can be defined as ${h}_{X}^{2}={\lambda }_{X1}^{2}+{\lambda }_{X2}^{2}+2{\lambda }_{X1}{\lambda }_{X2}\phi$ where ${h}_{X}^{2}$ is the communality of variable X, ${\lambda }_{X1}$ is the factor loading of variable X on factor 1, ${\lambda }_{X2}$ is the factor loading of variable X on factor 2, and $\phi$ is the correlation between factors 1 and 2 (see Appendix 3.1; Brown, 2014). In the case of equally sized cross-loadings, the formula reduces to ${h}_{X}^{2}=2{\lambda }^{2} +2{\lambda }^{2}\phi$. After substituting 0.90 in ${h}_{X}^{2}$, rearranging the equation and transforming it into an inequality, it indicates that the equally sized cross-loading $\lambda$ should be smaller than $\sqrt{\frac{0.9}{2+2\phi }}$ so the communality does not exceed 0.90. In our study, this formula could be employed (to determine the maximum equally sized cross-loadings so the communality does not exceed 0.90) since variables were restricted to have substantive and equally sized cross-loadings *only across two factors*. In the simulation conditions with more than two factors, we did not expand this formula to consider the contribution of *non-substantive* cross-loadings to the communality due to their small size (i.e., randomly draw from a normal distribution ℵ(0.00,0.05)). Nonetheless, we *empirically* tested every reproduced population correlation matrix to ensure that communalities of any of the variables did not exceed 0.90 (and in case one of the communalities exceeded 0.90, the reproduced population correlation matrix was re-generated) (Christensen et al., 2023).

3 According to the empirical definition used in this study based on Lancichinetti and Fortunato (2009), a variable with substantive and equally sized cross-loadings of 0.30 across two factors constitutes an overlapping symptom. The limitation of methods such as EFA-Ov (that uses a cut-off of 0.40 for cross-loadings, described below) to detect these symptoms is not a limitation of our simulation strategy but instead an inherent limitation of methods that rely on ‘hard’ cut-offs.

4 Notably, the overlap of the *obedient* item between two communities is discussed in the section “Empirical example”.

## Code availability

This simulation study has not been pre-registered. For reproducibility purposes, all the R code used in this simulation study is available at the Open Science Framework repository (https://osf.io/6c9bw/).
