# Supplementary material for: Comparing the Clique Percolation algorithm to other overlapping community detection algorithms in psychological networks: A Monte Carlo simulation study
Source: Behav Res Methods. 2024 May 1;56(7):7219–40. doi: 10.3758/s13428-024-02415-2 (PMC11362237; doi:10.3758/s13428-024-02415-2)
Supplement: Supplementary file 1 — Supplementary file1 (DOCX 2.57 MB) [file 13428_2024_2415_MOESM1_ESM.docx]

Comparing the Clique Percolation with other overlapping community detection algorithms in psychological networks: A Monte Carlo simulation study
(Supplementary Material)

Pedro Henrique Ribeiro Santiago^[[1]](#footnote-1)^

Gustavo Soares^[[2]](#footnote-2)^

Adrian Quintero^[[3]](#footnote-3)^

Lisa Jamieson^[[4]](#footnote-4)^

Supplementary Table 1: Outcomes of the simulation study (Omega, Sensitivity, and Specificity).

|  | Omega | | | | | | Sensitivity | | | | | | Specificity | | | | | |
| --- | --- | --- | --- | --- | --- | --- | --- | --- | --- | --- | --- | --- | --- | --- | --- | --- | --- | --- |
| Condition | CPSigMod | CPMod | CPRat | CPEnt | Walk-Ov | EFA-Ov | CPSigMod | CPMod | CPRat | CPEnt | Walk-Ov | EFA-Ov | CPSigMod | CPMod | CPRat | CPEnt | Walk-Ov | EFA-Ov |
| Cont.0.40Load.000Cross.4Var.300Size.2Fac.0.00Fcor | 0.49 | 0.49 | 0.40 | 0.38 | 0.66 | 0.99 | - | - | - | - | - | - | - | - | - | - | - | - |
| Cont.0.40Load.000Cross.4Var.300Size.2Fac.0.50Fcor | 0.28 | 0.28 | 0.19 | 0.20 | 0.42 | 0.54 | - | - | - | - | - | - | - | - | - | - | - | - |
| Cont.0.40Load.000Cross.4Var.300Size.2Fac.0.70Fcor | 0.16 | 0.16 | 0.11 | 0.13 | 0.21 | 0.06 | - | - | - | - | - | - | - | - | - | - | - | - |
| Cont.0.40Load.000Cross.4Var.500Size.2Fac.0.00Fcor | 0.86 | 0.86 | 0.74 | 0.76 | 0.97 | 0.99 | - | - | - | - | - | - | - | - | - | - | - | - |
| Cont.0.40Load.000Cross.4Var.500Size.2Fac.0.50Fcor | 0.53 | 0.53 | 0.43 | 0.45 | 0.68 | 0.52 | - | - | - | - | - | - | - | - | - | - | - | - |
| Cont.0.40Load.000Cross.4Var.500Size.2Fac.0.70Fcor | 0.23 | 0.23 | 0.14 | 0.19 | 0.27 | 0.06 | - | - | - | - | - | - | - | - | - | - | - | - |
| Cont.0.40Load.000Cross.4Var.1000Size.2Fac.0.00Fcor | 0.99 | 0.99 | 0.85 | 0.86 | 1.00 | 1.00 | - | - | - | - | - | - | - | - | - | - | - | - |
| Cont.0.40Load.000Cross.4Var.1000Size.2Fac.0.50Fcor | 0.69 | 0.69 | 0.59 | 0.63 | 0.79 | 0.85 | - | - | - | - | - | - | - | - | - | - | - | - |
| Cont.0.40Load.000Cross.4Var.1000Size.2Fac.0.70Fcor | 0.35 | 0.35 | 0.27 | 0.27 | 0.34 | 0.15 | - | - | - | - | - | - | - | - | - | - | - | - |
| Cont.0.40Load.000Cross.4Var.300Size.4Fac.0.00Fcor | 0.21 | 0.21 | 0.16 | 0.17 | 0.41 | 0.91 | - | - | - | - | - | - | - | - | - | - | - | - |
| Cont.0.40Load.000Cross.4Var.300Size.4Fac.0.50Fcor | 0.06 | 0.06 | 0.04 | 0.05 | 0.17 | 0.23 | - | - | - | - | - | - | - | - | - | - | - | - |
| Cont.0.40Load.000Cross.4Var.300Size.4Fac.0.70Fcor | 0.05 | 0.05 | 0.03 | 0.04 | 0.09 | 0.02 | - | - | - | - | - | - | - | - | - | - | - | - |
| Cont.0.40Load.000Cross.4Var.500Size.4Fac.0.00Fcor | 0.72 | 0.72 | 0.65 | 0.72 | 0.93 | 0.98 | - | - | - | - | - | - | - | - | - | - | - | - |
| Cont.0.40Load.000Cross.4Var.500Size.4Fac.0.50Fcor | 0.27 | 0.27 | 0.19 | 0.24 | 0.51 | 0.32 | - | - | - | - | - | - | - | - | - | - | - | - |
| Cont.0.40Load.000Cross.4Var.500Size.4Fac.0.70Fcor | 0.13 | 0.13 | 0.08 | 0.10 | 0.24 | 0.03 | - | - | - | - | - | - | - | - | - | - | - | - |
| Cont.0.40Load.000Cross.4Var.1000Size.4Fac.0.00Fcor | 0.95 | 0.95 | 0.79 | 0.92 | 1.00 | 1.00 | - | - | - | - | - | - | - | - | - | - | - | - |
| Cont.0.40Load.000Cross.4Var.1000Size.4Fac.0.50Fcor | 0.39 | 0.39 | 0.29 | 0.38 | 0.76 | 0.60 | - | - | - | - | - | - | - | - | - | - | - | - |
| Cont.0.40Load.000Cross.4Var.1000Size.4Fac.0.70Fcor | 0.13 | 0.14 | 0.10 | 0.12 | 0.31 | 0.02 | - | - | - | - | - | - | - | - | - | - | - | - |
| Cont.0.40Load.000Cross.8Var.300Size.2Fac.0.00Fcor | 0.45 | 0.45 | 0.30 | 0.29 | 0.57 | 1.00 | - | - | - | - | - | - | - | - | - | - | - | - |
| Cont.0.40Load.000Cross.8Var.300Size.2Fac.0.50Fcor | 0.21 | 0.21 | 0.14 | 0.16 | 0.38 | 0.90 | - | - | - | - | - | - | - | - | - | - | - | - |
| Cont.0.40Load.000Cross.8Var.300Size.2Fac.0.70Fcor | 0.11 | 0.11 | 0.05 | 0.08 | 0.26 | 0.24 | - | - | - | - | - | - | - | - | - | - | - | - |
| Cont.0.40Load.000Cross.8Var.500Size.2Fac.0.00Fcor | 0.95 | 0.95 | 0.61 | 0.55 | 1.00 | 1.00 | - | - | - | - | - | - | - | - | - | - | - | - |
| Cont.0.40Load.000Cross.8Var.500Size.2Fac.0.50Fcor | 0.60 | 0.60 | 0.42 | 0.42 | 0.89 | 0.97 | - | - | - | - | - | - | - | - | - | - | - | - |
| Cont.0.40Load.000Cross.8Var.500Size.2Fac.0.70Fcor | 0.28 | 0.28 | 0.16 | 0.20 | 0.63 | 0.45 | - | - | - | - | - | - | - | - | - | - | - | - |
| Cont.0.40Load.000Cross.8Var.1000Size.2Fac.0.00Fcor | 0.98 | 0.99 | 0.65 | 0.60 | 1.00 | 1.00 | - | - | - | - | - | - | - | - | - | - | - | - |
| Cont.0.40Load.000Cross.8Var.1000Size.2Fac.0.50Fcor | 0.79 | 0.79 | 0.53 | 0.55 | 0.99 | 1.00 | - | - | - | - | - | - | - | - | - | - | - | - |
| Cont.0.40Load.000Cross.8Var.1000Size.2Fac.0.70Fcor | 0.45 | 0.45 | 0.32 | 0.37 | 0.87 | 0.83 | - | - | - | - | - | - | - | - | - | - | - | - |
| Cont.0.40Load.000Cross.8Var.300Size.4Fac.0.00Fcor | 0.14 | 0.14 | 0.09 | 0.13 | 0.23 | 1.00 | - | - | - | - | - | - | - | - | - | - | - | - |
| Cont.0.40Load.000Cross.8Var.300Size.4Fac.0.50Fcor | 0.02 | 0.02 | 0.01 | 0.01 | 0.05 | 0.64 | - | - | - | - | - | - | - | - | - | - | - | - |
| Cont.0.40Load.000Cross.8Var.300Size.4Fac.0.70Fcor | 0.02 | 0.02 | 0.01 | 0.01 | 0.05 | 0.08 | - | - | - | - | - | - | - | - | - | - | - | - |
| Cont.0.40Load.000Cross.8Var.500Size.4Fac.0.00Fcor | 0.89 | 0.89 | 0.48 | 0.72 | 0.97 | 1.00 | - | - | - | - | - | - | - | - | - | - | - | - |
| Cont.0.40Load.000Cross.8Var.500Size.4Fac.0.50Fcor | 0.29 | 0.29 | 0.16 | 0.26 | 0.67 | 0.91 | - | - | - | - | - | - | - | - | - | - | - | - |
| Cont.0.40Load.000Cross.8Var.500Size.4Fac.0.70Fcor | 0.12 | 0.12 | 0.07 | 0.11 | 0.38 | 0.13 | - | - | - | - | - | - | - | - | - | - | - | - |
| Cont.0.40Load.000Cross.8Var.1000Size.4Fac.0.00Fcor | 0.98 | 0.99 | 0.56 | 0.80 | 1.00 | 1.00 | - | - | - | - | - | - | - | - | - | - | - | - |
| Cont.0.40Load.000Cross.8Var.1000Size.4Fac.0.50Fcor | 0.52 | 0.52 | 0.26 | 0.49 | 0.98 | 1.00 | - | - | - | - | - | - | - | - | - | - | - | - |
| Cont.0.40Load.000Cross.8Var.1000Size.4Fac.0.70Fcor | 0.22 | 0.22 | 0.12 | 0.19 | 0.73 | 0.50 | - | - | - | - | - | - | - | - | - | - | - | - |
| Cont.0.40Load.125Cross.4Var.300Size.2Fac.0.00Fcor | 0.26 | 0.26 | 0.18 | 0.15 | 0.40 | 0.69 | 11.00 | 11.00 | 10.00 | 8.00 | 10.00 | 0.00 | 96.14 | 96.14 | 95.57 | 95.71 | 98.29 | 100.00 |
| Cont.0.40Load.125Cross.4Var.300Size.2Fac.0.50Fcor | 0.27 | 0.27 | 0.20 | 0.18 | 0.38 | 0.16 | 31.00 | 31.00 | 33.00 | 29.00 | 58.00 | 0.00 | 92.86 | 92.86 | 90.43 | 91.57 | 89.43 | 100.00 |
| Cont.0.40Load.125Cross.4Var.300Size.2Fac.0.70Fcor | 0.17 | 0.16 | 0.13 | 0.12 | 0.22 | 0.01 | 35.00 | 34.00 | 44.00 | 36.00 | 46.00 | 0.00 | 92.86 | 92.86 | 89.71 | 92.71 | 93.29 | 100.00 |
| Cont.0.40Load.125Cross.4Var.500Size.2Fac.0.00Fcor | 0.60 | 0.59 | 0.52 | 0.39 | 0.71 | 0.73 | 44.00 | 44.00 | 47.00 | 28.00 | 36.00 | 0.00 | 96.86 | 96.71 | 96.43 | 96.57 | 97.29 | 100.00 |
| Cont.0.40Load.125Cross.4Var.500Size.2Fac.0.50Fcor | 0.44 | 0.44 | 0.33 | 0.31 | 0.66 | 0.28 | 55.00 | 55.00 | 55.00 | 42.00 | 82.00 | 0.00 | 90.43 | 90.43 | 90.43 | 94.00 | 90.57 | 100.00 |
| Cont.0.40Load.125Cross.4Var.500Size.2Fac.0.70Fcor | 0.22 | 0.22 | 0.15 | 0.16 | 0.25 | 0.01 | 35.00 | 35.00 | 39.00 | 33.00 | 41.00 | 0.00 | 93.86 | 93.86 | 92.14 | 93.57 | 93.86 | 100.00 |
| Cont.0.40Load.125Cross.4Var.1000Size.2Fac.0.00Fcor | 0.85 | 0.85 | 0.80 | 0.62 | 0.91 | 0.75 | 67.00 | 65.00 | 68.00 | 36.00 | 66.00 | 0.00 | 97.71 | 98.00 | 95.71 | 96.43 | 99.57 | 100.00 |
| Cont.0.40Load.125Cross.4Var.1000Size.2Fac.0.50Fcor | 0.53 | 0.53 | 0.44 | 0.40 | 0.73 | 0.47 | 62.00 | 62.00 | 63.00 | 51.00 | 94.00 | 0.00 | 93.57 | 93.57 | 91.86 | 95.43 | 90.43 | 100.00 |
| Cont.0.40Load.125Cross.4Var.1000Size.2Fac.0.70Fcor | 0.21 | 0.21 | 0.18 | 0.16 | 0.23 | 0.02 | 28.00 | 28.00 | 34.00 | 31.00 | 39.00 | 0.00 | 96.29 | 96.29 | 95.14 | 94.86 | 93.57 | 100.00 |
| Cont.0.40Load.125Cross.4Var.300Size.4Fac.0.00Fcor | 0.14 | 0.14 | 0.11 | 0.13 | 0.28 | 0.77 | 6.00 | 6.00 | 4.50 | 6.00 | 1.00 | 0.00 | 98.86 | 98.86 | 99.07 | 98.93 | 99.86 | 100.00 |
| Cont.0.40Load.125Cross.4Var.300Size.4Fac.0.50Fcor | 0.12 | 0.12 | 0.08 | 0.10 | 0.22 | 0.16 | 18.50 | 18.50 | 14.50 | 21.00 | 22.50 | 0.00 | 95.79 | 95.79 | 95.86 | 95.71 | 97.57 | 100.00 |
| Cont.0.40Load.125Cross.4Var.300Size.4Fac.0.70Fcor | 0.12 | 0.12 | 0.08 | 0.11 | 0.17 | 0.00 | 28.00 | 28.00 | 25.50 | 34.00 | 45.50 | 0.00 | 91.21 | 91.21 | 89.79 | 90.57 | 94.36 | 100.00 |
| Cont.0.40Load.125Cross.4Var.500Size.4Fac.0.00Fcor | 0.56 | 0.56 | 0.50 | 0.52 | 0.68 | 0.81 | 30.00 | 30.50 | 21.00 | 20.00 | 8.50 | 0.00 | 96.57 | 96.57 | 96.64 | 96.29 | 99.64 | 100.00 |
| Cont.0.40Load.125Cross.4Var.500Size.4Fac.0.50Fcor | 0.29 | 0.29 | 0.19 | 0.26 | 0.52 | 0.28 | 39.50 | 39.50 | 37.50 | 46.50 | 51.00 | 0.00 | 90.21 | 90.21 | 87.50 | 87.43 | 96.00 | 100.00 |
| Cont.0.40Load.125Cross.4Var.500Size.4Fac.0.70Fcor | 0.15 | 0.15 | 0.09 | 0.11 | 0.29 | 0.01 | 37.00 | 37.00 | 45.50 | 52.00 | 57.50 | 0.00 | 90.29 | 90.29 | 86.50 | 87.64 | 93.71 | 100.00 |
| Cont.0.40Load.125Cross.4Var.1000Size.4Fac.0.00Fcor | 0.83 | 0.83 | 0.66 | 0.81 | 0.84 | 0.83 | 56.50 | 55.00 | 33.00 | 52.50 | 13.50 | 0.00 | 96.29 | 96.43 | 97.14 | 96.57 | 100.00 | 100.00 |
| Cont.0.40Load.125Cross.4Var.1000Size.4Fac.0.50Fcor | 0.36 | 0.36 | 0.28 | 0.33 | 0.66 | 0.53 | 38.50 | 38.50 | 35.50 | 42.50 | 55.50 | 0.00 | 91.00 | 91.00 | 90.93 | 90.71 | 97.36 | 100.00 |
| Cont.0.40Load.125Cross.4Var.1000Size.4Fac.0.70Fcor | 0.17 | 0.17 | 0.12 | 0.14 | 0.37 | 0.03 | 34.00 | 34.00 | 34.50 | 40.00 | 69.00 | 0.00 | 90.43 | 90.43 | 88.79 | 88.93 | 93.64 | 100.00 |
| Cont.0.40Load.125Cross.8Var.300Size.2Fac.0.00Fcor | 0.22 | 0.22 | 0.16 | 0.15 | 0.32 | 0.75 | 9.50 | 9.50 | 9.00 | 9.50 | 2.50 | 0.00 | 96.93 | 97.07 | 94.36 | 93.71 | 99.50 | 100.00 |
| Cont.0.40Load.125Cross.8Var.300Size.2Fac.0.50Fcor | 0.23 | 0.23 | 0.16 | 0.17 | 0.50 | 0.54 | 39.50 | 40.00 | 35.50 | 44.50 | 44.00 | 0.00 | 90.00 | 89.86 | 88.07 | 88.57 | 97.43 | 100.00 |
| Cont.0.40Load.125Cross.8Var.300Size.2Fac.0.70Fcor | 0.15 | 0.15 | 0.11 | 0.13 | 0.38 | 0.09 | 44.50 | 44.50 | 42.50 | 59.50 | 52.00 | 0.00 | 86.50 | 86.50 | 83.21 | 84.29 | 95.43 | 100.00 |
| Cont.0.40Load.125Cross.8Var.500Size.2Fac.0.00Fcor | 0.69 | 0.69 | 0.42 | 0.37 | 0.68 | 0.75 | 47.50 | 47.00 | 24.00 | 27.00 | 8.50 | 0.00 | 94.50 | 94.43 | 89.00 | 86.43 | 99.36 | 100.00 |
| Cont.0.40Load.125Cross.8Var.500Size.2Fac.0.50Fcor | 0.45 | 0.45 | 0.25 | 0.28 | 0.85 | 0.70 | 63.00 | 63.00 | 52.50 | 58.50 | 76.50 | 0.00 | 88.79 | 88.79 | 85.93 | 87.71 | 98.86 | 100.00 |
| Cont.0.40Load.125Cross.8Var.500Size.2Fac.0.70Fcor | 0.23 | 0.23 | 0.13 | 0.18 | 0.66 | 0.19 | 52.00 | 52.00 | 52.50 | 60.00 | 80.50 | 0.00 | 87.71 | 87.71 | 84.21 | 85.64 | 96.64 | 100.00 |
| Cont.0.40Load.125Cross.8Var.1000Size.2Fac.0.00Fcor | 0.88 | 0.88 | 0.53 | 0.41 | 0.74 | 0.75 | 63.00 | 63.00 | 23.50 | 17.00 | 9.00 | 0.00 | 98.86 | 98.86 | 90.00 | 87.50 | 99.86 | 100.00 |
| Cont.0.40Load.125Cross.8Var.1000Size.2Fac.0.50Fcor | 0.70 | 0.70 | 0.41 | 0.40 | 0.96 | 0.75 | 90.50 | 90.50 | 63.00 | 64.50 | 95.00 | 0.00 | 90.71 | 90.71 | 87.79 | 86.93 | 99.00 | 100.00 |
| Cont.0.40Load.125Cross.8Var.1000Size.2Fac.0.70Fcor | 0.31 | 0.31 | 0.21 | 0.23 | 0.85 | 0.44 | 58.00 | 58.00 | 52.50 | 56.50 | 97.00 | 0.00 | 88.07 | 88.07 | 88.50 | 88.86 | 96.71 | 100.00 |
| Cont.0.40Load.125Cross.8Var.300Size.4Fac.0.00Fcor | 0.02 | 0.02 | 0.02 | 0.01 | 0.06 | 0.83 | 0.50 | 0.50 | 0.50 | 0.00 | 0.00 | 0.00 | 99.71 | 99.71 | 99.68 | 99.79 | 99.96 | 100.00 |
| Cont.0.40Load.125Cross.8Var.300Size.4Fac.0.50Fcor | 0.05 | 0.05 | 0.03 | 0.04 | 0.12 | 0.52 | 10.00 | 10.00 | 8.75 | 11.50 | 7.75 | 0.00 | 97.96 | 97.96 | 97.93 | 97.68 | 99.68 | 100.00 |
| Cont.0.40Load.125Cross.8Var.300Size.4Fac.0.70Fcor | 0.07 | 0.07 | 0.05 | 0.06 | 0.16 | 0.04 | 21.75 | 21.75 | 21.50 | 28.00 | 23.50 | 0.00 | 95.43 | 95.43 | 95.00 | 93.68 | 98.71 | 100.00 |
| Cont.0.40Load.125Cross.8Var.500Size.4Fac.0.00Fcor | 0.70 | 0.70 | 0.41 | 0.58 | 0.77 | 0.84 | 43.75 | 42.50 | 25.25 | 28.75 | 3.25 | 0.00 | 93.71 | 94.00 | 93.29 | 89.79 | 100.00 | 100.00 |
| Cont.0.40Load.125Cross.8Var.500Size.4Fac.0.50Fcor | 0.33 | 0.33 | 0.16 | 0.31 | 0.71 | 0.74 | 63.75 | 63.75 | 46.25 | 68.00 | 35.50 | 0.00 | 84.79 | 84.79 | 83.89 | 82.00 | 99.36 | 100.00 |
| Cont.0.40Load.125Cross.8Var.500Size.4Fac.0.70Fcor | 0.15 | 0.15 | 0.10 | 0.14 | 0.41 | 0.11 | 52.25 | 52.25 | 47.00 | 62.00 | 39.00 | 0.00 | 84.39 | 84.39 | 85.29 | 81.00 | 98.32 | 100.00 |
| Cont.0.40Load.125Cross.8Var.1000Size.4Fac.0.00Fcor | 0.92 | 0.92 | 0.48 | 0.71 | 0.84 | 0.84 | 68.50 | 68.00 | 37.00 | 40.75 | 4.25 | 0.00 | 98.32 | 98.36 | 95.82 | 91.64 | 100.00 | 100.00 |
| Cont.0.40Load.125Cross.8Var.1000Size.4Fac.0.50Fcor | 0.41 | 0.41 | 0.20 | 0.39 | 0.85 | 0.84 | 63.50 | 63.50 | 48.00 | 65.50 | 55.00 | 0.00 | 89.25 | 89.25 | 89.25 | 87.46 | 99.64 | 100.00 |
| Cont.0.40Load.125Cross.8Var.1000Size.4Fac.0.70Fcor | 0.16 | 0.16 | 0.11 | 0.15 | 0.56 | 0.36 | 50.00 | 50.00 | 45.25 | 55.50 | 46.50 | 0.00 | 91.21 | 91.21 | 89.96 | 90.25 | 98.71 | 100.00 |
| Cont.0.40Load.Cross250.4Var.300Size.2Fac.0.00Fcor | 0.17 | 0.17 | 0.12 | 0.11 | 0.28 | 0.50 | 11.00 | 9.50 | 10.00 | 8.50 | 16.50 | 0.00 | 97.00 | 97.33 | 95.83 | 97.83 | 96.67 | 100.00 |
| Cont.0.40Load.Cross250.4Var.300Size.2Fac.0.50Fcor | 0.20 | 0.20 | 0.14 | 0.12 | 0.35 | 0.08 | 24.50 | 24.50 | 27.00 | 25.50 | 43.50 | 0.00 | 92.33 | 92.33 | 90.83 | 92.33 | 92.83 | 100.00 |
| Cont.0.40Load.Cross250.4Var.300Size.2Fac.0.70Fcor | 0.15 | 0.15 | 0.10 | 0.11 | 0.18 | 0.01 | 25.00 | 25.00 | 28.00 | 27.00 | 27.50 | 0.00 | 93.67 | 93.67 | 90.83 | 93.17 | 95.67 | 100.00 |
| Cont.0.40Load.Cross250.4Var.500Size.2Fac.0.00Fcor | 0.41 | 0.41 | 0.31 | 0.28 | 0.53 | 0.51 | 32.50 | 32.50 | 30.00 | 26.00 | 31.50 | 0.00 | 95.17 | 95.17 | 93.50 | 95.50 | 96.00 | 100.00 |
| Cont.0.40Load.Cross250.4Var.500Size.2Fac.0.50Fcor | 0.39 | 0.39 | 0.27 | 0.25 | 0.56 | 0.15 | 39.50 | 39.50 | 38.00 | 34.00 | 63.00 | 0.00 | 95.50 | 95.50 | 94.00 | 96.67 | 94.00 | 100.00 |
| Cont.0.40Load.Cross250.4Var.500Size.2Fac.0.70Fcor | 0.18 | 0.18 | 0.13 | 0.11 | 0.19 | 0.00 | 22.50 | 22.50 | 24.50 | 21.00 | 25.00 | 0.00 | 95.67 | 95.67 | 94.33 | 96.33 | 97.17 | 100.00 |
| Cont.0.40Load.Cross250.4Var.1000Size.2Fac.0.00Fcor | 0.65 | 0.66 | 0.49 | 0.45 | 0.68 | 0.53 | 55.00 | 55.00 | 42.00 | 40.50 | 49.50 | 0.00 | 96.33 | 96.33 | 93.00 | 94.00 | 97.50 | 100.00 |
| Cont.0.40Load.Cross250.4Var.1000Size.2Fac.0.50Fcor | 0.35 | 0.35 | 0.26 | 0.22 | 0.52 | 0.19 | 39.00 | 39.00 | 38.00 | 31.50 | 69.50 | 0.00 | 94.33 | 94.33 | 93.67 | 95.83 | 90.50 | 100.00 |
| Cont.0.40Load.Cross250.4Var.1000Size.2Fac.0.70Fcor | 0.13 | 0.13 | 0.08 | 0.08 | 0.10 | 0.01 | 14.00 | 14.00 | 16.00 | 14.00 | 15.00 | 0.00 | 97.00 | 97.00 | 97.17 | 98.33 | 97.50 | 100.00 |
| Cont.0.40Load.Cross250.4Var.300Size.4Fac.0.00Fcor | 0.07 | 0.07 | 0.06 | 0.06 | 0.15 | 0.64 | 3.25 | 3.25 | 3.25 | 2.75 | 1.25 | 0.00 | 98.67 | 98.67 | 98.75 | 98.83 | 99.50 | 100.00 |
| Cont.0.40Load.Cross250.4Var.300Size.4Fac.0.50Fcor | 0.19 | 0.19 | 0.13 | 0.17 | 0.28 | 0.12 | 26.00 | 26.25 | 22.75 | 28.00 | 29.50 | 0.00 | 94.17 | 94.08 | 94.42 | 93.25 | 96.83 | 100.00 |
| Cont.0.40Load.Cross250.4Var.300Size.4Fac.0.70Fcor | 0.14 | 0.14 | 0.09 | 0.12 | 0.20 | 0.01 | 32.75 | 32.75 | 30.00 | 36.25 | 40.50 | 0.00 | 92.08 | 92.08 | 89.42 | 91.08 | 95.83 | 100.00 |
| Cont.0.40Load.Cross250.4Var.500Size.4Fac.0.00Fcor | 0.41 | 0.41 | 0.33 | 0.39 | 0.52 | 0.67 | 24.25 | 24.25 | 18.25 | 19.75 | 6.75 | 0.00 | 96.08 | 96.08 | 96.25 | 95.67 | 99.83 | 100.00 |
| Cont.0.40Load.Cross250.4Var.500Size.4Fac.0.50Fcor | 0.28 | 0.28 | 0.19 | 0.26 | 0.47 | 0.22 | 35.50 | 35.50 | 37.25 | 39.50 | 45.25 | 0.00 | 93.00 | 93.00 | 91.33 | 93.17 | 97.17 | 100.00 |
| Cont.0.40Load.Cross250.4Var.500Size.4Fac.0.70Fcor | 0.17 | 0.17 | 0.10 | 0.14 | 0.27 | 0.01 | 25.25 | 25.25 | 33.75 | 34.75 | 49.25 | 0.00 | 95.08 | 95.08 | 91.67 | 93.67 | 96.58 | 100.00 |
| Cont.0.40Load.Cross250.4Var.1000Size.4Fac.0.00Fcor | 0.73 | 0.73 | 0.56 | 0.72 | 0.72 | 0.69 | 54.25 | 53.75 | 40.50 | 50.50 | 16.50 | 0.00 | 95.67 | 96.08 | 94.75 | 95.25 | 99.83 | 100.00 |
| Cont.0.40Load.Cross250.4Var.1000Size.4Fac.0.50Fcor | 0.30 | 0.30 | 0.24 | 0.29 | 0.54 | 0.37 | 43.25 | 43.25 | 45.00 | 46.50 | 56.00 | 0.00 | 93.17 | 93.17 | 91.50 | 93.75 | 97.50 | 100.00 |
| Cont.0.40Load.Cross250.4Var.1000Size.4Fac.0.70Fcor | 0.11 | 0.11 | 0.09 | 0.10 | 0.29 | 0.01 | 21.00 | 21.00 | 25.75 | 25.50 | 50.75 | 0.00 | 95.25 | 95.25 | 94.67 | 95.25 | 96.58 | 100.00 |
| Cont.0.40Load.Cross250.8Var.300Size.2Fac.0.00Fcor | 0.22 | 0.22 | 0.16 | 0.15 | 0.32 | 0.75 | 9.50 | 9.50 | 9.00 | 9.50 | 2.50 | 0.00 | 96.93 | 97.07 | 94.36 | 93.71 | 99.50 | 100.00 |
| Cont.0.40Load.Cross250.8Var.300Size.2Fac.0.50Fcor | 0.23 | 0.23 | 0.16 | 0.17 | 0.50 | 0.54 | 39.50 | 40.00 | 35.50 | 44.50 | 44.00 | 0.00 | 90.00 | 89.86 | 88.07 | 88.57 | 97.43 | 100.00 |
| Cont.0.40Load.Cross250.8Var.300Size.2Fac.0.70Fcor | 0.15 | 0.15 | 0.11 | 0.13 | 0.38 | 0.09 | 44.50 | 44.50 | 42.50 | 59.50 | 52.00 | 0.00 | 86.50 | 86.50 | 83.21 | 84.29 | 95.43 | 100.00 |
| Cont.0.40Load.Cross250.8Var.500Size.2Fac.0.00Fcor | 0.69 | 0.69 | 0.42 | 0.37 | 0.68 | 0.75 | 47.50 | 47.00 | 24.00 | 27.00 | 8.50 | 0.00 | 94.50 | 94.43 | 89.00 | 86.43 | 99.36 | 100.00 |
| Cont.0.40Load.Cross250.8Var.500Size.2Fac.0.50Fcor | 0.45 | 0.45 | 0.25 | 0.28 | 0.85 | 0.70 | 63.00 | 63.00 | 52.50 | 58.50 | 76.50 | 0.00 | 88.79 | 88.79 | 85.93 | 87.71 | 98.86 | 100.00 |
| Cont.0.40Load.Cross250.8Var.500Size.2Fac.0.70Fcor | 0.23 | 0.23 | 0.13 | 0.18 | 0.66 | 0.19 | 52.00 | 52.00 | 52.50 | 60.00 | 80.50 | 0.00 | 87.71 | 87.71 | 84.21 | 85.64 | 96.64 | 100.00 |
| Cont.0.40Load.Cross250.8Var.1000Size.2Fac.0.00Fcor | 0.88 | 0.88 | 0.53 | 0.41 | 0.74 | 0.75 | 63.00 | 63.00 | 23.50 | 17.00 | 9.00 | 0.00 | 98.86 | 98.86 | 90.00 | 87.50 | 99.86 | 100.00 |
| Cont.0.40Load.Cross250.8Var.1000Size.2Fac.0.50Fcor | 0.70 | 0.70 | 0.41 | 0.40 | 0.96 | 0.75 | 90.50 | 90.50 | 63.00 | 64.50 | 95.00 | 0.00 | 90.71 | 90.71 | 87.79 | 86.93 | 99.00 | 100.00 |
| Cont.0.40Load.Cross250.8Var.1000Size.2Fac.0.70Fcor | 0.31 | 0.31 | 0.21 | 0.23 | 0.85 | 0.44 | 58.00 | 58.00 | 52.50 | 56.50 | 97.00 | 0.00 | 88.07 | 88.07 | 88.50 | 88.86 | 96.71 | 100.00 |
| Cont.0.40Load.Cross250.8Var.300Size.4Fac.0.00Fcor | 0.01 | 0.01 | 0.01 | 0.01 | 0.02 | 0.68 | 0.12 | 0.12 | 0.12 | 0.12 | 0.00 | 0.00 | 99.88 | 99.88 | 99.88 | 99.88 | 99.96 | 100.00 |
| Cont.0.40Load.Cross250.8Var.300Size.4Fac.0.50Fcor | 0.11 | 0.11 | 0.09 | 0.11 | 0.23 | 0.40 | 22.62 | 22.62 | 20.00 | 23.38 | 12.75 | 0.00 | 95.92 | 95.92 | 95.17 | 95.46 | 99.71 | 100.00 |
| Cont.0.40Load.Cross250.8Var.300Size.4Fac.0.70Fcor | 0.14 | 0.14 | 0.11 | 0.13 | 0.23 | 0.03 | 35.12 | 35.50 | 31.50 | 39.25 | 22.62 | 0.00 | 92.58 | 92.58 | 92.29 | 91.29 | 99.21 | 100.00 |
| Cont.0.40Load.Cross250.8Var.500Size.4Fac.0.00Fcor | 0.41 | 0.41 | 0.27 | 0.34 | 0.46 | 0.69 | 27.75 | 27.38 | 21.12 | 20.00 | 1.62 | 0.00 | 92.92 | 93.38 | 93.67 | 91.21 | 99.83 | 100.00 |
| Cont.0.40Load.Cross250.8Var.500Size.4Fac.0.50Fcor | 0.28 | 0.28 | 0.19 | 0.28 | 0.55 | 0.58 | 50.62 | 50.62 | 42.12 | 55.50 | 20.75 | 0.00 | 87.12 | 87.12 | 86.46 | 85.29 | 99.42 | 100.00 |
| Cont.0.40Load.Cross250.8Var.500Size.4Fac.0.70Fcor | 0.16 | 0.16 | 0.12 | 0.16 | 0.34 | 0.03 | 42.38 | 42.38 | 42.00 | 52.50 | 26.62 | 0.00 | 89.71 | 89.71 | 88.62 | 87.33 | 99.50 | 100.00 |
| Cont.0.40Load.Cross250.8Var.1000Size.4Fac.0.00Fcor | 0.83 | 0.83 | 0.38 | 0.57 | 0.68 | 0.69 | 65.00 | 64.25 | 34.25 | 33.00 | 3.38 | 0.00 | 96.88 | 97.00 | 95.12 | 90.04 | 100.00 | 100.00 |
| Cont.0.40Load.Cross250.8Var.1000Size.4Fac.0.50Fcor | 0.36 | 0.36 | 0.21 | 0.33 | 0.65 | 0.68 | 54.88 | 54.88 | 44.00 | 56.50 | 30.12 | 0.00 | 90.67 | 90.67 | 90.79 | 89.83 | 99.54 | 100.00 |
| Cont.0.40Load.Cross250.8Var.1000Size.4Fac.0.70Fcor | 0.16 | 0.16 | 0.13 | 0.15 | 0.51 | 0.20 | 37.62 | 37.62 | 33.50 | 39.38 | 30.62 | 0.00 | 92.92 | 92.92 | 92.96 | 93.08 | 99.46 | 100.00 |
| Cont.0.55Load.000Cross.4Var.300Size.2Fac.0.00Fcor | 0.99 | 0.99 | 0.83 | 0.83 | 1.00 | 1.00 | - | - | - | - | - | - | - | - | - | - | - | - |
| Cont.0.55Load.000Cross.4Var.300Size.2Fac.0.50Fcor | 0.90 | 0.90 | 0.64 | 0.75 | 0.78 | 0.92 | - | - | - | - | - | - | - | - | - | - | - | - |
| Cont.0.55Load.000Cross.4Var.300Size.2Fac.0.70Fcor | 0.67 | 0.67 | 0.52 | 0.58 | 0.36 | 0.10 | - | - | - | - | - | - | - | - | - | - | - | - |
| Cont.0.55Load.000Cross.4Var.500Size.2Fac.0.00Fcor | 1.00 | 1.00 | 0.87 | 0.89 | 1.00 | 1.00 | - | - | - | - | - | - | - | - | - | - | - | - |
| Cont.0.55Load.000Cross.4Var.500Size.2Fac.0.50Fcor | 0.93 | 0.93 | 0.65 | 0.77 | 0.80 | 0.95 | - | - | - | - | - | - | - | - | - | - | - | - |
| Cont.0.55Load.000Cross.4Var.500Size.2Fac.0.70Fcor | 0.72 | 0.72 | 0.59 | 0.69 | 0.29 | 0.11 | - | - | - | - | - | - | - | - | - | - | - | - |
| Cont.0.55Load.000Cross.4Var.1000Size.2Fac.0.00Fcor | 0.99 | 1.00 | 0.84 | 0.90 | 1.00 | 1.00 | - | - | - | - | - | - | - | - | - | - | - | - |
| Cont.0.55Load.000Cross.4Var.1000Size.2Fac.0.50Fcor | 0.99 | 0.99 | 0.70 | 0.87 | 0.84 | 1.00 | - | - | - | - | - | - | - | - | - | - | - | - |
| Cont.0.55Load.000Cross.4Var.1000Size.2Fac.0.70Fcor | 0.78 | 0.78 | 0.67 | 0.76 | 0.36 | 0.25 | - | - | - | - | - | - | - | - | - | - | - | - |
| Cont.0.55Load.000Cross.4Var.300Size.4Fac.0.00Fcor | 0.99 | 0.99 | 0.74 | 0.91 | 1.00 | 1.00 | - | - | - | - | - | - | - | - | - | - | - | - |
| Cont.0.55Load.000Cross.4Var.300Size.4Fac.0.50Fcor | 0.71 | 0.71 | 0.32 | 0.69 | 0.86 | 0.63 | - | - | - | - | - | - | - | - | - | - | - | - |
| Cont.0.55Load.000Cross.4Var.300Size.4Fac.0.70Fcor | 0.43 | 0.43 | 0.13 | 0.37 | 0.43 | 0.02 | - | - | - | - | - | - | - | - | - | - | - | - |
| Cont.0.55Load.000Cross.4Var.500Size.4Fac.0.00Fcor | 1.00 | 1.00 | 0.78 | 0.94 | 1.00 | 1.00 | - | - | - | - | - | - | - | - | - | - | - | - |
| Cont.0.55Load.000Cross.4Var.500Size.4Fac.0.50Fcor | 0.83 | 0.83 | 0.31 | 0.79 | 0.93 | 0.85 | - | - | - | - | - | - | - | - | - | - | - | - |
| Cont.0.55Load.000Cross.4Var.500Size.4Fac.0.70Fcor | 0.51 | 0.51 | 0.21 | 0.49 | 0.64 | 0.05 | - | - | - | - | - | - | - | - | - | - | - | - |
| Cont.0.55Load.000Cross.4Var.1000Size.4Fac.0.00Fcor | 0.99 | 1.00 | 0.79 | 0.94 | 1.00 | 1.00 | - | - | - | - | - | - | - | - | - | - | - | - |
| Cont.0.55Load.000Cross.4Var.1000Size.4Fac.0.50Fcor | 0.93 | 0.93 | 0.25 | 0.82 | 0.96 | 0.99 | - | - | - | - | - | - | - | - | - | - | - | - |
| Cont.0.55Load.000Cross.4Var.1000Size.4Fac.0.70Fcor | 0.61 | 0.61 | 0.29 | 0.60 | 0.79 | 0.06 | - | - | - | - | - | - | - | - | - | - | - | - |
| Cont.0.55Load.000Cross.8Var.300Size.2Fac.0.00Fcor | 1.00 | 1.00 | 0.63 | 0.55 | 1.00 | 1.00 | - | - | - | - | - | - | - | - | - | - | - | - |
| Cont.0.55Load.000Cross.8Var.300Size.2Fac.0.50Fcor | 0.93 | 0.93 | 0.55 | 0.54 | 0.99 | 1.00 | - | - | - | - | - | - | - | - | - | - | - | - |
| Cont.0.55Load.000Cross.8Var.300Size.2Fac.0.70Fcor | 0.72 | 0.72 | 0.39 | 0.46 | 0.90 | 0.85 | - | - | - | - | - | - | - | - | - | - | - | - |
| Cont.0.55Load.000Cross.8Var.500Size.2Fac.0.00Fcor | 0.99 | 1.00 | 0.63 | 0.56 | 1.00 | 1.00 | - | - | - | - | - | - | - | - | - | - | - | - |
| Cont.0.55Load.000Cross.8Var.500Size.2Fac.0.50Fcor | 0.97 | 0.97 | 0.56 | 0.57 | 1.00 | 1.00 | - | - | - | - | - | - | - | - | - | - | - | - |
| Cont.0.55Load.000Cross.8Var.500Size.2Fac.0.70Fcor | 0.82 | 0.82 | 0.49 | 0.51 | 0.94 | 0.98 | - | - | - | - | - | - | - | - | - | - | - | - |
| Cont.0.55Load.000Cross.8Var.1000Size.2Fac.0.00Fcor | 0.99 | 1.00 | 0.68 | 0.59 | 1.00 | 1.00 | - | - | - | - | - | - | - | - | - | - | - | - |
| Cont.0.55Load.000Cross.8Var.1000Size.2Fac.0.50Fcor | 0.95 | 0.95 | 0.58 | 0.59 | 1.00 | 1.00 | - | - | - | - | - | - | - | - | - | - | - | - |
| Cont.0.55Load.000Cross.8Var.1000Size.2Fac.0.70Fcor | 0.81 | 0.81 | 0.56 | 0.55 | 0.98 | 1.00 | - | - | - | - | - | - | - | - | - | - | - | - |
| Cont.0.55Load.000Cross.8Var.300Size.4Fac.0.00Fcor | 0.99 | 1.00 | 0.57 | 0.78 | 1.00 | 1.00 | - | - | - | - | - | - | - | - | - | - | - | - |
| Cont.0.55Load.000Cross.8Var.300Size.4Fac.0.50Fcor | 0.79 | 0.79 | 0.31 | 0.67 | 1.00 | 1.00 | - | - | - | - | - | - | - | - | - | - | - | - |
| Cont.0.55Load.000Cross.8Var.300Size.4Fac.0.70Fcor | 0.58 | 0.58 | 0.17 | 0.51 | 0.92 | 0.46 | - | - | - | - | - | - | - | - | - | - | - | - |
| Cont.0.55Load.000Cross.8Var.500Size.4Fac.0.00Fcor | 1.00 | 1.00 | 0.62 | 0.81 | 1.00 | 1.00 | - | - | - | - | - | - | - | - | - | - | - | - |
| Cont.0.55Load.000Cross.8Var.500Size.4Fac.0.50Fcor | 0.88 | 0.88 | 0.36 | 0.72 | 1.00 | 1.00 | - | - | - | - | - | - | - | - | - | - | - | - |
| Cont.0.55Load.000Cross.8Var.500Size.4Fac.0.70Fcor | 0.69 | 0.69 | 0.20 | 0.58 | 0.97 | 0.73 | - | - | - | - | - | - | - | - | - | - | - | - |
| Cont.0.55Load.000Cross.8Var.1000Size.4Fac.0.00Fcor | 0.99 | 1.00 | 0.61 | 0.78 | 1.00 | 1.00 | - | - | - | - | - | - | - | - | - | - | - | - |
| Cont.0.55Load.000Cross.8Var.1000Size.4Fac.0.50Fcor | 0.97 | 0.97 | 0.29 | 0.65 | 1.00 | 1.00 | - | - | - | - | - | - | - | - | - | - | - | - |
| Cont.0.55Load.000Cross.8Var.1000Size.4Fac.0.70Fcor | 0.81 | 0.81 | 0.21 | 0.63 | 1.00 | 0.99 | - | - | - | - | - | - | - | - | - | - | - | - |
| Cont.0.55Load.125Cross.4Var.300Size.2Fac.0.00Fcor | 0.88 | 0.88 | 0.78 | 0.54 | 0.93 | 0.77 | 61.00 | 60.00 | 63.00 | 22.00 | 83.00 | 6.00 | 99.57 | 99.57 | 95.86 | 95.00 | 96.86 | 100.00 |
| Cont.0.55Load.125Cross.4Var.300Size.2Fac.0.50Fcor | 0.78 | 0.78 | 0.66 | 0.59 | 0.69 | 0.51 | 85.00 | 85.00 | 81.00 | 62.00 | 94.00 | 7.00 | 93.71 | 93.71 | 91.29 | 93.57 | 88.71 | 100.00 |
| Cont.0.55Load.125Cross.4Var.300Size.2Fac.0.70Fcor | 0.50 | 0.50 | 0.40 | 0.36 | 0.18 | 0.03 | 65.00 | 65.00 | 69.00 | 56.00 | 36.00 | 1.00 | 92.00 | 92.00 | 89.57 | 91.86 | 92.29 | 100.00 |
| Cont.0.55Load.125Cross.4Var.500Size.2Fac.0.00Fcor | 0.88 | 0.88 | 0.80 | 0.60 | 0.95 | 0.76 | 61.00 | 61.00 | 70.00 | 22.00 | 93.00 | 4.00 | 99.57 | 99.57 | 95.71 | 95.00 | 98.43 | 100.00 |
| Cont.0.55Load.125Cross.4Var.500Size.2Fac.0.50Fcor | 0.85 | 0.85 | 0.73 | 0.60 | 0.77 | 0.64 | 90.00 | 90.00 | 88.00 | 64.00 | 97.00 | 10.00 | 95.00 | 95.00 | 92.43 | 95.86 | 90.86 | 100.00 |
| Cont.0.55Load.125Cross.4Var.500Size.2Fac.0.70Fcor | 0.57 | 0.57 | 0.52 | 0.45 | 0.15 | 0.01 | 77.00 | 77.00 | 78.00 | 62.00 | 31.00 | 0.00 | 91.14 | 91.14 | 91.00 | 94.29 | 92.43 | 100.00 |
| Cont.0.55Load.125Cross.4Var.1000Size.2Fac.0.00Fcor | 0.91 | 0.91 | 0.81 | 0.60 | 1.00 | 0.77 | 66.00 | 67.00 | 70.00 | 21.00 | 100.00 | 5.00 | 100.00 | 100.00 | 97.57 | 97.71 | 99.86 | 100.00 |
| Cont.0.55Load.125Cross.4Var.1000Size.2Fac.0.50Fcor | 0.86 | 0.86 | 0.75 | 0.67 | 0.78 | 0.75 | 93.00 | 93.00 | 92.00 | 79.00 | 100.00 | 9.00 | 95.86 | 95.86 | 95.00 | 96.00 | 90.29 | 100.00 |
| Cont.0.55Load.125Cross.4Var.1000Size.2Fac.0.70Fcor | 0.66 | 0.66 | 0.57 | 0.51 | 0.14 | 0.04 | 76.00 | 76.00 | 81.00 | 67.00 | 22.00 | 1.00 | 93.86 | 93.86 | 94.86 | 97.29 | 96.43 | 100.00 |
| Cont.0.55Load.125Cross.4Var.300Size.4Fac.0.00Fcor | 0.90 | 0.91 | 0.68 | 0.79 | 0.89 | 0.85 | 61.50 | 61.50 | 29.00 | 40.50 | 39.50 | 8.00 | 98.79 | 98.71 | 98.00 | 96.64 | 99.79 | 100.00 |
| Cont.0.55Load.125Cross.4Var.300Size.4Fac.0.50Fcor | 0.61 | 0.61 | 0.33 | 0.61 | 0.76 | 0.50 | 63.50 | 63.50 | 45.00 | 72.50 | 80.00 | 2.50 | 91.14 | 91.14 | 87.64 | 90.00 | 95.71 | 100.00 |
| Cont.0.55Load.125Cross.4Var.300Size.4Fac.0.70Fcor | 0.35 | 0.35 | 0.19 | 0.33 | 0.45 | 0.01 | 49.00 | 49.00 | 49.50 | 59.50 | 76.00 | 0.00 | 87.79 | 87.93 | 83.93 | 86.79 | 91.21 | 100.00 |
| Cont.0.55Load.125Cross.4Var.500Size.4Fac.0.00Fcor | 0.94 | 0.94 | 0.67 | 0.80 | 0.95 | 0.84 | 68.50 | 68.50 | 39.00 | 39.00 | 69.50 | 7.00 | 99.36 | 99.57 | 97.57 | 97.64 | 100.00 | 100.00 |
| Cont.0.55Load.125Cross.4Var.500Size.4Fac.0.50Fcor | 0.78 | 0.78 | 0.37 | 0.74 | 0.85 | 0.66 | 76.00 | 76.00 | 54.00 | 79.00 | 87.50 | 5.00 | 95.00 | 95.00 | 87.36 | 93.71 | 96.86 | 100.00 |
| Cont.0.55Load.125Cross.4Var.500Size.4Fac.0.70Fcor | 0.44 | 0.44 | 0.24 | 0.39 | 0.56 | 0.02 | 58.50 | 58.50 | 49.50 | 70.50 | 80.00 | 0.00 | 90.29 | 90.29 | 84.64 | 85.93 | 92.50 | 100.00 |
| Cont.0.55Load.125Cross.4Var.1000Size.4Fac.0.00Fcor | 0.95 | 0.95 | 0.58 | 0.80 | 0.99 | 0.84 | 73.00 | 74.00 | 33.00 | 42.00 | 93.50 | 5.50 | 99.79 | 99.93 | 95.36 | 98.64 | 100.00 | 100.00 |
| Cont.0.55Load.125Cross.4Var.1000Size.4Fac.0.50Fcor | 0.82 | 0.82 | 0.29 | 0.78 | 0.89 | 0.79 | 84.50 | 84.50 | 48.00 | 80.00 | 91.00 | 3.00 | 95.50 | 95.50 | 85.50 | 94.29 | 97.79 | 100.00 |
| Cont.0.55Load.125Cross.4Var.1000Size.4Fac.0.70Fcor | 0.51 | 0.51 | 0.32 | 0.49 | 0.66 | 0.07 | 62.50 | 62.50 | 50.50 | 68.50 | 85.00 | 0.00 | 91.36 | 91.36 | 86.71 | 89.93 | 94.14 | 100.00 |
| Cont.0.55Load.125Cross.8Var.300Size.2Fac.0.00Fcor | 0.85 | 0.84 | 0.51 | 0.40 | 0.77 | 0.77 | 46.50 | 45.50 | 13.00 | 13.50 | 23.50 | 7.50 | 99.57 | 99.57 | 90.57 | 83.36 | 99.00 | 100.00 |
| Cont.0.55Load.125Cross.8Var.300Size.2Fac.0.50Fcor | 0.81 | 0.81 | 0.36 | 0.40 | 0.96 | 0.78 | 86.00 | 86.00 | 51.50 | 61.50 | 92.50 | 10.50 | 94.14 | 94.14 | 83.07 | 82.57 | 99.21 | 100.00 |
| Cont.0.55Load.125Cross.8Var.300Size.2Fac.0.70Fcor | 0.51 | 0.51 | 0.23 | 0.31 | 0.89 | 0.42 | 76.50 | 76.50 | 52.00 | 69.00 | 96.00 | 4.50 | 86.29 | 86.29 | 81.07 | 83.86 | 96.71 | 100.00 |
| Cont.0.55Load.125Cross.8Var.500Size.2Fac.0.00Fcor | 0.86 | 0.86 | 0.53 | 0.37 | 0.76 | 0.77 | 51.00 | 49.50 | 14.50 | 9.50 | 29.50 | 6.00 | 99.86 | 99.86 | 94.64 | 90.93 | 98.57 | 100.00 |
| Cont.0.55Load.125Cross.8Var.500Size.2Fac.0.50Fcor | 0.94 | 0.94 | 0.44 | 0.44 | 0.99 | 0.77 | 94.00 | 94.00 | 54.50 | 59.50 | 97.50 | 6.00 | 97.57 | 97.57 | 84.57 | 81.50 | 99.79 | 100.00 |
| Cont.0.55Load.125Cross.8Var.500Size.2Fac.0.70Fcor | 0.70 | 0.70 | 0.34 | 0.43 | 0.96 | 0.64 | 96.00 | 96.00 | 69.00 | 79.00 | 99.00 | 5.00 | 86.64 | 86.64 | 83.79 | 87.50 | 98.43 | 100.00 |
| Cont.0.55Load.125Cross.8Var.1000Size.2Fac.0.00Fcor | 0.91 | 0.89 | 0.49 | 0.40 | 0.83 | 0.76 | 70.50 | 64.50 | 24.50 | 10.00 | 49.00 | 3.00 | 100.00 | 100.00 | 89.43 | 88.43 | 99.43 | 100.00 |
| Cont.0.55Load.125Cross.8Var.1000Size.2Fac.0.50Fcor | 0.96 | 0.96 | 0.47 | 0.47 | 1.00 | 0.76 | 98.50 | 98.50 | 54.00 | 54.50 | 100.00 | 4.00 | 98.36 | 98.36 | 83.79 | 88.14 | 99.86 | 100.00 |
| Cont.0.55Load.125Cross.8Var.1000Size.2Fac.0.70Fcor | 0.73 | 0.73 | 0.39 | 0.48 | 0.98 | 0.76 | 95.00 | 95.00 | 72.50 | 79.50 | 100.00 | 6.00 | 89.29 | 89.29 | 86.86 | 91.50 | 98.86 | 100.00 |
| Cont.0.55Load.125Cross.8Var.300Size.4Fac.0.00Fcor | 0.93 | 0.93 | 0.51 | 0.63 | 0.86 | 0.86 | 64.00 | 63.00 | 33.75 | 14.50 | 14.00 | 12.50 | 99.43 | 99.57 | 96.04 | 87.82 | 100.00 | 100.00 |
| Cont.0.55Load.125Cross.8Var.300Size.4Fac.0.50Fcor | 0.74 | 0.74 | 0.31 | 0.60 | 0.92 | 0.85 | 81.75 | 81.75 | 55.75 | 69.00 | 74.25 | 10.75 | 89.89 | 89.89 | 85.61 | 83.64 | 99.61 | 100.00 |
| Cont.0.55Load.125Cross.8Var.300Size.4Fac.0.70Fcor | 0.45 | 0.45 | 0.19 | 0.42 | 0.80 | 0.26 | 73.25 | 72.75 | 53.25 | 75.75 | 62.75 | 2.25 | 80.71 | 80.93 | 82.43 | 79.82 | 99.43 | 100.00 |
| Cont.0.55Load.125Cross.8Var.500Size.4Fac.0.00Fcor | 0.93 | 0.93 | 0.54 | 0.66 | 0.87 | 0.86 | 57.75 | 57.75 | 36.75 | 11.75 | 21.50 | 10.50 | 99.93 | 100.00 | 96.32 | 91.46 | 100.00 | 100.00 |
| Cont.0.55Load.125Cross.8Var.500Size.4Fac.0.50Fcor | 0.89 | 0.89 | 0.37 | 0.68 | 0.97 | 0.86 | 93.25 | 93.25 | 58.75 | 74.75 | 91.50 | 11.00 | 95.32 | 95.32 | 90.71 | 85.89 | 100.00 | 100.00 |
| Cont.0.55Load.125Cross.8Var.500Size.4Fac.0.70Fcor | 0.61 | 0.61 | 0.24 | 0.54 | 0.91 | 0.54 | 83.00 | 83.00 | 57.00 | 80.75 | 82.50 | 4.00 | 86.29 | 86.29 | 84.04 | 83.39 | 99.86 | 100.00 |
| Cont.0.55Load.125Cross.8Var.1000Size.4Fac.0.00Fcor | 0.97 | 0.97 | 0.51 | 0.65 | 0.92 | 0.85 | 81.75 | 82.00 | 41.00 | 27.75 | 50.00 | 9.75 | 99.96 | 100.00 | 94.93 | 93.68 | 100.00 | 100.00 |
| Cont.0.55Load.125Cross.8Var.1000Size.4Fac.0.50Fcor | 0.96 | 0.96 | 0.38 | 0.67 | 0.98 | 0.85 | 97.00 | 97.00 | 62.50 | 69.00 | 99.25 | 8.50 | 98.07 | 98.07 | 87.54 | 88.14 | 99.93 | 100.00 |
| Cont.0.55Load.125Cross.8Var.1000Size.4Fac.0.70Fcor | 0.70 | 0.70 | 0.30 | 0.62 | 0.97 | 0.79 | 92.25 | 92.25 | 64.00 | 82.75 | 95.00 | 2.00 | 85.96 | 85.96 | 84.25 | 87.18 | 99.79 | 100.00 |
| Cont.0.55Load.Cross250.4Var.300Size.2Fac.0.00Fcor | 0.66 | 0.65 | 0.51 | 0.42 | 0.63 | 0.54 | 51.50 | 49.50 | 43.00 | 36.00 | 62.50 | 3.50 | 96.83 | 97.00 | 89.50 | 92.33 | 89.00 | 100.00 |
| Cont.0.55Load.Cross250.4Var.300Size.2Fac.0.50Fcor | 0.68 | 0.68 | 0.54 | 0.42 | 0.48 | 0.25 | 78.50 | 78.50 | 70.00 | 47.50 | 73.00 | 4.00 | 91.83 | 91.83 | 87.33 | 94.17 | 87.83 | 100.00 |
| Cont.0.55Load.Cross250.4Var.300Size.2Fac.0.70Fcor | 0.41 | 0.41 | 0.33 | 0.27 | 0.07 | 0.01 | 49.00 | 49.00 | 48.50 | 39.00 | 9.50 | 0.00 | 93.67 | 93.67 | 91.67 | 95.33 | 98.50 | 100.00 |
| Cont.0.55Load.Cross250.4Var.500Size.2Fac.0.00Fcor | 0.77 | 0.76 | 0.51 | 0.42 | 0.68 | 0.53 | 68.50 | 67.50 | 40.00 | 26.00 | 77.00 | 0.50 | 97.17 | 97.33 | 91.17 | 94.50 | 89.33 | 100.00 |
| Cont.0.55Load.Cross250.4Var.500Size.2Fac.0.50Fcor | 0.83 | 0.83 | 0.62 | 0.55 | 0.55 | 0.34 | 86.00 | 86.00 | 77.00 | 62.00 | 77.50 | 1.00 | 96.50 | 96.50 | 91.00 | 96.00 | 89.67 | 100.00 |
| Cont.0.55Load.Cross250.4Var.500Size.2Fac.0.70Fcor | 0.50 | 0.50 | 0.40 | 0.30 | 0.03 | 0.00 | 59.50 | 59.50 | 59.00 | 41.50 | 6.00 | 0.00 | 93.33 | 93.33 | 92.50 | 95.33 | 98.83 | 100.00 |
| Cont.0.55Load.Cross250.4Var.1000Size.2Fac.0.00Fcor | 0.84 | 0.84 | 0.49 | 0.39 | 0.84 | 0.53 | 75.00 | 75.00 | 36.00 | 24.50 | 94.00 | 0.00 | 99.17 | 99.17 | 92.67 | 95.17 | 93.83 | 100.00 |
| Cont.0.55Load.Cross250.4Var.1000Size.2Fac.0.50Fcor | 0.88 | 0.88 | 0.69 | 0.57 | 0.56 | 0.41 | 89.50 | 89.50 | 81.00 | 58.50 | 77.50 | 1.50 | 98.50 | 98.50 | 92.00 | 97.67 | 90.00 | 100.00 |
| Cont.0.55Load.Cross250.4Var.1000Size.2Fac.0.70Fcor | 0.53 | 0.53 | 0.43 | 0.32 | 0.01 | 0.00 | 56.00 | 56.00 | 58.00 | 39.00 | 1.00 | 0.00 | 97.33 | 97.33 | 95.83 | 98.50 | 99.83 | 100.00 |
| Cont.0.55Load.Cross250.4Var.300Size.4Fac.0.00Fcor | 0.82 | 0.82 | 0.55 | 0.73 | 0.80 | 0.72 | 59.75 | 59.25 | 33.00 | 45.50 | 38.00 | 10.50 | 97.58 | 97.67 | 97.75 | 95.58 | 99.75 | 100.00 |
| Cont.0.55Load.Cross250.4Var.300Size.4Fac.0.50Fcor | 0.60 | 0.60 | 0.32 | 0.57 | 0.68 | 0.40 | 63.00 | 63.25 | 56.75 | 66.50 | 78.25 | 5.00 | 91.92 | 92.00 | 90.58 | 91.50 | 95.42 | 100.00 |
| Cont.0.55Load.Cross250.4Var.300Size.4Fac.0.70Fcor | 0.34 | 0.34 | 0.22 | 0.33 | 0.37 | 0.01 | 53.00 | 53.00 | 48.75 | 55.75 | 61.25 | 0.00 | 92.08 | 92.08 | 89.25 | 91.08 | 95.67 | 100.00 |
| Cont.0.55Load.Cross250.4Var.500Size.4Fac.0.00Fcor | 0.91 | 0.91 | 0.57 | 0.74 | 0.89 | 0.71 | 72.25 | 72.25 | 46.75 | 51.50 | 65.00 | 7.00 | 99.50 | 99.58 | 97.58 | 97.33 | 100.00 | 100.00 |
| Cont.0.55Load.Cross250.4Var.500Size.4Fac.0.50Fcor | 0.72 | 0.72 | 0.37 | 0.69 | 0.78 | 0.54 | 73.25 | 73.25 | 60.75 | 72.00 | 84.50 | 6.00 | 95.08 | 95.08 | 89.42 | 92.83 | 97.92 | 100.00 |
| Cont.0.55Load.Cross250.4Var.500Size.4Fac.0.70Fcor | 0.37 | 0.37 | 0.25 | 0.34 | 0.39 | 0.01 | 55.00 | 55.00 | 53.50 | 60.00 | 62.25 | 0.00 | 93.17 | 93.17 | 90.08 | 91.58 | 97.33 | 100.00 |
| Cont.0.55Load.Cross250.4Var.1000Size.4Fac.0.00Fcor | 0.94 | 0.94 | 0.57 | 0.68 | 0.97 | 0.71 | 82.25 | 82.50 | 59.00 | 41.25 | 91.75 | 6.00 | 100.00 | 100.00 | 95.50 | 99.17 | 99.92 | 100.00 |
| Cont.0.55Load.Cross250.4Var.1000Size.4Fac.0.50Fcor | 0.81 | 0.81 | 0.36 | 0.77 | 0.84 | 0.64 | 89.25 | 89.25 | 63.25 | 83.25 | 93.25 | 4.75 | 95.25 | 95.25 | 89.67 | 93.83 | 98.33 | 100.00 |
| Cont.0.55Load.Cross250.4Var.1000Size.4Fac.0.70Fcor | 0.42 | 0.42 | 0.28 | 0.43 | 0.36 | 0.03 | 64.50 | 64.50 | 59.50 | 66.75 | 51.00 | 0.00 | 93.42 | 93.42 | 90.92 | 93.92 | 98.17 | 100.00 |
| Cont.0.55Load.Cross250.8Var.300Size.2Fac.0.00Fcor | 0.85 | 0.84 | 0.51 | 0.40 | 0.77 | 0.77 | 46.50 | 45.50 | 13.00 | 13.50 | 23.50 | 7.50 | 99.57 | 99.57 | 90.57 | 83.36 | 99.00 | 100.00 |
| Cont.0.55Load.Cross250.8Var.300Size.2Fac.0.50Fcor | 0.81 | 0.81 | 0.36 | 0.40 | 0.96 | 0.78 | 86.00 | 86.00 | 51.50 | 61.50 | 92.50 | 10.50 | 94.14 | 94.14 | 83.07 | 82.57 | 99.21 | 100.00 |
| Cont.0.55Load.Cross250.8Var.300Size.2Fac.0.70Fcor | 0.51 | 0.51 | 0.23 | 0.31 | 0.89 | 0.42 | 76.50 | 76.50 | 52.00 | 69.00 | 96.00 | 4.50 | 86.29 | 86.29 | 81.07 | 83.86 | 96.71 | 100.00 |
| Cont.0.55Load.Cross250.8Var.500Size.2Fac.0.00Fcor | 0.86 | 0.86 | 0.53 | 0.37 | 0.76 | 0.77 | 51.00 | 49.50 | 14.50 | 9.50 | 29.50 | 6.00 | 99.86 | 99.86 | 94.64 | 90.93 | 98.57 | 100.00 |
| Cont.0.55Load.Cross250.8Var.500Size.2Fac.0.50Fcor | 0.94 | 0.94 | 0.44 | 0.44 | 0.99 | 0.77 | 94.00 | 94.00 | 54.50 | 59.50 | 97.50 | 6.00 | 97.57 | 97.57 | 84.57 | 81.50 | 99.79 | 100.00 |
| Cont.0.55Load.Cross250.8Var.500Size.2Fac.0.70Fcor | 0.70 | 0.70 | 0.34 | 0.43 | 0.96 | 0.64 | 96.00 | 96.00 | 69.00 | 79.00 | 99.00 | 5.00 | 86.64 | 86.64 | 83.79 | 87.50 | 98.43 | 100.00 |
| Cont.0.55Load.Cross250.8Var.1000Size.2Fac.0.00Fcor | 0.91 | 0.89 | 0.49 | 0.40 | 0.83 | 0.76 | 70.50 | 64.50 | 24.50 | 10.00 | 49.00 | 3.00 | 100.00 | 100.00 | 89.43 | 88.43 | 99.43 | 100.00 |
| Cont.0.55Load.Cross250.8Var.1000Size.2Fac.0.50Fcor | 0.96 | 0.96 | 0.47 | 0.47 | 1.00 | 0.76 | 98.50 | 98.50 | 54.00 | 54.50 | 100.00 | 4.00 | 98.36 | 98.36 | 83.79 | 88.14 | 99.86 | 100.00 |
| Cont.0.55Load.Cross250.8Var.1000Size.2Fac.0.70Fcor | 0.73 | 0.73 | 0.39 | 0.48 | 0.98 | 0.76 | 95.00 | 95.00 | 72.50 | 79.50 | 100.00 | 6.00 | 89.29 | 89.29 | 86.86 | 91.50 | 98.86 | 100.00 |
| Cont.0.55Load.Cross250.8Var.300Size.4Fac.0.00Fcor | 0.86 | 0.86 | 0.42 | 0.49 | 0.69 | 0.72 | 66.25 | 65.75 | 33.12 | 19.00 | 7.12 | 10.12 | 97.04 | 97.00 | 96.58 | 85.38 | 99.71 | 100.00 |
| Cont.0.55Load.Cross250.8Var.300Size.4Fac.0.50Fcor | 0.64 | 0.64 | 0.29 | 0.50 | 0.78 | 0.70 | 75.62 | 75.62 | 48.38 | 63.00 | 50.38 | 9.00 | 87.83 | 87.83 | 86.04 | 84.12 | 99.25 | 100.00 |
| Cont.0.55Load.Cross250.8Var.300Size.4Fac.0.70Fcor | 0.38 | 0.38 | 0.20 | 0.35 | 0.66 | 0.17 | 64.25 | 64.25 | 44.62 | 64.00 | 40.62 | 0.75 | 85.79 | 85.79 | 86.00 | 84.42 | 99.67 | 100.00 |
| Cont.0.55Load.Cross250.8Var.500Size.4Fac.0.00Fcor | 0.90 | 0.90 | 0.45 | 0.54 | 0.69 | 0.73 | 70.12 | 70.12 | 42.00 | 21.00 | 8.75 | 12.38 | 99.75 | 99.75 | 96.88 | 89.17 | 99.71 | 100.00 |
| Cont.0.55Load.Cross250.8Var.500Size.4Fac.0.50Fcor | 0.83 | 0.83 | 0.32 | 0.59 | 0.82 | 0.72 | 88.12 | 88.12 | 50.25 | 68.62 | 62.12 | 10.88 | 94.46 | 94.46 | 89.58 | 82.12 | 99.38 | 100.00 |
| Cont.0.55Load.Cross250.8Var.500Size.4Fac.0.70Fcor | 0.52 | 0.52 | 0.24 | 0.47 | 0.77 | 0.32 | 72.00 | 72.00 | 51.75 | 74.88 | 55.25 | 2.12 | 88.38 | 88.38 | 86.50 | 83.96 | 99.71 | 100.00 |
| Cont.0.55Load.Cross250.8Var.1000Size.4Fac.0.00Fcor | 0.91 | 0.91 | 0.42 | 0.56 | 0.72 | 0.72 | 73.75 | 73.38 | 45.00 | 25.12 | 18.38 | 9.12 | 99.88 | 99.92 | 96.79 | 92.54 | 99.71 | 100.00 |
| Cont.0.55Load.Cross250.8Var.1000Size.4Fac.0.50Fcor | 0.94 | 0.94 | 0.43 | 0.69 | 0.86 | 0.72 | 97.25 | 97.25 | 56.88 | 69.88 | 77.38 | 9.88 | 97.88 | 97.88 | 91.92 | 88.21 | 99.33 | 100.00 |
| Cont.0.55Load.Cross250.8Var.1000Size.4Fac.0.70Fcor | 0.66 | 0.66 | 0.30 | 0.59 | 0.83 | 0.61 | 86.25 | 86.25 | 50.75 | 74.88 | 68.12 | 3.00 | 89.88 | 89.88 | 91.67 | 91.29 | 99.79 | 100.00 |
| Cont.0.70Load.000Cross.4Var.300Size.2Fac.0.00Fcor | 1.00 | 1.00 | 0.81 | 0.86 | 1.00 | 1.00 | - | - | - | - | - | - | - | - | - | - | - | - |
| Cont.0.70Load.000Cross.4Var.300Size.2Fac.0.50Fcor | 1.00 | 1.00 | 0.63 | 0.84 | 0.92 | 0.99 | - | - | - | - | - | - | - | - | - | - | - | - |
| Cont.0.70Load.000Cross.4Var.300Size.2Fac.0.70Fcor | 0.97 | 0.97 | 0.63 | 0.80 | 0.57 | 0.24 | - | - | - | - | - | - | - | - | - | - | - | - |
| Cont.0.70Load.000Cross.4Var.500Size.2Fac.0.00Fcor | 0.99 | 1.00 | 0.84 | 0.89 | 1.00 | 1.00 | - | - | - | - | - | - | - | - | - | - | - | - |
| Cont.0.70Load.000Cross.4Var.500Size.2Fac.0.50Fcor | 1.00 | 1.00 | 0.68 | 0.83 | 0.96 | 1.00 | - | - | - | - | - | - | - | - | - | - | - | - |
| Cont.0.70Load.000Cross.4Var.500Size.2Fac.0.70Fcor | 0.98 | 0.98 | 0.64 | 0.82 | 0.55 | 0.25 | - | - | - | - | - | - | - | - | - | - | - | - |
| Cont.0.70Load.000Cross.4Var.1000Size.2Fac.0.00Fcor | 1.00 | 1.00 | 0.82 | 0.92 | 1.00 | 1.00 | - | - | - | - | - | - | - | - | - | - | - | - |
| Cont.0.70Load.000Cross.4Var.1000Size.2Fac.0.50Fcor | 1.00 | 1.00 | 0.68 | 0.86 | 0.98 | 1.00 | - | - | - | - | - | - | - | - | - | - | - | - |
| Cont.0.70Load.000Cross.4Var.1000Size.2Fac.0.70Fcor | 1.00 | 1.00 | 0.67 | 0.83 | 0.62 | 0.43 | - | - | - | - | - | - | - | - | - | - | - | - |
| Cont.0.70Load.000Cross.4Var.300Size.4Fac.0.00Fcor | 0.99 | 1.00 | 0.79 | 0.94 | 1.00 | 1.00 | - | - | - | - | - | - | - | - | - | - | - | - |
| Cont.0.70Load.000Cross.4Var.300Size.4Fac.0.50Fcor | 1.00 | 1.00 | 0.25 | 0.85 | 0.98 | 0.95 | - | - | - | - | - | - | - | - | - | - | - | - |
| Cont.0.70Load.000Cross.4Var.300Size.4Fac.0.70Fcor | 0.91 | 0.91 | 0.22 | 0.76 | 0.87 | 0.07 | - | - | - | - | - | - | - | - | - | - | - | - |
| Cont.0.70Load.000Cross.4Var.500Size.4Fac.0.00Fcor | 0.99 | 1.00 | 0.82 | 0.95 | 1.00 | 1.00 | - | - | - | - | - | - | - | - | - | - | - | - |
| Cont.0.70Load.000Cross.4Var.500Size.4Fac.0.50Fcor | 1.00 | 1.00 | 0.25 | 0.84 | 0.99 | 0.99 | - | - | - | - | - | - | - | - | - | - | - | - |
| Cont.0.70Load.000Cross.4Var.500Size.4Fac.0.70Fcor | 0.95 | 0.95 | 0.20 | 0.82 | 0.92 | 0.12 | - | - | - | - | - | - | - | - | - | - | - | - |
| Cont.0.70Load.000Cross.4Var.1000Size.4Fac.0.00Fcor | 0.99 | 1.00 | 0.73 | 0.94 | 1.00 | 1.00 | - | - | - | - | - | - | - | - | - | - | - | - |
| Cont.0.70Load.000Cross.4Var.1000Size.4Fac.0.50Fcor | 1.00 | 1.00 | 0.24 | 0.81 | 0.99 | 1.00 | - | - | - | - | - | - | - | - | - | - | - | - |
| Cont.0.70Load.000Cross.4Var.1000Size.4Fac.0.70Fcor | 0.99 | 0.99 | 0.26 | 0.89 | 0.95 | 0.25 | - | - | - | - | - | - | - | - | - | - | - | - |
| Cont.0.70Load.000Cross.8Var.300Size.2Fac.0.00Fcor | 0.98 | 1.00 | 0.64 | 0.53 | 1.00 | 1.00 | - | - | - | - | - | - | - | - | - | - | - | - |
| Cont.0.70Load.000Cross.8Var.300Size.2Fac.0.50Fcor | 1.00 | 1.00 | 0.58 | 0.55 | 1.00 | 1.00 | - | - | - | - | - | - | - | - | - | - | - | - |
| Cont.0.70Load.000Cross.8Var.300Size.2Fac.0.70Fcor | 0.99 | 0.99 | 0.52 | 0.55 | 1.00 | 1.00 | - | - | - | - | - | - | - | - | - | - | - | - |
| Cont.0.70Load.000Cross.8Var.500Size.2Fac.0.00Fcor | 0.99 | 1.00 | 0.67 | 0.61 | 1.00 | 1.00 | - | - | - | - | - | - | - | - | - | - | - | - |
| Cont.0.70Load.000Cross.8Var.500Size.2Fac.0.50Fcor | 1.00 | 1.00 | 0.62 | 0.62 | 1.00 | 1.00 | - | - | - | - | - | - | - | - | - | - | - | - |
| Cont.0.70Load.000Cross.8Var.500Size.2Fac.0.70Fcor | 0.99 | 0.99 | 0.59 | 0.63 | 1.00 | 1.00 | - | - | - | - | - | - | - | - | - | - | - | - |
| Cont.0.70Load.000Cross.8Var.1000Size.2Fac.0.00Fcor | 0.99 | 1.00 | 0.69 | 0.63 | 1.00 | 1.00 | - | - | - | - | - | - | - | - | - | - | - | - |
| Cont.0.70Load.000Cross.8Var.1000Size.2Fac.0.50Fcor | 1.00 | 1.00 | 0.60 | 0.68 | 1.00 | 1.00 | - | - | - | - | - | - | - | - | - | - | - | - |
| Cont.0.70Load.000Cross.8Var.1000Size.2Fac.0.70Fcor | 0.96 | 0.96 | 0.54 | 0.64 | 1.00 | 1.00 | - | - | - | - | - | - | - | - | - | - | - | - |
| Cont.0.70Load.000Cross.8Var.300Size.4Fac.0.00Fcor | 0.99 | 1.00 | 0.62 | 0.76 | 1.00 | 1.00 | - | - | - | - | - | - | - | - | - | - | - | - |
| Cont.0.70Load.000Cross.8Var.300Size.4Fac.0.50Fcor | 1.00 | 1.00 | 0.40 | 0.72 | 1.00 | 1.00 | - | - | - | - | - | - | - | - | - | - | - | - |
| Cont.0.70Load.000Cross.8Var.300Size.4Fac.0.70Fcor | 0.94 | 0.94 | 0.27 | 0.71 | 1.00 | 0.88 | - | - | - | - | - | - | - | - | - | - | - | - |
| Cont.0.70Load.000Cross.8Var.500Size.4Fac.0.00Fcor | 0.99 | 1.00 | 0.54 | 0.80 | 1.00 | 1.00 | - | - | - | - | - | - | - | - | - | - | - | - |
| Cont.0.70Load.000Cross.8Var.500Size.4Fac.0.50Fcor | 1.00 | 1.00 | 0.37 | 0.80 | 1.00 | 1.00 | - | - | - | - | - | - | - | - | - | - | - | - |
| Cont.0.70Load.000Cross.8Var.500Size.4Fac.0.70Fcor | 0.97 | 0.97 | 0.25 | 0.77 | 1.00 | 0.99 | - | - | - | - | - | - | - | - | - | - | - | - |
| Cont.0.70Load.000Cross.8Var.1000Size.4Fac.0.00Fcor | 0.99 | 1.00 | 0.60 | 0.74 | 1.00 | 1.00 | - | - | - | - | - | - | - | - | - | - | - | - |
| Cont.0.70Load.000Cross.8Var.1000Size.4Fac.0.50Fcor | 1.00 | 1.00 | 0.29 | 0.76 | 1.00 | 1.00 | - | - | - | - | - | - | - | - | - | - | - | - |
| Cont.0.70Load.000Cross.8Var.1000Size.4Fac.0.70Fcor | 0.99 | 0.99 | 0.26 | 0.67 | 1.00 | 1.00 | - | - | - | - | - | - | - | - | - | - | - | - |
| Cont.0.70Load.125Cross.4Var.300Size.2Fac.0.00Fcor | 0.85 | 0.85 | 0.72 | 0.55 | 0.95 | 0.97 | 47.00 | 48.00 | 56.00 | 10.00 | 100.00 | 86.00 | 100.00 | 100.00 | 95.86 | 96.29 | 97.00 | 100.00 |
| Cont.0.70Load.125Cross.4Var.300Size.2Fac.0.50Fcor | 0.99 | 0.99 | 0.84 | 0.61 | 0.89 | 0.90 | 98.00 | 98.00 | 93.00 | 60.00 | 98.00 | 83.00 | 99.86 | 99.86 | 95.71 | 94.43 | 96.00 | 100.00 |
| Cont.0.70Load.125Cross.4Var.300Size.2Fac.0.70Fcor | 0.96 | 0.96 | 0.79 | 0.60 | 0.25 | 0.07 | 100.00 | 100.00 | 100.00 | 78.00 | 31.00 | 7.00 | 98.86 | 98.86 | 93.29 | 96.71 | 97.57 | 100.00 |
| Cont.0.70Load.125Cross.4Var.500Size.2Fac.0.00Fcor | 0.83 | 0.84 | 0.73 | 0.54 | 0.98 | 0.97 | 40.00 | 42.00 | 60.00 | 2.00 | 100.00 | 90.00 | 100.00 | 100.00 | 95.71 | 97.43 | 98.86 | 100.00 |
| Cont.0.70Load.125Cross.4Var.500Size.2Fac.0.50Fcor | 0.99 | 0.99 | 0.82 | 0.61 | 0.94 | 0.94 | 96.00 | 96.00 | 97.00 | 67.00 | 99.00 | 84.00 | 100.00 | 100.00 | 95.71 | 96.43 | 97.71 | 100.00 |
| Cont.0.70Load.125Cross.4Var.500Size.2Fac.0.70Fcor | 0.98 | 0.98 | 0.80 | 0.58 | 0.16 | 0.05 | 100.00 | 100.00 | 100.00 | 81.00 | 19.00 | 4.00 | 99.43 | 99.43 | 96.14 | 98.43 | 98.57 | 100.00 |
| Cont.0.70Load.125Cross.4Var.1000Size.2Fac.0.00Fcor | 0.82 | 0.82 | 0.74 | 0.57 | 0.99 | 0.99 | 36.00 | 37.00 | 58.00 | 3.00 | 100.00 | 97.00 | 100.00 | 100.00 | 97.29 | 98.86 | 99.14 | 100.00 |
| Cont.0.70Load.125Cross.4Var.1000Size.2Fac.0.50Fcor | 1.00 | 1.00 | 0.85 | 0.55 | 0.95 | 0.99 | 100.00 | 100.00 | 99.00 | 55.00 | 100.00 | 95.00 | 100.00 | 100.00 | 95.71 | 97.57 | 97.71 | 100.00 |
| Cont.0.70Load.125Cross.4Var.1000Size.2Fac.0.70Fcor | 0.99 | 0.99 | 0.84 | 0.63 | 0.09 | 0.06 | 100.00 | 100.00 | 100.00 | 77.00 | 11.00 | 6.00 | 99.57 | 99.57 | 96.71 | 99.00 | 99.14 | 100.00 |
| Cont.0.70Load.125Cross.4Var.300Size.4Fac.0.00Fcor | 0.93 | 0.93 | 0.60 | 0.71 | 0.99 | 0.97 | 62.00 | 63.00 | 29.50 | 14.50 | 95.00 | 80.00 | 99.79 | 99.93 | 97.07 | 98.86 | 99.93 | 100.00 |
| Cont.0.70Load.125Cross.4Var.300Size.4Fac.0.50Fcor | 0.96 | 0.96 | 0.39 | 0.77 | 0.95 | 0.81 | 91.50 | 92.00 | 62.50 | 73.00 | 96.50 | 48.00 | 99.14 | 99.14 | 86.07 | 91.50 | 98.93 | 100.00 |
| Cont.0.70Load.125Cross.4Var.300Size.4Fac.0.70Fcor | 0.83 | 0.83 | 0.28 | 0.77 | 0.79 | 0.05 | 86.00 | 86.00 | 60.50 | 84.50 | 84.00 | 2.00 | 95.79 | 95.79 | 84.21 | 93.50 | 96.79 | 99.93 |
| Cont.0.70Load.125Cross.4Var.500Size.4Fac.0.00Fcor | 0.95 | 0.95 | 0.56 | 0.68 | 1.00 | 0.98 | 72.00 | 73.00 | 33.00 | 4.50 | 99.00 | 90.50 | 99.86 | 100.00 | 95.07 | 98.43 | 99.79 | 100.00 |
| Cont.0.70Load.125Cross.4Var.500Size.4Fac.0.50Fcor | 0.98 | 0.98 | 0.37 | 0.79 | 0.96 | 0.93 | 97.50 | 97.50 | 65.00 | 75.50 | 97.50 | 73.00 | 99.50 | 99.50 | 87.43 | 92.57 | 99.29 | 100.00 |
| Cont.0.70Load.125Cross.4Var.500Size.4Fac.0.70Fcor | 0.87 | 0.87 | 0.37 | 0.83 | 0.84 | 0.10 | 87.50 | 87.50 | 52.50 | 91.50 | 90.50 | 3.50 | 97.43 | 97.43 | 87.79 | 95.86 | 97.64 | 100.00 |
| Cont.0.70Load.125Cross.4Var.1000Size.4Fac.0.00Fcor | 0.97 | 0.97 | 0.42 | 0.69 | 0.99 | 0.99 | 82.50 | 81.50 | 23.00 | 4.50 | 98.50 | 95.00 | 99.93 | 100.00 | 92.50 | 98.71 | 99.86 | 100.00 |
| Cont.0.70Load.125Cross.4Var.1000Size.4Fac.0.50Fcor | 0.99 | 0.99 | 0.38 | 0.85 | 0.97 | 0.97 | 96.00 | 96.00 | 62.50 | 86.50 | 98.00 | 86.50 | 99.93 | 99.93 | 87.71 | 95.14 | 99.57 | 100.00 |
| Cont.0.70Load.125Cross.4Var.1000Size.4Fac.0.70Fcor | 0.91 | 0.91 | 0.37 | 0.83 | 0.84 | 0.19 | 94.50 | 94.50 | 55.50 | 91.50 | 91.00 | 7.50 | 98.64 | 98.64 | 86.93 | 95.86 | 97.93 | 100.00 |
| Cont.0.70Load.125Cross.8Var.300Size.2Fac.0.00Fcor | 0.83 | 0.79 | 0.49 | 0.33 | 0.78 | 0.96 | 44.00 | 36.50 | 12.00 | 1.00 | 52.00 | 86.50 | 99.36 | 99.14 | 91.93 | 81.50 | 97.21 | 100.00 |
| Cont.0.70Load.125Cross.8Var.300Size.2Fac.0.50Fcor | 0.97 | 0.97 | 0.46 | 0.42 | 0.98 | 0.98 | 87.50 | 87.50 | 45.00 | 48.00 | 99.50 | 94.00 | 100.00 | 100.00 | 79.71 | 75.64 | 98.29 | 100.00 |
| Cont.0.70Load.125Cross.8Var.300Size.2Fac.0.70Fcor | 0.98 | 0.98 | 0.28 | 0.42 | 1.00 | 0.89 | 98.50 | 98.50 | 50.00 | 75.00 | 100.00 | 82.00 | 99.36 | 99.36 | 82.93 | 85.29 | 99.57 | 100.00 |
| Cont.0.70Load.125Cross.8Var.500Size.2Fac.0.00Fcor | 0.85 | 0.79 | 0.51 | 0.39 | 0.79 | 0.98 | 55.50 | 43.00 | 30.50 | 6.00 | 55.00 | 94.00 | 99.43 | 99.29 | 89.43 | 86.07 | 97.14 | 100.00 |
| Cont.0.70Load.125Cross.8Var.500Size.2Fac.0.50Fcor | 0.97 | 0.97 | 0.46 | 0.44 | 0.99 | 0.99 | 88.00 | 88.00 | 38.00 | 43.50 | 99.50 | 96.00 | 100.00 | 100.00 | 80.50 | 74.64 | 98.57 | 100.00 |
| Cont.0.70Load.125Cross.8Var.500Size.2Fac.0.70Fcor | 1.00 | 1.00 | 0.42 | 0.55 | 1.00 | 0.98 | 100.00 | 100.00 | 58.50 | 84.00 | 100.00 | 94.00 | 100.00 | 100.00 | 88.07 | 89.86 | 100.00 | 100.00 |
| Cont.0.70Load.125Cross.8Var.1000Size.2Fac.0.00Fcor | 0.87 | 0.83 | 0.54 | 0.43 | 0.85 | 1.00 | 65.50 | 57.50 | 46.50 | 11.00 | 70.50 | 98.50 | 99.43 | 99.43 | 87.29 | 87.93 | 98.71 | 100.00 |
| Cont.0.70Load.125Cross.8Var.1000Size.2Fac.0.50Fcor | 0.98 | 0.98 | 0.50 | 0.51 | 0.99 | 1.00 | 90.50 | 90.50 | 42.00 | 49.50 | 100.00 | 99.50 | 100.00 | 100.00 | 85.36 | 82.50 | 99.07 | 100.00 |
| Cont.0.70Load.125Cross.8Var.1000Size.2Fac.0.70Fcor | 1.00 | 1.00 | 0.44 | 0.59 | 1.00 | 1.00 | 100.00 | 100.00 | 70.50 | 94.50 | 100.00 | 99.00 | 100.00 | 100.00 | 89.71 | 97.50 | 100.00 | 100.00 |
| Cont.0.70Load.125Cross.8Var.300Size.4Fac.0.00Fcor | 0.94 | 0.94 | 0.55 | 0.58 | 0.91 | 0.99 | 62.25 | 62.50 | 38.00 | 4.25 | 43.00 | 95.00 | 99.82 | 100.00 | 94.46 | 88.39 | 100.00 | 100.00 |
| Cont.0.70Load.125Cross.8Var.300Size.4Fac.0.50Fcor | 0.99 | 0.99 | 0.51 | 0.67 | 0.99 | 0.99 | 95.75 | 95.75 | 62.75 | 60.75 | 99.50 | 94.25 | 99.68 | 99.68 | 92.96 | 83.18 | 99.75 | 100.00 |
| Cont.0.70Load.125Cross.8Var.300Size.4Fac.0.70Fcor | 0.96 | 0.96 | 0.38 | 0.66 | 1.00 | 0.77 | 97.25 | 97.25 | 61.25 | 83.75 | 99.00 | 52.25 | 97.86 | 97.86 | 90.21 | 86.25 | 99.93 | 99.89 |
| Cont.0.70Load.125Cross.8Var.500Size.4Fac.0.00Fcor | 0.94 | 0.94 | 0.55 | 0.61 | 0.95 | 1.00 | 64.75 | 64.75 | 42.00 | 4.00 | 70.75 | 99.00 | 99.96 | 100.00 | 95.86 | 92.93 | 100.00 | 100.00 |
| Cont.0.70Load.125Cross.8Var.500Size.4Fac.0.50Fcor | 0.99 | 0.99 | 0.53 | 0.69 | 0.99 | 0.99 | 95.00 | 95.00 | 64.25 | 66.50 | 100.00 | 96.75 | 99.93 | 99.93 | 95.11 | 86.79 | 99.86 | 100.00 |
| Cont.0.70Load.125Cross.8Var.500Size.4Fac.0.70Fcor | 0.99 | 0.99 | 0.41 | 0.75 | 1.00 | 0.95 | 98.75 | 98.75 | 63.25 | 86.75 | 99.50 | 81.50 | 99.36 | 99.36 | 90.39 | 91.36 | 100.00 | 99.93 |
| Cont.0.70Load.125Cross.8Var.1000Size.4Fac.0.00Fcor | 0.97 | 0.97 | 0.47 | 0.64 | 1.00 | 1.00 | 83.00 | 83.50 | 56.25 | 24.25 | 98.50 | 99.75 | 99.93 | 99.96 | 89.00 | 91.79 | 100.00 | 100.00 |
| Cont.0.70Load.125Cross.8Var.1000Size.4Fac.0.50Fcor | 1.00 | 1.00 | 0.49 | 0.78 | 0.99 | 1.00 | 97.25 | 97.25 | 64.75 | 77.50 | 100.00 | 99.50 | 100.00 | 100.00 | 94.00 | 91.07 | 99.75 | 100.00 |
| Cont.0.70Load.125Cross.8Var.1000Size.4Fac.0.70Fcor | 0.99 | 0.99 | 0.40 | 0.77 | 1.00 | 0.99 | 99.75 | 99.75 | 65.00 | 91.50 | 100.00 | 94.50 | 99.64 | 99.64 | 92.00 | 93.75 | 100.00 | 100.00 |
| Cont.0.70Load.Cross250.4Var.300Size.2Fac.0.00Fcor | 0.69 | 0.66 | 0.45 | 0.29 | 0.56 | 0.83 | 55.00 | 48.50 | 35.50 | 7.50 | 92.00 | 67.00 | 98.50 | 98.83 | 90.83 | 95.17 | 75.67 | 100.00 |
| Cont.0.70Load.Cross250.4Var.300Size.2Fac.0.50Fcor | 0.97 | 0.97 | 0.51 | 0.49 | 0.66 | 0.66 | 94.50 | 94.50 | 48.50 | 50.00 | 86.00 | 55.00 | 99.67 | 99.67 | 82.67 | 90.67 | 90.17 | 100.00 |
| Cont.0.70Load.Cross250.4Var.300Size.2Fac.0.70Fcor | 0.98 | 0.98 | 0.79 | 0.61 | 0.02 | 0.01 | 100.00 | 100.00 | 95.00 | 66.50 | 2.00 | 1.00 | 99.00 | 99.00 | 93.17 | 98.33 | 99.83 | 100.00 |
| Cont.0.70Load.Cross250.4Var.500Size.2Fac.0.00Fcor | 0.83 | 0.81 | 0.42 | 0.26 | 0.59 | 0.88 | 74.00 | 71.50 | 46.00 | 3.00 | 98.00 | 78.00 | 99.50 | 99.33 | 86.83 | 98.50 | 75.83 | 100.00 |
| Cont.0.70Load.Cross250.4Var.500Size.2Fac.0.50Fcor | 1.00 | 1.00 | 0.43 | 0.47 | 0.64 | 0.72 | 99.00 | 99.00 | 41.00 | 44.00 | 81.00 | 64.50 | 100.00 | 100.00 | 76.00 | 87.83 | 91.83 | 100.00 |
| Cont.0.70Load.Cross250.4Var.500Size.2Fac.0.70Fcor | 0.99 | 0.99 | 0.82 | 0.61 | 0.01 | 0.00 | 100.00 | 100.00 | 94.50 | 64.50 | 1.00 | 0.00 | 99.67 | 99.67 | 95.00 | 99.50 | 100.00 | 100.00 |
| Cont.0.70Load.Cross250.4Var.1000Size.2Fac.0.00Fcor | 0.95 | 0.95 | 0.38 | 0.25 | 0.62 | 0.91 | 91.00 | 91.00 | 37.00 | 4.50 | 99.00 | 84.00 | 99.83 | 99.83 | 90.00 | 96.50 | 77.83 | 100.00 |
| Cont.0.70Load.Cross250.4Var.1000Size.2Fac.0.50Fcor | 1.00 | 1.00 | 0.45 | 0.46 | 0.70 | 0.88 | 100.00 | 100.00 | 46.50 | 48.50 | 81.00 | 81.00 | 100.00 | 100.00 | 72.50 | 82.83 | 95.17 | 100.00 |
| Cont.0.70Load.Cross250.4Var.1000Size.2Fac.0.70Fcor | 1.00 | 1.00 | 0.91 | 0.67 | 0.00 | 0.00 | 100.00 | 100.00 | 100.00 | 72.00 | 0.00 | 0.00 | 100.00 | 100.00 | 99.17 | 100.00 | 100.00 | 100.00 |
| Cont.0.70Load.Cross250.4Var.300Size.4Fac.0.00Fcor | 0.90 | 0.90 | 0.53 | 0.53 | 0.98 | 0.94 | 67.75 | 68.50 | 49.00 | 19.25 | 93.50 | 81.75 | 99.83 | 99.92 | 96.92 | 99.25 | 99.67 | 100.00 |
| Cont.0.70Load.Cross250.4Var.300Size.4Fac.0.50Fcor | 0.99 | 0.99 | 0.48 | 0.80 | 0.92 | 0.72 | 97.50 | 97.50 | 64.75 | 80.50 | 96.25 | 49.75 | 99.75 | 99.75 | 95.25 | 92.75 | 98.42 | 100.00 |
| Cont.0.70Load.Cross250.4Var.300Size.4Fac.0.70Fcor | 0.88 | 0.88 | 0.40 | 0.80 | 0.60 | 0.02 | 93.75 | 93.75 | 63.50 | 92.75 | 71.25 | 0.75 | 97.50 | 97.50 | 90.92 | 94.58 | 97.58 | 99.92 |
| Cont.0.70Load.Cross250.4Var.500Size.4Fac.0.00Fcor | 0.92 | 0.92 | 0.51 | 0.43 | 0.99 | 0.97 | 74.25 | 75.00 | 61.25 | 5.50 | 99.00 | 90.50 | 100.00 | 100.00 | 92.58 | 99.08 | 99.50 | 100.00 |
| Cont.0.70Load.Cross250.4Var.500Size.4Fac.0.50Fcor | 0.99 | 0.99 | 0.49 | 0.88 | 0.96 | 0.82 | 96.75 | 96.75 | 65.75 | 89.50 | 98.00 | 65.00 | 100.00 | 100.00 | 95.00 | 93.50 | 99.33 | 99.50 |
| Cont.0.70Load.Cross250.4Var.500Size.4Fac.0.70Fcor | 0.94 | 0.94 | 0.41 | 0.84 | 0.56 | 0.05 | 98.25 | 98.25 | 67.00 | 95.00 | 64.25 | 1.50 | 98.67 | 98.67 | 90.17 | 96.83 | 98.17 | 100.00 |
| Cont.0.70Load.Cross250.4Var.1000Size.4Fac.0.00Fcor | 0.97 | 0.97 | 0.41 | 0.39 | 0.99 | 0.99 | 89.75 | 90.00 | 62.75 | 0.25 | 99.50 | 97.50 | 100.00 | 100.00 | 85.33 | 100.00 | 99.33 | 100.00 |
| Cont.0.70Load.Cross250.4Var.1000Size.4Fac.0.50Fcor | 0.99 | 0.99 | 0.56 | 0.86 | 0.96 | 0.93 | 97.25 | 97.25 | 69.25 | 84.25 | 99.00 | 84.75 | 100.00 | 100.00 | 95.58 | 96.00 | 99.75 | 100.00 |
| Cont.0.70Load.Cross250.4Var.1000Size.4Fac.0.70Fcor | 0.96 | 0.96 | 0.42 | 0.84 | 0.46 | 0.10 | 98.25 | 98.25 | 71.00 | 95.50 | 50.00 | 2.75 | 99.25 | 99.25 | 91.58 | 97.00 | 99.25 | 100.00 |
| Cont.0.70Load.Cross250.8Var.300Size.2Fac.0.00Fcor | 0.83 | 0.79 | 0.49 | 0.33 | 0.78 | 0.96 | 44.00 | 36.50 | 12.00 | 1.00 | 52.00 | 86.50 | 99.36 | 99.14 | 91.93 | 81.50 | 97.21 | 100.00 |
| Cont.0.70Load.Cross250.8Var.300Size.2Fac.0.50Fcor | 0.97 | 0.97 | 0.46 | 0.42 | 0.98 | 0.98 | 87.50 | 87.50 | 45.00 | 48.00 | 99.50 | 94.00 | 100.00 | 100.00 | 79.71 | 75.64 | 98.29 | 100.00 |
| Cont.0.70Load.Cross250.8Var.300Size.2Fac.0.70Fcor | 0.98 | 0.98 | 0.28 | 0.42 | 1.00 | 0.89 | 98.50 | 98.50 | 50.00 | 75.00 | 100.00 | 82.00 | 99.36 | 99.36 | 82.93 | 85.29 | 99.57 | 100.00 |
| Cont.0.70Load.Cross250.8Var.500Size.2Fac.0.00Fcor | 0.85 | 0.79 | 0.51 | 0.39 | 0.79 | 0.98 | 55.50 | 43.00 | 30.50 | 6.00 | 55.00 | 94.00 | 99.43 | 99.29 | 89.43 | 86.07 | 97.14 | 100.00 |
| Cont.0.70Load.Cross250.8Var.500Size.2Fac.0.50Fcor | 0.97 | 0.97 | 0.46 | 0.44 | 0.99 | 0.99 | 88.00 | 88.00 | 38.00 | 43.50 | 99.50 | 96.00 | 100.00 | 100.00 | 80.50 | 74.64 | 98.57 | 100.00 |
| Cont.0.70Load.Cross250.8Var.500Size.2Fac.0.70Fcor | 1.00 | 1.00 | 0.42 | 0.55 | 1.00 | 0.98 | 100.00 | 100.00 | 58.50 | 84.00 | 100.00 | 94.00 | 100.00 | 100.00 | 88.07 | 89.86 | 100.00 | 100.00 |
| Cont.0.70Load.Cross250.8Var.1000Size.2Fac.0.00Fcor | 0.87 | 0.83 | 0.54 | 0.43 | 0.85 | 1.00 | 65.50 | 57.50 | 46.50 | 11.00 | 70.50 | 98.50 | 99.43 | 99.43 | 87.29 | 87.93 | 98.71 | 100.00 |
| Cont.0.70Load.Cross250.8Var.1000Size.2Fac.0.50Fcor | 0.98 | 0.98 | 0.50 | 0.51 | 0.99 | 1.00 | 90.50 | 90.50 | 42.00 | 49.50 | 100.00 | 99.50 | 100.00 | 100.00 | 85.36 | 82.50 | 99.07 | 100.00 |
| Cont.0.70Load.Cross250.8Var.1000Size.2Fac.0.70Fcor | 1.00 | 1.00 | 0.44 | 0.59 | 1.00 | 1.00 | 100.00 | 100.00 | 70.50 | 94.50 | 100.00 | 99.00 | 100.00 | 100.00 | 89.71 | 97.50 | 100.00 | 100.00 |
| Cont.0.70Load.Cross250.8Var.300Size.4Fac.0.00Fcor | 0.90 | 0.90 | 0.40 | 0.44 | 0.72 | 0.98 | 69.75 | 70.00 | 41.38 | 10.38 | 15.62 | 92.62 | 99.67 | 99.75 | 97.92 | 88.00 | 99.83 | 100.00 |
| Cont.0.70Load.Cross250.8Var.300Size.4Fac.0.50Fcor | 0.97 | 0.97 | 0.43 | 0.59 | 0.85 | 0.98 | 93.88 | 93.88 | 50.38 | 56.38 | 71.25 | 92.38 | 99.71 | 99.71 | 94.38 | 80.33 | 97.79 | 100.00 |
| Cont.0.70Load.Cross250.8Var.300Size.4Fac.0.70Fcor | 0.93 | 0.93 | 0.33 | 0.57 | 0.88 | 0.63 | 95.88 | 95.88 | 51.12 | 72.88 | 76.50 | 43.38 | 96.79 | 96.71 | 90.96 | 82.67 | 99.50 | 99.92 |
| Cont.0.70Load.Cross250.8Var.500Size.4Fac.0.00Fcor | 0.92 | 0.92 | 0.43 | 0.48 | 0.75 | 0.99 | 76.50 | 76.12 | 48.25 | 12.62 | 29.38 | 97.00 | 99.79 | 99.88 | 95.92 | 89.38 | 99.62 | 100.00 |
| Cont.0.70Load.Cross250.8Var.500Size.4Fac.0.50Fcor | 0.97 | 0.97 | 0.44 | 0.53 | 0.86 | 0.99 | 91.12 | 90.88 | 49.25 | 50.38 | 76.75 | 97.88 | 99.96 | 99.96 | 95.42 | 81.12 | 97.92 | 100.00 |
| Cont.0.70Load.Cross250.8Var.500Size.4Fac.0.70Fcor | 0.99 | 0.99 | 0.40 | 0.63 | 0.91 | 0.87 | 96.88 | 96.88 | 53.62 | 72.88 | 83.50 | 72.50 | 99.75 | 99.75 | 92.71 | 89.75 | 99.21 | 99.96 |
| Cont.0.70Load.Cross250.8Var.1000Size.4Fac.0.00Fcor | 0.95 | 0.95 | 0.37 | 0.52 | 0.84 | 1.00 | 86.50 | 86.12 | 52.38 | 20.75 | 52.12 | 99.75 | 99.50 | 99.50 | 90.83 | 90.71 | 99.88 | 100.00 |
| Cont.0.70Load.Cross250.8Var.1000Size.4Fac.0.50Fcor | 0.97 | 0.97 | 0.40 | 0.56 | 0.87 | 1.00 | 91.12 | 91.12 | 43.38 | 47.38 | 81.62 | 99.50 | 100.00 | 100.00 | 98.00 | 82.04 | 97.00 | 100.00 |
| Cont.0.70Load.Cross250.8Var.1000Size.4Fac.0.70Fcor | 1.00 | 1.00 | 0.39 | 0.65 | 0.92 | 0.96 | 99.62 | 99.62 | 49.00 | 76.00 | 89.75 | 87.00 | 99.83 | 99.83 | 95.29 | 92.12 | 98.96 | 100.00 |
| Ord.0.40Load.000Cross.4Var.300Size.2Fac.0.00Fcor | 0.43 | 0.43 | 0.31 | 0.30 | 0.70 | 0.92 | - | - | - | - | - | - | - | - | - | - | - | - |
| Ord.0.40Load.000Cross.4Var.300Size.2Fac.0.50Fcor | 0.26 | 0.26 | 0.20 | 0.19 | 0.38 | 0.33 | - | - | - | - | - | - | - | - | - | - | - | - |
| Ord.0.40Load.000Cross.4Var.300Size.2Fac.0.70Fcor | 0.13 | 0.12 | 0.08 | 0.10 | 0.17 | 0.05 | - | - | - | - | - | - | - | - | - | - | - | - |
| Ord.0.40Load.000Cross.4Var.500Size.2Fac.0.00Fcor | 0.78 | 0.78 | 0.63 | 0.58 | 0.92 | 0.99 | - | - | - | - | - | - | - | - | - | - | - | - |
| Ord.0.40Load.000Cross.4Var.500Size.2Fac.0.50Fcor | 0.46 | 0.46 | 0.36 | 0.39 | 0.67 | 0.52 | - | - | - | - | - | - | - | - | - | - | - | - |
| Ord.0.40Load.000Cross.4Var.500Size.2Fac.0.70Fcor | 0.22 | 0.22 | 0.15 | 0.15 | 0.31 | 0.08 | - | - | - | - | - | - | - | - | - | - | - | - |
| Ord.0.40Load.000Cross.4Var.1000Size.2Fac.0.00Fcor | 0.96 | 0.96 | 0.85 | 0.80 | 0.99 | 1.00 | - | - | - | - | - | - | - | - | - | - | - | - |
| Ord.0.40Load.000Cross.4Var.1000Size.2Fac.0.50Fcor | 0.62 | 0.62 | 0.53 | 0.56 | 0.74 | 0.77 | - | - | - | - | - | - | - | - | - | - | - | - |
| Ord.0.40Load.000Cross.4Var.1000Size.2Fac.0.70Fcor | 0.28 | 0.28 | 0.21 | 0.20 | 0.33 | 0.06 | - | - | - | - | - | - | - | - | - | - | - | - |
| Ord.0.40Load.000Cross.4Var.300Size.4Fac.0.00Fcor | 0.25 | 0.25 | 0.22 | 0.24 | 0.46 | 0.84 | - | - | - | - | - | - | - | - | - | - | - | - |
| Ord.0.40Load.000Cross.4Var.300Size.4Fac.0.50Fcor | 0.11 | 0.11 | 0.08 | 0.10 | 0.25 | 0.14 | - | - | - | - | - | - | - | - | - | - | - | - |
| Ord.0.40Load.000Cross.4Var.300Size.4Fac.0.70Fcor | 0.07 | 0.07 | 0.04 | 0.06 | 0.13 | 0.01 | - | - | - | - | - | - | - | - | - | - | - | - |
| Ord.0.40Load.000Cross.4Var.500Size.4Fac.0.00Fcor | 0.67 | 0.67 | 0.59 | 0.66 | 0.92 | 0.98 | - | - | - | - | - | - | - | - | - | - | - | - |
| Ord.0.40Load.000Cross.4Var.500Size.4Fac.0.50Fcor | 0.29 | 0.29 | 0.21 | 0.26 | 0.52 | 0.29 | - | - | - | - | - | - | - | - | - | - | - | - |
| Ord.0.40Load.000Cross.4Var.500Size.4Fac.0.70Fcor | 0.14 | 0.14 | 0.08 | 0.12 | 0.23 | 0.02 | - | - | - | - | - | - | - | - | - | - | - | - |
| Ord.0.40Load.000Cross.4Var.1000Size.4Fac.0.00Fcor | 0.91 | 0.91 | 0.77 | 0.89 | 0.99 | 1.00 | - | - | - | - | - | - | - | - | - | - | - | - |
| Ord.0.40Load.000Cross.4Var.1000Size.4Fac.0.50Fcor | 0.40 | 0.40 | 0.28 | 0.38 | 0.68 | 0.54 | - | - | - | - | - | - | - | - | - | - | - | - |
| Ord.0.40Load.000Cross.4Var.1000Size.4Fac.0.70Fcor | 0.15 | 0.15 | 0.11 | 0.13 | 0.31 | 0.03 | - | - | - | - | - | - | - | - | - | - | - | - |
| Ord.0.40Load.000Cross.8Var.300Size.2Fac.0.00Fcor | 0.54 | 0.54 | 0.37 | 0.39 | 0.71 | 1.00 | - | - | - | - | - | - | - | - | - | - | - | - |
| Ord.0.40Load.000Cross.8Var.300Size.2Fac.0.50Fcor | 0.26 | 0.26 | 0.16 | 0.22 | 0.50 | 0.82 | - | - | - | - | - | - | - | - | - | - | - | - |
| Ord.0.40Load.000Cross.8Var.300Size.2Fac.0.70Fcor | 0.15 | 0.15 | 0.10 | 0.14 | 0.30 | 0.19 | - | - | - | - | - | - | - | - | - | - | - | - |
| Ord.0.40Load.000Cross.8Var.500Size.2Fac.0.00Fcor | 0.88 | 0.88 | 0.61 | 0.54 | 0.98 | 1.00 | - | - | - | - | - | - | - | - | - | - | - | - |
| Ord.0.40Load.000Cross.8Var.500Size.2Fac.0.50Fcor | 0.57 | 0.57 | 0.38 | 0.40 | 0.91 | 0.96 | - | - | - | - | - | - | - | - | - | - | - | - |
| Ord.0.40Load.000Cross.8Var.500Size.2Fac.0.70Fcor | 0.27 | 0.27 | 0.13 | 0.20 | 0.61 | 0.31 | - | - | - | - | - | - | - | - | - | - | - | - |
| Ord.0.40Load.000Cross.8Var.1000Size.2Fac.0.00Fcor | 0.97 | 0.99 | 0.63 | 0.60 | 1.00 | 1.00 | - | - | - | - | - | - | - | - | - | - | - | - |
| Ord.0.40Load.000Cross.8Var.1000Size.2Fac.0.50Fcor | 0.75 | 0.75 | 0.52 | 0.51 | 0.99 | 1.00 | - | - | - | - | - | - | - | - | - | - | - | - |
| Ord.0.40Load.000Cross.8Var.1000Size.2Fac.0.70Fcor | 0.40 | 0.40 | 0.23 | 0.30 | 0.86 | 0.71 | - | - | - | - | - | - | - | - | - | - | - | - |
| Ord.0.40Load.000Cross.8Var.300Size.4Fac.0.00Fcor | 0.24 | 0.24 | 0.17 | 0.22 | 0.40 | 0.98 | - | - | - | - | - | - | - | - | - | - | - | - |
| Ord.0.40Load.000Cross.8Var.300Size.4Fac.0.50Fcor | 0.06 | 0.06 | 0.05 | 0.06 | 0.18 | 0.53 | - | - | - | - | - | - | - | - | - | - | - | - |
| Ord.0.40Load.000Cross.8Var.300Size.4Fac.0.70Fcor | 0.04 | 0.04 | 0.03 | 0.04 | 0.10 | 0.05 | - | - | - | - | - | - | - | - | - | - | - | - |
| Ord.0.40Load.000Cross.8Var.500Size.4Fac.0.00Fcor | 0.82 | 0.82 | 0.48 | 0.69 | 0.97 | 1.00 | - | - | - | - | - | - | - | - | - | - | - | - |
| Ord.0.40Load.000Cross.8Var.500Size.4Fac.0.50Fcor | 0.33 | 0.33 | 0.19 | 0.31 | 0.77 | 0.83 | - | - | - | - | - | - | - | - | - | - | - | - |
| Ord.0.40Load.000Cross.8Var.500Size.4Fac.0.70Fcor | 0.13 | 0.13 | 0.06 | 0.11 | 0.36 | 0.11 | - | - | - | - | - | - | - | - | - | - | - | - |
| Ord.0.40Load.000Cross.8Var.1000Size.4Fac.0.00Fcor | 0.97 | 0.98 | 0.53 | 0.79 | 1.00 | 1.00 | - | - | - | - | - | - | - | - | - | - | - | - |
| Ord.0.40Load.000Cross.8Var.1000Size.4Fac.0.50Fcor | 0.48 | 0.48 | 0.24 | 0.46 | 0.94 | 0.99 | - | - | - | - | - | - | - | - | - | - | - | - |
| Ord.0.40Load.000Cross.8Var.1000Size.4Fac.0.70Fcor | 0.19 | 0.19 | 0.08 | 0.18 | 0.64 | 0.37 | - | - | - | - | - | - | - | - | - | - | - | - |
| Ord.0.40Load.125Cross.4Var.300Size.2Fac.0.00Fcor | 0.33 | 0.33 | 0.26 | 0.21 | 0.52 | 0.70 | 18.00 | 18.00 | 18.00 | 11.00 | 21.00 | 0.00 | 95.86 | 96.00 | 95.14 | 95.14 | 96.43 | 100.00 |
| Ord.0.40Load.125Cross.4Var.300Size.2Fac.0.50Fcor | 0.26 | 0.26 | 0.18 | 0.18 | 0.44 | 0.19 | 36.00 | 36.00 | 36.00 | 35.00 | 58.00 | 0.00 | 90.86 | 90.86 | 89.57 | 91.57 | 88.86 | 100.00 |
| Ord.0.40Load.125Cross.4Var.300Size.2Fac.0.70Fcor | 0.20 | 0.19 | 0.13 | 0.12 | 0.28 | 0.03 | 29.00 | 30.00 | 31.00 | 26.00 | 55.00 | 0.00 | 90.86 | 90.43 | 88.14 | 90.57 | 89.43 | 100.00 |
| Ord.0.40Load.125Cross.4Var.500Size.2Fac.0.00Fcor | 0.55 | 0.55 | 0.42 | 0.35 | 0.74 | 0.74 | 42.00 | 42.00 | 44.00 | 28.00 | 37.00 | 0.00 | 95.14 | 95.14 | 93.14 | 94.86 | 97.14 | 100.00 |
| Ord.0.40Load.125Cross.4Var.500Size.2Fac.0.50Fcor | 0.38 | 0.39 | 0.27 | 0.26 | 0.60 | 0.32 | 45.00 | 46.00 | 47.00 | 39.00 | 81.00 | 0.00 | 91.57 | 91.43 | 90.29 | 92.29 | 90.71 | 100.00 |
| Ord.0.40Load.125Cross.4Var.500Size.2Fac.0.70Fcor | 0.21 | 0.21 | 0.14 | 0.13 | 0.27 | 0.03 | 32.00 | 32.00 | 34.00 | 30.00 | 48.00 | 0.00 | 92.29 | 92.29 | 90.43 | 91.14 | 93.43 | 100.00 |
| Ord.0.40Load.125Cross.4Var.1000Size.2Fac.0.00Fcor | 0.82 | 0.82 | 0.74 | 0.60 | 0.88 | 0.75 | 64.00 | 64.00 | 62.00 | 36.00 | 62.00 | 0.00 | 96.71 | 96.71 | 95.00 | 95.00 | 98.71 | 100.00 |
| Ord.0.40Load.125Cross.4Var.1000Size.2Fac.0.50Fcor | 0.48 | 0.48 | 0.42 | 0.40 | 0.64 | 0.41 | 64.00 | 64.00 | 67.00 | 60.00 | 91.00 | 0.00 | 90.71 | 90.71 | 88.57 | 90.43 | 87.29 | 100.00 |
| Ord.0.40Load.125Cross.4Var.1000Size.2Fac.0.70Fcor | 0.21 | 0.21 | 0.16 | 0.17 | 0.20 | 0.02 | 36.00 | 36.00 | 41.00 | 36.00 | 34.00 | 0.00 | 92.86 | 92.86 | 91.57 | 94.57 | 93.29 | 100.00 |
| Ord.0.40Load.125Cross.4Var.300Size.4Fac.0.00Fcor | 0.15 | 0.15 | 0.11 | 0.11 | 0.34 | 0.68 | 4.50 | 4.50 | 3.00 | 3.00 | 1.50 | 0.00 | 98.79 | 98.57 | 98.86 | 98.79 | 99.86 | 100.00 |
| Ord.0.40Load.125Cross.4Var.300Size.4Fac.0.50Fcor | 0.16 | 0.16 | 0.12 | 0.14 | 0.26 | 0.15 | 25.00 | 25.00 | 23.50 | 28.00 | 27.50 | 0.00 | 93.57 | 93.57 | 93.21 | 93.14 | 95.79 | 100.00 |
| Ord.0.40Load.125Cross.4Var.300Size.4Fac.0.70Fcor | 0.15 | 0.15 | 0.11 | 0.13 | 0.19 | 0.00 | 34.50 | 35.00 | 36.00 | 43.00 | 47.00 | 0.00 | 87.64 | 87.36 | 86.29 | 86.64 | 93.57 | 100.00 |
| Ord.0.40Load.125Cross.4Var.500Size.4Fac.0.00Fcor | 0.53 | 0.53 | 0.46 | 0.51 | 0.71 | 0.79 | 22.00 | 21.50 | 15.00 | 22.50 | 7.00 | 0.00 | 97.00 | 97.29 | 97.29 | 96.93 | 99.71 | 100.00 |
| Ord.0.40Load.125Cross.4Var.500Size.4Fac.0.50Fcor | 0.31 | 0.31 | 0.23 | 0.28 | 0.50 | 0.20 | 39.00 | 39.00 | 45.00 | 49.50 | 51.00 | 0.00 | 88.86 | 88.86 | 85.36 | 86.71 | 95.86 | 100.00 |
| Ord.0.40Load.125Cross.4Var.500Size.4Fac.0.70Fcor | 0.18 | 0.18 | 0.13 | 0.16 | 0.24 | 0.01 | 45.00 | 44.00 | 42.00 | 53.00 | 65.50 | 0.00 | 89.36 | 89.79 | 86.07 | 86.00 | 94.21 | 100.00 |
| Ord.0.40Load.125Cross.4Var.1000Size.4Fac.0.00Fcor | 0.76 | 0.76 | 0.65 | 0.75 | 0.84 | 0.83 | 42.00 | 42.00 | 30.00 | 40.00 | 16.50 | 0.00 | 96.71 | 96.71 | 97.21 | 96.50 | 100.00 | 100.00 |
| Ord.0.40Load.125Cross.4Var.1000Size.4Fac.0.50Fcor | 0.33 | 0.33 | 0.25 | 0.31 | 0.61 | 0.44 | 42.50 | 43.00 | 40.00 | 50.50 | 60.50 | 0.00 | 91.43 | 91.43 | 90.50 | 89.57 | 95.71 | 100.00 |
| Ord.0.40Load.125Cross.4Var.1000Size.4Fac.0.70Fcor | 0.16 | 0.16 | 0.11 | 0.13 | 0.32 | 0.02 | 34.50 | 34.50 | 39.00 | 43.50 | 59.50 | 0.00 | 89.57 | 89.57 | 87.79 | 88.14 | 93.93 | 100.00 |
| Ord.0.40Load.125Cross.8Var.300Size.2Fac.0.00Fcor | 0.29 | 0.29 | 0.18 | 0.19 | 0.42 | 0.74 | 20.50 | 21.00 | 11.50 | 14.00 | 6.50 | 0.00 | 93.29 | 93.50 | 91.79 | 91.36 | 99.14 | 100.00 |
| Ord.0.40Load.125Cross.8Var.300Size.2Fac.0.50Fcor | 0.25 | 0.25 | 0.16 | 0.21 | 0.52 | 0.46 | 44.50 | 45.00 | 37.00 | 54.50 | 47.50 | 0.00 | 87.50 | 87.36 | 84.00 | 84.36 | 95.86 | 100.00 |
| Ord.0.40Load.125Cross.8Var.300Size.2Fac.0.70Fcor | 0.17 | 0.17 | 0.10 | 0.16 | 0.37 | 0.07 | 53.00 | 53.00 | 47.00 | 63.00 | 59.50 | 0.00 | 86.14 | 86.14 | 81.64 | 81.64 | 95.36 | 100.00 |
| Ord.0.40Load.125Cross.8Var.500Size.2Fac.0.00Fcor | 0.68 | 0.68 | 0.41 | 0.40 | 0.73 | 0.75 | 50.50 | 50.00 | 27.50 | 32.50 | 17.00 | 0.00 | 93.07 | 93.29 | 88.14 | 83.64 | 99.71 | 100.00 |
| Ord.0.40Load.125Cross.8Var.500Size.2Fac.0.50Fcor | 0.39 | 0.39 | 0.23 | 0.26 | 0.82 | 0.66 | 57.50 | 57.50 | 49.00 | 54.00 | 75.50 | 0.00 | 86.36 | 86.36 | 84.36 | 83.93 | 97.86 | 100.00 |
| Ord.0.40Load.125Cross.8Var.500Size.2Fac.0.70Fcor | 0.21 | 0.21 | 0.10 | 0.18 | 0.60 | 0.15 | 56.50 | 56.50 | 45.50 | 62.50 | 77.00 | 0.00 | 87.64 | 87.64 | 83.21 | 87.36 | 95.71 | 100.00 |
| Ord.0.40Load.125Cross.8Var.1000Size.2Fac.0.00Fcor | 0.84 | 0.84 | 0.48 | 0.42 | 0.74 | 0.75 | 63.00 | 62.50 | 24.50 | 27.50 | 14.00 | 0.00 | 97.64 | 97.50 | 89.14 | 86.00 | 99.43 | 100.00 |
| Ord.0.40Load.125Cross.8Var.1000Size.2Fac.0.50Fcor | 0.59 | 0.59 | 0.34 | 0.37 | 0.94 | 0.75 | 79.00 | 79.00 | 61.50 | 70.00 | 89.50 | 0.00 | 86.29 | 86.29 | 84.29 | 84.93 | 98.79 | 100.00 |
| Ord.0.40Load.125Cross.8Var.1000Size.2Fac.0.70Fcor | 0.30 | 0.30 | 0.18 | 0.22 | 0.84 | 0.32 | 60.00 | 60.00 | 63.00 | 65.50 | 96.50 | 0.00 | 86.57 | 86.57 | 83.86 | 86.00 | 96.36 | 100.00 |
| Ord.0.40Load.125Cross.8Var.300Size.4Fac.0.00Fcor | 0.12 | 0.12 | 0.09 | 0.10 | 0.25 | 0.82 | 5.25 | 5.25 | 2.25 | 3.50 | 0.50 | 0.00 | 97.64 | 97.64 | 98.07 | 97.39 | 100.00 | 100.00 |
| Ord.0.40Load.125Cross.8Var.300Size.4Fac.0.50Fcor | 0.13 | 0.13 | 0.10 | 0.12 | 0.28 | 0.43 | 29.00 | 29.00 | 26.50 | 32.75 | 15.75 | 0.00 | 92.96 | 92.96 | 92.86 | 91.43 | 99.11 | 100.00 |
| Ord.0.40Load.125Cross.8Var.300Size.4Fac.0.70Fcor | 0.11 | 0.11 | 0.09 | 0.10 | 0.21 | 0.03 | 42.50 | 42.50 | 42.75 | 49.25 | 27.50 | 0.00 | 88.36 | 88.36 | 87.29 | 85.25 | 98.18 | 100.00 |
| Ord.0.40Load.125Cross.8Var.500Size.4Fac.0.00Fcor | 0.65 | 0.65 | 0.41 | 0.54 | 0.82 | 0.84 | 41.75 | 41.75 | 22.00 | 24.25 | 2.00 | 0.00 | 89.64 | 89.64 | 93.14 | 88.14 | 100.00 | 100.00 |
| Ord.0.40Load.125Cross.8Var.500Size.4Fac.0.50Fcor | 0.30 | 0.30 | 0.17 | 0.29 | 0.63 | 0.68 | 56.50 | 56.50 | 44.25 | 62.75 | 26.25 | 0.00 | 82.32 | 82.32 | 84.93 | 79.21 | 98.86 | 100.00 |
| Ord.0.40Load.125Cross.8Var.500Size.4Fac.0.70Fcor | 0.16 | 0.16 | 0.09 | 0.15 | 0.33 | 0.05 | 54.25 | 54.25 | 52.25 | 66.50 | 32.00 | 0.00 | 84.32 | 84.32 | 83.21 | 78.93 | 98.68 | 100.00 |
| Ord.0.40Load.125Cross.8Var.1000Size.4Fac.0.00Fcor | 0.90 | 0.90 | 0.51 | 0.67 | 0.84 | 0.84 | 62.75 | 62.50 | 31.00 | 29.00 | 3.50 | 0.00 | 97.82 | 97.82 | 95.54 | 91.61 | 100.00 | 100.00 |
| Ord.0.40Load.125Cross.8Var.1000Size.4Fac.0.50Fcor | 0.42 | 0.42 | 0.19 | 0.40 | 0.81 | 0.82 | 70.00 | 70.00 | 48.00 | 70.50 | 45.25 | 0.00 | 86.29 | 86.29 | 88.36 | 83.61 | 99.36 | 100.00 |
| Ord.0.40Load.125Cross.8Var.1000Size.4Fac.0.70Fcor | 0.16 | 0.16 | 0.09 | 0.16 | 0.57 | 0.25 | 56.00 | 56.00 | 49.00 | 61.25 | 41.75 | 0.00 | 87.04 | 87.04 | 85.18 | 84.14 | 98.54 | 100.00 |
| Ord.0.40Load.Cross250.4Var.300Size.2Fac.0.00Fcor | 0.18 | 0.18 | 0.13 | 0.12 | 0.28 | 0.40 | 10.00 | 11.00 | 13.00 | 11.00 | 20.00 | 0.00 | 96.00 | 95.33 | 93.50 | 95.50 | 94.00 | 100.00 |
| Ord.0.40Load.Cross250.4Var.300Size.2Fac.0.50Fcor | 0.22 | 0.21 | 0.13 | 0.12 | 0.32 | 0.07 | 22.50 | 22.50 | 25.00 | 22.50 | 44.50 | 0.00 | 95.17 | 95.17 | 93.67 | 94.50 | 92.00 | 100.00 |
| Ord.0.40Load.Cross250.4Var.300Size.2Fac.0.70Fcor | 0.15 | 0.15 | 0.08 | 0.08 | 0.18 | 0.01 | 23.50 | 25.00 | 25.00 | 22.00 | 34.00 | 0.00 | 93.17 | 92.83 | 92.17 | 94.83 | 93.17 | 100.00 |
| Ord.0.40Load.Cross250.4Var.500Size.2Fac.0.00Fcor | 0.39 | 0.39 | 0.30 | 0.26 | 0.51 | 0.49 | 28.50 | 28.50 | 28.00 | 21.50 | 31.50 | 0.00 | 94.83 | 94.50 | 93.17 | 94.17 | 95.17 | 100.00 |
| Ord.0.40Load.Cross250.4Var.500Size.2Fac.0.50Fcor | 0.31 | 0.31 | 0.21 | 0.21 | 0.46 | 0.11 | 33.50 | 33.50 | 35.00 | 31.50 | 57.50 | 0.00 | 94.00 | 94.00 | 92.83 | 95.17 | 92.67 | 100.00 |
| Ord.0.40Load.Cross250.4Var.500Size.2Fac.0.70Fcor | 0.19 | 0.19 | 0.13 | 0.11 | 0.20 | 0.00 | 25.00 | 25.00 | 30.50 | 22.50 | 26.50 | 0.00 | 93.50 | 93.50 | 91.83 | 94.67 | 97.67 | 100.00 |
| Ord.0.40Load.Cross250.4Var.1000Size.2Fac.0.00Fcor | 0.60 | 0.60 | 0.46 | 0.44 | 0.67 | 0.53 | 56.00 | 56.00 | 44.50 | 41.50 | 52.00 | 0.00 | 94.17 | 94.17 | 91.50 | 94.67 | 96.50 | 100.00 |
| Ord.0.40Load.Cross250.4Var.1000Size.2Fac.0.50Fcor | 0.41 | 0.41 | 0.31 | 0.29 | 0.56 | 0.15 | 42.00 | 42.00 | 45.50 | 37.50 | 71.00 | 0.00 | 95.33 | 95.33 | 91.83 | 95.17 | 92.17 | 100.00 |
| Ord.0.40Load.Cross250.4Var.1000Size.2Fac.0.70Fcor | 0.16 | 0.16 | 0.13 | 0.12 | 0.11 | 0.01 | 20.50 | 20.50 | 20.50 | 18.00 | 16.00 | 0.00 | 95.83 | 95.83 | 95.67 | 96.50 | 97.50 | 100.00 |
| Ord.0.40Load.Cross250.4Var.300Size.4Fac.0.00Fcor | 0.07 | 0.07 | 0.06 | 0.07 | 0.18 | 0.56 | 2.00 | 1.50 | 2.25 | 2.50 | 0.25 | 0.00 | 99.25 | 99.33 | 99.08 | 99.08 | 99.83 | 100.00 |
| Ord.0.40Load.Cross250.4Var.300Size.4Fac.0.50Fcor | 0.21 | 0.21 | 0.14 | 0.19 | 0.29 | 0.11 | 30.50 | 30.75 | 35.00 | 40.00 | 29.50 | 0.00 | 90.00 | 90.25 | 87.17 | 88.83 | 95.67 | 100.00 |
| Ord.0.40Load.Cross250.4Var.300Size.4Fac.0.70Fcor | 0.14 | 0.14 | 0.10 | 0.14 | 0.20 | 0.00 | 37.00 | 37.00 | 36.25 | 43.75 | 46.00 | 0.00 | 89.83 | 89.83 | 87.83 | 89.33 | 95.00 | 100.00 |
| Ord.0.40Load.Cross250.4Var.500Size.4Fac.0.00Fcor | 0.37 | 0.37 | 0.31 | 0.36 | 0.54 | 0.66 | 19.75 | 20.00 | 17.75 | 18.50 | 8.25 | 0.00 | 93.58 | 93.83 | 94.25 | 94.58 | 99.42 | 100.00 |
| Ord.0.40Load.Cross250.4Var.500Size.4Fac.0.50Fcor | 0.28 | 0.28 | 0.17 | 0.25 | 0.41 | 0.16 | 39.25 | 38.25 | 36.25 | 44.75 | 43.75 | 0.00 | 91.17 | 91.67 | 88.92 | 89.58 | 97.08 | 100.00 |
| Ord.0.40Load.Cross250.4Var.500Size.4Fac.0.70Fcor | 0.16 | 0.16 | 0.12 | 0.14 | 0.25 | 0.00 | 30.25 | 30.25 | 31.50 | 36.75 | 46.50 | 0.00 | 91.67 | 91.67 | 90.50 | 92.58 | 96.33 | 100.00 |
| Ord.0.40Load.Cross250.4Var.1000Size.4Fac.0.00Fcor | 0.66 | 0.66 | 0.51 | 0.64 | 0.71 | 0.69 | 49.25 | 49.50 | 37.00 | 44.25 | 15.25 | 0.00 | 94.75 | 94.75 | 95.67 | 94.50 | 99.92 | 100.00 |
| Ord.0.40Load.Cross250.4Var.1000Size.4Fac.0.50Fcor | 0.29 | 0.29 | 0.22 | 0.28 | 0.53 | 0.30 | 41.00 | 41.00 | 44.00 | 43.50 | 56.25 | 0.00 | 93.58 | 93.58 | 90.58 | 92.42 | 97.75 | 100.00 |
| Ord.0.40Load.Cross250.4Var.1000Size.4Fac.0.70Fcor | 0.12 | 0.12 | 0.11 | 0.12 | 0.29 | 0.01 | 21.50 | 21.50 | 31.75 | 32.50 | 51.50 | 0.00 | 95.00 | 95.00 | 93.83 | 93.08 | 97.00 | 100.00 |
| Ord.0.40Load.Cross250.8Var.300Size.2Fac.0.00Fcor | 0.29 | 0.29 | 0.18 | 0.19 | 0.42 | 0.74 | 20.50 | 21.00 | 11.50 | 14.00 | 6.50 | 0.00 | 93.29 | 93.50 | 91.79 | 91.36 | 99.14 | 100.00 |
| Ord.0.40Load.Cross250.8Var.300Size.2Fac.0.50Fcor | 0.25 | 0.25 | 0.16 | 0.21 | 0.52 | 0.46 | 44.50 | 45.00 | 37.00 | 54.50 | 47.50 | 0.00 | 87.50 | 87.36 | 84.00 | 84.36 | 95.86 | 100.00 |
| Ord.0.40Load.Cross250.8Var.300Size.2Fac.0.70Fcor | 0.17 | 0.17 | 0.10 | 0.16 | 0.37 | 0.07 | 53.00 | 53.00 | 47.00 | 63.00 | 59.50 | 0.00 | 86.14 | 86.14 | 81.64 | 81.64 | 95.36 | 100.00 |
| Ord.0.40Load.Cross250.8Var.500Size.2Fac.0.00Fcor | 0.68 | 0.68 | 0.41 | 0.40 | 0.73 | 0.75 | 50.50 | 50.00 | 27.50 | 32.50 | 17.00 | 0.00 | 93.07 | 93.29 | 88.14 | 83.64 | 99.71 | 100.00 |
| Ord.0.40Load.Cross250.8Var.500Size.2Fac.0.50Fcor | 0.39 | 0.39 | 0.23 | 0.26 | 0.82 | 0.66 | 57.50 | 57.50 | 49.00 | 54.00 | 75.50 | 0.00 | 86.36 | 86.36 | 84.36 | 83.93 | 97.86 | 100.00 |
| Ord.0.40Load.Cross250.8Var.500Size.2Fac.0.70Fcor | 0.21 | 0.21 | 0.10 | 0.18 | 0.60 | 0.15 | 56.50 | 56.50 | 45.50 | 62.50 | 77.00 | 0.00 | 87.64 | 87.64 | 83.21 | 87.36 | 95.71 | 100.00 |
| Ord.0.40Load.Cross250.8Var.1000Size.2Fac.0.00Fcor | 0.84 | 0.84 | 0.48 | 0.42 | 0.74 | 0.75 | 63.00 | 62.50 | 24.50 | 27.50 | 14.00 | 0.00 | 97.64 | 97.50 | 89.14 | 86.00 | 99.43 | 100.00 |
| Ord.0.40Load.Cross250.8Var.1000Size.2Fac.0.50Fcor | 0.59 | 0.59 | 0.34 | 0.37 | 0.94 | 0.75 | 79.00 | 79.00 | 61.50 | 70.00 | 89.50 | 0.00 | 86.29 | 86.29 | 84.29 | 84.93 | 98.79 | 100.00 |
| Ord.0.40Load.Cross250.8Var.1000Size.2Fac.0.70Fcor | 0.30 | 0.30 | 0.18 | 0.22 | 0.84 | 0.32 | 60.00 | 60.00 | 63.00 | 65.50 | 96.50 | 0.00 | 86.57 | 86.57 | 83.86 | 86.00 | 96.36 | 100.00 |
| Ord.0.40Load.Cross250.8Var.300Size.4Fac.0.00Fcor | 0.05 | 0.05 | 0.03 | 0.04 | 0.11 | 0.68 | 2.38 | 2.38 | 2.50 | 1.62 | 0.38 | 0.00 | 98.83 | 98.83 | 98.96 | 98.88 | 99.92 | 100.00 |
| Ord.0.40Load.Cross250.8Var.300Size.4Fac.0.50Fcor | 0.19 | 0.19 | 0.14 | 0.18 | 0.34 | 0.33 | 39.88 | 39.88 | 32.75 | 45.88 | 16.62 | 0.00 | 89.96 | 89.96 | 90.71 | 88.33 | 99.00 | 100.00 |
| Ord.0.40Load.Cross250.8Var.300Size.4Fac.0.70Fcor | 0.15 | 0.15 | 0.12 | 0.14 | 0.24 | 0.02 | 38.38 | 38.38 | 36.88 | 45.75 | 21.62 | 0.00 | 89.96 | 89.96 | 87.62 | 87.21 | 98.79 | 100.00 |
| Ord.0.40Load.Cross250.8Var.500Size.4Fac.0.00Fcor | 0.46 | 0.46 | 0.30 | 0.40 | 0.58 | 0.69 | 32.25 | 31.00 | 19.25 | 26.38 | 1.50 | 0.00 | 88.33 | 88.12 | 91.17 | 85.88 | 99.88 | 100.00 |
| Ord.0.40Load.Cross250.8Var.500Size.4Fac.0.50Fcor | 0.26 | 0.26 | 0.17 | 0.24 | 0.53 | 0.52 | 50.25 | 49.88 | 44.88 | 57.12 | 20.25 | 0.00 | 85.83 | 86.17 | 83.29 | 81.83 | 99.29 | 100.00 |
| Ord.0.40Load.Cross250.8Var.500Size.4Fac.0.70Fcor | 0.15 | 0.15 | 0.12 | 0.16 | 0.30 | 0.01 | 41.62 | 41.62 | 40.88 | 53.00 | 22.62 | 0.00 | 88.83 | 88.83 | 88.21 | 85.58 | 99.17 | 100.00 |
| Ord.0.40Load.Cross250.8Var.1000Size.4Fac.0.00Fcor | 0.76 | 0.76 | 0.42 | 0.60 | 0.67 | 0.69 | 54.38 | 54.38 | 35.00 | 38.12 | 2.62 | 0.00 | 95.04 | 95.04 | 94.50 | 88.71 | 99.96 | 100.00 |
| Ord.0.40Load.Cross250.8Var.1000Size.4Fac.0.50Fcor | 0.30 | 0.30 | 0.16 | 0.28 | 0.63 | 0.68 | 50.38 | 50.38 | 38.00 | 53.88 | 29.25 | 0.00 | 90.04 | 90.04 | 89.88 | 88.92 | 99.54 | 100.00 |
| Ord.0.40Load.Cross250.8Var.1000Size.4Fac.0.70Fcor | 0.16 | 0.16 | 0.12 | 0.16 | 0.43 | 0.12 | 37.62 | 37.62 | 40.38 | 48.00 | 29.38 | 0.00 | 93.00 | 93.00 | 91.83 | 90.92 | 99.38 | 100.00 |
| Ord.0.55Load.000Cross.4Var.300Size.2Fac.0.00Fcor | 0.99 | 1.00 | 0.80 | 0.78 | 1.00 | 1.00 | - | - | - | - | - | - | - | - | - | - | - | - |
| Ord.0.55Load.000Cross.4Var.300Size.2Fac.0.50Fcor | 0.84 | 0.84 | 0.57 | 0.68 | 0.72 | 0.72 | - | - | - | - | - | - | - | - | - | - | - | - |
| Ord.0.55Load.000Cross.4Var.300Size.2Fac.0.70Fcor | 0.52 | 0.52 | 0.39 | 0.44 | 0.30 | 0.11 | - | - | - | - | - | - | - | - | - | - | - | - |
| Ord.0.55Load.000Cross.4Var.500Size.2Fac.0.00Fcor | 1.00 | 1.00 | 0.84 | 0.84 | 1.00 | 1.00 | - | - | - | - | - | - | - | - | - | - | - | - |
| Ord.0.55Load.000Cross.4Var.500Size.2Fac.0.50Fcor | 0.95 | 0.95 | 0.65 | 0.75 | 0.82 | 0.95 | - | - | - | - | - | - | - | - | - | - | - | - |
| Ord.0.55Load.000Cross.4Var.500Size.2Fac.0.70Fcor | 0.67 | 0.67 | 0.54 | 0.59 | 0.36 | 0.17 | - | - | - | - | - | - | - | - | - | - | - | - |
| Ord.0.55Load.000Cross.4Var.1000Size.2Fac.0.00Fcor | 0.99 | 1.00 | 0.87 | 0.87 | 1.00 | 1.00 | - | - | - | - | - | - | - | - | - | - | - | - |
| Ord.0.55Load.000Cross.4Var.1000Size.2Fac.0.50Fcor | 0.97 | 0.97 | 0.71 | 0.83 | 0.83 | 0.99 | - | - | - | - | - | - | - | - | - | - | - | - |
| Ord.0.55Load.000Cross.4Var.1000Size.2Fac.0.70Fcor | 0.75 | 0.75 | 0.62 | 0.71 | 0.37 | 0.25 | - | - | - | - | - | - | - | - | - | - | - | - |
| Ord.0.55Load.000Cross.4Var.300Size.4Fac.0.00Fcor | 0.98 | 0.98 | 0.70 | 0.91 | 1.00 | 1.00 | - | - | - | - | - | - | - | - | - | - | - | - |
| Ord.0.55Load.000Cross.4Var.300Size.4Fac.0.50Fcor | 0.64 | 0.64 | 0.31 | 0.60 | 0.84 | 0.60 | - | - | - | - | - | - | - | - | - | - | - | - |
| Ord.0.55Load.000Cross.4Var.300Size.4Fac.0.70Fcor | 0.38 | 0.38 | 0.13 | 0.35 | 0.43 | 0.01 | - | - | - | - | - | - | - | - | - | - | - | - |
| Ord.0.55Load.000Cross.4Var.500Size.4Fac.0.00Fcor | 0.99 | 1.00 | 0.76 | 0.94 | 1.00 | 1.00 | - | - | - | - | - | - | - | - | - | - | - | - |
| Ord.0.55Load.000Cross.4Var.500Size.4Fac.0.50Fcor | 0.81 | 0.81 | 0.30 | 0.74 | 0.89 | 0.81 | - | - | - | - | - | - | - | - | - | - | - | - |
| Ord.0.55Load.000Cross.4Var.500Size.4Fac.0.70Fcor | 0.54 | 0.54 | 0.17 | 0.45 | 0.56 | 0.04 | - | - | - | - | - | - | - | - | - | - | - | - |
| Ord.0.55Load.000Cross.4Var.1000Size.4Fac.0.00Fcor | 0.98 | 1.00 | 0.72 | 0.94 | 1.00 | 1.00 | - | - | - | - | - | - | - | - | - | - | - | - |
| Ord.0.55Load.000Cross.4Var.1000Size.4Fac.0.50Fcor | 0.93 | 0.93 | 0.26 | 0.80 | 0.93 | 0.95 | - | - | - | - | - | - | - | - | - | - | - | - |
| Ord.0.55Load.000Cross.4Var.1000Size.4Fac.0.70Fcor | 0.64 | 0.64 | 0.21 | 0.60 | 0.73 | 0.09 | - | - | - | - | - | - | - | - | - | - | - | - |
| Ord.0.55Load.000Cross.8Var.300Size.2Fac.0.00Fcor | 0.99 | 1.00 | 0.59 | 0.55 | 1.00 | 1.00 | - | - | - | - | - | - | - | - | - | - | - | - |
| Ord.0.55Load.000Cross.8Var.300Size.2Fac.0.50Fcor | 0.86 | 0.86 | 0.50 | 0.51 | 0.98 | 1.00 | - | - | - | - | - | - | - | - | - | - | - | - |
| Ord.0.55Load.000Cross.8Var.300Size.2Fac.0.70Fcor | 0.65 | 0.65 | 0.33 | 0.43 | 0.87 | 0.71 | - | - | - | - | - | - | - | - | - | - | - | - |
| Ord.0.55Load.000Cross.8Var.500Size.2Fac.0.00Fcor | 0.98 | 1.00 | 0.63 | 0.57 | 1.00 | 1.00 | - | - | - | - | - | - | - | - | - | - | - | - |
| Ord.0.55Load.000Cross.8Var.500Size.2Fac.0.50Fcor | 0.96 | 0.96 | 0.53 | 0.52 | 1.00 | 1.00 | - | - | - | - | - | - | - | - | - | - | - | - |
| Ord.0.55Load.000Cross.8Var.500Size.2Fac.0.70Fcor | 0.78 | 0.78 | 0.43 | 0.47 | 0.93 | 0.96 | - | - | - | - | - | - | - | - | - | - | - | - |
| Ord.0.55Load.000Cross.8Var.1000Size.2Fac.0.00Fcor | 0.99 | 1.00 | 0.64 | 0.60 | 1.00 | 1.00 | - | - | - | - | - | - | - | - | - | - | - | - |
| Ord.0.55Load.000Cross.8Var.1000Size.2Fac.0.50Fcor | 0.98 | 0.98 | 0.54 | 0.60 | 1.00 | 1.00 | - | - | - | - | - | - | - | - | - | - | - | - |
| Ord.0.55Load.000Cross.8Var.1000Size.2Fac.0.70Fcor | 0.87 | 0.87 | 0.48 | 0.50 | 0.97 | 0.99 | - | - | - | - | - | - | - | - | - | - | - | - |
| Ord.0.55Load.000Cross.8Var.300Size.4Fac.0.00Fcor | 0.99 | 1.00 | 0.53 | 0.70 | 1.00 | 1.00 | - | - | - | - | - | - | - | - | - | - | - | - |
| Ord.0.55Load.000Cross.8Var.300Size.4Fac.0.50Fcor | 0.72 | 0.72 | 0.29 | 0.64 | 0.98 | 0.98 | - | - | - | - | - | - | - | - | - | - | - | - |
| Ord.0.55Load.000Cross.8Var.300Size.4Fac.0.70Fcor | 0.47 | 0.47 | 0.10 | 0.43 | 0.82 | 0.31 | - | - | - | - | - | - | - | - | - | - | - | - |
| Ord.0.55Load.000Cross.8Var.500Size.4Fac.0.00Fcor | 0.99 | 1.00 | 0.61 | 0.76 | 1.00 | 1.00 | - | - | - | - | - | - | - | - | - | - | - | - |
| Ord.0.55Load.000Cross.8Var.500Size.4Fac.0.50Fcor | 0.88 | 0.88 | 0.31 | 0.71 | 1.00 | 1.00 | - | - | - | - | - | - | - | - | - | - | - | - |
| Ord.0.55Load.000Cross.8Var.500Size.4Fac.0.70Fcor | 0.63 | 0.63 | 0.17 | 0.56 | 0.96 | 0.70 | - | - | - | - | - | - | - | - | - | - | - | - |
| Ord.0.55Load.000Cross.8Var.1000Size.4Fac.0.00Fcor | 0.99 | 1.00 | 0.62 | 0.80 | 1.00 | 1.00 | - | - | - | - | - | - | - | - | - | - | - | - |
| Ord.0.55Load.000Cross.8Var.1000Size.4Fac.0.50Fcor | 0.98 | 0.98 | 0.33 | 0.71 | 1.00 | 1.00 | - | - | - | - | - | - | - | - | - | - | - | - |
| Ord.0.55Load.000Cross.8Var.1000Size.4Fac.0.70Fcor | 0.83 | 0.83 | 0.17 | 0.63 | 1.00 | 0.98 | - | - | - | - | - | - | - | - | - | - | - | - |
| Ord.0.55Load.125Cross.4Var.300Size.2Fac.0.00Fcor | 0.86 | 0.86 | 0.75 | 0.54 | 0.88 | 0.76 | 64.00 | 63.00 | 61.00 | 22.00 | 82.00 | 3.00 | 97.43 | 97.43 | 94.14 | 93.57 | 95.57 | 100.00 |
| Ord.0.55Load.125Cross.4Var.300Size.2Fac.0.50Fcor | 0.74 | 0.74 | 0.58 | 0.49 | 0.63 | 0.47 | 83.00 | 83.00 | 77.00 | 58.00 | 94.00 | 3.00 | 92.29 | 92.29 | 88.00 | 90.57 | 85.00 | 100.00 |
| Ord.0.55Load.125Cross.4Var.300Size.2Fac.0.70Fcor | 0.52 | 0.52 | 0.39 | 0.41 | 0.24 | 0.02 | 74.00 | 73.00 | 77.00 | 68.00 | 43.00 | 0.00 | 90.43 | 90.57 | 85.71 | 90.00 | 91.29 | 100.00 |
| Ord.0.55Load.125Cross.4Var.500Size.2Fac.0.00Fcor | 0.91 | 0.91 | 0.77 | 0.56 | 0.94 | 0.76 | 73.00 | 71.00 | 54.00 | 18.00 | 92.00 | 2.00 | 99.29 | 99.43 | 95.43 | 96.57 | 97.29 | 100.00 |
| Ord.0.55Load.125Cross.4Var.500Size.2Fac.0.50Fcor | 0.81 | 0.81 | 0.72 | 0.60 | 0.72 | 0.61 | 90.00 | 90.00 | 91.00 | 71.00 | 99.00 | 1.00 | 94.71 | 94.71 | 92.14 | 93.00 | 88.14 | 100.00 |
| Ord.0.55Load.125Cross.4Var.500Size.2Fac.0.70Fcor | 0.50 | 0.50 | 0.42 | 0.42 | 0.18 | 0.04 | 72.00 | 72.00 | 75.00 | 62.00 | 36.00 | 0.00 | 90.14 | 90.14 | 87.86 | 92.86 | 91.71 | 100.00 |
| Ord.0.55Load.125Cross.4Var.1000Size.2Fac.0.00Fcor | 0.93 | 0.93 | 0.80 | 0.59 | 0.98 | 0.76 | 76.00 | 75.00 | 70.00 | 18.00 | 98.00 | 1.00 | 99.71 | 100.00 | 97.29 | 98.57 | 99.29 | 100.00 |
| Ord.0.55Load.125Cross.4Var.1000Size.2Fac.0.50Fcor | 0.86 | 0.86 | 0.74 | 0.61 | 0.75 | 0.68 | 96.00 | 96.00 | 91.00 | 66.00 | 98.00 | 1.00 | 95.57 | 95.57 | 92.43 | 95.57 | 89.57 | 100.00 |
| Ord.0.55Load.125Cross.4Var.1000Size.2Fac.0.70Fcor | 0.61 | 0.61 | 0.53 | 0.48 | 0.11 | 0.04 | 78.00 | 78.00 | 82.00 | 69.00 | 17.00 | 0.00 | 92.43 | 92.43 | 91.43 | 95.71 | 97.14 | 100.00 |
| Ord.0.55Load.125Cross.4Var.300Size.4Fac.0.00Fcor | 0.88 | 0.88 | 0.61 | 0.78 | 0.89 | 0.83 | 55.50 | 55.50 | 24.50 | 39.00 | 33.00 | 1.00 | 97.86 | 98.00 | 97.00 | 95.93 | 100.00 | 100.00 |
| Ord.0.55Load.125Cross.4Var.300Size.4Fac.0.50Fcor | 0.57 | 0.57 | 0.35 | 0.56 | 0.75 | 0.47 | 68.50 | 68.50 | 50.50 | 75.00 | 79.50 | 1.50 | 90.07 | 90.07 | 86.64 | 87.79 | 93.86 | 100.00 |
| Ord.0.55Load.125Cross.4Var.300Size.4Fac.0.70Fcor | 0.35 | 0.35 | 0.20 | 0.35 | 0.42 | 0.01 | 64.00 | 64.00 | 54.50 | 67.00 | 74.00 | 0.00 | 85.79 | 85.79 | 82.64 | 83.21 | 90.50 | 100.00 |
| Ord.0.55Load.125Cross.4Var.500Size.4Fac.0.00Fcor | 0.95 | 0.95 | 0.61 | 0.79 | 0.94 | 0.84 | 72.50 | 73.00 | 29.50 | 38.00 | 64.50 | 2.50 | 99.57 | 99.71 | 95.93 | 97.36 | 99.93 | 100.00 |
| Ord.0.55Load.125Cross.4Var.500Size.4Fac.0.50Fcor | 0.71 | 0.71 | 0.33 | 0.68 | 0.80 | 0.62 | 67.50 | 67.50 | 55.50 | 77.50 | 84.00 | 2.50 | 92.50 | 92.50 | 86.00 | 87.50 | 96.00 | 100.00 |
| Ord.0.55Load.125Cross.4Var.500Size.4Fac.0.70Fcor | 0.42 | 0.42 | 0.21 | 0.40 | 0.52 | 0.02 | 61.50 | 61.50 | 49.00 | 73.00 | 74.50 | 0.00 | 88.29 | 88.29 | 80.64 | 83.64 | 92.36 | 100.00 |
| Ord.0.55Load.125Cross.4Var.1000Size.4Fac.0.00Fcor | 0.95 | 0.95 | 0.56 | 0.80 | 0.98 | 0.83 | 73.50 | 74.00 | 33.00 | 39.50 | 91.50 | 0.00 | 99.57 | 99.64 | 95.79 | 98.43 | 100.00 | 100.00 |
| Ord.0.55Load.125Cross.4Var.1000Size.4Fac.0.50Fcor | 0.82 | 0.82 | 0.33 | 0.79 | 0.89 | 0.76 | 86.00 | 86.00 | 50.00 | 84.00 | 91.00 | 2.50 | 94.57 | 94.57 | 85.43 | 93.93 | 97.79 | 100.00 |
| Ord.0.55Load.125Cross.4Var.1000Size.4Fac.0.70Fcor | 0.47 | 0.47 | 0.30 | 0.46 | 0.62 | 0.05 | 62.00 | 62.00 | 49.50 | 77.00 | 82.50 | 0.00 | 87.86 | 87.86 | 83.79 | 85.93 | 94.07 | 100.00 |
| Ord.0.55Load.125Cross.8Var.300Size.2Fac.0.00Fcor | 0.84 | 0.84 | 0.44 | 0.38 | 0.77 | 0.76 | 51.50 | 51.00 | 17.00 | 11.50 | 25.00 | 3.00 | 98.29 | 98.36 | 88.29 | 82.64 | 98.86 | 100.00 |
| Ord.0.55Load.125Cross.8Var.300Size.2Fac.0.50Fcor | 0.79 | 0.79 | 0.38 | 0.34 | 0.95 | 0.76 | 85.00 | 85.00 | 55.50 | 56.00 | 89.00 | 2.50 | 92.36 | 92.36 | 80.07 | 80.21 | 98.86 | 100.00 |
| Ord.0.55Load.125Cross.8Var.300Size.2Fac.0.70Fcor | 0.49 | 0.49 | 0.20 | 0.31 | 0.86 | 0.35 | 78.50 | 78.50 | 58.50 | 76.00 | 94.00 | 0.50 | 84.71 | 84.71 | 79.79 | 80.71 | 96.86 | 100.00 |
| Ord.0.55Load.125Cross.8Var.500Size.2Fac.0.00Fcor | 0.85 | 0.83 | 0.48 | 0.41 | 0.79 | 0.75 | 47.50 | 43.00 | 7.50 | 8.00 | 34.50 | 0.00 | 99.86 | 99.86 | 91.43 | 86.86 | 99.29 | 100.00 |
| Ord.0.55Load.125Cross.8Var.500Size.2Fac.0.50Fcor | 0.91 | 0.91 | 0.43 | 0.43 | 0.98 | 0.76 | 92.00 | 92.00 | 58.50 | 62.50 | 96.00 | 1.50 | 96.93 | 96.93 | 82.07 | 82.79 | 99.71 | 100.00 |
| Ord.0.55Load.125Cross.8Var.500Size.2Fac.0.70Fcor | 0.64 | 0.64 | 0.31 | 0.36 | 0.93 | 0.52 | 86.00 | 86.00 | 67.00 | 73.00 | 97.50 | 0.00 | 86.93 | 86.93 | 81.29 | 82.43 | 97.29 | 100.00 |
| Ord.0.55Load.125Cross.8Var.1000Size.2Fac.0.00Fcor | 0.91 | 0.89 | 0.52 | 0.43 | 0.87 | 0.76 | 70.00 | 64.50 | 22.00 | 14.00 | 62.00 | 0.50 | 100.00 | 100.00 | 92.07 | 88.64 | 99.57 | 100.00 |
| Ord.0.55Load.125Cross.8Var.1000Size.2Fac.0.50Fcor | 0.98 | 0.98 | 0.50 | 0.49 | 1.00 | 0.76 | 99.50 | 99.50 | 64.00 | 62.50 | 100.00 | 1.00 | 99.00 | 99.00 | 86.71 | 88.14 | 99.93 | 100.00 |
| Ord.0.55Load.125Cross.8Var.1000Size.2Fac.0.70Fcor | 0.78 | 0.78 | 0.37 | 0.42 | 0.98 | 0.73 | 95.00 | 95.00 | 65.00 | 74.00 | 100.00 | 0.50 | 91.57 | 91.57 | 87.21 | 88.50 | 99.29 | 100.00 |
| Ord.0.55Load.125Cross.8Var.300Size.4Fac.0.00Fcor | 0.93 | 0.93 | 0.43 | 0.58 | 0.86 | 0.84 | 68.00 | 67.50 | 27.75 | 13.75 | 12.00 | 3.75 | 98.86 | 98.93 | 94.46 | 86.57 | 100.00 | 100.00 |
| Ord.0.55Load.125Cross.8Var.300Size.4Fac.0.50Fcor | 0.67 | 0.67 | 0.25 | 0.56 | 0.92 | 0.83 | 79.75 | 79.75 | 49.50 | 72.25 | 72.75 | 3.00 | 86.07 | 86.07 | 85.14 | 79.61 | 99.57 | 100.00 |
| Ord.0.55Load.125Cross.8Var.300Size.4Fac.0.70Fcor | 0.41 | 0.41 | 0.16 | 0.38 | 0.72 | 0.23 | 73.75 | 73.50 | 55.75 | 76.25 | 49.50 | 1.50 | 78.68 | 79.14 | 78.54 | 76.61 | 99.21 | 100.00 |
| Ord.0.55Load.125Cross.8Var.500Size.4Fac.0.00Fcor | 0.95 | 0.95 | 0.54 | 0.63 | 0.87 | 0.84 | 69.50 | 69.50 | 42.00 | 14.25 | 19.75 | 3.00 | 99.82 | 99.86 | 96.61 | 88.18 | 100.00 | 100.00 |
| Ord.0.55Load.125Cross.8Var.500Size.4Fac.0.50Fcor | 0.83 | 0.83 | 0.36 | 0.66 | 0.96 | 0.84 | 87.25 | 87.25 | 60.50 | 68.25 | 85.75 | 2.00 | 91.29 | 91.29 | 87.00 | 83.61 | 99.89 | 100.00 |
| Ord.0.55Load.125Cross.8Var.500Size.4Fac.0.70Fcor | 0.56 | 0.56 | 0.19 | 0.49 | 0.90 | 0.46 | 81.50 | 81.50 | 60.00 | 78.25 | 77.00 | 0.50 | 82.32 | 82.32 | 81.14 | 79.82 | 99.68 | 100.00 |
| Ord.0.55Load.125Cross.8Var.1000Size.4Fac.0.00Fcor | 0.95 | 0.95 | 0.56 | 0.63 | 0.91 | 0.84 | 68.50 | 68.50 | 43.50 | 17.50 | 44.25 | 0.50 | 99.82 | 100.00 | 95.32 | 92.64 | 100.00 | 100.00 |
| Ord.0.55Load.125Cross.8Var.1000Size.4Fac.0.50Fcor | 0.95 | 0.95 | 0.35 | 0.67 | 0.98 | 0.84 | 94.25 | 94.25 | 60.75 | 71.00 | 95.75 | 1.25 | 97.89 | 97.89 | 90.00 | 86.61 | 99.93 | 100.00 |
| Ord.0.55Load.125Cross.8Var.1000Size.4Fac.0.70Fcor | 0.76 | 0.76 | 0.29 | 0.62 | 0.97 | 0.77 | 91.75 | 91.75 | 63.25 | 85.00 | 90.75 | 0.25 | 89.61 | 89.61 | 83.54 | 85.36 | 99.86 | 100.00 |
| Ord.0.55Load.Cross250.4Var.300Size.2Fac.0.00Fcor | 0.61 | 0.61 | 0.48 | 0.37 | 0.59 | 0.53 | 44.00 | 43.50 | 34.50 | 29.50 | 57.50 | 1.50 | 96.17 | 96.17 | 90.67 | 91.00 | 88.83 | 100.00 |
| Ord.0.55Load.Cross250.4Var.300Size.2Fac.0.50Fcor | 0.58 | 0.58 | 0.38 | 0.33 | 0.50 | 0.16 | 60.50 | 60.50 | 53.50 | 41.00 | 73.00 | 1.50 | 94.50 | 94.50 | 89.33 | 94.67 | 88.67 | 100.00 |
| Ord.0.55Load.Cross250.4Var.300Size.2Fac.0.70Fcor | 0.32 | 0.32 | 0.24 | 0.21 | 0.11 | 0.00 | 42.50 | 42.50 | 46.00 | 35.50 | 18.50 | 0.00 | 92.17 | 92.17 | 89.50 | 93.67 | 96.00 | 100.00 |
| Ord.0.55Load.Cross250.4Var.500Size.2Fac.0.00Fcor | 0.72 | 0.71 | 0.49 | 0.45 | 0.70 | 0.53 | 59.00 | 57.50 | 38.00 | 29.50 | 76.50 | 0.00 | 97.17 | 97.50 | 90.50 | 93.17 | 90.00 | 100.00 |
| Ord.0.55Load.Cross250.4Var.500Size.2Fac.0.50Fcor | 0.81 | 0.81 | 0.61 | 0.51 | 0.56 | 0.28 | 84.50 | 84.50 | 75.00 | 59.00 | 80.50 | 0.00 | 96.33 | 96.33 | 89.33 | 94.00 | 88.50 | 100.00 |
| Ord.0.55Load.Cross250.4Var.500Size.2Fac.0.70Fcor | 0.46 | 0.46 | 0.37 | 0.35 | 0.04 | 0.01 | 53.50 | 53.50 | 51.00 | 43.00 | 7.00 | 0.00 | 94.33 | 94.33 | 93.17 | 97.00 | 98.67 | 100.00 |
| Ord.0.55Load.Cross250.4Var.1000Size.2Fac.0.00Fcor | 0.85 | 0.82 | 0.54 | 0.45 | 0.76 | 0.53 | 77.50 | 73.00 | 49.00 | 27.50 | 90.00 | 0.00 | 98.00 | 98.50 | 90.83 | 96.00 | 90.50 | 100.00 |
| Ord.0.55Load.Cross250.4Var.1000Size.2Fac.0.50Fcor | 0.81 | 0.81 | 0.63 | 0.53 | 0.49 | 0.33 | 87.00 | 87.00 | 74.00 | 57.50 | 76.00 | 0.00 | 95.33 | 95.33 | 89.83 | 95.50 | 87.67 | 100.00 |
| Ord.0.55Load.Cross250.4Var.1000Size.2Fac.0.70Fcor | 0.61 | 0.61 | 0.53 | 0.40 | 0.02 | 0.00 | 67.50 | 67.50 | 66.00 | 44.50 | 3.00 | 0.00 | 94.83 | 94.83 | 94.00 | 97.00 | 99.67 | 100.00 |
| Ord.0.55Load.Cross250.4Var.300Size.4Fac.0.00Fcor | 0.77 | 0.77 | 0.53 | 0.66 | 0.77 | 0.70 | 49.00 | 49.00 | 33.50 | 36.50 | 29.75 | 3.25 | 97.33 | 97.67 | 96.08 | 94.50 | 99.33 | 100.00 |
| Ord.0.55Load.Cross250.4Var.300Size.4Fac.0.50Fcor | 0.56 | 0.56 | 0.29 | 0.53 | 0.67 | 0.34 | 65.50 | 65.50 | 51.50 | 69.50 | 76.00 | 0.25 | 91.67 | 91.67 | 88.42 | 90.17 | 95.25 | 100.00 |
| Ord.0.55Load.Cross250.4Var.300Size.4Fac.0.70Fcor | 0.31 | 0.31 | 0.18 | 0.31 | 0.38 | 0.00 | 47.00 | 47.00 | 51.50 | 54.50 | 63.25 | 0.00 | 90.58 | 90.58 | 84.92 | 89.25 | 94.83 | 100.00 |
| Ord.0.55Load.Cross250.4Var.500Size.4Fac.0.00Fcor | 0.89 | 0.89 | 0.58 | 0.75 | 0.87 | 0.70 | 72.50 | 71.50 | 53.75 | 49.00 | 60.25 | 2.00 | 98.92 | 99.33 | 97.00 | 97.17 | 99.83 | 100.00 |
| Ord.0.55Load.Cross250.4Var.500Size.4Fac.0.50Fcor | 0.67 | 0.67 | 0.33 | 0.64 | 0.76 | 0.44 | 73.50 | 72.75 | 59.50 | 69.25 | 85.25 | 1.25 | 92.83 | 92.75 | 89.50 | 91.08 | 96.67 | 100.00 |
| Ord.0.55Load.Cross250.4Var.500Size.4Fac.0.70Fcor | 0.41 | 0.41 | 0.21 | 0.39 | 0.41 | 0.00 | 53.75 | 53.75 | 51.25 | 61.00 | 62.75 | 0.00 | 92.17 | 92.17 | 85.75 | 89.83 | 96.00 | 100.00 |
| Ord.0.55Load.Cross250.4Var.1000Size.4Fac.0.00Fcor | 0.94 | 0.94 | 0.54 | 0.76 | 0.96 | 0.69 | 82.50 | 82.75 | 57.75 | 54.75 | 89.25 | 0.50 | 99.58 | 99.58 | 94.33 | 97.25 | 99.92 | 100.00 |
| Ord.0.55Load.Cross250.4Var.1000Size.4Fac.0.50Fcor | 0.82 | 0.82 | 0.35 | 0.76 | 0.79 | 0.60 | 86.25 | 86.25 | 64.25 | 84.25 | 86.00 | 2.50 | 96.25 | 96.25 | 88.75 | 92.67 | 96.83 | 100.00 |
| Ord.0.55Load.Cross250.4Var.1000Size.4Fac.0.70Fcor | 0.43 | 0.43 | 0.30 | 0.42 | 0.35 | 0.02 | 64.25 | 64.25 | 57.50 | 63.50 | 53.50 | 0.00 | 92.50 | 92.50 | 91.67 | 92.00 | 97.42 | 100.00 |
| Ord.0.55Load.Cross250.8Var.300Size.2Fac.0.00Fcor | 0.84 | 0.84 | 0.44 | 0.38 | 0.77 | 0.76 | 51.50 | 51.00 | 17.00 | 11.50 | 25.00 | 3.00 | 98.29 | 98.36 | 88.29 | 82.64 | 98.86 | 100.00 |
| Ord.0.55Load.Cross250.8Var.300Size.2Fac.0.50Fcor | 0.79 | 0.79 | 0.38 | 0.34 | 0.95 | 0.76 | 85.00 | 85.00 | 55.50 | 56.00 | 89.00 | 2.50 | 92.36 | 92.36 | 80.07 | 80.21 | 98.86 | 100.00 |
| Ord.0.55Load.Cross250.8Var.300Size.2Fac.0.70Fcor | 0.49 | 0.49 | 0.20 | 0.31 | 0.86 | 0.35 | 78.50 | 78.50 | 58.50 | 76.00 | 94.00 | 0.50 | 84.71 | 84.71 | 79.79 | 80.71 | 96.86 | 100.00 |
| Ord.0.55Load.Cross250.8Var.500Size.2Fac.0.00Fcor | 0.85 | 0.83 | 0.48 | 0.41 | 0.79 | 0.75 | 47.50 | 43.00 | 7.50 | 8.00 | 34.50 | 0.00 | 99.86 | 99.86 | 91.43 | 86.86 | 99.29 | 100.00 |
| Ord.0.55Load.Cross250.8Var.500Size.2Fac.0.50Fcor | 0.91 | 0.91 | 0.43 | 0.43 | 0.98 | 0.76 | 92.00 | 92.00 | 58.50 | 62.50 | 96.00 | 1.50 | 96.93 | 96.93 | 82.07 | 82.79 | 99.71 | 100.00 |
| Ord.0.55Load.Cross250.8Var.500Size.2Fac.0.70Fcor | 0.64 | 0.64 | 0.31 | 0.36 | 0.93 | 0.52 | 86.00 | 86.00 | 67.00 | 73.00 | 97.50 | 0.00 | 86.93 | 86.93 | 81.29 | 82.43 | 97.29 | 100.00 |
| Ord.0.55Load.Cross250.8Var.1000Size.2Fac.0.00Fcor | 0.91 | 0.89 | 0.52 | 0.43 | 0.87 | 0.76 | 70.00 | 64.50 | 22.00 | 14.00 | 62.00 | 0.50 | 100.00 | 100.00 | 92.07 | 88.64 | 99.57 | 100.00 |
| Ord.0.55Load.Cross250.8Var.1000Size.2Fac.0.50Fcor | 0.98 | 0.98 | 0.50 | 0.49 | 1.00 | 0.76 | 99.50 | 99.50 | 64.00 | 62.50 | 100.00 | 1.00 | 99.00 | 99.00 | 86.71 | 88.14 | 99.93 | 100.00 |
| Ord.0.55Load.Cross250.8Var.1000Size.2Fac.0.70Fcor | 0.78 | 0.78 | 0.37 | 0.42 | 0.98 | 0.73 | 95.00 | 95.00 | 65.00 | 74.00 | 100.00 | 0.50 | 91.57 | 91.57 | 87.21 | 88.50 | 99.29 | 100.00 |
| Ord.0.55Load.Cross250.8Var.300Size.4Fac.0.00Fcor | 0.81 | 0.81 | 0.45 | 0.48 | 0.70 | 0.70 | 60.00 | 59.62 | 41.12 | 23.88 | 9.62 | 4.00 | 94.58 | 95.04 | 93.42 | 83.21 | 100.00 | 100.00 |
| Ord.0.55Load.Cross250.8Var.300Size.4Fac.0.50Fcor | 0.58 | 0.58 | 0.25 | 0.43 | 0.75 | 0.68 | 69.88 | 70.25 | 46.00 | 58.00 | 45.50 | 4.00 | 85.54 | 85.62 | 83.42 | 82.00 | 99.46 | 100.00 |
| Ord.0.55Load.Cross250.8Var.300Size.4Fac.0.70Fcor | 0.35 | 0.35 | 0.17 | 0.31 | 0.60 | 0.12 | 63.38 | 63.50 | 51.50 | 64.25 | 38.50 | 0.50 | 84.54 | 84.50 | 81.12 | 81.12 | 99.62 | 100.00 |
| Ord.0.55Load.Cross250.8Var.500Size.4Fac.0.00Fcor | 0.89 | 0.89 | 0.46 | 0.52 | 0.71 | 0.70 | 69.75 | 69.38 | 40.62 | 19.75 | 11.12 | 3.38 | 99.17 | 99.25 | 96.88 | 86.38 | 99.83 | 100.00 |
| Ord.0.55Load.Cross250.8Var.500Size.4Fac.0.50Fcor | 0.73 | 0.73 | 0.32 | 0.54 | 0.82 | 0.70 | 84.25 | 84.25 | 49.38 | 66.62 | 58.75 | 2.75 | 87.42 | 87.42 | 86.25 | 79.00 | 99.46 | 100.00 |
| Ord.0.55Load.Cross250.8Var.500Size.4Fac.0.70Fcor | 0.46 | 0.46 | 0.21 | 0.41 | 0.75 | 0.25 | 66.38 | 66.38 | 47.12 | 70.75 | 48.88 | 0.62 | 85.92 | 85.92 | 84.83 | 81.88 | 99.71 | 100.00 |
| Ord.0.55Load.Cross250.8Var.1000Size.4Fac.0.00Fcor | 0.91 | 0.91 | 0.44 | 0.58 | 0.72 | 0.69 | 73.50 | 73.75 | 48.25 | 25.50 | 18.62 | 0.88 | 99.83 | 99.96 | 95.83 | 92.17 | 99.79 | 100.00 |
| Ord.0.55Load.Cross250.8Var.1000Size.4Fac.0.50Fcor | 0.92 | 0.92 | 0.39 | 0.67 | 0.87 | 0.70 | 91.25 | 91.25 | 57.38 | 68.88 | 72.25 | 1.62 | 97.79 | 97.79 | 90.79 | 85.62 | 99.83 | 100.00 |
| Ord.0.55Load.Cross250.8Var.1000Size.4Fac.0.70Fcor | 0.64 | 0.64 | 0.27 | 0.55 | 0.83 | 0.58 | 76.88 | 76.88 | 51.50 | 73.00 | 61.75 | 1.12 | 90.12 | 90.12 | 88.33 | 86.33 | 99.83 | 100.00 |
| Ord.0.70Load.000Cross.4Var.300Size.2Fac.0.00Fcor | 0.99 | 1.00 | 0.80 | 0.82 | 1.00 | 1.00 | - | - | - | - | - | - | - | - | - | - | - | - |
| Ord.0.70Load.000Cross.4Var.300Size.2Fac.0.50Fcor | 1.00 | 1.00 | 0.65 | 0.76 | 0.91 | 0.98 | - | - | - | - | - | - | - | - | - | - | - | - |
| Ord.0.70Load.000Cross.4Var.300Size.2Fac.0.70Fcor | 0.94 | 0.94 | 0.65 | 0.75 | 0.46 | 0.21 | - | - | - | - | - | - | - | - | - | - | - | - |
| Ord.0.70Load.000Cross.4Var.500Size.2Fac.0.00Fcor | 1.00 | 1.00 | 0.82 | 0.85 | 1.00 | 1.00 | - | - | - | - | - | - | - | - | - | - | - | - |
| Ord.0.70Load.000Cross.4Var.500Size.2Fac.0.50Fcor | 1.00 | 1.00 | 0.66 | 0.79 | 0.96 | 1.00 | - | - | - | - | - | - | - | - | - | - | - | - |
| Ord.0.70Load.000Cross.4Var.500Size.2Fac.0.70Fcor | 1.00 | 1.00 | 0.67 | 0.82 | 0.59 | 0.37 | - | - | - | - | - | - | - | - | - | - | - | - |
| Ord.0.70Load.000Cross.4Var.1000Size.2Fac.0.00Fcor | 0.99 | 1.00 | 0.79 | 0.87 | 1.00 | 1.00 | - | - | - | - | - | - | - | - | - | - | - | - |
| Ord.0.70Load.000Cross.4Var.1000Size.2Fac.0.50Fcor | 1.00 | 1.00 | 0.67 | 0.83 | 0.96 | 1.00 | - | - | - | - | - | - | - | - | - | - | - | - |
| Ord.0.70Load.000Cross.4Var.1000Size.2Fac.0.70Fcor | 0.99 | 0.99 | 0.64 | 0.84 | 0.63 | 0.44 | - | - | - | - | - | - | - | - | - | - | - | - |
| Ord.0.70Load.000Cross.4Var.300Size.4Fac.0.00Fcor | 0.99 | 1.00 | 0.75 | 0.93 | 1.00 | 1.00 | - | - | - | - | - | - | - | - | - | - | - | - |
| Ord.0.70Load.000Cross.4Var.300Size.4Fac.0.50Fcor | 0.99 | 0.99 | 0.29 | 0.86 | 0.97 | 0.94 | - | - | - | - | - | - | - | - | - | - | - | - |
| Ord.0.70Load.000Cross.4Var.300Size.4Fac.0.70Fcor | 0.85 | 0.85 | 0.22 | 0.70 | 0.85 | 0.04 | - | - | - | - | - | - | - | - | - | - | - | - |
| Ord.0.70Load.000Cross.4Var.500Size.4Fac.0.00Fcor | 0.99 | 1.00 | 0.76 | 0.94 | 1.00 | 1.00 | - | - | - | - | - | - | - | - | - | - | - | - |
| Ord.0.70Load.000Cross.4Var.500Size.4Fac.0.50Fcor | 1.00 | 1.00 | 0.24 | 0.86 | 0.98 | 1.00 | - | - | - | - | - | - | - | - | - | - | - | - |
| Ord.0.70Load.000Cross.4Var.500Size.4Fac.0.70Fcor | 0.94 | 0.94 | 0.23 | 0.76 | 0.92 | 0.11 | - | - | - | - | - | - | - | - | - | - | - | - |
| Ord.0.70Load.000Cross.4Var.1000Size.4Fac.0.00Fcor | 0.99 | 1.00 | 0.68 | 0.93 | 1.00 | 1.00 | - | - | - | - | - | - | - | - | - | - | - | - |
| Ord.0.70Load.000Cross.4Var.1000Size.4Fac.0.50Fcor | 1.00 | 1.00 | 0.27 | 0.83 | 0.99 | 1.00 | - | - | - | - | - | - | - | - | - | - | - | - |
| Ord.0.70Load.000Cross.4Var.1000Size.4Fac.0.70Fcor | 0.97 | 0.97 | 0.26 | 0.83 | 0.93 | 0.24 | - | - | - | - | - | - | - | - | - | - | - | - |
| Ord.0.70Load.000Cross.8Var.300Size.2Fac.0.00Fcor | 0.99 | 1.00 | 0.61 | 0.56 | 1.00 | 1.00 | - | - | - | - | - | - | - | - | - | - | - | - |
| Ord.0.70Load.000Cross.8Var.300Size.2Fac.0.50Fcor | 1.00 | 1.00 | 0.64 | 0.53 | 1.00 | 1.00 | - | - | - | - | - | - | - | - | - | - | - | - |
| Ord.0.70Load.000Cross.8Var.300Size.2Fac.0.70Fcor | 0.96 | 0.96 | 0.55 | 0.52 | 0.99 | 0.99 | - | - | - | - | - | - | - | - | - | - | - | - |
| Ord.0.70Load.000Cross.8Var.500Size.2Fac.0.00Fcor | 0.99 | 1.00 | 0.63 | 0.51 | 1.00 | 1.00 | - | - | - | - | - | - | - | - | - | - | - | - |
| Ord.0.70Load.000Cross.8Var.500Size.2Fac.0.50Fcor | 1.00 | 1.00 | 0.61 | 0.59 | 1.00 | 1.00 | - | - | - | - | - | - | - | - | - | - | - | - |
| Ord.0.70Load.000Cross.8Var.500Size.2Fac.0.70Fcor | 0.99 | 0.99 | 0.55 | 0.57 | 1.00 | 1.00 | - | - | - | - | - | - | - | - | - | - | - | - |
| Ord.0.70Load.000Cross.8Var.1000Size.2Fac.0.00Fcor | 0.99 | 1.00 | 0.67 | 0.59 | 1.00 | 1.00 | - | - | - | - | - | - | - | - | - | - | - | - |
| Ord.0.70Load.000Cross.8Var.1000Size.2Fac.0.50Fcor | 1.00 | 1.00 | 0.58 | 0.62 | 1.00 | 1.00 | - | - | - | - | - | - | - | - | - | - | - | - |
| Ord.0.70Load.000Cross.8Var.1000Size.2Fac.0.70Fcor | 0.98 | 0.98 | 0.51 | 0.58 | 1.00 | 1.00 | - | - | - | - | - | - | - | - | - | - | - | - |
| Ord.0.70Load.000Cross.8Var.300Size.4Fac.0.00Fcor | 0.99 | 1.00 | 0.54 | 0.65 | 1.00 | 1.00 | - | - | - | - | - | - | - | - | - | - | - | - |
| Ord.0.70Load.000Cross.8Var.300Size.4Fac.0.50Fcor | 0.99 | 0.99 | 0.34 | 0.68 | 1.00 | 1.00 | - | - | - | - | - | - | - | - | - | - | - | - |
| Ord.0.70Load.000Cross.8Var.300Size.4Fac.0.70Fcor | 0.90 | 0.90 | 0.22 | 0.67 | 1.00 | 0.86 | - | - | - | - | - | - | - | - | - | - | - | - |
| Ord.0.70Load.000Cross.8Var.500Size.4Fac.0.00Fcor | 0.99 | 1.00 | 0.57 | 0.77 | 1.00 | 1.00 | - | - | - | - | - | - | - | - | - | - | - | - |
| Ord.0.70Load.000Cross.8Var.500Size.4Fac.0.50Fcor | 1.00 | 1.00 | 0.35 | 0.70 | 1.00 | 1.00 | - | - | - | - | - | - | - | - | - | - | - | - |
| Ord.0.70Load.000Cross.8Var.500Size.4Fac.0.70Fcor | 0.96 | 0.96 | 0.29 | 0.71 | 1.00 | 0.99 | - | - | - | - | - | - | - | - | - | - | - | - |
| Ord.0.70Load.000Cross.8Var.1000Size.4Fac.0.00Fcor | 0.99 | 1.00 | 0.56 | 0.76 | 1.00 | 1.00 | - | - | - | - | - | - | - | - | - | - | - | - |
| Ord.0.70Load.000Cross.8Var.1000Size.4Fac.0.50Fcor | 1.00 | 1.00 | 0.31 | 0.76 | 1.00 | 1.00 | - | - | - | - | - | - | - | - | - | - | - | - |
| Ord.0.70Load.000Cross.8Var.1000Size.4Fac.0.70Fcor | 0.99 | 0.99 | 0.25 | 0.75 | 1.00 | 1.00 | - | - | - | - | - | - | - | - | - | - | - | - |
| Ord.0.70Load.125Cross.4Var.300Size.2Fac.0.00Fcor | 0.85 | 0.85 | 0.73 | 0.54 | 0.91 | 0.90 | 48.00 | 47.00 | 60.00 | 14.00 | 100.00 | 61.00 | 100.00 | 100.00 | 94.14 | 95.43 | 95.57 | 100.00 |
| Ord.0.70Load.125Cross.4Var.300Size.2Fac.0.50Fcor | 0.97 | 0.97 | 0.82 | 0.59 | 0.86 | 0.85 | 91.00 | 91.00 | 94.00 | 63.00 | 100.00 | 68.00 | 99.86 | 99.86 | 94.29 | 94.29 | 93.43 | 100.00 |
| Ord.0.70Load.125Cross.4Var.300Size.2Fac.0.70Fcor | 0.94 | 0.94 | 0.75 | 0.58 | 0.29 | 0.06 | 98.00 | 98.00 | 98.00 | 84.00 | 38.00 | 4.00 | 98.43 | 98.43 | 93.86 | 97.57 | 96.00 | 100.00 |
| Ord.0.70Load.125Cross.4Var.500Size.2Fac.0.00Fcor | 0.85 | 0.85 | 0.73 | 0.50 | 0.95 | 0.90 | 45.00 | 47.00 | 61.00 | 1.00 | 100.00 | 60.00 | 100.00 | 100.00 | 95.14 | 96.57 | 97.86 | 100.00 |
| Ord.0.70Load.125Cross.4Var.500Size.2Fac.0.50Fcor | 0.99 | 0.99 | 0.83 | 0.64 | 0.90 | 0.93 | 95.00 | 95.00 | 96.00 | 72.00 | 100.00 | 76.00 | 100.00 | 100.00 | 95.86 | 93.00 | 95.29 | 100.00 |
| Ord.0.70Load.125Cross.4Var.500Size.2Fac.0.70Fcor | 0.95 | 0.95 | 0.80 | 0.57 | 0.19 | 0.07 | 100.00 | 100.00 | 100.00 | 79.00 | 23.00 | 6.00 | 97.86 | 97.86 | 94.43 | 97.14 | 98.43 | 100.00 |
| Ord.0.70Load.125Cross.4Var.1000Size.2Fac.0.00Fcor | 0.82 | 0.82 | 0.76 | 0.55 | 0.98 | 0.97 | 34.00 | 35.00 | 67.00 | 7.00 | 100.00 | 88.00 | 100.00 | 100.00 | 96.29 | 97.86 | 99.00 | 100.00 |
| Ord.0.70Load.125Cross.4Var.1000Size.2Fac.0.50Fcor | 1.00 | 1.00 | 0.85 | 0.58 | 0.96 | 0.97 | 100.00 | 100.00 | 97.00 | 64.00 | 100.00 | 88.00 | 100.00 | 100.00 | 95.71 | 97.29 | 98.14 | 100.00 |
| Ord.0.70Load.125Cross.4Var.1000Size.2Fac.0.70Fcor | 0.99 | 0.99 | 0.83 | 0.60 | 0.08 | 0.05 | 100.00 | 100.00 | 100.00 | 79.00 | 8.00 | 4.00 | 99.57 | 99.57 | 96.29 | 99.57 | 99.86 | 100.00 |
| Ord.0.70Load.125Cross.4Var.300Size.4Fac.0.00Fcor | 0.93 | 0.93 | 0.60 | 0.71 | 0.98 | 0.94 | 59.50 | 60.00 | 31.50 | 16.00 | 93.00 | 65.00 | 100.00 | 100.00 | 95.79 | 96.57 | 99.36 | 100.00 |
| Ord.0.70Load.125Cross.4Var.300Size.4Fac.0.50Fcor | 0.96 | 0.96 | 0.38 | 0.80 | 0.96 | 0.76 | 90.50 | 90.50 | 56.50 | 78.50 | 98.50 | 36.00 | 98.86 | 98.86 | 86.36 | 92.64 | 98.57 | 100.00 |
| Ord.0.70Load.125Cross.4Var.300Size.4Fac.0.70Fcor | 0.79 | 0.79 | 0.30 | 0.71 | 0.76 | 0.05 | 86.50 | 86.50 | 62.00 | 82.00 | 87.50 | 2.00 | 93.07 | 93.07 | 83.36 | 89.71 | 95.00 | 100.00 |
| Ord.0.70Load.125Cross.4Var.500Size.4Fac.0.00Fcor | 0.95 | 0.95 | 0.54 | 0.67 | 0.99 | 0.96 | 73.50 | 73.00 | 33.00 | 9.00 | 98.50 | 75.00 | 99.71 | 100.00 | 94.64 | 98.21 | 99.57 | 100.00 |
| Ord.0.70Load.125Cross.4Var.500Size.4Fac.0.50Fcor | 0.98 | 0.98 | 0.38 | 0.81 | 0.96 | 0.89 | 91.50 | 91.50 | 64.50 | 81.50 | 96.50 | 55.00 | 99.71 | 99.71 | 87.36 | 92.00 | 99.57 | 100.00 |
| Ord.0.70Load.125Cross.4Var.500Size.4Fac.0.70Fcor | 0.86 | 0.86 | 0.35 | 0.74 | 0.85 | 0.08 | 88.00 | 88.00 | 58.50 | 90.00 | 89.50 | 3.50 | 96.57 | 96.57 | 85.21 | 91.57 | 96.86 | 100.00 |
| Ord.0.70Load.125Cross.4Var.1000Size.4Fac.0.00Fcor | 0.97 | 0.97 | 0.41 | 0.68 | 0.99 | 0.98 | 83.50 | 85.00 | 23.00 | 5.00 | 99.00 | 87.00 | 100.00 | 100.00 | 91.93 | 99.43 | 99.50 | 100.00 |
| Ord.0.70Load.125Cross.4Var.1000Size.4Fac.0.50Fcor | 0.98 | 0.98 | 0.32 | 0.81 | 0.97 | 0.95 | 95.50 | 95.50 | 64.00 | 86.50 | 97.50 | 74.50 | 99.64 | 99.64 | 83.93 | 91.21 | 99.29 | 100.00 |
| Ord.0.70Load.125Cross.4Var.1000Size.4Fac.0.70Fcor | 0.91 | 0.91 | 0.37 | 0.81 | 0.85 | 0.21 | 93.00 | 93.00 | 57.50 | 93.50 | 90.00 | 6.50 | 97.93 | 97.93 | 90.00 | 95.36 | 97.57 | 99.93 |
| Ord.0.70Load.125Cross.8Var.300Size.2Fac.0.00Fcor | 0.84 | 0.83 | 0.49 | 0.38 | 0.81 | 0.91 | 48.50 | 45.50 | 17.50 | 7.50 | 59.00 | 63.50 | 99.79 | 99.79 | 86.29 | 79.29 | 96.07 | 100.00 |
| Ord.0.70Load.125Cross.8Var.300Size.2Fac.0.50Fcor | 0.97 | 0.97 | 0.45 | 0.38 | 0.97 | 0.94 | 91.00 | 91.00 | 39.50 | 41.00 | 98.50 | 77.00 | 99.57 | 99.57 | 79.79 | 74.29 | 98.07 | 100.00 |
| Ord.0.70Load.125Cross.8Var.300Size.2Fac.0.70Fcor | 0.95 | 0.95 | 0.22 | 0.44 | 0.99 | 0.82 | 96.00 | 96.00 | 46.00 | 80.50 | 100.00 | 61.50 | 98.57 | 98.57 | 81.14 | 81.57 | 99.43 | 100.00 |
| Ord.0.70Load.125Cross.8Var.500Size.2Fac.0.00Fcor | 0.83 | 0.80 | 0.50 | 0.41 | 0.84 | 0.94 | 49.50 | 44.00 | 22.00 | 8.50 | 68.50 | 79.00 | 99.64 | 99.64 | 88.79 | 84.29 | 97.21 | 100.00 |
| Ord.0.70Load.125Cross.8Var.500Size.2Fac.0.50Fcor | 0.98 | 0.98 | 0.39 | 0.42 | 0.99 | 0.97 | 94.00 | 94.00 | 41.00 | 50.00 | 100.00 | 88.00 | 100.00 | 100.00 | 79.50 | 72.79 | 99.36 | 100.00 |
| Ord.0.70Load.125Cross.8Var.500Size.2Fac.0.70Fcor | 0.98 | 0.98 | 0.33 | 0.45 | 1.00 | 0.95 | 98.00 | 98.00 | 60.00 | 78.50 | 100.00 | 81.00 | 99.36 | 99.36 | 83.00 | 86.79 | 99.93 | 100.00 |
| Ord.0.70Load.125Cross.8Var.1000Size.2Fac.0.00Fcor | 0.85 | 0.79 | 0.55 | 0.39 | 0.88 | 0.97 | 55.50 | 41.50 | 35.00 | 4.50 | 74.50 | 89.50 | 99.71 | 99.57 | 89.00 | 89.14 | 98.50 | 100.00 |
| Ord.0.70Load.125Cross.8Var.1000Size.2Fac.0.50Fcor | 0.98 | 0.98 | 0.41 | 0.41 | 0.99 | 0.99 | 94.50 | 94.50 | 35.50 | 40.00 | 100.00 | 97.00 | 100.00 | 100.00 | 81.00 | 77.14 | 99.00 | 100.00 |
| Ord.0.70Load.125Cross.8Var.1000Size.2Fac.0.70Fcor | 1.00 | 1.00 | 0.42 | 0.56 | 1.00 | 0.99 | 99.50 | 99.50 | 61.50 | 90.50 | 100.00 | 95.00 | 100.00 | 100.00 | 86.21 | 94.79 | 100.00 | 100.00 |
| Ord.0.70Load.125Cross.8Var.300Size.4Fac.0.00Fcor | 0.94 | 0.94 | 0.50 | 0.56 | 0.91 | 0.96 | 60.50 | 60.50 | 37.75 | 4.25 | 42.00 | 75.50 | 99.86 | 99.89 | 94.18 | 82.50 | 100.00 | 100.00 |
| Ord.0.70Load.125Cross.8Var.300Size.4Fac.0.50Fcor | 0.97 | 0.97 | 0.48 | 0.62 | 0.99 | 0.96 | 95.50 | 95.50 | 63.00 | 59.00 | 97.50 | 77.25 | 98.64 | 98.64 | 92.11 | 81.86 | 99.75 | 100.00 |
| Ord.0.70Load.125Cross.8Var.300Size.4Fac.0.70Fcor | 0.87 | 0.87 | 0.39 | 0.62 | 0.99 | 0.74 | 95.50 | 95.50 | 58.75 | 77.00 | 96.00 | 40.00 | 92.46 | 92.46 | 88.04 | 84.86 | 99.93 | 99.96 |
| Ord.0.70Load.125Cross.8Var.500Size.4Fac.0.00Fcor | 0.94 | 0.94 | 0.53 | 0.60 | 0.96 | 0.98 | 65.25 | 65.50 | 48.75 | 6.50 | 73.25 | 87.25 | 99.75 | 99.96 | 93.75 | 89.11 | 100.00 | 100.00 |
| Ord.0.70Load.125Cross.8Var.500Size.4Fac.0.50Fcor | 0.99 | 0.99 | 0.55 | 0.66 | 0.99 | 0.98 | 96.75 | 96.75 | 68.00 | 65.00 | 99.75 | 88.50 | 99.89 | 99.89 | 93.36 | 83.36 | 99.93 | 100.00 |
| Ord.0.70Load.125Cross.8Var.500Size.4Fac.0.70Fcor | 0.96 | 0.96 | 0.42 | 0.69 | 0.99 | 0.90 | 99.50 | 99.50 | 64.50 | 84.00 | 99.00 | 59.75 | 97.43 | 97.43 | 90.89 | 88.43 | 99.93 | 100.00 |
| Ord.0.70Load.125Cross.8Var.1000Size.4Fac.0.00Fcor | 0.96 | 0.96 | 0.50 | 0.57 | 0.99 | 0.99 | 76.50 | 76.50 | 55.50 | 13.00 | 95.50 | 94.25 | 99.82 | 99.96 | 90.86 | 93.32 | 99.96 | 100.00 |
| Ord.0.70Load.125Cross.8Var.1000Size.4Fac.0.50Fcor | 0.99 | 0.99 | 0.56 | 0.79 | 0.99 | 0.99 | 95.00 | 95.00 | 65.25 | 74.00 | 100.00 | 96.75 | 100.00 | 100.00 | 95.25 | 91.04 | 99.89 | 100.00 |
| Ord.0.70Load.125Cross.8Var.1000Size.4Fac.0.70Fcor | 0.99 | 0.99 | 0.39 | 0.78 | 0.99 | 0.97 | 99.50 | 99.50 | 62.50 | 91.25 | 99.75 | 82.25 | 99.64 | 99.64 | 91.71 | 92.89 | 99.89 | 100.00 |
| Ord.0.70Load.Cross250.4Var.300Size.2Fac.0.00Fcor | 0.66 | 0.63 | 0.45 | 0.33 | 0.55 | 0.68 | 49.00 | 43.50 | 34.00 | 17.00 | 87.50 | 36.50 | 98.17 | 98.17 | 90.67 | 91.17 | 77.33 | 100.00 |
| Ord.0.70Load.Cross250.4Var.300Size.2Fac.0.50Fcor | 0.94 | 0.94 | 0.39 | 0.42 | 0.65 | 0.45 | 93.50 | 93.50 | 46.50 | 48.00 | 78.00 | 28.50 | 97.83 | 97.83 | 81.50 | 90.33 | 92.83 | 100.00 |
| Ord.0.70Load.Cross250.4Var.300Size.2Fac.0.70Fcor | 0.87 | 0.87 | 0.65 | 0.51 | 0.03 | 0.01 | 90.00 | 90.00 | 85.50 | 57.00 | 4.00 | 0.00 | 98.00 | 98.00 | 91.33 | 99.00 | 99.33 | 100.00 |
| Ord.0.70Load.Cross250.4Var.500Size.2Fac.0.00Fcor | 0.77 | 0.76 | 0.43 | 0.28 | 0.56 | 0.72 | 65.50 | 63.50 | 40.50 | 9.50 | 93.50 | 45.50 | 99.33 | 99.00 | 86.83 | 93.83 | 75.67 | 100.00 |
| Ord.0.70Load.Cross250.4Var.500Size.2Fac.0.50Fcor | 0.99 | 0.99 | 0.43 | 0.47 | 0.63 | 0.65 | 98.50 | 98.50 | 46.50 | 50.50 | 80.00 | 47.00 | 100.00 | 100.00 | 78.17 | 89.00 | 92.00 | 100.00 |
| Ord.0.70Load.Cross250.4Var.500Size.2Fac.0.70Fcor | 0.98 | 0.98 | 0.80 | 0.62 | 0.00 | 0.00 | 98.00 | 98.00 | 92.50 | 66.50 | 0.00 | 0.00 | 99.83 | 99.83 | 94.50 | 98.50 | 100.00 | 100.00 |
| Ord.0.70Load.Cross250.4Var.1000Size.2Fac.0.00Fcor | 0.90 | 0.89 | 0.40 | 0.25 | 0.60 | 0.78 | 84.00 | 83.50 | 37.00 | 4.50 | 99.50 | 58.00 | 99.83 | 99.83 | 88.67 | 96.17 | 77.67 | 100.00 |
| Ord.0.70Load.Cross250.4Var.1000Size.2Fac.0.50Fcor | 0.99 | 0.99 | 0.47 | 0.52 | 0.67 | 0.77 | 99.00 | 99.00 | 46.50 | 49.50 | 84.00 | 60.00 | 100.00 | 100.00 | 77.50 | 86.17 | 91.67 | 100.00 |
| Ord.0.70Load.Cross250.4Var.1000Size.2Fac.0.70Fcor | 1.00 | 1.00 | 0.85 | 0.66 | 0.00 | 0.00 | 100.00 | 100.00 | 96.00 | 69.00 | 0.00 | 0.00 | 100.00 | 100.00 | 95.67 | 99.67 | 100.00 | 100.00 |
| Ord.0.70Load.Cross250.4Var.300Size.4Fac.0.00Fcor | 0.92 | 0.92 | 0.54 | 0.55 | 0.97 | 0.89 | 76.25 | 75.50 | 65.25 | 20.25 | 92.50 | 62.25 | 99.58 | 99.75 | 96.00 | 97.58 | 99.42 | 100.00 |
| Ord.0.70Load.Cross250.4Var.300Size.4Fac.0.50Fcor | 0.95 | 0.95 | 0.49 | 0.82 | 0.91 | 0.67 | 93.50 | 93.50 | 63.75 | 83.00 | 95.75 | 33.00 | 99.08 | 99.08 | 96.00 | 93.83 | 98.17 | 99.83 |
| Ord.0.70Load.Cross250.4Var.300Size.4Fac.0.70Fcor | 0.82 | 0.82 | 0.35 | 0.71 | 0.54 | 0.02 | 86.25 | 86.25 | 61.00 | 85.00 | 65.75 | 1.00 | 96.00 | 96.17 | 90.42 | 90.75 | 97.67 | 100.00 |
| Ord.0.70Load.Cross250.4Var.500Size.4Fac.0.00Fcor | 0.92 | 0.93 | 0.49 | 0.49 | 0.98 | 0.91 | 75.50 | 76.00 | 61.50 | 13.75 | 99.00 | 70.25 | 99.92 | 100.00 | 93.25 | 99.08 | 98.92 | 100.00 |
| Ord.0.70Load.Cross250.4Var.500Size.4Fac.0.50Fcor | 0.98 | 0.98 | 0.55 | 0.84 | 0.96 | 0.80 | 95.25 | 95.25 | 68.25 | 85.50 | 98.75 | 55.50 | 99.75 | 99.75 | 95.58 | 93.33 | 99.42 | 99.92 |
| Ord.0.70Load.Cross250.4Var.500Size.4Fac.0.70Fcor | 0.91 | 0.91 | 0.40 | 0.80 | 0.60 | 0.03 | 93.75 | 93.75 | 68.00 | 90.50 | 70.00 | 1.50 | 98.33 | 98.33 | 91.58 | 95.50 | 98.50 | 100.00 |
| Ord.0.70Load.Cross250.4Var.1000Size.4Fac.0.00Fcor | 0.96 | 0.96 | 0.45 | 0.43 | 0.99 | 0.95 | 88.25 | 88.00 | 64.75 | 4.25 | 100.00 | 83.00 | 100.00 | 100.00 | 89.75 | 100.00 | 99.17 | 100.00 |
| Ord.0.70Load.Cross250.4Var.1000Size.4Fac.0.50Fcor | 0.99 | 0.99 | 0.55 | 0.84 | 0.97 | 0.89 | 98.00 | 98.00 | 69.00 | 85.50 | 100.00 | 72.50 | 100.00 | 100.00 | 96.58 | 93.50 | 99.83 | 100.00 |
| Ord.0.70Load.Cross250.4Var.1000Size.4Fac.0.70Fcor | 0.95 | 0.95 | 0.43 | 0.82 | 0.53 | 0.11 | 99.75 | 99.75 | 68.00 | 94.50 | 60.00 | 3.00 | 98.92 | 98.92 | 91.00 | 95.00 | 98.25 | 100.00 |
| Ord.0.70Load.Cross250.8Var.300Size.2Fac.0.00Fcor | 0.84 | 0.83 | 0.49 | 0.38 | 0.81 | 0.91 | 48.50 | 45.50 | 17.50 | 7.50 | 59.00 | 63.50 | 99.79 | 99.79 | 86.29 | 79.29 | 96.07 | 100.00 |
| Ord.0.70Load.Cross250.8Var.300Size.2Fac.0.50Fcor | 0.97 | 0.97 | 0.45 | 0.38 | 0.97 | 0.94 | 91.00 | 91.00 | 39.50 | 41.00 | 98.50 | 77.00 | 99.57 | 99.57 | 79.79 | 74.29 | 98.07 | 100.00 |
| Ord.0.70Load.Cross250.8Var.300Size.2Fac.0.70Fcor | 0.95 | 0.95 | 0.22 | 0.44 | 0.99 | 0.82 | 96.00 | 96.00 | 46.00 | 80.50 | 100.00 | 61.50 | 98.57 | 98.57 | 81.14 | 81.57 | 99.43 | 100.00 |
| Ord.0.70Load.Cross250.8Var.500Size.2Fac.0.00Fcor | 0.83 | 0.80 | 0.50 | 0.41 | 0.84 | 0.94 | 49.50 | 44.00 | 22.00 | 8.50 | 68.50 | 79.00 | 99.64 | 99.64 | 88.79 | 84.29 | 97.21 | 100.00 |
| Ord.0.70Load.Cross250.8Var.500Size.2Fac.0.50Fcor | 0.98 | 0.98 | 0.39 | 0.42 | 0.99 | 0.97 | 94.00 | 94.00 | 41.00 | 50.00 | 100.00 | 88.00 | 100.00 | 100.00 | 79.50 | 72.79 | 99.36 | 100.00 |
| Ord.0.70Load.Cross250.8Var.500Size.2Fac.0.70Fcor | 0.98 | 0.98 | 0.33 | 0.45 | 1.00 | 0.95 | 98.00 | 98.00 | 60.00 | 78.50 | 100.00 | 81.00 | 99.36 | 99.36 | 83.00 | 86.79 | 99.93 | 100.00 |
| Ord.0.70Load.Cross250.8Var.1000Size.2Fac.0.00Fcor | 0.85 | 0.79 | 0.55 | 0.39 | 0.88 | 0.97 | 55.50 | 41.50 | 35.00 | 4.50 | 74.50 | 89.50 | 99.71 | 99.57 | 89.00 | 89.14 | 98.50 | 100.00 |
| Ord.0.70Load.Cross250.8Var.1000Size.2Fac.0.50Fcor | 0.98 | 0.98 | 0.41 | 0.41 | 0.99 | 0.99 | 94.50 | 94.50 | 35.50 | 40.00 | 100.00 | 97.00 | 100.00 | 100.00 | 81.00 | 77.14 | 99.00 | 100.00 |
| Ord.0.70Load.Cross250.8Var.1000Size.2Fac.0.70Fcor | 1.00 | 1.00 | 0.42 | 0.56 | 1.00 | 0.99 | 99.50 | 99.50 | 61.50 | 90.50 | 100.00 | 95.00 | 100.00 | 100.00 | 86.21 | 94.79 | 100.00 | 100.00 |
| Ord.0.70Load.Cross250.8Var.300Size.4Fac.0.00Fcor | 0.89 | 0.89 | 0.43 | 0.44 | 0.73 | 0.93 | 67.62 | 68.75 | 43.62 | 9.50 | 19.25 | 76.75 | 99.21 | 99.42 | 95.88 | 85.12 | 99.75 | 100.00 |
| Ord.0.70Load.Cross250.8Var.300Size.4Fac.0.50Fcor | 0.94 | 0.94 | 0.41 | 0.52 | 0.86 | 0.92 | 89.38 | 89.38 | 49.25 | 52.25 | 68.88 | 76.25 | 98.42 | 98.42 | 93.46 | 75.83 | 99.21 | 100.00 |
| Ord.0.70Load.Cross250.8Var.300Size.4Fac.0.70Fcor | 0.86 | 0.86 | 0.31 | 0.53 | 0.88 | 0.58 | 91.38 | 91.38 | 50.88 | 72.38 | 71.00 | 31.38 | 93.58 | 93.58 | 88.46 | 80.54 | 99.79 | 99.88 |
| Ord.0.70Load.Cross250.8Var.500Size.4Fac.0.00Fcor | 0.92 | 0.91 | 0.41 | 0.45 | 0.77 | 0.95 | 75.75 | 74.75 | 49.50 | 12.12 | 32.50 | 84.25 | 99.50 | 99.62 | 94.62 | 86.54 | 99.75 | 100.00 |
| Ord.0.70Load.Cross250.8Var.500Size.4Fac.0.50Fcor | 0.96 | 0.96 | 0.40 | 0.53 | 0.87 | 0.95 | 87.25 | 87.25 | 43.50 | 50.88 | 73.12 | 85.88 | 99.88 | 99.88 | 94.79 | 77.92 | 98.33 | 100.00 |
| Ord.0.70Load.Cross250.8Var.500Size.4Fac.0.70Fcor | 0.95 | 0.95 | 0.39 | 0.60 | 0.89 | 0.81 | 94.50 | 94.50 | 53.25 | 73.88 | 79.12 | 54.25 | 98.75 | 98.75 | 91.50 | 84.50 | 99.62 | 100.00 |
| Ord.0.70Load.Cross250.8Var.1000Size.4Fac.0.00Fcor | 0.93 | 0.93 | 0.38 | 0.54 | 0.85 | 0.99 | 80.62 | 80.12 | 53.38 | 22.12 | 56.50 | 96.38 | 99.50 | 99.58 | 89.75 | 91.04 | 99.54 | 100.00 |
| Ord.0.70Load.Cross250.8Var.1000Size.4Fac.0.50Fcor | 0.96 | 0.96 | 0.41 | 0.60 | 0.88 | 0.98 | 87.00 | 87.00 | 45.88 | 52.25 | 79.50 | 94.50 | 100.00 | 100.00 | 97.29 | 83.17 | 98.04 | 100.00 |
| Ord.0.70Load.Cross250.8Var.1000Size.4Fac.0.70Fcor | 0.99 | 0.99 | 0.36 | 0.60 | 0.91 | 0.91 | 98.50 | 98.50 | 48.00 | 73.00 | 87.88 | 70.75 | 99.92 | 99.92 | 94.46 | 90.71 | 98.96 | 100.00 |
| Dich.0.40Load.000Cross.4Var.300Size.2Fac.0.00Fcor | 0.25 | 0.25 | 0.15 | 0.16 | 0.44 | 0.65 | - | - | - | - | - | - | - | - | - | - | - | - |
| Dich.0.40Load.000Cross.4Var.300Size.2Fac.0.50Fcor | 0.16 | 0.16 | 0.10 | 0.11 | 0.25 | 0.20 | - | - | - | - | - | - | - | - | - | - | - | - |
| Dich.0.40Load.000Cross.4Var.300Size.2Fac.0.70Fcor | 0.09 | 0.09 | 0.07 | 0.07 | 0.13 | 0.04 | - | - | - | - | - | - | - | - | - | - | - | - |
| Dich.0.40Load.000Cross.4Var.500Size.2Fac.0.00Fcor | 0.56 | 0.56 | 0.45 | 0.45 | 0.80 | 0.93 | - | - | - | - | - | - | - | - | - | - | - | - |
| Dich.0.40Load.000Cross.4Var.500Size.2Fac.0.50Fcor | 0.27 | 0.27 | 0.19 | 0.21 | 0.46 | 0.31 | - | - | - | - | - | - | - | - | - | - | - | - |
| Dich.0.40Load.000Cross.4Var.500Size.2Fac.0.70Fcor | 0.17 | 0.17 | 0.12 | 0.13 | 0.26 | 0.09 | - | - | - | - | - | - | - | - | - | - | - | - |
| Dich.0.40Load.000Cross.4Var.1000Size.2Fac.0.00Fcor | 0.83 | 0.83 | 0.67 | 0.65 | 0.97 | 1.00 | - | - | - | - | - | - | - | - | - | - | - | - |
| Dich.0.40Load.000Cross.4Var.1000Size.2Fac.0.50Fcor | 0.45 | 0.45 | 0.37 | 0.40 | 0.62 | 0.51 | - | - | - | - | - | - | - | - | - | - | - | - |
| Dich.0.40Load.000Cross.4Var.1000Size.2Fac.0.70Fcor | 0.22 | 0.22 | 0.17 | 0.17 | 0.28 | 0.06 | - | - | - | - | - | - | - | - | - | - | - | - |
| Dich.0.40Load.000Cross.4Var.300Size.4Fac.0.00Fcor | 0.20 | 0.20 | 0.16 | 0.18 | 0.41 | 0.47 | - | - | - | - | - | - | - | - | - | - | - | - |
| Dich.0.40Load.000Cross.4Var.300Size.4Fac.0.50Fcor | 0.12 | 0.12 | 0.08 | 0.11 | 0.20 | 0.07 | - | - | - | - | - | - | - | - | - | - | - | - |
| Dich.0.40Load.000Cross.4Var.300Size.4Fac.0.70Fcor | 0.08 | 0.08 | 0.05 | 0.07 | 0.08 | 0.01 | - | - | - | - | - | - | - | - | - | - | - | - |
| Dich.0.40Load.000Cross.4Var.500Size.4Fac.0.00Fcor | 0.44 | 0.44 | 0.34 | 0.42 | 0.68 | 0.76 | - | - | - | - | - | - | - | - | - | - | - | - |
| Dich.0.40Load.000Cross.4Var.500Size.4Fac.0.50Fcor | 0.19 | 0.19 | 0.14 | 0.19 | 0.32 | 0.12 | - | - | - | - | - | - | - | - | - | - | - | - |
| Dich.0.40Load.000Cross.4Var.500Size.4Fac.0.70Fcor | 0.12 | 0.12 | 0.06 | 0.11 | 0.14 | 0.03 | - | - | - | - | - | - | - | - | - | - | - | - |
| Dich.0.40Load.000Cross.4Var.1000Size.4Fac.0.00Fcor | 0.72 | 0.72 | 0.62 | 0.70 | 0.94 | 0.99 | - | - | - | - | - | - | - | - | - | - | - | - |
| Dich.0.40Load.000Cross.4Var.1000Size.4Fac.0.50Fcor | 0.29 | 0.29 | 0.19 | 0.28 | 0.52 | 0.29 | - | - | - | - | - | - | - | - | - | - | - | - |
| Dich.0.40Load.000Cross.4Var.1000Size.4Fac.0.70Fcor | 0.14 | 0.14 | 0.08 | 0.13 | 0.19 | 0.01 | - | - | - | - | - | - | - | - | - | - | - | - |
| Dich.0.40Load.000Cross.8Var.300Size.2Fac.0.00Fcor | 0.37 | 0.36 | 0.29 | 0.28 | 0.64 | 0.93 | - | - | - | - | - | - | - | - | - | - | - | - |
| Dich.0.40Load.000Cross.8Var.300Size.2Fac.0.50Fcor | 0.19 | 0.19 | 0.13 | 0.17 | 0.37 | 0.44 | - | - | - | - | - | - | - | - | - | - | - | - |
| Dich.0.40Load.000Cross.8Var.300Size.2Fac.0.70Fcor | 0.11 | 0.11 | 0.08 | 0.11 | 0.19 | 0.08 | - | - | - | - | - | - | - | - | - | - | - | - |
| Dich.0.40Load.000Cross.8Var.500Size.2Fac.0.00Fcor | 0.56 | 0.56 | 0.40 | 0.41 | 0.85 | 0.99 | - | - | - | - | - | - | - | - | - | - | - | - |
| Dich.0.40Load.000Cross.8Var.500Size.2Fac.0.50Fcor | 0.33 | 0.33 | 0.22 | 0.27 | 0.65 | 0.75 | - | - | - | - | - | - | - | - | - | - | - | - |
| Dich.0.40Load.000Cross.8Var.500Size.2Fac.0.70Fcor | 0.17 | 0.17 | 0.11 | 0.14 | 0.31 | 0.18 | - | - | - | - | - | - | - | - | - | - | - | - |
| Dich.0.40Load.000Cross.8Var.1000Size.2Fac.0.00Fcor | 0.88 | 0.89 | 0.59 | 0.51 | 0.99 | 1.00 | - | - | - | - | - | - | - | - | - | - | - | - |
| Dich.0.40Load.000Cross.8Var.1000Size.2Fac.0.50Fcor | 0.57 | 0.57 | 0.33 | 0.42 | 0.93 | 0.97 | - | - | - | - | - | - | - | - | - | - | - | - |
| Dich.0.40Load.000Cross.8Var.1000Size.2Fac.0.70Fcor | 0.28 | 0.28 | 0.14 | 0.24 | 0.61 | 0.37 | - | - | - | - | - | - | - | - | - | - | - | - |
| Dich.0.40Load.000Cross.8Var.300Size.4Fac.0.00Fcor | 0.34 | 0.34 | 0.20 | 0.33 | 0.58 | 0.87 | - | - | - | - | - | - | - | - | - | - | - | - |
| Dich.0.40Load.000Cross.8Var.300Size.4Fac.0.50Fcor | 0.14 | 0.15 | 0.06 | 0.14 | 0.25 | 0.23 | - | - | - | - | - | - | - | - | - | - | - | - |
| Dich.0.40Load.000Cross.8Var.300Size.4Fac.0.70Fcor | 0.08 | 0.08 | 0.03 | 0.08 | 0.13 | 0.03 | - | - | - | - | - | - | - | - | - | - | - | - |
| Dich.0.40Load.000Cross.8Var.500Size.4Fac.0.00Fcor | 0.52 | 0.53 | 0.30 | 0.50 | 0.87 | 0.98 | - | - | - | - | - | - | - | - | - | - | - | - |
| Dich.0.40Load.000Cross.8Var.500Size.4Fac.0.50Fcor | 0.25 | 0.25 | 0.10 | 0.23 | 0.50 | 0.50 | - | - | - | - | - | - | - | - | - | - | - | - |
| Dich.0.40Load.000Cross.8Var.500Size.4Fac.0.70Fcor | 0.10 | 0.10 | 0.04 | 0.09 | 0.18 | 0.04 | - | - | - | - | - | - | - | - | - | - | - | - |
| Dich.0.40Load.000Cross.8Var.1000Size.4Fac.0.00Fcor | 0.80 | 0.81 | 0.46 | 0.69 | 1.00 | 1.00 | - | - | - | - | - | - | - | - | - | - | - | - |
| Dich.0.40Load.000Cross.8Var.1000Size.4Fac.0.50Fcor | 0.35 | 0.35 | 0.17 | 0.35 | 0.80 | 0.86 | - | - | - | - | - | - | - | - | - | - | - | - |
| Dich.0.40Load.000Cross.8Var.1000Size.4Fac.0.70Fcor | 0.14 | 0.14 | 0.06 | 0.12 | 0.35 | 0.13 | - | - | - | - | - | - | - | - | - | - | - | - |
| Dich.0.40Load.125Cross.4Var.300Size.2Fac.0.00Fcor | 0.19 | 0.20 | 0.12 | 0.13 | 0.38 | 0.51 | 13.00 | 15.00 | 17.00 | 18.00 | 28.00 | 0.00 | 94.00 | 93.57 | 90.86 | 92.14 | 90.00 | 100.00 |
| Dich.0.40Load.125Cross.4Var.300Size.2Fac.0.50Fcor | 0.20 | 0.20 | 0.16 | 0.15 | 0.29 | 0.15 | 31.00 | 30.00 | 38.00 | 40.00 | 50.00 | 0.00 | 89.14 | 88.43 | 84.71 | 86.43 | 82.71 | 100.00 |
| Dich.0.40Load.125Cross.4Var.300Size.2Fac.0.70Fcor | 0.15 | 0.15 | 0.10 | 0.11 | 0.17 | 0.03 | 29.00 | 30.00 | 36.00 | 34.00 | 48.00 | 0.00 | 87.71 | 87.57 | 84.43 | 86.57 | 81.00 | 100.00 |
| Dich.0.40Load.125Cross.4Var.500Size.2Fac.0.00Fcor | 0.31 | 0.31 | 0.23 | 0.19 | 0.47 | 0.63 | 21.00 | 22.00 | 23.00 | 20.00 | 35.00 | 0.00 | 92.43 | 91.57 | 90.86 | 92.29 | 88.29 | 100.00 |
| Dich.0.40Load.125Cross.4Var.500Size.2Fac.0.50Fcor | 0.28 | 0.28 | 0.18 | 0.19 | 0.38 | 0.15 | 35.00 | 35.00 | 40.00 | 41.00 | 66.00 | 0.00 | 91.57 | 91.43 | 87.43 | 90.57 | 86.71 | 100.00 |
| Dich.0.40Load.125Cross.4Var.500Size.2Fac.0.70Fcor | 0.21 | 0.21 | 0.16 | 0.17 | 0.21 | 0.05 | 40.00 | 40.00 | 43.00 | 41.00 | 55.00 | 0.00 | 88.29 | 87.86 | 84.71 | 89.14 | 86.14 | 100.00 |
| Dich.0.40Load.125Cross.4Var.1000Size.2Fac.0.00Fcor | 0.60 | 0.60 | 0.48 | 0.41 | 0.79 | 0.75 | 41.00 | 41.00 | 39.00 | 30.00 | 48.00 | 0.00 | 92.86 | 92.86 | 91.14 | 93.57 | 96.14 | 100.00 |
| Dich.0.40Load.125Cross.4Var.1000Size.2Fac.0.50Fcor | 0.37 | 0.37 | 0.26 | 0.28 | 0.54 | 0.20 | 44.00 | 44.00 | 48.00 | 42.00 | 80.00 | 0.00 | 91.00 | 90.43 | 86.86 | 90.43 | 87.71 | 100.00 |
| Dich.0.40Load.125Cross.4Var.1000Size.2Fac.0.70Fcor | 0.15 | 0.15 | 0.13 | 0.15 | 0.20 | 0.01 | 33.00 | 33.00 | 41.00 | 39.00 | 45.00 | 0.00 | 89.29 | 89.14 | 84.86 | 90.14 | 90.14 | 100.00 |
| Dich.0.40Load.125Cross.4Var.300Size.4Fac.0.00Fcor | 0.19 | 0.19 | 0.14 | 0.17 | 0.32 | 0.45 | 15.50 | 15.00 | 14.50 | 17.00 | 6.00 | 0.00 | 90.50 | 91.29 | 89.29 | 89.64 | 96.29 | 100.00 |
| Dich.0.40Load.125Cross.4Var.300Size.4Fac.0.50Fcor | 0.17 | 0.18 | 0.11 | 0.17 | 0.22 | 0.08 | 40.00 | 40.50 | 33.50 | 44.50 | 30.50 | 0.00 | 84.50 | 84.86 | 81.50 | 82.93 | 92.07 | 100.00 |
| Dich.0.40Load.125Cross.4Var.300Size.4Fac.0.70Fcor | 0.12 | 0.12 | 0.09 | 0.11 | 0.12 | 0.01 | 40.00 | 38.50 | 38.00 | 47.00 | 44.00 | 0.00 | 81.93 | 81.93 | 82.43 | 79.43 | 89.71 | 100.00 |
| Dich.0.40Load.125Cross.4Var.500Size.4Fac.0.00Fcor | 0.31 | 0.31 | 0.26 | 0.29 | 0.55 | 0.66 | 18.00 | 17.00 | 15.00 | 19.50 | 8.00 | 0.00 | 90.00 | 89.93 | 89.93 | 90.36 | 97.71 | 100.00 |
| Dich.0.40Load.125Cross.4Var.500Size.4Fac.0.50Fcor | 0.23 | 0.22 | 0.14 | 0.19 | 0.31 | 0.09 | 36.50 | 38.00 | 37.50 | 48.50 | 41.00 | 0.00 | 84.79 | 84.57 | 81.21 | 81.36 | 93.71 | 100.00 |
| Dich.0.40Load.125Cross.4Var.500Size.4Fac.0.70Fcor | 0.14 | 0.14 | 0.09 | 0.15 | 0.18 | 0.00 | 45.00 | 44.50 | 42.50 | 60.50 | 53.00 | 0.00 | 84.50 | 84.79 | 83.00 | 81.50 | 92.79 | 100.00 |
| Dich.0.40Load.125Cross.4Var.1000Size.4Fac.0.00Fcor | 0.59 | 0.59 | 0.50 | 0.58 | 0.78 | 0.81 | 29.00 | 29.50 | 26.00 | 28.50 | 9.00 | 0.00 | 91.79 | 91.50 | 92.00 | 92.50 | 99.64 | 100.00 |
| Dich.0.40Load.125Cross.4Var.1000Size.4Fac.0.50Fcor | 0.30 | 0.30 | 0.20 | 0.27 | 0.46 | 0.22 | 37.50 | 38.00 | 37.50 | 47.50 | 46.00 | 0.00 | 87.64 | 87.00 | 83.57 | 84.50 | 94.36 | 100.00 |
| Dich.0.40Load.125Cross.4Var.1000Size.4Fac.0.70Fcor | 0.17 | 0.17 | 0.10 | 0.15 | 0.23 | 0.01 | 44.00 | 43.50 | 40.50 | 55.00 | 59.00 | 0.00 | 85.93 | 86.43 | 81.07 | 80.14 | 93.00 | 100.00 |
| Dich.0.40Load.125Cross.8Var.300Size.2Fac.0.00Fcor | 0.25 | 0.25 | 0.17 | 0.20 | 0.42 | 0.70 | 24.50 | 25.00 | 25.00 | 27.00 | 13.50 | 0.00 | 81.86 | 80.50 | 81.00 | 76.86 | 92.57 | 100.00 |
| Dich.0.40Load.125Cross.8Var.300Size.2Fac.0.50Fcor | 0.16 | 0.16 | 0.12 | 0.15 | 0.28 | 0.27 | 40.50 | 41.00 | 38.00 | 46.00 | 40.00 | 0.00 | 80.50 | 80.21 | 78.00 | 75.79 | 91.07 | 100.00 |
| Dich.0.40Load.125Cross.8Var.300Size.2Fac.0.70Fcor | 0.12 | 0.12 | 0.09 | 0.12 | 0.18 | 0.04 | 45.50 | 45.50 | 42.00 | 57.00 | 50.50 | 0.00 | 82.79 | 82.71 | 79.43 | 77.07 | 90.21 | 100.00 |
| Dich.0.40Load.125Cross.8Var.500Size.2Fac.0.00Fcor | 0.37 | 0.36 | 0.24 | 0.29 | 0.61 | 0.75 | 29.00 | 29.00 | 27.50 | 29.50 | 10.00 | 0.00 | 85.50 | 85.50 | 85.71 | 82.93 | 97.07 | 100.00 |
| Dich.0.40Load.125Cross.8Var.500Size.2Fac.0.50Fcor | 0.24 | 0.24 | 0.16 | 0.21 | 0.49 | 0.42 | 47.00 | 45.50 | 46.00 | 50.00 | 53.00 | 0.00 | 82.79 | 83.29 | 80.07 | 80.00 | 94.14 | 100.00 |
| Dich.0.40Load.125Cross.8Var.500Size.2Fac.0.70Fcor | 0.17 | 0.17 | 0.10 | 0.16 | 0.35 | 0.07 | 57.00 | 57.00 | 45.00 | 63.50 | 63.50 | 0.00 | 83.50 | 83.50 | 79.07 | 79.57 | 93.43 | 100.00 |
| Dich.0.40Load.125Cross.8Var.1000Size.2Fac.0.00Fcor | 0.68 | 0.67 | 0.40 | 0.35 | 0.72 | 0.75 | 55.00 | 54.50 | 25.50 | 24.50 | 9.00 | 0.00 | 89.14 | 88.93 | 85.50 | 83.29 | 99.07 | 100.00 |
| Dich.0.40Load.125Cross.8Var.1000Size.2Fac.0.50Fcor | 0.36 | 0.36 | 0.20 | 0.25 | 0.79 | 0.69 | 53.50 | 54.00 | 44.50 | 60.50 | 73.00 | 0.00 | 86.29 | 86.29 | 80.00 | 82.86 | 97.36 | 100.00 |
| Dich.0.40Load.125Cross.8Var.1000Size.2Fac.0.70Fcor | 0.22 | 0.22 | 0.13 | 0.18 | 0.53 | 0.10 | 61.50 | 61.50 | 53.50 | 69.00 | 72.00 | 0.00 | 80.79 | 80.79 | 77.29 | 78.50 | 95.07 | 100.00 |
| Dich.0.40Load.125Cross.8Var.300Size.4Fac.0.00Fcor | 0.25 | 0.26 | 0.17 | 0.26 | 0.44 | 0.70 | 26.50 | 27.00 | 24.00 | 29.50 | 1.50 | 0.00 | 77.57 | 77.50 | 78.29 | 75.61 | 98.96 | 100.00 |
| Dich.0.40Load.125Cross.8Var.300Size.4Fac.0.50Fcor | 0.14 | 0.13 | 0.08 | 0.13 | 0.23 | 0.18 | 47.50 | 47.00 | 40.50 | 58.00 | 18.00 | 0.00 | 78.89 | 78.64 | 75.71 | 70.64 | 97.61 | 100.00 |
| Dich.0.40Load.125Cross.8Var.300Size.4Fac.0.70Fcor | 0.10 | 0.10 | 0.06 | 0.10 | 0.13 | 0.01 | 51.00 | 51.25 | 44.00 | 65.25 | 23.75 | 0.00 | 78.07 | 78.18 | 73.71 | 69.68 | 96.43 | 100.00 |
| Dich.0.40Load.125Cross.8Var.500Size.4Fac.0.00Fcor | 0.43 | 0.43 | 0.26 | 0.42 | 0.71 | 0.82 | 42.50 | 42.50 | 31.00 | 38.75 | 2.50 | 0.00 | 77.04 | 77.57 | 81.71 | 76.21 | 99.57 | 100.00 |
| Dich.0.40Load.125Cross.8Var.500Size.4Fac.0.50Fcor | 0.20 | 0.20 | 0.12 | 0.19 | 0.40 | 0.37 | 54.75 | 55.00 | 48.00 | 61.00 | 19.75 | 0.00 | 81.43 | 81.25 | 78.54 | 76.39 | 98.14 | 100.00 |
| Dich.0.40Load.125Cross.8Var.500Size.4Fac.0.70Fcor | 0.12 | 0.12 | 0.08 | 0.12 | 0.20 | 0.03 | 55.75 | 55.75 | 45.50 | 63.75 | 27.50 | 0.00 | 82.54 | 82.54 | 81.11 | 76.14 | 98.00 | 100.00 |
| Dich.0.40Load.125Cross.8Var.1000Size.4Fac.0.00Fcor | 0.69 | 0.69 | 0.40 | 0.56 | 0.82 | 0.84 | 47.25 | 47.25 | 26.25 | 28.25 | 3.25 | 0.00 | 88.86 | 88.86 | 89.93 | 85.68 | 100.00 | 100.00 |
| Dich.0.40Load.125Cross.8Var.1000Size.4Fac.0.50Fcor | 0.30 | 0.30 | 0.13 | 0.29 | 0.66 | 0.71 | 60.75 | 60.75 | 41.00 | 62.50 | 32.50 | 0.00 | 82.89 | 82.89 | 85.18 | 81.43 | 99.04 | 100.00 |
| Dich.0.40Load.125Cross.8Var.1000Size.4Fac.0.70Fcor | 0.15 | 0.15 | 0.09 | 0.13 | 0.34 | 0.06 | 57.50 | 57.50 | 55.25 | 64.00 | 34.00 | 0.00 | 83.46 | 83.46 | 79.79 | 79.46 | 98.32 | 100.00 |
| Dich.0.40Load.Cross250.4Var.300Size.2Fac.0.00Fcor | 0.14 | 0.14 | 0.09 | 0.08 | 0.24 | 0.25 | 18.00 | 18.50 | 20.50 | 17.00 | 28.00 | 0.00 | 91.50 | 92.00 | 90.67 | 91.00 | 86.00 | 100.00 |
| Dich.0.40Load.Cross250.4Var.300Size.2Fac.0.50Fcor | 0.15 | 0.15 | 0.10 | 0.09 | 0.24 | 0.06 | 26.00 | 27.00 | 29.00 | 22.00 | 52.50 | 0.00 | 90.67 | 89.33 | 88.50 | 91.50 | 86.17 | 100.00 |
| Dich.0.40Load.Cross250.4Var.300Size.2Fac.0.70Fcor | 0.13 | 0.12 | 0.07 | 0.08 | 0.18 | 0.01 | 25.00 | 26.00 | 29.50 | 27.50 | 48.00 | 0.00 | 89.83 | 89.67 | 86.17 | 88.33 | 89.67 | 100.00 |
| Dich.0.40Load.Cross250.4Var.500Size.2Fac.0.00Fcor | 0.22 | 0.21 | 0.16 | 0.15 | 0.34 | 0.41 | 20.50 | 20.00 | 22.50 | 20.50 | 28.00 | 0.00 | 90.33 | 90.17 | 88.17 | 90.83 | 89.33 | 100.00 |
| Dich.0.40Load.Cross250.4Var.500Size.2Fac.0.50Fcor | 0.24 | 0.25 | 0.16 | 0.17 | 0.36 | 0.11 | 29.50 | 32.00 | 36.50 | 35.50 | 56.50 | 0.00 | 92.83 | 92.17 | 88.00 | 91.50 | 89.50 | 100.00 |
| Dich.0.40Load.Cross250.4Var.500Size.2Fac.0.70Fcor | 0.14 | 0.14 | 0.09 | 0.10 | 0.18 | 0.00 | 27.00 | 26.00 | 32.50 | 28.00 | 38.00 | 0.00 | 89.17 | 88.83 | 85.00 | 90.00 | 91.17 | 100.00 |
| Dich.0.40Load.Cross250.4Var.1000Size.2Fac.0.00Fcor | 0.38 | 0.38 | 0.30 | 0.28 | 0.54 | 0.51 | 30.50 | 28.50 | 30.00 | 30.00 | 42.00 | 0.00 | 92.33 | 92.00 | 89.17 | 91.00 | 92.17 | 100.00 |
| Dich.0.40Load.Cross250.4Var.1000Size.2Fac.0.50Fcor | 0.22 | 0.22 | 0.16 | 0.17 | 0.39 | 0.10 | 26.50 | 26.50 | 33.00 | 28.00 | 54.00 | 0.00 | 90.50 | 90.50 | 89.00 | 93.67 | 89.67 | 100.00 |
| Dich.0.40Load.Cross250.4Var.1000Size.2Fac.0.70Fcor | 0.16 | 0.16 | 0.12 | 0.11 | 0.12 | 0.01 | 27.00 | 27.50 | 30.50 | 25.50 | 21.50 | 0.00 | 91.83 | 91.50 | 90.67 | 95.00 | 94.50 | 100.00 |
| Dich.0.40Load.Cross250.4Var.300Size.4Fac.0.00Fcor | 0.16 | 0.16 | 0.13 | 0.15 | 0.27 | 0.39 | 12.75 | 12.75 | 14.00 | 13.75 | 6.00 | 0.00 | 90.50 | 90.33 | 90.00 | 89.83 | 95.75 | 100.00 |
| Dich.0.40Load.Cross250.4Var.300Size.4Fac.0.50Fcor | 0.15 | 0.16 | 0.12 | 0.15 | 0.19 | 0.04 | 32.50 | 31.75 | 30.75 | 37.25 | 30.00 | 0.00 | 87.00 | 87.33 | 84.42 | 83.58 | 92.17 | 100.00 |
| Dich.0.40Load.Cross250.4Var.300Size.4Fac.0.70Fcor | 0.14 | 0.14 | 0.11 | 0.13 | 0.14 | 0.01 | 35.50 | 35.25 | 38.25 | 44.25 | 33.75 | 0.00 | 86.58 | 86.67 | 81.75 | 82.42 | 92.58 | 100.00 |
| Dich.0.40Load.Cross250.4Var.500Size.4Fac.0.00Fcor | 0.27 | 0.27 | 0.22 | 0.26 | 0.45 | 0.54 | 21.50 | 21.75 | 19.75 | 22.25 | 7.25 | 0.00 | 89.08 | 88.42 | 89.67 | 88.58 | 96.83 | 100.00 |
| Dich.0.40Load.Cross250.4Var.500Size.4Fac.0.50Fcor | 0.21 | 0.21 | 0.16 | 0.20 | 0.30 | 0.09 | 35.00 | 36.50 | 39.75 | 41.50 | 33.75 | 0.00 | 89.50 | 88.67 | 86.50 | 85.83 | 95.67 | 100.00 |
| Dich.0.40Load.Cross250.4Var.500Size.4Fac.0.70Fcor | 0.16 | 0.16 | 0.13 | 0.16 | 0.18 | 0.01 | 34.75 | 34.75 | 42.50 | 46.00 | 42.25 | 0.00 | 85.83 | 85.92 | 84.67 | 83.33 | 94.08 | 100.00 |
| Dich.0.40Load.Cross250.4Var.1000Size.4Fac.0.00Fcor | 0.46 | 0.46 | 0.40 | 0.45 | 0.59 | 0.65 | 29.50 | 29.50 | 25.50 | 28.00 | 8.00 | 0.00 | 90.17 | 90.50 | 91.33 | 89.92 | 99.17 | 100.00 |
| Dich.0.40Load.Cross250.4Var.1000Size.4Fac.0.50Fcor | 0.25 | 0.25 | 0.18 | 0.25 | 0.41 | 0.17 | 45.00 | 45.75 | 46.75 | 52.50 | 45.25 | 0.00 | 87.00 | 86.75 | 83.33 | 83.92 | 96.67 | 100.00 |
| Dich.0.40Load.Cross250.4Var.1000Size.4Fac.0.70Fcor | 0.15 | 0.15 | 0.12 | 0.14 | 0.22 | 0.01 | 35.50 | 36.25 | 37.75 | 46.00 | 47.00 | 0.00 | 89.50 | 89.08 | 86.50 | 86.83 | 95.33 | 100.00 |
| Dich.0.40Load.Cross250.8Var.300Size.2Fac.0.00Fcor | 0.25 | 0.25 | 0.17 | 0.20 | 0.42 | 0.70 | 24.50 | 25.00 | 25.00 | 27.00 | 13.50 | 0.00 | 81.86 | 80.50 | 81.00 | 76.86 | 92.57 | 100.00 |
| Dich.0.40Load.Cross250.8Var.300Size.2Fac.0.50Fcor | 0.16 | 0.16 | 0.12 | 0.15 | 0.28 | 0.27 | 40.50 | 41.00 | 38.00 | 46.00 | 40.00 | 0.00 | 80.50 | 80.21 | 78.00 | 75.79 | 91.07 | 100.00 |
| Dich.0.40Load.Cross250.8Var.300Size.2Fac.0.70Fcor | 0.12 | 0.12 | 0.09 | 0.12 | 0.18 | 0.04 | 45.50 | 45.50 | 42.00 | 57.00 | 50.50 | 0.00 | 82.79 | 82.71 | 79.43 | 77.07 | 90.21 | 100.00 |
| Dich.0.40Load.Cross250.8Var.500Size.2Fac.0.00Fcor | 0.37 | 0.36 | 0.24 | 0.29 | 0.61 | 0.75 | 29.00 | 29.00 | 27.50 | 29.50 | 10.00 | 0.00 | 85.50 | 85.50 | 85.71 | 82.93 | 97.07 | 100.00 |
| Dich.0.40Load.Cross250.8Var.500Size.2Fac.0.50Fcor | 0.24 | 0.24 | 0.16 | 0.21 | 0.49 | 0.42 | 47.00 | 45.50 | 46.00 | 50.00 | 53.00 | 0.00 | 82.79 | 83.29 | 80.07 | 80.00 | 94.14 | 100.00 |
| Dich.0.40Load.Cross250.8Var.500Size.2Fac.0.70Fcor | 0.17 | 0.17 | 0.10 | 0.16 | 0.35 | 0.07 | 57.00 | 57.00 | 45.00 | 63.50 | 63.50 | 0.00 | 83.50 | 83.50 | 79.07 | 79.57 | 93.43 | 100.00 |
| Dich.0.40Load.Cross250.8Var.1000Size.2Fac.0.00Fcor | 0.68 | 0.67 | 0.40 | 0.35 | 0.72 | 0.75 | 55.00 | 54.50 | 25.50 | 24.50 | 9.00 | 0.00 | 89.14 | 88.93 | 85.50 | 83.29 | 99.07 | 100.00 |
| Dich.0.40Load.Cross250.8Var.1000Size.2Fac.0.50Fcor | 0.36 | 0.36 | 0.20 | 0.25 | 0.79 | 0.69 | 53.50 | 54.00 | 44.50 | 60.50 | 73.00 | 0.00 | 86.29 | 86.29 | 80.00 | 82.86 | 97.36 | 100.00 |
| Dich.0.40Load.Cross250.8Var.1000Size.2Fac.0.70Fcor | 0.22 | 0.22 | 0.13 | 0.18 | 0.53 | 0.10 | 61.50 | 61.50 | 53.50 | 69.00 | 72.00 | 0.00 | 80.79 | 80.79 | 77.29 | 78.50 | 95.07 | 100.00 |
| Dich.0.40Load.Cross250.8Var.300Size.4Fac.0.00Fcor | 0.19 | 0.19 | 0.12 | 0.19 | 0.34 | 0.56 | 26.62 | 26.38 | 25.50 | 32.12 | 3.00 | 0.00 | 76.96 | 77.29 | 78.17 | 73.92 | 98.21 | 100.00 |
| Dich.0.40Load.Cross250.8Var.300Size.4Fac.0.50Fcor | 0.14 | 0.14 | 0.10 | 0.15 | 0.21 | 0.17 | 44.25 | 44.75 | 41.25 | 55.38 | 13.88 | 0.00 | 79.50 | 78.88 | 74.79 | 72.38 | 97.79 | 100.00 |
| Dich.0.40Load.Cross250.8Var.300Size.4Fac.0.70Fcor | 0.12 | 0.11 | 0.10 | 0.13 | 0.13 | 0.01 | 48.25 | 48.75 | 44.50 | 58.50 | 21.38 | 0.00 | 82.92 | 82.17 | 78.21 | 77.42 | 98.29 | 100.00 |
| Dich.0.40Load.Cross250.8Var.500Size.4Fac.0.00Fcor | 0.31 | 0.32 | 0.18 | 0.30 | 0.52 | 0.67 | 36.25 | 36.50 | 30.12 | 37.50 | 3.12 | 0.00 | 77.42 | 76.67 | 78.33 | 74.38 | 99.21 | 100.00 |
| Dich.0.40Load.Cross250.8Var.500Size.4Fac.0.50Fcor | 0.18 | 0.18 | 0.14 | 0.18 | 0.31 | 0.26 | 44.38 | 43.50 | 41.25 | 53.75 | 16.62 | 0.00 | 86.38 | 86.75 | 83.04 | 82.58 | 98.79 | 100.00 |
| Dich.0.40Load.Cross250.8Var.500Size.4Fac.0.70Fcor | 0.12 | 0.12 | 0.11 | 0.13 | 0.19 | 0.01 | 47.00 | 47.50 | 43.62 | 60.75 | 19.62 | 0.00 | 84.79 | 84.29 | 81.04 | 78.88 | 98.79 | 100.00 |
| Dich.0.40Load.Cross250.8Var.1000Size.4Fac.0.00Fcor | 0.52 | 0.53 | 0.35 | 0.45 | 0.66 | 0.69 | 37.12 | 37.25 | 26.12 | 29.25 | 2.38 | 0.00 | 84.83 | 84.79 | 88.33 | 82.42 | 99.92 | 100.00 |
| Dich.0.40Load.Cross250.8Var.1000Size.4Fac.0.50Fcor | 0.25 | 0.25 | 0.15 | 0.24 | 0.52 | 0.55 | 49.75 | 49.75 | 42.38 | 56.50 | 20.12 | 0.00 | 84.88 | 84.88 | 85.83 | 80.58 | 99.38 | 100.00 |
| Dich.0.40Load.Cross250.8Var.1000Size.4Fac.0.70Fcor | 0.16 | 0.16 | 0.13 | 0.16 | 0.31 | 0.04 | 50.12 | 50.12 | 51.88 | 63.50 | 22.12 | 0.00 | 84.75 | 84.75 | 79.71 | 79.38 | 98.96 | 100.00 |
| Dich.0.55Load.000Cross.4Var.300Size.2Fac.0.00Fcor | 0.84 | 0.84 | 0.65 | 0.68 | 0.91 | 0.99 | - | - | - | - | - | - | - | - | - | - | - | - |
| Dich.0.55Load.000Cross.4Var.300Size.2Fac.0.50Fcor | 0.56 | 0.55 | 0.43 | 0.42 | 0.49 | 0.51 | - | - | - | - | - | - | - | - | - | - | - | - |
| Dich.0.55Load.000Cross.4Var.300Size.2Fac.0.70Fcor | 0.32 | 0.32 | 0.23 | 0.27 | 0.20 | 0.09 | - | - | - | - | - | - | - | - | - | - | - | - |
| Dich.0.55Load.000Cross.4Var.500Size.2Fac.0.00Fcor | 0.97 | 0.97 | 0.75 | 0.77 | 0.97 | 1.00 | - | - | - | - | - | - | - | - | - | - | - | - |
| Dich.0.55Load.000Cross.4Var.500Size.2Fac.0.50Fcor | 0.78 | 0.78 | 0.54 | 0.62 | 0.67 | 0.79 | - | - | - | - | - | - | - | - | - | - | - | - |
| Dich.0.55Load.000Cross.4Var.500Size.2Fac.0.70Fcor | 0.49 | 0.49 | 0.37 | 0.42 | 0.24 | 0.10 | - | - | - | - | - | - | - | - | - | - | - | - |
| Dich.0.55Load.000Cross.4Var.1000Size.2Fac.0.00Fcor | 0.99 | 1.00 | 0.80 | 0.83 | 0.99 | 1.00 | - | - | - | - | - | - | - | - | - | - | - | - |
| Dich.0.55Load.000Cross.4Var.1000Size.2Fac.0.50Fcor | 0.93 | 0.93 | 0.63 | 0.81 | 0.74 | 0.92 | - | - | - | - | - | - | - | - | - | - | - | - |
| Dich.0.55Load.000Cross.4Var.1000Size.2Fac.0.70Fcor | 0.71 | 0.71 | 0.54 | 0.60 | 0.33 | 0.18 | - | - | - | - | - | - | - | - | - | - | - | - |
| Dich.0.55Load.000Cross.4Var.300Size.4Fac.0.00Fcor | 0.84 | 0.84 | 0.59 | 0.80 | 0.98 | 0.99 | - | - | - | - | - | - | - | - | - | - | - | - |
| Dich.0.55Load.000Cross.4Var.300Size.4Fac.0.50Fcor | 0.48 | 0.48 | 0.23 | 0.45 | 0.57 | 0.36 | - | - | - | - | - | - | - | - | - | - | - | - |
| Dich.0.55Load.000Cross.4Var.300Size.4Fac.0.70Fcor | 0.23 | 0.23 | 0.12 | 0.21 | 0.25 | 0.02 | - | - | - | - | - | - | - | - | - | - | - | - |
| Dich.0.55Load.000Cross.4Var.500Size.4Fac.0.00Fcor | 0.96 | 0.96 | 0.65 | 0.89 | 1.00 | 1.00 | - | - | - | - | - | - | - | - | - | - | - | - |
| Dich.0.55Load.000Cross.4Var.500Size.4Fac.0.50Fcor | 0.63 | 0.63 | 0.23 | 0.59 | 0.71 | 0.54 | - | - | - | - | - | - | - | - | - | - | - | - |
| Dich.0.55Load.000Cross.4Var.500Size.4Fac.0.70Fcor | 0.33 | 0.33 | 0.14 | 0.31 | 0.30 | 0.03 | - | - | - | - | - | - | - | - | - | - | - | - |
| Dich.0.55Load.000Cross.4Var.1000Size.4Fac.0.00Fcor | 0.99 | 1.00 | 0.69 | 0.92 | 1.00 | 1.00 | - | - | - | - | - | - | - | - | - | - | - | - |
| Dich.0.55Load.000Cross.4Var.1000Size.4Fac.0.50Fcor | 0.82 | 0.82 | 0.26 | 0.74 | 0.89 | 0.84 | - | - | - | - | - | - | - | - | - | - | - | - |
| Dich.0.55Load.000Cross.4Var.1000Size.4Fac.0.70Fcor | 0.44 | 0.44 | 0.17 | 0.40 | 0.50 | 0.05 | - | - | - | - | - | - | - | - | - | - | - | - |
| Dich.0.55Load.000Cross.8Var.300Size.2Fac.0.00Fcor | 0.87 | 0.88 | 0.52 | 0.52 | 0.99 | 1.00 | - | - | - | - | - | - | - | - | - | - | - | - |
| Dich.0.55Load.000Cross.8Var.300Size.2Fac.0.50Fcor | 0.61 | 0.61 | 0.39 | 0.44 | 0.89 | 0.95 | - | - | - | - | - | - | - | - | - | - | - | - |
| Dich.0.55Load.000Cross.8Var.300Size.2Fac.0.70Fcor | 0.34 | 0.34 | 0.18 | 0.28 | 0.62 | 0.41 | - | - | - | - | - | - | - | - | - | - | - | - |
| Dich.0.55Load.000Cross.8Var.500Size.2Fac.0.00Fcor | 0.96 | 0.96 | 0.56 | 0.53 | 1.00 | 1.00 | - | - | - | - | - | - | - | - | - | - | - | - |
| Dich.0.55Load.000Cross.8Var.500Size.2Fac.0.50Fcor | 0.78 | 0.79 | 0.44 | 0.48 | 0.98 | 1.00 | - | - | - | - | - | - | - | - | - | - | - | - |
| Dich.0.55Load.000Cross.8Var.500Size.2Fac.0.70Fcor | 0.50 | 0.49 | 0.28 | 0.37 | 0.78 | 0.72 | - | - | - | - | - | - | - | - | - | - | - | - |
| Dich.0.55Load.000Cross.8Var.1000Size.2Fac.0.00Fcor | 0.98 | 1.00 | 0.65 | 0.58 | 1.00 | 1.00 | - | - | - | - | - | - | - | - | - | - | - | - |
| Dich.0.55Load.000Cross.8Var.1000Size.2Fac.0.50Fcor | 0.96 | 0.96 | 0.54 | 0.51 | 1.00 | 1.00 | - | - | - | - | - | - | - | - | - | - | - | - |
| Dich.0.55Load.000Cross.8Var.1000Size.2Fac.0.70Fcor | 0.73 | 0.73 | 0.40 | 0.51 | 0.95 | 0.95 | - | - | - | - | - | - | - | - | - | - | - | - |
| Dich.0.55Load.000Cross.8Var.300Size.4Fac.0.00Fcor | 0.90 | 0.90 | 0.48 | 0.67 | 1.00 | 1.00 | - | - | - | - | - | - | - | - | - | - | - | - |
| Dich.0.55Load.000Cross.8Var.300Size.4Fac.0.50Fcor | 0.50 | 0.50 | 0.22 | 0.50 | 0.86 | 0.88 | - | - | - | - | - | - | - | - | - | - | - | - |
| Dich.0.55Load.000Cross.8Var.300Size.4Fac.0.70Fcor | 0.28 | 0.28 | 0.08 | 0.27 | 0.50 | 0.15 | - | - | - | - | - | - | - | - | - | - | - | - |
| Dich.0.55Load.000Cross.8Var.500Size.4Fac.0.00Fcor | 0.97 | 0.98 | 0.49 | 0.70 | 1.00 | 1.00 | - | - | - | - | - | - | - | - | - | - | - | - |
| Dich.0.55Load.000Cross.8Var.500Size.4Fac.0.50Fcor | 0.72 | 0.72 | 0.21 | 0.63 | 0.98 | 0.99 | - | - | - | - | - | - | - | - | - | - | - | - |
| Dich.0.55Load.000Cross.8Var.500Size.4Fac.0.70Fcor | 0.41 | 0.41 | 0.11 | 0.37 | 0.76 | 0.38 | - | - | - | - | - | - | - | - | - | - | - | - |
| Dich.0.55Load.000Cross.8Var.1000Size.4Fac.0.00Fcor | 0.99 | 1.00 | 0.49 | 0.74 | 1.00 | 1.00 | - | - | - | - | - | - | - | - | - | - | - | - |
| Dich.0.55Load.000Cross.8Var.1000Size.4Fac.0.50Fcor | 0.88 | 0.88 | 0.24 | 0.72 | 1.00 | 1.00 | - | - | - | - | - | - | - | - | - | - | - | - |
| Dich.0.55Load.000Cross.8Var.1000Size.4Fac.0.70Fcor | 0.61 | 0.61 | 0.16 | 0.55 | 0.96 | 0.78 | - | - | - | - | - | - | - | - | - | - | - | - |
| Dich.0.55Load.125Cross.4Var.300Size.2Fac.0.00Fcor | 0.65 | 0.65 | 0.51 | 0.44 | 0.67 | 0.76 | 39.00 | 39.00 | 37.00 | 23.00 | 74.00 | 0.00 | 92.86 | 93.14 | 89.86 | 90.43 | 86.00 | 100.00 |
| Dich.0.55Load.125Cross.4Var.300Size.2Fac.0.50Fcor | 0.51 | 0.51 | 0.43 | 0.41 | 0.46 | 0.32 | 63.00 | 63.00 | 64.00 | 60.00 | 86.00 | 0.00 | 88.29 | 88.00 | 81.00 | 87.29 | 77.86 | 100.00 |
| Dich.0.55Load.125Cross.4Var.300Size.2Fac.0.70Fcor | 0.36 | 0.36 | 0.27 | 0.28 | 0.16 | 0.05 | 65.00 | 66.00 | 64.00 | 60.00 | 49.00 | 0.00 | 84.71 | 84.43 | 80.43 | 87.29 | 83.29 | 100.00 |
| Dich.0.55Load.125Cross.4Var.500Size.2Fac.0.00Fcor | 0.75 | 0.75 | 0.61 | 0.49 | 0.74 | 0.75 | 56.00 | 55.00 | 54.00 | 26.00 | 80.00 | 0.00 | 94.29 | 94.29 | 89.43 | 92.00 | 88.43 | 100.00 |
| Dich.0.55Load.125Cross.4Var.500Size.2Fac.0.50Fcor | 0.59 | 0.59 | 0.49 | 0.43 | 0.49 | 0.41 | 69.00 | 69.00 | 65.00 | 59.00 | 93.00 | 0.00 | 89.29 | 89.29 | 84.00 | 83.71 | 79.57 | 100.00 |
| Dich.0.55Load.125Cross.4Var.500Size.2Fac.0.70Fcor | 0.38 | 0.38 | 0.31 | 0.31 | 0.16 | 0.05 | 62.00 | 63.00 | 63.00 | 53.00 | 41.00 | 0.00 | 89.29 | 88.86 | 85.43 | 92.57 | 87.00 | 100.00 |
| Dich.0.55Load.125Cross.4Var.1000Size.2Fac.0.00Fcor | 0.89 | 0.90 | 0.78 | 0.59 | 0.91 | 0.75 | 72.00 | 72.00 | 71.00 | 27.00 | 98.00 | 0.00 | 98.29 | 98.14 | 93.43 | 93.57 | 95.00 | 100.00 |
| Dich.0.55Load.125Cross.4Var.1000Size.2Fac.0.50Fcor | 0.79 | 0.79 | 0.71 | 0.55 | 0.68 | 0.53 | 92.00 | 92.00 | 92.00 | 70.00 | 95.00 | 0.00 | 92.57 | 92.57 | 90.86 | 92.29 | 88.14 | 100.00 |
| Dich.0.55Load.125Cross.4Var.1000Size.2Fac.0.70Fcor | 0.54 | 0.54 | 0.40 | 0.37 | 0.15 | 0.02 | 65.00 | 65.00 | 65.00 | 59.00 | 26.00 | 0.00 | 93.43 | 93.43 | 91.00 | 94.14 | 95.00 | 100.00 |
| Dich.0.55Load.125Cross.4Var.300Size.4Fac.0.00Fcor | 0.68 | 0.68 | 0.53 | 0.66 | 0.80 | 0.81 | 39.50 | 39.00 | 19.50 | 33.50 | 21.50 | 0.00 | 91.93 | 92.64 | 93.50 | 91.43 | 98.14 | 100.00 |
| Dich.0.55Load.125Cross.4Var.300Size.4Fac.0.50Fcor | 0.40 | 0.40 | 0.24 | 0.39 | 0.51 | 0.25 | 51.50 | 50.00 | 43.00 | 61.00 | 64.00 | 0.00 | 82.21 | 82.43 | 79.64 | 78.86 | 88.71 | 100.00 |
| Dich.0.55Load.125Cross.4Var.300Size.4Fac.0.70Fcor | 0.26 | 0.26 | 0.13 | 0.24 | 0.28 | 0.01 | 61.50 | 62.00 | 51.50 | 74.00 | 69.50 | 0.00 | 79.14 | 79.21 | 77.29 | 73.07 | 86.93 | 100.00 |
| Dich.0.55Load.125Cross.4Var.500Size.4Fac.0.00Fcor | 0.84 | 0.84 | 0.55 | 0.73 | 0.88 | 0.83 | 58.50 | 59.00 | 38.00 | 39.00 | 50.50 | 0.00 | 95.57 | 95.86 | 90.86 | 92.36 | 98.43 | 100.00 |
| Dich.0.55Load.125Cross.4Var.500Size.4Fac.0.50Fcor | 0.60 | 0.59 | 0.29 | 0.55 | 0.69 | 0.40 | 61.50 | 61.50 | 49.00 | 71.00 | 81.00 | 0.00 | 87.50 | 87.14 | 81.00 | 82.64 | 92.86 | 100.00 |
| Dich.0.55Load.125Cross.4Var.500Size.4Fac.0.70Fcor | 0.32 | 0.32 | 0.17 | 0.33 | 0.40 | 0.02 | 56.00 | 58.00 | 42.00 | 69.00 | 82.00 | 0.00 | 86.36 | 85.71 | 80.43 | 82.50 | 87.93 | 100.00 |
| Dich.0.55Load.125Cross.4Var.1000Size.4Fac.0.00Fcor | 0.93 | 0.93 | 0.49 | 0.78 | 0.95 | 0.83 | 70.50 | 70.00 | 37.50 | 39.50 | 76.50 | 0.00 | 99.29 | 99.57 | 90.79 | 96.00 | 99.43 | 100.00 |
| Dich.0.55Load.125Cross.4Var.1000Size.4Fac.0.50Fcor | 0.76 | 0.76 | 0.33 | 0.73 | 0.83 | 0.63 | 75.50 | 75.50 | 50.50 | 80.00 | 88.50 | 0.00 | 93.64 | 93.64 | 84.07 | 90.29 | 95.36 | 100.00 |
| Dich.0.55Load.125Cross.4Var.1000Size.4Fac.0.70Fcor | 0.39 | 0.39 | 0.21 | 0.35 | 0.50 | 0.03 | 58.00 | 58.00 | 47.50 | 68.50 | 72.50 | 0.00 | 87.14 | 87.14 | 80.64 | 81.86 | 91.29 | 100.00 |
| Dich.0.55Load.125Cross.8Var.300Size.2Fac.0.00Fcor | 0.64 | 0.63 | 0.38 | 0.34 | 0.75 | 0.75 | 49.50 | 47.50 | 27.00 | 24.00 | 34.00 | 0.00 | 89.29 | 89.21 | 80.50 | 74.36 | 95.50 | 100.00 |
| Dich.0.55Load.125Cross.8Var.300Size.2Fac.0.50Fcor | 0.42 | 0.41 | 0.20 | 0.27 | 0.79 | 0.67 | 61.50 | 62.00 | 44.00 | 63.00 | 78.50 | 0.00 | 81.07 | 81.07 | 76.07 | 75.00 | 94.21 | 100.00 |
| Dich.0.55Load.125Cross.8Var.300Size.2Fac.0.70Fcor | 0.24 | 0.25 | 0.15 | 0.20 | 0.52 | 0.17 | 58.00 | 57.50 | 49.50 | 71.00 | 77.50 | 0.00 | 78.79 | 80.00 | 74.50 | 73.07 | 90.14 | 100.00 |
| Dich.0.55Load.125Cross.8Var.500Size.2Fac.0.00Fcor | 0.73 | 0.73 | 0.43 | 0.37 | 0.81 | 0.75 | 54.00 | 52.50 | 27.00 | 26.00 | 43.50 | 0.00 | 91.86 | 91.64 | 82.71 | 75.57 | 97.14 | 100.00 |
| Dich.0.55Load.125Cross.8Var.500Size.2Fac.0.50Fcor | 0.57 | 0.57 | 0.28 | 0.35 | 0.92 | 0.75 | 70.00 | 70.00 | 54.50 | 70.00 | 88.00 | 0.00 | 84.21 | 84.57 | 76.29 | 72.57 | 97.71 | 100.00 |
| Dich.0.55Load.125Cross.8Var.500Size.2Fac.0.70Fcor | 0.35 | 0.35 | 0.15 | 0.24 | 0.74 | 0.34 | 65.50 | 65.50 | 56.00 | 72.00 | 91.00 | 0.00 | 82.21 | 82.29 | 74.86 | 77.64 | 92.50 | 100.00 |
| Dich.0.55Load.125Cross.8Var.1000Size.2Fac.0.00Fcor | 0.87 | 0.85 | 0.49 | 0.40 | 0.85 | 0.75 | 58.50 | 55.00 | 24.00 | 16.00 | 55.50 | 0.00 | 98.71 | 99.00 | 88.14 | 80.14 | 98.43 | 100.00 |
| Dich.0.55Load.125Cross.8Var.1000Size.2Fac.0.50Fcor | 0.84 | 0.84 | 0.41 | 0.37 | 0.98 | 0.75 | 84.50 | 85.50 | 54.00 | 47.00 | 95.50 | 0.00 | 93.93 | 93.50 | 80.29 | 80.50 | 99.71 | 100.00 |
| Dich.0.55Load.125Cross.8Var.1000Size.2Fac.0.70Fcor | 0.60 | 0.60 | 0.23 | 0.38 | 0.93 | 0.62 | 84.00 | 84.50 | 57.00 | 78.00 | 98.50 | 0.00 | 86.14 | 85.93 | 81.36 | 80.93 | 97.00 | 100.00 |
| Dich.0.55Load.125Cross.8Var.300Size.4Fac.0.00Fcor | 0.76 | 0.76 | 0.43 | 0.55 | 0.84 | 0.84 | 52.00 | 50.75 | 27.50 | 24.00 | 8.00 | 0.25 | 88.89 | 89.36 | 90.18 | 82.64 | 99.96 | 100.00 |
| Dich.0.55Load.125Cross.8Var.300Size.4Fac.0.50Fcor | 0.42 | 0.42 | 0.18 | 0.40 | 0.73 | 0.70 | 66.00 | 66.25 | 49.50 | 67.75 | 46.25 | 0.50 | 76.25 | 76.54 | 79.39 | 74.79 | 98.04 | 100.00 |
| Dich.0.55Load.125Cross.8Var.300Size.4Fac.0.70Fcor | 0.23 | 0.23 | 0.12 | 0.22 | 0.44 | 0.07 | 61.25 | 61.50 | 52.00 | 70.50 | 37.75 | 0.00 | 77.54 | 77.50 | 72.43 | 70.71 | 97.71 | 100.00 |
| Dich.0.55Load.125Cross.8Var.500Size.4Fac.0.00Fcor | 0.91 | 0.91 | 0.44 | 0.56 | 0.87 | 0.84 | 68.50 | 68.00 | 35.00 | 21.00 | 19.25 | 0.00 | 97.04 | 97.04 | 93.36 | 82.32 | 100.00 | 100.00 |
| Dich.0.55Load.125Cross.8Var.500Size.4Fac.0.50Fcor | 0.61 | 0.62 | 0.19 | 0.53 | 0.88 | 0.83 | 80.50 | 81.00 | 47.00 | 70.25 | 62.00 | 0.00 | 77.21 | 77.14 | 80.93 | 75.36 | 99.50 | 100.00 |
| Dich.0.55Load.125Cross.8Var.500Size.4Fac.0.70Fcor | 0.36 | 0.36 | 0.12 | 0.35 | 0.64 | 0.23 | 75.50 | 75.75 | 50.00 | 79.50 | 51.00 | 0.00 | 74.46 | 74.46 | 73.96 | 70.71 | 98.68 | 100.00 |
| Dich.0.55Load.125Cross.8Var.1000Size.4Fac.0.00Fcor | 0.96 | 0.96 | 0.50 | 0.59 | 0.90 | 0.84 | 75.50 | 75.25 | 52.00 | 13.00 | 37.25 | 0.00 | 99.68 | 99.79 | 92.82 | 82.79 | 99.96 | 100.00 |
| Dich.0.55Load.125Cross.8Var.1000Size.4Fac.0.50Fcor | 0.81 | 0.81 | 0.32 | 0.60 | 0.96 | 0.84 | 88.75 | 89.00 | 55.75 | 62.75 | 81.25 | 0.00 | 88.07 | 87.96 | 85.29 | 81.36 | 99.86 | 100.00 |
| Dich.0.55Load.125Cross.8Var.1000Size.4Fac.0.70Fcor | 0.51 | 0.51 | 0.17 | 0.46 | 0.87 | 0.52 | 77.00 | 76.00 | 53.75 | 79.50 | 71.00 | 0.00 | 79.32 | 79.50 | 79.11 | 75.61 | 99.64 | 100.00 |
| Dich.0.55Load.Cross250.4Var.300Size.2Fac.0.00Fcor | 0.40 | 0.40 | 0.34 | 0.28 | 0.45 | 0.50 | 31.50 | 31.50 | 33.50 | 31.50 | 57.00 | 0.00 | 88.83 | 88.33 | 83.83 | 83.17 | 78.50 | 100.00 |
| Dich.0.55Load.Cross250.4Var.300Size.2Fac.0.50Fcor | 0.35 | 0.34 | 0.25 | 0.21 | 0.31 | 0.10 | 40.50 | 41.50 | 40.50 | 32.50 | 60.50 | 0.00 | 88.33 | 88.00 | 84.17 | 89.33 | 83.17 | 100.00 |
| Dich.0.55Load.Cross250.4Var.300Size.2Fac.0.70Fcor | 0.19 | 0.18 | 0.13 | 0.15 | 0.11 | 0.02 | 32.00 | 32.00 | 34.50 | 29.50 | 20.00 | 0.00 | 84.83 | 84.67 | 82.83 | 87.00 | 95.00 | 100.00 |
| Dich.0.55Load.Cross250.4Var.500Size.2Fac.0.00Fcor | 0.47 | 0.46 | 0.37 | 0.29 | 0.44 | 0.53 | 35.50 | 35.50 | 40.50 | 28.50 | 71.50 | 0.00 | 91.50 | 90.50 | 83.83 | 87.33 | 75.17 | 100.00 |
| Dich.0.55Load.Cross250.4Var.500Size.2Fac.0.50Fcor | 0.48 | 0.48 | 0.35 | 0.33 | 0.41 | 0.17 | 53.50 | 53.50 | 54.50 | 42.50 | 68.50 | 0.00 | 92.17 | 92.67 | 87.17 | 92.50 | 84.33 | 100.00 |
| Dich.0.55Load.Cross250.4Var.500Size.2Fac.0.70Fcor | 0.28 | 0.28 | 0.21 | 0.19 | 0.07 | 0.01 | 45.50 | 45.50 | 46.50 | 36.50 | 12.50 | 0.00 | 87.00 | 87.17 | 84.83 | 90.50 | 95.83 | 100.00 |
| Dich.0.55Load.Cross250.4Var.1000Size.2Fac.0.00Fcor | 0.63 | 0.63 | 0.45 | 0.38 | 0.58 | 0.53 | 51.50 | 52.00 | 40.50 | 28.50 | 82.00 | 0.00 | 95.17 | 95.00 | 88.83 | 91.17 | 82.00 | 100.00 |
| Dich.0.55Load.Cross250.4Var.1000Size.2Fac.0.50Fcor | 0.69 | 0.69 | 0.48 | 0.47 | 0.46 | 0.22 | 79.50 | 79.50 | 66.50 | 56.00 | 74.00 | 0.00 | 89.83 | 89.83 | 85.67 | 92.83 | 87.17 | 100.00 |
| Dich.0.55Load.Cross250.4Var.1000Size.2Fac.0.70Fcor | 0.41 | 0.41 | 0.34 | 0.28 | 0.05 | 0.00 | 48.50 | 48.50 | 48.00 | 37.50 | 8.00 | 0.00 | 92.33 | 92.33 | 91.67 | 95.17 | 98.83 | 100.00 |
| Dich.0.55Load.Cross250.4Var.300Size.4Fac.0.00Fcor | 0.56 | 0.56 | 0.41 | 0.54 | 0.66 | 0.67 | 33.75 | 34.75 | 27.00 | 30.50 | 23.75 | 0.00 | 91.25 | 90.92 | 92.17 | 90.83 | 96.00 | 100.00 |
| Dich.0.55Load.Cross250.4Var.300Size.4Fac.0.50Fcor | 0.40 | 0.40 | 0.21 | 0.37 | 0.46 | 0.19 | 52.25 | 51.75 | 48.25 | 57.75 | 59.75 | 0.00 | 86.25 | 86.50 | 79.83 | 81.17 | 91.58 | 100.00 |
| Dich.0.55Load.Cross250.4Var.300Size.4Fac.0.70Fcor | 0.22 | 0.22 | 0.14 | 0.22 | 0.26 | 0.01 | 47.50 | 47.50 | 44.25 | 56.75 | 63.00 | 0.00 | 84.50 | 85.00 | 81.00 | 79.92 | 90.83 | 100.00 |
| Dich.0.55Load.Cross250.4Var.500Size.4Fac.0.00Fcor | 0.74 | 0.73 | 0.48 | 0.65 | 0.77 | 0.69 | 51.25 | 50.50 | 46.25 | 43.75 | 41.75 | 0.00 | 94.17 | 94.42 | 92.08 | 91.17 | 97.25 | 100.00 |
| Dich.0.55Load.Cross250.4Var.500Size.4Fac.0.50Fcor | 0.49 | 0.49 | 0.27 | 0.48 | 0.60 | 0.32 | 59.75 | 60.25 | 52.25 | 59.00 | 73.50 | 0.00 | 86.58 | 86.50 | 84.33 | 85.50 | 92.33 | 100.00 |
| Dich.0.55Load.Cross250.4Var.500Size.4Fac.0.70Fcor | 0.30 | 0.29 | 0.17 | 0.28 | 0.35 | 0.01 | 50.00 | 49.25 | 41.50 | 61.00 | 67.00 | 0.00 | 86.75 | 86.42 | 84.25 | 82.33 | 92.92 | 100.00 |
| Dich.0.55Load.Cross250.4Var.1000Size.4Fac.0.00Fcor | 0.86 | 0.86 | 0.45 | 0.73 | 0.90 | 0.69 | 68.00 | 66.75 | 59.25 | 52.75 | 75.75 | 0.00 | 97.42 | 97.58 | 90.83 | 93.33 | 98.50 | 100.00 |
| Dich.0.55Load.Cross250.4Var.1000Size.4Fac.0.50Fcor | 0.68 | 0.68 | 0.33 | 0.63 | 0.74 | 0.49 | 69.25 | 69.50 | 59.75 | 67.25 | 83.00 | 0.00 | 92.33 | 92.58 | 86.17 | 88.42 | 96.33 | 100.00 |
| Dich.0.55Load.Cross250.4Var.1000Size.4Fac.0.70Fcor | 0.38 | 0.38 | 0.20 | 0.35 | 0.39 | 0.02 | 51.00 | 51.00 | 48.50 | 60.75 | 60.75 | 0.00 | 91.42 | 91.42 | 86.75 | 87.50 | 95.58 | 100.00 |
| Dich.0.55Load.Cross250.8Var.300Size.2Fac.0.00Fcor | 0.64 | 0.63 | 0.38 | 0.34 | 0.75 | 0.75 | 49.50 | 47.50 | 27.00 | 24.00 | 34.00 | 0.00 | 89.29 | 89.21 | 80.50 | 74.36 | 95.50 | 100.00 |
| Dich.0.55Load.Cross250.8Var.300Size.2Fac.0.50Fcor | 0.42 | 0.41 | 0.20 | 0.27 | 0.79 | 0.67 | 61.50 | 62.00 | 44.00 | 63.00 | 78.50 | 0.00 | 81.07 | 81.07 | 76.07 | 75.00 | 94.21 | 100.00 |
| Dich.0.55Load.Cross250.8Var.300Size.2Fac.0.70Fcor | 0.24 | 0.25 | 0.15 | 0.20 | 0.52 | 0.17 | 58.00 | 57.50 | 49.50 | 71.00 | 77.50 | 0.00 | 78.79 | 80.00 | 74.50 | 73.07 | 90.14 | 100.00 |
| Dich.0.55Load.Cross250.8Var.500Size.2Fac.0.00Fcor | 0.73 | 0.73 | 0.43 | 0.37 | 0.81 | 0.75 | 54.00 | 52.50 | 27.00 | 26.00 | 43.50 | 0.00 | 91.86 | 91.64 | 82.71 | 75.57 | 97.14 | 100.00 |
| Dich.0.55Load.Cross250.8Var.500Size.2Fac.0.50Fcor | 0.57 | 0.57 | 0.28 | 0.35 | 0.92 | 0.75 | 70.00 | 70.00 | 54.50 | 70.00 | 88.00 | 0.00 | 84.21 | 84.57 | 76.29 | 72.57 | 97.71 | 100.00 |
| Dich.0.55Load.Cross250.8Var.500Size.2Fac.0.70Fcor | 0.35 | 0.35 | 0.15 | 0.24 | 0.74 | 0.34 | 65.50 | 65.50 | 56.00 | 72.00 | 91.00 | 0.00 | 82.21 | 82.29 | 74.86 | 77.64 | 92.50 | 100.00 |
| Dich.0.55Load.Cross250.8Var.1000Size.2Fac.0.00Fcor | 0.87 | 0.85 | 0.49 | 0.40 | 0.85 | 0.75 | 58.50 | 55.00 | 24.00 | 16.00 | 55.50 | 0.00 | 98.71 | 99.00 | 88.14 | 80.14 | 98.43 | 100.00 |
| Dich.0.55Load.Cross250.8Var.1000Size.2Fac.0.50Fcor | 0.84 | 0.84 | 0.41 | 0.37 | 0.98 | 0.75 | 84.50 | 85.50 | 54.00 | 47.00 | 95.50 | 0.00 | 93.93 | 93.50 | 80.29 | 80.50 | 99.71 | 100.00 |
| Dich.0.55Load.Cross250.8Var.1000Size.2Fac.0.70Fcor | 0.60 | 0.60 | 0.23 | 0.38 | 0.93 | 0.62 | 84.00 | 84.50 | 57.00 | 78.00 | 98.50 | 0.00 | 86.14 | 85.93 | 81.36 | 80.93 | 97.00 | 100.00 |
| Dich.0.55Load.Cross250.8Var.300Size.4Fac.0.00Fcor | 0.59 | 0.59 | 0.35 | 0.41 | 0.68 | 0.69 | 41.50 | 41.00 | 27.62 | 25.88 | 8.12 | 0.62 | 86.83 | 86.92 | 88.00 | 80.96 | 99.62 | 100.00 |
| Dich.0.55Load.Cross250.8Var.300Size.4Fac.0.50Fcor | 0.38 | 0.38 | 0.18 | 0.35 | 0.62 | 0.54 | 60.75 | 59.88 | 47.25 | 65.75 | 30.00 | 0.12 | 76.50 | 76.79 | 77.75 | 74.21 | 98.88 | 100.00 |
| Dich.0.55Load.Cross250.8Var.300Size.4Fac.0.70Fcor | 0.22 | 0.22 | 0.14 | 0.23 | 0.38 | 0.05 | 54.25 | 55.00 | 49.38 | 68.00 | 25.88 | 0.00 | 80.33 | 79.54 | 75.67 | 71.21 | 98.33 | 100.00 |
| Dich.0.55Load.Cross250.8Var.500Size.4Fac.0.00Fcor | 0.79 | 0.78 | 0.45 | 0.48 | 0.70 | 0.69 | 65.00 | 63.62 | 44.12 | 28.12 | 11.50 | 0.00 | 92.21 | 92.50 | 91.33 | 80.08 | 100.00 | 100.00 |
| Dich.0.55Load.Cross250.8Var.500Size.4Fac.0.50Fcor | 0.49 | 0.49 | 0.19 | 0.42 | 0.72 | 0.66 | 69.00 | 69.00 | 45.75 | 63.88 | 39.75 | 0.00 | 78.38 | 78.38 | 79.46 | 75.96 | 99.42 | 100.00 |
| Dich.0.55Load.Cross250.8Var.500Size.4Fac.0.70Fcor | 0.28 | 0.28 | 0.16 | 0.28 | 0.51 | 0.12 | 65.25 | 66.00 | 51.75 | 73.75 | 29.38 | 0.00 | 77.96 | 77.17 | 76.75 | 72.62 | 99.33 | 100.00 |
| Dich.0.55Load.Cross250.8Var.1000Size.4Fac.0.00Fcor | 0.89 | 0.89 | 0.45 | 0.51 | 0.72 | 0.69 | 75.88 | 75.75 | 46.00 | 20.88 | 19.88 | 0.00 | 97.92 | 98.04 | 94.33 | 83.25 | 99.62 | 100.00 |
| Dich.0.55Load.Cross250.8Var.1000Size.4Fac.0.50Fcor | 0.74 | 0.74 | 0.29 | 0.51 | 0.80 | 0.69 | 81.38 | 81.38 | 52.50 | 61.00 | 53.62 | 0.00 | 87.71 | 87.71 | 85.38 | 78.21 | 99.67 | 100.00 |
| Dich.0.55Load.Cross250.8Var.1000Size.4Fac.0.70Fcor | 0.44 | 0.44 | 0.18 | 0.40 | 0.72 | 0.34 | 71.50 | 71.00 | 49.38 | 73.62 | 47.12 | 0.00 | 79.75 | 79.83 | 79.50 | 76.88 | 99.50 | 100.00 |
| Dich.0.70Load.000Cross.4Var.300Size.2Fac.0.00Fcor | 0.98 | 0.98 | 0.71 | 0.75 | 0.97 | 1.00 | - | - | - | - | - | - | - | - | - | - | - | - |
| Dich.0.70Load.000Cross.4Var.300Size.2Fac.0.50Fcor | 0.92 | 0.92 | 0.63 | 0.69 | 0.78 | 0.93 | - | - | - | - | - | - | - | - | - | - | - | - |
| Dich.0.70Load.000Cross.4Var.300Size.2Fac.0.70Fcor | 0.76 | 0.75 | 0.51 | 0.60 | 0.36 | 0.18 | - | - | - | - | - | - | - | - | - | - | - | - |
| Dich.0.70Load.000Cross.4Var.500Size.2Fac.0.00Fcor | 0.99 | 1.00 | 0.74 | 0.81 | 1.00 | 1.00 | - | - | - | - | - | - | - | - | - | - | - | - |
| Dich.0.70Load.000Cross.4Var.500Size.2Fac.0.50Fcor | 0.99 | 0.99 | 0.71 | 0.80 | 0.89 | 0.98 | - | - | - | - | - | - | - | - | - | - | - | - |
| Dich.0.70Load.000Cross.4Var.500Size.2Fac.0.70Fcor | 0.90 | 0.90 | 0.59 | 0.67 | 0.49 | 0.29 | - | - | - | - | - | - | - | - | - | - | - | - |
| Dich.0.70Load.000Cross.4Var.1000Size.2Fac.0.00Fcor | 1.00 | 1.00 | 0.77 | 0.86 | 1.00 | 1.00 | - | - | - | - | - | - | - | - | - | - | - | - |
| Dich.0.70Load.000Cross.4Var.1000Size.2Fac.0.50Fcor | 1.00 | 1.00 | 0.64 | 0.85 | 0.94 | 1.00 | - | - | - | - | - | - | - | - | - | - | - | - |
| Dich.0.70Load.000Cross.4Var.1000Size.2Fac.0.70Fcor | 0.98 | 0.98 | 0.66 | 0.79 | 0.58 | 0.42 | - | - | - | - | - | - | - | - | - | - | - | - |
| Dich.0.70Load.000Cross.4Var.300Size.4Fac.0.00Fcor | 0.98 | 0.99 | 0.59 | 0.84 | 0.99 | 1.00 | - | - | - | - | - | - | - | - | - | - | - | - |
| Dich.0.70Load.000Cross.4Var.300Size.4Fac.0.50Fcor | 0.88 | 0.87 | 0.25 | 0.76 | 0.88 | 0.76 | - | - | - | - | - | - | - | - | - | - | - | - |
| Dich.0.70Load.000Cross.4Var.300Size.4Fac.0.70Fcor | 0.62 | 0.61 | 0.17 | 0.54 | 0.59 | 0.04 | - | - | - | - | - | - | - | - | - | - | - | - |
| Dich.0.70Load.000Cross.4Var.500Size.4Fac.0.00Fcor | 0.99 | 1.00 | 0.57 | 0.88 | 1.00 | 1.00 | - | - | - | - | - | - | - | - | - | - | - | - |
| Dich.0.70Load.000Cross.4Var.500Size.4Fac.0.50Fcor | 0.96 | 0.96 | 0.32 | 0.82 | 0.95 | 0.96 | - | - | - | - | - | - | - | - | - | - | - | - |
| Dich.0.70Load.000Cross.4Var.500Size.4Fac.0.70Fcor | 0.78 | 0.78 | 0.21 | 0.68 | 0.79 | 0.07 | - | - | - | - | - | - | - | - | - | - | - | - |
| Dich.0.70Load.000Cross.4Var.1000Size.4Fac.0.00Fcor | 0.98 | 1.00 | 0.54 | 0.90 | 1.00 | 1.00 | - | - | - | - | - | - | - | - | - | - | - | - |
| Dich.0.70Load.000Cross.4Var.1000Size.4Fac.0.50Fcor | 1.00 | 1.00 | 0.30 | 0.84 | 0.98 | 1.00 | - | - | - | - | - | - | - | - | - | - | - | - |
| Dich.0.70Load.000Cross.4Var.1000Size.4Fac.0.70Fcor | 0.91 | 0.91 | 0.21 | 0.70 | 0.88 | 0.17 | - | - | - | - | - | - | - | - | - | - | - | - |
| Dich.0.70Load.000Cross.8Var.300Size.2Fac.0.00Fcor | 0.94 | 0.94 | 0.52 | 0.49 | 0.99 | 1.00 | - | - | - | - | - | - | - | - | - | - | - | - |
| Dich.0.70Load.000Cross.8Var.300Size.2Fac.0.50Fcor | 0.85 | 0.85 | 0.46 | 0.48 | 0.98 | 1.00 | - | - | - | - | - | - | - | - | - | - | - | - |
| Dich.0.70Load.000Cross.8Var.300Size.2Fac.0.70Fcor | 0.67 | 0.67 | 0.35 | 0.43 | 0.94 | 0.89 | - | - | - | - | - | - | - | - | - | - | - | - |
| Dich.0.70Load.000Cross.8Var.500Size.2Fac.0.00Fcor | 0.98 | 0.99 | 0.62 | 0.50 | 1.00 | 1.00 | - | - | - | - | - | - | - | - | - | - | - | - |
| Dich.0.70Load.000Cross.8Var.500Size.2Fac.0.50Fcor | 0.96 | 0.96 | 0.56 | 0.48 | 1.00 | 1.00 | - | - | - | - | - | - | - | - | - | - | - | - |
| Dich.0.70Load.000Cross.8Var.500Size.2Fac.0.70Fcor | 0.86 | 0.86 | 0.47 | 0.51 | 0.98 | 1.00 | - | - | - | - | - | - | - | - | - | - | - | - |
| Dich.0.70Load.000Cross.8Var.1000Size.2Fac.0.00Fcor | 0.99 | 1.00 | 0.65 | 0.59 | 1.00 | 1.00 | - | - | - | - | - | - | - | - | - | - | - | - |
| Dich.0.70Load.000Cross.8Var.1000Size.2Fac.0.50Fcor | 1.00 | 1.00 | 0.61 | 0.58 | 1.00 | 1.00 | - | - | - | - | - | - | - | - | - | - | - | - |
| Dich.0.70Load.000Cross.8Var.1000Size.2Fac.0.70Fcor | 0.98 | 0.98 | 0.55 | 0.55 | 0.99 | 1.00 | - | - | - | - | - | - | - | - | - | - | - | - |
| Dich.0.70Load.000Cross.8Var.300Size.4Fac.0.00Fcor | 0.98 | 0.98 | 0.46 | 0.64 | 1.00 | 1.00 | - | - | - | - | - | - | - | - | - | - | - | - |
| Dich.0.70Load.000Cross.8Var.300Size.4Fac.0.50Fcor | 0.83 | 0.83 | 0.32 | 0.64 | 1.00 | 1.00 | - | - | - | - | - | - | - | - | - | - | - | - |
| Dich.0.70Load.000Cross.8Var.300Size.4Fac.0.70Fcor | 0.61 | 0.61 | 0.15 | 0.55 | 0.93 | 0.62 | - | - | - | - | - | - | - | - | - | - | - | - |
| Dich.0.70Load.000Cross.8Var.500Size.4Fac.0.00Fcor | 0.99 | 1.00 | 0.46 | 0.68 | 1.00 | 1.00 | - | - | - | - | - | - | - | - | - | - | - | - |
| Dich.0.70Load.000Cross.8Var.500Size.4Fac.0.50Fcor | 0.94 | 0.94 | 0.35 | 0.72 | 1.00 | 1.00 | - | - | - | - | - | - | - | - | - | - | - | - |
| Dich.0.70Load.000Cross.8Var.500Size.4Fac.0.70Fcor | 0.77 | 0.77 | 0.20 | 0.63 | 1.00 | 0.90 | - | - | - | - | - | - | - | - | - | - | - | - |
| Dich.0.70Load.000Cross.8Var.1000Size.4Fac.0.00Fcor | 0.99 | 1.00 | 0.55 | 0.70 | 1.00 | 1.00 | - | - | - | - | - | - | - | - | - | - | - | - |
| Dich.0.70Load.000Cross.8Var.1000Size.4Fac.0.50Fcor | 0.99 | 0.99 | 0.33 | 0.69 | 1.00 | 1.00 | - | - | - | - | - | - | - | - | - | - | - | - |
| Dich.0.70Load.000Cross.8Var.1000Size.4Fac.0.70Fcor | 0.96 | 0.96 | 0.25 | 0.70 | 1.00 | 1.00 | - | - | - | - | - | - | - | - | - | - | - | - |
| Dich.0.70Load.125Cross.4Var.300Size.2Fac.0.00Fcor | 0.79 | 0.79 | 0.65 | 0.51 | 0.69 | 0.78 | 54.00 | 53.00 | 48.00 | 26.00 | 96.00 | 10.00 | 95.57 | 95.71 | 88.71 | 89.57 | 85.57 | 100.00 |
| Dich.0.70Load.125Cross.4Var.300Size.2Fac.0.50Fcor | 0.78 | 0.77 | 0.55 | 0.52 | 0.62 | 0.64 | 78.00 | 76.00 | 73.00 | 56.00 | 99.00 | 19.00 | 93.14 | 93.57 | 87.00 | 89.43 | 82.86 | 100.00 |
| Dich.0.70Load.125Cross.4Var.300Size.2Fac.0.70Fcor | 0.72 | 0.72 | 0.50 | 0.49 | 0.20 | 0.06 | 85.00 | 85.00 | 88.00 | 77.00 | 39.00 | 1.00 | 93.43 | 93.43 | 88.29 | 91.29 | 92.43 | 100.00 |
| Dich.0.70Load.125Cross.4Var.500Size.2Fac.0.00Fcor | 0.83 | 0.83 | 0.67 | 0.49 | 0.81 | 0.77 | 53.00 | 53.00 | 55.00 | 15.00 | 100.00 | 5.00 | 98.29 | 98.43 | 92.14 | 93.14 | 90.43 | 100.00 |
| Dich.0.70Load.125Cross.4Var.500Size.2Fac.0.50Fcor | 0.92 | 0.91 | 0.71 | 0.58 | 0.75 | 0.73 | 93.00 | 92.00 | 86.00 | 65.00 | 100.00 | 13.00 | 97.29 | 97.29 | 89.57 | 89.71 | 88.86 | 100.00 |
| Dich.0.70Load.125Cross.4Var.500Size.2Fac.0.70Fcor | 0.86 | 0.86 | 0.65 | 0.52 | 0.22 | 0.05 | 94.00 | 94.00 | 89.00 | 81.00 | 33.00 | 1.00 | 96.86 | 97.00 | 91.57 | 94.29 | 94.86 | 100.00 |
| Dich.0.70Load.125Cross.4Var.1000Size.2Fac.0.00Fcor | 0.84 | 0.84 | 0.78 | 0.51 | 0.92 | 0.75 | 45.00 | 45.00 | 67.00 | 6.00 | 100.00 | 2.00 | 99.86 | 99.86 | 97.00 | 96.00 | 96.14 | 100.00 |
| Dich.0.70Load.125Cross.4Var.1000Size.2Fac.0.50Fcor | 0.99 | 0.99 | 0.82 | 0.64 | 0.90 | 0.78 | 98.00 | 98.00 | 93.00 | 73.00 | 100.00 | 16.00 | 100.00 | 100.00 | 96.43 | 94.71 | 95.29 | 100.00 |
| Dich.0.70Load.125Cross.4Var.1000Size.2Fac.0.70Fcor | 0.95 | 0.95 | 0.76 | 0.58 | 0.14 | 0.05 | 99.00 | 99.00 | 99.00 | 85.00 | 17.00 | 0.00 | 98.00 | 98.00 | 95.00 | 96.00 | 98.86 | 100.00 |
| Dich.0.70Load.125Cross.4Var.300Size.4Fac.0.00Fcor | 0.89 | 0.88 | 0.50 | 0.71 | 0.91 | 0.85 | 60.00 | 58.50 | 34.00 | 28.00 | 84.00 | 12.50 | 97.57 | 98.00 | 90.93 | 92.57 | 95.79 | 100.00 |
| Dich.0.70Load.125Cross.4Var.300Size.4Fac.0.50Fcor | 0.79 | 0.79 | 0.35 | 0.69 | 0.82 | 0.63 | 73.00 | 73.00 | 48.00 | 66.00 | 87.00 | 6.50 | 93.29 | 93.57 | 82.50 | 87.07 | 92.71 | 100.00 |
| Dich.0.70Load.125Cross.4Var.300Size.4Fac.0.70Fcor | 0.57 | 0.57 | 0.24 | 0.54 | 0.56 | 0.04 | 68.00 | 69.00 | 53.50 | 82.00 | 78.50 | 0.00 | 86.93 | 86.93 | 77.79 | 80.79 | 89.57 | 100.00 |
| Dich.0.70Load.125Cross.4Var.500Size.4Fac.0.00Fcor | 0.92 | 0.92 | 0.43 | 0.70 | 0.95 | 0.84 | 67.00 | 66.50 | 30.50 | 19.50 | 94.50 | 4.50 | 99.29 | 99.36 | 88.50 | 93.14 | 97.00 | 100.00 |
| Dich.0.70Load.125Cross.4Var.500Size.4Fac.0.50Fcor | 0.90 | 0.90 | 0.36 | 0.81 | 0.91 | 0.76 | 90.00 | 89.00 | 44.50 | 80.50 | 94.00 | 7.50 | 95.21 | 95.07 | 84.93 | 90.57 | 96.86 | 100.00 |
| Dich.0.70Load.125Cross.4Var.500Size.4Fac.0.70Fcor | 0.69 | 0.68 | 0.32 | 0.61 | 0.72 | 0.04 | 74.00 | 72.50 | 56.50 | 77.50 | 87.50 | 0.00 | 92.07 | 92.36 | 82.36 | 85.93 | 93.71 | 100.00 |
| Dich.0.70Load.125Cross.4Var.1000Size.4Fac.0.00Fcor | 0.95 | 0.94 | 0.41 | 0.70 | 0.97 | 0.84 | 70.00 | 69.50 | 25.50 | 18.00 | 98.00 | 4.50 | 99.93 | 99.93 | 89.50 | 95.07 | 98.14 | 100.00 |
| Dich.0.70Load.125Cross.4Var.1000Size.4Fac.0.50Fcor | 0.97 | 0.97 | 0.38 | 0.81 | 0.94 | 0.83 | 92.50 | 92.50 | 54.00 | 82.50 | 97.50 | 12.50 | 99.50 | 99.50 | 87.00 | 91.79 | 97.86 | 100.00 |
| Dich.0.70Load.125Cross.4Var.1000Size.4Fac.0.70Fcor | 0.82 | 0.82 | 0.36 | 0.74 | 0.79 | 0.17 | 85.00 | 85.00 | 55.50 | 84.00 | 86.00 | 0.50 | 94.14 | 94.14 | 84.71 | 89.64 | 96.07 | 100.00 |
| Dich.0.70Load.125Cross.8Var.300Size.2Fac.0.00Fcor | 0.65 | 0.64 | 0.32 | 0.34 | 0.82 | 0.78 | 54.00 | 50.50 | 33.50 | 24.00 | 67.50 | 10.00 | 86.21 | 86.50 | 75.43 | 68.86 | 94.86 | 100.00 |
| Dich.0.70Load.125Cross.8Var.300Size.2Fac.0.50Fcor | 0.68 | 0.67 | 0.31 | 0.33 | 0.91 | 0.79 | 75.00 | 74.50 | 49.50 | 56.50 | 90.50 | 16.00 | 86.14 | 85.79 | 74.93 | 72.07 | 96.07 | 100.00 |
| Dich.0.70Load.125Cross.8Var.300Size.2Fac.0.70Fcor | 0.53 | 0.53 | 0.22 | 0.31 | 0.92 | 0.57 | 70.50 | 71.00 | 47.50 | 68.50 | 95.50 | 18.00 | 82.93 | 82.64 | 78.71 | 74.86 | 96.79 | 100.00 |
| Dich.0.70Load.125Cross.8Var.500Size.2Fac.0.00Fcor | 0.80 | 0.78 | 0.38 | 0.37 | 0.86 | 0.77 | 57.00 | 54.50 | 28.00 | 20.00 | 70.00 | 5.00 | 94.50 | 94.36 | 81.07 | 73.79 | 96.43 | 100.00 |
| Dich.0.70Load.125Cross.8Var.500Size.2Fac.0.50Fcor | 0.91 | 0.91 | 0.37 | 0.37 | 0.96 | 0.80 | 91.00 | 91.00 | 46.50 | 57.00 | 96.50 | 20.00 | 96.50 | 96.50 | 80.07 | 74.57 | 97.29 | 100.00 |
| Dich.0.70Load.125Cross.8Var.500Size.2Fac.0.70Fcor | 0.75 | 0.75 | 0.20 | 0.36 | 0.97 | 0.76 | 91.50 | 91.50 | 50.00 | 73.00 | 98.00 | 23.00 | 91.14 | 91.14 | 78.64 | 76.79 | 98.79 | 100.00 |
| Dich.0.70Load.125Cross.8Var.1000Size.2Fac.0.00Fcor | 0.87 | 0.86 | 0.48 | 0.38 | 0.88 | 0.76 | 56.00 | 53.50 | 27.00 | 6.50 | 75.50 | 2.50 | 99.50 | 99.50 | 86.36 | 80.36 | 97.29 | 100.00 |
| Dich.0.70Load.125Cross.8Var.1000Size.2Fac.0.50Fcor | 0.96 | 0.96 | 0.38 | 0.42 | 0.98 | 0.81 | 87.00 | 87.00 | 41.50 | 45.00 | 98.50 | 26.00 | 99.93 | 99.93 | 79.07 | 73.71 | 98.29 | 100.00 |
| Dich.0.70Load.125Cross.8Var.1000Size.2Fac.0.70Fcor | 0.96 | 0.96 | 0.27 | 0.49 | 1.00 | 0.83 | 96.50 | 96.50 | 49.00 | 83.00 | 100.00 | 34.00 | 98.79 | 98.79 | 84.14 | 82.43 | 99.64 | 100.00 |
| Dich.0.70Load.125Cross.8Var.300Size.4Fac.0.00Fcor | 0.91 | 0.91 | 0.45 | 0.56 | 0.91 | 0.86 | 74.50 | 72.75 | 47.50 | 15.75 | 43.25 | 13.50 | 95.68 | 95.75 | 90.61 | 76.96 | 99.96 | 100.00 |
| Dich.0.70Load.125Cross.8Var.300Size.4Fac.0.50Fcor | 0.76 | 0.76 | 0.31 | 0.57 | 0.96 | 0.87 | 83.00 | 82.75 | 54.00 | 63.25 | 82.75 | 21.50 | 85.75 | 85.93 | 85.21 | 77.68 | 99.79 | 100.00 |
| Dich.0.70Load.125Cross.8Var.300Size.4Fac.0.70Fcor | 0.54 | 0.54 | 0.20 | 0.47 | 0.88 | 0.52 | 78.00 | 78.00 | 55.00 | 74.00 | 75.00 | 6.75 | 76.21 | 76.36 | 76.43 | 75.39 | 99.18 | 100.00 |
| Dich.0.70Load.125Cross.8Var.500Size.4Fac.0.00Fcor | 0.94 | 0.94 | 0.45 | 0.54 | 0.94 | 0.86 | 74.25 | 74.25 | 51.50 | 13.50 | 65.25 | 10.25 | 98.36 | 98.46 | 90.86 | 78.50 | 99.96 | 100.00 |
| Dich.0.70Load.125Cross.8Var.500Size.4Fac.0.50Fcor | 0.91 | 0.91 | 0.37 | 0.60 | 0.99 | 0.88 | 94.25 | 94.25 | 52.25 | 62.75 | 95.25 | 23.75 | 94.75 | 94.75 | 88.04 | 76.89 | 99.89 | 100.00 |
| Dich.0.70Load.125Cross.8Var.500Size.4Fac.0.70Fcor | 0.73 | 0.73 | 0.26 | 0.58 | 0.97 | 0.72 | 89.25 | 89.25 | 50.75 | 76.75 | 89.50 | 12.00 | 82.57 | 82.57 | 84.79 | 78.00 | 99.93 | 100.00 |
| Dich.0.70Load.125Cross.8Var.1000Size.4Fac.0.00Fcor | 0.96 | 0.96 | 0.40 | 0.59 | 0.98 | 0.85 | 78.00 | 78.25 | 53.00 | 10.50 | 90.50 | 9.25 | 99.61 | 99.79 | 90.64 | 80.68 | 100.00 | 100.00 |
| Dich.0.70Load.125Cross.8Var.1000Size.4Fac.0.50Fcor | 0.98 | 0.98 | 0.43 | 0.65 | 0.99 | 0.89 | 95.25 | 95.50 | 56.50 | 62.50 | 98.00 | 28.75 | 99.57 | 99.57 | 92.32 | 81.46 | 100.00 | 100.00 |
| Dich.0.70Load.125Cross.8Var.1000Size.4Fac.0.70Fcor | 0.93 | 0.93 | 0.39 | 0.62 | 0.99 | 0.85 | 97.75 | 97.75 | 61.25 | 80.00 | 97.50 | 12.00 | 96.18 | 96.21 | 90.04 | 83.32 | 99.89 | 100.00 |
| Dich.0.70Load.Cross250.4Var.300Size.2Fac.0.00Fcor | 0.50 | 0.48 | 0.37 | 0.29 | 0.38 | 0.55 | 34.50 | 33.00 | 35.50 | 28.00 | 79.50 | 4.50 | 92.17 | 91.17 | 81.33 | 82.67 | 66.83 | 100.00 |
| Dich.0.70Load.Cross250.4Var.300Size.2Fac.0.50Fcor | 0.57 | 0.57 | 0.36 | 0.35 | 0.44 | 0.23 | 57.00 | 57.00 | 48.50 | 45.00 | 65.00 | 2.50 | 89.17 | 89.17 | 79.17 | 84.50 | 87.00 | 100.00 |
| Dich.0.70Load.Cross250.4Var.300Size.2Fac.0.70Fcor | 0.46 | 0.46 | 0.28 | 0.30 | 0.05 | 0.01 | 55.00 | 55.00 | 49.50 | 47.50 | 6.50 | 0.00 | 89.83 | 89.83 | 85.33 | 90.17 | 99.17 | 100.00 |
| Dich.0.70Load.Cross250.4Var.500Size.2Fac.0.00Fcor | 0.53 | 0.51 | 0.35 | 0.30 | 0.48 | 0.54 | 35.50 | 32.00 | 35.00 | 20.00 | 85.00 | 3.50 | 92.83 | 93.67 | 82.50 | 90.00 | 74.00 | 100.00 |
| Dich.0.70Load.Cross250.4Var.500Size.2Fac.0.50Fcor | 0.81 | 0.81 | 0.43 | 0.45 | 0.57 | 0.41 | 82.50 | 83.00 | 60.00 | 55.50 | 78.50 | 9.50 | 94.33 | 94.17 | 78.67 | 85.33 | 87.67 | 100.00 |
| Dich.0.70Load.Cross250.4Var.500Size.2Fac.0.70Fcor | 0.75 | 0.75 | 0.44 | 0.41 | 0.02 | 0.02 | 76.50 | 76.50 | 61.50 | 52.50 | 3.00 | 0.00 | 96.33 | 96.33 | 87.50 | 93.33 | 99.67 | 100.00 |
| Dich.0.70Load.Cross250.4Var.1000Size.2Fac.0.00Fcor | 0.69 | 0.67 | 0.45 | 0.28 | 0.54 | 0.53 | 56.00 | 51.50 | 35.50 | 6.00 | 95.50 | 1.00 | 98.50 | 98.83 | 88.50 | 95.33 | 75.00 | 100.00 |
| Dich.0.70Load.Cross250.4Var.1000Size.2Fac.0.50Fcor | 0.97 | 0.97 | 0.45 | 0.48 | 0.62 | 0.50 | 98.50 | 98.50 | 50.50 | 54.50 | 80.00 | 11.00 | 99.33 | 99.33 | 80.17 | 87.17 | 91.67 | 100.00 |
| Dich.0.70Load.Cross250.4Var.1000Size.2Fac.0.70Fcor | 0.95 | 0.95 | 0.72 | 0.61 | 0.02 | 0.01 | 96.50 | 96.50 | 90.50 | 71.50 | 2.00 | 0.00 | 98.67 | 98.67 | 92.50 | 97.33 | 100.00 | 100.00 |
| Dich.0.70Load.Cross250.4Var.300Size.4Fac.0.00Fcor | 0.75 | 0.75 | 0.41 | 0.61 | 0.81 | 0.72 | 53.75 | 54.75 | 52.75 | 39.50 | 81.25 | 8.75 | 93.58 | 93.83 | 90.17 | 88.58 | 89.42 | 100.00 |
| Dich.0.70Load.Cross250.4Var.300Size.4Fac.0.50Fcor | 0.70 | 0.70 | 0.32 | 0.65 | 0.72 | 0.48 | 69.00 | 68.75 | 54.00 | 72.25 | 86.50 | 6.50 | 89.75 | 89.67 | 85.25 | 84.58 | 91.50 | 100.00 |
| Dich.0.70Load.Cross250.4Var.300Size.4Fac.0.70Fcor | 0.51 | 0.51 | 0.26 | 0.51 | 0.49 | 0.02 | 61.00 | 62.75 | 55.50 | 70.00 | 67.25 | 0.00 | 89.25 | 88.67 | 84.00 | 85.67 | 93.33 | 100.00 |
| Dich.0.70Load.Cross250.4Var.500Size.4Fac.0.00Fcor | 0.85 | 0.84 | 0.43 | 0.56 | 0.88 | 0.71 | 69.00 | 68.50 | 58.25 | 29.50 | 94.25 | 5.75 | 96.67 | 96.58 | 88.33 | 91.08 | 92.17 | 100.00 |
| Dich.0.70Load.Cross250.4Var.500Size.4Fac.0.50Fcor | 0.87 | 0.88 | 0.43 | 0.72 | 0.87 | 0.62 | 85.50 | 85.50 | 63.75 | 72.75 | 94.25 | 10.25 | 95.00 | 95.17 | 90.25 | 90.08 | 96.33 | 100.00 |
| Dich.0.70Load.Cross250.4Var.500Size.4Fac.0.70Fcor | 0.65 | 0.64 | 0.34 | 0.63 | 0.54 | 0.04 | 73.00 | 72.00 | 56.50 | 81.25 | 70.00 | 0.50 | 91.25 | 91.42 | 87.33 | 87.33 | 96.50 | 100.00 |
| Dich.0.70Load.Cross250.4Var.1000Size.4Fac.0.00Fcor | 0.92 | 0.92 | 0.45 | 0.56 | 0.94 | 0.70 | 76.75 | 77.25 | 63.75 | 23.25 | 99.00 | 4.00 | 99.67 | 99.67 | 88.33 | 97.75 | 95.42 | 100.00 |
| Dich.0.70Load.Cross250.4Var.1000Size.4Fac.0.50Fcor | 0.98 | 0.98 | 0.51 | 0.77 | 0.92 | 0.69 | 96.00 | 96.00 | 68.75 | 75.25 | 97.00 | 12.75 | 99.75 | 99.75 | 93.00 | 91.67 | 98.58 | 100.00 |
| Dich.0.70Load.Cross250.4Var.1000Size.4Fac.0.70Fcor | 0.84 | 0.84 | 0.37 | 0.73 | 0.59 | 0.08 | 88.50 | 88.50 | 57.25 | 88.75 | 70.00 | 0.75 | 95.83 | 95.83 | 90.75 | 92.00 | 97.17 | 100.00 |
| Dich.0.70Load.Cross250.8Var.300Size.2Fac.0.00Fcor | 0.65 | 0.64 | 0.32 | 0.34 | 0.82 | 0.78 | 54.00 | 50.50 | 33.50 | 24.00 | 67.50 | 10.00 | 86.21 | 86.50 | 75.43 | 68.86 | 94.86 | 100.00 |
| Dich.0.70Load.Cross250.8Var.300Size.2Fac.0.50Fcor | 0.68 | 0.67 | 0.31 | 0.33 | 0.91 | 0.79 | 75.00 | 74.50 | 49.50 | 56.50 | 90.50 | 16.00 | 86.14 | 85.79 | 74.93 | 72.07 | 96.07 | 100.00 |
| Dich.0.70Load.Cross250.8Var.300Size.2Fac.0.70Fcor | 0.53 | 0.53 | 0.22 | 0.31 | 0.92 | 0.57 | 70.50 | 71.00 | 47.50 | 68.50 | 95.50 | 18.00 | 82.93 | 82.64 | 78.71 | 74.86 | 96.79 | 100.00 |
| Dich.0.70Load.Cross250.8Var.500Size.2Fac.0.00Fcor | 0.80 | 0.78 | 0.38 | 0.37 | 0.86 | 0.77 | 57.00 | 54.50 | 28.00 | 20.00 | 70.00 | 5.00 | 94.50 | 94.36 | 81.07 | 73.79 | 96.43 | 100.00 |
| Dich.0.70Load.Cross250.8Var.500Size.2Fac.0.50Fcor | 0.91 | 0.91 | 0.37 | 0.37 | 0.96 | 0.80 | 91.00 | 91.00 | 46.50 | 57.00 | 96.50 | 20.00 | 96.50 | 96.50 | 80.07 | 74.57 | 97.29 | 100.00 |
| Dich.0.70Load.Cross250.8Var.500Size.2Fac.0.70Fcor | 0.75 | 0.75 | 0.20 | 0.36 | 0.97 | 0.76 | 91.50 | 91.50 | 50.00 | 73.00 | 98.00 | 23.00 | 91.14 | 91.14 | 78.64 | 76.79 | 98.79 | 100.00 |
| Dich.0.70Load.Cross250.8Var.1000Size.2Fac.0.00Fcor | 0.87 | 0.86 | 0.48 | 0.38 | 0.88 | 0.76 | 56.00 | 53.50 | 27.00 | 6.50 | 75.50 | 2.50 | 99.50 | 99.50 | 86.36 | 80.36 | 97.29 | 100.00 |
| Dich.0.70Load.Cross250.8Var.1000Size.2Fac.0.50Fcor | 0.96 | 0.96 | 0.38 | 0.42 | 0.98 | 0.81 | 87.00 | 87.00 | 41.50 | 45.00 | 98.50 | 26.00 | 99.93 | 99.93 | 79.07 | 73.71 | 98.29 | 100.00 |
| Dich.0.70Load.Cross250.8Var.1000Size.2Fac.0.70Fcor | 0.96 | 0.96 | 0.27 | 0.49 | 1.00 | 0.83 | 96.50 | 96.50 | 49.00 | 83.00 | 100.00 | 34.00 | 98.79 | 98.79 | 84.14 | 82.43 | 99.64 | 100.00 |
| Dich.0.70Load.Cross250.8Var.300Size.4Fac.0.00Fcor | 0.76 | 0.76 | 0.31 | 0.46 | 0.74 | 0.73 | 64.88 | 64.25 | 43.38 | 28.12 | 26.50 | 12.00 | 87.96 | 87.50 | 86.58 | 74.12 | 98.92 | 100.00 |
| Dich.0.70Load.Cross250.8Var.300Size.4Fac.0.50Fcor | 0.64 | 0.64 | 0.26 | 0.45 | 0.82 | 0.75 | 71.88 | 70.75 | 46.88 | 59.88 | 56.25 | 19.12 | 80.38 | 80.83 | 82.33 | 72.79 | 98.96 | 100.00 |
| Dich.0.70Load.Cross250.8Var.300Size.4Fac.0.70Fcor | 0.44 | 0.44 | 0.18 | 0.39 | 0.74 | 0.36 | 64.00 | 63.50 | 44.50 | 67.50 | 48.88 | 4.50 | 79.04 | 79.83 | 78.21 | 76.83 | 99.38 | 99.96 |
| Dich.0.70Load.Cross250.8Var.500Size.4Fac.0.00Fcor | 0.81 | 0.81 | 0.32 | 0.48 | 0.77 | 0.71 | 68.25 | 67.50 | 51.62 | 23.50 | 40.12 | 8.75 | 91.21 | 91.46 | 86.75 | 75.75 | 98.62 | 100.00 |
| Dich.0.70Load.Cross250.8Var.500Size.4Fac.0.50Fcor | 0.79 | 0.78 | 0.37 | 0.50 | 0.86 | 0.76 | 81.12 | 80.38 | 50.00 | 60.38 | 62.88 | 24.25 | 90.38 | 90.62 | 87.04 | 71.25 | 99.50 | 100.00 |
| Dich.0.70Load.Cross250.8Var.500Size.4Fac.0.70Fcor | 0.67 | 0.67 | 0.25 | 0.48 | 0.85 | 0.59 | 78.50 | 78.25 | 48.75 | 71.50 | 64.25 | 10.50 | 85.33 | 85.42 | 83.92 | 77.62 | 99.75 | 100.00 |
| Dich.0.70Load.Cross250.8Var.1000Size.4Fac.0.00Fcor | 0.91 | 0.90 | 0.35 | 0.47 | 0.84 | 0.72 | 77.25 | 75.75 | 51.25 | 17.62 | 57.38 | 9.88 | 98.29 | 98.33 | 88.79 | 80.67 | 99.42 | 100.00 |
| Dich.0.70Load.Cross250.8Var.1000Size.4Fac.0.50Fcor | 0.96 | 0.96 | 0.44 | 0.54 | 0.88 | 0.78 | 91.25 | 91.38 | 53.62 | 53.88 | 72.12 | 30.62 | 99.38 | 99.42 | 94.04 | 76.96 | 99.25 | 100.00 |
| Dich.0.70Load.Cross250.8Var.1000Size.4Fac.0.70Fcor | 0.89 | 0.89 | 0.32 | 0.55 | 0.90 | 0.74 | 91.12 | 91.12 | 48.75 | 72.00 | 76.12 | 17.00 | 95.67 | 95.67 | 91.00 | 80.50 | 99.83 | 100.00 |
| Note. Cont= Continuous; Ord= Ordinal; Dich= Dichotomous; Load= Size of primary factor loadings; Cross= Proportion of observed variables with substantive cross-loadings; Var = Number of variables per factor; Size = Sample size; Fac = Number of factors; Fcor = Factor correlation; CPSigMod = CP algorithm with maximisation of the signed fuzzy modularity for signed weighted networks; CPMod = CP algorithm with maximisation of the fuzzy modularity for signed weighted networks; CPRat = CP algorithm with minimisation of the ratio between the two largest communities when the ratio is above or equal 2; CPEnt = CP algorithm with maximisation of entropy; Walk-Ov = Walktrap algorithm with overlapping nodes identified through network loadings >= \|.15\|; EFA-Ov = Exploratory Factor Analysis with overlapping nodes identified through factor loadings >= \|.40\|. | | | | | | | | | | | | | | | | | | |

Supplementary Table 2: Outcomes of the simulation study (Isolated Nodes, Mean Bias Error, Mean Absolute Error).

|  | Isolated Nodes | | | | Mean Bias Error | | | | | | Mean Absolute Error | | | | | |
| --- | --- | --- | --- | --- | --- | --- | --- | --- | --- | --- | --- | --- | --- | --- | --- | --- |
| Condition | CPSigMod | CPMod | CPRat | CPEnt | CPSigMod | CPMod | CPRat | CPEnt | Walk-Ov | EFA-Ov | CPSigMod | CPMod | CPRat | CPEnt | Walk-Ov | EFA-Ov |
| Cont.0.40Load.000Cross.4Var.300Size.2Fac.0.00Fcor | 3.59 | 3.59 | 4.11 | 4.40 | -0.79 | -0.79 | -0.86 | -0.92 | -0.37 | 0.00 | 0.85 | 0.85 | 0.94 | 1.00 | 0.43 | 0.00 |
| Cont.0.40Load.000Cross.4Var.300Size.2Fac.0.50Fcor | 4.27 | 4.27 | 4.89 | 5.19 | -0.96 | -0.96 | -1.09 | -1.13 | -0.47 | -0.35 | 1.04 | 1.04 | 1.19 | 1.27 | 0.59 | 0.35 |
| Cont.0.40Load.000Cross.4Var.300Size.2Fac.0.70Fcor | 4.13 | 4.13 | 5.29 | 5.56 | -1.00 | -1.00 | -1.17 | -1.21 | -0.57 | -0.90 | 1.06 | 1.06 | 1.23 | 1.27 | 0.69 | 0.90 |
| Cont.0.40Load.000Cross.4Var.500Size.2Fac.0.00Fcor | 0.59 | 0.59 | 1.17 | 1.32 | -0.05 | -0.05 | -0.12 | -0.15 | 0.01 | 0.00 | 0.17 | 0.17 | 0.28 | 0.31 | 0.01 | 0.00 |
| Cont.0.40Load.000Cross.4Var.500Size.2Fac.0.50Fcor | 1.36 | 1.36 | 2.08 | 2.37 | -0.14 | -0.14 | -0.26 | -0.31 | -0.03 | -0.44 | 0.30 | 0.30 | 0.50 | 0.55 | 0.15 | 0.44 |
| Cont.0.40Load.000Cross.4Var.500Size.2Fac.0.70Fcor | 1.23 | 1.23 | 4.50 | 4.79 | -0.45 | -0.45 | -0.91 | -0.97 | -0.33 | -0.94 | 0.67 | 0.67 | 1.13 | 1.19 | 0.37 | 0.94 |
| Cont.0.40Load.000Cross.4Var.1000Size.2Fac.0.00Fcor | 0.03 | 0.03 | 0.32 | 0.75 | 0.00 | 0.00 | 0.05 | -0.02 | 0.00 | 0.00 | 0.00 | 0.00 | 0.05 | 0.18 | 0.00 | 0.00 |
| Cont.0.40Load.000Cross.4Var.1000Size.2Fac.0.50Fcor | 0.82 | 0.82 | 1.44 | 1.86 | -0.13 | -0.13 | -0.20 | -0.28 | 0.00 | -0.15 | 0.19 | 0.19 | 0.32 | 0.42 | 0.00 | 0.15 |
| Cont.0.40Load.000Cross.4Var.1000Size.2Fac.0.70Fcor | 1.03 | 1.03 | 3.90 | 4.15 | -0.43 | -0.43 | -0.82 | -0.86 | -0.26 | -0.84 | 0.57 | 0.57 | 0.94 | 1.00 | 0.26 | 0.84 |
| Cont.0.40Load.000Cross.4Var.300Size.4Fac.0.00Fcor | 12.57 | 12.57 | 13.32 | 13.08 | -2.99 | -2.99 | -3.19 | -3.14 | -1.75 | -0.10 | 2.99 | 2.99 | 3.19 | 3.14 | 1.91 | 0.14 |
| Cont.0.40Load.000Cross.4Var.300Size.4Fac.0.50Fcor | 13.63 | 13.63 | 13.69 | 13.72 | -3.30 | -3.30 | -3.36 | -3.31 | -2.40 | -1.98 | 3.36 | 3.36 | 3.40 | 3.37 | 2.48 | 1.98 |
| Cont.0.40Load.000Cross.4Var.300Size.4Fac.0.70Fcor | 11.24 | 11.24 | 11.61 | 11.95 | -2.91 | -2.91 | -2.99 | -2.95 | -2.11 | -2.91 | 2.95 | 2.95 | 3.03 | 2.97 | 2.17 | 2.91 |
| Cont.0.40Load.000Cross.4Var.500Size.4Fac.0.00Fcor | 3.64 | 3.64 | 4.88 | 4.12 | -0.67 | -0.67 | -0.96 | -0.71 | -0.09 | -0.02 | 0.85 | 0.85 | 1.10 | 0.91 | 0.13 | 0.02 |
| Cont.0.40Load.000Cross.4Var.500Size.4Fac.0.50Fcor | 6.48 | 6.48 | 6.35 | 6.92 | -1.79 | -1.79 | -2.01 | -1.76 | -0.70 | -1.84 | 1.81 | 1.81 | 2.03 | 1.78 | 0.84 | 1.84 |
| Cont.0.40Load.000Cross.4Var.500Size.4Fac.0.70Fcor | 6.28 | 6.28 | 6.40 | 7.54 | -1.86 | -1.86 | -2.02 | -1.82 | -1.31 | -2.89 | 1.94 | 1.94 | 2.08 | 1.90 | 1.37 | 2.89 |
| Cont.0.40Load.000Cross.4Var.1000Size.4Fac.0.00Fcor | 0.50 | 0.50 | 2.85 | 1.06 | -0.02 | -0.02 | -0.45 | -0.05 | 0.00 | 0.00 | 0.12 | 0.12 | 0.59 | 0.23 | 0.00 | 0.00 |
| Cont.0.40Load.000Cross.4Var.1000Size.4Fac.0.50Fcor | 4.02 | 4.02 | 4.02 | 5.55 | -1.36 | -1.36 | -1.59 | -1.38 | -0.27 | -1.04 | 1.44 | 1.44 | 1.63 | 1.46 | 0.39 | 1.04 |
| Cont.0.40Load.000Cross.4Var.1000Size.4Fac.0.70Fcor | 4.06 | 4.15 | 7.42 | 8.46 | -2.06 | -2.06 | -2.35 | -2.34 | -1.19 | -2.88 | 2.10 | 2.10 | 2.39 | 2.38 | 1.23 | 2.88 |
| Cont.0.40Load.000Cross.8Var.300Size.2Fac.0.00Fcor | 7.56 | 7.56 | 9.13 | 8.99 | -0.61 | -0.61 | -0.46 | -0.27 | -0.50 | 0.01 | 1.05 | 1.05 | 1.26 | 1.45 | 0.68 | 0.01 |
| Cont.0.40Load.000Cross.8Var.300Size.2Fac.0.50Fcor | 9.92 | 9.92 | 9.97 | 10.62 | -0.75 | -0.75 | -0.75 | -0.62 | -0.58 | -0.02 | 1.35 | 1.35 | 1.37 | 1.52 | 1.00 | 0.02 |
| Cont.0.40Load.000Cross.8Var.300Size.2Fac.0.70Fcor | 9.23 | 9.23 | 9.23 | 9.98 | -0.45 | -0.45 | -0.59 | -0.34 | -0.36 | -0.71 | 1.41 | 1.41 | 1.33 | 1.62 | 1.04 | 0.71 |
| Cont.0.40Load.000Cross.8Var.500Size.2Fac.0.00Fcor | 0.23 | 0.24 | 3.24 | 3.69 | 0.08 | 0.07 | 0.40 | 0.63 | 0.00 | 0.00 | 0.08 | 0.07 | 0.40 | 0.63 | 0.00 | 0.00 |
| Cont.0.40Load.000Cross.8Var.500Size.2Fac.0.50Fcor | 2.22 | 2.22 | 2.77 | 4.12 | 0.21 | 0.21 | 0.31 | 0.57 | -0.08 | 0.00 | 0.49 | 0.49 | 0.55 | 0.83 | 0.12 | 0.00 |
| Cont.0.40Load.000Cross.8Var.500Size.2Fac.0.70Fcor | 4.24 | 4.24 | 4.18 | 5.65 | 0.23 | 0.23 | 0.17 | 0.50 | 0.02 | -0.47 | 0.81 | 0.81 | 0.69 | 1.02 | 0.30 | 0.47 |
| Cont.0.40Load.000Cross.8Var.1000Size.2Fac.0.00Fcor | 0.02 | 0.02 | 2.58 | 3.14 | 0.02 | 0.02 | 0.60 | 0.80 | 0.00 | 0.00 | 0.02 | 0.02 | 0.60 | 0.80 | 0.00 | 0.00 |
| Cont.0.40Load.000Cross.8Var.1000Size.2Fac.0.50Fcor | 0.60 | 0.60 | 2.35 | 3.12 | 0.21 | 0.21 | 0.46 | 0.71 | 0.00 | 0.00 | 0.23 | 0.23 | 0.50 | 0.77 | 0.00 | 0.00 |
| Cont.0.40Load.000Cross.8Var.1000Size.2Fac.0.70Fcor | 2.58 | 2.63 | 3.10 | 4.16 | 0.48 | 0.48 | 0.47 | 0.71 | 0.02 | -0.16 | 0.66 | 0.66 | 0.59 | 0.85 | 0.02 | 0.16 |
| Cont.0.40Load.000Cross.8Var.300Size.4Fac.0.00Fcor | 26.75 | 26.75 | 28.54 | 27.07 | -2.94 | -2.94 | -3.18 | -2.86 | -2.18 | 0.03 | 3.32 | 3.32 | 3.40 | 3.44 | 2.52 | 0.03 |
| Cont.0.40Load.000Cross.8Var.300Size.4Fac.0.50Fcor | 30.83 | 30.83 | 31.04 | 30.95 | -3.69 | -3.69 | -3.81 | -3.73 | -2.89 | -0.72 | 3.79 | 3.79 | 3.83 | 3.83 | 3.29 | 0.72 |
| Cont.0.40Load.000Cross.8Var.300Size.4Fac.0.70Fcor | 28.88 | 28.88 | 28.90 | 29.01 | -3.21 | -3.21 | -3.48 | -3.26 | -2.27 | -2.61 | 3.45 | 3.45 | 3.48 | 3.50 | 2.73 | 2.61 |
| Cont.0.40Load.000Cross.8Var.500Size.4Fac.0.00Fcor | 1.78 | 1.80 | 11.08 | 5.37 | 0.42 | 0.41 | -0.48 | 1.41 | -0.08 | 0.00 | 0.62 | 0.61 | 1.32 | 1.59 | 0.08 | 0.00 |
| Cont.0.40Load.000Cross.8Var.500Size.4Fac.0.50Fcor | 13.96 | 13.96 | 12.83 | 13.95 | -0.53 | -0.53 | -1.08 | -0.22 | -0.13 | -0.14 | 1.73 | 1.73 | 1.74 | 1.86 | 0.75 | 0.14 |
| Cont.0.40Load.000Cross.8Var.500Size.4Fac.0.70Fcor | 14.97 | 14.97 | 14.57 | 15.53 | -0.63 | -0.63 | -1.31 | -0.48 | -0.18 | -2.46 | 1.87 | 1.87 | 1.95 | 1.94 | 0.96 | 2.46 |
| Cont.0.40Load.000Cross.8Var.1000Size.4Fac.0.00Fcor | 0.18 | 0.18 | 9.72 | 3.94 | 0.11 | 0.11 | -0.35 | 0.86 | 0.00 | 0.00 | 0.11 | 0.11 | 0.99 | 0.86 | 0.00 | 0.00 |
| Cont.0.40Load.000Cross.8Var.1000Size.4Fac.0.50Fcor | 6.44 | 6.44 | 6.85 | 6.65 | 0.01 | 0.01 | -0.97 | 0.48 | 0.03 | 0.00 | 0.77 | 0.77 | 1.39 | 0.78 | 0.05 | 0.00 |
| Cont.0.40Load.000Cross.8Var.1000Size.4Fac.0.70Fcor | 12.91 | 12.91 | 9.68 | 11.89 | -0.43 | -0.43 | -1.01 | -0.18 | -0.03 | -1.33 | 1.29 | 1.29 | 1.29 | 1.24 | 0.47 | 1.33 |
| Cont.0.40Load.125Cross.4Var.300Size.2Fac.0.00Fcor | 4.77 | 4.77 | 5.67 | 5.94 | -1.07 | -1.07 | -1.27 | -1.31 | -0.46 | -0.05 | 1.19 | 1.19 | 1.41 | 1.47 | 0.60 | 0.05 |
| Cont.0.40Load.125Cross.4Var.300Size.2Fac.0.50Fcor | 2.98 | 3.00 | 4.49 | 4.84 | -0.70 | -0.70 | -0.91 | -0.96 | -0.20 | -0.71 | 0.82 | 0.82 | 1.05 | 1.10 | 0.44 | 0.71 |
| Cont.0.40Load.125Cross.4Var.300Size.2Fac.0.70Fcor | 2.28 | 2.27 | 4.63 | 5.06 | -0.67 | -0.68 | -0.96 | -1.04 | -0.43 | -0.98 | 0.75 | 0.76 | 1.08 | 1.18 | 0.57 | 0.98 |
| Cont.0.40Load.125Cross.4Var.500Size.2Fac.0.00Fcor | 1.86 | 1.79 | 2.57 | 3.47 | -0.40 | -0.39 | -0.56 | -0.70 | -0.10 | 0.00 | 0.44 | 0.43 | 0.60 | 0.76 | 0.18 | 0.00 |
| Cont.0.40Load.125Cross.4Var.500Size.2Fac.0.50Fcor | 1.17 | 1.17 | 3.38 | 4.15 | -0.31 | -0.31 | -0.64 | -0.79 | -0.05 | -0.60 | 0.45 | 0.45 | 0.80 | 0.97 | 0.09 | 0.60 |
| Cont.0.40Load.125Cross.4Var.500Size.2Fac.0.70Fcor | 1.09 | 1.10 | 4.98 | 5.26 | -0.59 | -0.59 | -1.10 | -1.16 | -0.53 | -0.96 | 0.63 | 0.63 | 1.14 | 1.20 | 0.55 | 0.96 |
| Cont.0.40Load.125Cross.4Var.1000Size.2Fac.0.00Fcor | 0.14 | 0.16 | 0.32 | 1.32 | 0.00 | 0.00 | 0.04 | -0.10 | 0.00 | 0.00 | 0.04 | 0.04 | 0.08 | 0.28 | 0.00 | 0.00 |
| Cont.0.40Load.125Cross.4Var.1000Size.2Fac.0.50Fcor | 0.90 | 0.90 | 2.76 | 3.40 | -0.28 | -0.28 | -0.53 | -0.65 | -0.05 | -0.36 | 0.36 | 0.36 | 0.63 | 0.75 | 0.05 | 0.36 |
| Cont.0.40Load.125Cross.4Var.1000Size.2Fac.0.70Fcor | 0.59 | 0.59 | 5.42 | 5.56 | -0.70 | -0.70 | -1.26 | -1.29 | -0.60 | -0.98 | 0.72 | 0.72 | 1.26 | 1.31 | 0.62 | 0.98 |
| Cont.0.40Load.125Cross.4Var.300Size.4Fac.0.00Fcor | 13.15 | 13.15 | 13.65 | 13.48 | -3.12 | -3.12 | -3.28 | -3.21 | -2.03 | -0.10 | 3.20 | 3.20 | 3.36 | 3.29 | 2.09 | 0.14 |
| Cont.0.40Load.125Cross.4Var.300Size.4Fac.0.50Fcor | 10.98 | 11.07 | 11.42 | 12.21 | -2.86 | -2.86 | -3.14 | -2.97 | -1.67 | -2.27 | 2.86 | 2.86 | 3.14 | 2.97 | 1.77 | 2.27 |
| Cont.0.40Load.125Cross.4Var.300Size.4Fac.0.70Fcor | 7.35 | 7.39 | 8.28 | 9.30 | -2.22 | -2.22 | -2.38 | -2.29 | -1.69 | -2.94 | 2.30 | 2.30 | 2.44 | 2.37 | 1.69 | 2.94 |
| Cont.0.40Load.125Cross.4Var.500Size.4Fac.0.00Fcor | 4.84 | 4.85 | 5.53 | 5.62 | -1.11 | -1.09 | -1.31 | -1.11 | -0.62 | -0.05 | 1.21 | 1.21 | 1.41 | 1.31 | 0.66 | 0.07 |
| Cont.0.40Load.125Cross.4Var.500Size.4Fac.0.50Fcor | 5.50 | 5.50 | 4.99 | 6.45 | -1.75 | -1.75 | -1.92 | -1.71 | -0.70 | -1.98 | 1.79 | 1.79 | 1.92 | 1.75 | 0.80 | 1.98 |
| Cont.0.40Load.125Cross.4Var.500Size.4Fac.0.70Fcor | 4.27 | 4.27 | 6.16 | 7.39 | -2.13 | -2.13 | -2.15 | -2.12 | -1.22 | -2.96 | 2.13 | 2.13 | 2.15 | 2.12 | 1.30 | 2.96 |
| Cont.0.40Load.125Cross.4Var.1000Size.4Fac.0.00Fcor | 1.08 | 1.02 | 2.88 | 1.67 | -0.18 | -0.18 | -0.77 | -0.19 | -0.06 | 0.00 | 0.38 | 0.38 | 0.91 | 0.45 | 0.06 | 0.00 |
| Cont.0.40Load.125Cross.4Var.1000Size.4Fac.0.50Fcor | 4.58 | 4.60 | 5.14 | 6.42 | -1.70 | -1.70 | -1.90 | -1.75 | -0.63 | -1.25 | 1.72 | 1.72 | 1.92 | 1.77 | 0.69 | 1.25 |
| Cont.0.40Load.125Cross.4Var.1000Size.4Fac.0.70Fcor | 4.23 | 4.23 | 8.57 | 9.17 | -2.29 | -2.29 | -2.51 | -2.47 | -1.24 | -2.92 | 2.31 | 2.31 | 2.53 | 2.49 | 1.28 | 2.92 |
| Cont.0.40Load.125Cross.8Var.300Size.2Fac.0.00Fcor | 10.09 | 10.10 | 10.45 | 10.82 | -0.96 | -0.95 | -0.77 | -0.63 | -0.44 | 0.00 | 1.26 | 1.27 | 1.51 | 1.65 | 0.94 | 0.00 |
| Cont.0.40Load.125Cross.8Var.300Size.2Fac.0.50Fcor | 7.23 | 7.24 | 7.48 | 8.52 | -0.15 | -0.15 | -0.24 | 0.02 | -0.04 | -0.19 | 1.05 | 1.05 | 1.08 | 1.34 | 0.58 | 0.19 |
| Cont.0.40Load.125Cross.8Var.300Size.2Fac.0.70Fcor | 5.57 | 5.57 | 5.61 | 7.14 | 0.17 | 0.17 | 0.03 | 0.34 | 0.27 | -0.83 | 0.85 | 0.85 | 0.83 | 1.14 | 0.51 | 0.83 |
| Cont.0.40Load.125Cross.8Var.500Size.2Fac.0.00Fcor | 1.62 | 1.62 | 3.41 | 4.40 | 0.08 | 0.10 | 0.41 | 0.88 | 0.10 | 0.00 | 0.32 | 0.34 | 0.65 | 1.12 | 0.32 | 0.00 |
| Cont.0.40Load.125Cross.8Var.500Size.2Fac.0.50Fcor | 2.82 | 2.92 | 3.90 | 5.43 | 0.12 | 0.12 | 0.21 | 0.52 | 0.13 | -0.03 | 0.48 | 0.48 | 0.49 | 0.86 | 0.13 | 0.03 |
| Cont.0.40Load.125Cross.8Var.500Size.2Fac.0.70Fcor | 3.94 | 3.94 | 4.87 | 6.11 | 0.16 | 0.16 | 0.09 | 0.28 | 0.36 | -0.67 | 0.62 | 0.62 | 0.49 | 0.76 | 0.36 | 0.67 |
| Cont.0.40Load.125Cross.8Var.1000Size.2Fac.0.00Fcor | 0.16 | 0.16 | 2.40 | 3.49 | 0.06 | 0.06 | 0.64 | 0.89 | 0.19 | 0.00 | 0.06 | 0.06 | 0.64 | 0.91 | 0.19 | 0.00 |
| Cont.0.40Load.125Cross.8Var.1000Size.2Fac.0.50Fcor | 0.91 | 0.91 | 2.94 | 4.19 | 0.08 | 0.08 | 0.22 | 0.39 | 0.02 | 0.00 | 0.12 | 0.12 | 0.30 | 0.57 | 0.02 | 0.00 |
| Cont.0.40Load.125Cross.8Var.1000Size.2Fac.0.70Fcor | 3.28 | 3.28 | 6.53 | 6.72 | -0.12 | -0.12 | -0.25 | -0.26 | 0.05 | -0.36 | 0.50 | 0.50 | 0.71 | 0.72 | 0.07 | 0.36 |
| Cont.0.40Load.125Cross.8Var.300Size.4Fac.0.00Fcor | 31.01 | 31.01 | 30.97 | 31.16 | -3.77 | -3.77 | -3.78 | -3.79 | -3.16 | 0.02 | 3.79 | 3.79 | 3.80 | 3.79 | 3.32 | 0.02 |
| Cont.0.40Load.125Cross.8Var.300Size.4Fac.0.50Fcor | 27.24 | 27.24 | 27.60 | 28.00 | -3.09 | -3.09 | -3.28 | -3.15 | -1.74 | -0.92 | 3.17 | 3.17 | 3.34 | 3.23 | 2.44 | 0.92 |
| Cont.0.40Load.125Cross.8Var.300Size.4Fac.0.70Fcor | 21.52 | 21.52 | 21.85 | 21.91 | -2.14 | -2.14 | -2.43 | -2.09 | -0.69 | -2.82 | 2.54 | 2.54 | 2.61 | 2.53 | 1.63 | 2.82 |
| Cont.0.40Load.125Cross.8Var.500Size.4Fac.0.00Fcor | 4.82 | 4.84 | 10.67 | 7.45 | 0.18 | 0.17 | -0.79 | 0.87 | -0.14 | 0.00 | 1.02 | 1.01 | 1.45 | 1.65 | 0.30 | 0.00 |
| Cont.0.40Load.125Cross.8Var.500Size.4Fac.0.50Fcor | 9.64 | 9.64 | 6.57 | 9.90 | 0.00 | 0.00 | -0.63 | 0.72 | 0.11 | -0.26 | 1.14 | 1.14 | 1.19 | 1.24 | 0.49 | 0.26 |
| Cont.0.40Load.125Cross.8Var.500Size.4Fac.0.70Fcor | 11.68 | 11.68 | 10.30 | 11.96 | -0.41 | -0.41 | -0.96 | 0.01 | -0.45 | -2.57 | 1.49 | 1.49 | 1.64 | 1.41 | 0.83 | 2.57 |
| Cont.0.40Load.125Cross.8Var.1000Size.4Fac.0.00Fcor | 0.55 | 0.55 | 6.78 | 3.85 | 0.15 | 0.15 | -0.82 | 0.98 | 0.00 | 0.00 | 0.19 | 0.19 | 1.18 | 1.00 | 0.00 | 0.00 |
| Cont.0.40Load.125Cross.8Var.1000Size.4Fac.0.50Fcor | 8.24 | 8.24 | 8.81 | 9.40 | -0.59 | -0.59 | -1.46 | 0.02 | 0.04 | 0.00 | 1.09 | 1.09 | 1.50 | 0.96 | 0.20 | 0.00 |
| Cont.0.40Load.125Cross.8Var.1000Size.4Fac.0.70Fcor | 12.51 | 12.51 | 13.89 | 16.00 | -1.25 | -1.25 | -1.54 | -1.04 | -0.32 | -1.63 | 1.57 | 1.57 | 1.70 | 1.40 | 0.70 | 1.63 |
| Cont.0.40Load.Cross250.4Var.300Size.2Fac.0.00Fcor | 4.92 | 4.97 | 5.68 | 6.09 | -1.15 | -1.16 | -1.33 | -1.39 | -0.48 | -0.04 | 1.25 | 1.26 | 1.39 | 1.49 | 0.74 | 0.04 |
| Cont.0.40Load.Cross250.4Var.300Size.2Fac.0.50Fcor | 1.98 | 1.98 | 4.79 | 5.25 | -0.63 | -0.63 | -1.03 | -1.09 | -0.24 | -0.80 | 0.73 | 0.73 | 1.13 | 1.23 | 0.44 | 0.80 |
| Cont.0.40Load.Cross250.4Var.300Size.2Fac.0.70Fcor | 1.31 | 1.29 | 4.78 | 5.20 | -0.62 | -0.62 | -1.05 | -1.11 | -0.59 | -0.96 | 0.64 | 0.64 | 1.07 | 1.15 | 0.65 | 0.96 |
| Cont.0.40Load.Cross250.4Var.500Size.2Fac.0.00Fcor | 1.97 | 1.96 | 3.28 | 3.68 | -0.37 | -0.37 | -0.60 | -0.67 | 0.08 | 0.00 | 0.51 | 0.51 | 0.76 | 0.81 | 0.24 | 0.00 |
| Cont.0.40Load.Cross250.4Var.500Size.2Fac.0.50Fcor | 1.10 | 1.10 | 4.20 | 4.68 | -0.50 | -0.50 | -0.94 | -1.03 | -0.18 | -0.68 | 0.52 | 0.52 | 0.98 | 1.07 | 0.24 | 0.68 |
| Cont.0.40Load.Cross250.4Var.500Size.2Fac.0.70Fcor | 0.70 | 0.70 | 5.61 | 5.99 | -0.69 | -0.69 | -1.31 | -1.38 | -0.66 | -1.00 | 0.75 | 0.75 | 1.37 | 1.44 | 0.70 | 1.00 |
| Cont.0.40Load.Cross250.4Var.1000Size.2Fac.0.00Fcor | 0.55 | 0.59 | 1.46 | 2.08 | -0.10 | -0.10 | -0.14 | -0.25 | 0.09 | 0.00 | 0.20 | 0.20 | 0.34 | 0.49 | 0.09 | 0.00 |
| Cont.0.40Load.Cross250.4Var.1000Size.2Fac.0.50Fcor | 0.66 | 0.66 | 4.53 | 4.94 | -0.55 | -0.55 | -1.06 | -1.15 | -0.25 | -0.64 | 0.57 | 0.57 | 1.10 | 1.17 | 0.25 | 0.64 |
| Cont.0.40Load.Cross250.4Var.1000Size.2Fac.0.70Fcor | 0.56 | 0.56 | 6.59 | 6.74 | -0.84 | -0.84 | -1.60 | -1.64 | -0.84 | -0.99 | 0.84 | 0.84 | 1.60 | 1.64 | 0.84 | 0.99 |
| Cont.0.40Load.Cross250.4Var.300Size.4Fac.0.00Fcor | 14.16 | 14.16 | 14.38 | 14.36 | -3.44 | -3.44 | -3.50 | -3.50 | -2.27 | -0.18 | 3.54 | 3.54 | 3.60 | 3.58 | 2.41 | 0.20 |
| Cont.0.40Load.Cross250.4Var.300Size.4Fac.0.50Fcor | 7.70 | 7.77 | 8.65 | 9.24 | -2.23 | -2.22 | -2.53 | -2.27 | -1.31 | -2.50 | 2.29 | 2.28 | 2.53 | 2.33 | 1.33 | 2.50 |
| Cont.0.40Load.Cross250.4Var.300Size.4Fac.0.70Fcor | 6.30 | 6.30 | 8.41 | 9.09 | -2.15 | -2.15 | -2.40 | -2.31 | -1.48 | -2.95 | 2.19 | 2.19 | 2.42 | 2.35 | 1.54 | 2.95 |
| Cont.0.40Load.Cross250.4Var.500Size.4Fac.0.00Fcor | 6.72 | 6.72 | 7.49 | 7.33 | -1.51 | -1.51 | -1.85 | -1.55 | -0.55 | -0.08 | 1.77 | 1.77 | 2.03 | 1.83 | 0.69 | 0.08 |
| Cont.0.40Load.Cross250.4Var.500Size.4Fac.0.50Fcor | 4.61 | 4.61 | 4.98 | 6.91 | -1.84 | -1.84 | -1.97 | -1.83 | -0.83 | -2.20 | 1.86 | 1.86 | 1.99 | 1.87 | 0.89 | 2.20 |
| Cont.0.40Load.Cross250.4Var.500Size.4Fac.0.70Fcor | 5.04 | 5.04 | 7.92 | 9.00 | -2.30 | -2.30 | -2.45 | -2.42 | -1.36 | -2.97 | 2.32 | 2.32 | 2.45 | 2.44 | 1.38 | 2.97 |
| Cont.0.40Load.Cross250.4Var.1000Size.4Fac.0.00Fcor | 1.42 | 1.44 | 2.59 | 1.89 | -0.17 | -0.18 | -0.68 | -0.14 | -0.03 | -0.01 | 0.45 | 0.44 | 0.96 | 0.52 | 0.09 | 0.01 |
| Cont.0.40Load.Cross250.4Var.1000Size.4Fac.0.50Fcor | 3.90 | 3.90 | 5.37 | 6.43 | -1.71 | -1.71 | -1.85 | -1.69 | -0.93 | -1.61 | 1.73 | 1.73 | 1.87 | 1.71 | 1.03 | 1.61 |
| Cont.0.40Load.Cross250.4Var.1000Size.4Fac.0.70Fcor | 2.99 | 2.99 | 10.26 | 10.98 | -2.54 | -2.54 | -2.85 | -2.89 | -1.52 | -2.97 | 2.54 | 2.54 | 2.85 | 2.89 | 1.54 | 2.97 |
| Cont.0.40Load.Cross250.8Var.300Size.2Fac.0.00Fcor | 10.09 | 10.10 | 10.45 | 10.82 | -0.96 | -0.95 | -0.77 | -0.63 | -0.44 | 0.00 | 1.26 | 1.27 | 1.51 | 1.65 | 0.94 | 0.00 |
| Cont.0.40Load.Cross250.8Var.300Size.2Fac.0.50Fcor | 7.23 | 7.24 | 7.48 | 8.52 | -0.15 | -0.15 | -0.24 | 0.02 | -0.04 | -0.19 | 1.05 | 1.05 | 1.08 | 1.34 | 0.58 | 0.19 |
| Cont.0.40Load.Cross250.8Var.300Size.2Fac.0.70Fcor | 5.57 | 5.57 | 5.61 | 7.14 | 0.17 | 0.17 | 0.03 | 0.34 | 0.27 | -0.83 | 0.85 | 0.85 | 0.83 | 1.14 | 0.51 | 0.83 |
| Cont.0.40Load.Cross250.8Var.500Size.2Fac.0.00Fcor | 1.62 | 1.62 | 3.41 | 4.40 | 0.08 | 0.10 | 0.41 | 0.88 | 0.10 | 0.00 | 0.32 | 0.34 | 0.65 | 1.12 | 0.32 | 0.00 |
| Cont.0.40Load.Cross250.8Var.500Size.2Fac.0.50Fcor | 2.82 | 2.92 | 3.90 | 5.43 | 0.12 | 0.12 | 0.21 | 0.52 | 0.13 | -0.03 | 0.48 | 0.48 | 0.49 | 0.86 | 0.13 | 0.03 |
| Cont.0.40Load.Cross250.8Var.500Size.2Fac.0.70Fcor | 3.94 | 3.94 | 4.87 | 6.11 | 0.16 | 0.16 | 0.09 | 0.28 | 0.36 | -0.67 | 0.62 | 0.62 | 0.49 | 0.76 | 0.36 | 0.67 |
| Cont.0.40Load.Cross250.8Var.1000Size.2Fac.0.00Fcor | 0.16 | 0.16 | 2.40 | 3.49 | 0.06 | 0.06 | 0.64 | 0.89 | 0.19 | 0.00 | 0.06 | 0.06 | 0.64 | 0.91 | 0.19 | 0.00 |
| Cont.0.40Load.Cross250.8Var.1000Size.2Fac.0.50Fcor | 0.91 | 0.91 | 2.94 | 4.19 | 0.08 | 0.08 | 0.22 | 0.39 | 0.02 | 0.00 | 0.12 | 0.12 | 0.30 | 0.57 | 0.02 | 0.00 |
| Cont.0.40Load.Cross250.8Var.1000Size.2Fac.0.70Fcor | 3.28 | 3.28 | 6.53 | 6.72 | -0.12 | -0.12 | -0.25 | -0.26 | 0.05 | -0.36 | 0.50 | 0.50 | 0.71 | 0.72 | 0.07 | 0.36 |
| Cont.0.40Load.Cross250.8Var.300Size.4Fac.0.00Fcor | 31.52 | 31.52 | 31.58 | 31.58 | -3.87 | -3.87 | -3.89 | -3.89 | -3.07 | 0.01 | 3.93 | 3.93 | 3.95 | 3.95 | 3.35 | 0.01 |
| Cont.0.40Load.Cross250.8Var.300Size.4Fac.0.50Fcor | 21.63 | 21.63 | 21.14 | 21.81 | -1.79 | -1.79 | -2.26 | -1.86 | -0.67 | -1.14 | 2.35 | 2.35 | 2.42 | 2.42 | 1.63 | 1.14 |
| Cont.0.40Load.Cross250.8Var.300Size.4Fac.0.70Fcor | 15.50 | 15.43 | 15.25 | 16.11 | -0.99 | -1.00 | -1.44 | -0.97 | -0.05 | -2.84 | 1.65 | 1.66 | 1.84 | 1.71 | 1.03 | 2.84 |
| Cont.0.40Load.Cross250.8Var.500Size.4Fac.0.00Fcor | 12.61 | 12.67 | 14.33 | 14.22 | -0.58 | -0.62 | -1.26 | -0.23 | -0.60 | 0.00 | 2.06 | 2.04 | 2.10 | 2.41 | 1.08 | 0.00 |
| Cont.0.40Load.Cross250.8Var.500Size.4Fac.0.50Fcor | 10.60 | 10.60 | 8.41 | 10.69 | -0.14 | -0.14 | -0.69 | 0.36 | 0.02 | -0.44 | 1.34 | 1.34 | 1.31 | 1.24 | 0.58 | 0.44 |
| Cont.0.40Load.Cross250.8Var.500Size.4Fac.0.70Fcor | 12.79 | 12.63 | 11.47 | 13.20 | -0.54 | -0.54 | -1.20 | -0.37 | -0.37 | -2.84 | 1.60 | 1.60 | 1.44 | 1.49 | 0.97 | 2.84 |
| Cont.0.40Load.Cross250.8Var.1000Size.4Fac.0.00Fcor | 0.75 | 0.84 | 3.57 | 4.92 | 0.15 | 0.15 | -1.00 | 1.16 | 0.08 | 0.00 | 0.25 | 0.25 | 1.44 | 1.20 | 0.08 | 0.00 |
| Cont.0.40Load.Cross250.8Var.1000Size.4Fac.0.50Fcor | 9.62 | 9.62 | 9.91 | 11.10 | -0.80 | -0.80 | -1.16 | -0.31 | 0.18 | -0.03 | 1.20 | 1.20 | 1.34 | 1.01 | 0.56 | 0.03 |
| Cont.0.40Load.Cross250.8Var.1000Size.4Fac.0.70Fcor | 11.53 | 11.53 | 14.70 | 16.17 | -1.42 | -1.42 | -1.64 | -1.23 | -0.27 | -2.15 | 1.74 | 1.74 | 1.76 | 1.61 | 0.77 | 2.15 |
| Cont.0.55Load.000Cross.4Var.300Size.2Fac.0.00Fcor | 0.01 | 0.01 | 0.38 | 0.86 | 0.00 | 0.00 | 0.09 | 0.01 | 0.00 | 0.00 | 0.00 | 0.00 | 0.09 | 0.19 | 0.00 | 0.00 |
| Cont.0.55Load.000Cross.4Var.300Size.2Fac.0.50Fcor | 0.07 | 0.07 | 0.18 | 0.86 | 0.05 | 0.05 | 0.11 | 0.08 | 0.00 | -0.08 | 0.05 | 0.05 | 0.11 | 0.28 | 0.00 | 0.08 |
| Cont.0.55Load.000Cross.4Var.300Size.2Fac.0.70Fcor | 0.50 | 0.50 | 0.70 | 1.70 | -0.02 | -0.02 | -0.02 | -0.13 | -0.20 | -0.90 | 0.16 | 0.16 | 0.22 | 0.41 | 0.20 | 0.90 |
| Cont.0.55Load.000Cross.4Var.500Size.2Fac.0.00Fcor | 0.00 | 0.00 | 0.29 | 0.55 | 0.00 | 0.00 | 0.02 | 0.01 | 0.00 | 0.00 | 0.00 | 0.00 | 0.02 | 0.09 | 0.00 | 0.00 |
| Cont.0.55Load.000Cross.4Var.500Size.2Fac.0.50Fcor | 0.03 | 0.03 | 0.42 | 0.78 | 0.00 | 0.00 | 0.04 | 0.09 | 0.00 | -0.05 | 0.02 | 0.02 | 0.08 | 0.21 | 0.00 | 0.05 |
| Cont.0.55Load.000Cross.4Var.500Size.2Fac.0.70Fcor | 0.26 | 0.26 | 0.45 | 1.14 | -0.01 | -0.01 | 0.04 | -0.07 | -0.32 | -0.89 | 0.07 | 0.07 | 0.12 | 0.29 | 0.32 | 0.89 |
| Cont.0.55Load.000Cross.4Var.1000Size.2Fac.0.00Fcor | 0.00 | 0.00 | 0.29 | 0.47 | 0.00 | 0.00 | 0.05 | 0.03 | 0.00 | 0.00 | 0.00 | 0.00 | 0.05 | 0.11 | 0.00 | 0.00 |
| Cont.0.55Load.000Cross.4Var.1000Size.2Fac.0.50Fcor | 0.00 | 0.00 | 0.25 | 0.64 | 0.00 | 0.00 | 0.07 | 0.00 | 0.00 | 0.00 | 0.00 | 0.00 | 0.07 | 0.14 | 0.00 | 0.00 |
| Cont.0.55Load.000Cross.4Var.1000Size.2Fac.0.70Fcor | 0.36 | 0.36 | 0.48 | 1.07 | -0.02 | -0.02 | -0.03 | -0.12 | -0.21 | -0.75 | 0.08 | 0.08 | 0.09 | 0.24 | 0.21 | 0.75 |
| Cont.0.55Load.000Cross.4Var.300Size.4Fac.0.00Fcor | 0.01 | 0.01 | 3.29 | 0.97 | 0.02 | 0.02 | -0.60 | 0.12 | 0.00 | 0.00 | 0.02 | 0.02 | 0.68 | 0.24 | 0.00 | 0.00 |
| Cont.0.55Load.000Cross.4Var.300Size.4Fac.0.50Fcor | 1.86 | 1.86 | 1.94 | 2.40 | -0.30 | -0.30 | -1.33 | -0.21 | -0.06 | -1.03 | 0.48 | 0.48 | 1.35 | 0.49 | 0.14 | 1.03 |
| Cont.0.55Load.000Cross.4Var.300Size.4Fac.0.70Fcor | 3.13 | 3.13 | 1.89 | 3.46 | -0.68 | -0.68 | -1.31 | -0.45 | -0.91 | -2.92 | 0.98 | 0.98 | 1.43 | 0.89 | 0.95 | 2.92 |
| Cont.0.55Load.000Cross.4Var.500Size.4Fac.0.00Fcor | 0.00 | 0.00 | 2.60 | 0.85 | 0.00 | 0.00 | -0.50 | -0.02 | 0.00 | 0.00 | 0.00 | 0.00 | 0.54 | 0.14 | 0.00 | 0.00 |
| Cont.0.55Load.000Cross.4Var.500Size.4Fac.0.50Fcor | 0.77 | 0.77 | 0.79 | 1.52 | -0.19 | -0.19 | -1.28 | -0.08 | 0.01 | -0.39 | 0.25 | 0.25 | 1.32 | 0.40 | 0.03 | 0.39 |
| Cont.0.55Load.000Cross.4Var.500Size.4Fac.0.70Fcor | 2.32 | 2.32 | 1.19 | 2.99 | -0.68 | -0.68 | -1.32 | -0.50 | -0.44 | -2.81 | 0.84 | 0.84 | 1.38 | 0.72 | 0.50 | 2.81 |
| Cont.0.55Load.000Cross.4Var.1000Size.4Fac.0.00Fcor | 0.00 | 0.00 | 1.86 | 0.64 | 0.00 | 0.00 | -0.35 | 0.06 | 0.00 | 0.00 | 0.00 | 0.00 | 0.55 | 0.16 | 0.00 | 0.00 |
| Cont.0.55Load.000Cross.4Var.1000Size.4Fac.0.50Fcor | 0.11 | 0.11 | 0.81 | 0.77 | -0.03 | -0.03 | -1.32 | 0.04 | 0.00 | -0.03 | 0.03 | 0.03 | 1.40 | 0.14 | 0.00 | 0.03 |
| Cont.0.55Load.000Cross.4Var.1000Size.4Fac.0.70Fcor | 1.69 | 1.69 | 0.94 | 2.47 | -0.63 | -0.63 | -1.31 | -0.57 | -0.17 | -2.78 | 0.65 | 0.65 | 1.31 | 0.61 | 0.17 | 2.78 |
| Cont.0.55Load.000Cross.8Var.300Size.2Fac.0.00Fcor | 0.00 | 0.00 | 2.70 | 3.31 | 0.00 | 0.00 | 0.83 | 1.08 | 0.00 | 0.00 | 0.00 | 0.00 | 0.83 | 1.08 | 0.00 | 0.00 |
| Cont.0.55Load.000Cross.8Var.300Size.2Fac.0.50Fcor | 0.09 | 0.09 | 2.42 | 3.30 | 0.06 | 0.06 | 0.62 | 0.98 | 0.00 | 0.00 | 0.06 | 0.06 | 0.62 | 0.98 | 0.00 | 0.00 |
| Cont.0.55Load.000Cross.8Var.300Size.2Fac.0.70Fcor | 0.74 | 0.74 | 2.04 | 2.90 | 0.43 | 0.43 | 0.67 | 1.14 | 0.00 | -0.15 | 0.45 | 0.45 | 0.67 | 1.14 | 0.00 | 0.15 |
| Cont.0.55Load.000Cross.8Var.500Size.2Fac.0.00Fcor | 0.00 | 0.00 | 2.70 | 3.41 | 0.00 | 0.00 | 0.57 | 0.82 | 0.00 | 0.00 | 0.00 | 0.00 | 0.57 | 0.82 | 0.00 | 0.00 |
| Cont.0.55Load.000Cross.8Var.500Size.2Fac.0.50Fcor | 0.01 | 0.01 | 1.90 | 2.85 | 0.01 | 0.01 | 0.41 | 0.93 | 0.00 | 0.00 | 0.01 | 0.01 | 0.41 | 0.93 | 0.00 | 0.00 |
| Cont.0.55Load.000Cross.8Var.500Size.2Fac.0.70Fcor | 0.22 | 0.22 | 1.52 | 3.12 | 0.09 | 0.09 | 0.42 | 0.85 | 0.00 | -0.02 | 0.09 | 0.09 | 0.42 | 0.85 | 0.00 | 0.02 |
| Cont.0.55Load.000Cross.8Var.1000Size.2Fac.0.00Fcor | 0.00 | 0.00 | 1.84 | 3.12 | 0.00 | 0.00 | 0.59 | 0.80 | 0.00 | 0.00 | 0.00 | 0.00 | 0.59 | 0.80 | 0.00 | 0.00 |
| Cont.0.55Load.000Cross.8Var.1000Size.2Fac.0.50Fcor | 0.02 | 0.02 | 1.81 | 2.64 | 0.00 | 0.00 | 0.53 | 0.92 | 0.00 | 0.00 | 0.00 | 0.00 | 0.53 | 0.92 | 0.00 | 0.00 |
| Cont.0.55Load.000Cross.8Var.1000Size.2Fac.0.70Fcor | 0.28 | 0.28 | 2.28 | 3.42 | 0.22 | 0.22 | 0.48 | 0.73 | 0.00 | 0.00 | 0.22 | 0.22 | 0.48 | 0.73 | 0.00 | 0.00 |
| Cont.0.55Load.000Cross.8Var.300Size.4Fac.0.00Fcor | 0.00 | 0.00 | 10.02 | 3.48 | 0.00 | 0.00 | -0.19 | 1.62 | 0.00 | 0.00 | 0.00 | 0.00 | 1.19 | 1.62 | 0.00 | 0.00 |
| Cont.0.55Load.000Cross.8Var.300Size.4Fac.0.50Fcor | 1.62 | 1.62 | 1.94 | 4.94 | 0.31 | 0.31 | -0.88 | 1.74 | 0.00 | 0.00 | 0.41 | 0.41 | 1.28 | 1.78 | 0.00 | 0.00 |
| Cont.0.55Load.000Cross.8Var.300Size.4Fac.0.70Fcor | 3.52 | 3.52 | 2.19 | 4.78 | 1.32 | 1.32 | -0.38 | 2.50 | 0.04 | -1.58 | 1.56 | 1.56 | 1.30 | 2.56 | 0.08 | 1.58 |
| Cont.0.55Load.000Cross.8Var.500Size.4Fac.0.00Fcor | 0.00 | 0.00 | 7.78 | 3.05 | 0.00 | 0.00 | 0.00 | 1.37 | 0.00 | 0.00 | 0.00 | 0.00 | 1.06 | 1.37 | 0.00 | 0.00 |
| Cont.0.55Load.000Cross.8Var.500Size.4Fac.0.50Fcor | 0.67 | 0.67 | 2.58 | 4.44 | 0.06 | 0.06 | -0.95 | 1.05 | 0.00 | 0.00 | 0.08 | 0.08 | 1.31 | 1.09 | 0.00 | 0.00 |
| Cont.0.55Load.000Cross.8Var.500Size.4Fac.0.70Fcor | 2.48 | 2.41 | 2.64 | 4.54 | 0.33 | 0.35 | -0.69 | 1.44 | 0.00 | -0.75 | 0.65 | 0.65 | 1.37 | 1.58 | 0.02 | 0.75 |
| Cont.0.55Load.000Cross.8Var.1000Size.4Fac.0.00Fcor | 0.00 | 0.00 | 5.83 | 3.87 | 0.00 | 0.00 | -0.08 | 1.17 | 0.00 | 0.00 | 0.00 | 0.00 | 1.08 | 1.17 | 0.00 | 0.00 |
| Cont.0.55Load.000Cross.8Var.1000Size.4Fac.0.50Fcor | 0.01 | 0.01 | 1.89 | 4.24 | 0.03 | 0.03 | -1.10 | 1.22 | 0.00 | 0.00 | 0.03 | 0.03 | 1.30 | 1.22 | 0.00 | 0.00 |
| Cont.0.55Load.000Cross.8Var.1000Size.4Fac.0.70Fcor | 0.58 | 0.58 | 3.85 | 5.74 | 0.07 | 0.07 | -0.92 | 0.87 | 0.00 | -0.02 | 0.15 | 0.15 | 1.20 | 1.11 | 0.00 | 0.02 |
| Cont.0.55Load.125Cross.4Var.300Size.2Fac.0.00Fcor | 0.07 | 0.07 | 0.29 | 1.48 | -0.01 | -0.01 | 0.04 | -0.07 | 0.00 | 0.00 | 0.01 | 0.01 | 0.08 | 0.35 | 0.00 | 0.00 |
| Cont.0.55Load.125Cross.4Var.300Size.2Fac.0.50Fcor | 0.33 | 0.33 | 0.72 | 1.69 | -0.02 | -0.02 | -0.04 | -0.15 | -0.06 | -0.33 | 0.10 | 0.10 | 0.16 | 0.41 | 0.06 | 0.33 |
| Cont.0.55Load.125Cross.4Var.300Size.2Fac.0.70Fcor | 0.88 | 0.88 | 2.70 | 3.33 | -0.25 | -0.25 | -0.48 | -0.58 | -0.64 | -0.96 | 0.41 | 0.41 | 0.68 | 0.82 | 0.64 | 0.96 |
| Cont.0.55Load.125Cross.4Var.500Size.2Fac.0.00Fcor | 0.00 | 0.00 | 0.19 | 0.94 | 0.00 | 0.00 | 0.04 | 0.03 | 0.00 | 0.00 | 0.00 | 0.00 | 0.04 | 0.19 | 0.00 | 0.00 |
| Cont.0.55Load.125Cross.4Var.500Size.2Fac.0.50Fcor | 0.16 | 0.16 | 0.61 | 1.77 | -0.03 | -0.03 | -0.05 | -0.22 | -0.03 | -0.18 | 0.05 | 0.05 | 0.11 | 0.36 | 0.03 | 0.18 |
| Cont.0.55Load.125Cross.4Var.500Size.2Fac.0.70Fcor | 0.58 | 0.58 | 1.88 | 2.86 | -0.19 | -0.19 | -0.34 | -0.49 | -0.69 | -0.98 | 0.25 | 0.25 | 0.42 | 0.63 | 0.69 | 0.98 |
| Cont.0.55Load.125Cross.4Var.1000Size.2Fac.0.00Fcor | 0.00 | 0.00 | 0.23 | 0.83 | 0.00 | 0.00 | 0.00 | 0.00 | 0.00 | 0.00 | 0.00 | 0.00 | 0.00 | 0.06 | 0.00 | 0.00 |
| Cont.0.55Load.125Cross.4Var.1000Size.2Fac.0.50Fcor | 0.24 | 0.24 | 0.64 | 1.32 | -0.01 | -0.01 | -0.03 | -0.10 | 0.00 | -0.04 | 0.05 | 0.05 | 0.09 | 0.24 | 0.00 | 0.04 |
| Cont.0.55Load.125Cross.4Var.1000Size.2Fac.0.70Fcor | 0.75 | 0.75 | 2.03 | 2.77 | -0.23 | -0.23 | -0.37 | -0.51 | -0.78 | -0.95 | 0.25 | 0.25 | 0.39 | 0.53 | 0.78 | 0.95 |
| Cont.0.55Load.125Cross.4Var.300Size.4Fac.0.00Fcor | 0.17 | 0.15 | 2.49 | 1.46 | 0.00 | 0.03 | -0.61 | 0.03 | -0.01 | 0.00 | 0.06 | 0.05 | 0.81 | 0.37 | 0.01 | 0.00 |
| Cont.0.55Load.125Cross.4Var.300Size.4Fac.0.50Fcor | 1.94 | 1.94 | 1.62 | 3.04 | -0.63 | -0.63 | -1.47 | -0.49 | -0.39 | -1.42 | 0.75 | 0.75 | 1.47 | 0.65 | 0.47 | 1.42 |
| Cont.0.55Load.125Cross.4Var.300Size.4Fac.0.70Fcor | 3.64 | 3.66 | 3.19 | 4.78 | -1.38 | -1.38 | -1.72 | -1.27 | -0.96 | -2.95 | 1.46 | 1.46 | 1.76 | 1.37 | 1.04 | 2.95 |
| Cont.0.55Load.125Cross.4Var.500Size.4Fac.0.00Fcor | 0.00 | 0.00 | 1.69 | 1.01 | 0.01 | 0.01 | -0.72 | 0.10 | -0.01 | 0.00 | 0.01 | 0.01 | 0.82 | 0.20 | 0.01 | 0.00 |
| Cont.0.55Load.125Cross.4Var.500Size.4Fac.0.50Fcor | 0.87 | 0.87 | 0.84 | 1.98 | -0.31 | -0.31 | -1.39 | -0.19 | -0.31 | -0.89 | 0.45 | 0.45 | 1.41 | 0.47 | 0.31 | 0.89 |
| Cont.0.55Load.125Cross.4Var.500Size.4Fac.0.70Fcor | 3.03 | 3.03 | 1.78 | 4.20 | -1.34 | -1.34 | -1.69 | -1.21 | -0.85 | -2.94 | 1.38 | 1.38 | 1.69 | 1.25 | 0.89 | 2.94 |
| Cont.0.55Load.125Cross.4Var.1000Size.4Fac.0.00Fcor | 0.00 | 0.00 | 1.15 | 1.10 | 0.00 | 0.00 | -0.98 | 0.02 | -0.02 | 0.00 | 0.00 | 0.00 | 1.02 | 0.08 | 0.02 | 0.00 |
| Cont.0.55Load.125Cross.4Var.1000Size.4Fac.0.50Fcor | 0.41 | 0.41 | 0.37 | 1.63 | -0.28 | -0.28 | -1.69 | -0.24 | -0.23 | -0.30 | 0.38 | 0.38 | 1.71 | 0.42 | 0.23 | 0.30 |
| Cont.0.55Load.125Cross.4Var.1000Size.4Fac.0.70Fcor | 2.62 | 2.62 | 2.05 | 3.99 | -1.21 | -1.21 | -1.59 | -1.17 | -0.60 | -2.80 | 1.27 | 1.27 | 1.65 | 1.27 | 0.66 | 2.80 |
| Cont.0.55Load.125Cross.8Var.300Size.2Fac.0.00Fcor | 0.12 | 0.14 | 2.21 | 3.00 | 0.01 | 0.02 | 0.64 | 1.17 | 0.22 | 0.00 | 0.01 | 0.02 | 0.64 | 1.17 | 0.22 | 0.00 |
| Cont.0.55Load.125Cross.8Var.300Size.2Fac.0.50Fcor | 0.48 | 0.48 | 2.38 | 3.36 | 0.13 | 0.13 | 0.66 | 1.13 | 0.05 | 0.00 | 0.17 | 0.17 | 0.66 | 1.13 | 0.05 | 0.00 |
| Cont.0.55Load.125Cross.8Var.300Size.2Fac.0.70Fcor | 1.82 | 1.82 | 1.99 | 4.31 | 0.14 | 0.14 | 0.45 | 0.73 | 0.04 | -0.44 | 0.28 | 0.28 | 0.49 | 0.81 | 0.04 | 0.44 |
| Cont.0.55Load.125Cross.8Var.500Size.2Fac.0.00Fcor | 0.08 | 0.11 | 2.22 | 3.69 | 0.01 | 0.01 | 0.39 | 0.82 | 0.34 | 0.00 | 0.01 | 0.01 | 0.39 | 0.84 | 0.34 | 0.00 |
| Cont.0.55Load.125Cross.8Var.500Size.2Fac.0.50Fcor | 0.05 | 0.05 | 1.89 | 3.06 | 0.04 | 0.04 | 0.69 | 1.10 | 0.01 | 0.00 | 0.04 | 0.04 | 0.69 | 1.12 | 0.01 | 0.00 |
| Cont.0.55Load.125Cross.8Var.500Size.2Fac.0.70Fcor | 0.68 | 0.68 | 2.38 | 3.49 | 0.21 | 0.21 | 0.45 | 0.66 | 0.00 | -0.17 | 0.23 | 0.23 | 0.45 | 0.70 | 0.00 | 0.17 |
| Cont.0.55Load.125Cross.8Var.1000Size.2Fac.0.00Fcor | 0.11 | 0.21 | 2.40 | 3.61 | 0.00 | 0.00 | 0.52 | 0.84 | 0.26 | 0.00 | 0.00 | 0.00 | 0.52 | 0.84 | 0.26 | 0.00 |
| Cont.0.55Load.125Cross.8Var.1000Size.2Fac.0.50Fcor | 0.00 | 0.00 | 1.83 | 3.35 | 0.00 | 0.00 | 0.62 | 0.71 | 0.00 | 0.00 | 0.00 | 0.00 | 0.62 | 0.75 | 0.00 | 0.00 |
| Cont.0.55Load.125Cross.8Var.1000Size.2Fac.0.70Fcor | 0.53 | 0.53 | 2.57 | 3.57 | 0.01 | 0.01 | 0.14 | 0.14 | 0.00 | -0.01 | 0.07 | 0.07 | 0.18 | 0.26 | 0.00 | 0.01 |
| Cont.0.55Load.125Cross.8Var.300Size.4Fac.0.00Fcor | 0.04 | 0.05 | 5.98 | 4.29 | 0.03 | 0.03 | -0.56 | 1.63 | 0.00 | 0.00 | 0.03 | 0.03 | 1.18 | 1.63 | 0.00 | 0.00 |
| Cont.0.55Load.125Cross.8Var.300Size.4Fac.0.50Fcor | 2.16 | 2.16 | 2.02 | 4.94 | 0.19 | 0.19 | -0.62 | 2.06 | 0.02 | -0.01 | 0.55 | 0.55 | 1.32 | 2.12 | 0.08 | 0.01 |
| Cont.0.55Load.125Cross.8Var.300Size.4Fac.0.70Fcor | 5.71 | 5.81 | 3.85 | 7.22 | 0.68 | 0.67 | -0.73 | 1.64 | -0.04 | -2.13 | 1.44 | 1.45 | 1.25 | 1.96 | 0.26 | 2.13 |
| Cont.0.55Load.125Cross.8Var.500Size.4Fac.0.00Fcor | 0.00 | 0.00 | 5.88 | 3.79 | 0.00 | 0.00 | -0.61 | 1.01 | 0.00 | 0.00 | 0.00 | 0.00 | 1.15 | 1.01 | 0.00 | 0.00 |
| Cont.0.55Load.125Cross.8Var.500Size.4Fac.0.50Fcor | 0.52 | 0.52 | 2.19 | 4.42 | 0.07 | 0.07 | -1.13 | 1.52 | 0.04 | 0.00 | 0.15 | 0.15 | 1.39 | 1.52 | 0.04 | 0.00 |
| Cont.0.55Load.125Cross.8Var.500Size.4Fac.0.70Fcor | 3.34 | 3.34 | 4.16 | 6.11 | 0.08 | 0.08 | -0.81 | 1.05 | 0.00 | -1.23 | 0.82 | 0.82 | 1.35 | 1.35 | 0.14 | 1.23 |
| Cont.0.55Load.125Cross.8Var.1000Size.4Fac.0.00Fcor | 0.00 | 0.00 | 5.57 | 4.88 | 0.00 | 0.00 | -0.52 | 1.01 | 0.00 | 0.00 | 0.00 | 0.00 | 1.14 | 1.01 | 0.00 | 0.00 |
| Cont.0.55Load.125Cross.8Var.1000Size.4Fac.0.50Fcor | 0.04 | 0.04 | 3.03 | 4.22 | -0.01 | -0.01 | -0.96 | 1.48 | 0.09 | 0.00 | 0.01 | 0.01 | 1.28 | 1.48 | 0.09 | 0.00 |
| Cont.0.55Load.125Cross.8Var.1000Size.4Fac.0.70Fcor | 0.93 | 0.93 | 4.56 | 4.75 | 0.08 | 0.08 | -0.70 | 1.16 | 0.04 | -0.21 | 0.40 | 0.40 | 1.28 | 1.26 | 0.04 | 0.21 |
| Cont.0.55Load.Cross250.4Var.300Size.2Fac.0.00Fcor | 0.36 | 0.39 | 0.57 | 1.53 | 0.00 | 0.02 | 0.06 | 0.04 | 0.19 | 0.00 | 0.10 | 0.12 | 0.18 | 0.36 | 0.19 | 0.00 |
| Cont.0.55Load.Cross250.4Var.300Size.2Fac.0.50Fcor | 0.28 | 0.28 | 0.98 | 2.40 | -0.10 | -0.10 | -0.13 | -0.45 | -0.24 | -0.54 | 0.14 | 0.14 | 0.23 | 0.57 | 0.24 | 0.54 |
| Cont.0.55Load.Cross250.4Var.300Size.2Fac.0.70Fcor | 0.66 | 0.66 | 3.47 | 4.23 | -0.43 | -0.43 | -0.79 | -0.94 | -0.90 | -0.99 | 0.43 | 0.43 | 0.81 | 0.98 | 0.90 | 0.99 |
| Cont.0.55Load.Cross250.4Var.500Size.2Fac.0.00Fcor | 0.13 | 0.15 | 0.47 | 1.35 | 0.05 | 0.05 | 0.18 | 0.10 | 0.10 | 0.00 | 0.07 | 0.07 | 0.18 | 0.30 | 0.10 | 0.00 |
| Cont.0.55Load.Cross250.4Var.500Size.2Fac.0.50Fcor | 0.24 | 0.24 | 0.90 | 1.86 | -0.07 | -0.07 | -0.11 | -0.31 | -0.22 | -0.37 | 0.11 | 0.11 | 0.21 | 0.43 | 0.22 | 0.37 |
| Cont.0.55Load.Cross250.4Var.500Size.2Fac.0.70Fcor | 0.68 | 0.68 | 3.12 | 3.94 | -0.37 | -0.37 | -0.72 | -0.90 | -0.94 | -1.00 | 0.39 | 0.39 | 0.72 | 0.92 | 0.94 | 1.00 |
| Cont.0.55Load.Cross250.4Var.1000Size.2Fac.0.00Fcor | 0.09 | 0.09 | 0.58 | 1.32 | 0.02 | 0.02 | 0.11 | 0.13 | 0.02 | 0.00 | 0.02 | 0.02 | 0.11 | 0.17 | 0.02 | 0.00 |
| Cont.0.55Load.Cross250.4Var.1000Size.2Fac.0.50Fcor | 0.35 | 0.35 | 0.75 | 1.88 | -0.08 | -0.08 | -0.12 | -0.38 | -0.22 | -0.23 | 0.08 | 0.08 | 0.12 | 0.40 | 0.22 | 0.23 |
| Cont.0.55Load.Cross250.4Var.1000Size.2Fac.0.70Fcor | 0.71 | 0.71 | 3.41 | 4.28 | -0.44 | -0.44 | -0.78 | -0.99 | -0.99 | -1.00 | 0.44 | 0.44 | 0.78 | 0.99 | 0.99 | 1.00 |
| Cont.0.55Load.Cross250.4Var.300Size.4Fac.0.00Fcor | 0.56 | 0.56 | 2.68 | 1.34 | -0.03 | -0.03 | -0.92 | 0.14 | -0.01 | -0.01 | 0.21 | 0.21 | 1.04 | 0.36 | 0.01 | 0.01 |
| Cont.0.55Load.Cross250.4Var.300Size.4Fac.0.50Fcor | 2.10 | 2.14 | 1.86 | 3.14 | -0.66 | -0.67 | -1.42 | -0.48 | -0.61 | -1.65 | 0.82 | 0.83 | 1.46 | 0.84 | 0.81 | 1.65 |
| Cont.0.55Load.Cross250.4Var.300Size.4Fac.0.70Fcor | 3.88 | 3.88 | 3.84 | 5.46 | -1.47 | -1.47 | -1.69 | -1.31 | -1.40 | -2.98 | 1.55 | 1.55 | 1.71 | 1.41 | 1.48 | 2.98 |
| Cont.0.55Load.Cross250.4Var.500Size.4Fac.0.00Fcor | 0.02 | 0.02 | 2.01 | 1.18 | 0.01 | 0.01 | -0.89 | 0.10 | -0.02 | 0.00 | 0.01 | 0.01 | 0.93 | 0.10 | 0.02 | 0.00 |
| Cont.0.55Load.Cross250.4Var.500Size.4Fac.0.50Fcor | 1.28 | 1.28 | 0.88 | 2.26 | -0.49 | -0.49 | -1.32 | -0.25 | -0.50 | -1.10 | 0.61 | 0.61 | 1.34 | 0.61 | 0.60 | 1.10 |
| Cont.0.55Load.Cross250.4Var.500Size.4Fac.0.70Fcor | 3.35 | 3.35 | 2.73 | 5.01 | -1.51 | -1.51 | -1.73 | -1.46 | -1.51 | -2.96 | 1.55 | 1.55 | 1.75 | 1.50 | 1.53 | 2.96 |
| Cont.0.55Load.Cross250.4Var.1000Size.4Fac.0.00Fcor | 0.00 | 0.00 | 0.90 | 1.56 | 0.00 | 0.00 | -0.91 | 0.05 | -0.01 | 0.00 | 0.00 | 0.00 | 0.91 | 0.05 | 0.01 | 0.00 |
| Cont.0.55Load.Cross250.4Var.1000Size.4Fac.0.50Fcor | 0.33 | 0.33 | 0.77 | 1.41 | -0.15 | -0.15 | -1.47 | 0.01 | -0.30 | -0.48 | 0.25 | 0.25 | 1.51 | 0.33 | 0.36 | 0.48 |
| Cont.0.55Load.Cross250.4Var.1000Size.4Fac.0.70Fcor | 2.82 | 2.82 | 2.58 | 4.08 | -1.37 | -1.37 | -1.71 | -1.25 | -1.67 | -2.93 | 1.37 | 1.37 | 1.71 | 1.27 | 1.77 | 2.93 |
| Cont.0.55Load.Cross250.8Var.300Size.2Fac.0.00Fcor | 0.12 | 0.14 | 2.21 | 3.00 | 0.01 | 0.02 | 0.64 | 1.17 | 0.22 | 0.00 | 0.01 | 0.02 | 0.64 | 1.17 | 0.22 | 0.00 |
| Cont.0.55Load.Cross250.8Var.300Size.2Fac.0.50Fcor | 0.48 | 0.48 | 2.38 | 3.36 | 0.13 | 0.13 | 0.66 | 1.13 | 0.05 | 0.00 | 0.17 | 0.17 | 0.66 | 1.13 | 0.05 | 0.00 |
| Cont.0.55Load.Cross250.8Var.300Size.2Fac.0.70Fcor | 1.82 | 1.82 | 1.99 | 4.31 | 0.14 | 0.14 | 0.45 | 0.73 | 0.04 | -0.44 | 0.28 | 0.28 | 0.49 | 0.81 | 0.04 | 0.44 |
| Cont.0.55Load.Cross250.8Var.500Size.2Fac.0.00Fcor | 0.08 | 0.11 | 2.22 | 3.69 | 0.01 | 0.01 | 0.39 | 0.82 | 0.34 | 0.00 | 0.01 | 0.01 | 0.39 | 0.84 | 0.34 | 0.00 |
| Cont.0.55Load.Cross250.8Var.500Size.2Fac.0.50Fcor | 0.05 | 0.05 | 1.89 | 3.06 | 0.04 | 0.04 | 0.69 | 1.10 | 0.01 | 0.00 | 0.04 | 0.04 | 0.69 | 1.12 | 0.01 | 0.00 |
| Cont.0.55Load.Cross250.8Var.500Size.2Fac.0.70Fcor | 0.68 | 0.68 | 2.38 | 3.49 | 0.21 | 0.21 | 0.45 | 0.66 | 0.00 | -0.17 | 0.23 | 0.23 | 0.45 | 0.70 | 0.00 | 0.17 |
| Cont.0.55Load.Cross250.8Var.1000Size.2Fac.0.00Fcor | 0.11 | 0.21 | 2.40 | 3.61 | 0.00 | 0.00 | 0.52 | 0.84 | 0.26 | 0.00 | 0.00 | 0.00 | 0.52 | 0.84 | 0.26 | 0.00 |
| Cont.0.55Load.Cross250.8Var.1000Size.2Fac.0.50Fcor | 0.00 | 0.00 | 1.83 | 3.35 | 0.00 | 0.00 | 0.62 | 0.71 | 0.00 | 0.00 | 0.00 | 0.00 | 0.62 | 0.75 | 0.00 | 0.00 |
| Cont.0.55Load.Cross250.8Var.1000Size.2Fac.0.70Fcor | 0.53 | 0.53 | 2.57 | 3.57 | 0.01 | 0.01 | 0.14 | 0.14 | 0.00 | -0.01 | 0.07 | 0.07 | 0.18 | 0.26 | 0.00 | 0.01 |
| Cont.0.55Load.Cross250.8Var.300Size.4Fac.0.00Fcor | 0.23 | 0.28 | 3.55 | 4.82 | 0.27 | 0.29 | -1.14 | 1.83 | 0.12 | 0.00 | 0.29 | 0.29 | 1.40 | 1.83 | 0.12 | 0.00 |
| Cont.0.55Load.Cross250.8Var.300Size.4Fac.0.50Fcor | 2.67 | 2.67 | 3.25 | 5.59 | 0.61 | 0.61 | -0.33 | 2.29 | 0.21 | -0.07 | 0.95 | 0.95 | 1.29 | 2.29 | 0.33 | 0.07 |
| Cont.0.55Load.Cross250.8Var.300Size.4Fac.0.70Fcor | 7.29 | 7.29 | 6.21 | 8.25 | 0.73 | 0.73 | -0.35 | 1.59 | -0.16 | -2.35 | 1.41 | 1.41 | 1.53 | 1.81 | 0.46 | 2.35 |
| Cont.0.55Load.Cross250.8Var.500Size.4Fac.0.00Fcor | 0.07 | 0.07 | 1.81 | 4.31 | 0.01 | 0.01 | -1.21 | 1.26 | 0.18 | 0.00 | 0.01 | 0.01 | 1.45 | 1.26 | 0.18 | 0.00 |
| Cont.0.55Load.Cross250.8Var.500Size.4Fac.0.50Fcor | 0.95 | 0.95 | 2.85 | 4.32 | 0.15 | 0.15 | -0.93 | 2.02 | 0.32 | 0.00 | 0.35 | 0.35 | 1.61 | 2.04 | 0.34 | 0.00 |
| Cont.0.55Load.Cross250.8Var.500Size.4Fac.0.70Fcor | 5.11 | 5.11 | 3.69 | 6.01 | -0.12 | -0.12 | -0.94 | 1.17 | 0.10 | -1.75 | 0.88 | 0.88 | 1.30 | 1.41 | 0.32 | 1.75 |
| Cont.0.55Load.Cross250.8Var.1000Size.4Fac.0.00Fcor | 0.03 | 0.04 | 1.10 | 4.91 | 0.00 | 0.00 | -1.48 | 0.85 | 0.13 | 0.00 | 0.00 | 0.00 | 1.52 | 0.87 | 0.13 | 0.00 |
| Cont.0.55Load.Cross250.8Var.1000Size.4Fac.0.50Fcor | 0.09 | 0.09 | 2.46 | 3.06 | 0.02 | 0.02 | -0.63 | 1.37 | 0.31 | 0.00 | 0.04 | 0.04 | 1.35 | 1.37 | 0.39 | 0.00 |
| Cont.0.55Load.Cross250.8Var.1000Size.4Fac.0.70Fcor | 1.78 | 1.78 | 6.86 | 5.11 | -0.17 | -0.17 | -0.81 | 0.66 | 0.17 | -0.50 | 0.37 | 0.37 | 1.19 | 0.92 | 0.31 | 0.50 |
| Cont.0.70Load.000Cross.4Var.300Size.2Fac.0.00Fcor | 0.00 | 0.00 | 0.36 | 0.64 | 0.00 | 0.00 | 0.08 | 0.05 | 0.00 | 0.00 | 0.00 | 0.00 | 0.08 | 0.13 | 0.00 | 0.00 |
| Cont.0.70Load.000Cross.4Var.300Size.2Fac.0.50Fcor | 0.00 | 0.00 | 0.15 | 0.57 | 0.00 | 0.00 | 0.06 | 0.07 | 0.00 | -0.01 | 0.00 | 0.00 | 0.06 | 0.13 | 0.00 | 0.01 |
| Cont.0.70Load.000Cross.4Var.300Size.2Fac.0.70Fcor | 0.00 | 0.00 | 0.24 | 0.76 | 0.00 | 0.00 | 0.03 | 0.03 | -0.14 | -0.76 | 0.00 | 0.00 | 0.03 | 0.19 | 0.14 | 0.76 |
| Cont.0.70Load.000Cross.4Var.500Size.2Fac.0.00Fcor | 0.00 | 0.00 | 0.29 | 0.53 | 0.00 | 0.00 | 0.01 | -0.01 | 0.00 | 0.00 | 0.00 | 0.00 | 0.01 | 0.05 | 0.00 | 0.00 |
| Cont.0.70Load.000Cross.4Var.500Size.2Fac.0.50Fcor | 0.00 | 0.00 | 0.21 | 0.63 | 0.00 | 0.00 | 0.09 | 0.07 | 0.00 | 0.00 | 0.00 | 0.00 | 0.09 | 0.17 | 0.00 | 0.00 |
| Cont.0.70Load.000Cross.4Var.500Size.2Fac.0.70Fcor | 0.00 | 0.00 | 0.16 | 0.68 | 0.00 | 0.00 | 0.06 | 0.02 | -0.18 | -0.75 | 0.00 | 0.00 | 0.06 | 0.20 | 0.18 | 0.75 |
| Cont.0.70Load.000Cross.4Var.1000Size.2Fac.0.00Fcor | 0.00 | 0.00 | 0.21 | 0.31 | 0.00 | 0.00 | 0.06 | 0.05 | 0.00 | 0.00 | 0.00 | 0.00 | 0.06 | 0.09 | 0.00 | 0.00 |
| Cont.0.70Load.000Cross.4Var.1000Size.2Fac.0.50Fcor | 0.00 | 0.00 | 0.24 | 0.59 | 0.00 | 0.00 | 0.04 | 0.04 | 0.00 | 0.00 | 0.00 | 0.00 | 0.04 | 0.16 | 0.00 | 0.00 |
| Cont.0.70Load.000Cross.4Var.1000Size.2Fac.0.70Fcor | 0.00 | 0.00 | 0.23 | 0.79 | 0.00 | 0.00 | 0.06 | -0.02 | -0.11 | -0.57 | 0.00 | 0.00 | 0.06 | 0.20 | 0.11 | 0.57 |
| Cont.0.70Load.000Cross.4Var.300Size.4Fac.0.00Fcor | 0.00 | 0.00 | 1.66 | 0.60 | 0.00 | 0.00 | -0.35 | 0.12 | 0.00 | 0.00 | 0.00 | 0.00 | 0.45 | 0.16 | 0.00 | 0.00 |
| Cont.0.70Load.000Cross.4Var.300Size.4Fac.0.50Fcor | 0.00 | 0.00 | 0.96 | 1.07 | 0.00 | 0.00 | -1.34 | 0.07 | 0.00 | -0.15 | 0.00 | 0.00 | 1.34 | 0.17 | 0.00 | 0.15 |
| Cont.0.70Load.000Cross.4Var.300Size.4Fac.0.70Fcor | 0.30 | 0.30 | 0.93 | 1.32 | -0.01 | -0.01 | -1.31 | 0.16 | -0.05 | -2.76 | 0.11 | 0.11 | 1.35 | 0.34 | 0.05 | 2.76 |
| Cont.0.70Load.000Cross.4Var.500Size.4Fac.0.00Fcor | 0.00 | 0.00 | 1.07 | 0.49 | 0.00 | 0.00 | -0.22 | 0.09 | 0.00 | 0.00 | 0.00 | 0.00 | 0.28 | 0.11 | 0.00 | 0.00 |
| Cont.0.70Load.000Cross.4Var.500Size.4Fac.0.50Fcor | 0.00 | 0.00 | 0.86 | 0.68 | 0.00 | 0.00 | -1.36 | 0.18 | 0.00 | -0.04 | 0.00 | 0.00 | 1.42 | 0.22 | 0.00 | 0.04 |
| Cont.0.70Load.000Cross.4Var.500Size.4Fac.0.70Fcor | 0.14 | 0.14 | 0.82 | 0.97 | -0.02 | -0.02 | -1.43 | 0.11 | 0.00 | -2.60 | 0.04 | 0.04 | 1.53 | 0.23 | 0.00 | 2.60 |
| Cont.0.70Load.000Cross.4Var.1000Size.4Fac.0.00Fcor | 0.00 | 0.00 | 0.73 | 0.43 | 0.00 | 0.00 | -0.32 | 0.22 | 0.00 | 0.00 | 0.00 | 0.00 | 0.46 | 0.24 | 0.00 | 0.00 |
| Cont.0.70Load.000Cross.4Var.1000Size.4Fac.0.50Fcor | 0.00 | 0.00 | 0.95 | 0.68 | 0.00 | 0.00 | -1.44 | 0.08 | 0.00 | 0.00 | 0.00 | 0.00 | 1.46 | 0.14 | 0.00 | 0.00 |
| Cont.0.70Load.000Cross.4Var.1000Size.4Fac.0.70Fcor | 0.01 | 0.01 | 0.45 | 0.54 | 0.00 | 0.00 | -1.30 | 0.06 | 0.00 | -2.22 | 0.00 | 0.00 | 1.30 | 0.12 | 0.00 | 2.22 |
| Cont.0.70Load.000Cross.8Var.300Size.2Fac.0.00Fcor | 0.00 | 0.00 | 1.90 | 3.06 | 0.00 | 0.00 | 0.94 | 1.30 | 0.00 | 0.00 | 0.00 | 0.00 | 0.94 | 1.30 | 0.00 | 0.00 |
| Cont.0.70Load.000Cross.8Var.300Size.2Fac.0.50Fcor | 0.00 | 0.00 | 2.21 | 3.00 | 0.00 | 0.00 | 0.63 | 1.19 | 0.00 | 0.00 | 0.00 | 0.00 | 0.63 | 1.19 | 0.00 | 0.00 |
| Cont.0.70Load.000Cross.8Var.300Size.2Fac.0.70Fcor | 0.01 | 0.01 | 2.31 | 3.09 | 0.00 | 0.00 | 0.62 | 1.05 | 0.00 | 0.00 | 0.00 | 0.00 | 0.62 | 1.05 | 0.00 | 0.00 |
| Cont.0.70Load.000Cross.8Var.500Size.2Fac.0.00Fcor | 0.00 | 0.00 | 2.29 | 2.97 | 0.00 | 0.00 | 0.57 | 0.82 | 0.00 | 0.00 | 0.00 | 0.00 | 0.57 | 0.82 | 0.00 | 0.00 |
| Cont.0.70Load.000Cross.8Var.500Size.2Fac.0.50Fcor | 0.00 | 0.00 | 1.55 | 2.71 | 0.00 | 0.00 | 0.62 | 0.91 | 0.00 | 0.00 | 0.00 | 0.00 | 0.62 | 0.91 | 0.00 | 0.00 |
| Cont.0.70Load.000Cross.8Var.500Size.2Fac.0.70Fcor | 0.00 | 0.00 | 1.15 | 2.17 | 0.00 | 0.00 | 0.34 | 0.90 | 0.00 | 0.00 | 0.00 | 0.00 | 0.34 | 0.90 | 0.00 | 0.00 |
| Cont.0.70Load.000Cross.8Var.1000Size.2Fac.0.00Fcor | 0.00 | 0.00 | 1.43 | 2.42 | 0.00 | 0.00 | 0.64 | 1.01 | 0.00 | 0.00 | 0.00 | 0.00 | 0.64 | 1.01 | 0.00 | 0.00 |
| Cont.0.70Load.000Cross.8Var.1000Size.2Fac.0.50Fcor | 0.00 | 0.00 | 1.68 | 1.86 | 0.00 | 0.00 | 0.50 | 0.96 | 0.00 | 0.00 | 0.00 | 0.00 | 0.50 | 0.96 | 0.00 | 0.00 |
| Cont.0.70Load.000Cross.8Var.1000Size.2Fac.0.70Fcor | 0.00 | 0.00 | 1.41 | 2.69 | 0.00 | 0.00 | 0.31 | 0.66 | 0.00 | 0.00 | 0.00 | 0.00 | 0.31 | 0.66 | 0.00 | 0.00 |
| Cont.0.70Load.000Cross.8Var.300Size.4Fac.0.00Fcor | 0.00 | 0.00 | 6.72 | 3.78 | 0.00 | 0.00 | 0.09 | 1.73 | 0.00 | 0.00 | 0.00 | 0.00 | 1.13 | 1.73 | 0.00 | 0.00 |
| Cont.0.70Load.000Cross.8Var.300Size.4Fac.0.50Fcor | 0.00 | 0.00 | 2.24 | 4.47 | 0.00 | 0.00 | -0.59 | 1.97 | 0.00 | 0.00 | 0.00 | 0.00 | 1.29 | 1.97 | 0.00 | 0.00 |
| Cont.0.70Load.000Cross.8Var.300Size.4Fac.0.70Fcor | 0.09 | 0.09 | 2.60 | 4.47 | 0.06 | 0.06 | -0.98 | 1.94 | 0.00 | -0.37 | 0.06 | 0.06 | 1.30 | 1.94 | 0.00 | 0.37 |
| Cont.0.70Load.000Cross.8Var.500Size.4Fac.0.00Fcor | 0.00 | 0.00 | 6.69 | 3.32 | 0.00 | 0.00 | -0.39 | 1.13 | 0.00 | 0.00 | 0.00 | 0.00 | 1.09 | 1.13 | 0.00 | 0.00 |
| Cont.0.70Load.000Cross.8Var.500Size.4Fac.0.50Fcor | 0.00 | 0.00 | 1.79 | 2.98 | 0.00 | 0.00 | -0.99 | 1.12 | 0.00 | 0.00 | 0.00 | 0.00 | 1.23 | 1.12 | 0.00 | 0.00 |
| Cont.0.70Load.000Cross.8Var.500Size.4Fac.0.70Fcor | 0.05 | 0.05 | 1.19 | 2.90 | 0.02 | 0.02 | -1.12 | 1.11 | 0.00 | -0.02 | 0.02 | 0.02 | 1.34 | 1.11 | 0.00 | 0.02 |
| Cont.0.70Load.000Cross.8Var.1000Size.4Fac.0.00Fcor | 0.00 | 0.00 | 5.23 | 4.00 | 0.00 | 0.00 | -0.08 | 1.59 | 0.00 | 0.00 | 0.00 | 0.00 | 0.98 | 1.59 | 0.00 | 0.00 |
| Cont.0.70Load.000Cross.8Var.1000Size.4Fac.0.50Fcor | 0.00 | 0.00 | 4.29 | 3.95 | 0.00 | 0.00 | -1.09 | 0.84 | 0.00 | 0.00 | 0.00 | 0.00 | 1.33 | 0.84 | 0.00 | 0.00 |
| Cont.0.70Load.000Cross.8Var.1000Size.4Fac.0.70Fcor | 0.00 | 0.00 | 3.10 | 2.72 | 0.00 | 0.00 | -0.93 | 1.06 | 0.00 | 0.00 | 0.00 | 0.00 | 1.27 | 1.06 | 0.00 | 0.00 |
| Cont.0.70Load.125Cross.4Var.300Size.2Fac.0.00Fcor | 0.00 | 0.00 | 0.39 | 0.99 | 0.00 | 0.00 | 0.03 | 0.09 | 0.00 | 0.00 | 0.00 | 0.00 | 0.03 | 0.15 | 0.00 | 0.00 |
| Cont.0.70Load.125Cross.4Var.300Size.2Fac.0.50Fcor | 0.00 | 0.00 | 0.25 | 1.68 | 0.00 | 0.00 | 0.07 | -0.09 | -0.02 | -0.08 | 0.00 | 0.00 | 0.07 | 0.43 | 0.02 | 0.08 |
| Cont.0.70Load.125Cross.4Var.300Size.2Fac.0.70Fcor | 0.05 | 0.05 | 0.28 | 1.73 | 0.00 | 0.00 | 0.13 | -0.06 | -0.69 | -0.93 | 0.00 | 0.00 | 0.13 | 0.38 | 0.69 | 0.93 |
| Cont.0.70Load.125Cross.4Var.500Size.2Fac.0.00Fcor | 0.00 | 0.00 | 0.36 | 0.93 | 0.00 | 0.00 | 0.03 | 0.07 | 0.00 | 0.00 | 0.00 | 0.00 | 0.03 | 0.09 | 0.00 | 0.00 |
| Cont.0.70Load.125Cross.4Var.500Size.2Fac.0.50Fcor | 0.00 | 0.00 | 0.32 | 1.73 | 0.00 | 0.00 | 0.03 | -0.13 | -0.01 | -0.03 | 0.00 | 0.00 | 0.03 | 0.35 | 0.01 | 0.03 |
| Cont.0.70Load.125Cross.4Var.500Size.2Fac.0.70Fcor | 0.01 | 0.01 | 0.45 | 1.85 | 0.00 | 0.00 | 0.01 | -0.15 | -0.81 | -0.95 | 0.00 | 0.00 | 0.01 | 0.23 | 0.81 | 0.95 |
| Cont.0.70Load.125Cross.4Var.1000Size.2Fac.0.00Fcor | 0.00 | 0.00 | 0.42 | 0.79 | 0.00 | 0.00 | 0.01 | 0.02 | 0.00 | 0.00 | 0.00 | 0.00 | 0.01 | 0.02 | 0.00 | 0.00 |
| Cont.0.70Load.125Cross.4Var.1000Size.2Fac.0.50Fcor | 0.00 | 0.00 | 0.26 | 2.30 | 0.00 | 0.00 | 0.04 | -0.36 | 0.00 | 0.00 | 0.00 | 0.00 | 0.04 | 0.50 | 0.00 | 0.00 |
| Cont.0.70Load.125Cross.4Var.1000Size.2Fac.0.70Fcor | 0.00 | 0.00 | 0.29 | 1.68 | 0.00 | 0.00 | 0.01 | -0.22 | -0.89 | -0.94 | 0.00 | 0.00 | 0.01 | 0.24 | 0.89 | 0.94 |
| Cont.0.70Load.125Cross.4Var.300Size.4Fac.0.00Fcor | 0.00 | 0.00 | 1.00 | 1.71 | 0.00 | 0.00 | -0.96 | 0.02 | -0.01 | 0.00 | 0.00 | 0.00 | 0.98 | 0.14 | 0.01 | 0.00 |
| Cont.0.70Load.125Cross.4Var.300Size.4Fac.0.50Fcor | 0.05 | 0.05 | 0.58 | 1.52 | -0.04 | -0.03 | -1.35 | 0.21 | -0.08 | -0.59 | 0.06 | 0.05 | 1.35 | 0.35 | 0.08 | 0.59 |
| Cont.0.70Load.125Cross.4Var.300Size.4Fac.0.70Fcor | 0.49 | 0.49 | 0.60 | 1.84 | -0.16 | -0.16 | -1.57 | 0.05 | -0.48 | -2.85 | 0.28 | 0.28 | 1.57 | 0.35 | 0.48 | 2.85 |
| Cont.0.70Load.125Cross.4Var.500Size.4Fac.0.00Fcor | 0.00 | 0.00 | 0.43 | 1.72 | 0.00 | 0.00 | -1.25 | 0.07 | 0.00 | 0.00 | 0.00 | 0.00 | 1.25 | 0.09 | 0.00 | 0.00 |
| Cont.0.70Load.125Cross.4Var.500Size.4Fac.0.50Fcor | 0.01 | 0.01 | 0.31 | 1.36 | -0.01 | -0.01 | -1.46 | 0.25 | -0.08 | -0.17 | 0.01 | 0.01 | 1.46 | 0.33 | 0.08 | 0.17 |
| Cont.0.70Load.125Cross.4Var.500Size.4Fac.0.70Fcor | 0.39 | 0.39 | 0.30 | 1.51 | -0.20 | -0.20 | -1.46 | -0.02 | -0.27 | -2.74 | 0.22 | 0.22 | 1.48 | 0.22 | 0.27 | 2.74 |
| Cont.0.70Load.125Cross.4Var.1000Size.4Fac.0.00Fcor | 0.00 | 0.00 | 0.04 | 1.53 | 0.00 | 0.00 | -1.63 | 0.08 | -0.03 | 0.00 | 0.00 | 0.00 | 1.63 | 0.08 | 0.03 | 0.00 |
| Cont.0.70Load.125Cross.4Var.1000Size.4Fac.0.50Fcor | 0.02 | 0.02 | 0.53 | 0.95 | 0.01 | 0.01 | -1.45 | 0.09 | -0.06 | -0.06 | 0.01 | 0.01 | 1.45 | 0.19 | 0.06 | 0.06 |
| Cont.0.70Load.125Cross.4Var.1000Size.4Fac.0.70Fcor | 0.17 | 0.17 | 0.49 | 1.30 | -0.10 | -0.10 | -1.45 | -0.06 | -0.32 | -2.47 | 0.12 | 0.12 | 1.47 | 0.12 | 0.32 | 2.47 |
| Cont.0.70Load.125Cross.8Var.300Size.2Fac.0.00Fcor | 0.19 | 0.30 | 2.42 | 3.58 | 0.07 | 0.09 | 0.51 | 1.25 | 0.60 | 0.00 | 0.07 | 0.09 | 0.51 | 1.25 | 0.60 | 0.00 |
| Cont.0.70Load.125Cross.8Var.300Size.2Fac.0.50Fcor | 0.02 | 0.02 | 1.43 | 2.47 | 0.00 | 0.00 | 1.04 | 1.66 | 0.33 | 0.00 | 0.00 | 0.00 | 1.04 | 1.66 | 0.33 | 0.00 |
| Cont.0.70Load.125Cross.8Var.300Size.2Fac.0.70Fcor | 0.00 | 0.00 | 2.52 | 3.64 | 0.00 | 0.00 | 0.54 | 0.68 | 0.06 | -0.09 | 0.00 | 0.00 | 0.54 | 0.70 | 0.06 | 0.09 |
| Cont.0.70Load.125Cross.8Var.500Size.2Fac.0.00Fcor | 0.24 | 0.50 | 2.27 | 3.27 | 0.06 | 0.08 | 0.44 | 0.93 | 0.64 | 0.00 | 0.06 | 0.08 | 0.44 | 0.93 | 0.64 | 0.00 |
| Cont.0.70Load.125Cross.8Var.500Size.2Fac.0.50Fcor | 0.02 | 0.02 | 2.17 | 2.58 | 0.00 | 0.00 | 1.01 | 1.41 | 0.27 | 0.00 | 0.00 | 0.00 | 1.01 | 1.41 | 0.27 | 0.00 |
| Cont.0.70Load.125Cross.8Var.500Size.2Fac.0.70Fcor | 0.00 | 0.00 | 2.22 | 3.09 | 0.00 | 0.00 | 0.27 | 0.40 | 0.01 | 0.00 | 0.00 | 0.00 | 0.27 | 0.42 | 0.01 | 0.00 |
| Cont.0.70Load.125Cross.8Var.1000Size.2Fac.0.00Fcor | 0.31 | 0.46 | 1.55 | 2.92 | 0.08 | 0.08 | 0.35 | 0.76 | 0.65 | 0.00 | 0.08 | 0.08 | 0.35 | 0.76 | 0.65 | 0.00 |
| Cont.0.70Load.125Cross.8Var.1000Size.2Fac.0.50Fcor | 0.00 | 0.00 | 2.51 | 2.59 | 0.00 | 0.00 | 0.59 | 0.83 | 0.29 | 0.00 | 0.00 | 0.00 | 0.59 | 0.83 | 0.29 | 0.00 |
| Cont.0.70Load.125Cross.8Var.1000Size.2Fac.0.70Fcor | 0.00 | 0.00 | 2.43 | 3.59 | 0.00 | 0.00 | 0.20 | 0.09 | 0.00 | 0.00 | 0.00 | 0.00 | 0.20 | 0.15 | 0.00 | 0.00 |
| Cont.0.70Load.125Cross.8Var.300Size.4Fac.0.00Fcor | 0.00 | 0.00 | 3.96 | 4.89 | 0.00 | 0.00 | -0.30 | 1.42 | 0.00 | 0.00 | 0.00 | 0.00 | 1.14 | 1.42 | 0.00 | 0.00 |
| Cont.0.70Load.125Cross.8Var.300Size.4Fac.0.50Fcor | 0.00 | 0.00 | 1.81 | 4.00 | 0.04 | 0.04 | -0.84 | 2.40 | 0.09 | 0.00 | 0.04 | 0.04 | 1.36 | 2.40 | 0.09 | 0.00 |
| Cont.0.70Load.125Cross.8Var.300Size.4Fac.0.70Fcor | 0.22 | 0.22 | 1.85 | 4.94 | 0.01 | 0.01 | -1.00 | 2.07 | 0.02 | -0.63 | 0.05 | 0.05 | 1.40 | 2.07 | 0.02 | 0.63 |
| Cont.0.70Load.125Cross.8Var.500Size.4Fac.0.00Fcor | 0.00 | 0.00 | 4.49 | 4.77 | 0.00 | 0.00 | -0.59 | 0.88 | 0.00 | 0.00 | 0.00 | 0.00 | 0.93 | 0.88 | 0.00 | 0.00 |
| Cont.0.70Load.125Cross.8Var.500Size.4Fac.0.50Fcor | 0.00 | 0.00 | 1.98 | 4.42 | 0.00 | 0.00 | -1.04 | 1.73 | 0.06 | 0.00 | 0.00 | 0.00 | 1.26 | 1.73 | 0.06 | 0.00 |
| Cont.0.70Load.125Cross.8Var.500Size.4Fac.0.70Fcor | 0.01 | 0.01 | 3.02 | 4.30 | 0.00 | 0.00 | -1.01 | 0.94 | 0.02 | -0.07 | 0.00 | 0.00 | 1.27 | 0.94 | 0.02 | 0.07 |
| Cont.0.70Load.125Cross.8Var.1000Size.4Fac.0.00Fcor | 0.00 | 0.00 | 3.29 | 4.68 | 0.00 | 0.00 | -0.87 | 1.17 | 0.00 | 0.00 | 0.00 | 0.00 | 1.11 | 1.17 | 0.00 | 0.00 |
| Cont.0.70Load.125Cross.8Var.1000Size.4Fac.0.50Fcor | 0.00 | 0.00 | 1.99 | 3.16 | 0.00 | 0.00 | -0.91 | 1.15 | 0.08 | 0.00 | 0.00 | 0.00 | 1.41 | 1.15 | 0.08 | 0.00 |
| Cont.0.70Load.125Cross.8Var.1000Size.4Fac.0.70Fcor | 0.01 | 0.01 | 3.28 | 4.06 | 0.01 | 0.01 | -1.31 | 0.80 | 0.00 | 0.00 | 0.01 | 0.01 | 1.41 | 0.80 | 0.00 | 0.00 |
| Cont.0.70Load.Cross250.4Var.300Size.2Fac.0.00Fcor | 0.39 | 0.47 | 0.59 | 1.57 | 0.04 | 0.07 | 0.10 | 0.18 | 0.27 | 0.00 | 0.04 | 0.07 | 0.10 | 0.18 | 0.27 | 0.00 |
| Cont.0.70Load.Cross250.4Var.300Size.2Fac.0.50Fcor | 0.00 | 0.00 | 0.36 | 1.51 | 0.01 | 0.01 | 0.15 | -0.08 | -0.12 | -0.22 | 0.01 | 0.01 | 0.15 | 0.38 | 0.16 | 0.22 |
| Cont.0.70Load.Cross250.4Var.300Size.2Fac.0.70Fcor | 0.00 | 0.00 | 0.25 | 1.62 | 0.02 | 0.02 | 0.03 | -0.29 | -0.98 | -0.99 | 0.02 | 0.02 | 0.03 | 0.37 | 0.98 | 0.99 |
| Cont.0.70Load.Cross250.4Var.500Size.2Fac.0.00Fcor | 0.18 | 0.20 | 0.50 | 1.85 | 0.02 | 0.04 | 0.15 | 0.05 | 0.24 | 0.00 | 0.02 | 0.04 | 0.15 | 0.05 | 0.24 | 0.00 |
| Cont.0.70Load.Cross250.4Var.500Size.2Fac.0.50Fcor | 0.00 | 0.00 | 0.38 | 1.52 | 0.00 | 0.00 | 0.13 | -0.10 | -0.17 | -0.19 | 0.00 | 0.00 | 0.13 | 0.34 | 0.21 | 0.19 |
| Cont.0.70Load.Cross250.4Var.500Size.2Fac.0.70Fcor | 0.00 | 0.00 | 0.23 | 1.70 | 0.00 | 0.00 | 0.00 | -0.35 | -0.99 | -1.00 | 0.00 | 0.00 | 0.00 | 0.35 | 0.99 | 1.00 |
| Cont.0.70Load.Cross250.4Var.1000Size.2Fac.0.00Fcor | 0.02 | 0.02 | 0.85 | 1.78 | 0.01 | 0.01 | 0.14 | 0.11 | 0.18 | 0.00 | 0.01 | 0.01 | 0.14 | 0.11 | 0.18 | 0.00 |
| Cont.0.70Load.Cross250.4Var.1000Size.2Fac.0.50Fcor | 0.00 | 0.00 | 0.29 | 1.31 | 0.00 | 0.00 | 0.21 | 0.02 | -0.19 | -0.05 | 0.00 | 0.00 | 0.21 | 0.44 | 0.19 | 0.05 |
| Cont.0.70Load.Cross250.4Var.1000Size.2Fac.0.70Fcor | 0.00 | 0.00 | 0.29 | 1.45 | 0.00 | 0.00 | 0.00 | -0.28 | -1.00 | -1.00 | 0.00 | 0.00 | 0.00 | 0.28 | 1.00 | 1.00 |
| Cont.0.70Load.Cross250.4Var.300Size.4Fac.0.00Fcor | 0.00 | 0.00 | 1.17 | 2.63 | 0.00 | 0.00 | -1.06 | 0.04 | 0.00 | 0.00 | 0.00 | 0.00 | 1.08 | 0.04 | 0.00 | 0.00 |
| Cont.0.70Load.Cross250.4Var.300Size.4Fac.0.50Fcor | 0.00 | 0.00 | 0.33 | 1.24 | 0.00 | 0.00 | -1.33 | 0.44 | -0.16 | -0.79 | 0.00 | 0.00 | 1.35 | 0.44 | 0.16 | 0.79 |
| Cont.0.70Load.Cross250.4Var.300Size.4Fac.0.70Fcor | 0.39 | 0.39 | 1.12 | 1.33 | -0.13 | -0.13 | -1.32 | 0.21 | -1.05 | -2.95 | 0.19 | 0.19 | 1.34 | 0.33 | 1.05 | 2.95 |
| Cont.0.70Load.Cross250.4Var.500Size.4Fac.0.00Fcor | 0.00 | 0.00 | 0.51 | 3.36 | 0.00 | 0.00 | -1.07 | 0.04 | -0.01 | 0.00 | 0.00 | 0.00 | 1.07 | 0.04 | 0.01 | 0.00 |
| Cont.0.70Load.Cross250.4Var.500Size.4Fac.0.50Fcor | 0.00 | 0.00 | 0.32 | 0.71 | -0.01 | -0.01 | -1.29 | 0.44 | -0.07 | -0.43 | 0.01 | 0.01 | 1.37 | 0.44 | 0.09 | 0.43 |
| Cont.0.70Load.Cross250.4Var.500Size.4Fac.0.70Fcor | 0.15 | 0.15 | 0.80 | 1.44 | -0.04 | -0.04 | -1.25 | 0.17 | -1.19 | -2.89 | 0.04 | 0.04 | 1.25 | 0.23 | 1.21 | 2.89 |
| Cont.0.70Load.Cross250.4Var.1000Size.4Fac.0.00Fcor | 0.00 | 0.00 | 0.33 | 3.68 | 0.00 | 0.00 | -1.14 | 0.00 | -0.02 | 0.00 | 0.00 | 0.00 | 1.18 | 0.00 | 0.02 | 0.00 |
| Cont.0.70Load.Cross250.4Var.1000Size.4Fac.0.50Fcor | 0.00 | 0.00 | 0.40 | 1.05 | 0.00 | 0.00 | -1.12 | 0.23 | -0.06 | -0.11 | 0.00 | 0.00 | 1.14 | 0.23 | 0.06 | 0.11 |
| Cont.0.70Load.Cross250.4Var.1000Size.4Fac.0.70Fcor | 0.07 | 0.07 | 0.51 | 1.29 | -0.02 | -0.02 | -1.30 | 0.12 | -1.56 | -2.74 | 0.02 | 0.02 | 1.32 | 0.12 | 1.58 | 2.74 |
| Cont.0.70Load.Cross250.8Var.300Size.2Fac.0.00Fcor | 0.19 | 0.30 | 2.42 | 3.58 | 0.07 | 0.09 | 0.51 | 1.25 | 0.60 | 0.00 | 0.07 | 0.09 | 0.51 | 1.25 | 0.60 | 0.00 |
| Cont.0.70Load.Cross250.8Var.300Size.2Fac.0.50Fcor | 0.02 | 0.02 | 1.43 | 2.47 | 0.00 | 0.00 | 1.04 | 1.66 | 0.33 | 0.00 | 0.00 | 0.00 | 1.04 | 1.66 | 0.33 | 0.00 |
| Cont.0.70Load.Cross250.8Var.300Size.2Fac.0.70Fcor | 0.00 | 0.00 | 2.52 | 3.64 | 0.00 | 0.00 | 0.54 | 0.68 | 0.06 | -0.09 | 0.00 | 0.00 | 0.54 | 0.70 | 0.06 | 0.09 |
| Cont.0.70Load.Cross250.8Var.500Size.2Fac.0.00Fcor | 0.24 | 0.50 | 2.27 | 3.27 | 0.06 | 0.08 | 0.44 | 0.93 | 0.64 | 0.00 | 0.06 | 0.08 | 0.44 | 0.93 | 0.64 | 0.00 |
| Cont.0.70Load.Cross250.8Var.500Size.2Fac.0.50Fcor | 0.02 | 0.02 | 2.17 | 2.58 | 0.00 | 0.00 | 1.01 | 1.41 | 0.27 | 0.00 | 0.00 | 0.00 | 1.01 | 1.41 | 0.27 | 0.00 |
| Cont.0.70Load.Cross250.8Var.500Size.2Fac.0.70Fcor | 0.00 | 0.00 | 2.22 | 3.09 | 0.00 | 0.00 | 0.27 | 0.40 | 0.01 | 0.00 | 0.00 | 0.00 | 0.27 | 0.42 | 0.01 | 0.00 |
| Cont.0.70Load.Cross250.8Var.1000Size.2Fac.0.00Fcor | 0.31 | 0.46 | 1.55 | 2.92 | 0.08 | 0.08 | 0.35 | 0.76 | 0.65 | 0.00 | 0.08 | 0.08 | 0.35 | 0.76 | 0.65 | 0.00 |
| Cont.0.70Load.Cross250.8Var.1000Size.2Fac.0.50Fcor | 0.00 | 0.00 | 2.51 | 2.59 | 0.00 | 0.00 | 0.59 | 0.83 | 0.29 | 0.00 | 0.00 | 0.00 | 0.59 | 0.83 | 0.29 | 0.00 |
| Cont.0.70Load.Cross250.8Var.1000Size.2Fac.0.70Fcor | 0.00 | 0.00 | 2.43 | 3.59 | 0.00 | 0.00 | 0.20 | 0.09 | 0.00 | 0.00 | 0.00 | 0.00 | 0.20 | 0.15 | 0.00 | 0.00 |
| Cont.0.70Load.Cross250.8Var.300Size.4Fac.0.00Fcor | 0.03 | 0.03 | 0.64 | 5.75 | 0.00 | 0.00 | -1.63 | 1.67 | 0.04 | 0.00 | 0.00 | 0.00 | 1.71 | 1.67 | 0.04 | 0.00 |
| Cont.0.70Load.Cross250.8Var.300Size.4Fac.0.50Fcor | 0.00 | 0.00 | 1.23 | 3.19 | 0.01 | 0.01 | -0.97 | 2.80 | 0.68 | 0.00 | 0.01 | 0.01 | 1.47 | 2.80 | 0.68 | 0.00 |
| Cont.0.70Load.Cross250.8Var.300Size.4Fac.0.70Fcor | 0.21 | 0.20 | 1.57 | 5.09 | 0.04 | 0.04 | -0.95 | 2.20 | 0.26 | -1.02 | 0.04 | 0.04 | 1.49 | 2.20 | 0.34 | 1.02 |
| Cont.0.70Load.Cross250.8Var.500Size.4Fac.0.00Fcor | 0.02 | 0.02 | 0.81 | 4.81 | 0.00 | 0.00 | -1.46 | 1.44 | 0.21 | 0.00 | 0.00 | 0.00 | 1.52 | 1.44 | 0.21 | 0.00 |
| Cont.0.70Load.Cross250.8Var.500Size.4Fac.0.50Fcor | 0.00 | 0.00 | 1.40 | 4.61 | 0.00 | 0.00 | -1.16 | 2.66 | 0.73 | 0.00 | 0.00 | 0.00 | 1.48 | 2.66 | 0.73 | 0.00 |
| Cont.0.70Load.Cross250.8Var.500Size.4Fac.0.70Fcor | 0.01 | 0.01 | 2.59 | 5.02 | 0.00 | 0.00 | -0.78 | 1.22 | 0.31 | -0.22 | 0.00 | 0.00 | 1.36 | 1.22 | 0.31 | 0.22 |
| Cont.0.70Load.Cross250.8Var.1000Size.4Fac.0.00Fcor | 0.00 | 0.00 | 0.12 | 4.87 | 0.00 | 0.01 | -1.54 | 1.22 | 0.06 | 0.00 | 0.00 | 0.01 | 1.58 | 1.22 | 0.06 | 0.00 |
| Cont.0.70Load.Cross250.8Var.1000Size.4Fac.0.50Fcor | 0.00 | 0.00 | 1.69 | 4.26 | 0.00 | 0.00 | -1.54 | 2.25 | 1.17 | 0.00 | 0.00 | 0.00 | 1.62 | 2.25 | 1.17 | 0.00 |
| Cont.0.70Load.Cross250.8Var.1000Size.4Fac.0.70Fcor | 0.00 | 0.00 | 3.98 | 5.41 | 0.00 | 0.00 | -1.07 | 0.85 | 0.55 | 0.00 | 0.00 | 0.00 | 1.25 | 0.85 | 0.55 | 0.00 |
| Ord.0.40Load.000Cross.4Var.300Size.2Fac.0.00Fcor | 3.91 | 3.90 | 4.93 | 5.08 | -0.82 | -0.82 | -1.05 | -1.07 | -0.21 | 0.02 | 0.90 | 0.90 | 1.19 | 1.21 | 0.33 | 0.06 |
| Ord.0.40Load.000Cross.4Var.300Size.2Fac.0.50Fcor | 3.54 | 3.52 | 4.42 | 4.91 | -0.73 | -0.72 | -0.90 | -1.00 | -0.33 | -0.54 | 0.89 | 0.88 | 1.06 | 1.18 | 0.51 | 0.56 |
| Ord.0.40Load.000Cross.4Var.300Size.2Fac.0.70Fcor | 3.54 | 3.52 | 4.77 | 5.31 | -0.87 | -0.86 | -1.02 | -1.10 | -0.35 | -0.91 | 0.99 | 1.00 | 1.18 | 1.26 | 0.61 | 0.91 |
| Ord.0.40Load.000Cross.4Var.500Size.2Fac.0.00Fcor | 1.33 | 1.33 | 2.21 | 2.77 | -0.23 | -0.23 | -0.39 | -0.48 | 0.00 | 0.00 | 0.33 | 0.33 | 0.49 | 0.64 | 0.10 | 0.00 |
| Ord.0.40Load.000Cross.4Var.500Size.2Fac.0.50Fcor | 1.67 | 1.67 | 2.58 | 3.31 | -0.26 | -0.26 | -0.44 | -0.56 | 0.10 | -0.40 | 0.44 | 0.44 | 0.64 | 0.78 | 0.16 | 0.40 |
| Ord.0.40Load.000Cross.4Var.500Size.2Fac.0.70Fcor | 1.55 | 1.55 | 4.23 | 4.62 | -0.48 | -0.48 | -0.90 | -0.95 | -0.24 | -0.90 | 0.58 | 0.58 | 0.94 | 1.05 | 0.36 | 0.90 |
| Ord.0.40Load.000Cross.4Var.1000Size.2Fac.0.00Fcor | 0.10 | 0.11 | 0.53 | 1.19 | 0.00 | 0.00 | 0.02 | -0.14 | 0.00 | 0.00 | 0.04 | 0.04 | 0.10 | 0.26 | 0.00 | 0.00 |
| Ord.0.40Load.000Cross.4Var.1000Size.2Fac.0.50Fcor | 0.63 | 0.63 | 1.26 | 1.89 | -0.12 | -0.12 | -0.17 | -0.27 | -0.01 | -0.20 | 0.20 | 0.20 | 0.27 | 0.41 | 0.01 | 0.20 |
| Ord.0.40Load.000Cross.4Var.1000Size.2Fac.0.70Fcor | 0.90 | 0.93 | 4.18 | 4.32 | -0.50 | -0.50 | -0.92 | -0.94 | -0.35 | -0.93 | 0.56 | 0.56 | 0.96 | 1.00 | 0.35 | 0.93 |
| Ord.0.40Load.000Cross.4Var.300Size.4Fac.0.00Fcor | 11.41 | 11.39 | 11.89 | 11.67 | -2.67 | -2.67 | -2.81 | -2.73 | -1.28 | -0.10 | 2.69 | 2.69 | 2.83 | 2.77 | 1.46 | 0.28 |
| Ord.0.40Load.000Cross.4Var.300Size.4Fac.0.50Fcor | 11.71 | 11.71 | 11.72 | 12.21 | -2.77 | -2.77 | -2.88 | -2.81 | -1.64 | -2.22 | 2.85 | 2.85 | 2.96 | 2.91 | 1.86 | 2.24 |
| Ord.0.40Load.000Cross.4Var.300Size.4Fac.0.70Fcor | 9.70 | 9.73 | 9.64 | 10.14 | -2.43 | -2.44 | -2.60 | -2.38 | -1.74 | -2.91 | 2.53 | 2.54 | 2.64 | 2.50 | 1.86 | 2.91 |
| Ord.0.40Load.000Cross.4Var.500Size.4Fac.0.00Fcor | 4.10 | 4.06 | 5.45 | 4.69 | -0.80 | -0.81 | -1.19 | -0.82 | -0.10 | -0.01 | 1.00 | 1.01 | 1.29 | 1.06 | 0.16 | 0.01 |
| Ord.0.40Load.000Cross.4Var.500Size.4Fac.0.50Fcor | 5.63 | 5.63 | 5.65 | 6.58 | -1.35 | -1.35 | -1.59 | -1.35 | -0.65 | -1.88 | 1.51 | 1.51 | 1.73 | 1.53 | 0.83 | 1.88 |
| Ord.0.40Load.000Cross.4Var.500Size.4Fac.0.70Fcor | 5.24 | 5.33 | 5.01 | 6.23 | -1.51 | -1.55 | -1.64 | -1.37 | -1.28 | -2.92 | 1.71 | 1.73 | 1.88 | 1.65 | 1.34 | 2.92 |
| Ord.0.40Load.000Cross.4Var.1000Size.4Fac.0.00Fcor | 0.80 | 0.79 | 2.78 | 1.50 | -0.04 | -0.04 | -0.47 | -0.11 | -0.01 | -0.01 | 0.20 | 0.20 | 0.61 | 0.31 | 0.01 | 0.01 |
| Ord.0.40Load.000Cross.4Var.1000Size.4Fac.0.50Fcor | 4.10 | 4.10 | 3.52 | 5.11 | -1.27 | -1.27 | -1.45 | -1.20 | -0.43 | -1.21 | 1.35 | 1.35 | 1.51 | 1.32 | 0.51 | 1.21 |
| Ord.0.40Load.000Cross.4Var.1000Size.4Fac.0.70Fcor | 4.67 | 4.68 | 5.61 | 6.84 | -1.81 | -1.81 | -1.90 | -1.87 | -1.23 | -2.87 | 1.83 | 1.83 | 1.92 | 1.89 | 1.31 | 2.87 |
| Ord.0.40Load.000Cross.8Var.300Size.2Fac.0.00Fcor | 5.42 | 5.43 | 7.29 | 6.82 | -0.26 | -0.26 | -0.10 | 0.19 | -0.10 | 0.00 | 0.72 | 0.72 | 0.98 | 1.27 | 0.58 | 0.00 |
| Ord.0.40Load.000Cross.8Var.300Size.2Fac.0.50Fcor | 7.67 | 7.67 | 8.00 | 8.27 | -0.15 | -0.13 | -0.34 | 0.03 | -0.25 | -0.09 | 1.27 | 1.29 | 1.14 | 1.51 | 0.85 | 0.09 |
| Ord.0.40Load.000Cross.8Var.300Size.2Fac.0.70Fcor | 6.76 | 6.70 | 6.53 | 7.19 | 0.29 | 0.30 | -0.06 | 0.47 | 0.10 | -0.76 | 1.39 | 1.40 | 1.06 | 1.63 | 0.90 | 0.76 |
| Ord.0.40Load.000Cross.8Var.500Size.2Fac.0.00Fcor | 0.58 | 0.58 | 2.94 | 3.47 | 0.13 | 0.13 | 0.57 | 0.92 | 0.04 | 0.00 | 0.17 | 0.17 | 0.61 | 0.96 | 0.04 | 0.00 |
| Ord.0.40Load.000Cross.8Var.500Size.2Fac.0.50Fcor | 1.90 | 1.95 | 2.65 | 4.10 | 0.43 | 0.44 | 0.52 | 0.87 | 0.08 | 0.00 | 0.53 | 0.54 | 0.60 | 0.95 | 0.08 | 0.00 |
| Ord.0.40Load.000Cross.8Var.500Size.2Fac.0.70Fcor | 3.81 | 3.81 | 2.63 | 4.46 | 0.60 | 0.60 | 0.43 | 0.86 | 0.18 | -0.60 | 0.80 | 0.80 | 0.55 | 1.04 | 0.24 | 0.60 |
| Ord.0.40Load.000Cross.8Var.1000Size.2Fac.0.00Fcor | 0.10 | 0.10 | 2.87 | 3.33 | 0.01 | 0.01 | 0.46 | 0.67 | 0.00 | 0.00 | 0.01 | 0.01 | 0.46 | 0.69 | 0.00 | 0.00 |
| Ord.0.40Load.000Cross.8Var.1000Size.2Fac.0.50Fcor | 0.82 | 0.82 | 2.27 | 3.51 | 0.17 | 0.17 | 0.50 | 0.73 | 0.00 | 0.00 | 0.21 | 0.21 | 0.50 | 0.75 | 0.00 | 0.00 |
| Ord.0.40Load.000Cross.8Var.1000Size.2Fac.0.70Fcor | 2.71 | 2.71 | 3.32 | 4.78 | 0.35 | 0.35 | 0.38 | 0.55 | 0.02 | -0.25 | 0.61 | 0.61 | 0.62 | 0.85 | 0.02 | 0.25 |
| Ord.0.40Load.000Cross.8Var.300Size.4Fac.0.00Fcor | 22.26 | 22.26 | 24.61 | 23.01 | -1.99 | -1.99 | -2.35 | -1.96 | -1.00 | 0.06 | 2.79 | 2.79 | 2.83 | 2.88 | 1.96 | 0.06 |
| Ord.0.40Load.000Cross.8Var.300Size.4Fac.0.50Fcor | 26.26 | 26.26 | 26.32 | 26.50 | -2.58 | -2.58 | -2.86 | -2.62 | -1.50 | -0.97 | 3.14 | 3.14 | 3.02 | 3.24 | 2.28 | 0.97 |
| Ord.0.40Load.000Cross.8Var.300Size.4Fac.0.70Fcor | 24.65 | 24.65 | 24.37 | 24.48 | -2.19 | -2.19 | -2.47 | -2.19 | -0.88 | -2.76 | 2.77 | 2.77 | 2.75 | 2.81 | 2.02 | 2.76 |
| Ord.0.40Load.000Cross.8Var.500Size.4Fac.0.00Fcor | 2.41 | 2.43 | 10.08 | 5.73 | 0.60 | 0.60 | -0.45 | 1.61 | -0.02 | 0.00 | 0.86 | 0.86 | 1.33 | 1.79 | 0.06 | 0.00 |
| Ord.0.40Load.000Cross.8Var.500Size.4Fac.0.50Fcor | 9.59 | 9.59 | 8.85 | 10.33 | 0.60 | 0.60 | -0.19 | 1.06 | 0.20 | -0.30 | 1.50 | 1.50 | 1.37 | 1.70 | 0.38 | 0.30 |
| Ord.0.40Load.000Cross.8Var.500Size.4Fac.0.70Fcor | 12.31 | 12.31 | 8.68 | 10.75 | 0.31 | 0.31 | -0.44 | 0.85 | -0.11 | -2.57 | 1.65 | 1.65 | 1.32 | 1.71 | 0.83 | 2.57 |
| Ord.0.40Load.000Cross.8Var.1000Size.4Fac.0.00Fcor | 0.08 | 0.09 | 10.48 | 3.87 | 0.07 | 0.06 | -0.34 | 1.12 | 0.00 | 0.00 | 0.07 | 0.06 | 0.98 | 1.12 | 0.00 | 0.00 |
| Ord.0.40Load.000Cross.8Var.1000Size.4Fac.0.50Fcor | 6.44 | 6.44 | 5.15 | 6.90 | 0.21 | 0.21 | -0.95 | 0.57 | 0.08 | 0.00 | 1.03 | 1.03 | 1.23 | 1.01 | 0.12 | 0.00 |
| Ord.0.40Load.000Cross.8Var.1000Size.4Fac.0.70Fcor | 12.51 | 12.44 | 8.22 | 11.70 | 0.14 | 0.11 | -0.78 | 0.39 | -0.13 | -1.70 | 1.36 | 1.35 | 1.62 | 1.33 | 0.57 | 1.70 |
| Ord.0.40Load.125Cross.4Var.300Size.2Fac.0.00Fcor | 3.81 | 3.81 | 4.74 | 5.12 | -0.80 | -0.80 | -1.02 | -1.07 | -0.16 | 0.00 | 0.90 | 0.90 | 1.14 | 1.17 | 0.40 | 0.02 |
| Ord.0.40Load.125Cross.4Var.300Size.2Fac.0.50Fcor | 2.56 | 2.55 | 4.28 | 4.56 | -0.50 | -0.50 | -0.83 | -0.87 | -0.08 | -0.66 | 0.72 | 0.72 | 1.01 | 1.09 | 0.26 | 0.68 |
| Ord.0.40Load.125Cross.4Var.300Size.2Fac.0.70Fcor | 1.78 | 1.76 | 4.61 | 5.03 | -0.54 | -0.53 | -0.92 | -1.01 | -0.21 | -0.95 | 0.70 | 0.69 | 1.12 | 1.19 | 0.45 | 0.95 |
| Ord.0.40Load.125Cross.4Var.500Size.2Fac.0.00Fcor | 1.73 | 1.74 | 2.96 | 3.75 | -0.34 | -0.34 | -0.55 | -0.71 | 0.05 | -0.01 | 0.40 | 0.40 | 0.73 | 0.91 | 0.07 | 0.01 |
| Ord.0.40Load.125Cross.4Var.500Size.2Fac.0.50Fcor | 1.15 | 1.20 | 3.67 | 4.20 | -0.36 | -0.35 | -0.69 | -0.79 | -0.09 | -0.51 | 0.52 | 0.51 | 0.87 | 0.99 | 0.11 | 0.53 |
| Ord.0.40Load.125Cross.4Var.500Size.2Fac.0.70Fcor | 1.40 | 1.40 | 4.81 | 5.09 | -0.58 | -0.58 | -1.05 | -1.09 | -0.37 | -0.96 | 0.66 | 0.66 | 1.15 | 1.21 | 0.51 | 0.96 |
| Ord.0.40Load.125Cross.4Var.1000Size.2Fac.0.00Fcor | 0.35 | 0.35 | 0.75 | 1.58 | -0.03 | -0.03 | -0.05 | -0.18 | 0.00 | 0.00 | 0.11 | 0.11 | 0.23 | 0.46 | 0.00 | 0.00 |
| Ord.0.40Load.125Cross.4Var.1000Size.2Fac.0.50Fcor | 0.62 | 0.62 | 2.41 | 2.83 | -0.24 | -0.24 | -0.41 | -0.48 | -0.06 | -0.44 | 0.40 | 0.40 | 0.59 | 0.66 | 0.10 | 0.44 |
| Ord.0.40Load.125Cross.4Var.1000Size.2Fac.0.70Fcor | 0.79 | 0.79 | 4.92 | 5.24 | -0.59 | -0.59 | -1.08 | -1.15 | -0.65 | -0.98 | 0.65 | 0.65 | 1.16 | 1.23 | 0.65 | 0.98 |
| Ord.0.40Load.125Cross.4Var.300Size.4Fac.0.00Fcor | 12.81 | 12.78 | 13.51 | 13.49 | -3.05 | -3.05 | -3.30 | -3.23 | -1.54 | -0.19 | 3.07 | 3.07 | 3.32 | 3.25 | 1.72 | 0.33 |
| Ord.0.40Load.125Cross.4Var.300Size.4Fac.0.50Fcor | 8.46 | 8.46 | 9.00 | 9.86 | -2.38 | -2.38 | -2.55 | -2.45 | -1.23 | -2.30 | 2.42 | 2.42 | 2.57 | 2.51 | 1.41 | 2.32 |
| Ord.0.40Load.125Cross.4Var.300Size.4Fac.0.70Fcor | 6.71 | 6.68 | 6.77 | 7.58 | -1.87 | -1.87 | -1.96 | -1.69 | -1.27 | -2.97 | 1.99 | 1.99 | 2.02 | 1.85 | 1.39 | 2.97 |
| Ord.0.40Load.125Cross.4Var.500Size.4Fac.0.00Fcor | 5.49 | 5.52 | 6.26 | 5.94 | -1.20 | -1.21 | -1.48 | -1.28 | -0.27 | -0.06 | 1.30 | 1.31 | 1.54 | 1.38 | 0.39 | 0.08 |
| Ord.0.40Load.125Cross.4Var.500Size.4Fac.0.50Fcor | 4.72 | 4.72 | 4.46 | 5.43 | -1.47 | -1.47 | -1.58 | -1.39 | -0.79 | -2.28 | 1.61 | 1.61 | 1.68 | 1.53 | 0.87 | 2.28 |
| Ord.0.40Load.125Cross.4Var.500Size.4Fac.0.70Fcor | 5.47 | 5.55 | 5.69 | 6.63 | -1.74 | -1.77 | -1.84 | -1.64 | -1.33 | -2.96 | 1.84 | 1.87 | 1.86 | 1.76 | 1.37 | 2.96 |
| Ord.0.40Load.125Cross.4Var.1000Size.4Fac.0.00Fcor | 1.52 | 1.52 | 2.68 | 2.46 | -0.35 | -0.35 | -0.82 | -0.37 | -0.07 | 0.00 | 0.49 | 0.49 | 0.88 | 0.57 | 0.09 | 0.00 |
| Ord.0.40Load.125Cross.4Var.1000Size.4Fac.0.50Fcor | 4.63 | 4.65 | 4.69 | 6.03 | -1.66 | -1.66 | -1.81 | -1.62 | -0.69 | -1.54 | 1.70 | 1.70 | 1.81 | 1.66 | 0.73 | 1.54 |
| Ord.0.40Load.125Cross.4Var.1000Size.4Fac.0.70Fcor | 4.64 | 4.64 | 7.55 | 8.40 | -2.27 | -2.27 | -2.36 | -2.37 | -1.45 | -2.93 | 2.27 | 2.27 | 2.36 | 2.37 | 1.45 | 2.93 |
| Ord.0.40Load.125Cross.8Var.300Size.2Fac.0.00Fcor | 7.76 | 7.79 | 8.38 | 9.04 | -0.37 | -0.39 | -0.42 | -0.13 | 0.04 | 0.00 | 1.19 | 1.17 | 1.26 | 1.55 | 0.96 | 0.00 |
| Ord.0.40Load.125Cross.8Var.300Size.2Fac.0.50Fcor | 5.05 | 5.04 | 4.60 | 5.73 | 0.40 | 0.43 | 0.26 | 0.71 | 0.55 | -0.29 | 0.88 | 0.91 | 0.66 | 1.11 | 0.65 | 0.29 |
| Ord.0.40Load.125Cross.8Var.300Size.2Fac.0.70Fcor | 5.44 | 5.44 | 4.43 | 5.96 | 0.43 | 0.43 | 0.19 | 0.68 | 0.53 | -0.87 | 0.87 | 0.87 | 0.63 | 1.16 | 0.67 | 0.87 |
| Ord.0.40Load.125Cross.8Var.500Size.2Fac.0.00Fcor | 1.33 | 1.32 | 3.20 | 3.53 | 0.28 | 0.28 | 0.48 | 0.95 | 0.22 | 0.00 | 0.40 | 0.40 | 0.60 | 1.07 | 0.24 | 0.00 |
| Ord.0.40Load.125Cross.8Var.500Size.2Fac.0.50Fcor | 3.16 | 3.16 | 3.83 | 5.18 | 0.29 | 0.29 | 0.33 | 0.59 | 0.15 | -0.06 | 0.59 | 0.59 | 0.53 | 0.81 | 0.15 | 0.06 |
| Ord.0.40Load.125Cross.8Var.500Size.2Fac.0.70Fcor | 4.01 | 4.01 | 4.84 | 6.01 | 0.16 | 0.16 | 0.19 | 0.26 | 0.39 | -0.76 | 0.62 | 0.62 | 0.59 | 0.72 | 0.41 | 0.76 |
| Ord.0.40Load.125Cross.8Var.1000Size.2Fac.0.00Fcor | 0.36 | 0.37 | 2.60 | 3.33 | 0.09 | 0.11 | 0.67 | 0.94 | 0.24 | 0.00 | 0.09 | 0.11 | 0.67 | 0.96 | 0.24 | 0.00 |
| Ord.0.40Load.125Cross.8Var.1000Size.2Fac.0.50Fcor | 1.48 | 1.48 | 2.90 | 3.85 | 0.14 | 0.14 | 0.51 | 0.62 | 0.02 | 0.00 | 0.36 | 0.36 | 0.55 | 0.72 | 0.02 | 0.00 |
| Ord.0.40Load.125Cross.8Var.1000Size.2Fac.0.70Fcor | 2.93 | 2.93 | 4.29 | 5.57 | -0.05 | -0.05 | 0.08 | 0.12 | 0.07 | -0.55 | 0.51 | 0.51 | 0.48 | 0.66 | 0.09 | 0.55 |
| Ord.0.40Load.125Cross.8Var.300Size.4Fac.0.00Fcor | 25.58 | 25.58 | 26.65 | 26.26 | -2.54 | -2.54 | -2.73 | -2.45 | -1.09 | 0.03 | 3.06 | 3.06 | 3.17 | 3.21 | 2.23 | 0.03 |
| Ord.0.40Load.125Cross.8Var.300Size.4Fac.0.50Fcor | 20.33 | 20.37 | 19.53 | 19.76 | -1.28 | -1.27 | -1.65 | -1.10 | -0.17 | -1.13 | 2.26 | 2.25 | 2.31 | 2.30 | 1.65 | 1.13 |
| Ord.0.40Load.125Cross.8Var.300Size.4Fac.0.70Fcor | 16.00 | 16.00 | 15.72 | 15.79 | -0.12 | -0.12 | -0.67 | 0.12 | 0.23 | -2.84 | 1.68 | 1.68 | 1.61 | 1.78 | 1.17 | 2.84 |
| Ord.0.40Load.125Cross.8Var.500Size.4Fac.0.00Fcor | 4.17 | 4.17 | 9.27 | 7.35 | 0.69 | 0.69 | -0.40 | 1.36 | 0.08 | 0.00 | 1.13 | 1.13 | 1.26 | 1.64 | 0.08 | 0.00 |
| Ord.0.40Load.125Cross.8Var.500Size.4Fac.0.50Fcor | 10.44 | 10.44 | 7.41 | 9.71 | 0.57 | 0.57 | -0.62 | 1.02 | -0.05 | -0.39 | 1.55 | 1.55 | 1.34 | 1.54 | 0.53 | 0.39 |
| Ord.0.40Load.125Cross.8Var.500Size.4Fac.0.70Fcor | 12.24 | 12.24 | 9.51 | 11.19 | 0.19 | 0.19 | -0.49 | 0.80 | -0.20 | -2.74 | 1.55 | 1.55 | 1.41 | 1.62 | 0.82 | 2.74 |
| Ord.0.40Load.125Cross.8Var.1000Size.4Fac.0.00Fcor | 0.43 | 0.46 | 7.25 | 4.56 | 0.17 | 0.19 | -0.59 | 1.04 | 0.00 | 0.00 | 0.21 | 0.21 | 1.17 | 1.04 | 0.00 | 0.00 |
| Ord.0.40Load.125Cross.8Var.1000Size.4Fac.0.50Fcor | 7.78 | 7.78 | 7.65 | 8.52 | -0.24 | -0.24 | -1.12 | 0.27 | 0.15 | -0.02 | 1.10 | 1.10 | 1.40 | 0.99 | 0.33 | 0.02 |
| Ord.0.40Load.125Cross.8Var.1000Size.4Fac.0.70Fcor | 11.85 | 11.85 | 10.60 | 13.36 | -0.81 | -0.81 | -1.32 | -0.52 | -0.32 | -2.06 | 1.45 | 1.45 | 1.62 | 1.40 | 0.66 | 2.06 |
| Ord.0.40Load.Cross250.4Var.300Size.2Fac.0.00Fcor | 4.31 | 4.32 | 5.07 | 5.40 | -1.01 | -0.98 | -1.11 | -1.17 | -0.26 | -0.14 | 1.11 | 1.10 | 1.25 | 1.29 | 0.64 | 0.18 |
| Ord.0.40Load.Cross250.4Var.300Size.2Fac.0.50Fcor | 2.22 | 2.20 | 4.75 | 5.11 | -0.57 | -0.57 | -0.99 | -1.02 | -0.17 | -0.80 | 0.67 | 0.67 | 1.09 | 1.20 | 0.33 | 0.80 |
| Ord.0.40Load.Cross250.4Var.300Size.2Fac.0.70Fcor | 1.42 | 1.41 | 5.02 | 5.44 | -0.57 | -0.55 | -1.09 | -1.15 | -0.40 | -0.96 | 0.69 | 0.67 | 1.19 | 1.27 | 0.44 | 0.96 |
| Ord.0.40Load.Cross250.4Var.500Size.2Fac.0.00Fcor | 1.90 | 1.88 | 3.28 | 3.85 | -0.40 | -0.39 | -0.63 | -0.70 | 0.18 | -0.06 | 0.54 | 0.53 | 0.81 | 0.98 | 0.26 | 0.06 |
| Ord.0.40Load.Cross250.4Var.500Size.2Fac.0.50Fcor | 1.26 | 1.26 | 4.46 | 4.77 | -0.51 | -0.51 | -0.93 | -0.98 | -0.23 | -0.77 | 0.61 | 0.61 | 1.03 | 1.08 | 0.27 | 0.77 |
| Ord.0.40Load.Cross250.4Var.500Size.2Fac.0.70Fcor | 0.89 | 0.89 | 4.91 | 5.48 | -0.65 | -0.65 | -1.11 | -1.23 | -0.62 | -1.00 | 0.69 | 0.69 | 1.17 | 1.31 | 0.66 | 1.00 |
| Ord.0.40Load.Cross250.4Var.1000Size.2Fac.0.00Fcor | 0.67 | 0.67 | 1.56 | 2.13 | -0.05 | -0.05 | -0.18 | -0.24 | 0.11 | 0.00 | 0.23 | 0.23 | 0.38 | 0.48 | 0.11 | 0.00 |
| Ord.0.40Load.Cross250.4Var.1000Size.2Fac.0.50Fcor | 0.90 | 0.94 | 3.68 | 4.20 | -0.51 | -0.51 | -0.82 | -0.95 | -0.23 | -0.70 | 0.55 | 0.55 | 0.86 | 0.99 | 0.25 | 0.70 |
| Ord.0.40Load.Cross250.4Var.1000Size.2Fac.0.70Fcor | 0.41 | 0.41 | 6.07 | 6.23 | -0.75 | -0.75 | -1.47 | -1.51 | -0.81 | -0.99 | 0.77 | 0.77 | 1.49 | 1.53 | 0.83 | 0.99 |
| Ord.0.40Load.Cross250.4Var.300Size.4Fac.0.00Fcor | 13.98 | 13.99 | 14.17 | 14.12 | -3.40 | -3.41 | -3.46 | -3.44 | -2.05 | -0.26 | 3.42 | 3.41 | 3.48 | 3.46 | 2.17 | 0.32 |
| Ord.0.40Load.Cross250.4Var.300Size.4Fac.0.50Fcor | 6.47 | 6.54 | 5.56 | 7.13 | -1.64 | -1.64 | -1.82 | -1.48 | -1.09 | -2.53 | 1.80 | 1.80 | 1.90 | 1.68 | 1.23 | 2.53 |
| Ord.0.40Load.Cross250.4Var.300Size.4Fac.0.70Fcor | 4.87 | 4.87 | 5.99 | 7.30 | -1.73 | -1.73 | -1.96 | -1.68 | -1.21 | -2.98 | 1.89 | 1.89 | 2.02 | 1.84 | 1.33 | 2.98 |
| Ord.0.40Load.Cross250.4Var.500Size.4Fac.0.00Fcor | 6.50 | 6.51 | 7.04 | 7.13 | -1.34 | -1.36 | -1.66 | -1.40 | -0.29 | -0.07 | 1.52 | 1.54 | 1.72 | 1.58 | 0.59 | 0.09 |
| Ord.0.40Load.Cross250.4Var.500Size.4Fac.0.50Fcor | 4.91 | 5.03 | 4.81 | 6.35 | -1.53 | -1.55 | -1.79 | -1.46 | -0.99 | -2.39 | 1.71 | 1.73 | 1.89 | 1.64 | 1.11 | 2.39 |
| Ord.0.40Load.Cross250.4Var.500Size.4Fac.0.70Fcor | 4.76 | 4.76 | 7.65 | 8.33 | -2.08 | -2.08 | -2.25 | -2.23 | -1.35 | -2.98 | 2.14 | 2.14 | 2.31 | 2.29 | 1.39 | 2.98 |
| Ord.0.40Load.Cross250.4Var.1000Size.4Fac.0.00Fcor | 1.71 | 1.70 | 2.76 | 2.49 | -0.38 | -0.38 | -0.93 | -0.34 | -0.12 | 0.00 | 0.68 | 0.68 | 1.07 | 0.72 | 0.12 | 0.00 |
| Ord.0.40Load.Cross250.4Var.1000Size.4Fac.0.50Fcor | 3.97 | 3.98 | 4.07 | 5.65 | -1.73 | -1.73 | -1.85 | -1.69 | -0.86 | -1.91 | 1.73 | 1.73 | 1.85 | 1.69 | 0.94 | 1.91 |
| Ord.0.40Load.Cross250.4Var.1000Size.4Fac.0.70Fcor | 3.25 | 3.25 | 8.91 | 9.45 | -2.47 | -2.47 | -2.64 | -2.60 | -1.53 | -2.96 | 2.47 | 2.47 | 2.64 | 2.60 | 1.53 | 2.96 |
| Ord.0.40Load.Cross250.8Var.300Size.2Fac.0.00Fcor | 7.76 | 7.79 | 8.38 | 9.04 | -0.37 | -0.39 | -0.42 | -0.13 | 0.04 | 0.00 | 1.19 | 1.17 | 1.26 | 1.55 | 0.96 | 0.00 |
| Ord.0.40Load.Cross250.8Var.300Size.2Fac.0.50Fcor | 5.05 | 5.04 | 4.60 | 5.73 | 0.40 | 0.43 | 0.26 | 0.71 | 0.55 | -0.29 | 0.88 | 0.91 | 0.66 | 1.11 | 0.65 | 0.29 |
| Ord.0.40Load.Cross250.8Var.300Size.2Fac.0.70Fcor | 5.44 | 5.44 | 4.43 | 5.96 | 0.43 | 0.43 | 0.19 | 0.68 | 0.53 | -0.87 | 0.87 | 0.87 | 0.63 | 1.16 | 0.67 | 0.87 |
| Ord.0.40Load.Cross250.8Var.500Size.2Fac.0.00Fcor | 1.33 | 1.32 | 3.20 | 3.53 | 0.28 | 0.28 | 0.48 | 0.95 | 0.22 | 0.00 | 0.40 | 0.40 | 0.60 | 1.07 | 0.24 | 0.00 |
| Ord.0.40Load.Cross250.8Var.500Size.2Fac.0.50Fcor | 3.16 | 3.16 | 3.83 | 5.18 | 0.29 | 0.29 | 0.33 | 0.59 | 0.15 | -0.06 | 0.59 | 0.59 | 0.53 | 0.81 | 0.15 | 0.06 |
| Ord.0.40Load.Cross250.8Var.500Size.2Fac.0.70Fcor | 4.01 | 4.01 | 4.84 | 6.01 | 0.16 | 0.16 | 0.19 | 0.26 | 0.39 | -0.76 | 0.62 | 0.62 | 0.59 | 0.72 | 0.41 | 0.76 |
| Ord.0.40Load.Cross250.8Var.1000Size.2Fac.0.00Fcor | 0.36 | 0.37 | 2.60 | 3.33 | 0.09 | 0.11 | 0.67 | 0.94 | 0.24 | 0.00 | 0.09 | 0.11 | 0.67 | 0.96 | 0.24 | 0.00 |
| Ord.0.40Load.Cross250.8Var.1000Size.2Fac.0.50Fcor | 1.48 | 1.48 | 2.90 | 3.85 | 0.14 | 0.14 | 0.51 | 0.62 | 0.02 | 0.00 | 0.36 | 0.36 | 0.55 | 0.72 | 0.02 | 0.00 |
| Ord.0.40Load.Cross250.8Var.1000Size.2Fac.0.70Fcor | 2.93 | 2.93 | 4.29 | 5.57 | -0.05 | -0.05 | 0.08 | 0.12 | 0.07 | -0.55 | 0.51 | 0.51 | 0.48 | 0.66 | 0.09 | 0.55 |
| Ord.0.40Load.Cross250.8Var.300Size.4Fac.0.00Fcor | 29.21 | 29.21 | 29.44 | 29.48 | -3.36 | -3.36 | -3.45 | -3.33 | -2.02 | 0.02 | 3.58 | 3.58 | 3.59 | 3.63 | 2.72 | 0.02 |
| Ord.0.40Load.Cross250.8Var.300Size.4Fac.0.50Fcor | 14.07 | 14.07 | 13.24 | 14.53 | 0.01 | 0.01 | -0.89 | 0.22 | 0.19 | -1.38 | 1.73 | 1.73 | 1.69 | 1.64 | 0.97 | 1.38 |
| Ord.0.40Load.Cross250.8Var.300Size.4Fac.0.70Fcor | 13.91 | 13.91 | 12.08 | 13.29 | -0.33 | -0.33 | -0.74 | 0.13 | 0.20 | -2.90 | 1.61 | 1.61 | 1.40 | 1.45 | 0.96 | 2.90 |
| Ord.0.40Load.Cross250.8Var.500Size.4Fac.0.00Fcor | 7.89 | 7.94 | 10.89 | 9.34 | 0.64 | 0.65 | -0.10 | 1.27 | -0.08 | 0.00 | 1.76 | 1.79 | 1.66 | 2.17 | 0.60 | 0.00 |
| Ord.0.40Load.Cross250.8Var.500Size.4Fac.0.50Fcor | 10.20 | 10.20 | 8.22 | 10.04 | 0.53 | 0.46 | -0.22 | 1.33 | 0.03 | -0.67 | 1.57 | 1.54 | 1.46 | 1.75 | 0.55 | 0.67 |
| Ord.0.40Load.Cross250.8Var.500Size.4Fac.0.70Fcor | 13.27 | 13.27 | 10.88 | 12.23 | -0.17 | -0.17 | -0.96 | 0.27 | -0.25 | -2.94 | 1.59 | 1.59 | 1.66 | 1.59 | 0.95 | 2.94 |
| Ord.0.40Load.Cross250.8Var.1000Size.4Fac.0.00Fcor | 1.15 | 1.15 | 4.86 | 4.00 | 0.41 | 0.41 | -0.79 | 1.33 | 0.10 | 0.00 | 0.53 | 0.53 | 1.29 | 1.35 | 0.10 | 0.00 |
| Ord.0.40Load.Cross250.8Var.1000Size.4Fac.0.50Fcor | 11.74 | 11.74 | 9.03 | 12.25 | -0.64 | -0.64 | -1.24 | -0.16 | 0.10 | -0.01 | 1.16 | 1.16 | 1.46 | 1.08 | 0.58 | 0.01 |
| Ord.0.40Load.Cross250.8Var.1000Size.4Fac.0.70Fcor | 12.11 | 12.11 | 12.47 | 14.48 | -1.27 | -1.27 | -1.46 | -0.85 | -0.37 | -2.47 | 1.71 | 1.71 | 1.76 | 1.45 | 0.87 | 2.47 |
| Ord.0.55Load.000Cross.4Var.300Size.2Fac.0.00Fcor | 0.00 | 0.00 | 0.50 | 1.21 | 0.00 | 0.00 | 0.07 | -0.08 | 0.00 | 0.00 | 0.00 | 0.00 | 0.07 | 0.24 | 0.00 | 0.00 |
| Ord.0.55Load.000Cross.4Var.300Size.2Fac.0.50Fcor | 0.17 | 0.17 | 0.44 | 1.29 | 0.06 | 0.06 | 0.02 | 0.02 | -0.01 | -0.27 | 0.12 | 0.12 | 0.10 | 0.40 | 0.01 | 0.27 |
| Ord.0.55Load.000Cross.4Var.300Size.2Fac.0.70Fcor | 0.57 | 0.57 | 1.65 | 2.29 | 0.00 | 0.00 | -0.12 | -0.22 | -0.32 | -0.88 | 0.36 | 0.36 | 0.48 | 0.64 | 0.34 | 0.88 |
| Ord.0.55Load.000Cross.4Var.500Size.2Fac.0.00Fcor | 0.00 | 0.00 | 0.36 | 0.90 | 0.00 | 0.00 | 0.07 | -0.05 | 0.00 | 0.00 | 0.00 | 0.00 | 0.07 | 0.19 | 0.00 | 0.00 |
| Ord.0.55Load.000Cross.4Var.500Size.2Fac.0.50Fcor | 0.02 | 0.02 | 0.31 | 0.99 | 0.04 | 0.04 | 0.09 | 0.03 | 0.00 | -0.05 | 0.04 | 0.04 | 0.09 | 0.23 | 0.00 | 0.05 |
| Ord.0.55Load.000Cross.4Var.500Size.2Fac.0.70Fcor | 0.38 | 0.38 | 0.66 | 1.43 | 0.06 | 0.06 | 0.06 | -0.05 | -0.19 | -0.83 | 0.14 | 0.14 | 0.22 | 0.39 | 0.19 | 0.83 |
| Ord.0.55Load.000Cross.4Var.1000Size.2Fac.0.00Fcor | 0.00 | 0.00 | 0.22 | 0.68 | 0.00 | 0.00 | 0.09 | 0.01 | 0.00 | 0.00 | 0.00 | 0.00 | 0.09 | 0.21 | 0.00 | 0.00 |
| Ord.0.55Load.000Cross.4Var.1000Size.2Fac.0.50Fcor | 0.01 | 0.01 | 0.19 | 0.60 | 0.00 | 0.00 | 0.11 | 0.05 | 0.00 | -0.01 | 0.00 | 0.00 | 0.11 | 0.19 | 0.00 | 0.01 |
| Ord.0.55Load.000Cross.4Var.1000Size.2Fac.0.70Fcor | 0.31 | 0.31 | 0.88 | 1.55 | -0.04 | -0.04 | -0.10 | -0.22 | -0.19 | -0.75 | 0.16 | 0.16 | 0.22 | 0.36 | 0.19 | 0.75 |
| Ord.0.55Load.000Cross.4Var.300Size.4Fac.0.00Fcor | 0.06 | 0.06 | 3.90 | 1.01 | 0.01 | 0.01 | -0.60 | 0.20 | 0.00 | 0.00 | 0.01 | 0.01 | 0.82 | 0.30 | 0.00 | 0.00 |
| Ord.0.55Load.000Cross.4Var.300Size.4Fac.0.50Fcor | 2.21 | 2.21 | 1.99 | 3.25 | -0.39 | -0.39 | -1.23 | -0.26 | -0.07 | -1.07 | 0.63 | 0.63 | 1.41 | 0.66 | 0.13 | 1.07 |
| Ord.0.55Load.000Cross.4Var.300Size.4Fac.0.70Fcor | 3.48 | 3.48 | 1.86 | 3.43 | -0.67 | -0.67 | -1.36 | -0.35 | -0.70 | -2.94 | 1.07 | 1.07 | 1.54 | 0.87 | 0.78 | 2.94 |
| Ord.0.55Load.000Cross.4Var.500Size.4Fac.0.00Fcor | 0.00 | 0.00 | 2.62 | 0.70 | 0.00 | 0.00 | -0.51 | 0.04 | 0.00 | 0.00 | 0.00 | 0.00 | 0.59 | 0.12 | 0.00 | 0.00 |
| Ord.0.55Load.000Cross.4Var.500Size.4Fac.0.50Fcor | 0.75 | 0.75 | 1.37 | 1.47 | -0.12 | -0.12 | -1.25 | 0.14 | -0.06 | -0.53 | 0.30 | 0.30 | 1.37 | 0.44 | 0.06 | 0.53 |
| Ord.0.55Load.000Cross.4Var.500Size.4Fac.0.70Fcor | 2.56 | 2.62 | 1.58 | 2.86 | -0.49 | -0.47 | -1.30 | -0.21 | -0.61 | -2.85 | 0.83 | 0.81 | 1.40 | 0.81 | 0.65 | 2.85 |
| Ord.0.55Load.000Cross.4Var.1000Size.4Fac.0.00Fcor | 0.00 | 0.00 | 1.80 | 0.77 | 0.00 | 0.00 | -0.53 | 0.00 | 0.00 | 0.00 | 0.00 | 0.00 | 0.55 | 0.12 | 0.00 | 0.00 |
| Ord.0.55Load.000Cross.4Var.1000Size.4Fac.0.50Fcor | 0.26 | 0.26 | 0.75 | 0.98 | -0.01 | -0.01 | -1.25 | 0.07 | -0.04 | -0.13 | 0.07 | 0.07 | 1.27 | 0.15 | 0.04 | 0.13 |
| Ord.0.55Load.000Cross.4Var.1000Size.4Fac.0.70Fcor | 2.04 | 2.04 | 0.92 | 2.55 | -0.47 | -0.47 | -1.46 | -0.43 | -0.24 | -2.67 | 0.59 | 0.59 | 1.48 | 0.63 | 0.24 | 2.67 |
| Ord.0.55Load.000Cross.8Var.300Size.2Fac.0.00Fcor | 0.00 | 0.00 | 2.90 | 3.03 | 0.00 | 0.00 | 0.97 | 1.30 | 0.00 | 0.00 | 0.00 | 0.00 | 0.97 | 1.30 | 0.00 | 0.00 |
| Ord.0.55Load.000Cross.8Var.300Size.2Fac.0.50Fcor | 0.22 | 0.22 | 2.22 | 3.02 | 0.10 | 0.10 | 0.74 | 1.41 | 0.00 | 0.00 | 0.10 | 0.10 | 0.74 | 1.41 | 0.00 | 0.00 |
| Ord.0.55Load.000Cross.8Var.300Size.2Fac.0.70Fcor | 0.86 | 0.88 | 1.92 | 2.79 | 0.69 | 0.68 | 0.79 | 1.39 | 0.02 | -0.26 | 0.69 | 0.68 | 0.79 | 1.39 | 0.02 | 0.26 |
| Ord.0.55Load.000Cross.8Var.500Size.2Fac.0.00Fcor | 0.00 | 0.00 | 2.57 | 3.19 | 0.00 | 0.00 | 0.71 | 0.97 | 0.00 | 0.00 | 0.00 | 0.00 | 0.71 | 0.97 | 0.00 | 0.00 |
| Ord.0.55Load.000Cross.8Var.500Size.2Fac.0.50Fcor | 0.03 | 0.03 | 2.17 | 3.23 | 0.03 | 0.03 | 0.52 | 1.03 | 0.00 | 0.00 | 0.03 | 0.03 | 0.52 | 1.03 | 0.00 | 0.00 |
| Ord.0.55Load.000Cross.8Var.500Size.2Fac.0.70Fcor | 0.44 | 0.44 | 2.20 | 3.12 | 0.24 | 0.24 | 0.67 | 1.18 | 0.00 | -0.04 | 0.24 | 0.24 | 0.67 | 1.20 | 0.00 | 0.04 |
| Ord.0.55Load.000Cross.8Var.1000Size.2Fac.0.00Fcor | 0.00 | 0.00 | 1.82 | 3.12 | 0.00 | 0.00 | 0.67 | 0.82 | 0.00 | 0.00 | 0.00 | 0.00 | 0.67 | 0.82 | 0.00 | 0.00 |
| Ord.0.55Load.000Cross.8Var.1000Size.2Fac.0.50Fcor | 0.00 | 0.00 | 2.00 | 3.04 | 0.00 | 0.00 | 0.35 | 0.70 | 0.00 | 0.00 | 0.00 | 0.00 | 0.35 | 0.70 | 0.00 | 0.00 |
| Ord.0.55Load.000Cross.8Var.1000Size.2Fac.0.70Fcor | 0.06 | 0.06 | 1.81 | 3.61 | 0.16 | 0.16 | 0.40 | 0.66 | 0.00 | -0.01 | 0.16 | 0.16 | 0.40 | 0.66 | 0.00 | 0.01 |
| Ord.0.55Load.000Cross.8Var.300Size.4Fac.0.00Fcor | 0.00 | 0.01 | 9.70 | 5.16 | 0.02 | 0.01 | 0.24 | 1.84 | 0.00 | 0.00 | 0.02 | 0.01 | 1.40 | 1.84 | 0.00 | 0.00 |
| Ord.0.55Load.000Cross.8Var.300Size.4Fac.0.50Fcor | 1.95 | 1.95 | 2.94 | 5.08 | 0.52 | 0.52 | -0.71 | 2.15 | 0.01 | -0.03 | 0.86 | 0.86 | 1.37 | 2.21 | 0.01 | 0.03 |
| Ord.0.55Load.000Cross.8Var.300Size.4Fac.0.70Fcor | 4.27 | 4.22 | 2.02 | 4.49 | 2.01 | 1.93 | -0.16 | 3.02 | -0.07 | -1.95 | 2.19 | 2.15 | 1.58 | 3.06 | 0.19 | 1.95 |
| Ord.0.55Load.000Cross.8Var.500Size.4Fac.0.00Fcor | 0.00 | 0.00 | 8.22 | 3.50 | 0.00 | 0.00 | 0.25 | 1.98 | 0.00 | 0.00 | 0.00 | 0.00 | 1.41 | 1.98 | 0.00 | 0.00 |
| Ord.0.55Load.000Cross.8Var.500Size.4Fac.0.50Fcor | 0.65 | 0.65 | 2.12 | 4.08 | 0.24 | 0.24 | -0.99 | 1.81 | 0.00 | 0.00 | 0.26 | 0.26 | 1.27 | 1.81 | 0.00 | 0.00 |
| Ord.0.55Load.000Cross.8Var.500Size.4Fac.0.70Fcor | 2.43 | 2.43 | 2.97 | 4.80 | 1.01 | 1.01 | -0.51 | 1.94 | 0.00 | -0.86 | 1.19 | 1.19 | 1.31 | 1.96 | 0.02 | 0.86 |
| Ord.0.55Load.000Cross.8Var.1000Size.4Fac.0.00Fcor | 0.00 | 0.00 | 5.46 | 3.63 | 0.00 | 0.00 | -0.35 | 0.95 | 0.00 | 0.00 | 0.00 | 0.00 | 0.95 | 0.95 | 0.00 | 0.00 |
| Ord.0.55Load.000Cross.8Var.1000Size.4Fac.0.50Fcor | 0.01 | 0.01 | 2.08 | 2.99 | 0.01 | 0.01 | -1.00 | 1.12 | 0.00 | 0.00 | 0.01 | 0.01 | 1.18 | 1.14 | 0.00 | 0.00 |
| Ord.0.55Load.000Cross.8Var.1000Size.4Fac.0.70Fcor | 0.49 | 0.49 | 1.23 | 4.45 | 0.21 | 0.21 | -1.09 | 1.18 | 0.00 | -0.07 | 0.21 | 0.21 | 1.37 | 1.22 | 0.00 | 0.07 |
| Ord.0.55Load.125Cross.4Var.300Size.2Fac.0.00Fcor | 0.06 | 0.06 | 0.29 | 1.33 | 0.04 | 0.05 | 0.07 | 0.02 | 0.00 | 0.00 | 0.06 | 0.07 | 0.11 | 0.32 | 0.00 | 0.00 |
| Ord.0.55Load.125Cross.4Var.300Size.2Fac.0.50Fcor | 0.37 | 0.37 | 0.85 | 2.12 | 0.02 | 0.02 | -0.01 | -0.14 | -0.03 | -0.37 | 0.16 | 0.16 | 0.23 | 0.50 | 0.05 | 0.37 |
| Ord.0.55Load.125Cross.4Var.300Size.2Fac.0.70Fcor | 0.86 | 0.88 | 1.81 | 2.53 | -0.13 | -0.14 | -0.14 | -0.23 | -0.56 | -0.98 | 0.33 | 0.34 | 0.42 | 0.59 | 0.58 | 0.98 |
| Ord.0.55Load.125Cross.4Var.500Size.2Fac.0.00Fcor | 0.01 | 0.02 | 0.24 | 1.18 | 0.00 | 0.01 | 0.08 | 0.00 | 0.00 | 0.00 | 0.00 | 0.01 | 0.08 | 0.22 | 0.00 | 0.00 |
| Ord.0.55Load.125Cross.4Var.500Size.2Fac.0.50Fcor | 0.23 | 0.23 | 0.56 | 1.50 | -0.01 | -0.01 | 0.03 | 0.01 | -0.01 | -0.19 | 0.09 | 0.09 | 0.19 | 0.41 | 0.01 | 0.19 |
| Ord.0.55Load.125Cross.4Var.500Size.2Fac.0.70Fcor | 0.69 | 0.69 | 1.93 | 2.76 | -0.20 | -0.20 | -0.33 | -0.45 | -0.64 | -0.95 | 0.28 | 0.28 | 0.39 | 0.57 | 0.64 | 0.95 |
| Ord.0.55Load.125Cross.4Var.1000Size.2Fac.0.00Fcor | 0.00 | 0.01 | 0.26 | 0.97 | 0.00 | 0.00 | 0.01 | -0.04 | 0.00 | 0.00 | 0.00 | 0.00 | 0.01 | 0.08 | 0.00 | 0.00 |
| Ord.0.55Load.125Cross.4Var.1000Size.2Fac.0.50Fcor | 0.16 | 0.16 | 0.41 | 1.72 | -0.01 | -0.01 | 0.03 | -0.17 | -0.02 | -0.10 | 0.01 | 0.01 | 0.07 | 0.31 | 0.02 | 0.10 |
| Ord.0.55Load.125Cross.4Var.1000Size.2Fac.0.70Fcor | 0.69 | 0.69 | 1.89 | 2.73 | -0.20 | -0.20 | -0.32 | -0.42 | -0.83 | -0.95 | 0.22 | 0.22 | 0.36 | 0.52 | 0.83 | 0.95 |
| Ord.0.55Load.125Cross.4Var.300Size.4Fac.0.00Fcor | 0.26 | 0.28 | 2.82 | 1.47 | 0.00 | 0.00 | -0.79 | 0.09 | 0.00 | 0.00 | 0.12 | 0.12 | 0.93 | 0.29 | 0.00 | 0.00 |
| Ord.0.55Load.125Cross.4Var.300Size.4Fac.0.50Fcor | 1.84 | 1.84 | 1.90 | 3.20 | -0.64 | -0.64 | -1.36 | -0.39 | -0.33 | -1.41 | 0.82 | 0.82 | 1.38 | 0.77 | 0.39 | 1.41 |
| Ord.0.55Load.125Cross.4Var.300Size.4Fac.0.70Fcor | 3.09 | 3.09 | 2.50 | 3.82 | -1.03 | -1.03 | -1.45 | -0.77 | -0.99 | -2.95 | 1.25 | 1.25 | 1.51 | 1.01 | 1.03 | 2.95 |
| Ord.0.55Load.125Cross.4Var.500Size.4Fac.0.00Fcor | 0.01 | 0.01 | 1.30 | 1.17 | 0.00 | 0.00 | -0.90 | 0.09 | -0.01 | 0.00 | 0.00 | 0.00 | 0.98 | 0.21 | 0.01 | 0.00 |
| Ord.0.55Load.125Cross.4Var.500Size.4Fac.0.50Fcor | 1.13 | 1.13 | 0.96 | 2.03 | -0.42 | -0.42 | -1.49 | -0.06 | -0.38 | -1.07 | 0.70 | 0.70 | 1.55 | 0.58 | 0.42 | 1.07 |
| Ord.0.55Load.125Cross.4Var.500Size.4Fac.0.70Fcor | 3.23 | 3.23 | 1.99 | 4.15 | -1.14 | -1.14 | -1.53 | -0.90 | -0.98 | -2.93 | 1.22 | 1.22 | 1.57 | 1.08 | 1.00 | 2.93 |
| Ord.0.55Load.125Cross.4Var.1000Size.4Fac.0.00Fcor | 0.00 | 0.00 | 1.22 | 1.01 | 0.00 | 0.00 | -1.03 | 0.05 | -0.01 | 0.00 | 0.00 | 0.00 | 1.09 | 0.09 | 0.01 | 0.00 |
| Ord.0.55Load.125Cross.4Var.1000Size.4Fac.0.50Fcor | 0.34 | 0.34 | 0.59 | 1.58 | -0.16 | -0.16 | -1.56 | -0.01 | -0.19 | -0.45 | 0.24 | 0.24 | 1.56 | 0.37 | 0.25 | 0.45 |
| Ord.0.55Load.125Cross.4Var.1000Size.4Fac.0.70Fcor | 2.10 | 2.10 | 1.75 | 3.49 | -1.07 | -1.07 | -1.54 | -0.90 | -0.76 | -2.86 | 1.15 | 1.15 | 1.56 | 1.02 | 0.80 | 2.86 |
| Ord.0.55Load.125Cross.8Var.300Size.2Fac.0.00Fcor | 0.16 | 0.17 | 2.67 | 3.24 | 0.03 | 0.04 | 0.72 | 1.24 | 0.25 | 0.00 | 0.03 | 0.04 | 0.72 | 1.24 | 0.25 | 0.00 |
| Ord.0.55Load.125Cross.8Var.300Size.2Fac.0.50Fcor | 0.58 | 0.58 | 1.79 | 3.48 | 0.16 | 0.16 | 0.90 | 1.43 | 0.06 | 0.00 | 0.20 | 0.20 | 0.90 | 1.43 | 0.06 | 0.00 |
| Ord.0.55Load.125Cross.8Var.300Size.2Fac.0.70Fcor | 1.90 | 1.90 | 2.42 | 3.60 | 0.41 | 0.41 | 0.83 | 1.30 | 0.06 | -0.52 | 0.55 | 0.55 | 0.83 | 1.36 | 0.08 | 0.52 |
| Ord.0.55Load.125Cross.8Var.500Size.2Fac.0.00Fcor | 0.13 | 0.21 | 2.76 | 3.19 | 0.01 | 0.01 | 0.46 | 0.87 | 0.22 | 0.00 | 0.01 | 0.01 | 0.46 | 0.87 | 0.22 | 0.00 |
| Ord.0.55Load.125Cross.8Var.500Size.2Fac.0.50Fcor | 0.12 | 0.12 | 2.17 | 3.38 | 0.04 | 0.04 | 0.76 | 1.14 | 0.04 | 0.00 | 0.04 | 0.04 | 0.76 | 1.14 | 0.04 | 0.00 |
| Ord.0.55Load.125Cross.8Var.500Size.2Fac.0.70Fcor | 1.07 | 1.07 | 2.70 | 3.64 | 0.18 | 0.18 | 0.62 | 0.91 | 0.04 | -0.31 | 0.28 | 0.28 | 0.62 | 0.91 | 0.04 | 0.31 |
| Ord.0.55Load.125Cross.8Var.1000Size.2Fac.0.00Fcor | 0.09 | 0.17 | 2.20 | 3.19 | 0.00 | 0.00 | 0.46 | 0.83 | 0.21 | 0.00 | 0.00 | 0.00 | 0.46 | 0.83 | 0.21 | 0.00 |
| Ord.0.55Load.125Cross.8Var.1000Size.2Fac.0.50Fcor | 0.01 | 0.01 | 1.93 | 3.21 | 0.00 | 0.00 | 0.60 | 0.79 | 0.01 | 0.00 | 0.00 | 0.00 | 0.60 | 0.79 | 0.01 | 0.00 |
| Ord.0.55Load.125Cross.8Var.1000Size.2Fac.0.70Fcor | 0.39 | 0.39 | 2.81 | 3.72 | 0.03 | 0.03 | 0.25 | 0.37 | 0.00 | -0.04 | 0.05 | 0.05 | 0.25 | 0.43 | 0.00 | 0.04 |
| Ord.0.55Load.125Cross.8Var.300Size.4Fac.0.00Fcor | 0.12 | 0.13 | 7.83 | 5.23 | 0.03 | 0.03 | -0.55 | 1.74 | 0.00 | 0.00 | 0.03 | 0.03 | 1.37 | 1.74 | 0.00 | 0.00 |
| Ord.0.55Load.125Cross.8Var.300Size.4Fac.0.50Fcor | 2.28 | 2.28 | 3.08 | 5.33 | 0.57 | 0.57 | -0.76 | 2.56 | 0.07 | -0.05 | 0.87 | 0.87 | 1.42 | 2.62 | 0.09 | 0.05 |
| Ord.0.55Load.125Cross.8Var.300Size.4Fac.0.70Fcor | 5.84 | 5.92 | 3.56 | 6.81 | 1.02 | 0.99 | -0.08 | 2.26 | -0.09 | -2.25 | 1.48 | 1.45 | 1.26 | 2.38 | 0.37 | 2.25 |
| Ord.0.55Load.125Cross.8Var.500Size.4Fac.0.00Fcor | 0.01 | 0.01 | 4.65 | 4.32 | 0.00 | 0.00 | -0.76 | 1.45 | 0.00 | 0.00 | 0.00 | 0.00 | 1.16 | 1.45 | 0.00 | 0.00 |
| Ord.0.55Load.125Cross.8Var.500Size.4Fac.0.50Fcor | 1.03 | 1.03 | 1.96 | 4.39 | 0.26 | 0.26 | -0.79 | 1.97 | 0.06 | 0.00 | 0.48 | 0.48 | 1.41 | 1.99 | 0.06 | 0.00 |
| Ord.0.55Load.125Cross.8Var.500Size.4Fac.0.70Fcor | 3.99 | 3.99 | 2.60 | 5.68 | 0.75 | 0.75 | -0.54 | 2.02 | 0.02 | -1.46 | 1.29 | 1.29 | 1.54 | 2.10 | 0.12 | 1.46 |
| Ord.0.55Load.125Cross.8Var.1000Size.4Fac.0.00Fcor | 0.00 | 0.00 | 3.22 | 4.84 | 0.00 | 0.00 | -0.55 | 1.05 | 0.00 | 0.00 | 0.00 | 0.00 | 1.03 | 1.05 | 0.00 | 0.00 |
| Ord.0.55Load.125Cross.8Var.1000Size.4Fac.0.50Fcor | 0.04 | 0.04 | 3.20 | 4.10 | 0.00 | 0.00 | -1.16 | 1.21 | 0.06 | 0.00 | 0.02 | 0.02 | 1.50 | 1.21 | 0.06 | 0.00 |
| Ord.0.55Load.125Cross.8Var.1000Size.4Fac.0.70Fcor | 1.77 | 1.77 | 3.21 | 4.72 | 0.11 | 0.11 | -0.79 | 1.11 | 0.00 | -0.29 | 0.39 | 0.39 | 1.37 | 1.15 | 0.02 | 0.29 |
| Ord.0.55Load.Cross250.4Var.300Size.2Fac.0.00Fcor | 0.28 | 0.30 | 0.47 | 1.65 | 0.07 | 0.06 | 0.13 | 0.08 | 0.18 | 0.00 | 0.15 | 0.16 | 0.21 | 0.48 | 0.18 | 0.00 |
| Ord.0.55Load.Cross250.4Var.300Size.2Fac.0.50Fcor | 0.86 | 0.86 | 2.21 | 3.22 | -0.24 | -0.24 | -0.39 | -0.53 | -0.21 | -0.70 | 0.30 | 0.30 | 0.49 | 0.69 | 0.27 | 0.70 |
| Ord.0.55Load.Cross250.4Var.300Size.2Fac.0.70Fcor | 0.71 | 0.71 | 3.48 | 4.25 | -0.47 | -0.47 | -0.74 | -0.91 | -0.78 | -1.00 | 0.51 | 0.51 | 0.78 | 0.95 | 0.82 | 1.00 |
| Ord.0.55Load.Cross250.4Var.500Size.2Fac.0.00Fcor | 0.31 | 0.35 | 0.64 | 1.18 | 0.06 | 0.06 | 0.13 | 0.13 | 0.13 | 0.00 | 0.12 | 0.12 | 0.21 | 0.31 | 0.13 | 0.00 |
| Ord.0.55Load.Cross250.4Var.500Size.2Fac.0.50Fcor | 0.34 | 0.34 | 0.99 | 2.11 | -0.07 | -0.07 | -0.05 | -0.29 | -0.18 | -0.47 | 0.15 | 0.15 | 0.27 | 0.53 | 0.18 | 0.47 |
| Ord.0.55Load.Cross250.4Var.500Size.2Fac.0.70Fcor | 0.67 | 0.67 | 3.46 | 4.04 | -0.43 | -0.43 | -0.80 | -0.93 | -0.93 | -0.99 | 0.43 | 0.43 | 0.80 | 0.93 | 0.93 | 0.99 |
| Ord.0.55Load.Cross250.4Var.1000Size.2Fac.0.00Fcor | 0.03 | 0.13 | 0.46 | 1.17 | 0.03 | 0.05 | 0.19 | 0.13 | 0.06 | 0.00 | 0.03 | 0.05 | 0.19 | 0.19 | 0.06 | 0.00 |
| Ord.0.55Load.Cross250.4Var.1000Size.2Fac.0.50Fcor | 0.19 | 0.19 | 0.80 | 1.89 | -0.06 | -0.06 | -0.07 | -0.31 | -0.24 | -0.37 | 0.10 | 0.10 | 0.17 | 0.43 | 0.24 | 0.37 |
| Ord.0.55Load.Cross250.4Var.1000Size.2Fac.0.70Fcor | 0.47 | 0.47 | 2.50 | 3.45 | -0.30 | -0.30 | -0.58 | -0.82 | -0.97 | -1.00 | 0.30 | 0.30 | 0.58 | 0.82 | 0.97 | 1.00 |
| Ord.0.55Load.Cross250.4Var.300Size.4Fac.0.00Fcor | 0.68 | 0.68 | 2.53 | 1.84 | -0.03 | -0.05 | -0.76 | 0.09 | -0.05 | 0.00 | 0.23 | 0.25 | 0.90 | 0.37 | 0.09 | 0.00 |
| Ord.0.55Load.Cross250.4Var.300Size.4Fac.0.50Fcor | 1.63 | 1.63 | 1.40 | 3.01 | -0.62 | -0.62 | -1.44 | -0.33 | -0.53 | -1.79 | 0.90 | 0.90 | 1.52 | 0.83 | 0.63 | 1.79 |
| Ord.0.55Load.Cross250.4Var.300Size.4Fac.0.70Fcor | 3.53 | 3.53 | 2.24 | 4.72 | -1.35 | -1.35 | -1.58 | -1.06 | -1.30 | -2.99 | 1.45 | 1.45 | 1.62 | 1.20 | 1.36 | 2.99 |
| Ord.0.55Load.Cross250.4Var.500Size.4Fac.0.00Fcor | 0.09 | 0.11 | 1.38 | 1.12 | -0.05 | -0.05 | -0.93 | 0.13 | -0.02 | 0.00 | 0.05 | 0.05 | 0.95 | 0.19 | 0.02 | 0.00 |
| Ord.0.55Load.Cross250.4Var.500Size.4Fac.0.50Fcor | 1.25 | 1.28 | 1.01 | 2.52 | -0.54 | -0.53 | -1.47 | -0.16 | -0.42 | -1.50 | 0.66 | 0.65 | 1.49 | 0.62 | 0.50 | 1.50 |
| Ord.0.55Load.Cross250.4Var.500Size.4Fac.0.70Fcor | 3.54 | 3.54 | 2.39 | 4.29 | -1.21 | -1.21 | -1.62 | -0.99 | -1.36 | -2.99 | 1.31 | 1.31 | 1.68 | 1.13 | 1.46 | 2.99 |
| Ord.0.55Load.Cross250.4Var.1000Size.4Fac.0.00Fcor | 0.00 | 0.00 | 0.78 | 1.05 | 0.00 | 0.00 | -1.02 | 0.12 | -0.02 | 0.00 | 0.00 | 0.00 | 1.08 | 0.12 | 0.02 | 0.00 |
| Ord.0.55Load.Cross250.4Var.1000Size.4Fac.0.50Fcor | 0.62 | 0.62 | 0.78 | 1.28 | -0.22 | -0.22 | -1.46 | 0.07 | -0.52 | -0.69 | 0.34 | 0.34 | 1.46 | 0.35 | 0.60 | 0.69 |
| Ord.0.55Load.Cross250.4Var.1000Size.4Fac.0.70Fcor | 2.67 | 2.67 | 2.79 | 4.17 | -1.32 | -1.32 | -1.64 | -1.23 | -1.77 | -2.94 | 1.38 | 1.38 | 1.66 | 1.29 | 1.79 | 2.94 |
| Ord.0.55Load.Cross250.8Var.300Size.2Fac.0.00Fcor | 0.16 | 0.17 | 2.67 | 3.24 | 0.03 | 0.04 | 0.72 | 1.24 | 0.25 | 0.00 | 0.03 | 0.04 | 0.72 | 1.24 | 0.25 | 0.00 |
| Ord.0.55Load.Cross250.8Var.300Size.2Fac.0.50Fcor | 0.58 | 0.58 | 1.79 | 3.48 | 0.16 | 0.16 | 0.90 | 1.43 | 0.06 | 0.00 | 0.20 | 0.20 | 0.90 | 1.43 | 0.06 | 0.00 |
| Ord.0.55Load.Cross250.8Var.300Size.2Fac.0.70Fcor | 1.90 | 1.90 | 2.42 | 3.60 | 0.41 | 0.41 | 0.83 | 1.30 | 0.06 | -0.52 | 0.55 | 0.55 | 0.83 | 1.36 | 0.08 | 0.52 |
| Ord.0.55Load.Cross250.8Var.500Size.2Fac.0.00Fcor | 0.13 | 0.21 | 2.76 | 3.19 | 0.01 | 0.01 | 0.46 | 0.87 | 0.22 | 0.00 | 0.01 | 0.01 | 0.46 | 0.87 | 0.22 | 0.00 |
| Ord.0.55Load.Cross250.8Var.500Size.2Fac.0.50Fcor | 0.12 | 0.12 | 2.17 | 3.38 | 0.04 | 0.04 | 0.76 | 1.14 | 0.04 | 0.00 | 0.04 | 0.04 | 0.76 | 1.14 | 0.04 | 0.00 |
| Ord.0.55Load.Cross250.8Var.500Size.2Fac.0.70Fcor | 1.07 | 1.07 | 2.70 | 3.64 | 0.18 | 0.18 | 0.62 | 0.91 | 0.04 | -0.31 | 0.28 | 0.28 | 0.62 | 0.91 | 0.04 | 0.31 |
| Ord.0.55Load.Cross250.8Var.1000Size.2Fac.0.00Fcor | 0.09 | 0.17 | 2.20 | 3.19 | 0.00 | 0.00 | 0.46 | 0.83 | 0.21 | 0.00 | 0.00 | 0.00 | 0.46 | 0.83 | 0.21 | 0.00 |
| Ord.0.55Load.Cross250.8Var.1000Size.2Fac.0.50Fcor | 0.01 | 0.01 | 1.93 | 3.21 | 0.00 | 0.00 | 0.60 | 0.79 | 0.01 | 0.00 | 0.00 | 0.00 | 0.60 | 0.79 | 0.01 | 0.00 |
| Ord.0.55Load.Cross250.8Var.1000Size.2Fac.0.70Fcor | 0.39 | 0.39 | 2.81 | 3.72 | 0.03 | 0.03 | 0.25 | 0.37 | 0.00 | -0.04 | 0.05 | 0.05 | 0.25 | 0.43 | 0.00 | 0.04 |
| Ord.0.55Load.Cross250.8Var.300Size.4Fac.0.00Fcor | 0.59 | 0.61 | 2.88 | 5.11 | 0.39 | 0.35 | -0.46 | 2.19 | 0.04 | 0.00 | 0.45 | 0.43 | 1.54 | 2.19 | 0.04 | 0.00 |
| Ord.0.55Load.Cross250.8Var.300Size.4Fac.0.50Fcor | 4.16 | 4.14 | 4.17 | 6.42 | 0.70 | 0.75 | -0.32 | 2.43 | -0.02 | -0.10 | 1.18 | 1.23 | 1.50 | 2.53 | 0.20 | 0.10 |
| Ord.0.55Load.Cross250.8Var.300Size.4Fac.0.70Fcor | 8.17 | 8.17 | 5.88 | 8.22 | 1.25 | 1.24 | -0.06 | 2.03 | -0.02 | -2.59 | 1.83 | 1.82 | 1.22 | 2.23 | 0.60 | 2.59 |
| Ord.0.55Load.Cross250.8Var.500Size.4Fac.0.00Fcor | 0.09 | 0.09 | 1.80 | 4.54 | 0.04 | 0.05 | -1.11 | 1.67 | 0.07 | 0.00 | 0.04 | 0.05 | 1.45 | 1.67 | 0.07 | 0.00 |
| Ord.0.55Load.Cross250.8Var.500Size.4Fac.0.50Fcor | 1.32 | 1.32 | 2.35 | 4.46 | 0.49 | 0.49 | -0.39 | 2.74 | 0.22 | 0.00 | 0.67 | 0.67 | 1.43 | 2.74 | 0.22 | 0.00 |
| Ord.0.55Load.Cross250.8Var.500Size.4Fac.0.70Fcor | 6.27 | 6.27 | 4.82 | 6.75 | 0.16 | 0.16 | -0.38 | 1.58 | 0.03 | -2.09 | 1.24 | 1.24 | 1.48 | 1.80 | 0.27 | 2.09 |
| Ord.0.55Load.Cross250.8Var.1000Size.4Fac.0.00Fcor | 0.03 | 0.03 | 1.06 | 4.22 | 0.00 | 0.00 | -1.43 | 0.92 | 0.13 | 0.00 | 0.00 | 0.00 | 1.49 | 0.92 | 0.13 | 0.00 |
| Ord.0.55Load.Cross250.8Var.1000Size.4Fac.0.50Fcor | 0.20 | 0.20 | 1.83 | 3.47 | 0.04 | 0.04 | -0.95 | 1.51 | 0.26 | 0.00 | 0.06 | 0.06 | 1.45 | 1.53 | 0.26 | 0.00 |
| Ord.0.55Load.Cross250.8Var.1000Size.4Fac.0.70Fcor | 3.18 | 3.18 | 4.21 | 5.69 | -0.09 | -0.09 | -0.87 | 0.86 | 0.21 | -0.60 | 0.83 | 0.83 | 1.29 | 1.20 | 0.31 | 0.60 |
| Ord.0.70Load.000Cross.4Var.300Size.2Fac.0.00Fcor | 0.00 | 0.00 | 0.34 | 0.92 | 0.00 | 0.00 | 0.07 | 0.05 | 0.00 | 0.00 | 0.00 | 0.00 | 0.07 | 0.21 | 0.00 | 0.00 |
| Ord.0.70Load.000Cross.4Var.300Size.2Fac.0.50Fcor | 0.00 | 0.00 | 0.21 | 1.05 | 0.00 | 0.00 | 0.07 | -0.02 | 0.00 | -0.02 | 0.00 | 0.00 | 0.07 | 0.18 | 0.00 | 0.02 |
| Ord.0.70Load.000Cross.4Var.300Size.2Fac.0.70Fcor | 0.06 | 0.06 | 0.26 | 0.90 | 0.02 | 0.02 | 0.09 | 0.07 | -0.24 | -0.79 | 0.02 | 0.02 | 0.09 | 0.25 | 0.24 | 0.79 |
| Ord.0.70Load.000Cross.4Var.500Size.2Fac.0.00Fcor | 0.00 | 0.00 | 0.39 | 0.78 | 0.00 | 0.00 | 0.07 | 0.03 | 0.00 | 0.00 | 0.00 | 0.00 | 0.07 | 0.17 | 0.00 | 0.00 |
| Ord.0.70Load.000Cross.4Var.500Size.2Fac.0.50Fcor | 0.00 | 0.00 | 0.29 | 0.84 | 0.00 | 0.00 | 0.05 | 0.00 | 0.00 | 0.00 | 0.00 | 0.00 | 0.05 | 0.16 | 0.00 | 0.00 |
| Ord.0.70Load.000Cross.4Var.500Size.2Fac.0.70Fcor | 0.00 | 0.00 | 0.25 | 0.66 | 0.00 | 0.00 | 0.09 | 0.07 | -0.11 | -0.63 | 0.00 | 0.00 | 0.09 | 0.19 | 0.11 | 0.63 |
| Ord.0.70Load.000Cross.4Var.1000Size.2Fac.0.00Fcor | 0.00 | 0.00 | 0.33 | 0.53 | 0.00 | 0.00 | 0.06 | 0.08 | 0.00 | 0.00 | 0.00 | 0.00 | 0.06 | 0.14 | 0.00 | 0.00 |
| Ord.0.70Load.000Cross.4Var.1000Size.2Fac.0.50Fcor | 0.00 | 0.00 | 0.22 | 0.64 | 0.00 | 0.00 | 0.03 | -0.01 | 0.00 | 0.00 | 0.00 | 0.00 | 0.03 | 0.09 | 0.00 | 0.00 |
| Ord.0.70Load.000Cross.4Var.1000Size.2Fac.0.70Fcor | 0.01 | 0.01 | 0.21 | 0.80 | 0.00 | 0.00 | 0.05 | -0.03 | -0.12 | -0.56 | 0.00 | 0.00 | 0.05 | 0.17 | 0.12 | 0.56 |
| Ord.0.70Load.000Cross.4Var.300Size.4Fac.0.00Fcor | 0.00 | 0.00 | 2.38 | 0.77 | 0.00 | 0.00 | -0.43 | 0.20 | 0.00 | 0.00 | 0.00 | 0.00 | 0.55 | 0.28 | 0.00 | 0.00 |
| Ord.0.70Load.000Cross.4Var.300Size.4Fac.0.50Fcor | 0.00 | 0.00 | 0.76 | 0.74 | 0.01 | 0.01 | -1.25 | 0.36 | 0.00 | -0.17 | 0.01 | 0.01 | 1.31 | 0.42 | 0.00 | 0.17 |
| Ord.0.70Load.000Cross.4Var.300Size.4Fac.0.70Fcor | 0.43 | 0.43 | 0.76 | 1.39 | -0.06 | -0.06 | -1.30 | 0.19 | -0.03 | -2.86 | 0.18 | 0.18 | 1.40 | 0.51 | 0.05 | 2.86 |
| Ord.0.70Load.000Cross.4Var.500Size.4Fac.0.00Fcor | 0.00 | 0.00 | 1.29 | 0.64 | 0.00 | 0.00 | -0.35 | 0.13 | 0.00 | 0.00 | 0.00 | 0.00 | 0.47 | 0.15 | 0.00 | 0.00 |
| Ord.0.70Load.000Cross.4Var.500Size.4Fac.0.50Fcor | 0.00 | 0.00 | 0.90 | 0.95 | 0.00 | 0.00 | -1.43 | 0.17 | 0.00 | 0.00 | 0.00 | 0.00 | 1.47 | 0.23 | 0.00 | 0.00 |
| Ord.0.70Load.000Cross.4Var.500Size.4Fac.0.70Fcor | 0.10 | 0.10 | 0.89 | 1.08 | 0.02 | 0.02 | -1.33 | 0.21 | -0.02 | -2.62 | 0.04 | 0.04 | 1.37 | 0.35 | 0.02 | 2.62 |
| Ord.0.70Load.000Cross.4Var.1000Size.4Fac.0.00Fcor | 0.00 | 0.00 | 1.02 | 0.52 | 0.00 | 0.00 | -0.49 | 0.26 | 0.00 | 0.00 | 0.00 | 0.00 | 0.59 | 0.28 | 0.00 | 0.00 |
| Ord.0.70Load.000Cross.4Var.1000Size.4Fac.0.50Fcor | 0.00 | 0.00 | 0.90 | 0.89 | 0.00 | 0.00 | -1.20 | 0.18 | 0.00 | 0.00 | 0.00 | 0.00 | 1.30 | 0.26 | 0.00 | 0.00 |
| Ord.0.70Load.000Cross.4Var.1000Size.4Fac.0.70Fcor | 0.03 | 0.03 | 0.41 | 0.77 | 0.00 | 0.00 | -1.21 | 0.15 | 0.00 | -2.23 | 0.02 | 0.02 | 1.23 | 0.23 | 0.00 | 2.23 |
| Ord.0.70Load.000Cross.8Var.300Size.2Fac.0.00Fcor | 0.00 | 0.00 | 2.18 | 2.76 | 0.00 | 0.00 | 0.97 | 1.26 | 0.00 | 0.00 | 0.00 | 0.00 | 0.97 | 1.26 | 0.00 | 0.00 |
| Ord.0.70Load.000Cross.8Var.300Size.2Fac.0.50Fcor | 0.00 | 0.00 | 1.58 | 2.86 | 0.00 | 0.00 | 0.81 | 1.51 | 0.00 | 0.00 | 0.00 | 0.00 | 0.81 | 1.51 | 0.00 | 0.00 |
| Ord.0.70Load.000Cross.8Var.300Size.2Fac.0.70Fcor | 0.06 | 0.06 | 1.79 | 2.89 | 0.02 | 0.02 | 0.75 | 1.58 | 0.00 | -0.01 | 0.02 | 0.02 | 0.75 | 1.58 | 0.00 | 0.01 |
| Ord.0.70Load.000Cross.8Var.500Size.2Fac.0.00Fcor | 0.00 | 0.00 | 2.27 | 3.61 | 0.00 | 0.00 | 0.70 | 1.05 | 0.00 | 0.00 | 0.00 | 0.00 | 0.70 | 1.05 | 0.00 | 0.00 |
| Ord.0.70Load.000Cross.8Var.500Size.2Fac.0.50Fcor | 0.00 | 0.00 | 1.93 | 2.77 | 0.00 | 0.00 | 0.65 | 1.10 | 0.00 | 0.00 | 0.00 | 0.00 | 0.65 | 1.10 | 0.00 | 0.00 |
| Ord.0.70Load.000Cross.8Var.500Size.2Fac.0.70Fcor | 0.00 | 0.00 | 2.13 | 2.92 | 0.02 | 0.02 | 0.53 | 1.04 | 0.00 | 0.00 | 0.02 | 0.02 | 0.53 | 1.04 | 0.00 | 0.00 |
| Ord.0.70Load.000Cross.8Var.1000Size.2Fac.0.00Fcor | 0.00 | 0.00 | 1.90 | 2.88 | 0.00 | 0.00 | 0.69 | 1.03 | 0.00 | 0.00 | 0.00 | 0.00 | 0.69 | 1.03 | 0.00 | 0.00 |
| Ord.0.70Load.000Cross.8Var.1000Size.2Fac.0.50Fcor | 0.00 | 0.00 | 1.79 | 2.76 | 0.00 | 0.00 | 0.50 | 0.75 | 0.00 | 0.00 | 0.00 | 0.00 | 0.50 | 0.75 | 0.00 | 0.00 |
| Ord.0.70Load.000Cross.8Var.1000Size.2Fac.0.70Fcor | 0.00 | 0.00 | 2.42 | 3.09 | 0.00 | 0.00 | 0.57 | 0.86 | 0.00 | 0.00 | 0.00 | 0.00 | 0.57 | 0.86 | 0.00 | 0.00 |
| Ord.0.70Load.000Cross.8Var.300Size.4Fac.0.00Fcor | 0.00 | 0.00 | 6.92 | 4.90 | 0.00 | 0.00 | 0.87 | 2.78 | 0.00 | 0.00 | 0.00 | 0.00 | 1.65 | 2.78 | 0.00 | 0.00 |
| Ord.0.70Load.000Cross.8Var.300Size.4Fac.0.50Fcor | 0.01 | 0.01 | 2.19 | 4.54 | 0.01 | 0.01 | -0.64 | 2.71 | 0.00 | 0.00 | 0.01 | 0.01 | 1.52 | 2.71 | 0.00 | 0.00 |
| Ord.0.70Load.000Cross.8Var.300Size.4Fac.0.70Fcor | 0.37 | 0.37 | 2.86 | 4.78 | 0.14 | 0.14 | -0.88 | 2.18 | 0.00 | -0.41 | 0.16 | 0.16 | 1.34 | 2.18 | 0.00 | 0.41 |
| Ord.0.70Load.000Cross.8Var.500Size.4Fac.0.00Fcor | 0.00 | 0.00 | 5.66 | 3.15 | 0.00 | 0.00 | 0.22 | 1.83 | 0.00 | 0.00 | 0.00 | 0.00 | 1.38 | 1.83 | 0.00 | 0.00 |
| Ord.0.70Load.000Cross.8Var.500Size.4Fac.0.50Fcor | 0.00 | 0.00 | 2.60 | 4.96 | 0.00 | 0.00 | -0.83 | 1.81 | 0.00 | 0.00 | 0.00 | 0.00 | 1.25 | 1.81 | 0.00 | 0.00 |
| Ord.0.70Load.000Cross.8Var.500Size.4Fac.0.70Fcor | 0.05 | 0.05 | 2.32 | 4.53 | 0.03 | 0.03 | -1.03 | 1.67 | 0.00 | -0.02 | 0.03 | 0.03 | 1.27 | 1.67 | 0.00 | 0.02 |
| Ord.0.70Load.000Cross.8Var.1000Size.4Fac.0.00Fcor | 0.00 | 0.00 | 6.25 | 3.77 | 0.00 | 0.00 | 0.06 | 1.52 | 0.00 | 0.00 | 0.00 | 0.00 | 1.06 | 1.52 | 0.00 | 0.00 |
| Ord.0.70Load.000Cross.8Var.1000Size.4Fac.0.50Fcor | 0.00 | 0.00 | 2.86 | 3.89 | 0.00 | 0.00 | -0.88 | 0.93 | 0.00 | 0.00 | 0.00 | 0.00 | 1.22 | 0.93 | 0.00 | 0.00 |
| Ord.0.70Load.000Cross.8Var.1000Size.4Fac.0.70Fcor | 0.02 | 0.02 | 2.48 | 3.08 | 0.01 | 0.01 | -1.06 | 1.01 | 0.00 | 0.00 | 0.01 | 0.01 | 1.34 | 1.01 | 0.00 | 0.00 |
| Ord.0.70Load.125Cross.4Var.300Size.2Fac.0.00Fcor | 0.01 | 0.01 | 0.25 | 1.04 | 0.00 | 0.00 | 0.05 | 0.08 | 0.00 | 0.00 | 0.00 | 0.00 | 0.05 | 0.16 | 0.00 | 0.00 |
| Ord.0.70Load.125Cross.4Var.300Size.2Fac.0.50Fcor | 0.01 | 0.01 | 0.20 | 1.67 | 0.00 | 0.00 | 0.10 | -0.06 | 0.00 | -0.09 | 0.00 | 0.00 | 0.10 | 0.42 | 0.00 | 0.09 |
| Ord.0.70Load.125Cross.4Var.300Size.2Fac.0.70Fcor | 0.06 | 0.06 | 0.38 | 1.73 | 0.00 | 0.00 | 0.09 | 0.02 | -0.62 | -0.94 | 0.00 | 0.00 | 0.09 | 0.30 | 0.62 | 0.94 |
| Ord.0.70Load.125Cross.4Var.500Size.2Fac.0.00Fcor | 0.00 | 0.00 | 0.30 | 1.17 | 0.00 | 0.00 | 0.02 | 0.07 | 0.00 | 0.00 | 0.00 | 0.00 | 0.02 | 0.15 | 0.00 | 0.00 |
| Ord.0.70Load.125Cross.4Var.500Size.2Fac.0.50Fcor | 0.00 | 0.00 | 0.28 | 1.42 | 0.00 | 0.00 | 0.06 | 0.06 | 0.00 | -0.01 | 0.00 | 0.00 | 0.06 | 0.44 | 0.00 | 0.01 |
| Ord.0.70Load.125Cross.4Var.500Size.2Fac.0.70Fcor | 0.02 | 0.02 | 0.32 | 1.83 | 0.00 | 0.00 | 0.06 | -0.10 | -0.77 | -0.93 | 0.00 | 0.00 | 0.06 | 0.32 | 0.77 | 0.93 |
| Ord.0.70Load.125Cross.4Var.1000Size.2Fac.0.00Fcor | 0.00 | 0.00 | 0.34 | 0.84 | 0.00 | 0.00 | 0.01 | 0.03 | 0.00 | 0.00 | 0.00 | 0.00 | 0.01 | 0.03 | 0.00 | 0.00 |
| Ord.0.70Load.125Cross.4Var.1000Size.2Fac.0.50Fcor | 0.00 | 0.00 | 0.21 | 2.01 | 0.00 | 0.00 | 0.05 | -0.24 | 0.00 | 0.00 | 0.00 | 0.00 | 0.05 | 0.42 | 0.00 | 0.00 |
| Ord.0.70Load.125Cross.4Var.1000Size.2Fac.0.70Fcor | 0.01 | 0.01 | 0.33 | 1.83 | 0.00 | 0.00 | 0.01 | -0.20 | -0.92 | -0.95 | 0.00 | 0.00 | 0.01 | 0.22 | 0.92 | 0.95 |
| Ord.0.70Load.125Cross.4Var.300Size.4Fac.0.00Fcor | 0.00 | 0.00 | 0.91 | 1.49 | 0.00 | 0.00 | -0.93 | 0.20 | 0.00 | 0.00 | 0.00 | 0.00 | 1.07 | 0.24 | 0.00 | 0.00 |
| Ord.0.70Load.125Cross.4Var.300Size.4Fac.0.50Fcor | 0.04 | 0.04 | 0.34 | 1.23 | 0.00 | 0.00 | -1.40 | 0.33 | -0.03 | -0.66 | 0.04 | 0.04 | 1.42 | 0.41 | 0.03 | 0.66 |
| Ord.0.70Load.125Cross.4Var.300Size.4Fac.0.70Fcor | 0.60 | 0.60 | 0.87 | 1.96 | -0.14 | -0.14 | -1.42 | 0.05 | -0.45 | -2.86 | 0.36 | 0.36 | 1.46 | 0.45 | 0.47 | 2.86 |
| Ord.0.70Load.125Cross.4Var.500Size.4Fac.0.00Fcor | 0.00 | 0.00 | 0.31 | 1.87 | 0.00 | -0.01 | -1.25 | 0.11 | -0.01 | 0.00 | 0.00 | 0.01 | 1.27 | 0.11 | 0.01 | 0.00 |
| Ord.0.70Load.125Cross.4Var.500Size.4Fac.0.50Fcor | 0.00 | 0.00 | 0.34 | 1.14 | 0.00 | 0.00 | -1.37 | 0.33 | -0.10 | -0.23 | 0.00 | 0.00 | 1.41 | 0.37 | 0.10 | 0.23 |
| Ord.0.70Load.125Cross.4Var.500Size.4Fac.0.70Fcor | 0.33 | 0.33 | 0.46 | 1.72 | -0.16 | -0.16 | -1.37 | 0.13 | -0.27 | -2.77 | 0.24 | 0.24 | 1.39 | 0.31 | 0.29 | 2.77 |
| Ord.0.70Load.125Cross.4Var.1000Size.4Fac.0.00Fcor | 0.00 | 0.00 | 0.09 | 1.69 | 0.00 | 0.00 | -1.62 | 0.04 | -0.02 | 0.00 | 0.00 | 0.00 | 1.62 | 0.04 | 0.02 | 0.00 |
| Ord.0.70Load.125Cross.4Var.1000Size.4Fac.0.50Fcor | 0.00 | 0.00 | 0.38 | 1.05 | -0.01 | -0.01 | -1.45 | 0.18 | -0.05 | -0.03 | 0.01 | 0.01 | 1.45 | 0.32 | 0.05 | 0.03 |
| Ord.0.70Load.125Cross.4Var.1000Size.4Fac.0.70Fcor | 0.24 | 0.24 | 0.39 | 1.53 | -0.13 | -0.13 | -1.52 | -0.01 | -0.31 | -2.44 | 0.15 | 0.15 | 1.52 | 0.19 | 0.33 | 2.44 |
| Ord.0.70Load.125Cross.8Var.300Size.2Fac.0.00Fcor | 0.21 | 0.25 | 1.76 | 2.93 | 0.02 | 0.04 | 0.83 | 1.44 | 0.49 | 0.00 | 0.02 | 0.04 | 0.83 | 1.44 | 0.49 | 0.00 |
| Ord.0.70Load.125Cross.8Var.300Size.2Fac.0.50Fcor | 0.01 | 0.01 | 1.83 | 2.61 | 0.01 | 0.01 | 1.10 | 1.89 | 0.29 | 0.00 | 0.01 | 0.01 | 1.10 | 1.89 | 0.29 | 0.00 |
| Ord.0.70Load.125Cross.8Var.300Size.2Fac.0.70Fcor | 0.13 | 0.13 | 2.54 | 2.99 | 0.01 | 0.01 | 0.81 | 1.07 | 0.06 | -0.11 | 0.01 | 0.01 | 0.81 | 1.07 | 0.06 | 0.11 |
| Ord.0.70Load.125Cross.8Var.500Size.2Fac.0.00Fcor | 0.37 | 0.50 | 2.12 | 2.89 | 0.04 | 0.04 | 0.55 | 1.03 | 0.59 | 0.00 | 0.04 | 0.04 | 0.55 | 1.03 | 0.59 | 0.00 |
| Ord.0.70Load.125Cross.8Var.500Size.2Fac.0.50Fcor | 0.00 | 0.00 | 2.48 | 2.53 | 0.00 | 0.00 | 0.99 | 1.61 | 0.22 | 0.00 | 0.00 | 0.00 | 0.99 | 1.61 | 0.22 | 0.00 |
| Ord.0.70Load.125Cross.8Var.500Size.2Fac.0.70Fcor | 0.02 | 0.02 | 2.44 | 3.07 | 0.01 | 0.01 | 0.67 | 0.72 | 0.02 | 0.00 | 0.01 | 0.01 | 0.67 | 0.72 | 0.02 | 0.00 |
| Ord.0.70Load.125Cross.8Var.1000Size.2Fac.0.00Fcor | 0.29 | 0.53 | 1.66 | 3.40 | 0.04 | 0.06 | 0.38 | 0.74 | 0.63 | 0.00 | 0.04 | 0.06 | 0.38 | 0.74 | 0.63 | 0.00 |
| Ord.0.70Load.125Cross.8Var.1000Size.2Fac.0.50Fcor | 0.04 | 0.04 | 2.86 | 3.05 | 0.00 | 0.00 | 0.89 | 1.20 | 0.31 | 0.00 | 0.00 | 0.00 | 0.89 | 1.20 | 0.31 | 0.00 |
| Ord.0.70Load.125Cross.8Var.1000Size.2Fac.0.70Fcor | 0.00 | 0.00 | 1.71 | 3.43 | 0.00 | 0.00 | 0.26 | 0.19 | 0.00 | 0.00 | 0.00 | 0.00 | 0.26 | 0.27 | 0.00 | 0.00 |
| Ord.0.70Load.125Cross.8Var.300Size.4Fac.0.00Fcor | 0.00 | 0.00 | 5.02 | 5.10 | 0.00 | 0.01 | -0.48 | 2.18 | 0.00 | 0.00 | 0.00 | 0.01 | 1.14 | 2.18 | 0.00 | 0.00 |
| Ord.0.70Load.125Cross.8Var.300Size.4Fac.0.50Fcor | 0.03 | 0.03 | 2.03 | 4.32 | 0.04 | 0.04 | -0.84 | 2.93 | 0.05 | 0.00 | 0.04 | 0.04 | 1.46 | 2.93 | 0.05 | 0.00 |
| Ord.0.70Load.125Cross.8Var.300Size.4Fac.0.70Fcor | 0.47 | 0.47 | 2.82 | 5.39 | 0.21 | 0.21 | -0.72 | 2.41 | 0.02 | -0.67 | 0.29 | 0.29 | 1.34 | 2.41 | 0.02 | 0.67 |
| Ord.0.70Load.125Cross.8Var.500Size.4Fac.0.00Fcor | 0.00 | 0.00 | 4.34 | 4.80 | 0.00 | 0.00 | -0.64 | 1.28 | 0.00 | 0.00 | 0.00 | 0.00 | 1.08 | 1.28 | 0.00 | 0.00 |
| Ord.0.70Load.125Cross.8Var.500Size.4Fac.0.50Fcor | 0.00 | 0.00 | 1.86 | 4.12 | 0.00 | 0.00 | -0.74 | 2.52 | 0.04 | 0.00 | 0.00 | 0.00 | 1.30 | 2.52 | 0.04 | 0.00 |
| Ord.0.70Load.125Cross.8Var.500Size.4Fac.0.70Fcor | 0.03 | 0.03 | 2.42 | 4.96 | 0.03 | 0.03 | -0.97 | 1.77 | 0.00 | -0.14 | 0.03 | 0.03 | 1.39 | 1.77 | 0.00 | 0.14 |
| Ord.0.70Load.125Cross.8Var.1000Size.4Fac.0.00Fcor | 0.00 | 0.00 | 2.28 | 5.93 | 0.00 | 0.00 | -0.71 | 1.13 | 0.02 | 0.00 | 0.00 | 0.00 | 1.17 | 1.13 | 0.02 | 0.00 |
| Ord.0.70Load.125Cross.8Var.1000Size.4Fac.0.50Fcor | 0.00 | 0.00 | 2.27 | 2.91 | 0.00 | 0.00 | -1.01 | 1.02 | 0.04 | 0.00 | 0.00 | 0.00 | 1.17 | 1.02 | 0.04 | 0.00 |
| Ord.0.70Load.125Cross.8Var.1000Size.4Fac.0.70Fcor | 0.02 | 0.02 | 3.21 | 3.62 | 0.00 | 0.00 | -1.24 | 0.84 | 0.06 | 0.00 | 0.00 | 0.00 | 1.32 | 0.84 | 0.06 | 0.00 |
| Ord.0.70Load.Cross250.4Var.300Size.2Fac.0.00Fcor | 0.31 | 0.39 | 0.52 | 1.39 | 0.05 | 0.09 | 0.16 | 0.27 | 0.22 | 0.00 | 0.05 | 0.09 | 0.16 | 0.31 | 0.22 | 0.00 |
| Ord.0.70Load.Cross250.4Var.300Size.2Fac.0.50Fcor | 0.03 | 0.03 | 0.74 | 1.64 | 0.04 | 0.04 | 0.12 | -0.01 | -0.18 | -0.39 | 0.06 | 0.06 | 0.12 | 0.25 | 0.24 | 0.39 |
| Ord.0.70Load.Cross250.4Var.300Size.2Fac.0.70Fcor | 0.31 | 0.31 | 0.60 | 2.23 | -0.09 | -0.09 | -0.07 | -0.45 | -0.95 | -0.99 | 0.09 | 0.09 | 0.09 | 0.47 | 0.97 | 0.99 |
| Ord.0.70Load.Cross250.4Var.500Size.2Fac.0.00Fcor | 0.26 | 0.24 | 0.45 | 1.61 | 0.02 | 0.06 | 0.22 | 0.18 | 0.24 | 0.00 | 0.02 | 0.06 | 0.22 | 0.18 | 0.24 | 0.00 |
| Ord.0.70Load.Cross250.4Var.500Size.2Fac.0.50Fcor | 0.03 | 0.03 | 0.48 | 1.49 | -0.01 | -0.01 | 0.16 | -0.02 | -0.19 | -0.16 | 0.01 | 0.01 | 0.16 | 0.36 | 0.21 | 0.16 |
| Ord.0.70Load.Cross250.4Var.500Size.2Fac.0.70Fcor | 0.07 | 0.07 | 0.41 | 1.67 | -0.02 | -0.02 | -0.01 | -0.32 | -1.00 | -1.00 | 0.02 | 0.02 | 0.07 | 0.36 | 1.00 | 1.00 |
| Ord.0.70Load.Cross250.4Var.1000Size.2Fac.0.00Fcor | 0.11 | 0.11 | 0.71 | 1.80 | 0.01 | 0.01 | 0.17 | 0.10 | 0.17 | 0.00 | 0.01 | 0.01 | 0.17 | 0.10 | 0.17 | 0.00 |
| Ord.0.70Load.Cross250.4Var.1000Size.2Fac.0.50Fcor | 0.00 | 0.00 | 0.31 | 1.19 | 0.00 | 0.00 | 0.13 | 0.01 | -0.14 | -0.06 | 0.00 | 0.00 | 0.13 | 0.37 | 0.18 | 0.06 |
| Ord.0.70Load.Cross250.4Var.1000Size.2Fac.0.70Fcor | 0.00 | 0.00 | 0.20 | 1.48 | 0.00 | 0.00 | 0.00 | -0.31 | -1.00 | -1.00 | 0.00 | 0.00 | 0.00 | 0.31 | 1.00 | 1.00 |
| Ord.0.70Load.Cross250.4Var.300Size.4Fac.0.00Fcor | 0.00 | 0.00 | 0.53 | 2.46 | 0.00 | 0.01 | -1.12 | 0.12 | 0.00 | 0.00 | 0.00 | 0.01 | 1.12 | 0.12 | 0.00 | 0.00 |
| Ord.0.70Load.Cross250.4Var.300Size.4Fac.0.50Fcor | 0.01 | 0.01 | 0.41 | 0.97 | -0.04 | -0.04 | -1.28 | 0.44 | -0.16 | -0.85 | 0.04 | 0.04 | 1.34 | 0.48 | 0.18 | 0.85 |
| Ord.0.70Load.Cross250.4Var.300Size.4Fac.0.70Fcor | 0.60 | 0.63 | 0.64 | 1.89 | -0.34 | -0.34 | -1.39 | 0.26 | -1.26 | -2.94 | 0.36 | 0.36 | 1.47 | 0.46 | 1.26 | 2.94 |
| Ord.0.70Load.Cross250.4Var.500Size.4Fac.0.00Fcor | 0.00 | 0.00 | 0.27 | 2.84 | 0.00 | 0.00 | -1.27 | 0.02 | -0.01 | 0.00 | 0.00 | 0.00 | 1.29 | 0.02 | 0.01 | 0.00 |
| Ord.0.70Load.Cross250.4Var.500Size.4Fac.0.50Fcor | 0.00 | 0.00 | 0.51 | 0.94 | 0.01 | 0.01 | -1.11 | 0.48 | -0.06 | -0.45 | 0.01 | 0.01 | 1.19 | 0.48 | 0.06 | 0.45 |
| Ord.0.70Load.Cross250.4Var.500Size.4Fac.0.70Fcor | 0.19 | 0.19 | 0.58 | 1.51 | -0.13 | -0.13 | -1.32 | 0.16 | -1.08 | -2.92 | 0.15 | 0.15 | 1.38 | 0.26 | 1.08 | 2.92 |
| Ord.0.70Load.Cross250.4Var.1000Size.4Fac.0.00Fcor | 0.00 | 0.00 | 0.22 | 3.37 | 0.00 | 0.00 | -1.22 | 0.00 | 0.00 | 0.00 | 0.00 | 0.00 | 1.22 | 0.00 | 0.00 | 0.00 |
| Ord.0.70Load.Cross250.4Var.1000Size.4Fac.0.50Fcor | 0.00 | 0.00 | 0.45 | 0.90 | 0.00 | 0.00 | -1.18 | 0.39 | -0.02 | -0.15 | 0.00 | 0.00 | 1.24 | 0.39 | 0.02 | 0.15 |
| Ord.0.70Load.Cross250.4Var.1000Size.4Fac.0.70Fcor | 0.11 | 0.11 | 0.33 | 1.37 | 0.02 | 0.02 | -1.30 | 0.14 | -1.27 | -2.70 | 0.02 | 0.02 | 1.30 | 0.14 | 1.27 | 2.70 |
| Ord.0.70Load.Cross250.8Var.300Size.2Fac.0.00Fcor | 0.21 | 0.25 | 1.76 | 2.93 | 0.02 | 0.04 | 0.83 | 1.44 | 0.49 | 0.00 | 0.02 | 0.04 | 0.83 | 1.44 | 0.49 | 0.00 |
| Ord.0.70Load.Cross250.8Var.300Size.2Fac.0.50Fcor | 0.01 | 0.01 | 1.83 | 2.61 | 0.01 | 0.01 | 1.10 | 1.89 | 0.29 | 0.00 | 0.01 | 0.01 | 1.10 | 1.89 | 0.29 | 0.00 |
| Ord.0.70Load.Cross250.8Var.300Size.2Fac.0.70Fcor | 0.13 | 0.13 | 2.54 | 2.99 | 0.01 | 0.01 | 0.81 | 1.07 | 0.06 | -0.11 | 0.01 | 0.01 | 0.81 | 1.07 | 0.06 | 0.11 |
| Ord.0.70Load.Cross250.8Var.500Size.2Fac.0.00Fcor | 0.37 | 0.50 | 2.12 | 2.89 | 0.04 | 0.04 | 0.55 | 1.03 | 0.59 | 0.00 | 0.04 | 0.04 | 0.55 | 1.03 | 0.59 | 0.00 |
| Ord.0.70Load.Cross250.8Var.500Size.2Fac.0.50Fcor | 0.00 | 0.00 | 2.48 | 2.53 | 0.00 | 0.00 | 0.99 | 1.61 | 0.22 | 0.00 | 0.00 | 0.00 | 0.99 | 1.61 | 0.22 | 0.00 |
| Ord.0.70Load.Cross250.8Var.500Size.2Fac.0.70Fcor | 0.02 | 0.02 | 2.44 | 3.07 | 0.01 | 0.01 | 0.67 | 0.72 | 0.02 | 0.00 | 0.01 | 0.01 | 0.67 | 0.72 | 0.02 | 0.00 |
| Ord.0.70Load.Cross250.8Var.1000Size.2Fac.0.00Fcor | 0.29 | 0.53 | 1.66 | 3.40 | 0.04 | 0.06 | 0.38 | 0.74 | 0.63 | 0.00 | 0.04 | 0.06 | 0.38 | 0.74 | 0.63 | 0.00 |
| Ord.0.70Load.Cross250.8Var.1000Size.2Fac.0.50Fcor | 0.04 | 0.04 | 2.86 | 3.05 | 0.00 | 0.00 | 0.89 | 1.20 | 0.31 | 0.00 | 0.00 | 0.00 | 0.89 | 1.20 | 0.31 | 0.00 |
| Ord.0.70Load.Cross250.8Var.1000Size.2Fac.0.70Fcor | 0.00 | 0.00 | 1.71 | 3.43 | 0.00 | 0.00 | 0.26 | 0.19 | 0.00 | 0.00 | 0.00 | 0.00 | 0.26 | 0.27 | 0.00 | 0.00 |
| Ord.0.70Load.Cross250.8Var.300Size.4Fac.0.00Fcor | 0.08 | 0.08 | 1.62 | 5.61 | 0.01 | 0.01 | -1.27 | 1.91 | 0.06 | 0.00 | 0.01 | 0.01 | 1.47 | 1.91 | 0.06 | 0.00 |
| Ord.0.70Load.Cross250.8Var.300Size.4Fac.0.50Fcor | 0.05 | 0.05 | 1.54 | 3.91 | 0.10 | 0.10 | -0.98 | 3.38 | 0.28 | 0.00 | 0.10 | 0.10 | 1.56 | 3.38 | 0.28 | 0.00 |
| Ord.0.70Load.Cross250.8Var.300Size.4Fac.0.70Fcor | 0.82 | 0.82 | 1.18 | 5.04 | 0.26 | 0.26 | -0.78 | 3.12 | 0.08 | -1.07 | 0.42 | 0.42 | 1.54 | 3.12 | 0.14 | 1.07 |
| Ord.0.70Load.Cross250.8Var.500Size.4Fac.0.00Fcor | 0.01 | 0.02 | 0.41 | 5.77 | 0.00 | 0.00 | -1.44 | 1.75 | 0.12 | 0.00 | 0.00 | 0.00 | 1.54 | 1.75 | 0.12 | 0.00 |
| Ord.0.70Load.Cross250.8Var.500Size.4Fac.0.50Fcor | 0.02 | 0.02 | 2.17 | 4.01 | 0.00 | 0.00 | -0.90 | 3.07 | 0.41 | 0.00 | 0.00 | 0.00 | 1.68 | 3.07 | 0.41 | 0.00 |
| Ord.0.70Load.Cross250.8Var.500Size.4Fac.0.70Fcor | 0.31 | 0.31 | 2.19 | 4.76 | -0.02 | -0.02 | -0.66 | 2.07 | 0.16 | -0.26 | 0.04 | 0.04 | 1.42 | 2.07 | 0.20 | 0.26 |
| Ord.0.70Load.Cross250.8Var.1000Size.4Fac.0.00Fcor | 0.04 | 0.06 | 0.48 | 4.39 | 0.00 | 0.00 | -1.50 | 1.17 | 0.15 | 0.00 | 0.00 | 0.00 | 1.54 | 1.17 | 0.15 | 0.00 |
| Ord.0.70Load.Cross250.8Var.1000Size.4Fac.0.50Fcor | 0.02 | 0.02 | 1.31 | 3.76 | 0.00 | 0.00 | -1.39 | 1.93 | 0.79 | 0.00 | 0.00 | 0.00 | 1.69 | 1.93 | 0.79 | 0.00 |
| Ord.0.70Load.Cross250.8Var.1000Size.4Fac.0.70Fcor | 0.00 | 0.00 | 2.94 | 5.69 | 0.00 | 0.00 | -1.09 | 1.27 | 0.48 | 0.00 | 0.00 | 0.00 | 1.53 | 1.27 | 0.48 | 0.00 |
| Dich.0.40Load.000Cross.4Var.300Size.2Fac.0.00Fcor | 3.80 | 3.79 | 4.96 | 5.16 | -0.68 | -0.68 | -0.96 | -0.95 | 0.10 | -0.23 | 0.90 | 0.92 | 1.16 | 1.23 | 0.42 | 0.37 |
| Dich.0.40Load.000Cross.4Var.300Size.2Fac.0.50Fcor | 3.34 | 3.39 | 4.27 | 4.64 | -0.61 | -0.59 | -0.79 | -0.86 | 0.15 | -0.63 | 0.79 | 0.79 | 1.01 | 1.10 | 0.35 | 0.69 |
| Dich.0.40Load.000Cross.4Var.300Size.2Fac.0.70Fcor | 2.36 | 2.36 | 3.92 | 4.22 | -0.37 | -0.37 | -0.66 | -0.66 | 0.09 | -0.87 | 0.67 | 0.67 | 0.90 | 1.00 | 0.41 | 0.87 |
| Dich.0.40Load.000Cross.4Var.500Size.2Fac.0.00Fcor | 2.03 | 2.05 | 2.79 | 3.17 | -0.28 | -0.27 | -0.45 | -0.46 | 0.07 | -0.01 | 0.44 | 0.43 | 0.59 | 0.68 | 0.11 | 0.01 |
| Dich.0.40Load.000Cross.4Var.500Size.2Fac.0.50Fcor | 2.24 | 2.26 | 3.73 | 3.98 | -0.43 | -0.41 | -0.68 | -0.71 | 0.17 | -0.58 | 0.67 | 0.65 | 0.96 | 1.03 | 0.27 | 0.58 |
| Dich.0.40Load.000Cross.4Var.500Size.2Fac.0.70Fcor | 1.87 | 1.87 | 3.60 | 4.13 | -0.41 | -0.41 | -0.58 | -0.66 | 0.11 | -0.87 | 0.55 | 0.55 | 0.78 | 0.84 | 0.27 | 0.87 |
| Dich.0.40Load.000Cross.4Var.1000Size.2Fac.0.00Fcor | 0.63 | 0.63 | 1.41 | 2.05 | -0.06 | -0.06 | -0.15 | -0.28 | 0.00 | 0.00 | 0.18 | 0.18 | 0.33 | 0.46 | 0.00 | 0.00 |
| Dich.0.40Load.000Cross.4Var.1000Size.2Fac.0.50Fcor | 0.80 | 0.83 | 2.18 | 2.76 | -0.15 | -0.15 | -0.31 | -0.43 | 0.01 | -0.42 | 0.37 | 0.37 | 0.57 | 0.69 | 0.05 | 0.42 |
| Dich.0.40Load.000Cross.4Var.1000Size.2Fac.0.70Fcor | 1.14 | 1.14 | 3.42 | 4.00 | -0.38 | -0.38 | -0.64 | -0.73 | -0.23 | -0.91 | 0.58 | 0.58 | 0.84 | 0.99 | 0.29 | 0.91 |
| Dich.0.40Load.000Cross.4Var.300Size.4Fac.0.00Fcor | 9.13 | 9.08 | 10.09 | 9.79 | -1.91 | -1.93 | -2.28 | -2.03 | -0.24 | -0.88 | 2.19 | 2.21 | 2.46 | 2.31 | 0.70 | 1.10 |
| Dich.0.40Load.000Cross.4Var.300Size.4Fac.0.50Fcor | 6.92 | 6.80 | 5.82 | 7.63 | -1.43 | -1.42 | -1.75 | -1.36 | -0.48 | -2.53 | 1.63 | 1.62 | 1.81 | 1.58 | 0.72 | 2.53 |
| Dich.0.40Load.000Cross.4Var.300Size.4Fac.0.70Fcor | 4.49 | 4.60 | 4.28 | 4.93 | -1.05 | -1.00 | -1.38 | -0.85 | -0.70 | -2.88 | 1.27 | 1.26 | 1.58 | 1.11 | 0.94 | 2.88 |
| Dich.0.40Load.000Cross.4Var.500Size.4Fac.0.00Fcor | 6.20 | 6.14 | 6.90 | 6.72 | -1.15 | -1.18 | -1.52 | -1.18 | -0.09 | -0.15 | 1.29 | 1.30 | 1.66 | 1.38 | 0.41 | 0.27 |
| Dich.0.40Load.000Cross.4Var.500Size.4Fac.0.50Fcor | 4.64 | 4.61 | 4.38 | 5.10 | -0.97 | -0.98 | -1.33 | -0.82 | -0.76 | -2.42 | 1.23 | 1.22 | 1.45 | 1.18 | 0.94 | 2.42 |
| Dich.0.40Load.000Cross.4Var.500Size.4Fac.0.70Fcor | 5.19 | 5.04 | 3.77 | 5.06 | -1.11 | -1.08 | -1.47 | -0.88 | -0.97 | -2.81 | 1.37 | 1.34 | 1.55 | 1.14 | 1.07 | 2.81 |
| Dich.0.40Load.000Cross.4Var.1000Size.4Fac.0.00Fcor | 2.85 | 2.90 | 3.68 | 3.66 | -0.41 | -0.42 | -0.70 | -0.44 | 0.02 | 0.02 | 0.67 | 0.68 | 0.90 | 0.72 | 0.04 | 0.02 |
| Dich.0.40Load.000Cross.4Var.1000Size.4Fac.0.50Fcor | 4.02 | 4.30 | 3.27 | 4.43 | -0.96 | -0.99 | -1.28 | -0.82 | -0.60 | -1.92 | 1.30 | 1.33 | 1.48 | 1.22 | 0.70 | 1.92 |
| Dich.0.40Load.000Cross.4Var.1000Size.4Fac.0.70Fcor | 4.60 | 4.60 | 4.01 | 5.22 | -1.31 | -1.30 | -1.50 | -1.25 | -1.38 | -2.93 | 1.41 | 1.40 | 1.54 | 1.37 | 1.42 | 2.93 |
| Dich.0.40Load.000Cross.8Var.300Size.2Fac.0.00Fcor | 3.85 | 3.99 | 4.59 | 5.50 | 0.81 | 0.82 | 0.65 | 1.03 | 0.81 | 0.11 | 1.11 | 1.10 | 1.05 | 1.43 | 0.83 | 0.11 |
| Dich.0.40Load.000Cross.8Var.300Size.2Fac.0.50Fcor | 3.97 | 3.78 | 3.25 | 4.35 | 1.02 | 1.16 | 0.68 | 1.51 | 1.10 | -0.33 | 1.14 | 1.28 | 0.84 | 1.67 | 1.10 | 0.39 |
| Dich.0.40Load.000Cross.8Var.300Size.2Fac.0.70Fcor | 4.02 | 4.05 | 3.28 | 4.38 | 1.33 | 1.34 | 1.02 | 1.54 | 0.99 | -0.82 | 1.43 | 1.46 | 1.14 | 1.66 | 1.03 | 0.82 |
| Dich.0.40Load.000Cross.8Var.500Size.2Fac.0.00Fcor | 2.53 | 2.54 | 4.02 | 4.14 | 0.73 | 0.75 | 0.68 | 1.18 | 0.42 | 0.02 | 0.83 | 0.85 | 0.80 | 1.30 | 0.42 | 0.02 |
| Dich.0.40Load.000Cross.8Var.500Size.2Fac.0.50Fcor | 3.29 | 3.28 | 2.86 | 4.03 | 1.09 | 1.10 | 0.75 | 1.43 | 0.44 | -0.11 | 1.29 | 1.28 | 0.87 | 1.57 | 0.44 | 0.13 |
| Dich.0.40Load.000Cross.8Var.500Size.2Fac.0.70Fcor | 3.99 | 3.99 | 2.67 | 4.40 | 1.25 | 1.25 | 0.88 | 1.52 | 0.63 | -0.76 | 1.31 | 1.31 | 0.92 | 1.56 | 0.71 | 0.76 |
| Dich.0.40Load.000Cross.8Var.1000Size.2Fac.0.00Fcor | 0.26 | 0.27 | 2.93 | 3.60 | 0.12 | 0.15 | 0.50 | 0.99 | 0.02 | 0.00 | 0.14 | 0.17 | 0.50 | 0.99 | 0.02 | 0.00 |
| Dich.0.40Load.000Cross.8Var.1000Size.2Fac.0.50Fcor | 1.32 | 1.32 | 2.75 | 3.89 | 0.56 | 0.56 | 0.62 | 1.02 | 0.03 | 0.00 | 0.60 | 0.60 | 0.70 | 1.10 | 0.03 | 0.02 |
| Dich.0.40Load.000Cross.8Var.1000Size.2Fac.0.70Fcor | 2.44 | 2.47 | 1.88 | 3.93 | 0.78 | 0.78 | 0.64 | 1.06 | 0.12 | -0.57 | 0.92 | 0.92 | 0.68 | 1.14 | 0.18 | 0.57 |
| Dich.0.40Load.000Cross.8Var.300Size.4Fac.0.00Fcor | 8.61 | 8.54 | 9.63 | 9.19 | 2.44 | 2.52 | 0.62 | 2.96 | 0.71 | 0.34 | 2.78 | 2.86 | 1.82 | 3.28 | 0.89 | 0.34 |
| Dich.0.40Load.000Cross.8Var.300Size.4Fac.0.50Fcor | 9.00 | 9.06 | 5.90 | 8.84 | 2.72 | 2.67 | 0.82 | 3.33 | 0.58 | -1.66 | 2.86 | 2.81 | 1.96 | 3.39 | 1.06 | 1.70 |
| Dich.0.40Load.000Cross.8Var.300Size.4Fac.0.70Fcor | 10.34 | 10.45 | 5.33 | 8.29 | 2.95 | 2.87 | 0.93 | 3.91 | 0.43 | -2.85 | 3.19 | 3.11 | 2.03 | 4.03 | 1.15 | 2.85 |
| Dich.0.40Load.000Cross.8Var.500Size.4Fac.0.00Fcor | 6.15 | 6.30 | 9.70 | 7.68 | 1.65 | 1.72 | 0.31 | 2.41 | 0.18 | 0.15 | 1.89 | 1.94 | 1.65 | 2.59 | 0.24 | 0.15 |
| Dich.0.40Load.000Cross.8Var.500Size.4Fac.0.50Fcor | 8.07 | 7.93 | 5.64 | 8.06 | 2.07 | 2.09 | 0.44 | 2.56 | 0.13 | -0.90 | 2.41 | 2.43 | 1.62 | 2.64 | 0.67 | 0.92 |
| Dich.0.40Load.000Cross.8Var.500Size.4Fac.0.70Fcor | 10.42 | 10.60 | 6.78 | 9.62 | 2.14 | 2.15 | 0.37 | 2.92 | 0.26 | -2.76 | 2.44 | 2.43 | 1.69 | 3.02 | 1.02 | 2.76 |
| Dich.0.40Load.000Cross.8Var.1000Size.4Fac.0.00Fcor | 2.54 | 2.63 | 11.51 | 5.47 | 1.05 | 1.05 | -0.06 | 1.88 | 0.01 | 0.02 | 1.19 | 1.17 | 1.16 | 1.96 | 0.01 | 0.02 |
| Dich.0.40Load.000Cross.8Var.1000Size.4Fac.0.50Fcor | 8.21 | 8.21 | 6.66 | 7.70 | 1.10 | 1.18 | -0.19 | 1.83 | 0.11 | -0.17 | 1.66 | 1.68 | 1.47 | 1.97 | 0.27 | 0.17 |
| Dich.0.40Load.000Cross.8Var.1000Size.4Fac.0.70Fcor | 12.50 | 12.50 | 7.64 | 9.83 | 0.80 | 0.80 | 0.18 | 1.50 | -0.25 | -2.46 | 1.78 | 1.78 | 1.46 | 1.88 | 0.95 | 2.46 |
| Dich.0.40Load.125Cross.4Var.300Size.2Fac.0.00Fcor | 3.29 | 3.34 | 4.75 | 4.97 | -0.67 | -0.64 | -0.95 | -0.96 | 0.21 | -0.18 | 0.77 | 0.76 | 1.09 | 1.16 | 0.27 | 0.30 |
| Dich.0.40Load.125Cross.4Var.300Size.2Fac.0.50Fcor | 1.92 | 1.99 | 3.63 | 3.94 | -0.31 | -0.27 | -0.49 | -0.49 | 0.12 | -0.71 | 0.69 | 0.69 | 0.91 | 0.99 | 0.28 | 0.73 |
| Dich.0.40Load.125Cross.4Var.300Size.2Fac.0.70Fcor | 1.84 | 1.86 | 3.64 | 4.05 | -0.36 | -0.34 | -0.59 | -0.62 | 0.10 | -0.92 | 0.60 | 0.60 | 0.85 | 0.96 | 0.34 | 0.92 |
| Dich.0.40Load.125Cross.4Var.500Size.2Fac.0.00Fcor | 2.20 | 2.34 | 3.84 | 4.37 | -0.45 | -0.42 | -0.72 | -0.82 | 0.23 | -0.07 | 0.63 | 0.60 | 0.92 | 0.98 | 0.29 | 0.09 |
| Dich.0.40Load.125Cross.4Var.500Size.2Fac.0.50Fcor | 1.65 | 1.66 | 3.53 | 4.06 | -0.37 | -0.37 | -0.65 | -0.69 | 0.05 | -0.73 | 0.55 | 0.55 | 0.87 | 0.97 | 0.35 | 0.73 |
| Dich.0.40Load.125Cross.4Var.500Size.2Fac.0.70Fcor | 1.60 | 1.61 | 3.34 | 3.94 | -0.32 | -0.31 | -0.57 | -0.66 | -0.13 | -0.91 | 0.52 | 0.51 | 0.75 | 0.86 | 0.35 | 0.91 |
| Dich.0.40Load.125Cross.4Var.1000Size.2Fac.0.00Fcor | 1.05 | 1.11 | 2.15 | 2.98 | -0.13 | -0.13 | -0.31 | -0.46 | 0.00 | 0.00 | 0.39 | 0.39 | 0.57 | 0.74 | 0.00 | 0.00 |
| Dich.0.40Load.125Cross.4Var.1000Size.2Fac.0.50Fcor | 1.21 | 1.21 | 3.25 | 3.80 | -0.36 | -0.35 | -0.60 | -0.68 | -0.08 | -0.67 | 0.46 | 0.45 | 0.76 | 0.90 | 0.16 | 0.67 |
| Dich.0.40Load.125Cross.4Var.1000Size.2Fac.0.70Fcor | 0.83 | 0.86 | 3.92 | 4.39 | -0.46 | -0.45 | -0.76 | -0.82 | -0.49 | -0.98 | 0.60 | 0.59 | 0.92 | 1.00 | 0.55 | 0.98 |
| Dich.0.40Load.125Cross.4Var.300Size.4Fac.0.00Fcor | 8.55 | 8.55 | 8.76 | 9.24 | -1.84 | -1.88 | -2.15 | -1.92 | -0.44 | -0.80 | 2.12 | 2.16 | 2.33 | 2.20 | 0.86 | 0.96 |
| Dich.0.40Load.125Cross.4Var.300Size.4Fac.0.50Fcor | 5.04 | 5.10 | 4.60 | 5.59 | -1.02 | -1.01 | -1.43 | -0.85 | -0.59 | -2.53 | 1.46 | 1.41 | 1.57 | 1.33 | 0.97 | 2.53 |
| Dich.0.40Load.125Cross.4Var.300Size.4Fac.0.70Fcor | 4.84 | 4.82 | 4.08 | 5.29 | -0.92 | -0.93 | -1.39 | -0.66 | -0.70 | -2.93 | 1.18 | 1.19 | 1.51 | 1.10 | 0.90 | 2.93 |
| Dich.0.40Load.125Cross.4Var.500Size.4Fac.0.00Fcor | 6.50 | 6.51 | 6.75 | 7.52 | -1.41 | -1.40 | -1.73 | -1.49 | -0.08 | -0.27 | 1.61 | 1.60 | 1.83 | 1.69 | 0.54 | 0.33 |
| Dich.0.40Load.125Cross.4Var.500Size.4Fac.0.50Fcor | 5.03 | 5.13 | 4.26 | 5.66 | -1.24 | -1.17 | -1.42 | -0.91 | -0.68 | -2.56 | 1.52 | 1.51 | 1.60 | 1.37 | 0.88 | 2.56 |
| Dich.0.40Load.125Cross.4Var.500Size.4Fac.0.70Fcor | 5.01 | 5.07 | 4.02 | 5.83 | -1.21 | -1.24 | -1.63 | -1.07 | -0.98 | -2.98 | 1.51 | 1.54 | 1.71 | 1.41 | 1.12 | 2.98 |
| Dich.0.40Load.125Cross.4Var.1000Size.4Fac.0.00Fcor | 3.40 | 3.37 | 3.90 | 4.01 | -0.54 | -0.53 | -0.89 | -0.56 | -0.10 | -0.02 | 0.82 | 0.81 | 1.11 | 0.86 | 0.16 | 0.02 |
| Dich.0.40Load.125Cross.4Var.1000Size.4Fac.0.50Fcor | 4.98 | 4.97 | 4.35 | 5.85 | -1.48 | -1.41 | -1.56 | -1.26 | -0.86 | -2.07 | 1.62 | 1.63 | 1.74 | 1.50 | 0.92 | 2.07 |
| Dich.0.40Load.125Cross.4Var.1000Size.4Fac.0.70Fcor | 4.67 | 4.82 | 4.41 | 6.06 | -1.65 | -1.66 | -1.73 | -1.47 | -1.43 | -2.97 | 1.71 | 1.72 | 1.85 | 1.63 | 1.45 | 2.97 |
| Dich.0.40Load.125Cross.8Var.300Size.2Fac.0.00Fcor | 3.90 | 3.85 | 3.32 | 4.28 | 1.01 | 1.09 | 0.63 | 1.44 | 0.99 | -0.01 | 1.17 | 1.25 | 0.75 | 1.56 | 1.03 | 0.03 |
| Dich.0.40Load.125Cross.8Var.300Size.2Fac.0.50Fcor | 3.86 | 3.78 | 2.94 | 4.15 | 1.24 | 1.34 | 1.07 | 1.70 | 1.08 | -0.50 | 1.30 | 1.38 | 1.07 | 1.70 | 1.12 | 0.52 |
| Dich.0.40Load.125Cross.8Var.300Size.2Fac.0.70Fcor | 4.24 | 4.22 | 3.32 | 4.45 | 1.06 | 1.05 | 0.74 | 1.42 | 1.03 | -0.92 | 1.20 | 1.21 | 0.78 | 1.46 | 1.07 | 0.92 |
| Dich.0.40Load.125Cross.8Var.500Size.2Fac.0.00Fcor | 3.15 | 3.24 | 3.99 | 4.44 | 0.76 | 0.80 | 0.54 | 1.20 | 0.53 | 0.02 | 0.94 | 0.94 | 0.74 | 1.42 | 0.53 | 0.02 |
| Dich.0.40Load.125Cross.8Var.500Size.2Fac.0.50Fcor | 3.89 | 3.94 | 3.10 | 4.68 | 0.85 | 0.85 | 0.66 | 1.20 | 0.78 | -0.27 | 0.93 | 0.93 | 0.70 | 1.26 | 0.78 | 0.27 |
| Dich.0.40Load.125Cross.8Var.500Size.2Fac.0.70Fcor | 4.53 | 4.47 | 2.95 | 4.82 | 0.84 | 0.84 | 0.56 | 1.14 | 0.75 | -0.84 | 1.00 | 1.00 | 0.60 | 1.18 | 0.81 | 0.84 |
| Dich.0.40Load.125Cross.8Var.1000Size.2Fac.0.00Fcor | 0.89 | 0.91 | 2.78 | 3.87 | 0.48 | 0.51 | 0.74 | 1.17 | 0.19 | 0.00 | 0.50 | 0.53 | 0.74 | 1.19 | 0.19 | 0.00 |
| Dich.0.40Load.125Cross.8Var.1000Size.2Fac.0.50Fcor | 3.29 | 3.32 | 2.86 | 4.44 | 0.49 | 0.50 | 0.68 | 0.94 | 0.23 | -0.04 | 0.77 | 0.76 | 0.76 | 1.08 | 0.23 | 0.04 |
| Dich.0.40Load.125Cross.8Var.1000Size.2Fac.0.70Fcor | 3.60 | 3.60 | 2.61 | 4.48 | 0.64 | 0.64 | 0.60 | 0.79 | 0.19 | -0.85 | 0.86 | 0.86 | 0.68 | 0.97 | 0.33 | 0.85 |
| Dich.0.40Load.125Cross.8Var.300Size.4Fac.0.00Fcor | 9.14 | 9.29 | 8.84 | 9.71 | 2.40 | 2.56 | 1.13 | 2.82 | 0.72 | 0.37 | 2.82 | 2.98 | 2.25 | 3.18 | 0.94 | 0.41 |
| Dich.0.40Load.125Cross.8Var.300Size.4Fac.0.50Fcor | 10.06 | 9.90 | 6.21 | 8.57 | 2.45 | 2.44 | 0.80 | 3.36 | 0.60 | -2.03 | 2.65 | 2.64 | 1.76 | 3.38 | 1.06 | 2.03 |
| Dich.0.40Load.125Cross.8Var.300Size.4Fac.0.70Fcor | 10.78 | 10.79 | 6.44 | 8.47 | 2.17 | 2.24 | 1.04 | 3.26 | 0.40 | -2.92 | 2.47 | 2.54 | 2.06 | 3.38 | 1.16 | 2.92 |
| Dich.0.40Load.125Cross.8Var.500Size.4Fac.0.00Fcor | 5.63 | 5.69 | 7.00 | 6.61 | 2.21 | 2.16 | 0.95 | 2.75 | 0.19 | 0.05 | 2.45 | 2.44 | 2.07 | 2.89 | 0.27 | 0.05 |
| Dich.0.40Load.125Cross.8Var.500Size.4Fac.0.50Fcor | 10.98 | 10.96 | 7.38 | 9.67 | 1.58 | 1.59 | 0.62 | 2.15 | 0.35 | -1.28 | 1.90 | 1.93 | 1.60 | 2.21 | 0.91 | 1.30 |
| Dich.0.40Load.125Cross.8Var.500Size.4Fac.0.70Fcor | 12.21 | 12.21 | 9.19 | 10.41 | 1.40 | 1.40 | 0.11 | 2.09 | 0.05 | -2.87 | 1.96 | 1.96 | 1.43 | 2.33 | 0.87 | 2.87 |
| Dich.0.40Load.125Cross.8Var.1000Size.4Fac.0.00Fcor | 3.11 | 3.11 | 8.49 | 5.92 | 1.19 | 1.19 | 0.22 | 1.91 | 0.04 | 0.01 | 1.33 | 1.33 | 1.44 | 1.97 | 0.04 | 0.01 |
| Dich.0.40Load.125Cross.8Var.1000Size.4Fac.0.50Fcor | 9.89 | 9.89 | 7.37 | 10.44 | 0.50 | 0.50 | -0.71 | 1.03 | 0.14 | -0.27 | 1.42 | 1.42 | 1.35 | 1.53 | 0.54 | 0.29 |
| Dich.0.40Load.125Cross.8Var.1000Size.4Fac.0.70Fcor | 11.79 | 11.79 | 8.50 | 11.09 | 0.21 | 0.21 | -0.48 | 0.63 | -0.34 | -2.73 | 1.45 | 1.45 | 1.28 | 1.49 | 0.84 | 2.73 |
| Dich.0.40Load.Cross250.4Var.300Size.2Fac.0.00Fcor | 2.91 | 2.91 | 4.38 | 4.66 | -0.53 | -0.53 | -0.84 | -0.86 | 0.22 | -0.40 | 0.69 | 0.69 | 1.00 | 1.06 | 0.30 | 0.48 |
| Dich.0.40Load.Cross250.4Var.300Size.2Fac.0.50Fcor | 1.61 | 1.59 | 3.76 | 4.34 | -0.35 | -0.29 | -0.69 | -0.76 | 0.06 | -0.82 | 0.59 | 0.53 | 0.87 | 1.02 | 0.28 | 0.82 |
| Dich.0.40Load.Cross250.4Var.300Size.2Fac.0.70Fcor | 1.45 | 1.44 | 3.77 | 4.30 | -0.35 | -0.33 | -0.63 | -0.69 | -0.09 | -0.94 | 0.65 | 0.65 | 0.89 | 1.03 | 0.43 | 0.96 |
| Dich.0.40Load.Cross250.4Var.500Size.2Fac.0.00Fcor | 2.44 | 2.49 | 3.91 | 4.41 | -0.38 | -0.36 | -0.65 | -0.73 | 0.34 | -0.12 | 0.68 | 0.68 | 0.95 | 1.03 | 0.34 | 0.16 |
| Dich.0.40Load.Cross250.4Var.500Size.2Fac.0.50Fcor | 1.51 | 1.50 | 3.20 | 3.80 | -0.39 | -0.35 | -0.56 | -0.60 | -0.01 | -0.74 | 0.59 | 0.57 | 0.76 | 0.90 | 0.23 | 0.74 |
| Dich.0.40Load.Cross250.4Var.500Size.2Fac.0.70Fcor | 1.41 | 1.49 | 3.81 | 4.54 | -0.49 | -0.49 | -0.71 | -0.84 | -0.35 | -1.00 | 0.53 | 0.53 | 0.85 | 0.96 | 0.45 | 1.00 |
| Dich.0.40Load.Cross250.4Var.1000Size.2Fac.0.00Fcor | 1.14 | 1.19 | 2.42 | 3.02 | -0.19 | -0.19 | -0.36 | -0.40 | 0.14 | -0.02 | 0.39 | 0.41 | 0.60 | 0.72 | 0.14 | 0.02 |
| Dich.0.40Load.Cross250.4Var.1000Size.2Fac.0.50Fcor | 0.95 | 0.97 | 4.03 | 4.61 | -0.53 | -0.53 | -0.85 | -0.98 | -0.23 | -0.78 | 0.61 | 0.61 | 0.91 | 1.06 | 0.29 | 0.78 |
| Dich.0.40Load.Cross250.4Var.1000Size.2Fac.0.70Fcor | 0.68 | 0.68 | 4.67 | 5.18 | -0.56 | -0.55 | -0.99 | -1.10 | -0.70 | -0.99 | 0.68 | 0.67 | 1.13 | 1.26 | 0.70 | 0.99 |
| Dich.0.40Load.Cross250.4Var.300Size.4Fac.0.00Fcor | 8.17 | 8.11 | 8.18 | 8.79 | -1.80 | -1.80 | -2.04 | -1.81 | -0.44 | -0.74 | 1.94 | 1.94 | 2.14 | 1.97 | 0.92 | 0.88 |
| Dich.0.40Load.Cross250.4Var.300Size.4Fac.0.50Fcor | 4.99 | 5.26 | 3.93 | 5.42 | -1.06 | -1.08 | -1.27 | -0.81 | -0.70 | -2.70 | 1.34 | 1.38 | 1.51 | 1.19 | 0.98 | 2.70 |
| Dich.0.40Load.Cross250.4Var.300Size.4Fac.0.70Fcor | 4.93 | 5.02 | 3.64 | 5.17 | -1.27 | -1.27 | -1.35 | -0.93 | -0.95 | -2.91 | 1.45 | 1.45 | 1.51 | 1.27 | 1.09 | 2.91 |
| Dich.0.40Load.Cross250.4Var.500Size.4Fac.0.00Fcor | 5.57 | 5.62 | 5.64 | 6.23 | -1.12 | -1.10 | -1.49 | -1.09 | -0.32 | -0.23 | 1.40 | 1.38 | 1.67 | 1.37 | 0.66 | 0.33 |
| Dich.0.40Load.Cross250.4Var.500Size.4Fac.0.50Fcor | 5.20 | 5.14 | 4.46 | 5.69 | -1.29 | -1.24 | -1.45 | -1.04 | -0.87 | -2.60 | 1.45 | 1.40 | 1.57 | 1.34 | 0.97 | 2.60 |
| Dich.0.40Load.Cross250.4Var.500Size.4Fac.0.70Fcor | 5.10 | 5.06 | 4.43 | 5.53 | -1.33 | -1.34 | -1.44 | -1.08 | -1.21 | -2.94 | 1.51 | 1.52 | 1.58 | 1.28 | 1.27 | 2.94 |
| Dich.0.40Load.Cross250.4Var.1000Size.4Fac.0.00Fcor | 3.77 | 3.72 | 4.12 | 4.40 | -0.71 | -0.71 | -1.02 | -0.64 | -0.27 | -0.05 | 1.07 | 1.07 | 1.32 | 1.06 | 0.41 | 0.09 |
| Dich.0.40Load.Cross250.4Var.1000Size.4Fac.0.50Fcor | 4.24 | 4.25 | 3.77 | 4.71 | -1.36 | -1.35 | -1.38 | -1.09 | -1.04 | -2.39 | 1.46 | 1.45 | 1.52 | 1.31 | 1.16 | 2.39 |
| Dich.0.40Load.Cross250.4Var.1000Size.4Fac.0.70Fcor | 4.44 | 4.36 | 5.03 | 6.34 | -1.96 | -1.95 | -1.90 | -1.78 | -1.52 | -2.98 | 1.98 | 1.97 | 1.92 | 1.80 | 1.54 | 2.98 |
| Dich.0.40Load.Cross250.8Var.300Size.2Fac.0.00Fcor | 3.90 | 3.85 | 3.32 | 4.28 | 1.01 | 1.09 | 0.63 | 1.44 | 0.99 | -0.01 | 1.17 | 1.25 | 0.75 | 1.56 | 1.03 | 0.03 |
| Dich.0.40Load.Cross250.8Var.300Size.2Fac.0.50Fcor | 3.86 | 3.78 | 2.94 | 4.15 | 1.24 | 1.34 | 1.07 | 1.70 | 1.08 | -0.50 | 1.30 | 1.38 | 1.07 | 1.70 | 1.12 | 0.52 |
| Dich.0.40Load.Cross250.8Var.300Size.2Fac.0.70Fcor | 4.24 | 4.22 | 3.32 | 4.45 | 1.06 | 1.05 | 0.74 | 1.42 | 1.03 | -0.92 | 1.20 | 1.21 | 0.78 | 1.46 | 1.07 | 0.92 |
| Dich.0.40Load.Cross250.8Var.500Size.2Fac.0.00Fcor | 3.15 | 3.24 | 3.99 | 4.44 | 0.76 | 0.80 | 0.54 | 1.20 | 0.53 | 0.02 | 0.94 | 0.94 | 0.74 | 1.42 | 0.53 | 0.02 |
| Dich.0.40Load.Cross250.8Var.500Size.2Fac.0.50Fcor | 3.89 | 3.94 | 3.10 | 4.68 | 0.85 | 0.85 | 0.66 | 1.20 | 0.78 | -0.27 | 0.93 | 0.93 | 0.70 | 1.26 | 0.78 | 0.27 |
| Dich.0.40Load.Cross250.8Var.500Size.2Fac.0.70Fcor | 4.53 | 4.47 | 2.95 | 4.82 | 0.84 | 0.84 | 0.56 | 1.14 | 0.75 | -0.84 | 1.00 | 1.00 | 0.60 | 1.18 | 0.81 | 0.84 |
| Dich.0.40Load.Cross250.8Var.1000Size.2Fac.0.00Fcor | 0.89 | 0.91 | 2.78 | 3.87 | 0.48 | 0.51 | 0.74 | 1.17 | 0.19 | 0.00 | 0.50 | 0.53 | 0.74 | 1.19 | 0.19 | 0.00 |
| Dich.0.40Load.Cross250.8Var.1000Size.2Fac.0.50Fcor | 3.29 | 3.32 | 2.86 | 4.44 | 0.49 | 0.50 | 0.68 | 0.94 | 0.23 | -0.04 | 0.77 | 0.76 | 0.76 | 1.08 | 0.23 | 0.04 |
| Dich.0.40Load.Cross250.8Var.1000Size.2Fac.0.70Fcor | 3.60 | 3.60 | 2.61 | 4.48 | 0.64 | 0.64 | 0.60 | 0.79 | 0.19 | -0.85 | 0.86 | 0.86 | 0.68 | 0.97 | 0.33 | 0.85 |
| Dich.0.40Load.Cross250.8Var.300Size.4Fac.0.00Fcor | 9.49 | 9.67 | 7.54 | 9.42 | 2.64 | 2.68 | 1.06 | 3.20 | 0.76 | 0.11 | 3.04 | 3.04 | 2.18 | 3.56 | 1.12 | 0.21 |
| Dich.0.40Load.Cross250.8Var.300Size.4Fac.0.50Fcor | 9.83 | 9.54 | 6.26 | 8.13 | 2.43 | 2.47 | 1.26 | 3.27 | 0.38 | -1.99 | 2.67 | 2.73 | 2.10 | 3.43 | 1.12 | 1.99 |
| Dich.0.40Load.Cross250.8Var.300Size.4Fac.0.70Fcor | 10.76 | 10.41 | 7.72 | 9.28 | 2.24 | 2.34 | 0.89 | 2.87 | 0.53 | -2.91 | 2.60 | 2.66 | 1.75 | 3.03 | 1.07 | 2.91 |
| Dich.0.40Load.Cross250.8Var.500Size.4Fac.0.00Fcor | 6.82 | 6.97 | 6.69 | 7.18 | 2.48 | 2.69 | 1.04 | 3.23 | 0.36 | 0.06 | 2.72 | 2.89 | 2.14 | 3.35 | 0.48 | 0.06 |
| Dich.0.40Load.Cross250.8Var.500Size.4Fac.0.50Fcor | 11.79 | 11.92 | 9.22 | 10.53 | 1.46 | 1.38 | 0.24 | 1.86 | 0.32 | -1.71 | 1.92 | 1.86 | 1.54 | 2.10 | 0.96 | 1.71 |
| Dich.0.40Load.Cross250.8Var.500Size.4Fac.0.70Fcor | 12.17 | 12.01 | 8.64 | 10.20 | 1.02 | 1.09 | 0.09 | 1.83 | 0.16 | -2.95 | 1.82 | 1.85 | 1.51 | 2.11 | 0.98 | 2.95 |
| Dich.0.40Load.Cross250.8Var.1000Size.4Fac.0.00Fcor | 4.20 | 4.19 | 6.68 | 6.12 | 1.57 | 1.56 | 0.34 | 2.22 | 0.13 | 0.01 | 1.71 | 1.68 | 1.52 | 2.30 | 0.13 | 0.01 |
| Dich.0.40Load.Cross250.8Var.1000Size.4Fac.0.50Fcor | 9.53 | 9.53 | 7.25 | 9.74 | 0.68 | 0.68 | -0.57 | 1.32 | 0.09 | -0.44 | 1.56 | 1.56 | 1.37 | 1.70 | 0.67 | 0.44 |
| Dich.0.40Load.Cross250.8Var.1000Size.4Fac.0.70Fcor | 11.15 | 11.15 | 8.27 | 10.22 | 0.33 | 0.33 | 0.09 | 1.00 | -0.32 | -2.79 | 1.59 | 1.59 | 1.55 | 1.66 | 0.94 | 2.79 |
| Dich.0.55Load.000Cross.4Var.300Size.2Fac.0.00Fcor | 0.29 | 0.30 | 0.77 | 1.29 | 0.09 | 0.08 | 0.14 | 0.13 | 0.03 | 0.00 | 0.15 | 0.14 | 0.22 | 0.35 | 0.03 | 0.00 |
| Dich.0.55Load.000Cross.4Var.300Size.2Fac.0.50Fcor | 0.64 | 0.64 | 1.03 | 1.76 | 0.02 | 0.03 | 0.10 | 0.10 | 0.06 | -0.43 | 0.20 | 0.17 | 0.26 | 0.46 | 0.10 | 0.43 |
| Dich.0.55Load.000Cross.4Var.300Size.2Fac.0.70Fcor | 0.87 | 0.87 | 1.49 | 2.41 | 0.02 | 0.02 | 0.01 | 0.00 | -0.22 | -0.88 | 0.40 | 0.40 | 0.49 | 0.78 | 0.34 | 0.88 |
| Dich.0.55Load.000Cross.4Var.500Size.2Fac.0.00Fcor | 0.02 | 0.02 | 0.42 | 0.96 | 0.03 | 0.03 | 0.20 | 0.17 | 0.00 | 0.00 | 0.03 | 0.03 | 0.20 | 0.33 | 0.00 | 0.00 |
| Dich.0.55Load.000Cross.4Var.500Size.2Fac.0.50Fcor | 0.46 | 0.48 | 0.73 | 1.54 | 0.00 | 0.00 | 0.05 | -0.02 | -0.01 | -0.20 | 0.14 | 0.14 | 0.21 | 0.38 | 0.01 | 0.20 |
| Dich.0.55Load.000Cross.4Var.500Size.2Fac.0.70Fcor | 0.67 | 0.75 | 0.97 | 2.02 | 0.05 | 0.03 | 0.10 | -0.01 | -0.29 | -0.89 | 0.31 | 0.31 | 0.34 | 0.63 | 0.29 | 0.89 |
| Dich.0.55Load.000Cross.4Var.1000Size.2Fac.0.00Fcor | 0.00 | 0.00 | 0.22 | 0.88 | 0.00 | 0.00 | 0.13 | 0.02 | 0.00 | 0.00 | 0.00 | 0.00 | 0.13 | 0.30 | 0.00 | 0.00 |
| Dich.0.55Load.000Cross.4Var.1000Size.2Fac.0.50Fcor | 0.06 | 0.06 | 0.28 | 0.91 | 0.02 | 0.02 | 0.07 | -0.01 | 0.00 | -0.08 | 0.04 | 0.04 | 0.11 | 0.25 | 0.00 | 0.08 |
| Dich.0.55Load.000Cross.4Var.1000Size.2Fac.0.70Fcor | 0.53 | 0.53 | 1.09 | 1.82 | -0.06 | -0.06 | -0.10 | -0.18 | -0.24 | -0.82 | 0.20 | 0.20 | 0.30 | 0.48 | 0.24 | 0.82 |
| Dich.0.55Load.000Cross.4Var.300Size.4Fac.0.00Fcor | 1.44 | 1.46 | 4.43 | 2.24 | -0.05 | -0.05 | -0.80 | 0.10 | 0.00 | 0.00 | 0.33 | 0.33 | 0.96 | 0.48 | 0.00 | 0.02 |
| Dich.0.55Load.000Cross.4Var.300Size.4Fac.0.50Fcor | 2.65 | 2.67 | 1.85 | 2.97 | -0.37 | -0.36 | -1.05 | 0.00 | -0.37 | -1.69 | 0.85 | 0.84 | 1.27 | 0.68 | 0.55 | 1.71 |
| Dich.0.55Load.000Cross.4Var.300Size.4Fac.0.70Fcor | 3.19 | 3.19 | 2.13 | 3.35 | -0.25 | -0.25 | -0.85 | 0.12 | -1.02 | -2.91 | 1.01 | 1.01 | 1.37 | 0.96 | 1.06 | 2.91 |
| Dich.0.55Load.000Cross.4Var.500Size.4Fac.0.00Fcor | 0.07 | 0.08 | 2.94 | 1.08 | 0.00 | -0.02 | -0.68 | 0.24 | 0.00 | 0.00 | 0.06 | 0.06 | 0.84 | 0.30 | 0.00 | 0.00 |
| Dich.0.55Load.000Cross.4Var.500Size.4Fac.0.50Fcor | 1.56 | 1.53 | 1.08 | 2.21 | -0.11 | -0.10 | -1.15 | 0.17 | -0.32 | -1.23 | 0.61 | 0.60 | 1.35 | 0.65 | 0.34 | 1.23 |
| Dich.0.55Load.000Cross.4Var.500Size.4Fac.0.70Fcor | 3.17 | 3.10 | 1.66 | 3.39 | -0.61 | -0.56 | -1.05 | -0.36 | -1.09 | -2.88 | 0.99 | 1.00 | 1.23 | 0.90 | 1.13 | 2.88 |
| Dich.0.55Load.000Cross.4Var.1000Size.4Fac.0.00Fcor | 0.00 | 0.00 | 1.15 | 0.82 | 0.00 | 0.00 | -0.51 | 0.20 | 0.00 | 0.00 | 0.00 | 0.00 | 0.61 | 0.26 | 0.00 | 0.00 |
| Dich.0.55Load.000Cross.4Var.1000Size.4Fac.0.50Fcor | 0.61 | 0.61 | 0.92 | 1.48 | -0.02 | -0.02 | -1.26 | 0.07 | -0.04 | -0.44 | 0.18 | 0.18 | 1.28 | 0.31 | 0.04 | 0.44 |
| Dich.0.55Load.000Cross.4Var.1000Size.4Fac.0.70Fcor | 2.33 | 2.33 | 1.18 | 3.12 | -0.52 | -0.52 | -1.12 | -0.39 | -0.75 | -2.81 | 0.84 | 0.84 | 1.32 | 0.85 | 0.75 | 2.81 |
| Dich.0.55Load.000Cross.8Var.300Size.2Fac.0.00Fcor | 0.20 | 0.21 | 2.85 | 2.72 | 0.22 | 0.24 | 1.03 | 1.77 | 0.00 | 0.00 | 0.24 | 0.26 | 1.03 | 1.77 | 0.00 | 0.00 |
| Dich.0.55Load.000Cross.8Var.300Size.2Fac.0.50Fcor | 0.97 | 0.93 | 1.44 | 2.52 | 0.82 | 0.87 | 0.98 | 2.12 | 0.10 | -0.02 | 0.82 | 0.87 | 0.98 | 2.12 | 0.10 | 0.02 |
| Dich.0.55Load.000Cross.8Var.300Size.2Fac.0.70Fcor | 2.20 | 2.22 | 1.28 | 2.82 | 1.52 | 1.50 | 0.86 | 2.19 | 0.37 | -0.55 | 1.54 | 1.52 | 0.86 | 2.19 | 0.41 | 0.55 |
| Dich.0.55Load.000Cross.8Var.500Size.2Fac.0.00Fcor | 0.01 | 0.04 | 2.60 | 2.82 | 0.05 | 0.04 | 0.99 | 1.62 | 0.00 | 0.00 | 0.05 | 0.04 | 0.99 | 1.62 | 0.00 | 0.00 |
| Dich.0.55Load.000Cross.8Var.500Size.2Fac.0.50Fcor | 0.30 | 0.31 | 2.12 | 2.97 | 0.50 | 0.52 | 0.73 | 1.73 | 0.00 | 0.00 | 0.50 | 0.52 | 0.73 | 1.73 | 0.00 | 0.00 |
| Dich.0.55Load.000Cross.8Var.500Size.2Fac.0.70Fcor | 1.22 | 1.30 | 1.59 | 2.44 | 1.12 | 1.12 | 1.06 | 2.09 | 0.06 | -0.24 | 1.14 | 1.14 | 1.06 | 2.09 | 0.06 | 0.24 |
| Dich.0.55Load.000Cross.8Var.1000Size.2Fac.0.00Fcor | 0.00 | 0.00 | 1.80 | 2.41 | 0.00 | 0.00 | 0.94 | 1.45 | 0.00 | 0.00 | 0.00 | 0.00 | 0.94 | 1.45 | 0.00 | 0.00 |
| Dich.0.55Load.000Cross.8Var.1000Size.2Fac.0.50Fcor | 0.06 | 0.06 | 2.11 | 3.07 | 0.01 | 0.01 | 0.78 | 1.41 | 0.00 | 0.00 | 0.01 | 0.01 | 0.78 | 1.41 | 0.00 | 0.00 |
| Dich.0.55Load.000Cross.8Var.1000Size.2Fac.0.70Fcor | 0.59 | 0.59 | 1.38 | 2.66 | 0.43 | 0.43 | 0.59 | 1.25 | 0.00 | -0.04 | 0.45 | 0.45 | 0.59 | 1.29 | 0.00 | 0.04 |
| Dich.0.55Load.000Cross.8Var.300Size.4Fac.0.00Fcor | 0.72 | 0.69 | 10.17 | 5.16 | 0.42 | 0.39 | -0.01 | 2.55 | 0.00 | 0.00 | 0.52 | 0.51 | 1.37 | 2.55 | 0.00 | 0.00 |
| Dich.0.55Load.000Cross.8Var.300Size.4Fac.0.50Fcor | 4.09 | 4.15 | 3.52 | 5.00 | 2.46 | 2.43 | 0.43 | 3.69 | 0.04 | -0.19 | 2.60 | 2.57 | 1.65 | 3.71 | 0.10 | 0.19 |
| Dich.0.55Load.000Cross.8Var.300Size.4Fac.0.70Fcor | 6.01 | 6.01 | 3.87 | 5.54 | 3.58 | 3.58 | 0.67 | 4.29 | -0.04 | -2.43 | 3.70 | 3.70 | 1.85 | 4.35 | 0.58 | 2.43 |
| Dich.0.55Load.000Cross.8Var.500Size.4Fac.0.00Fcor | 0.01 | 0.03 | 8.26 | 4.41 | 0.02 | 0.01 | 0.14 | 2.64 | 0.00 | 0.00 | 0.02 | 0.01 | 1.48 | 2.64 | 0.00 | 0.00 |
| Dich.0.55Load.000Cross.8Var.500Size.4Fac.0.50Fcor | 1.89 | 1.89 | 2.23 | 3.73 | 1.22 | 1.23 | -0.62 | 3.23 | 0.01 | 0.00 | 1.36 | 1.37 | 1.28 | 3.23 | 0.01 | 0.00 |
| Dich.0.55Load.000Cross.8Var.500Size.4Fac.0.70Fcor | 4.52 | 4.52 | 2.65 | 4.45 | 2.90 | 2.90 | 0.38 | 3.96 | -0.04 | -1.71 | 3.08 | 3.08 | 1.74 | 3.96 | 0.22 | 1.71 |
| Dich.0.55Load.000Cross.8Var.1000Size.4Fac.0.00Fcor | 0.00 | 0.00 | 6.11 | 4.02 | 0.00 | 0.00 | -0.40 | 1.98 | 0.00 | 0.00 | 0.00 | 0.00 | 1.38 | 1.98 | 0.00 | 0.00 |
| Dich.0.55Load.000Cross.8Var.1000Size.4Fac.0.50Fcor | 0.34 | 0.34 | 1.27 | 3.74 | 0.23 | 0.23 | -0.76 | 2.03 | 0.00 | 0.00 | 0.23 | 0.23 | 1.32 | 2.03 | 0.00 | 0.00 |
| Dich.0.55Load.000Cross.8Var.1000Size.4Fac.0.70Fcor | 2.26 | 2.29 | 1.40 | 3.92 | 1.64 | 1.65 | -0.15 | 3.00 | 0.00 | -0.62 | 1.78 | 1.79 | 1.57 | 3.00 | 0.02 | 0.62 |
| Dich.0.55Load.125Cross.4Var.300Size.2Fac.0.00Fcor | 0.64 | 0.70 | 1.19 | 1.96 | 0.04 | 0.03 | -0.03 | -0.04 | 0.06 | 0.00 | 0.26 | 0.27 | 0.29 | 0.50 | 0.06 | 0.00 |
| Dich.0.55Load.125Cross.4Var.300Size.2Fac.0.50Fcor | 0.60 | 0.64 | 0.67 | 1.86 | 0.05 | 0.06 | 0.16 | 0.14 | 0.02 | -0.53 | 0.21 | 0.20 | 0.20 | 0.44 | 0.16 | 0.53 |
| Dich.0.55Load.125Cross.4Var.300Size.2Fac.0.70Fcor | 0.83 | 0.84 | 1.55 | 2.66 | 0.02 | 0.05 | -0.07 | -0.14 | -0.41 | -0.94 | 0.32 | 0.33 | 0.41 | 0.56 | 0.53 | 0.94 |
| Dich.0.55Load.125Cross.4Var.500Size.2Fac.0.00Fcor | 0.35 | 0.38 | 0.62 | 1.61 | 0.01 | 0.04 | 0.06 | 0.01 | 0.03 | 0.00 | 0.13 | 0.16 | 0.18 | 0.41 | 0.03 | 0.00 |
| Dich.0.55Load.125Cross.4Var.500Size.2Fac.0.50Fcor | 0.53 | 0.53 | 1.03 | 1.86 | -0.03 | -0.03 | -0.04 | 0.01 | -0.04 | -0.43 | 0.19 | 0.19 | 0.24 | 0.49 | 0.10 | 0.43 |
| Dich.0.55Load.125Cross.4Var.500Size.2Fac.0.70Fcor | 0.95 | 0.95 | 2.19 | 3.16 | -0.19 | -0.17 | -0.35 | -0.46 | -0.53 | -0.93 | 0.33 | 0.33 | 0.49 | 0.66 | 0.55 | 0.93 |
| Dich.0.55Load.125Cross.4Var.1000Size.2Fac.0.00Fcor | 0.02 | 0.02 | 0.17 | 1.03 | 0.03 | 0.04 | 0.10 | 0.08 | 0.00 | 0.00 | 0.03 | 0.04 | 0.10 | 0.26 | 0.00 | 0.00 |
| Dich.0.55Load.125Cross.4Var.1000Size.2Fac.0.50Fcor | 0.26 | 0.26 | 0.57 | 1.84 | 0.03 | 0.03 | -0.01 | -0.07 | -0.05 | -0.29 | 0.11 | 0.11 | 0.15 | 0.41 | 0.05 | 0.29 |
| Dich.0.55Load.125Cross.4Var.1000Size.2Fac.0.70Fcor | 1.07 | 1.11 | 2.83 | 3.41 | -0.27 | -0.27 | -0.51 | -0.56 | -0.74 | -0.98 | 0.35 | 0.35 | 0.65 | 0.72 | 0.74 | 0.98 |
| Dich.0.55Load.125Cross.4Var.300Size.4Fac.0.00Fcor | 1.61 | 1.66 | 3.46 | 2.52 | -0.18 | -0.18 | -0.95 | 0.04 | -0.08 | -0.03 | 0.58 | 0.58 | 1.01 | 0.64 | 0.16 | 0.03 |
| Dich.0.55Load.125Cross.4Var.300Size.4Fac.0.50Fcor | 2.29 | 2.28 | 2.10 | 3.23 | -0.44 | -0.45 | -1.09 | 0.02 | -0.63 | -2.08 | 1.02 | 1.03 | 1.29 | 0.80 | 0.79 | 2.08 |
| Dich.0.55Load.125Cross.4Var.300Size.4Fac.0.70Fcor | 3.22 | 3.29 | 2.05 | 3.54 | -0.51 | -0.53 | -1.26 | -0.09 | -1.12 | -2.97 | 1.07 | 1.09 | 1.50 | 0.93 | 1.18 | 2.97 |
| Dich.0.55Load.125Cross.4Var.500Size.4Fac.0.00Fcor | 0.43 | 0.45 | 0.81 | 1.71 | -0.05 | -0.07 | -0.88 | 0.14 | -0.07 | 0.00 | 0.21 | 0.21 | 1.04 | 0.44 | 0.07 | 0.00 |
| Dich.0.55Load.125Cross.4Var.500Size.4Fac.0.50Fcor | 1.90 | 1.86 | 1.12 | 2.72 | -0.38 | -0.37 | -1.12 | 0.05 | -0.48 | -1.68 | 0.88 | 0.89 | 1.40 | 0.79 | 0.52 | 1.68 |
| Dich.0.55Load.125Cross.4Var.500Size.4Fac.0.70Fcor | 3.49 | 3.46 | 2.03 | 4.17 | -1.03 | -0.99 | -1.52 | -0.60 | -0.92 | -2.94 | 1.23 | 1.23 | 1.52 | 0.90 | 1.04 | 2.94 |
| Dich.0.55Load.125Cross.4Var.1000Size.4Fac.0.00Fcor | 0.01 | 0.02 | 0.29 | 1.29 | -0.03 | -0.04 | -1.24 | 0.09 | -0.05 | 0.00 | 0.03 | 0.04 | 1.28 | 0.27 | 0.05 | 0.00 |
| Dich.0.55Load.125Cross.4Var.1000Size.4Fac.0.50Fcor | 0.69 | 0.69 | 0.72 | 1.75 | -0.19 | -0.19 | -1.40 | 0.09 | -0.27 | -0.94 | 0.53 | 0.53 | 1.52 | 0.57 | 0.31 | 0.94 |
| Dich.0.55Load.125Cross.4Var.1000Size.4Fac.0.70Fcor | 3.07 | 3.06 | 2.14 | 3.81 | -1.23 | -1.23 | -1.53 | -0.95 | -1.04 | -2.92 | 1.29 | 1.29 | 1.57 | 1.13 | 1.04 | 2.92 |
| Dich.0.55Load.125Cross.8Var.300Size.2Fac.0.00Fcor | 0.86 | 0.86 | 1.85 | 2.64 | 0.49 | 0.52 | 0.97 | 2.09 | 0.22 | 0.00 | 0.49 | 0.52 | 0.97 | 2.09 | 0.22 | 0.00 |
| Dich.0.55Load.125Cross.8Var.300Size.2Fac.0.50Fcor | 1.84 | 1.79 | 1.58 | 3.36 | 1.23 | 1.25 | 0.91 | 2.06 | 0.18 | -0.09 | 1.23 | 1.25 | 0.91 | 2.06 | 0.18 | 0.09 |
| Dich.0.55Load.125Cross.8Var.300Size.2Fac.0.70Fcor | 2.79 | 2.95 | 1.78 | 3.49 | 1.39 | 1.36 | 0.92 | 2.05 | 0.40 | -0.76 | 1.45 | 1.42 | 0.92 | 2.05 | 0.46 | 0.76 |
| Dich.0.55Load.125Cross.8Var.500Size.2Fac.0.00Fcor | 0.35 | 0.34 | 1.85 | 2.67 | 0.23 | 0.25 | 0.80 | 1.76 | 0.16 | 0.00 | 0.23 | 0.25 | 0.80 | 1.76 | 0.16 | 0.00 |
| Dich.0.55Load.125Cross.8Var.500Size.2Fac.0.50Fcor | 1.35 | 1.39 | 1.84 | 2.62 | 0.73 | 0.75 | 1.00 | 2.22 | 0.06 | 0.00 | 0.81 | 0.83 | 1.00 | 2.22 | 0.06 | 0.00 |
| Dich.0.55Load.125Cross.8Var.500Size.2Fac.0.70Fcor | 2.76 | 2.82 | 1.44 | 3.72 | 0.88 | 0.89 | 0.73 | 1.57 | 0.15 | -0.50 | 1.02 | 1.03 | 0.73 | 1.59 | 0.15 | 0.50 |
| Dich.0.55Load.125Cross.8Var.1000Size.2Fac.0.00Fcor | 0.06 | 0.16 | 1.87 | 2.91 | 0.02 | 0.03 | 0.55 | 1.35 | 0.21 | 0.00 | 0.02 | 0.03 | 0.55 | 1.37 | 0.21 | 0.00 |
| Dich.0.55Load.125Cross.8Var.1000Size.2Fac.0.50Fcor | 0.33 | 0.30 | 1.65 | 3.18 | 0.13 | 0.14 | 0.77 | 1.27 | 0.03 | 0.00 | 0.15 | 0.16 | 0.81 | 1.31 | 0.03 | 0.00 |
| Dich.0.55Load.125Cross.8Var.1000Size.2Fac.0.70Fcor | 1.14 | 1.13 | 2.55 | 3.12 | 0.29 | 0.29 | 0.62 | 1.11 | 0.05 | -0.17 | 0.35 | 0.35 | 0.62 | 1.11 | 0.05 | 0.17 |
| Dich.0.55Load.125Cross.8Var.300Size.4Fac.0.00Fcor | 1.17 | 1.26 | 7.54 | 5.07 | 0.99 | 0.98 | 0.22 | 2.89 | 0.04 | 0.00 | 1.11 | 1.06 | 1.42 | 2.89 | 0.04 | 0.00 |
| Dich.0.55Load.125Cross.8Var.300Size.4Fac.0.50Fcor | 4.43 | 4.49 | 4.25 | 6.60 | 2.08 | 2.10 | 0.33 | 3.15 | 0.25 | -0.41 | 2.40 | 2.42 | 1.69 | 3.19 | 0.49 | 0.41 |
| Dich.0.55Load.125Cross.8Var.300Size.4Fac.0.70Fcor | 7.60 | 7.61 | 4.67 | 7.35 | 2.64 | 2.67 | 1.24 | 3.68 | 0.02 | -2.72 | 2.84 | 2.87 | 2.00 | 3.68 | 0.76 | 2.72 |
| Dich.0.55Load.125Cross.8Var.500Size.4Fac.0.00Fcor | 0.20 | 0.21 | 6.18 | 5.28 | 0.14 | 0.14 | -0.47 | 2.53 | 0.00 | 0.00 | 0.14 | 0.14 | 1.39 | 2.53 | 0.00 | 0.00 |
| Dich.0.55Load.125Cross.8Var.500Size.4Fac.0.50Fcor | 1.99 | 1.98 | 2.72 | 4.49 | 1.72 | 1.76 | -0.36 | 3.41 | 0.09 | -0.02 | 1.78 | 1.80 | 1.62 | 3.41 | 0.11 | 0.02 |
| Dich.0.55Load.125Cross.8Var.500Size.4Fac.0.70Fcor | 6.09 | 6.17 | 3.64 | 6.10 | 2.63 | 2.66 | 0.45 | 3.17 | -0.02 | -2.19 | 2.71 | 2.72 | 1.53 | 3.17 | 0.42 | 2.19 |
| Dich.0.55Load.125Cross.8Var.1000Size.4Fac.0.00Fcor | 0.00 | 0.00 | 2.04 | 4.39 | 0.00 | 0.00 | -0.82 | 2.13 | 0.02 | 0.00 | 0.00 | 0.00 | 1.32 | 2.13 | 0.02 | 0.00 |
| Dich.0.55Load.125Cross.8Var.1000Size.4Fac.0.50Fcor | 0.80 | 0.79 | 2.48 | 5.04 | 0.46 | 0.46 | -0.62 | 2.62 | 0.05 | 0.00 | 0.58 | 0.58 | 1.34 | 2.62 | 0.05 | 0.00 |
| Dich.0.55Load.125Cross.8Var.1000Size.4Fac.0.70Fcor | 4.62 | 4.69 | 3.63 | 5.45 | 1.04 | 1.03 | -0.38 | 2.42 | -0.08 | -1.20 | 1.48 | 1.49 | 1.38 | 2.48 | 0.18 | 1.20 |
| Dich.0.55Load.Cross250.4Var.300Size.2Fac.0.00Fcor | 0.94 | 0.89 | 1.29 | 2.04 | 0.05 | 0.06 | 0.02 | 0.08 | 0.21 | -0.04 | 0.33 | 0.32 | 0.42 | 0.58 | 0.21 | 0.04 |
| Dich.0.55Load.Cross250.4Var.300Size.2Fac.0.50Fcor | 0.71 | 0.73 | 1.71 | 3.19 | -0.20 | -0.18 | -0.27 | -0.43 | -0.23 | -0.76 | 0.32 | 0.30 | 0.37 | 0.65 | 0.35 | 0.76 |
| Dich.0.55Load.Cross250.4Var.300Size.2Fac.0.70Fcor | 1.10 | 1.07 | 2.95 | 3.77 | -0.27 | -0.27 | -0.49 | -0.58 | -0.72 | -0.97 | 0.49 | 0.49 | 0.67 | 0.88 | 0.72 | 0.97 |
| Dich.0.55Load.Cross250.4Var.500Size.2Fac.0.00Fcor | 0.76 | 0.68 | 0.96 | 2.19 | 0.07 | 0.11 | 0.06 | -0.06 | 0.09 | 0.00 | 0.27 | 0.29 | 0.38 | 0.56 | 0.09 | 0.00 |
| Dich.0.55Load.Cross250.4Var.500Size.2Fac.0.50Fcor | 0.82 | 0.81 | 1.84 | 2.80 | -0.19 | -0.19 | -0.23 | -0.40 | -0.21 | -0.66 | 0.35 | 0.35 | 0.41 | 0.66 | 0.31 | 0.66 |
| Dich.0.55Load.Cross250.4Var.500Size.2Fac.0.70Fcor | 0.84 | 0.85 | 2.61 | 3.62 | -0.29 | -0.29 | -0.45 | -0.60 | -0.83 | -0.99 | 0.41 | 0.41 | 0.59 | 0.80 | 0.85 | 0.99 |
| Dich.0.55Load.Cross250.4Var.1000Size.2Fac.0.00Fcor | 0.41 | 0.48 | 0.74 | 1.47 | 0.04 | 0.06 | 0.07 | 0.10 | 0.10 | 0.00 | 0.14 | 0.16 | 0.23 | 0.38 | 0.10 | 0.00 |
| Dich.0.55Load.Cross250.4Var.1000Size.2Fac.0.50Fcor | 0.34 | 0.34 | 1.00 | 2.08 | -0.02 | -0.02 | -0.09 | -0.26 | -0.25 | -0.57 | 0.22 | 0.22 | 0.23 | 0.52 | 0.25 | 0.57 |
| Dich.0.55Load.Cross250.4Var.1000Size.2Fac.0.70Fcor | 0.74 | 0.74 | 3.34 | 4.08 | -0.35 | -0.35 | -0.70 | -0.86 | -0.92 | -1.00 | 0.45 | 0.45 | 0.82 | 1.00 | 0.92 | 1.00 |
| Dich.0.55Load.Cross250.4Var.300Size.4Fac.0.00Fcor | 2.17 | 2.09 | 3.29 | 2.94 | -0.21 | -0.21 | -0.86 | -0.05 | -0.12 | -0.07 | 0.65 | 0.65 | 1.16 | 0.67 | 0.22 | 0.07 |
| Dich.0.55Load.Cross250.4Var.300Size.4Fac.0.50Fcor | 2.66 | 2.72 | 1.94 | 3.36 | -0.52 | -0.54 | -0.98 | -0.04 | -0.78 | -2.29 | 0.96 | 0.94 | 1.44 | 0.80 | 0.86 | 2.29 |
| Dich.0.55Load.Cross250.4Var.300Size.4Fac.0.70Fcor | 3.59 | 3.72 | 2.71 | 3.81 | -0.76 | -0.79 | -1.17 | -0.30 | -1.19 | -2.98 | 1.18 | 1.21 | 1.37 | 1.02 | 1.25 | 2.98 |
| Dich.0.55Load.Cross250.4Var.500Size.4Fac.0.00Fcor | 0.45 | 0.47 | 1.54 | 1.69 | 0.00 | -0.02 | -1.03 | 0.26 | -0.13 | 0.00 | 0.36 | 0.36 | 1.17 | 0.40 | 0.13 | 0.00 |
| Dich.0.55Load.Cross250.4Var.500Size.4Fac.0.50Fcor | 2.25 | 2.21 | 1.56 | 3.18 | -0.32 | -0.34 | -1.19 | -0.07 | -0.57 | -1.88 | 0.94 | 0.96 | 1.39 | 0.83 | 0.73 | 1.88 |
| Dich.0.55Load.Cross250.4Var.500Size.4Fac.0.70Fcor | 3.74 | 3.71 | 2.95 | 4.18 | -1.06 | -1.08 | -1.46 | -0.68 | -1.18 | -2.97 | 1.26 | 1.28 | 1.50 | 1.02 | 1.24 | 2.97 |
| Dich.0.55Load.Cross250.4Var.1000Size.4Fac.0.00Fcor | 0.12 | 0.12 | 0.63 | 0.86 | 0.00 | 0.00 | -1.16 | 0.35 | -0.08 | 0.00 | 0.08 | 0.10 | 1.20 | 0.35 | 0.08 | 0.00 |
| Dich.0.55Load.Cross250.4Var.1000Size.4Fac.0.50Fcor | 1.30 | 1.31 | 0.97 | 2.28 | -0.44 | -0.44 | -1.22 | -0.07 | -0.55 | -1.16 | 0.64 | 0.62 | 1.38 | 0.63 | 0.59 | 1.16 |
| Dich.0.55Load.Cross250.4Var.1000Size.4Fac.0.70Fcor | 3.39 | 3.39 | 2.54 | 4.16 | -1.30 | -1.30 | -1.60 | -1.04 | -1.42 | -2.93 | 1.36 | 1.36 | 1.62 | 1.16 | 1.42 | 2.93 |
| Dich.0.55Load.Cross250.8Var.300Size.2Fac.0.00Fcor | 0.86 | 0.86 | 1.85 | 2.64 | 0.49 | 0.52 | 0.97 | 2.09 | 0.22 | 0.00 | 0.49 | 0.52 | 0.97 | 2.09 | 0.22 | 0.00 |
| Dich.0.55Load.Cross250.8Var.300Size.2Fac.0.50Fcor | 1.84 | 1.79 | 1.58 | 3.36 | 1.23 | 1.25 | 0.91 | 2.06 | 0.18 | -0.09 | 1.23 | 1.25 | 0.91 | 2.06 | 0.18 | 0.09 |
| Dich.0.55Load.Cross250.8Var.300Size.2Fac.0.70Fcor | 2.79 | 2.95 | 1.78 | 3.49 | 1.39 | 1.36 | 0.92 | 2.05 | 0.40 | -0.76 | 1.45 | 1.42 | 0.92 | 2.05 | 0.46 | 0.76 |
| Dich.0.55Load.Cross250.8Var.500Size.2Fac.0.00Fcor | 0.35 | 0.34 | 1.85 | 2.67 | 0.23 | 0.25 | 0.80 | 1.76 | 0.16 | 0.00 | 0.23 | 0.25 | 0.80 | 1.76 | 0.16 | 0.00 |
| Dich.0.55Load.Cross250.8Var.500Size.2Fac.0.50Fcor | 1.35 | 1.39 | 1.84 | 2.62 | 0.73 | 0.75 | 1.00 | 2.22 | 0.06 | 0.00 | 0.81 | 0.83 | 1.00 | 2.22 | 0.06 | 0.00 |
| Dich.0.55Load.Cross250.8Var.500Size.2Fac.0.70Fcor | 2.76 | 2.82 | 1.44 | 3.72 | 0.88 | 0.89 | 0.73 | 1.57 | 0.15 | -0.50 | 1.02 | 1.03 | 0.73 | 1.59 | 0.15 | 0.50 |
| Dich.0.55Load.Cross250.8Var.1000Size.2Fac.0.00Fcor | 0.06 | 0.16 | 1.87 | 2.91 | 0.02 | 0.03 | 0.55 | 1.35 | 0.21 | 0.00 | 0.02 | 0.03 | 0.55 | 1.37 | 0.21 | 0.00 |
| Dich.0.55Load.Cross250.8Var.1000Size.2Fac.0.50Fcor | 0.33 | 0.30 | 1.65 | 3.18 | 0.13 | 0.14 | 0.77 | 1.27 | 0.03 | 0.00 | 0.15 | 0.16 | 0.81 | 1.31 | 0.03 | 0.00 |
| Dich.0.55Load.Cross250.8Var.1000Size.2Fac.0.70Fcor | 1.14 | 1.13 | 2.55 | 3.12 | 0.29 | 0.29 | 0.62 | 1.11 | 0.05 | -0.17 | 0.35 | 0.35 | 0.62 | 1.11 | 0.05 | 0.17 |
| Dich.0.55Load.Cross250.8Var.300Size.4Fac.0.00Fcor | 2.45 | 2.53 | 4.62 | 5.86 | 1.15 | 1.15 | 0.20 | 2.90 | 0.11 | 0.00 | 1.29 | 1.31 | 1.26 | 2.90 | 0.11 | 0.00 |
| Dich.0.55Load.Cross250.8Var.300Size.4Fac.0.50Fcor | 5.18 | 5.37 | 3.45 | 5.77 | 3.09 | 3.09 | 0.89 | 4.24 | -0.01 | -0.59 | 3.21 | 3.23 | 2.05 | 4.24 | 0.41 | 0.61 |
| Dich.0.55Load.Cross250.8Var.300Size.4Fac.0.70Fcor | 7.82 | 7.56 | 4.80 | 6.21 | 2.87 | 2.92 | 1.28 | 4.10 | 0.17 | -2.81 | 3.01 | 3.04 | 2.24 | 4.12 | 0.79 | 2.81 |
| Dich.0.55Load.Cross250.8Var.500Size.4Fac.0.00Fcor | 0.56 | 0.65 | 1.84 | 4.82 | 0.58 | 0.60 | -0.56 | 2.70 | 0.02 | 0.00 | 0.70 | 0.70 | 1.32 | 2.70 | 0.02 | 0.00 |
| Dich.0.55Load.Cross250.8Var.500Size.4Fac.0.50Fcor | 3.39 | 3.39 | 2.15 | 5.35 | 2.10 | 2.10 | 0.00 | 3.86 | 0.16 | -0.15 | 2.36 | 2.36 | 1.50 | 3.90 | 0.36 | 0.15 |
| Dich.0.55Load.Cross250.8Var.500Size.4Fac.0.70Fcor | 6.82 | 6.76 | 5.53 | 6.46 | 2.66 | 2.71 | 1.06 | 3.35 | -0.15 | -2.53 | 2.72 | 2.77 | 1.94 | 3.35 | 0.61 | 2.53 |
| Dich.0.55Load.Cross250.8Var.1000Size.4Fac.0.00Fcor | 0.11 | 0.13 | 1.39 | 4.33 | 0.10 | 0.11 | -1.03 | 2.08 | 0.08 | 0.00 | 0.10 | 0.11 | 1.37 | 2.08 | 0.10 | 0.00 |
| Dich.0.55Load.Cross250.8Var.1000Size.4Fac.0.50Fcor | 1.31 | 1.31 | 1.34 | 4.54 | 0.68 | 0.68 | -0.52 | 3.12 | 0.14 | 0.00 | 0.86 | 0.86 | 1.56 | 3.12 | 0.22 | 0.00 |
| Dich.0.55Load.Cross250.8Var.1000Size.4Fac.0.70Fcor | 4.93 | 5.05 | 3.81 | 5.89 | 1.26 | 1.23 | -0.01 | 2.46 | 0.04 | -1.72 | 1.68 | 1.69 | 1.41 | 2.56 | 0.40 | 1.72 |
| Dich.0.70Load.000Cross.4Var.300Size.2Fac.0.00Fcor | 0.05 | 0.05 | 0.47 | 1.15 | -0.01 | -0.01 | 0.21 | 0.22 | 0.00 | 0.00 | 0.01 | 0.01 | 0.21 | 0.40 | 0.00 | 0.00 |
| Dich.0.70Load.000Cross.4Var.300Size.2Fac.0.50Fcor | 0.04 | 0.04 | 0.27 | 1.02 | 0.04 | 0.04 | 0.24 | 0.34 | 0.00 | -0.07 | 0.04 | 0.04 | 0.24 | 0.44 | 0.00 | 0.07 |
| Dich.0.70Load.000Cross.4Var.300Size.2Fac.0.70Fcor | 0.31 | 0.30 | 0.41 | 1.23 | 0.13 | 0.13 | 0.19 | 0.20 | -0.25 | -0.82 | 0.15 | 0.15 | 0.23 | 0.40 | 0.27 | 0.82 |
| Dich.0.70Load.000Cross.4Var.500Size.2Fac.0.00Fcor | 0.00 | 0.00 | 0.30 | 0.73 | 0.00 | 0.00 | 0.16 | 0.19 | 0.00 | 0.00 | 0.00 | 0.00 | 0.16 | 0.31 | 0.00 | 0.00 |
| Dich.0.70Load.000Cross.4Var.500Size.2Fac.0.50Fcor | 0.00 | 0.00 | 0.26 | 0.73 | 0.00 | 0.00 | 0.14 | 0.17 | 0.00 | -0.02 | 0.00 | 0.00 | 0.14 | 0.27 | 0.00 | 0.02 |
| Dich.0.70Load.000Cross.4Var.500Size.2Fac.0.70Fcor | 0.10 | 0.10 | 0.33 | 0.95 | 0.04 | 0.04 | 0.10 | 0.20 | -0.21 | -0.71 | 0.06 | 0.06 | 0.10 | 0.34 | 0.21 | 0.71 |
| Dich.0.70Load.000Cross.4Var.1000Size.2Fac.0.00Fcor | 0.00 | 0.00 | 0.25 | 0.60 | 0.00 | 0.00 | 0.12 | 0.10 | 0.00 | 0.00 | 0.00 | 0.00 | 0.12 | 0.22 | 0.00 | 0.00 |
| Dich.0.70Load.000Cross.4Var.1000Size.2Fac.0.50Fcor | 0.00 | 0.00 | 0.13 | 0.66 | 0.00 | 0.00 | 0.10 | 0.07 | 0.00 | 0.00 | 0.00 | 0.00 | 0.10 | 0.25 | 0.00 | 0.00 |
| Dich.0.70Load.000Cross.4Var.1000Size.2Fac.0.70Fcor | 0.01 | 0.01 | 0.32 | 0.79 | 0.00 | 0.00 | 0.07 | 0.07 | -0.12 | -0.58 | 0.00 | 0.00 | 0.07 | 0.21 | 0.12 | 0.58 |
| Dich.0.70Load.000Cross.4Var.300Size.4Fac.0.00Fcor | 0.00 | 0.00 | 1.44 | 1.57 | 0.04 | 0.04 | -0.62 | 0.51 | 0.00 | 0.00 | 0.04 | 0.04 | 0.80 | 0.53 | 0.00 | 0.00 |
| Dich.0.70Load.000Cross.4Var.300Size.4Fac.0.50Fcor | 0.37 | 0.33 | 0.48 | 1.23 | 0.14 | 0.15 | -1.08 | 0.56 | -0.01 | -0.64 | 0.24 | 0.23 | 1.26 | 0.70 | 0.01 | 0.64 |
| Dich.0.70Load.000Cross.4Var.300Size.4Fac.0.70Fcor | 1.56 | 1.50 | 1.38 | 2.08 | 0.07 | 0.06 | -1.20 | 0.53 | -0.37 | -2.87 | 0.69 | 0.70 | 1.46 | 0.75 | 0.43 | 2.87 |
| Dich.0.70Load.000Cross.4Var.500Size.4Fac.0.00Fcor | 0.00 | 0.00 | 0.75 | 0.91 | 0.00 | 0.00 | -0.67 | 0.39 | 0.00 | 0.00 | 0.00 | 0.00 | 0.81 | 0.43 | 0.00 | 0.00 |
| Dich.0.70Load.000Cross.4Var.500Size.4Fac.0.50Fcor | 0.06 | 0.06 | 0.67 | 0.93 | 0.07 | 0.07 | -1.09 | 0.45 | 0.00 | -0.12 | 0.09 | 0.09 | 1.21 | 0.49 | 0.00 | 0.12 |
| Dich.0.70Load.000Cross.4Var.500Size.4Fac.0.70Fcor | 0.77 | 0.77 | 1.02 | 1.99 | 0.03 | 0.03 | -1.14 | 0.26 | -0.08 | -2.78 | 0.35 | 0.35 | 1.24 | 0.62 | 0.08 | 2.78 |
| Dich.0.70Load.000Cross.4Var.1000Size.4Fac.0.00Fcor | 0.00 | 0.00 | 0.56 | 0.72 | 0.00 | 0.00 | -0.69 | 0.33 | 0.00 | 0.00 | 0.00 | 0.00 | 0.81 | 0.35 | 0.00 | 0.00 |
| Dich.0.70Load.000Cross.4Var.1000Size.4Fac.0.50Fcor | 0.00 | 0.00 | 1.08 | 0.80 | 0.00 | 0.00 | -1.20 | 0.25 | 0.00 | -0.01 | 0.00 | 0.00 | 1.22 | 0.33 | 0.00 | 0.01 |
| Dich.0.70Load.000Cross.4Var.1000Size.4Fac.0.70Fcor | 0.23 | 0.23 | 1.01 | 1.43 | 0.04 | 0.04 | -1.28 | 0.41 | -0.02 | -2.47 | 0.12 | 0.12 | 1.38 | 0.53 | 0.02 | 2.47 |
| Dich.0.70Load.000Cross.8Var.300Size.2Fac.0.00Fcor | 0.00 | 0.01 | 2.47 | 2.49 | 0.08 | 0.10 | 1.10 | 2.20 | 0.00 | 0.00 | 0.08 | 0.10 | 1.10 | 2.20 | 0.00 | 0.00 |
| Dich.0.70Load.000Cross.8Var.300Size.2Fac.0.50Fcor | 0.17 | 0.18 | 1.87 | 2.55 | 0.29 | 0.29 | 0.95 | 2.12 | 0.00 | 0.00 | 0.29 | 0.29 | 0.95 | 2.12 | 0.00 | 0.00 |
| Dich.0.70Load.000Cross.8Var.300Size.2Fac.0.70Fcor | 0.69 | 0.66 | 1.76 | 2.49 | 0.66 | 0.69 | 0.87 | 2.35 | 0.02 | -0.11 | 0.66 | 0.69 | 0.87 | 2.35 | 0.02 | 0.11 |
| Dich.0.70Load.000Cross.8Var.500Size.2Fac.0.00Fcor | 0.00 | 0.00 | 1.57 | 2.59 | 0.00 | 0.01 | 1.31 | 1.96 | 0.00 | 0.00 | 0.00 | 0.01 | 1.31 | 1.96 | 0.00 | 0.00 |
| Dich.0.70Load.000Cross.8Var.500Size.2Fac.0.50Fcor | 0.03 | 0.03 | 1.93 | 2.88 | 0.01 | 0.01 | 0.90 | 1.90 | 0.00 | 0.00 | 0.01 | 0.01 | 0.90 | 1.90 | 0.00 | 0.00 |
| Dich.0.70Load.000Cross.8Var.500Size.2Fac.0.70Fcor | 0.17 | 0.17 | 2.15 | 2.53 | 0.14 | 0.14 | 0.74 | 1.79 | 0.00 | 0.00 | 0.14 | 0.14 | 0.74 | 1.79 | 0.00 | 0.00 |
| Dich.0.70Load.000Cross.8Var.1000Size.2Fac.0.00Fcor | 0.00 | 0.00 | 1.48 | 2.29 | 0.00 | 0.00 | 0.84 | 1.41 | 0.00 | 0.00 | 0.00 | 0.00 | 0.84 | 1.41 | 0.00 | 0.00 |
| Dich.0.70Load.000Cross.8Var.1000Size.2Fac.0.50Fcor | 0.00 | 0.00 | 2.01 | 2.63 | 0.00 | 0.01 | 0.70 | 1.22 | 0.00 | 0.00 | 0.00 | 0.01 | 0.70 | 1.22 | 0.00 | 0.00 |
| Dich.0.70Load.000Cross.8Var.1000Size.2Fac.0.70Fcor | 0.01 | 0.01 | 1.67 | 2.85 | 0.02 | 0.02 | 0.60 | 1.22 | 0.00 | 0.00 | 0.02 | 0.02 | 0.60 | 1.22 | 0.00 | 0.00 |
| Dich.0.70Load.000Cross.8Var.300Size.4Fac.0.00Fcor | 0.01 | 0.01 | 6.17 | 4.37 | 0.03 | 0.03 | 0.20 | 3.59 | 0.00 | 0.00 | 0.03 | 0.03 | 1.72 | 3.59 | 0.00 | 0.00 |
| Dich.0.70Load.000Cross.8Var.300Size.4Fac.0.50Fcor | 0.37 | 0.39 | 2.36 | 4.28 | 0.66 | 0.65 | -0.39 | 3.80 | 0.00 | -0.01 | 0.72 | 0.71 | 1.51 | 3.80 | 0.00 | 0.01 |
| Dich.0.70Load.000Cross.8Var.300Size.4Fac.0.70Fcor | 2.13 | 2.11 | 2.38 | 3.50 | 1.99 | 2.06 | -0.32 | 4.17 | 0.00 | -1.05 | 2.11 | 2.14 | 1.30 | 4.17 | 0.04 | 1.05 |
| Dich.0.70Load.000Cross.8Var.500Size.4Fac.0.00Fcor | 0.00 | 0.00 | 5.66 | 3.84 | 0.01 | 0.01 | 0.47 | 3.47 | 0.00 | 0.00 | 0.01 | 0.01 | 1.87 | 3.47 | 0.00 | 0.00 |
| Dich.0.70Load.000Cross.8Var.500Size.4Fac.0.50Fcor | 0.07 | 0.08 | 2.16 | 3.11 | 0.14 | 0.16 | -0.67 | 3.29 | 0.00 | 0.00 | 0.16 | 0.16 | 1.35 | 3.29 | 0.00 | 0.00 |
| Dich.0.70Load.000Cross.8Var.500Size.4Fac.0.70Fcor | 0.63 | 0.63 | 1.95 | 4.26 | 0.85 | 0.85 | -0.63 | 3.30 | 0.00 | -0.29 | 0.91 | 0.91 | 1.33 | 3.30 | 0.00 | 0.29 |
| Dich.0.70Load.000Cross.8Var.1000Size.4Fac.0.00Fcor | 0.00 | 0.00 | 2.29 | 3.61 | 0.00 | 0.00 | 0.28 | 2.90 | 0.00 | 0.00 | 0.00 | 0.00 | 1.52 | 2.90 | 0.00 | 0.00 |
| Dich.0.70Load.000Cross.8Var.1000Size.4Fac.0.50Fcor | 0.00 | 0.00 | 1.66 | 4.41 | 0.00 | 0.00 | -0.76 | 2.45 | 0.00 | 0.00 | 0.00 | 0.00 | 1.38 | 2.45 | 0.00 | 0.00 |
| Dich.0.70Load.000Cross.8Var.1000Size.4Fac.0.70Fcor | 0.17 | 0.17 | 2.20 | 4.37 | 0.05 | 0.05 | -0.96 | 2.25 | 0.00 | 0.00 | 0.05 | 0.05 | 1.42 | 2.25 | 0.00 | 0.00 |
| Dich.0.70Load.125Cross.4Var.300Size.2Fac.0.00Fcor | 0.13 | 0.15 | 0.35 | 1.20 | 0.03 | 0.04 | 0.15 | 0.20 | 0.03 | 0.00 | 0.07 | 0.08 | 0.19 | 0.32 | 0.03 | 0.00 |
| Dich.0.70Load.125Cross.4Var.300Size.2Fac.0.50Fcor | 0.18 | 0.21 | 0.43 | 1.55 | 0.07 | 0.06 | 0.10 | 0.18 | -0.01 | -0.20 | 0.11 | 0.10 | 0.10 | 0.38 | 0.01 | 0.20 |
| Dich.0.70Load.125Cross.4Var.300Size.2Fac.0.70Fcor | 0.50 | 0.51 | 0.96 | 1.91 | -0.02 | -0.01 | 0.07 | 0.19 | -0.60 | -0.91 | 0.14 | 0.15 | 0.19 | 0.57 | 0.62 | 0.91 |
| Dich.0.70Load.125Cross.4Var.500Size.2Fac.0.00Fcor | 0.05 | 0.06 | 0.35 | 1.34 | 0.02 | 0.02 | 0.06 | 0.09 | 0.00 | 0.00 | 0.02 | 0.02 | 0.06 | 0.31 | 0.00 | 0.00 |
| Dich.0.70Load.125Cross.4Var.500Size.2Fac.0.50Fcor | 0.04 | 0.04 | 0.31 | 1.19 | 0.03 | 0.03 | 0.23 | 0.27 | 0.00 | -0.08 | 0.03 | 0.03 | 0.23 | 0.45 | 0.00 | 0.08 |
| Dich.0.70Load.125Cross.4Var.500Size.2Fac.0.70Fcor | 0.25 | 0.26 | 0.58 | 1.90 | 0.02 | 0.02 | 0.08 | 0.07 | -0.66 | -0.94 | 0.06 | 0.06 | 0.12 | 0.35 | 0.68 | 0.94 |
| Dich.0.70Load.125Cross.4Var.1000Size.2Fac.0.00Fcor | 0.00 | 0.00 | 0.28 | 1.05 | 0.00 | 0.00 | 0.01 | 0.07 | 0.00 | 0.00 | 0.00 | 0.00 | 0.01 | 0.11 | 0.00 | 0.00 |
| Dich.0.70Load.125Cross.4Var.1000Size.2Fac.0.50Fcor | 0.00 | 0.00 | 0.32 | 1.39 | 0.00 | 0.00 | 0.04 | 0.03 | 0.00 | -0.01 | 0.00 | 0.00 | 0.04 | 0.31 | 0.00 | 0.01 |
| Dich.0.70Load.125Cross.4Var.1000Size.2Fac.0.70Fcor | 0.07 | 0.07 | 0.58 | 1.73 | 0.02 | 0.02 | 0.00 | -0.01 | -0.83 | -0.94 | 0.04 | 0.04 | 0.04 | 0.29 | 0.83 | 0.94 |
| Dich.0.70Load.125Cross.4Var.300Size.4Fac.0.00Fcor | 0.13 | 0.11 | 0.60 | 1.54 | -0.03 | -0.06 | -1.25 | 0.33 | -0.05 | 0.00 | 0.11 | 0.12 | 1.25 | 0.47 | 0.05 | 0.00 |
| Dich.0.70Load.125Cross.4Var.300Size.4Fac.0.50Fcor | 0.64 | 0.67 | 0.66 | 1.91 | -0.12 | -0.13 | -1.24 | 0.34 | -0.19 | -0.98 | 0.38 | 0.39 | 1.32 | 0.56 | 0.25 | 0.98 |
| Dich.0.70Load.125Cross.4Var.300Size.4Fac.0.70Fcor | 1.85 | 1.83 | 1.07 | 2.41 | -0.26 | -0.25 | -1.06 | 0.34 | -0.77 | -2.88 | 0.68 | 0.67 | 1.30 | 0.76 | 0.77 | 2.88 |
| Dich.0.70Load.125Cross.4Var.500Size.4Fac.0.00Fcor | 0.04 | 0.03 | 0.22 | 1.53 | -0.02 | -0.04 | -1.39 | 0.39 | -0.03 | 0.00 | 0.04 | 0.06 | 1.41 | 0.49 | 0.03 | 0.00 |
| Dich.0.70Load.125Cross.4Var.500Size.4Fac.0.50Fcor | 0.15 | 0.14 | 0.49 | 1.03 | 0.07 | 0.07 | -1.39 | 0.59 | -0.15 | -0.54 | 0.21 | 0.21 | 1.43 | 0.63 | 0.15 | 0.54 |
| Dich.0.70Load.125Cross.4Var.500Size.4Fac.0.70Fcor | 1.36 | 1.39 | 1.12 | 2.45 | -0.36 | -0.37 | -1.20 | 0.07 | -0.44 | -2.89 | 0.62 | 0.63 | 1.28 | 0.53 | 0.46 | 2.89 |
| Dich.0.70Load.125Cross.4Var.1000Size.4Fac.0.00Fcor | 0.00 | 0.00 | 0.12 | 1.47 | 0.00 | -0.01 | -1.55 | 0.27 | -0.03 | 0.00 | 0.00 | 0.01 | 1.55 | 0.27 | 0.03 | 0.00 |
| Dich.0.70Load.125Cross.4Var.1000Size.4Fac.0.50Fcor | 0.01 | 0.01 | 0.49 | 1.22 | -0.02 | -0.02 | -1.44 | 0.44 | -0.08 | -0.15 | 0.02 | 0.02 | 1.46 | 0.50 | 0.08 | 0.15 |
| Dich.0.70Load.125Cross.4Var.1000Size.4Fac.0.70Fcor | 0.58 | 0.58 | 0.57 | 1.67 | -0.12 | -0.12 | -1.33 | 0.09 | -0.43 | -2.53 | 0.36 | 0.36 | 1.37 | 0.39 | 0.43 | 2.53 |
| Dich.0.70Load.125Cross.8Var.300Size.2Fac.0.00Fcor | 0.49 | 0.60 | 1.53 | 2.41 | 0.50 | 0.54 | 0.93 | 2.30 | 0.23 | 0.00 | 0.52 | 0.56 | 0.93 | 2.30 | 0.23 | 0.00 |
| Dich.0.70Load.125Cross.8Var.300Size.2Fac.0.50Fcor | 0.71 | 0.70 | 1.37 | 2.67 | 0.61 | 0.67 | 1.11 | 2.41 | 0.23 | 0.00 | 0.65 | 0.71 | 1.11 | 2.41 | 0.23 | 0.00 |
| Dich.0.70Load.125Cross.8Var.300Size.2Fac.0.70Fcor | 1.45 | 1.46 | 2.22 | 2.78 | 0.75 | 0.79 | 0.90 | 1.98 | 0.26 | -0.29 | 0.81 | 0.85 | 0.90 | 1.98 | 0.26 | 0.29 |
| Dich.0.70Load.125Cross.8Var.500Size.2Fac.0.00Fcor | 0.19 | 0.25 | 2.21 | 2.72 | 0.26 | 0.27 | 0.82 | 1.85 | 0.29 | 0.00 | 0.26 | 0.27 | 0.82 | 1.85 | 0.29 | 0.00 |
| Dich.0.70Load.125Cross.8Var.500Size.2Fac.0.50Fcor | 0.14 | 0.14 | 1.57 | 2.64 | 0.11 | 0.11 | 0.98 | 2.06 | 0.26 | 0.00 | 0.11 | 0.11 | 0.98 | 2.06 | 0.26 | 0.00 |
| Dich.0.70Load.125Cross.8Var.500Size.2Fac.0.70Fcor | 0.77 | 0.77 | 2.05 | 2.98 | 0.29 | 0.29 | 0.89 | 1.65 | 0.12 | -0.06 | 0.31 | 0.31 | 0.89 | 1.67 | 0.12 | 0.06 |
| Dich.0.70Load.125Cross.8Var.1000Size.2Fac.0.00Fcor | 0.11 | 0.14 | 1.65 | 2.91 | 0.03 | 0.04 | 0.54 | 1.29 | 0.49 | 0.00 | 0.03 | 0.04 | 0.54 | 1.29 | 0.49 | 0.00 |
| Dich.0.70Load.125Cross.8Var.1000Size.2Fac.0.50Fcor | 0.02 | 0.02 | 1.88 | 2.34 | 0.00 | 0.00 | 0.97 | 1.60 | 0.32 | 0.00 | 0.00 | 0.00 | 0.97 | 1.60 | 0.32 | 0.00 |
| Dich.0.70Load.125Cross.8Var.1000Size.2Fac.0.70Fcor | 0.13 | 0.13 | 2.59 | 2.63 | 0.00 | 0.00 | 0.54 | 0.88 | 0.06 | 0.00 | 0.02 | 0.02 | 0.54 | 0.88 | 0.06 | 0.00 |
| Dich.0.70Load.125Cross.8Var.300Size.4Fac.0.00Fcor | 0.18 | 0.18 | 2.78 | 3.91 | 0.20 | 0.23 | -0.52 | 3.50 | 0.00 | 0.00 | 0.20 | 0.23 | 1.54 | 3.50 | 0.00 | 0.00 |
| Dich.0.70Load.125Cross.8Var.300Size.4Fac.0.50Fcor | 0.85 | 0.89 | 1.73 | 4.11 | 0.74 | 0.73 | -0.47 | 4.01 | 0.03 | 0.00 | 1.02 | 1.03 | 1.53 | 4.01 | 0.03 | 0.00 |
| Dich.0.70Load.125Cross.8Var.300Size.4Fac.0.70Fcor | 2.95 | 2.98 | 1.87 | 5.03 | 2.12 | 2.07 | 0.53 | 3.94 | 0.03 | -1.33 | 2.32 | 2.27 | 1.89 | 3.94 | 0.11 | 1.33 |
| Dich.0.70Load.125Cross.8Var.500Size.4Fac.0.00Fcor | 0.02 | 0.03 | 2.20 | 5.06 | 0.03 | 0.03 | -0.77 | 3.05 | 0.01 | 0.00 | 0.03 | 0.03 | 1.47 | 3.05 | 0.01 | 0.00 |
| Dich.0.70Load.125Cross.8Var.500Size.4Fac.0.50Fcor | 0.25 | 0.25 | 2.42 | 3.87 | 0.10 | 0.12 | -0.58 | 3.90 | 0.01 | 0.00 | 0.18 | 0.20 | 1.74 | 3.90 | 0.01 | 0.00 |
| Dich.0.70Load.125Cross.8Var.500Size.4Fac.0.70Fcor | 1.53 | 1.53 | 2.30 | 5.07 | 0.92 | 0.92 | -0.41 | 3.60 | 0.02 | -0.55 | 1.06 | 1.06 | 1.59 | 3.60 | 0.02 | 0.55 |
| Dich.0.70Load.125Cross.8Var.1000Size.4Fac.0.00Fcor | 0.00 | 0.00 | 2.59 | 4.27 | 0.00 | 0.00 | -1.08 | 2.28 | 0.01 | 0.00 | 0.00 | 0.00 | 1.30 | 2.28 | 0.01 | 0.00 |
| Dich.0.70Load.125Cross.8Var.1000Size.4Fac.0.50Fcor | 0.02 | 0.02 | 2.41 | 3.89 | 0.00 | 0.00 | -0.99 | 2.94 | 0.00 | 0.00 | 0.00 | 0.00 | 1.45 | 2.94 | 0.00 | 0.00 |
| Dich.0.70Load.125Cross.8Var.1000Size.4Fac.0.70Fcor | 0.21 | 0.22 | 2.57 | 5.41 | 0.08 | 0.08 | -0.93 | 2.62 | 0.02 | -0.02 | 0.08 | 0.08 | 1.45 | 2.62 | 0.02 | 0.02 |
| Dich.0.70Load.Cross250.4Var.300Size.2Fac.0.00Fcor | 0.52 | 0.51 | 0.36 | 1.38 | 0.16 | 0.20 | 0.16 | 0.31 | 0.25 | 0.00 | 0.20 | 0.24 | 0.20 | 0.51 | 0.25 | 0.00 |
| Dich.0.70Load.Cross250.4Var.300Size.2Fac.0.50Fcor | 0.70 | 0.67 | 0.75 | 1.85 | -0.02 | -0.02 | 0.09 | 0.03 | -0.22 | -0.57 | 0.30 | 0.30 | 0.29 | 0.59 | 0.32 | 0.57 |
| Dich.0.70Load.Cross250.4Var.300Size.2Fac.0.70Fcor | 0.74 | 0.74 | 2.05 | 2.99 | -0.18 | -0.18 | -0.29 | -0.44 | -0.92 | -0.99 | 0.32 | 0.32 | 0.51 | 0.70 | 0.94 | 0.99 |
| Dich.0.70Load.Cross250.4Var.500Size.2Fac.0.00Fcor | 0.45 | 0.56 | 0.61 | 1.57 | 0.16 | 0.16 | 0.19 | 0.23 | 0.19 | 0.00 | 0.20 | 0.20 | 0.27 | 0.35 | 0.19 | 0.00 |
| Dich.0.70Load.Cross250.4Var.500Size.2Fac.0.50Fcor | 0.22 | 0.20 | 0.44 | 1.45 | 0.03 | 0.04 | 0.21 | 0.17 | -0.11 | -0.29 | 0.15 | 0.14 | 0.25 | 0.51 | 0.21 | 0.29 |
| Dich.0.70Load.Cross250.4Var.500Size.2Fac.0.70Fcor | 0.47 | 0.47 | 1.32 | 2.47 | -0.13 | -0.13 | -0.14 | -0.35 | -0.97 | -0.97 | 0.21 | 0.21 | 0.34 | 0.59 | 0.97 | 0.97 |
| Dich.0.70Load.Cross250.4Var.1000Size.2Fac.0.00Fcor | 0.45 | 0.53 | 0.53 | 1.68 | 0.01 | 0.03 | 0.16 | 0.13 | 0.22 | 0.00 | 0.01 | 0.03 | 0.16 | 0.15 | 0.22 | 0.00 |
| Dich.0.70Load.Cross250.4Var.1000Size.2Fac.0.50Fcor | 0.03 | 0.03 | 0.40 | 1.40 | 0.01 | 0.01 | 0.16 | 0.07 | -0.19 | -0.14 | 0.01 | 0.01 | 0.16 | 0.39 | 0.21 | 0.14 |
| Dich.0.70Load.Cross250.4Var.1000Size.2Fac.0.70Fcor | 0.11 | 0.11 | 0.44 | 1.60 | -0.02 | -0.02 | 0.02 | -0.22 | -0.98 | -0.99 | 0.04 | 0.04 | 0.06 | 0.32 | 0.98 | 0.99 |
| Dich.0.70Load.Cross250.4Var.300Size.4Fac.0.00Fcor | 0.30 | 0.23 | 0.54 | 1.73 | -0.01 | -0.05 | -1.21 | 0.40 | -0.09 | 0.00 | 0.33 | 0.37 | 1.25 | 0.46 | 0.17 | 0.00 |
| Dich.0.70Load.Cross250.4Var.300Size.4Fac.0.50Fcor | 0.90 | 0.97 | 0.73 | 1.88 | -0.01 | 0.01 | -1.17 | 0.67 | -0.39 | -1.34 | 0.67 | 0.65 | 1.37 | 0.83 | 0.53 | 1.34 |
| Dich.0.70Load.Cross250.4Var.300Size.4Fac.0.70Fcor | 2.15 | 2.02 | 1.32 | 3.02 | -0.60 | -0.57 | -1.11 | 0.16 | -1.06 | -2.93 | 0.78 | 0.75 | 1.27 | 0.62 | 1.18 | 2.93 |
| Dich.0.70Load.Cross250.4Var.500Size.4Fac.0.00Fcor | 0.15 | 0.13 | 0.39 | 2.20 | -0.05 | -0.05 | -1.27 | 0.31 | -0.06 | 0.00 | 0.13 | 0.15 | 1.27 | 0.37 | 0.06 | 0.00 |
| Dich.0.70Load.Cross250.4Var.500Size.4Fac.0.50Fcor | 0.23 | 0.24 | 0.35 | 1.58 | 0.05 | 0.05 | -1.13 | 0.67 | -0.22 | -0.69 | 0.35 | 0.35 | 1.21 | 0.71 | 0.24 | 0.69 |
| Dich.0.70Load.Cross250.4Var.500Size.4Fac.0.70Fcor | 1.41 | 1.47 | 1.26 | 2.00 | -0.36 | -0.39 | -1.10 | 0.30 | -1.11 | -2.90 | 0.78 | 0.79 | 1.40 | 0.78 | 1.17 | 2.90 |
| Dich.0.70Load.Cross250.4Var.1000Size.4Fac.0.00Fcor | 0.01 | 0.01 | 0.26 | 2.32 | -0.01 | -0.01 | -1.18 | 0.11 | -0.04 | 0.00 | 0.01 | 0.01 | 1.20 | 0.11 | 0.04 | 0.00 |
| Dich.0.70Load.Cross250.4Var.1000Size.4Fac.0.50Fcor | 0.00 | 0.00 | 0.39 | 1.40 | -0.01 | -0.01 | -1.14 | 0.47 | -0.11 | -0.31 | 0.01 | 0.01 | 1.16 | 0.51 | 0.13 | 0.31 |
| Dich.0.70Load.Cross250.4Var.1000Size.4Fac.0.70Fcor | 0.54 | 0.54 | 1.55 | 1.81 | -0.22 | -0.22 | -1.37 | 0.33 | -1.01 | -2.79 | 0.28 | 0.28 | 1.41 | 0.41 | 1.07 | 2.79 |
| Dich.0.70Load.Cross250.8Var.300Size.2Fac.0.00Fcor | 0.49 | 0.60 | 1.53 | 2.41 | 0.50 | 0.54 | 0.93 | 2.30 | 0.23 | 0.00 | 0.52 | 0.56 | 0.93 | 2.30 | 0.23 | 0.00 |
| Dich.0.70Load.Cross250.8Var.300Size.2Fac.0.50Fcor | 0.71 | 0.70 | 1.37 | 2.67 | 0.61 | 0.67 | 1.11 | 2.41 | 0.23 | 0.00 | 0.65 | 0.71 | 1.11 | 2.41 | 0.23 | 0.00 |
| Dich.0.70Load.Cross250.8Var.300Size.2Fac.0.70Fcor | 1.45 | 1.46 | 2.22 | 2.78 | 0.75 | 0.79 | 0.90 | 1.98 | 0.26 | -0.29 | 0.81 | 0.85 | 0.90 | 1.98 | 0.26 | 0.29 |
| Dich.0.70Load.Cross250.8Var.500Size.2Fac.0.00Fcor | 0.19 | 0.25 | 2.21 | 2.72 | 0.26 | 0.27 | 0.82 | 1.85 | 0.29 | 0.00 | 0.26 | 0.27 | 0.82 | 1.85 | 0.29 | 0.00 |
| Dich.0.70Load.Cross250.8Var.500Size.2Fac.0.50Fcor | 0.14 | 0.14 | 1.57 | 2.64 | 0.11 | 0.11 | 0.98 | 2.06 | 0.26 | 0.00 | 0.11 | 0.11 | 0.98 | 2.06 | 0.26 | 0.00 |
| Dich.0.70Load.Cross250.8Var.500Size.2Fac.0.70Fcor | 0.77 | 0.77 | 2.05 | 2.98 | 0.29 | 0.29 | 0.89 | 1.65 | 0.12 | -0.06 | 0.31 | 0.31 | 0.89 | 1.67 | 0.12 | 0.06 |
| Dich.0.70Load.Cross250.8Var.1000Size.2Fac.0.00Fcor | 0.11 | 0.14 | 1.65 | 2.91 | 0.03 | 0.04 | 0.54 | 1.29 | 0.49 | 0.00 | 0.03 | 0.04 | 0.54 | 1.29 | 0.49 | 0.00 |
| Dich.0.70Load.Cross250.8Var.1000Size.2Fac.0.50Fcor | 0.02 | 0.02 | 1.88 | 2.34 | 0.00 | 0.00 | 0.97 | 1.60 | 0.32 | 0.00 | 0.00 | 0.00 | 0.97 | 1.60 | 0.32 | 0.00 |
| Dich.0.70Load.Cross250.8Var.1000Size.2Fac.0.70Fcor | 0.13 | 0.13 | 2.59 | 2.63 | 0.00 | 0.00 | 0.54 | 0.88 | 0.06 | 0.00 | 0.02 | 0.02 | 0.54 | 0.88 | 0.06 | 0.00 |
| Dich.0.70Load.Cross250.8Var.300Size.4Fac.0.00Fcor | 0.82 | 0.77 | 2.13 | 4.04 | 0.73 | 0.91 | -0.72 | 3.61 | 0.10 | 0.00 | 0.75 | 0.93 | 1.34 | 3.61 | 0.10 | 0.00 |
| Dich.0.70Load.Cross250.8Var.300Size.4Fac.0.50Fcor | 1.67 | 1.73 | 1.86 | 3.82 | 1.80 | 1.81 | 0.12 | 4.60 | 0.12 | -0.01 | 1.92 | 1.93 | 1.72 | 4.60 | 0.16 | 0.01 |
| Dich.0.70Load.Cross250.8Var.300Size.4Fac.0.70Fcor | 3.93 | 4.24 | 2.07 | 5.63 | 2.41 | 2.42 | 0.31 | 4.35 | 0.01 | -1.68 | 2.57 | 2.58 | 1.55 | 4.35 | 0.19 | 1.68 |
| Dich.0.70Load.Cross250.8Var.500Size.4Fac.0.00Fcor | 0.35 | 0.37 | 0.63 | 4.13 | 0.51 | 0.55 | -1.16 | 2.92 | 0.22 | 0.00 | 0.55 | 0.59 | 1.40 | 2.92 | 0.22 | 0.00 |
| Dich.0.70Load.Cross250.8Var.500Size.4Fac.0.50Fcor | 0.92 | 0.98 | 2.60 | 3.24 | 0.58 | 0.56 | -0.28 | 4.44 | 0.09 | 0.00 | 0.76 | 0.74 | 1.64 | 4.44 | 0.11 | 0.00 |
| Dich.0.70Load.Cross250.8Var.500Size.4Fac.0.70Fcor | 2.22 | 2.27 | 2.38 | 4.95 | 1.09 | 1.08 | 0.04 | 3.82 | 0.06 | -0.74 | 1.23 | 1.22 | 1.44 | 3.82 | 0.14 | 0.74 |
| Dich.0.70Load.Cross250.8Var.1000Size.4Fac.0.00Fcor | 0.06 | 0.08 | 1.06 | 5.01 | 0.08 | 0.08 | -1.23 | 2.34 | 0.17 | 0.00 | 0.08 | 0.08 | 1.37 | 2.34 | 0.17 | 0.00 |
| Dich.0.70Load.Cross250.8Var.1000Size.4Fac.0.50Fcor | 0.10 | 0.10 | 1.33 | 3.52 | 0.01 | 0.01 | -0.93 | 3.50 | 0.23 | 0.00 | 0.03 | 0.03 | 1.49 | 3.50 | 0.23 | 0.00 |
| Dich.0.70Load.Cross250.8Var.1000Size.4Fac.0.70Fcor | 0.60 | 0.60 | 1.92 | 4.58 | 0.12 | 0.12 | -0.86 | 3.07 | 0.11 | -0.02 | 0.24 | 0.24 | 1.62 | 3.07 | 0.15 | 0.02 |
| Note. Cont= Continuous; Ord= Ordinal; Dich= Dichotomous; Load= Size of primary factor loadings; Cross= Proportion of observed variables with substantive cross-loadings; Var = Number of variables per factor; Size = Sample size; Fac = Number of factors; Fcor = Factor correlation; CPSigMod = CP algorithm with maximisation of the signed fuzzy modularity for signed weighted networks; CPMod = CP algorithm with maximisation of the fuzzy modularity for signed weighted networks; CPRat = CP algorithm with minimisation of the ratio between the two largest communities when the ratio is above or equal 2; CPEnt = CP algorithm with maximisation of entropy; Walk-Ov = Walktrap algorithm with overlapping nodes identified through network loadings >= \|.15\|; EFA-Ov = Exploratory Factor Analysis with overlapping nodes identified through factor loadings >= \|.40\|. | | | | | | | | | | | | | | | | |

Table 3: ANOVA’s Partial eta squared effect sizes.

| Main effects and interactions | Partial eta squared |
| --- | --- |
| Primary Loadings | 0.25909 |
| Factor Correlation | 0.14505 |
| Proportion of substantive cross-loadings | 0.06902 |
| Sample Size | 0.05567 |
| Primary Loadings x Factor Correlation | 0.02291 |
| Number of Variables | 0.01772 |
| Primary Loadings x Sample Size | 0.01771 |
| Factor Correlation x Number of Variables | 0.01467 |
| Primary Loadings x Factor Correlation x Sample Size | 0.00840 |
| Factor Correlation x Proportion of substantive cross-loadings | 0.00690 |
| Primary Loadings x Number of Factors | 0.00622 |
| Number of Factors x Number of Variables x Proportion of substantive cross-loadings | 0.00442 |
| Number of Factors x Proportion of substantive cross-loadings | 0.00392 |
| Primary Loadings x Factor Correlation x Number of Variables | 0.00258 |
| Factor Correlation x Number of Factors | 0.00235 |
| Number of Variables x Proportion of substantive cross-loadings | 0.00201 |
| Primary Loadings x Proportion of substantive cross-loadings x Sample Size | 0.00185 |
| Number of Factors x Number of Variables | 0.00179 |
| Primary Loadings x Factor Correlation x Proportion of substantive cross-loadings | 0.00142 |
| Factor Correlation x Number of Variables x Sample Size | 0.00108 |
| Primary Loadings x Factor Correlation x Number of Factors x Sample Size | 0.00086 |
| Primary Loadings x Proportion of substantive cross-loadings | 0.00058 |
| Primary Loadings x Number of Variables x Sample Size | 0.00057 |
| Number of Factors | 0.00052 |
| Number of Variables x Sample Size | 0.00052 |
| Primary Loadings x Factor Correlation x Number of Variables x Sample Size | 0.00045 |
| Proportion of substantive cross-loadings x Sample Size | 0.00043 |
| Factor Correlation x Sample Size | 0.00033 |
| Data Category | 0.00032 |
| Primary Loadings x Number of Variables x Proportion of substantive cross-loadings | 0.00030 |
| Factor Correlation x Proportion of substantive cross-loadings x Sample Size | 0.00028 |
| Factor Correlation x Number of Variables x Proportion of substantive cross-loadings | 0.00027 |
| Data Category x Primary Loadings x Sample Size | 0.00024 |
| Factor Correlation x Number of Factors x Number of Variables | 0.00024 |
| Primary Loadings x Number of Variables | 0.00016 |
| Primary Loadings x Factor Correlation x Number of Variables x Proportion of substantive cross-loadings x Sample Size | 0.00014 |
| Number of Factors x Sample Size | 0.00014 |
| Primary Loadings x Number of Factors x Number of Variables x Proportion of substantive cross-loadings | 0.00013 |
| Primary Loadings x Factor Correlation x Number of Factors x Proportion of substantive cross-loadings | 0.00011 |
| Factor Correlation x Number of Factors x Number of Variables x Proportion of substantive cross-loadings | 0.00010 |
| Primary Loadings x Number of Factors x Sample Size | 0.00010 |
| Primary Loadings x Number of Factors x Number of Variables x Proportion of substantive cross-loadings x Sample Size | 0.00010 |
| Data Category x Factor Correlation x Number of Variables | 0.00008 |
| Factor Correlation x Number of Factors x Number of Variables x Sample Size | 0.00007 |
| Number of Variables x Proportion of substantive cross-loadings x Sample Size | 0.00007 |
| Factor Correlation x Number of Factors x Sample Size | 0.00007 |
| Data Category x Factor Correlation | 0.00006 |
| Primary Loadings x Factor Correlation x Number of Variables x Proportion of substantive cross-loadings | 0.00004 |
| Primary Loadings x Factor Correlation x Number of Factors x Number of Variables x Sample Size | 0.00004 |
| Data Category x Factor Correlation x Sample Size | 0.00004 |
| Primary Loadings x Factor Correlation x Number of Factors x Number of Variables x Proportion of substantive cross-loadings | 0.00003 |
| Primary Loadings x Number of Factors x Number of Variables | 0.00003 |
| Data Category x Primary Loadings x Factor Correlation x Number of Variables | 0.00003 |
| Data Category x Primary Loadings x Number of Factors x Number of Variables x Proportion of substantive cross-loadings | 0.00002 |
| Number of Factors x Number of Variables x Proportion of substantive cross-loadings x Sample Size | 0.00002 |
| Factor Correlation x Number of Variables x Proportion of substantive cross-loadings x Sample Size | 0.00002 |
| Primary Loadings x Number of Factors x Proportion of substantive cross-loadings | 0.00002 |
| Data Category x Primary Loadings x Number of Factors x Proportion of substantive cross-loadings | 0.00002 |
| Data Category x Number of Factors | 0.00002 |
| Data Category x Primary Loadings | 0.00002 |
| Data Category x Number of Variables x Sample Size | 0.00002 |
| Data Category x Primary Loadings x Number of Variables x Sample Size | 0.00002 |
| Primary Loadings x Factor Correlation x Proportion of substantive cross-loadings x Sample Size | 0.00002 |
| Number of Factors x Proportion of substantive cross-loadings x Sample Size | 0.00002 |
| Primary Loadings x Factor Correlation x Number of Factors x Proportion of substantive cross-loadings x Sample Size | 0.00001 |
| Data Category x Primary Loadings x Number of Variables | 0.00001 |
| Data Category x Primary Loadings x Number of Factors x Sample Size | 0.00001 |
| Primary Loadings x Number of Factors x Number of Variables x Sample Size | 0.00001 |
| Data Category x Number of Variables | 0.00001 |
| Data Category x Primary Loadings x Number of Factors | 0.00001 |
| Data Category x Primary Loadings x Number of Variables x Proportion of substantive cross-loadings | 0.00001 |
| Data Category x Number of Factors x Number of Variables x Sample Size | 0.00001 |
| Data Category x Factor Correlation x Number of Variables x Proportion of substantive cross-loadings | 0.00001 |
| Data Category x Primary Loadings x Proportion of substantive cross-loadings | 0.00001 |
| Primary Loadings x Factor Correlation x Number of Factors | 0.00001 |
| Data Category x Proportion of substantive cross-loadings x Sample Size | 0.00001 |
| Data Category x Primary Loadings x Proportion of substantive cross-loadings x Sample Size | 0.00001 |
| Primary Loadings x Number of Variables x Proportion of substantive cross-loadings x Sample Size | 0.00001 |
| Data Category x Number of Factors x Number of Variables | 0.00001 |
| Data Category x Primary Loadings x Number of Factors x Number of Variables x Sample Size | <0.00000 |
| Factor Correlation x Number of Factors x Number of Variables x Proportion of substantive cross-loadings x Sample Size | <0.00000 |
| Data Category x Primary Loadings x Factor Correlation x Number of Factors x Number of Variables | <0.00000 |
| Data Category x Sample Size | <0.00000 |
| Data Category x Number of Variables x Proportion of substantive cross-loadings | <0.00000 |
| Data Category x Factor Correlation x Number of Variables x Proportion of substantive cross-loadings x Sample Size | <0.00000 |
| Data Category x Number of Variables x Proportion of substantive cross-loadings x Sample Size | <0.00000 |
| Data Category x Primary Loadings x Factor Correlation x Number of Factors x Proportion of substantive cross-loadings x Sample Size | <0.00000 |
| Data Category x Number of Factors x Number of Variables x Proportion of substantive cross-loadings | <0.00000 |
| Primary Loadings x Factor Correlation x Number of Factors x Number of Variables | <0.00000 |
| Data Category x Primary Loadings x Factor Correlation x Proportion of substantive cross-loadings | <0.00000 |
| Data Category x Number of Factors x Sample Size | <0.00000 |
| Factor Correlation x Number of Factors x Proportion of substantive cross-loadings | <0.00000 |
| Data Category x Number of Factors x Number of Variables x Proportion of substantive cross-loadings x Sample Size | <0.00000 |
| Data Category x Factor Correlation x Number of Factors x Proportion of substantive cross-loadings x Sample Size | <0.00000 |
| Data Category x Primary Loadings x Number of Variables x Proportion of substantive cross-loadings x Sample Size | <0.00000 |
| Data Category x Number of Factors x Proportion of substantive cross-loadings | <0.00000 |
| Data Category x Primary Loadings x Number of Factors x Number of Variables | <0.00000 |
| Data Category x Primary Loadings x Factor Correlation | <0.00000 |
| Data Category x Factor Correlation x Number of Variables x Sample Size | <0.00000 |
| Data Category x Primary Loadings x Factor Correlation x Number of Variables x Sample Size | <0.00000 |
| Data Category x Factor Correlation x Proportion of substantive cross-loadings | <0.00000 |
| Data Category x Primary Loadings x Factor Correlation x Number of Factors x Number of Variables x Proportion of substantive cross-loadings x Sample Size | <0.00000 |
| Factor Correlation x Number of Factors x Proportion of substantive cross-loadings x Sample Size | <0.00000 |
| Data Category x Primary Loadings x Factor Correlation x Number of Factors | <0.00000 |
| Data Category x Number of Factors x Proportion of substantive cross-loadings x Sample Size | <0.00000 |
| Data Category x Factor Correlation x Number of Factors x Number of Variables x Proportion of substantive cross-loadings | <0.00000 |
| Data Category x Primary Loadings x Factor Correlation x Proportion of substantive cross-loadings x Sample Size | <0.00000 |
| Primary Loadings x Number of Factors x Proportion of substantive cross-loadings x Sample Size | <0.00000 |
| Data Category x Factor Correlation x Number of Factors x Proportion of substantive cross-loadings | <0.00000 |
| Data Category x Factor Correlation x Number of Factors x Number of Variables x Sample Size | <0.00000 |
| Number of Factors x Number of Variables x Sample Size | <0.00000 |
| Data Category x Primary Loadings x Factor Correlation x Number of Variables x Proportion of substantive cross-loadings | <0.00000 |
| Data Category x Factor Correlation x Number of Factors x Number of Variables | <0.00000 |
| Data Category x Primary Loadings x Factor Correlation x Number of Variables x Proportion of substantive cross-loadings x Sample Size | <0.00000 |
| Data Category x Primary Loadings x Factor Correlation x Number of Factors x Sample Size | <0.00000 |
| Data Category x Proportion of substantive cross-loadings | <0.00000 |
| Data Category x Factor Correlation x Proportion of substantive cross-loadings x Sample Size | <0.00000 |
| Data Category x Primary Loadings x Factor Correlation x Number of Factors x Proportion of substantive cross-loadings | <0.00000 |
| Data Category x Primary Loadings x Factor Correlation x Number of Factors x Number of Variables x Proportion of substantive cross-loadings | <0.00000 |
| Data Category x Factor Correlation x Number of Factors x Number of Variables x Proportion of substantive cross-loadings x Sample Size | <0.00000 |
| Data Category x Primary Loadings x Factor Correlation x Number of Factors x Number of Variables x Sample Size | <0.00000 |
| Data Category x Factor Correlation x Number of Factors | <0.00000 |
| Data Category x Primary Loadings x Number of Factors x Number of Variables x Proportion of substantive cross-loadings x Sample Size | <0.00000 |
| Primary Loadings x Factor Correlation x Number of Factors x Number of Variables x Proportion of substantive cross-loadings x Sample Size | <0.00000 |
| Data Category x Factor Correlation x Number of Factors x Sample Size | <0.00000 |
| Data Category x Primary Loadings x Factor Correlation x Sample Size | <0.00000 |
| Data Category x Primary Loadings x Number of Factors x Proportion of substantive cross-loadings x Sample Size | <0.00000 |
| Note. In the ANOVA, the Omega Index was the dependent variable, while variables manipulated in the simulation study (data categories; number of factors; number of variables per factor; factor correlation; size of primary factor loadings; proportion of observed variables with substantive cross-loadings; and sample size) were the independent variables. All main effects, two-, three-, four-, five-, six- and seven-way interactions were examined. | |


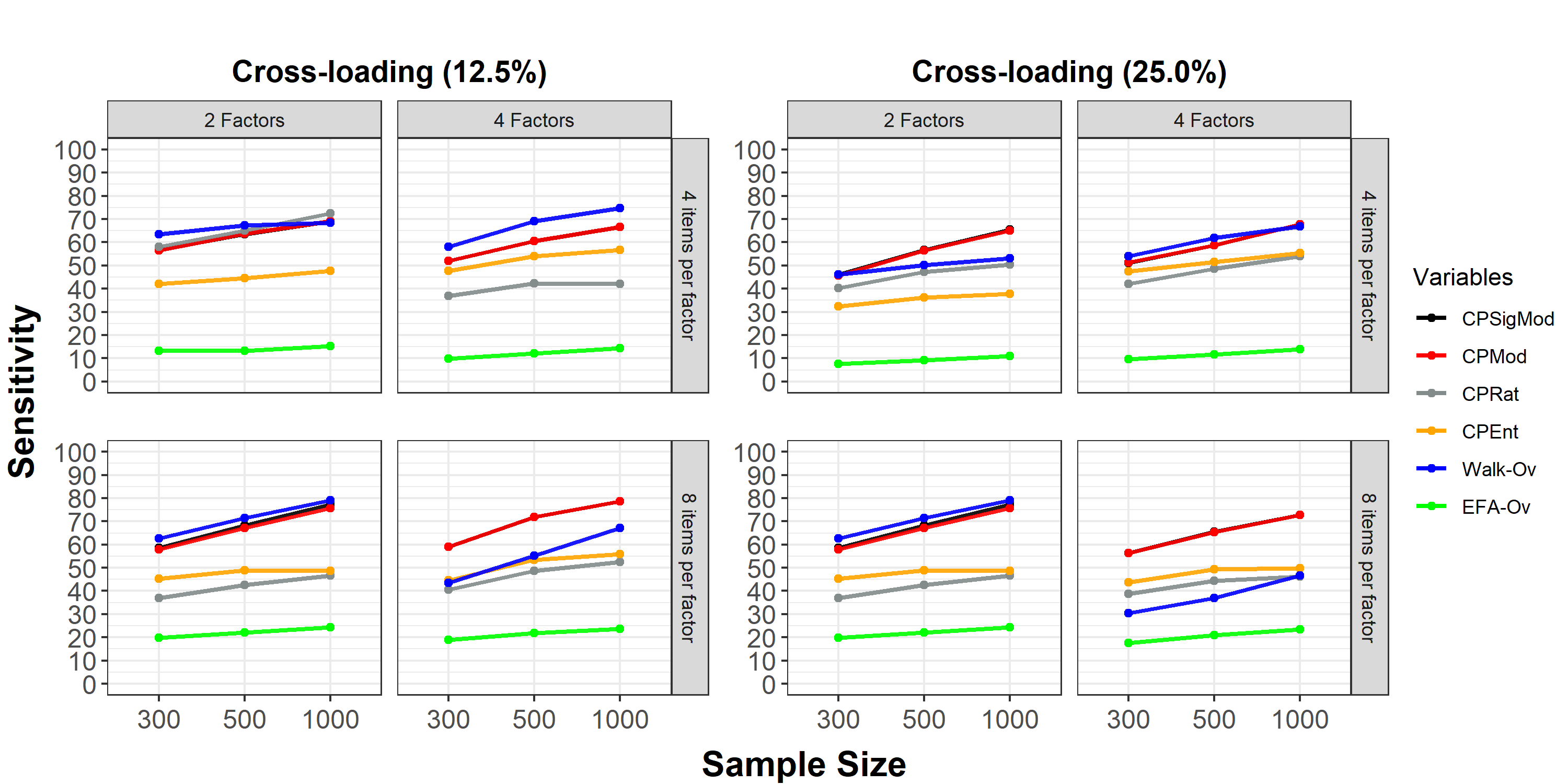


Supplementary Figure 1: Sensitivity according to sample size. Note. CPSigMod = CP algorithm with maximisation of the signed fuzzy modularity for signed weighted networks; CPMod = CP algorithm with maximisation of the fuzzy modularity for signed weighted networks; CPRat = CP algorithm with minimisation of the ratio between the two largest communities when the ratio is above or equal 2; CPEnt = CP algorithm with maximisation of entropy; Walk-Ov = Walktrap algorithm with overlapping nodes identified through network loadings >= |.15|; EFA-Ov = Exploratory Factor Analysis with overlapping nodes identified through factor loadings >= |.40|. The x-axis indicates sample size. The y-axis indicates the sensitivity. Higher values indicate higher sensitivity of the algorithm to detect overlapping symptoms.


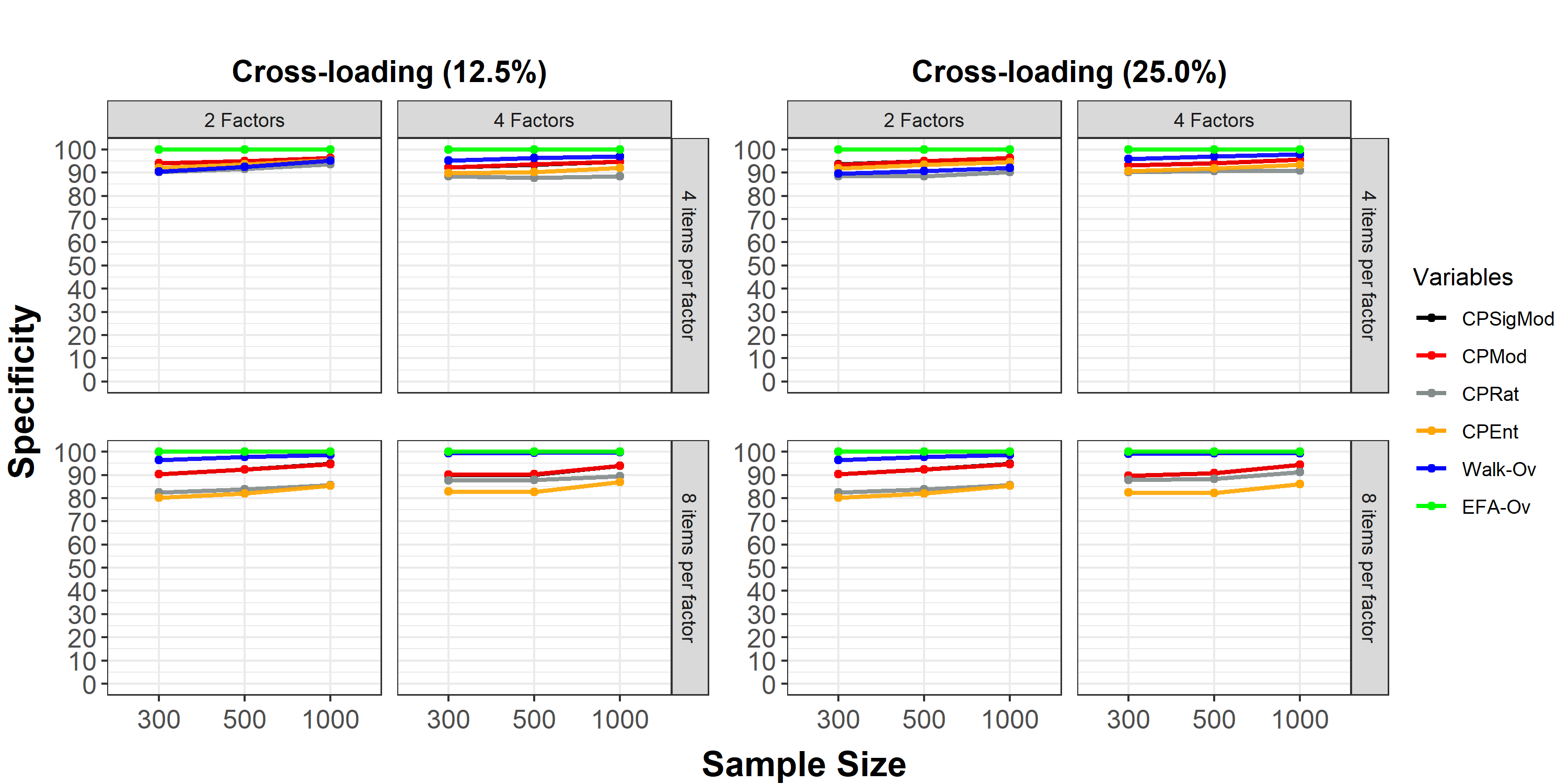


Supplementary Figure 2: Specificity according to sample size. Note. CPSigMod = CP algorithm with maximisation of the signed fuzzy modularity for signed weighted networks; CPMod = CP algorithm with maximisation of the fuzzy modularity for signed weighted networks; CPRat = CP algorithm with minimisation of the ratio between the two largest communities when the ratio is above or equal 2; CPEnt = CP algorithm with maximisation of entropy; Walk-Ov = Walktrap algorithm with overlapping nodes identified through network loadings >= |.15|; EFA-Ov = Exploratory Factor Analysis with overlapping nodes identified through factor loadings >= |.40|. The x-axis indicates sample size. The y-axis indicates the specificity. Higher values indicate higher specificity of the algorithm to detect non-overlapping symptoms.


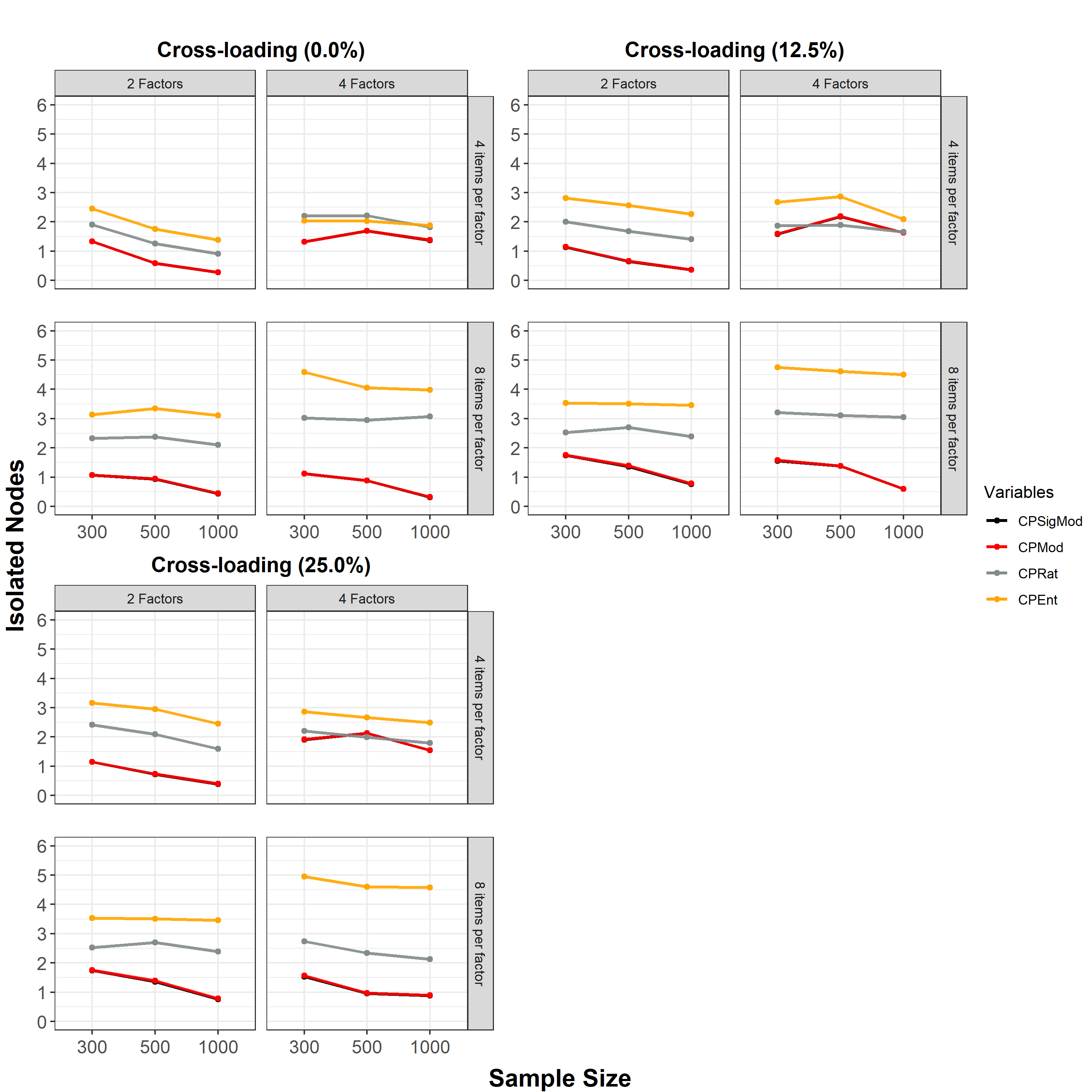


Supplementary Figure 3: Number of isolated nodes according to sample size. Note. CPSigMod = CP algorithm with maximisation of the signed fuzzy modularity for signed weighted networks; CPMod = CP algorithm with maximisation of the fuzzy modularity for signed weighted networks; CPRat = CP algorithm with minimisation of the ratio between the two largest communities when the ratio is above or equal 2; CPEnt = CP algorithm with maximisation of entropy; Walk-Ov = Walktrap algorithm with overlapping nodes identified through network loadings >= |.15|; EFA-Ov = Exploratory Factor Analysis with overlapping nodes identified through factor loadings >= |.40|. The x-axis indicates sample size. The y-axis indicates the number of isolated nodes. Lower values indicate that a lower number of nodes were not assigned to any community (i.e. isolated nodes) by the algorithm.


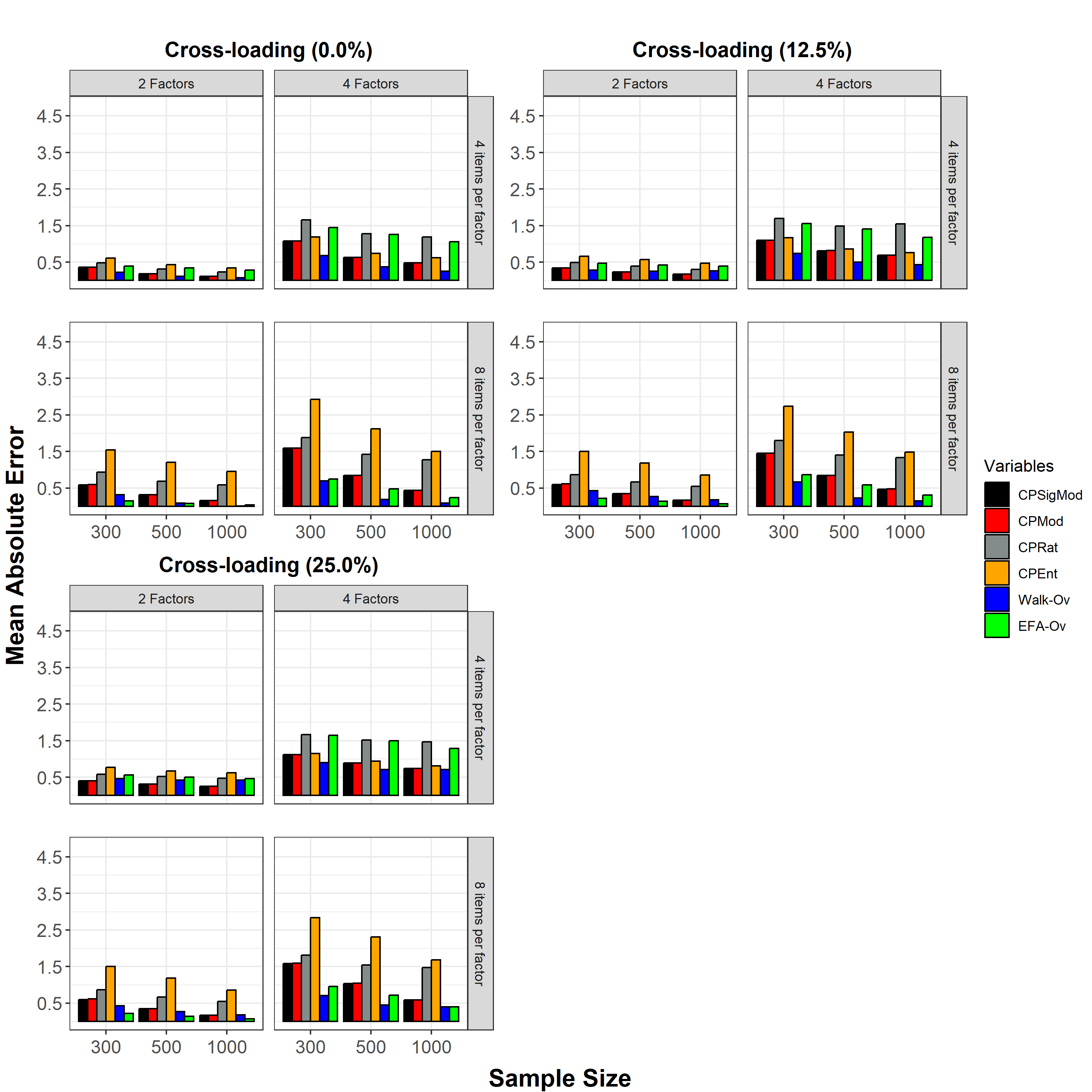


Supplementary Figure 4: Mean absolute error according to sample size. Note. CPSigMod = CP algorithm with maximisation of the signed fuzzy modularity for signed weighted networks; CPMod = CP algorithm with maximisation of the fuzzy modularity for signed weighted networks; CPRat = CP algorithm with minimisation of the ratio between the two largest communities when the ratio is above or equal 2; CPEnt = CP algorithm with maximisation of entropy; Walk-Ov = Walktrap algorithm with overlapping nodes identified through network loadings >= |.15|; EFA-Ov = Exploratory Factor Analysis with overlapping nodes identified through factor loadings >= |.40|. The x-axis indicates sample size. The y-axis indicates the mean absolute error. Higher values indicate higher absolute error regarding the number of identified dimensions.


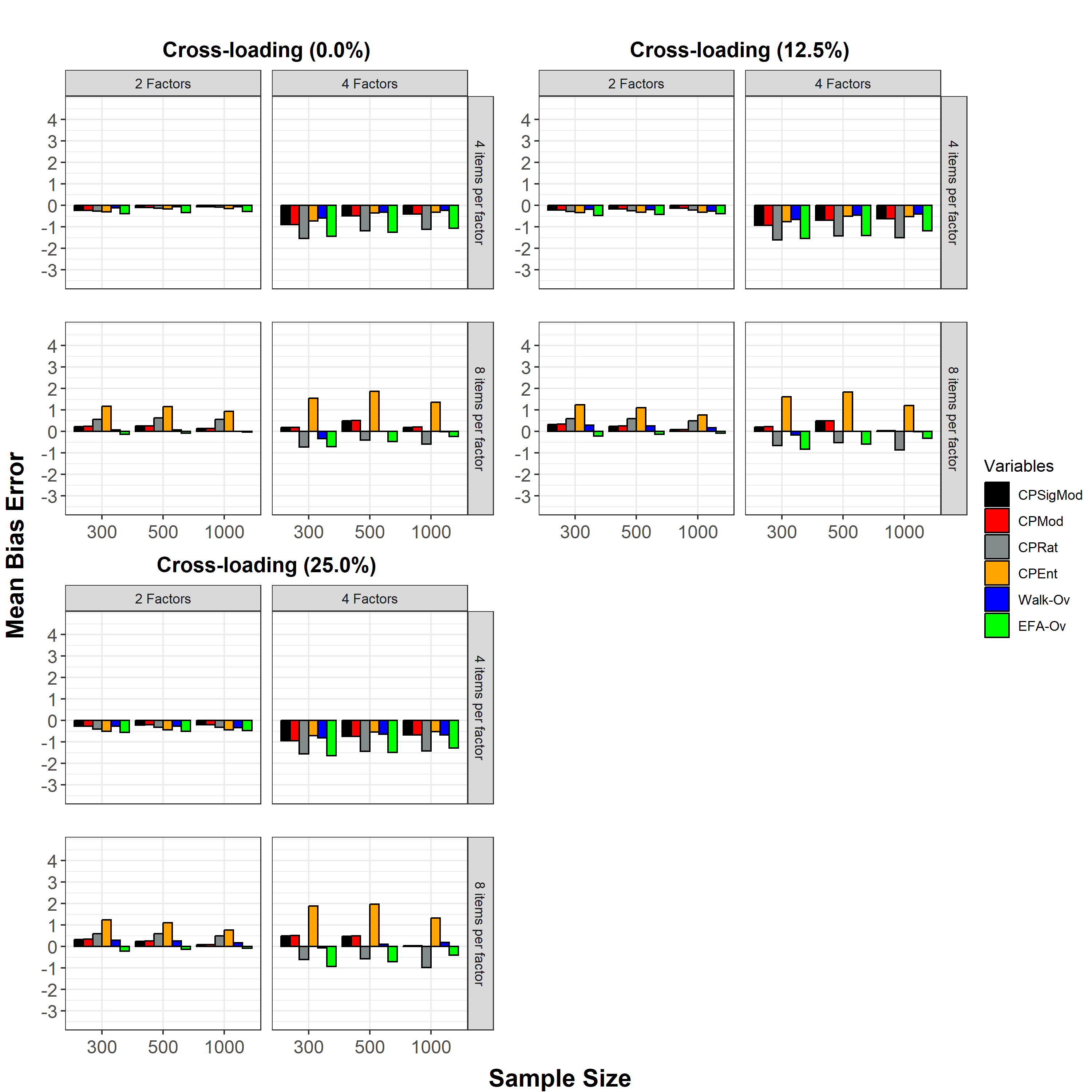


Supplementary Figure 5: Mean bias error according to sample size according to sample size. Note. CPSigMod = CP algorithm with maximisation of the signed fuzzy modularity for signed weighted networks; CPMod = CP algorithm with maximisation of the fuzzy modularity for signed weighted networks; CPRat = CP algorithm with minimisation of the ratio between the two largest communities when the ratio is above or equal 2; CPEnt = CP algorithm with maximisation of entropy; Walk-Ov = Walktrap algorithm with overlapping nodes identified through network loadings >= |.15|; EFA-Ov = Exploratory Factor Analysis with overlapping nodes identified through factor loadings >= |.40|. The x-axis indicates sample size. The y-axis indicates the mean bias error. Values higher or lower than zero indicate more bias in terms of a higher or lower number of dimensions identified, respectively.


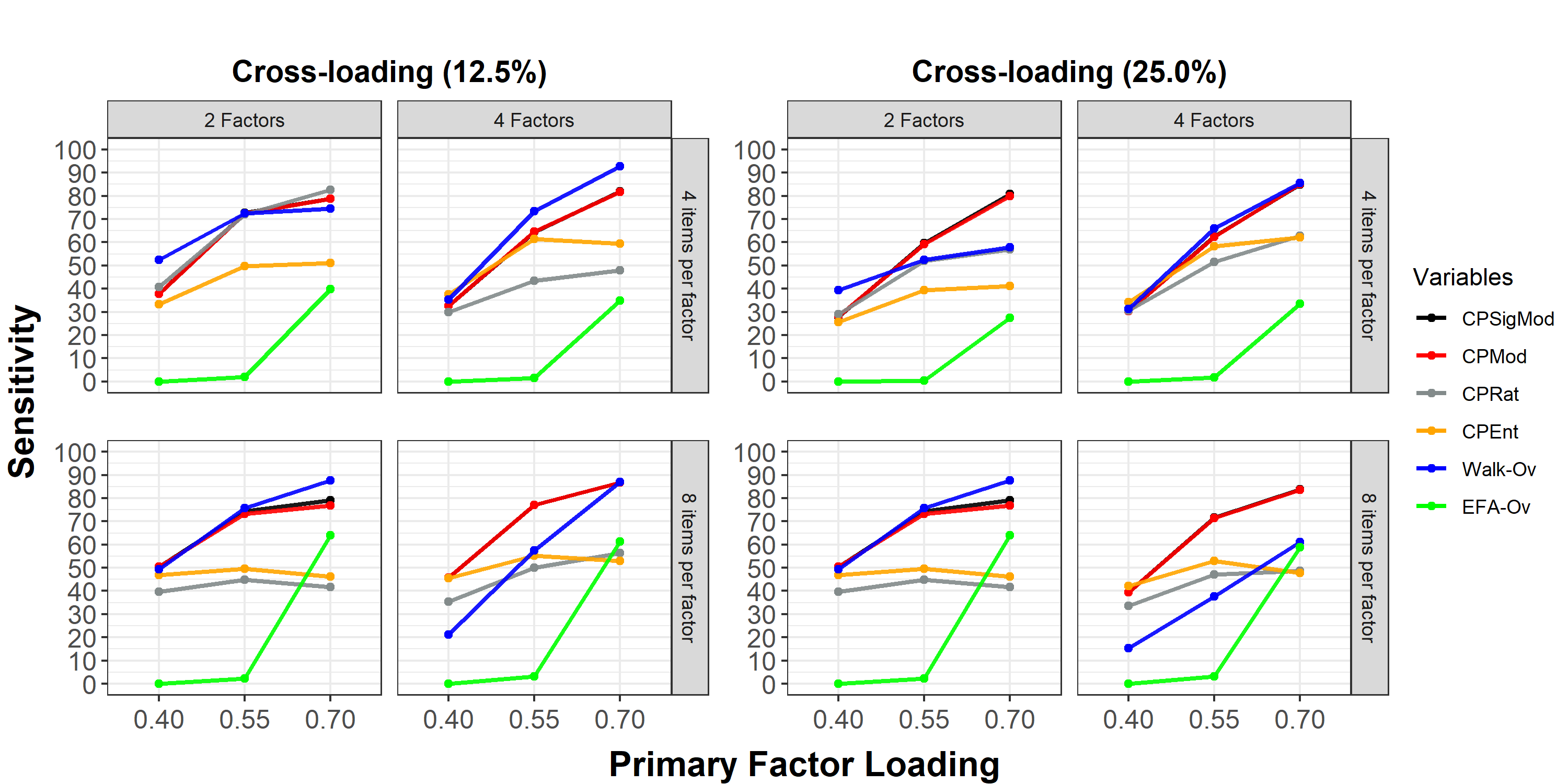


Supplementary Figure 6: Sensitivity according to primary factor loading. Note. CPSigMod = CP algorithm with maximisation of the signed fuzzy modularity for signed weighted networks; CPMod = CP algorithm with maximisation of the fuzzy modularity for signed weighted networks; CPRat = CP algorithm with minimisation of the ratio between the two largest communities when the ratio is above or equal 2; CPEnt = CP algorithm with maximisation of entropy; Walk-Ov = Walktrap algorithm with overlapping nodes identified through network loadings >= |.15|; EFA-Ov = Exploratory Factor Analysis with overlapping nodes identified through factor loadings >= |.40|. The x-axis indicates primary factor loading. The y-axis indicates the sensitivity. Higher values indicate higher sensitivity of the algorithm to detect overlapping symptoms.


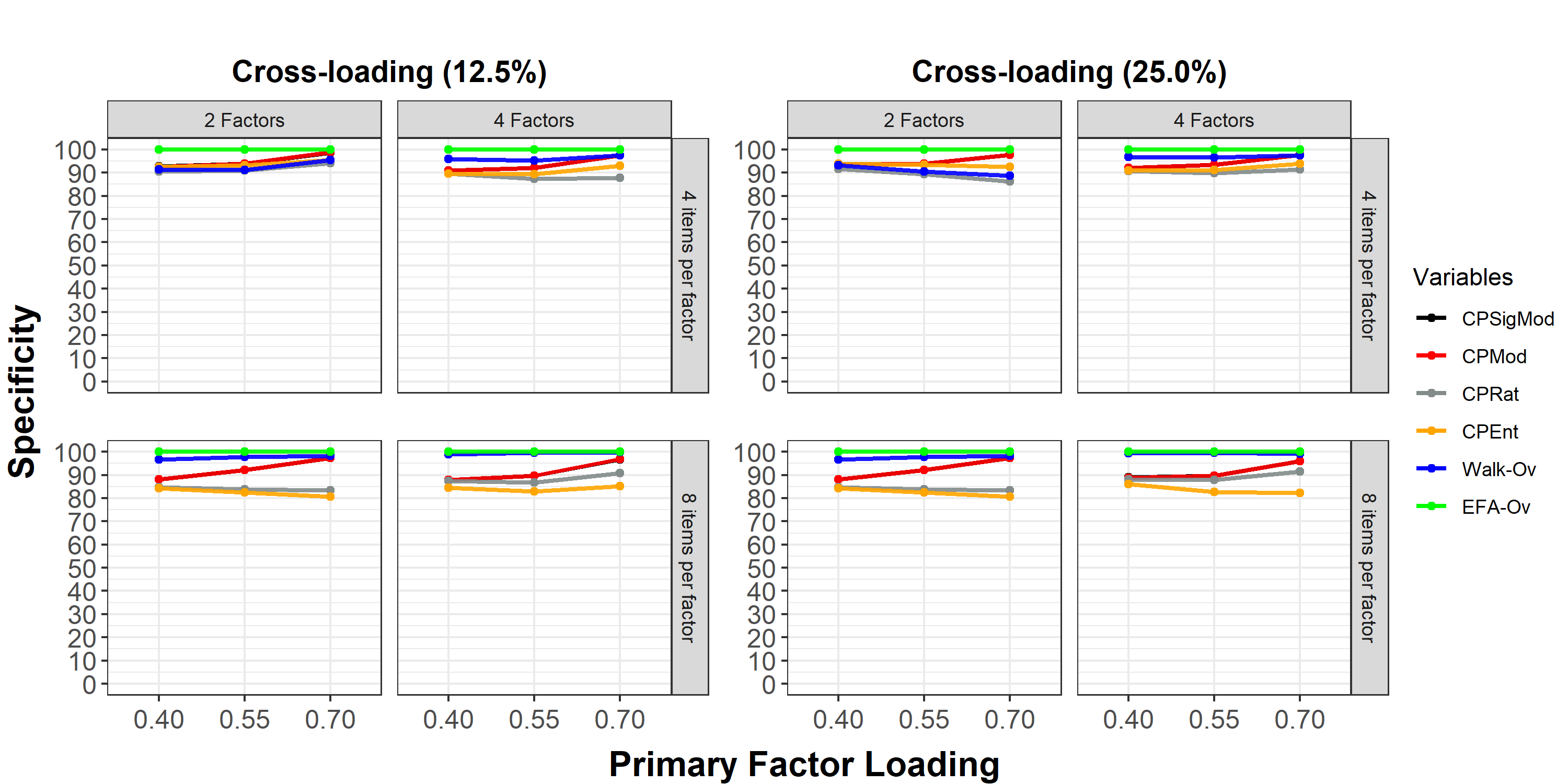


Supplementary Figure 7: Specificity according to primary factor loading. Note. CPSigMod = CP algorithm with maximisation of the signed fuzzy modularity for signed weighted networks; CPMod = CP algorithm with maximisation of the fuzzy modularity for signed weighted networks; CPRat = CP algorithm with minimisation of the ratio between the two largest communities when the ratio is above or equal 2; CPEnt = CP algorithm with maximisation of entropy; Walk-Ov = Walktrap algorithm with overlapping nodes identified through network loadings >= |.15|; EFA-Ov = Exploratory Factor Analysis with overlapping nodes identified through factor loadings >= |.40|. The x-axis indicates primary factor loading. The y-axis indicates the specificity. Higher values indicate higher specificity of the algorithm to detect non-overlapping symptoms.


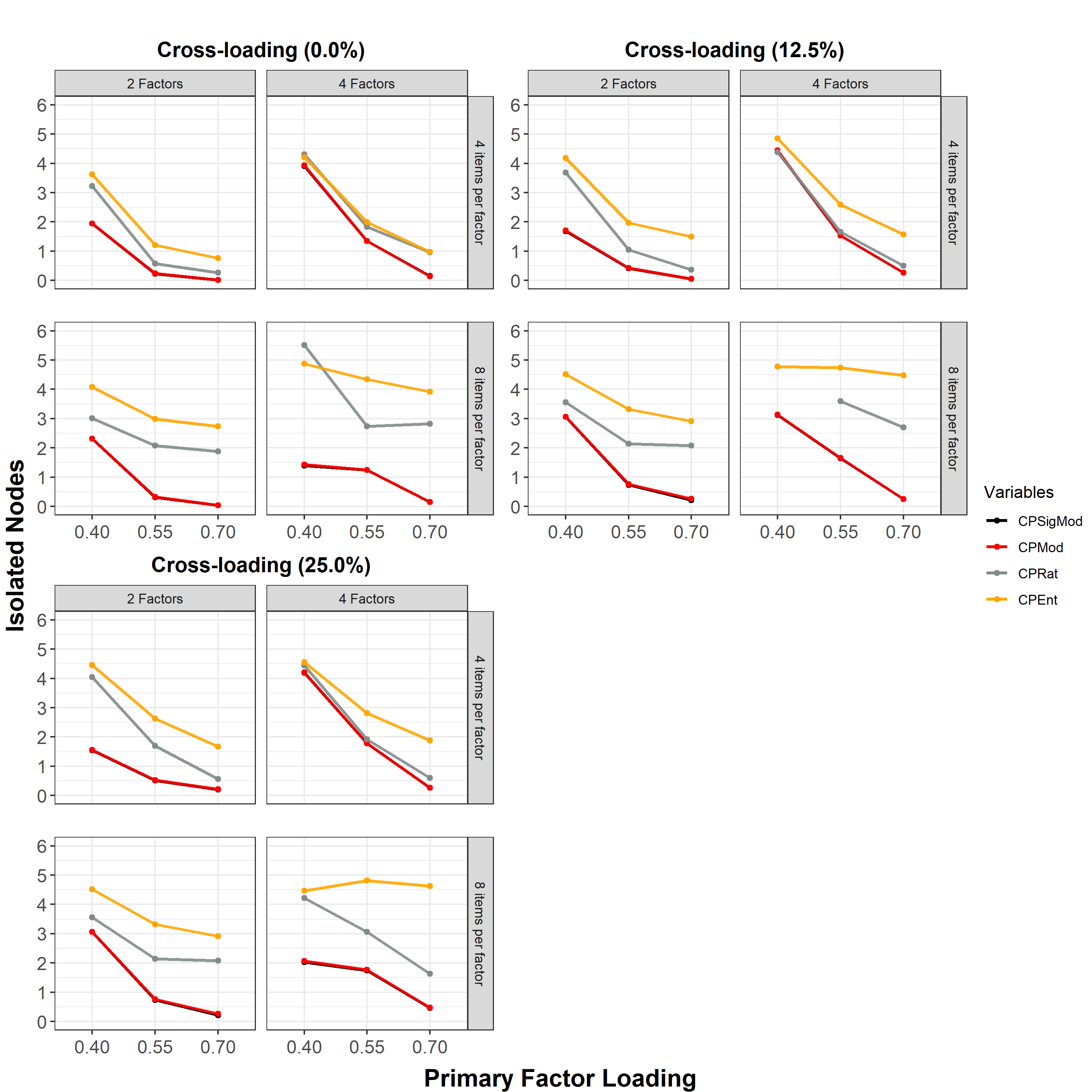


Supplementary Figure 8: Number of isolated nodes according to primary factor loading. Note. CPSigMod = CP algorithm with maximisation of the signed fuzzy modularity for signed weighted networks; CPMod = CP algorithm with maximisation of the fuzzy modularity for signed weighted networks; CPRat = CP algorithm with minimisation of the ratio between the two largest communities when the ratio is above or equal 2; CPEnt = CP algorithm with maximisation of entropy; Walk-Ov = Walktrap algorithm with overlapping nodes identified through network loadings >= |.15|; EFA-Ov = Exploratory Factor Analysis with overlapping nodes identified through factor loadings >= |.40|. The x-axis indicates primary factor loading. The y-axis indicates the number of isolated nodes. Lower values indicate that a lower number of nodes were not assigned to any community (i.e. isolated nodes) by the algorithm.


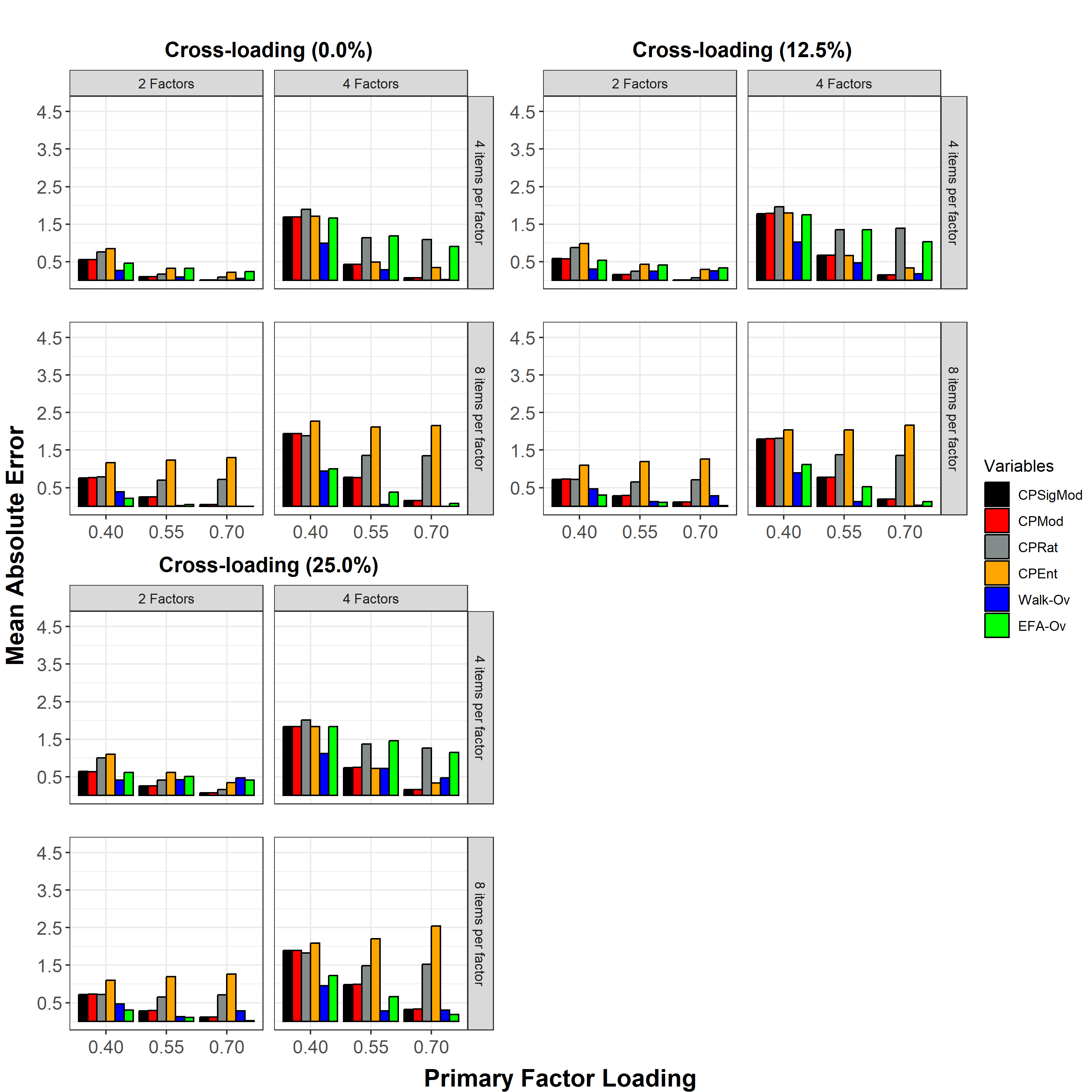


Supplementary Figure 9: Mean absolute error according to primary factor loading. Note. CPSigMod = CP algorithm with maximisation of the signed fuzzy modularity for signed weighted networks; CPMod = CP algorithm with maximisation of the fuzzy modularity for signed weighted networks; CPRat = CP algorithm with minimisation of the ratio between the two largest communities when the ratio is above or equal 2; CPEnt = CP algorithm with maximisation of entropy; Walk-Ov = Walktrap algorithm with overlapping nodes identified through network loadings >= |.15|; EFA-Ov = Exploratory Factor Analysis with overlapping nodes identified through factor loadings >= |.40|. The x-axis indicates primary factor loading. The y-axis indicates the mean absolute error. Higher values indicate higher absolute error regarding the number of identified dimensions.


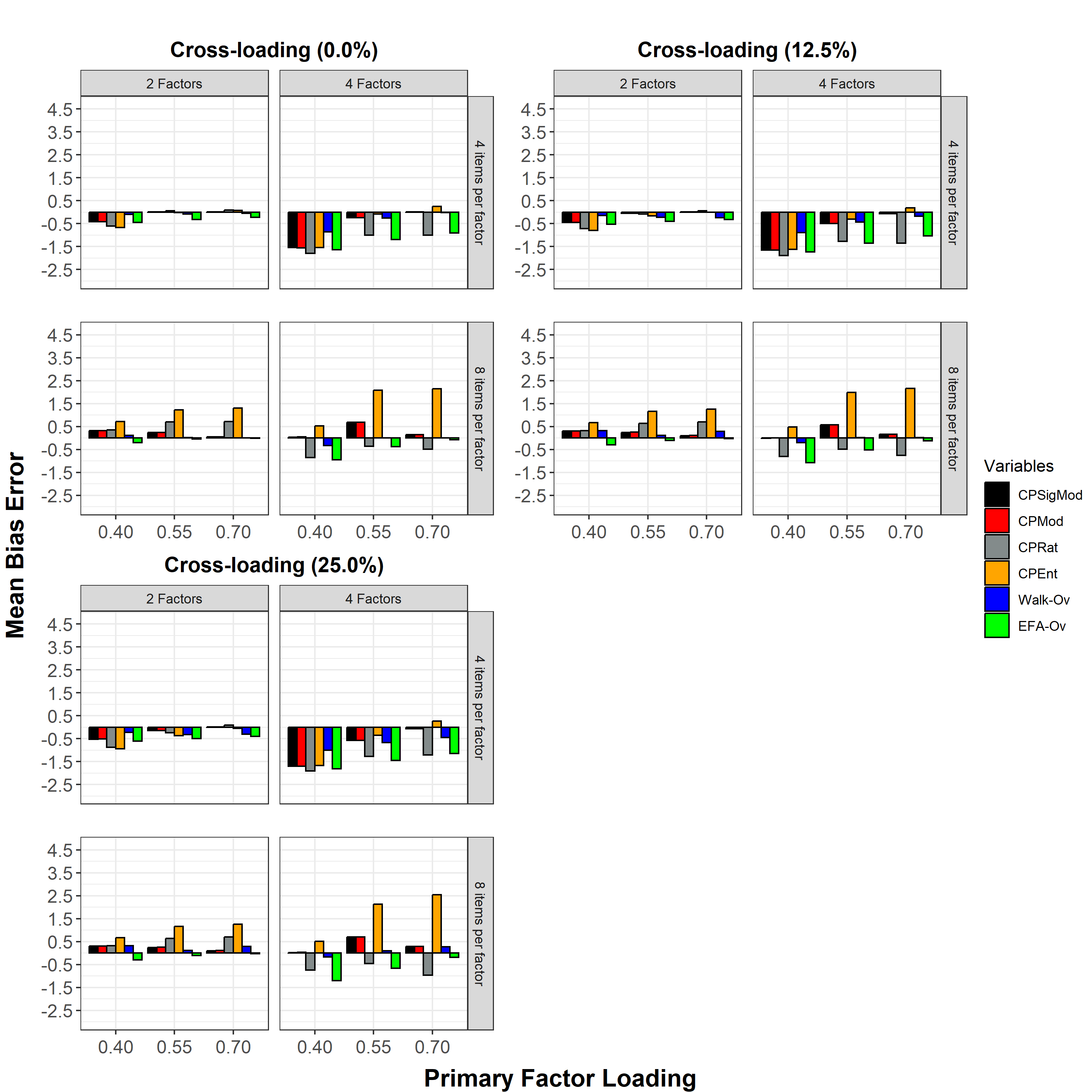


Supplementary Figure 10: Mean bias error according to primary factor loading. Note. CPSigMod = CP algorithm with maximisation of the signed fuzzy modularity for signed weighted networks; CPMod = CP algorithm with maximisation of the fuzzy modularity for signed weighted networks; CPRat = CP algorithm with minimisation of the ratio between the two largest communities when the ratio is above or equal 2; CPEnt = CP algorithm with maximisation of entropy; Walk-Ov = Walktrap algorithm with overlapping nodes identified through network loadings >= |.15|; EFA-Ov = Exploratory Factor Analysis with overlapping nodes identified through factor loadings >= |.40|. The x-axis indicates primary factor loading. The y-axis indicates the mean bias error. Values higher or lower than zero indicate more bias in terms of a higher or lower number of dimensions identified, respectively.


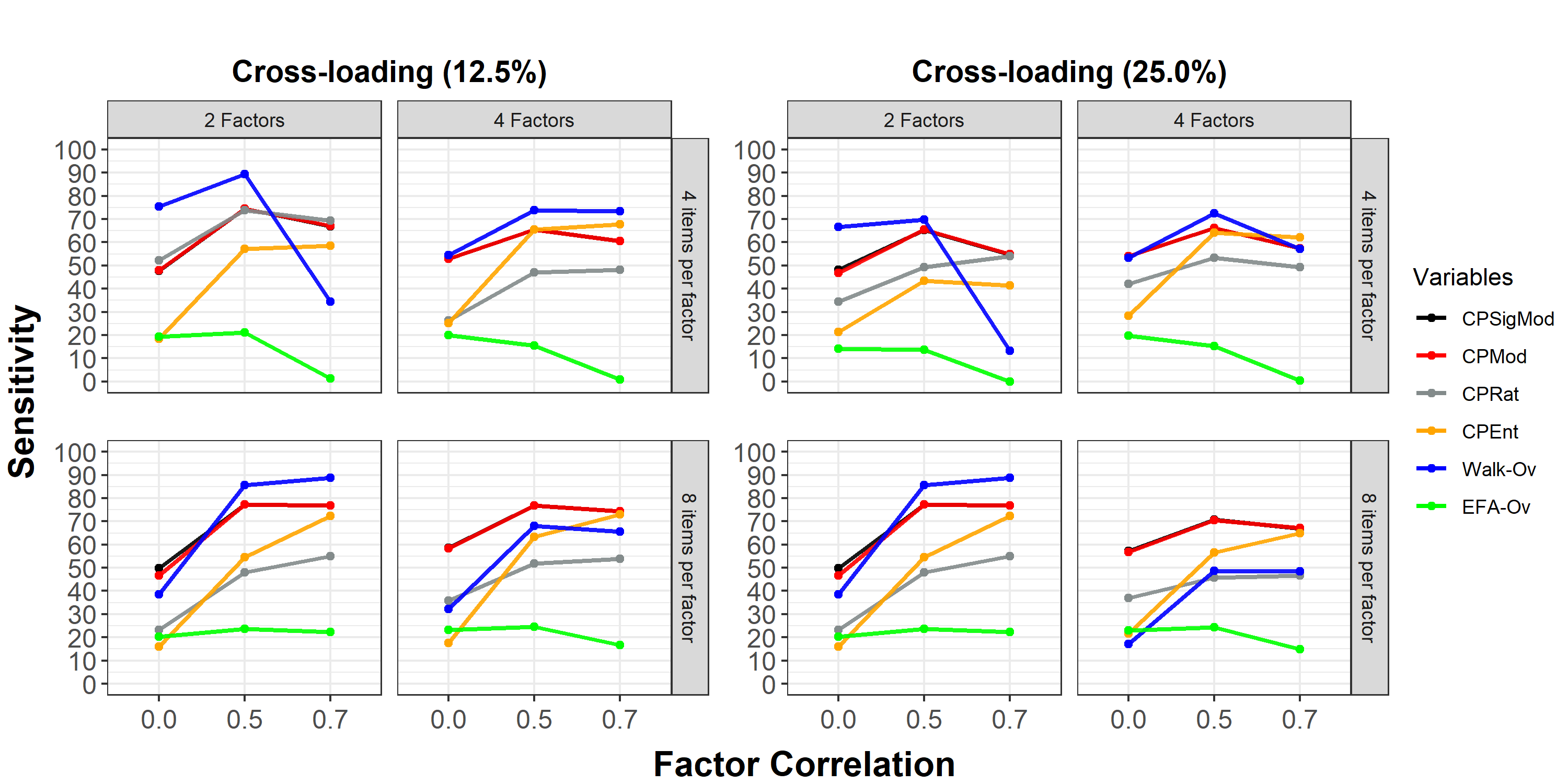


Supplementary Figure 11: Sensitivity according to factor correlation. Note. CPSigMod = CP algorithm with maximisation of the signed fuzzy modularity for signed weighted networks; CPMod = CP algorithm with maximisation of the fuzzy modularity for signed weighted networks; CPRat = CP algorithm with minimisation of the ratio between the two largest communities when the ratio is above or equal 2; CPEnt = CP algorithm with maximisation of entropy; Walk-Ov = Walktrap algorithm with overlapping nodes identified through network loadings >= |.15|; EFA-Ov = Exploratory Factor Analysis with overlapping nodes identified through factor loadings >= |.40|. The x-axis indicates factor correlation. The y-axis indicates the sensitivity. Higher values indicate higher sensitivity of the algorithm to detect overlapping symptoms.


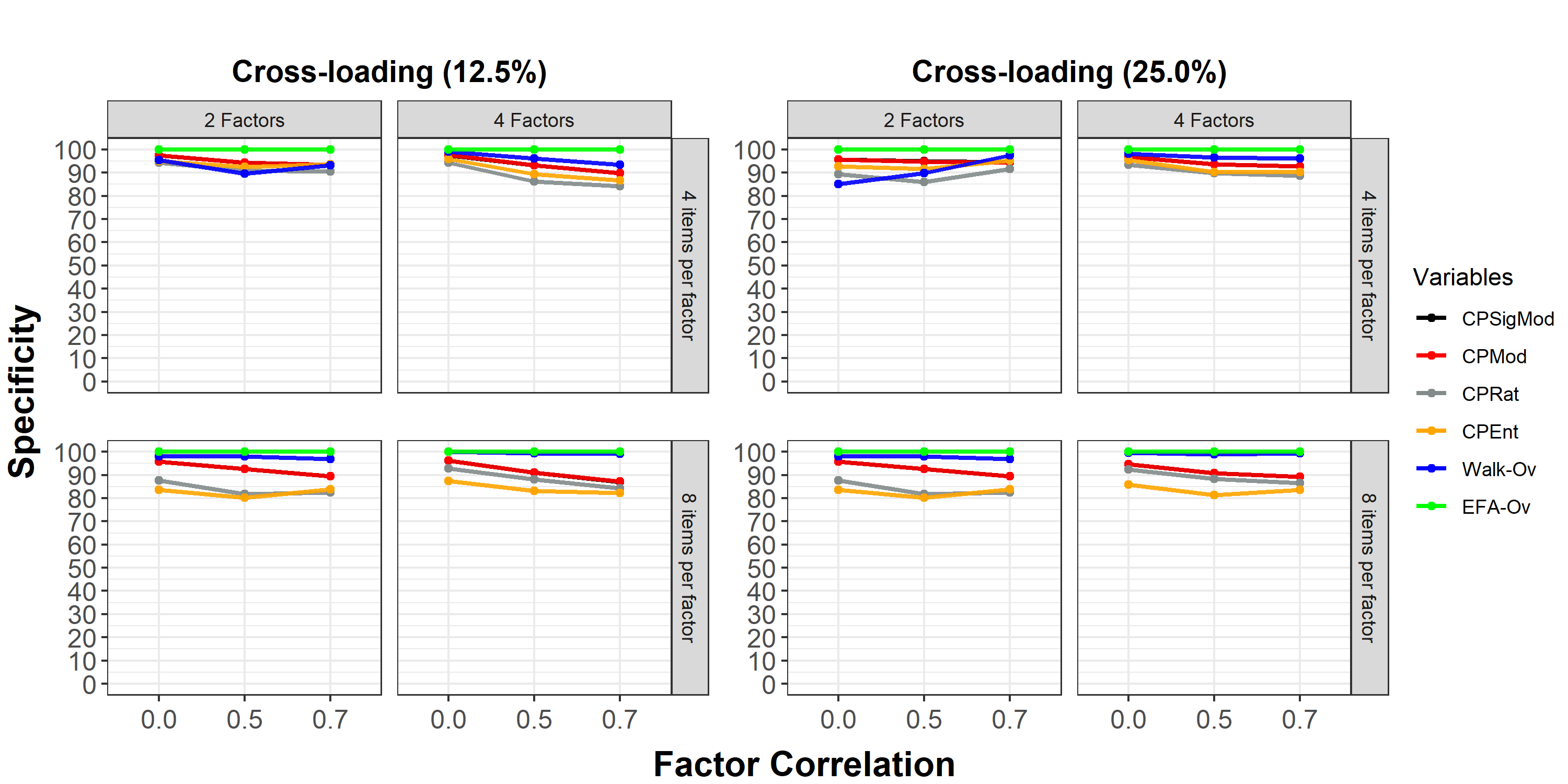


Supplementary Figure 12: Specificity according to factor correlation. Note. CPSigMod = CP algorithm with maximisation of the signed fuzzy modularity for signed weighted networks; CPMod = CP algorithm with maximisation of the fuzzy modularity for signed weighted networks; CPRat = CP algorithm with minimisation of the ratio between the two largest communities when the ratio is above or equal 2; CPEnt = CP algorithm with maximisation of entropy; Walk-Ov = Walktrap algorithm with overlapping nodes identified through network loadings >= |.15|; EFA-Ov = Exploratory Factor Analysis with overlapping nodes identified through factor loadings >= |.40|. The x-axis indicates factor correlation. The y-axis indicates the specificity. Higher values indicate higher specificity of the algorithm to detect non-overlapping symptoms.


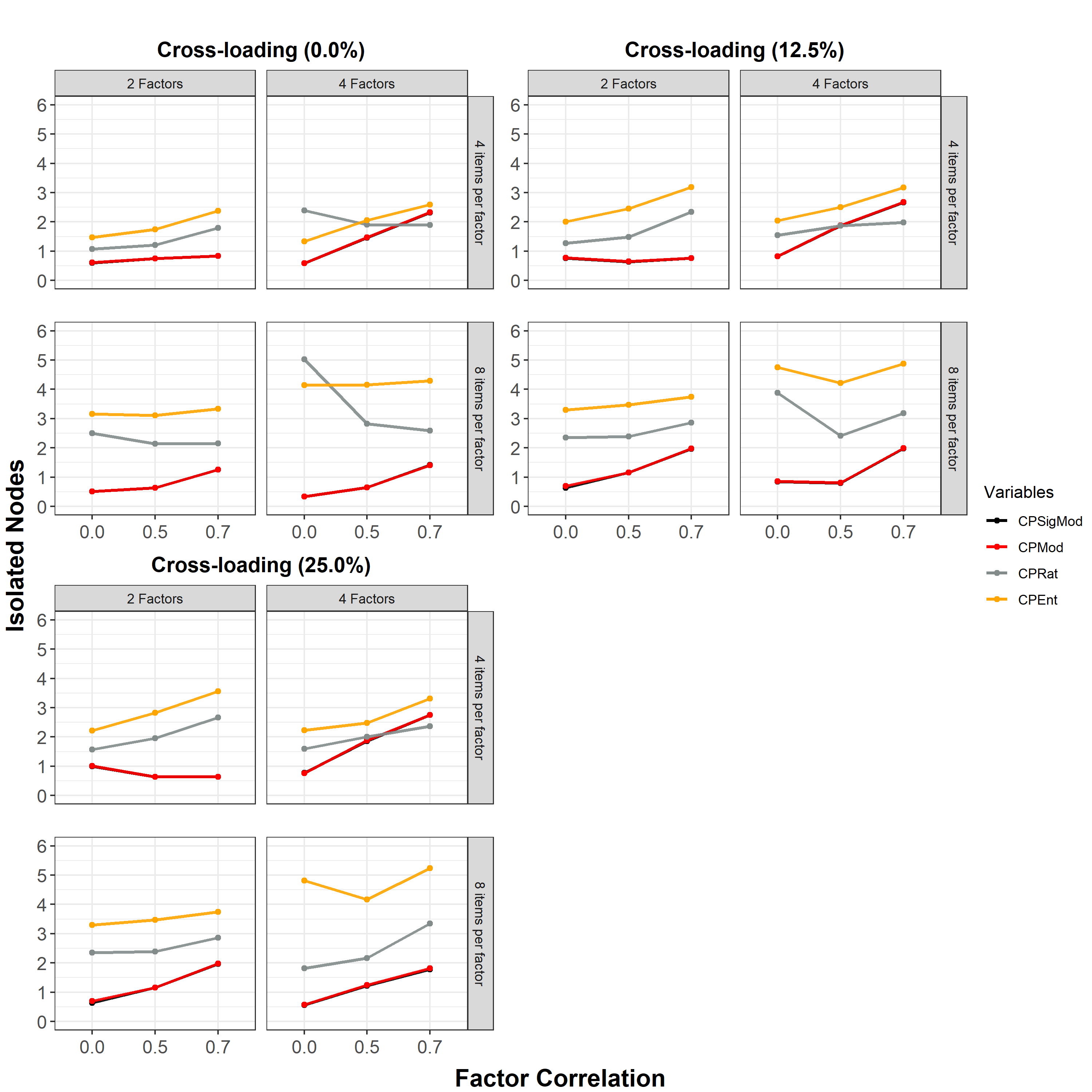


Supplementary Figure 13: Number of isolated nodes according to factor correlation. Note. CPSigMod = CP algorithm with maximisation of the signed fuzzy modularity for signed weighted networks; CPMod = CP algorithm with maximisation of the fuzzy modularity for signed weighted networks; CPRat = CP algorithm with minimisation of the ratio between the two largest communities when the ratio is above or equal 2; CPEnt = CP algorithm with maximisation of entropy; Walk-Ov = Walktrap algorithm with overlapping nodes identified through network loadings >= |.15|; EFA-Ov = Exploratory Factor Analysis with overlapping nodes identified through factor loadings >= |.40|. The x-axis indicates factor correlation. The y-axis indicates the number of isolated nodes. Lower values indicate that a lower number of nodes were not assigned to any community (i.e. isolated nodes) by the algorithm.


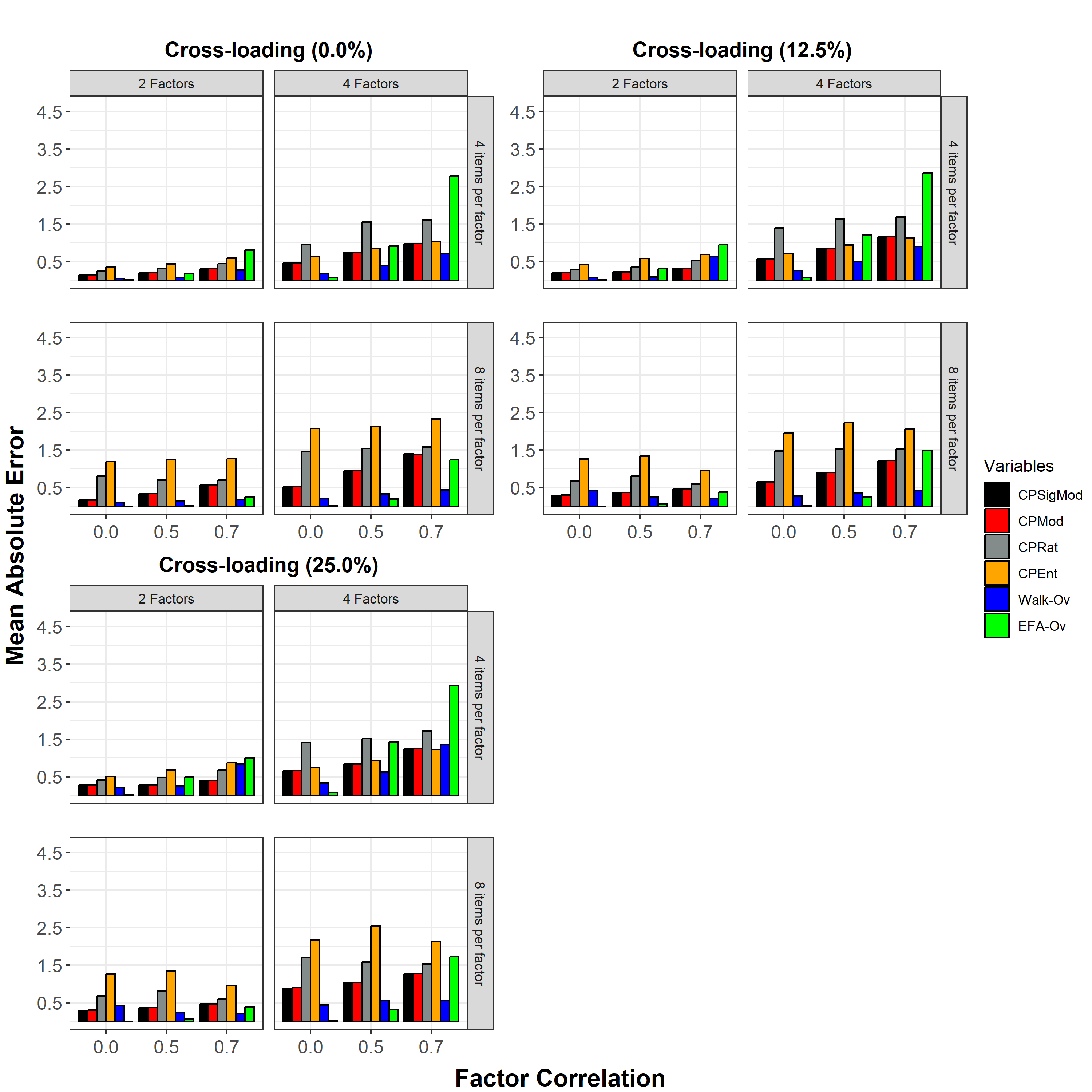


Supplementary Figure 14: Mean absolute error according to factor correlation. Note. CPSigMod = CP algorithm with maximisation of the signed fuzzy modularity for signed weighted networks; CPMod = CP algorithm with maximisation of the fuzzy modularity for signed weighted networks; CPRat = CP algorithm with minimisation of the ratio between the two largest communities when the ratio is above or equal 2; CPEnt = CP algorithm with maximisation of entropy; Walk-Ov = Walktrap algorithm with overlapping nodes identified through network loadings >= |.15|; EFA-Ov = Exploratory Factor Analysis with overlapping nodes identified through factor loadings >= |.40|. The x-axis indicates factor correlation. The y-axis indicates the mean absolute error. Higher values indicate higher absolute error regarding the number of identified dimensions.


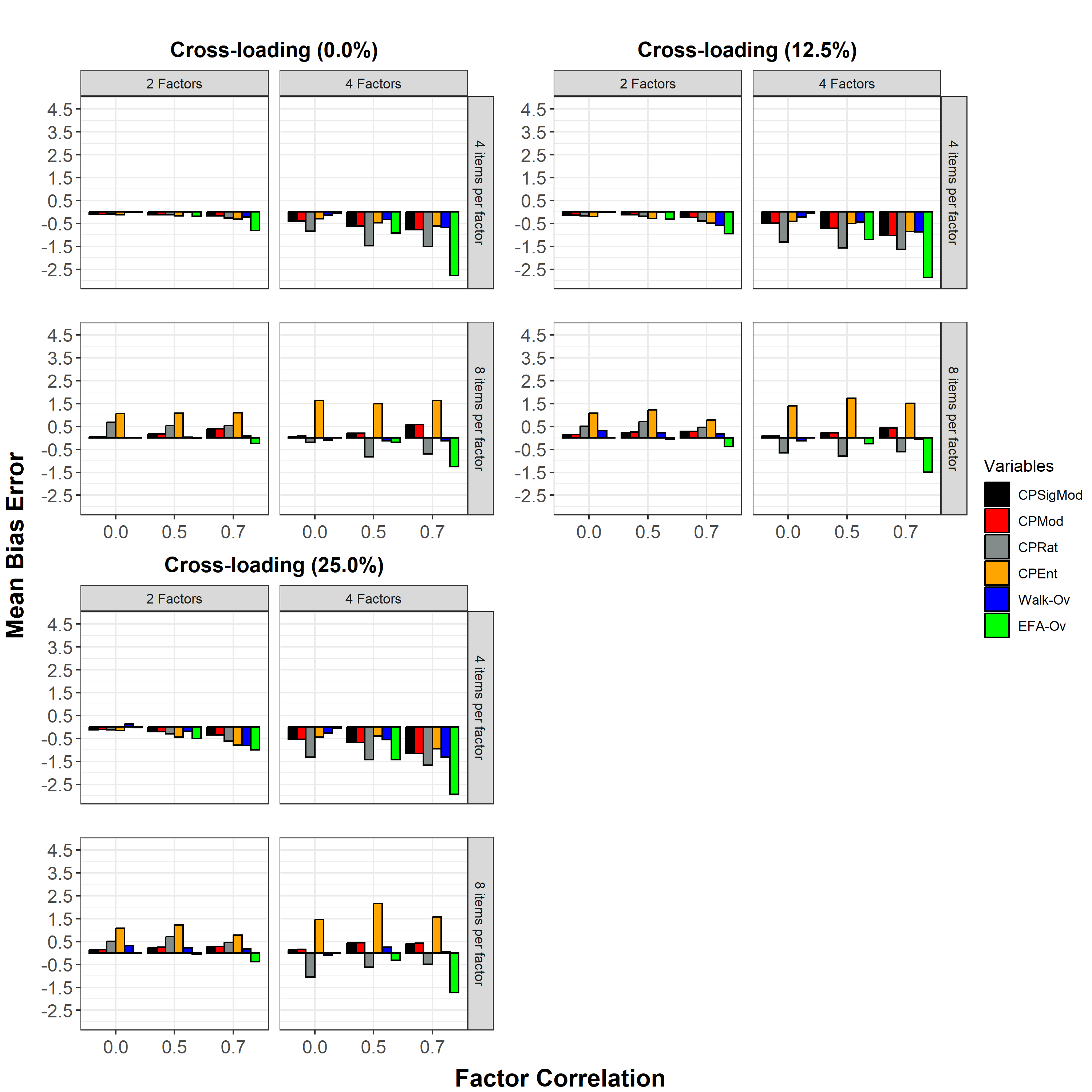


Supplementary Figure 15: Mean bias error according to factor correlation. Note. CPSigMod = CP algorithm with maximisation of the signed fuzzy modularity for signed weighted networks; CPMod = CP algorithm with maximisation of the fuzzy modularity for signed weighted networks; CPRat = CP algorithm with minimisation of the ratio between the two largest communities when the ratio is above or equal 2; CPEnt = CP algorithm with maximisation of entropy; Walk-Ov = Walktrap algorithm with overlapping nodes identified through network loadings >= |.15|; EFA-Ov = Exploratory Factor Analysis with overlapping nodes identified through factor loadings >= |.40|. The x-axis indicates factor correlation. The y-axis indicates the mean bias error. Values higher or lower than zero indicate more bias in terms of a higher or lower number of dimensions identified, respectively.


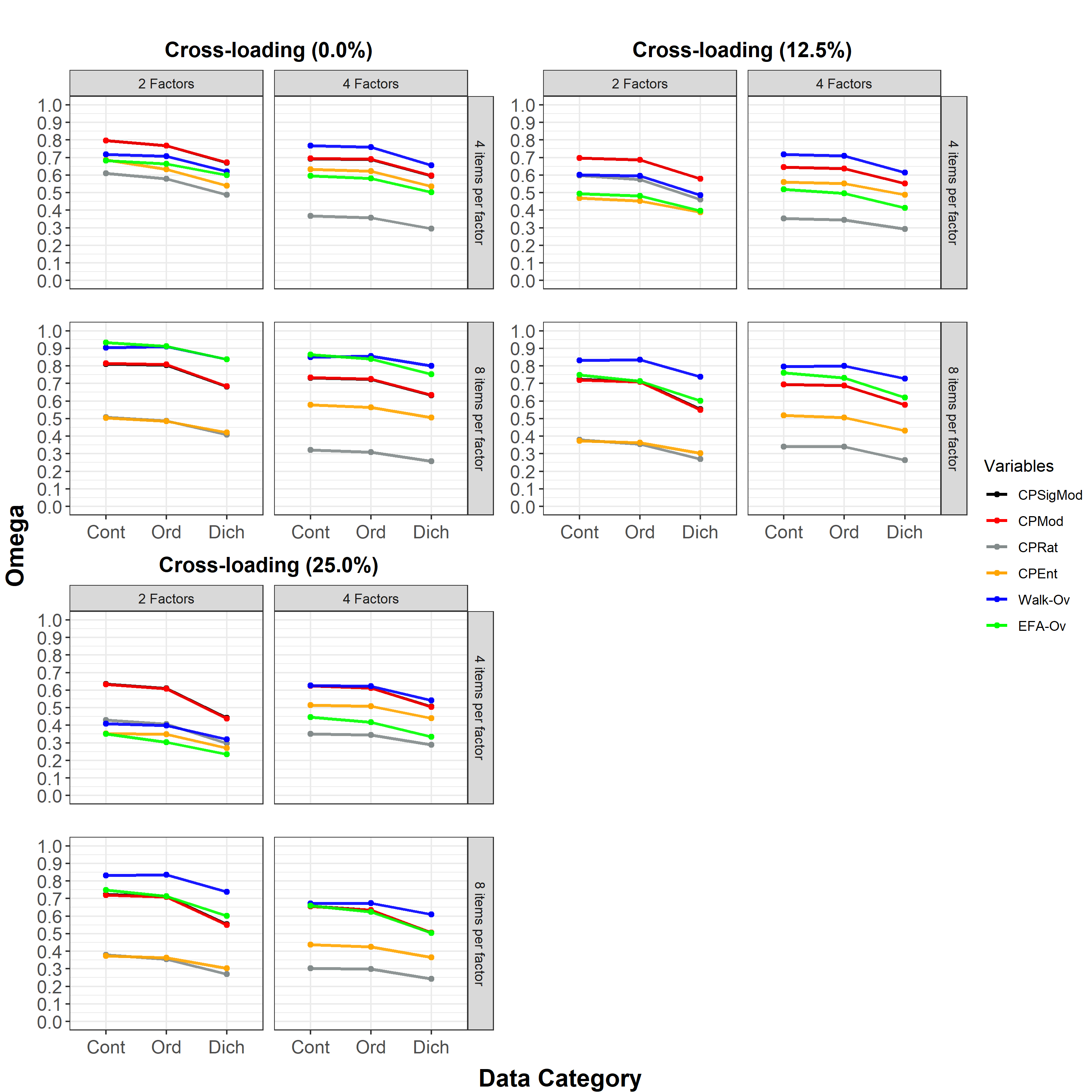


Supplementary Figure 16: Correct node assignment according to data category. Notes. CPSigMod = CP algorithm with maximisation of fuzzy modularity for signed weighted networks; CPMod = CP algorithm with maximisation of fuzzy modularity for weighted networks; CPRat = CP algorithm with minimisation of the ratio between the two largest communities when the ratio is above or equal 2; CPEnt = CP algorithm with maximisation of entropy; Walk-Ov = Walktrap algorithm with overlapping nodes identified through network loadings >= |.15|; EFA-Ov = EFA-Ov = Exploratory Factor Analysis with overlapping nodes identified through factor loadings >= |.40|. The x-axis indicates the data category. The y-axis indicates the Omega index. Higher values of the Omega index indicate more accurate node assignment to communities.


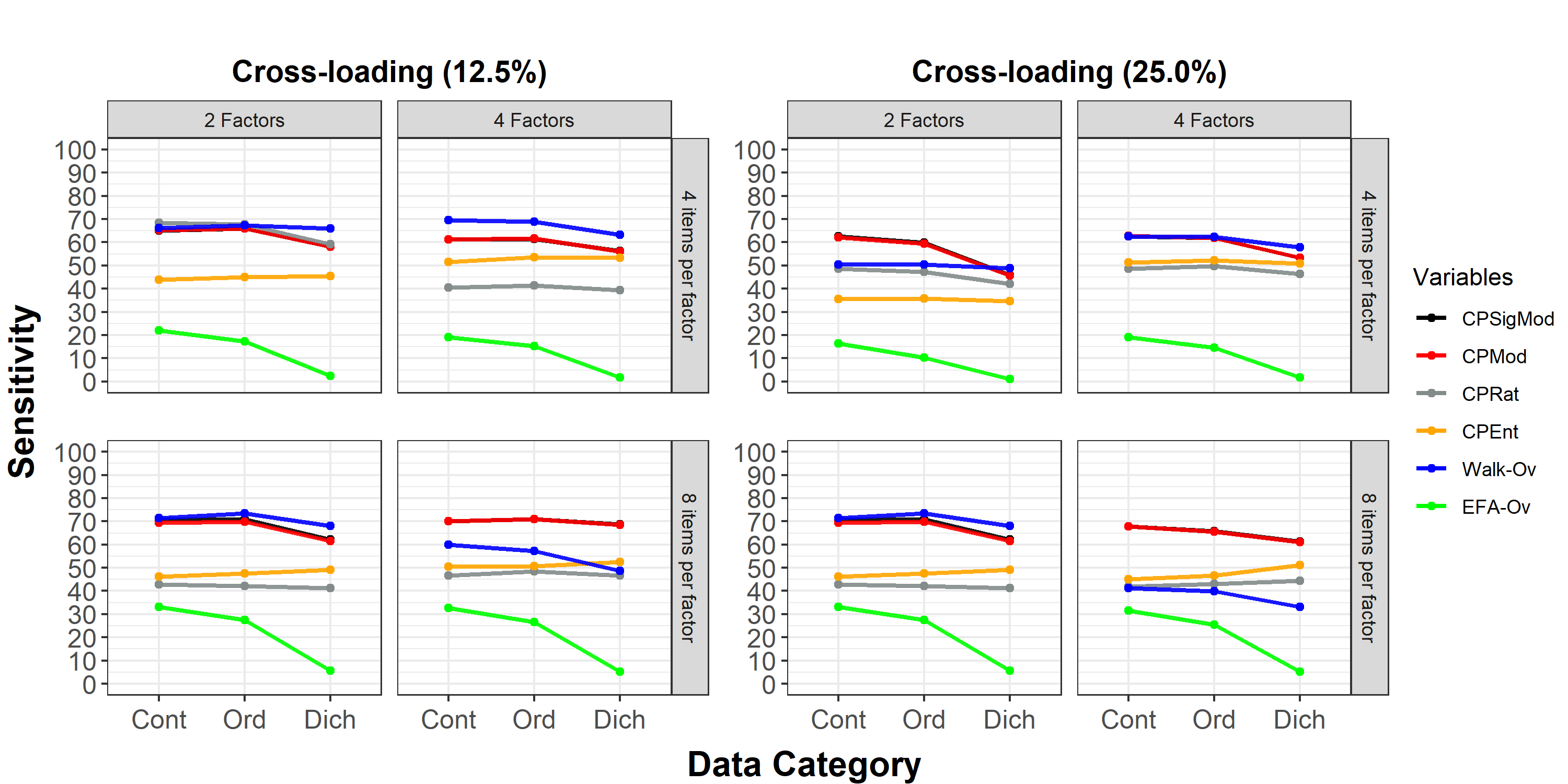


Supplementary Figure 17: Sensitivity according to data category. Note. CPSigMod = CP algorithm with maximisation of the signed fuzzy modularity for signed weighted networks; CPMod = CP algorithm with maximisation of the fuzzy modularity for signed weighted networks; CPRat = CP algorithm with minimisation of the ratio between the two largest communities when the ratio is above or equal 2; CPEnt = CP algorithm with maximisation of entropy; Walk-Ov = Walktrap algorithm with overlapping nodes identified through network loadings >= |.15|; EFA-Ov = Exploratory Factor Analysis with overlapping nodes identified through factor loadings >= |.40|. The x-axis indicates data category. The y-axis indicates the sensitivity. Higher values indicate higher sensitivity of the algorithm to detect overlapping symptoms.


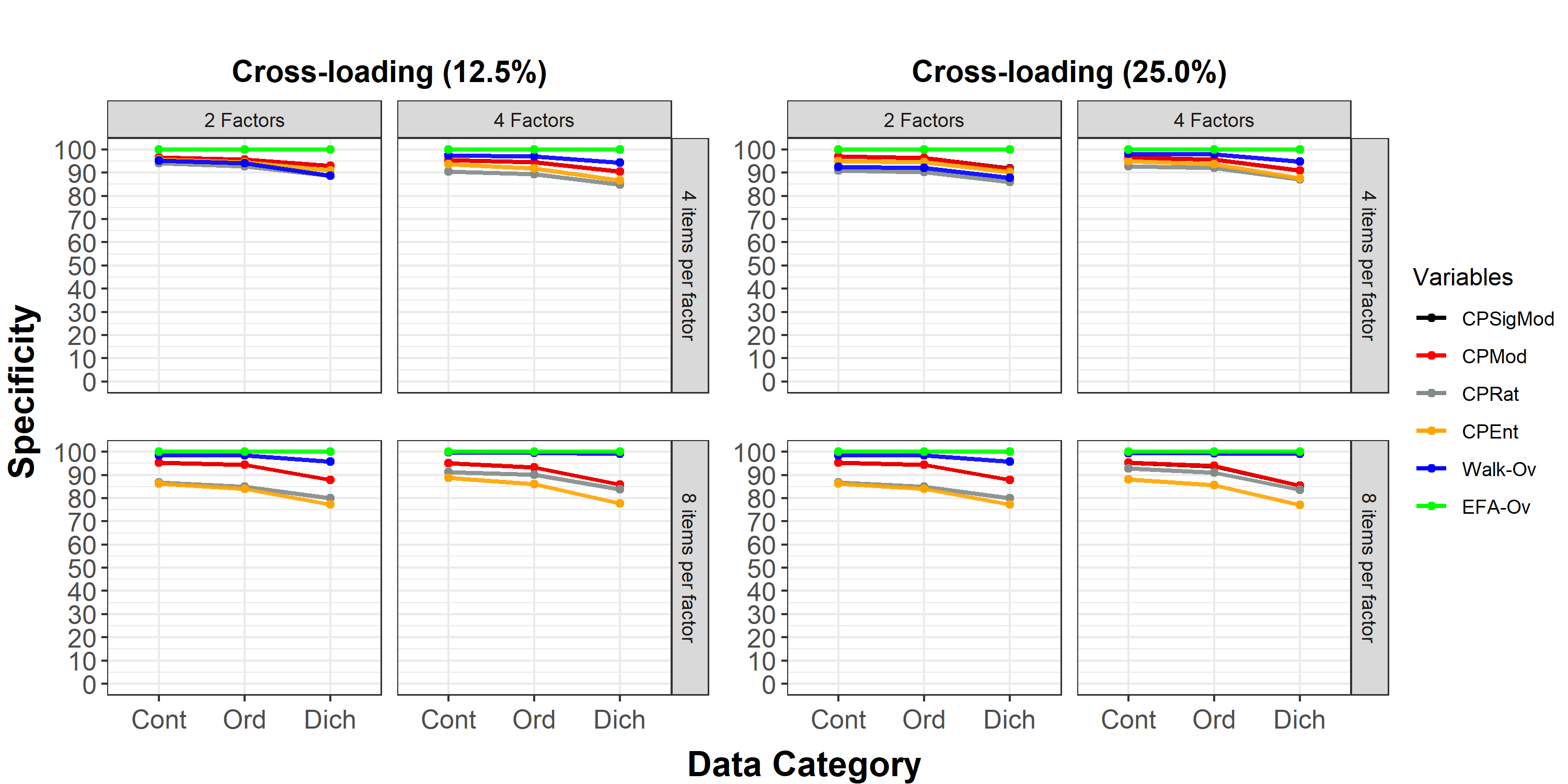


Supplementary Figure 18: Specificity according to data category. Note. CPSigMod = CP algorithm with maximisation of the signed fuzzy modularity for signed weighted networks; CPMod = CP algorithm with maximisation of the fuzzy modularity for signed weighted networks; CPRat = CP algorithm with minimisation of the ratio between the two largest communities when the ratio is above or equal 2; CPEnt = CP algorithm with maximisation of entropy; Walk-Ov = Walktrap algorithm with overlapping nodes identified through network loadings >= |.15|; EFA-Ov = Exploratory Factor Analysis with overlapping nodes identified through factor loadings >= |.40|. The x-axis indicates data category. The y-axis indicates the specificity. Higher values indicate higher specificity of the algorithm to detect non-overlapping symptoms.


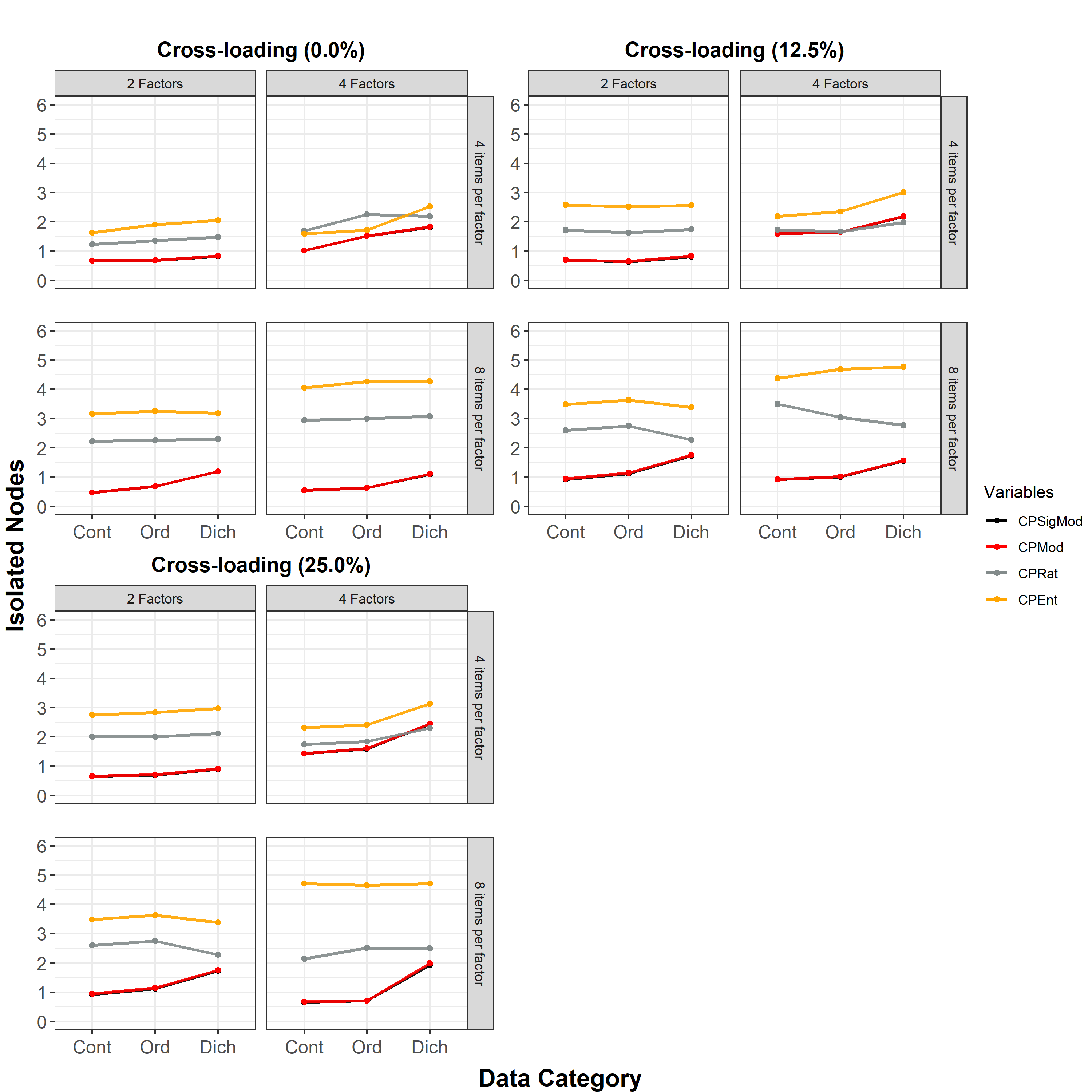


Supplementary Figure 19: Number of isolated nodes according to data category. Note. CPSigMod = CP algorithm with maximisation of the signed fuzzy modularity for signed weighted networks; CPMod = CP algorithm with maximisation of the fuzzy modularity for signed weighted networks; CPRat = CP algorithm with minimisation of the ratio between the two largest communities when the ratio is above or equal 2; CPEnt = CP algorithm with maximisation of entropy; Walk-Ov = Walktrap algorithm with overlapping nodes identified through network loadings >= |.15|; EFA-Ov = Exploratory Factor Analysis with overlapping nodes identified through factor loadings >= |.40|. The x-axis indicates data category. The y-axis indicates the number of isolated nodes. Lower values indicate that a lower number of nodes were not assigned to any community (i.e. isolated nodes) by the algorithm.


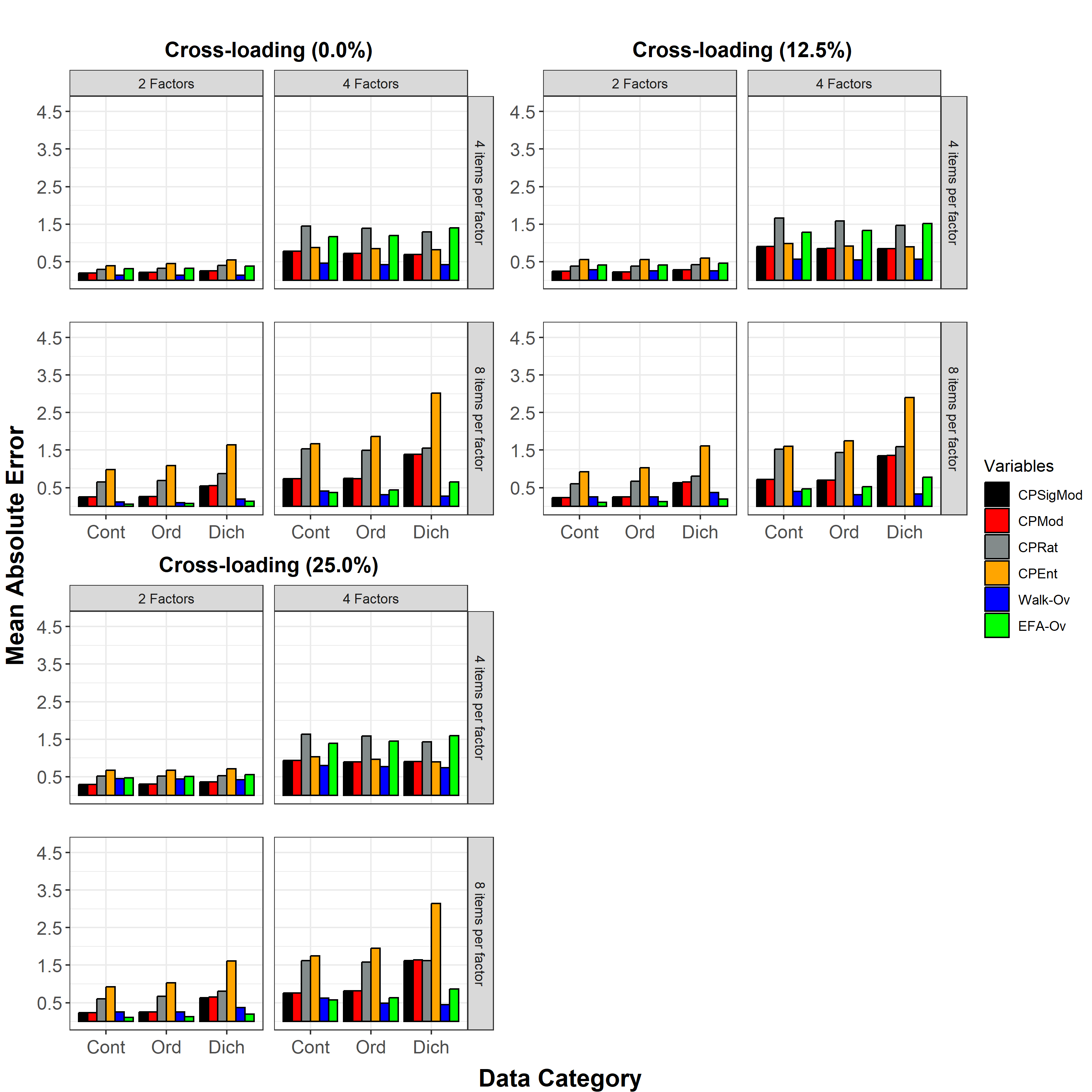


Supplementary Figure 20: Mean absolute error according to data category. Note. CPSigMod = CP algorithm with maximisation of the signed fuzzy modularity for signed weighted networks; CPMod = CP algorithm with maximisation of the fuzzy modularity for signed weighted networks; CPRat = CP algorithm with minimisation of the ratio between the two largest communities when the ratio is above or equal 2; CPEnt = CP algorithm with maximisation of entropy; Walk-Ov = Walktrap algorithm with overlapping nodes identified through network loadings >= |.15|; EFA-Ov = Exploratory Factor Analysis with overlapping nodes identified through factor loadings >= |.40|. The x-axis indicates data category. The y-axis indicates the mean absolute error. Higher values indicate higher absolute error regarding the number of identified dimensions.


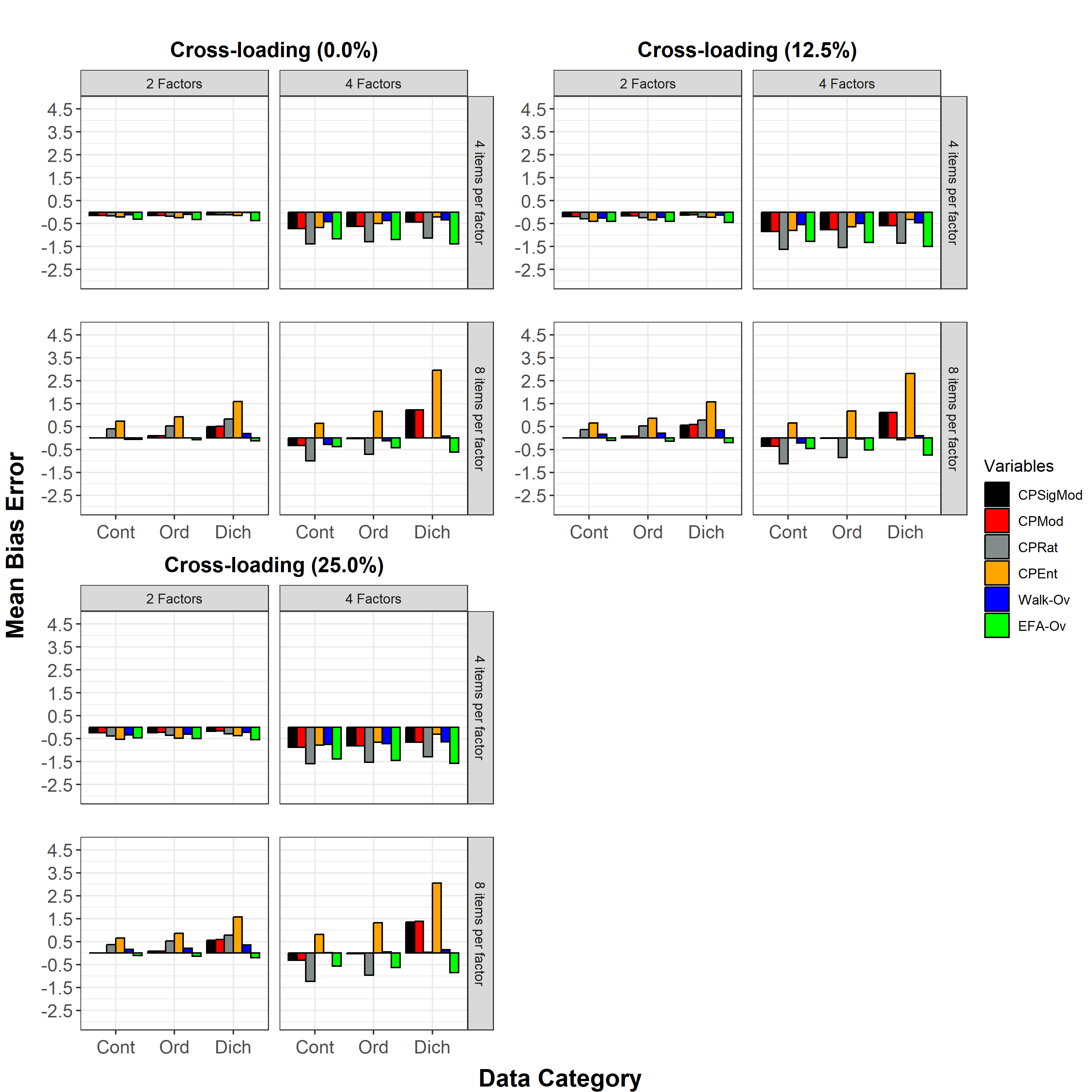


Supplementary Figure 21: Mean bias error according to data category. Note. CPSigMod = CP algorithm with maximisation of the signed fuzzy modularity for signed weighted networks; CPMod = CP algorithm with maximisation of the fuzzy modularity for signed weighted networks; CPRat = CP algorithm with minimisation of the ratio between the two largest communities when the ratio is above or equal 2; CPEnt = CP algorithm with maximisation of entropy; Walk-Ov = Walktrap algorithm with overlapping nodes identified through network loadings >= |.15|; EFA-Ov = Exploratory Factor Analysis with overlapping nodes identified through factor loadings >= |.40|. The x-axis indicates data category. The y-axis indicates the mean bias error. Values higher or lower than zero indicate more bias in terms of a higher or lower number of dimensions identified, respectively.

Supplementary Table 4: Item wording and item label.

| Item wording | Item label |
| --- | --- |
| Considerate of other people’s feelings | considerate |
| Restless, overactive, cannot stay still for long | restless |
| Often complains of headaches, stomach-aches or sickness | somatic |
| Shares readily with other children (treats, toys, pencils etc) | shares |
| Often loses temper | temper |
| Rather solitary, tends to play alone | solitary |
| Generally well behaved, usually does what adults request | obedient |
| Many worries, often seems worried | worries |
| Helpful if someone is hurt, upset or feeling ill | helps |
| Constantly fidgeting or squirming | fidgety |
| Has at least one good friend | friend |
| Often fights with other children or bullies them | fights |
| Often unhappy, depressed or tearful | unhappy |
| Generally liked by other children | popular |
| Easily distracted, concentration wanders | distractible |
| Nervous or clingy in new situations, easily loses confidence | clingy |
| Kind to younger children | kind |
| Often lies or cheats | lies |
| Picked on or bullied by other children | bullied |
| Often volunteers to help others (parents, teachers, other children) | helps |
| Thinks things out before acting | reflective |
| Steals from home, school, or elsewhere | steals |
| Gets along better with adults than with other children | adults |
| Many fears, easily scared | fears |
| Good attention span, see chores or homework through to the end | persistent |
| Note. SDQ for Parents of 4-10 year olds, Australian Version. Copyright Robert Goodman, 1999, UK. | |

Supplementary Table 5: Polychoric correlation matrix of the SDQ for children aged 4-10 years.

|  | considerate | shares | caring | kind | helps | restless | fidgety | distractible | reflective | persistent | somatic | worries | unhappy | clingy | fears | tempers | obedient | fights | lies | steals | solitary | friend | popular | bullied | adults |
| --- | --- | --- | --- | --- | --- | --- | --- | --- | --- | --- | --- | --- | --- | --- | --- | --- | --- | --- | --- | --- | --- | --- | --- | --- | --- |
| considerate | - |  |  |  |  |  |  |  |  |  |  |  |  |  |  |  |  |  |  |  |  |  |  |  |  |
| shares | 0.54 | - |  |  |  |  |  |  |  |  |  |  |  |  |  |  |  |  |  |  |  |  |  |  |  |
| caring | 0.59 | 0.47 | - |  |  |  |  |  |  |  |  |  |  |  |  |  |  |  |  |  |  |  |  |  |  |
| kind | 0.54 | 0.46 | 0.57 | - |  |  |  |  |  |  |  |  |  |  |  |  |  |  |  |  |  |  |  |  |  |
| helps | 0.45 | 0.41 | 0.58 | 0.47 | - |  |  |  |  |  |  |  |  |  |  |  |  |  |  |  |  |  |  |  |  |
| restless | 0.31 | 0.23 | 0.15 | 0.18 | 0.15 | - |  |  |  |  |  |  |  |  |  |  |  |  |  |  |  |  |  |  |  |
| fidgety | 0.30 | 0.19 | 0.14 | 0.16 | 0.14 | 0.81 | - |  |  |  |  |  |  |  |  |  |  |  |  |  |  |  |  |  |  |
| distractible | 0.28 | 0.21 | 0.13 | 0.15 | 0.22 | 0.60 | 0.63 | - |  |  |  |  |  |  |  |  |  |  |  |  |  |  |  |  |  |
| reflective | 0.47 | 0.31 | 0.31 | 0.31 | 0.34 | 0.42 | 0.41 | 0.49 | - |  |  |  |  |  |  |  |  |  |  |  |  |  |  |  |  |
| persistent | 0.35 | 0.28 | 0.22 | 0.24 | 0.31 | 0.52 | 0.53 | 0.75 | 0.55 | - |  |  |  |  |  |  |  |  |  |  |  |  |  |  |  |
| somatic | 0.15 | 0.07 | 0.05 | 0.05 | 0.01 | 0.19 | 0.24 | 0.19 | 0.07 | 0.14 | - |  |  |  |  |  |  |  |  |  |  |  |  |  |  |
| worries | 0.13 | 0.14 | 0.07 | 0.11 | 0.07 | 0.21 | 0.28 | 0.23 | 0.11 | 0.16 | 0.41 | - |  |  |  |  |  |  |  |  |  |  |  |  |  |
| unhappy | 0.29 | 0.25 | 0.13 | 0.24 | 0.08 | 0.28 | 0.33 | 0.29 | 0.19 | 0.27 | 0.42 | 0.58 | - |  |  |  |  |  |  |  |  |  |  |  |  |
| clingy | 0.13 | 0.11 | 0.13 | 0.10 | 0.15 | 0.15 | 0.20 | 0.21 | 0.10 | 0.16 | 0.27 | 0.43 | 0.36 | - |  |  |  |  |  |  |  |  |  |  |  |
| fears | 0.14 | 0.13 | 0.08 | 0.12 | 0.09 | 0.21 | 0.28 | 0.25 | 0.13 | 0.18 | 0.39 | 0.65 | 0.49 | 0.52 | - |  |  |  |  |  |  |  |  |  |  |
| tempers | 0.46 | 0.34 | 0.22 | 0.30 | 0.22 | 0.45 | 0.42 | 0.39 | 0.39 | 0.35 | 0.30 | 0.38 | 0.45 | 0.26 | 0.29 | - |  |  |  |  |  |  |  |  |  |
| obedient | 0.56 | 0.46 | 0.42 | 0.41 | 0.36 | 0.44 | 0.39 | 0.40 | 0.48 | 0.46 | 0.14 | 0.14 | 0.31 | 0.12 | 0.17 | 0.51 | - |  |  |  |  |  |  |  |  |
| fights | 0.51 | 0.37 | 0.29 | 0.33 | 0.19 | 0.42 | 0.42 | 0.38 | 0.42 | 0.34 | 0.18 | 0.23 | 0.44 | 0.19 | 0.19 | 0.56 | 0.50 | - |  |  |  |  |  |  |  |
| lies | 0.40 | 0.28 | 0.23 | 0.27 | 0.15 | 0.35 | 0.35 | 0.37 | 0.39 | 0.34 | 0.26 | 0.22 | 0.36 | 0.16 | 0.23 | 0.47 | 0.46 | 0.53 | - |  |  |  |  |  |  |
| steals | 0.42 | 0.30 | 0.25 | 0.22 | 0.17 | 0.30 | 0.31 | 0.32 | 0.31 | 0.29 | 0.24 | 0.22 | 0.35 | 0.14 | 0.28 | 0.46 | 0.44 | 0.49 | 0.69 | - |  |  |  |  |  |
| solitary | 0.21 | 0.22 | 0.20 | 0.25 | 0.15 | 0.11 | 0.16 | 0.18 | 0.07 | 0.12 | 0.18 | 0.28 | 0.26 | 0.30 | 0.26 | 0.17 | 0.12 | 0.18 | 0.12 | 0.13 | - |  |  |  |  |
| friend | 0.37 | 0.29 | 0.29 | 0.32 | 0.16 | 0.27 | 0.23 | 0.23 | 0.26 | 0.30 | 0.15 | 0.25 | 0.33 | 0.14 | 0.22 | 0.24 | 0.29 | 0.32 | 0.26 | 0.28 | 0.30 | - |  |  |  |
| popular | 0.49 | 0.42 | 0.33 | 0.41 | 0.25 | 0.29 | 0.29 | 0.31 | 0.37 | 0.39 | 0.21 | 0.29 | 0.35 | 0.16 | 0.27 | 0.36 | 0.47 | 0.53 | 0.36 | 0.39 | 0.29 | 0.58 | - |  |  |
| bullied | 0.20 | 0.22 | 0.08 | 0.13 | 0.00 | 0.29 | 0.29 | 0.34 | 0.23 | 0.28 | 0.27 | 0.38 | 0.48 | 0.26 | 0.35 | 0.31 | 0.25 | 0.46 | 0.34 | 0.32 | 0.24 | 0.37 | 0.47 | - |  |
| adults | 0.22 | 0.18 | 0.07 | 0.10 | -0.06 | 0.20 | 0.23 | 0.20 | 0.03 | 0.12 | 0.20 | 0.28 | 0.33 | 0.20 | 0.29 | 0.18 | 0.13 | 0.30 | 0.16 | 0.22 | 0.35 | 0.38 | 0.37 | 0.38 | - |
| Note. The items labels are displayed in Supplementary Table 4. | | | | | | | | | | | | | | | | | | | | | | | | | |

# Appendix A

**The Walktrap algorithm**

The Walktrap algorithm has been the most commonly applied community detection method in network psychometric research. The Walktrap algorithm considers that, given a fixed number of steps, a random walker starting at a certain node will visit another node with a given probability. More formally, the algorithm computes transition probabilities $P_{ij}^{t}=\frac{w_{ij}}{d(i)}$ , where $w_{ij}$ is the edge weight between observed variables *i* and *j*, $d(i)$ indicates the node *i* degree (the number of non-zero connections establish by node *i*) and *t* indicates the number of random walks. These transition probabilities indicate how well two nodes are connected (i.e. stronger transition probabilities indicate that nodes are more strongly connected). The intuition for community detection is that, considering that two nodes are within the same community, their transition probabilities to reach any other node in the network should be similar (e.g. higher probabilities of reaching nodes within the community and lower probabilities of reaching nodes outside of the community). This occurs because the random walks are more likely to be “trapped” inside a network community due to limited paths connecting to nodes belonging to other communities. Hence, a distance measure between two nodes can be calculated as:

$$r_{ij}(t)=\sqrt{\sum_{k=1}^{n} \frac{{(P_{ik}-P_{jk})}^{2}}{d(k)}}$$

( 10 )

, where *n* refers to the number of nodes in the network. The distance $r_{ij}$ considers the differences in transition probabilities between each of the two nodes and all other nodes in the network. For the reasons abovementioned (i.e. nodes within the same community have similar transition probabilities to other nodes in the network), nodes with small distances are more likely to belong to the same community. After distances are calculated for each node pair, the Walktrap algorithm uses Ward’s agglomerative clustering approach, initially considering each node as its own cluster and progressively combining nodes with small distances into larger clusters, ending up in a single cluster containing all nodes (Christensen, Garrido, & Golino, 2021).

# Appendix B

**Data simulation approach**

Following Golino et al. (2020) and Christensen et al. (2021), data was generated from multivariate normal factor models. The reproduced population matrix was computed:

$$R_{R}=\Omega\Phi\Omega'$$

( 11 )

where $R_{R}$ is the reproduced population correlation matrix, $\Omega$ is the *p* (variables) x *r* (factors) factor loading matrix, and the $\Phi$ is the *r* x *r* correlation matrix. The factor loading matrix $\Omega$ incorporates the primary factor loadings, the substantive and equally sized cross-loadings and the non-substantive cross-loadings. As means of illustration, Supplementary Table 6 provides one example of the factor loading matrix $\Omega$ of a factor model with 2 factors, 4 variables per factor, factor correlation of 0.50, primary factor loadings of 0.70, 12.5% of observed variables with substantive cross-loading of 0.50, and non-substantive cross-loadings randomly drawn from a normal distribution ℵ(0.00,0.05) from an observed variable to all other factors:

Supplementary Table 5: Factor loading matrix

| Variable | Factor 1 | Factor 2 |
| --- | --- | --- |
| V1 | **0.70** | -0.01 |
| V2 | **0.70** | 0.02 |
| V3 | **0.70** | -0.02 |
| V4 | **0.50** | **0.50** |
| V5 | 0.01 | **0.70** |
| V6 | -0.06 | **0.70** |
| V7 | 0.08 | **0.70** |
| V8 | 0.09 | **0.70** |

Note. Substantive factor loadings are highlighted in bold. The variable V4 has substantive and equally sized cross-loadings across the two factors, constituting an “overlapping” symptom. Additional examples can be found in the R-shiny app available at https://pedroribeirosantiago.shinyapps.io/cliquepercolation/.

To obtain the population correlation matrix, $R_{P}$, unities were included in the diagonal of $R_{R}$. We then performed a Cholesky decomposition:

$$R_{P}=U'U$$

( 12)

The population correlation matrix was re-generated if the population correlation matrix was not positive definite (i.e. one or more eigenvalues $\leq$ 0) or any item with communality greater than 0.90. Finally, the sample data with continuous variables was computed:

$$X=ZU$$

( 13 )

Where Z is a matrix of random multivariate normal data *n* (sample size) x *p* (number of variables). Following Ferrari and Barbiero (2012), to generate polytomous (5-point Likert scale) and dichotomous variable, we initially calculated the correlation matrix of the discretised variables $R_{O}$ based on the marginal distributions of the discretised variables and the population correlation matrix $R_{P}$ of the continuous variables as implied by the factor model. The marginal distributions were established in terms of cumulative probabilities for polytomous (0.2, 0.4, 0.6, 0.8, 1.0) and dichotomous (0.5, 1.0) variables. We then generated a matrix of multivariate discrete random variables of size *n* (sample size) x *p* (number of variables) based on the marginal distributions of the discretised variables and the correlation matrix of the discretised variables $R_{O}$. The generation of polytomous and dichotomous variables was conducted with the R package GenOrd (Barbiero & Ferrari, 2015).

# References

Barbiero, A., & Ferrari, P. A. (2015). GenOrd: Simulation of ordinal and discrete variables with given correlation matrix and marginal distributions (v. 1.4. 0).

Christensen, A. P., Garrido, L. E., & Golino, H. (2021). Comparing community detection algorithms in psychological data: A Monte Carlo simulation.

Ferrari, P. A., & Barbiero, A. (2012). Simulating ordinal data. *Multivariate Behavioral Research, 47*(4), 566-589.

Golino, H., Shi, D., Christensen, A. P., Garrido, L. E., Nieto, M. D., Sadana, R., . . . Martinez-Molina, A. (2020). Investigating the performance of exploratory graph analysis and traditional techniques to identify the number of latent factors: A simulation and tutorial. *Psychological Methods*.

1. Adelaide Dental School, [pedro.ribeirosantiago@adelaide.edu.au](mailto:pedro.ribeirosantiago@adelaide.edu.au) [↑](#footnote-ref-1)
2. Adelaide Dental School, [gustavo.soares@adelaide.edu.au](mailto:gustavo.soares@adelaide.edu.au) [↑](#footnote-ref-2)
3. Icfes - Colombian Institute for Educational Evaluation, [adrianquintero987@hotmail.com](mailto:adrianquintero987@hotmail.com) [↑](#footnote-ref-3)
4. Adelaide Dental School, [lisa.jamieson@adelaide.edu.au](mailto:lisa.jamieson@adelaide.edu.au) [↑](#footnote-ref-4)
